# Unnatural α-amino ethyl esters from diethyl malonate or ethyl β-bromo-α-hydroxyiminocarboxylate

**DOI:** 10.3762/bjoc.14.264

**Published:** 2018-11-16

**Authors:** Eloi P Coutant, Vincent Hervin, Glwadys Gagnot, Candice Ford, Racha Baatallah, Yves L Janin

**Affiliations:** 1 Unité de Chimie et Biocatalyse, Département de Biologie Structurale et Chimie, Institut Pasteur, 28 rue du Dr. Roux, 75724 Paris Cedex 15, France; 2 Unité Mixte de Recherche 3523, Centre National de la Recherche Scientifique, 28 rue du Dr. Roux, 75724 Paris Cedex 15, France; 3 Université Paris Descartes, Sorbonne Paris Cité, 12 rue de l'École de Médecine, 75006 Paris, France

**Keywords:** α-amino ester, α-hydroxyimino ester, [2 + 4] cycloadditions, [2 + 3] cycloadditions, Knoevenagel, nitrosoacrylate, Suzuki–Miyaura

## Abstract

We have explored here the scope of the age-old diethyl malonate-based accesses to α-amino esters involving Knoevenagel condensations of diethyl malonate on aldehydes, reductions of the resulting alkylidenemalonates, the preparation of the corresponding α-hydroxyimino esters and their final reduction. This synthetic pathway turned out to be general although some unexpected limitations were encountered. The synthetic modifications of some of the intermediates – using Suzuki–Miyaura coupling or cycloadditions – before undertaking the oximation step – provided accesses to further α-amino esters. Moreover, other pathways to α-hydroxyimino esters were explored including an attempt to improve the cycloadditions between ethyl β-bromo-α-hydroxyiminocarboxylate and various alkylfuranes.

## Introduction

**Scheme 1:**
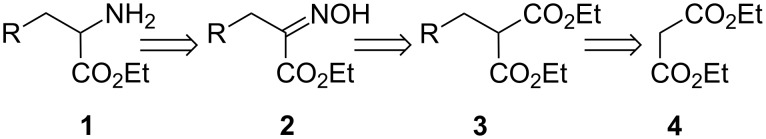
Malonate-based retrosynthesis of α-amino esters.

Our current work on the chemistry of imidazo[1,2-*a*]pyrazin-3(7*H*)-one luciferins [1] has led us to require a large variety of α-amino esters as starting material. In view of the limitations we encountered in the use of ethyl nitroacetate to reach such variety [2], we have focused here on diethyl malonate-based methods as an alternative. Indeed, as we reviewed recently [3], this approach was used for the historic preparation of racemic lysine from diethyl malonate and γ-chlorobutyronitrile [4] and appears to be of a very large scope. The key α-hydroxyimino esters **2** precursors to the α-amino esters **1** are usually prepared by oximation of substituted malonates **3**, themselves made, for instance, from diethyl malonate (**4**) and an alkylation reaction or a Knoevenagel condensation–reduction sequence (Scheme 1).

## Results and Discussion

**Scheme 2:**
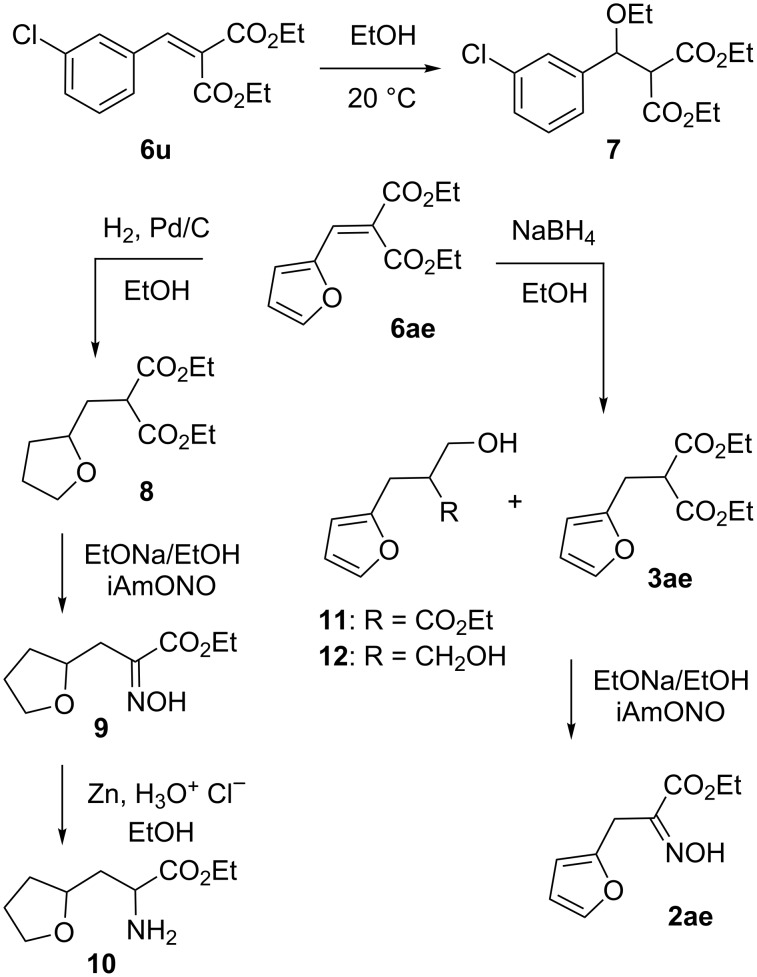
Some side products and synthesis of α-amino ester **10**.

As depicted in Table 1, Knoevenagel condensations of diethyl malonate (**4**) and aldehydes **5a**–**al** followed by reduction of the intermediate alkylidenemalonates **6a**–**al** led to the substituted malonates **3a**–**al**. A simple and general procedure was adopted for the condensation using fairly concentrated ethanolic solutions of diethyl malonate (**4**) and aldehydes **5a**–**al** along with catalytic amounts of piperidine and acetic acid as well as some 4 Å molecular sieves to trap the water formed in situ. The ^1^H NMR monitoring of crude samples pointed out a complete conversion, most often overnight at 60 °C, and the resulting solutions of alkylidenemalonates **6a**–**al** were then directly reduced to give the malonates **3a**–**al**. For this reduction step, palladium-based catalytic hydrogenation was preferably used although, when incompatible with the substrates, sodium borohydride was employed. In most cases, large proportion of the expected substituted malonates **3a**–**al** were observed by ^1^H NMR. Thus, in order to simplify even further this procedure, these crude malonates were subjected to the next step after a minimal work-up. Accordingly, they were dissolved in ethanol and treated first with sodium ethoxide followed by isoamyl nitrite (iAmONO) to give the corresponding α-hydroxyimino esters **2a**–**al**. These resulting compounds were then purified and isolated in yields reported in Table 1. These values usually reflected the eventual problems encountered in the course of the reduction or the oximation steps since the condensation was very often more than 98% complete. As further commented on and depicted in Scheme 2, in few instances (preparations of **2t**–**v** and **2ae**), explanations were found for the low yield observed and could be sometimes acted upon. In any case, the final reduction of the α-hydroxyimino esters **2b**–**al** using zinc and hydrochloric acid in ethanol gave the α-amino esters **1b**–**al**, which were usually of a high purity grade without recourse to a chromatography. In few cases, only a sample of the intermediate α-hydroxyimino esters was purified for analytical purposes. For instance, pure α-amino esters **1m** and **1y** were obtained in four steps from the corresponding aldehydes **5m** and **5y** without the recourse to any chromatography in quite acceptable overall yields.

**Table 1:** Synthesis of α-amino esters **1b**–**al** via α-hydroxyimino esters **2a**–**al**.

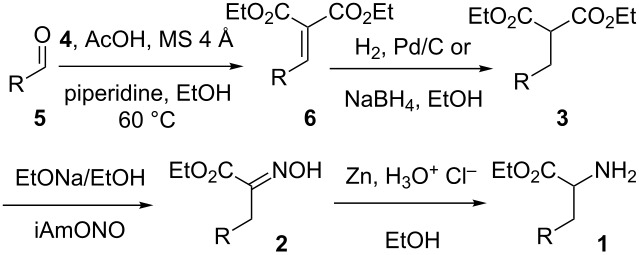			

Ar	R	% **2**^a^	% **1**

**a**	Ph	60	nd
**b**	2-MeC_6_H_4_	61	85
**c**	3-MeC_6_H_4_	63	85
**d**	4-MeC_6_H_4_	60	87
**e**	4-iPrC_6_H_4_	48	91
**f**	cyclopentyl	35	85
**g**	cyclohexyl	50	92
**h**	2-CF_3_C_6_H_4_	39	86
**i**	3-CF_3_C_6_H_4_	41	78
**j**	4-CF_3_C_6_H_4_	62	91
**k**	2-FC_6_H_4_	57	88
**l**	3-FC_6_H_4_	65	89
**m**	4-FC_6_H_4_	–	81^b^
**n**	2,4-F_2_C_6_H_3_	50	85
**o**	2,6-F_2_C_6_H_3_	54	94
**p**	2,3-F_2_C_6_H_3_	51	92
**q**	2,5-F_2_C_6_H_3_	50	95
**r**	3,5-F_2_C_6_H_3_	46	84
**s**	2,3,5-F_3_C_6_H_2_	35	94
**t**	2-ClC_6_H_4_	23/49^c^	94
**u**	3-ClC_6_H_4_	26/59^c^	92
**v**	4-ClC_6_H_4_	37/62^c^	85
**w**	4-BrC_6_H_4_	42	86
**x**	2-MeOC_6_H_4_	60	83
**y**	3-MeOC_6_H_4_	–	75^b^
**z**	2-BnOC_6_H_4_	48	91
**aa**	3-BnOC_6_H_4_	37	92
**ab**	4-BnOC_6_H_4_	40	94
**ac**	2-pyridyl	49	56
**ad**	3-pyridyl	43	63
**ae**	furan-2-yl	33/47^d^	89
**af**	5-methylfuran-2-yl	33	88
**ag**	4,5-dimethylfuran-2-yl	10/23^e^	90
**ah**	5-ethylfuran-2-yl	34	94
**ai**	5-trifluoromethylfuran-2-yl	36	95
**aj**	5-ethylthiophen-2-yl	29	92
**ak**	3-methylthiophen-2-yl	32	89
**al**	4,5-dimethylthiophen-2-yl	31	87

^a^Isolated yield from aldehydes **5**. ^b^Overall isolated yield from aldehydes **5m** or **5y**. ^c^Reactions run in isopropanol and NMe_4_BH_4_ used as a reductant. ^d^Reduction run at –10 °C for 90 minutes. ^e^Same as note d but using a THF/ethanol mixture.

Out of these results, we first sought an explanation for the modest yields observed for the preparation of the 2, 3 or 4-chlorophenyl-α-hydroxyimino esters **2t**–**v**. As depicted in Scheme 2, the ^1^H NMR monitoring of the ethanolic solution of the alkylidenemalonate **6u** pointed out a slow but steady 1,4-addition of ethanol to give compound **7** (a 30% conversion was observed over 3 weeks). Moreover, even if a fresh solution of compound **6u** was immediately treated with sodium borohydride, a proportion of this 1,4-adduct was seen in the reaction mixture amongst other side products. We also tried to allow for an eventual reversal of the ethanol addition and thus increased the reaction time of the reduction step, but compound **7** remained unaffected and even more side products arose. To avoid this 1,4-addition, we then resorted to use a less nucleophilic solvent, and tried isopropanol instead of ethanol. However, even if the ensuing reduction of **6u** using sodium borohydride led to a somewhat better yield of compound **3u** (as estimated by ^1^H NMR), side products resulting from over-reductions of the ester moieties were still observed. It is only when switching to tetramethylammonium borohydride that this was alleviated and the yields of α-hydroxyimino esters **2t**–**v** reached 49, 59 and 62% from aldehydes **5t**–**v**, respectively. Of note is that we also tried this reducing agent to improve the overall yield of the benzyloxy-bearing oxime **2ab**, but to no avail. We next focused on the furan-bearing α-hydroxyimino esters such as compound **2ae**. First of all, reduction of the alkylidenemalonate **6ae** using a palladium-based catalytic hydrogenation also led to the concomitant hydrogenation of the furan ring to give compound **8**. As depicted, this actually allowed us to prepare to the 2-tetrahydrofuranyl-bearing α-aminoester **10** in a 35% overall yield via α-hydroxyimino ester **9**, but it also forced us to use borohydrides to avoid the furan ring hydrogenation. Accordingly, an extensive study of the reduction of compound **6ae** with borohydrides was made. Trials included reactions run at 0 °C overnight, the use of wet ethanol or dry THF, the addition of less than one equivalent of the sodium borohydride as well as the use of tetramethylammonium borohydride or sodium cyanoborohydride, but none were overly successful. Indeed, a representative assay (dry THF, 24 hours at 0 °C, 0.7 equivalents of NaBH_4_) led to the isolation of 49% of compound **3ae**. Moreover, extensive purification procedures of the complex reaction mixtures led to the full characterization of compound **11** and **12** stemming from over-reduction. Interestingly, the latter one has actually been reported to occur upon a 48 hour long sodium borohydride reduction of *S*,*S*-diethyl 2-(furan-2-ylmethylene)propanebis(thioate), the bisthioester homolog of compound **6ae** [5]. Finally, the oximation of the pure malonate **3ae** only led to a 64% yield of the corresponding α-hydroxyimino ester **2ae**, thus accounting for the 33% overall yield reported in Table 1 when we proceeded without purification. It is only later that we found that shortening the reaction of the sodium borohydride reduction of **6ae** down to 1.5 hours and lowering the temperature to –10 °C led to a sizable improvement in the overall yield (47% instead of 33%). However, these conditions were a trade-off between reduction of **6ae** into **3ae** and over-reduction since ^1^H NMR analysis of this trial pointed out the coexistence of unreacted material **6ae** along with (traces) of the over-reduced compounds **11** and **12**. We applied this finding to the reduction of **6ag** and had to allow for the low solubility of this compound in ethanol and thus run the reaction in a mixture of ethanol and THF. In any case, we could obtain more of the corresponding α-hydroxyimino ester **2ag** but in a still very modest 23% overall yield. This last result, likely due to additional problems at the oximation stage, is thus only emphasizing the vagaries of this synthetic access that will sometime require a specific study.

**Scheme 3:**
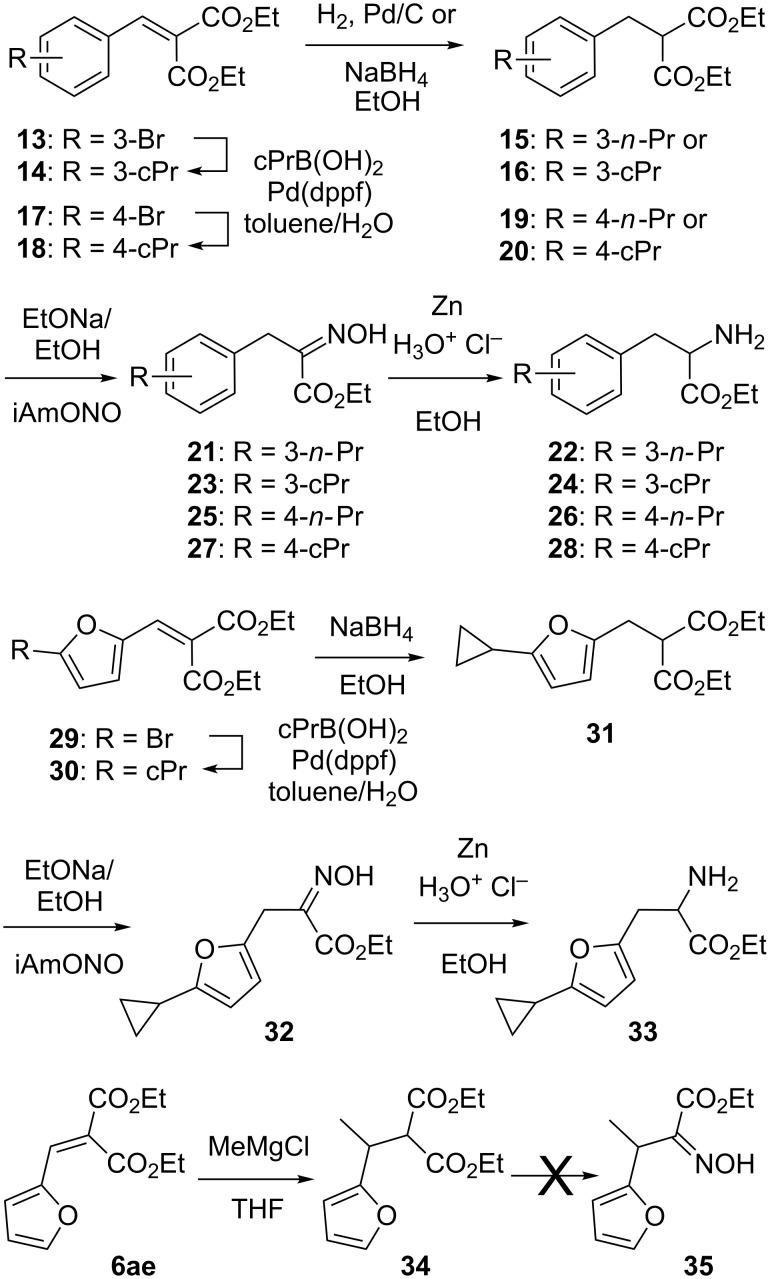
Syntheses of α-amino esters **22**, **24**, **26**, **28** and **33**.

As shown in Scheme 3, the lack or the cost of some more exotic aldehydes **5** were circumvented as we found out that Suzuki–Miyaura carbon–carbon coupling reactions could be achieved with the brominated alkylidenemalonates **13** or **17** as well as the 5-bromofuran derivative **29**. Indeed, coupling these compounds with cyclopropylboronic acid gave the corresponding cyclopropyl derivatives **14**, **18** or **30** in 60, 47 and 75% yield, respectively. The palladium-based reduction of compound **14** and **18** also led to the hydrogenation of their cyclopropyl ring into a propyl side chain to give compounds **15** and **19**. Alternatively, the recourse to sodium borohydride reduced these compounds as well as the furan-bearing derivative **30** into the target cyclopropyl-bearing derivatives **16**, **20** and **31**. The ensuing oximation of all these compounds provided the α-hydroxyimino esters **21**, **23**, **25**, **27** and **32** in 31–88% overall yield. These were then reduced into the corresponding α-amino esters **22**, **24**, **26**, **28** and **33** using zinc and hydrochloric acid. We also focused on the preparation of the β-methylated furan-bearing α-hydroxyimino ester **35**. The introduction of the methyl group was achieved using the previously reported methylmagnesium chloride 1,4-addition on diethyl furfurylidenemalonate (**6ae**) [6]. Quite unexpectedly, repeated attempts to obtain the α-hydroxyimino ester **35** from the resulting β-methylated derivative **34** failed. Trials were made not only with sodium ethoxide but also with the stronger lithium diisopropylamide. We do not have an explanation for this observation, although such oximation was achieved (in a low yield) when starting from the phenyl-bearing analogue **36** as described below. We suggest a somehow forbidding cation chelation by the oxygen of the furan ring which would prevent its reaction with isoamyl nitrite. Interestingly, a literature search for similar reactions starting from β,β-disubstituted malonates, led to two reports [7–8], whereas from β,β-substituted acetoacetates three examples were reported [9–11], but only one substrate [7] out of these features an aromatic substituent.

**Scheme 4:**
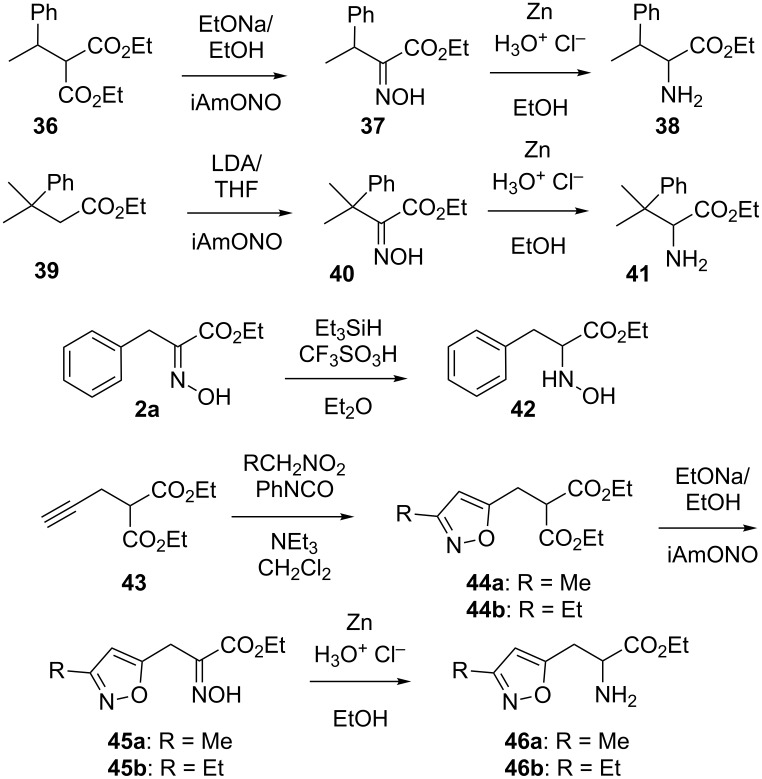
Syntheses of α-amino esters **38**, **41** and **46a**,**b**.

As depicted in Scheme 4 and contrary to our attempt to prepare the furan-bearing β-methyl oxime **35**, the phenyl-bearing β-methyl aminoester **38** was obtained via the substituted malonate **36** to give upon oximation the α-hydroxyimino ester **37** (although in only a 14% yield). A slightly different approach was used for the preparation of the even more hindered β,β-dimethyl aminoester **41**. Treatment of the monoester **39** [12–13] with lithium diisopropylamide and isoamyl nitrite overnight was once again not really successful as a rather poor 15% yield of the corresponding α-hydroxyimino ester **40** was isolated. Nevertheless, the ensuing reduction gave the desired α-amino esters **38** and **41**. The model reduction of the α-hydroxyimino ester **2a** into the corresponding *N*-hydroxylamino ester **42** was also studied. Initial trials using reported procedures [14–16] pointed out an exceedingly slow reaction requiring repeated additions of trimethylamine–borane complex on a solution of compound **2a** in diethyl ether saturated with hydrogen chloride (ether containing trifluoromethanesulfonic acid was also tried). This led only to a 40% yield probably because of the occurrence of stable boron-based complexes. In an attempt to improve this, we applied the reported use of triethylsilane for reducing oximes into *N*-hydroxylamines [17] to the case of the α-hydroxyimino ester **2a**. However, bringing the reduction of **2a** into the *N*-hydroxylamino derivative **42** to completion with this reagent still required a repeated addition of triethylsilane over a week and led to a 69% isolated yield. Interestingly, further literature search pointed out a very different synthetic access to such *N*-hydroxylamine derivative (via nitrones) which appears to be far more efficient [18–22]. The isoxazole-bearing α-amino esters **46a**,**b** were also prepared via α-hydroxyimino esters. Their preparation started with the alkylation of diethyl malonate (**4**) with propargyl bromide to give compound **43** (which could not be separated from unreacted malonate and the bis-alkylated derivative). In the next step, [2 + 3] cycloaddition reactions with nitroethane or 1-nitropropane gave the isoxazole-bearing compounds **44a**,**b** in 23 and 33% yield, respectively. Then, oximation of these compounds gave the α-hydroxyimino esters **45a**,**b** in 50 and 58% and upon their reduction, the expected α-amino esters **46a**,**b**.

As depicted in Table 2, in order to avoid the recourse to the sometime expensive furan-bearing aldehydes **5** and reach an even more diverse set of α-amino esters, we investigated the use of ethyl β-bromo-α-hydroxyiminocarboxylate **47** which has been developed by Gilchrist for the synthesis of many types of α-hydroxyimino esters [23–26] and then extensively used to prepare a wide range of amino acids [3,27]. This proceeds via a [2 + 4] cycloaddition between ethyl nitrosoacrylate, generated in situ from the reaction of sodium carbonate and furan **48**, to give the cycloadduct **49**. Then, upon heating, a rearrangement leads to the furan-bearing α-hydroxyimino esters **2**. Reports have thus described the preparation of **2ae** in a 46% yield from furan (**48ae**) [24] and compound **2af** in a 50% yield from 2-methylfuran (**48af**) [25]. For our part, we first made extensive trials to improve such yields using 2-methylfuran (**48af**) as a model substrate. It turned out that this [2 + 4] cycloaddition can proceed under a wide range of conditions. The original conditions stirring compound **47**, an excess of 2-methylfuran (**48af**) and solid sodium carbonate in dichloromethane for 24 hours gave the expected cycloadduct **49af** but we found out that a chromatography was enough to achieve its isomerisation into **2af**. Interestingly, we also found out that the excess 2-methylfuran (**48af**) was not required and that the reaction proceeded much faster and led directly to the α-hydroxyimino ester **2af** if tetrabutylammonium bromide was added as a phase transfer catalyst. For instance, (note b in Table 2), stirring a 1:1.1 proportion of compounds **47** and **48af**, sodium carbonate, and 0.01 equiv tetrabutylammonium bromide in toluene for 1.5 hours led to a 42% yield of the α-hydroxyimino ester **2af**. On the other hand, such catalysis was not required when stirring a 1:1.1 proportion of compounds **47** and **48af** in a mixture of ethyl acetate and water and an array of bases such as ammonium bicarbonate (41% yield, note c in Table 2). We tried under these conditions 1:2 or 2:1 proportions of compounds **47** and **48af** but the isolated yields of α-hydroxyimino ester **2af** were of (only) 56% and 54%, respectively. Later on, when using a 1:1 proportion of compounds **47** and **48af**, a slightly improved yield of 50% was achieved when switching from ammonium bicarbonate to lithium carbonate (note d in Table 2) or in the case of the preparation of compound **2ag** switching to sodium carbonate (note e in Table 2). Unfortunately, if the ^1^H NMR analysis of another chromatographic fraction obtained when purifying these trials pointed out the occurrence of inseparable mixture of aliphatic compounds, these could never be properly purified and identified. A mechanistic explanation for these average yields could be the occurrence upon the base reaction with compound **47** of *cis* and *trans* conformations of the nitrosoacrylate. The *cis* one would be susceptible to undergo an immediate [2 + 4] cycloaddition with the furan whereas the *trans* form would not, and since the equilibration between the two forms was reported [28] to be a slow process, it would potentially lead to decomposition materials. In any case, as depicted in Table 2, from the commercially available furans **48af**–**an** or from crude solutions of the less accessible (and volatile) compounds **48ao**–**ar** (their preparation greatly benefitted from the reported [29] synthesis of compound **48ap** and is fully described in the experimental section), we could isolate the corresponding α-hydroxyimino esters using a variety of conditions which were then reduced into the α-amino esters. Of note is that extensive decomposition was observed when starting from 2-methoxyfuran and as seen by ^1^H NMR, no oxime occurrence was observed from 2,3,4-trimethylfuran.

**Table 2:** Synthesis of furan-bearing α-amino esters by a [2 + 4] cycloaddition.

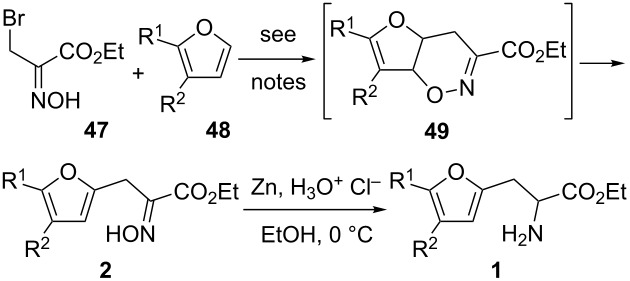				

Ar	R^1^	R^2^	% **2**^a^	% **1**^a^

**ae**	H	H	–	–
**af**	Me	H	42^b^/41^c^/50^d^	–
**ag**	Me	Me	38^b^/48^c^/50^e^	–
**ah**	Et	H	43^b^	–
**am**	*n-*Pr	H	40^b^	79
**an**	*n-*pentyl	H	32^c^	90
**ao**	Me	Et	21^c,f^	82
**ap**	(CH_2_)_4_		40^b,g^	82
**aq**	Et	Me	25^c, h^	90
**ar**	iPr	Me	30^c,i^	86

^a^Isolated yield. ^b^Toluene, NBu_4_Br (cat.), Na_2_CO_3_. ^c^H_2_O/AcOEt, NH_3_/HCO_3_H. ^d^H_2_O/AcOEt, Li_2_CO_3_. ^e^H_2_O/AcOEt, Na_2_CO_3_. ^f^Overall yield from 1-(2-methylfuran-3-yl)ethan-1-one. ^g^Overall yield from 6,7-dihydrobenzofuran-4(5*H*)-one. ^h^Overall yield from ethyl 2-ethylfuran-3-carboxylate. ^i^Overall yield from ethyl 2-isopropylfuran-3-carboxylate.

**Scheme 5:**
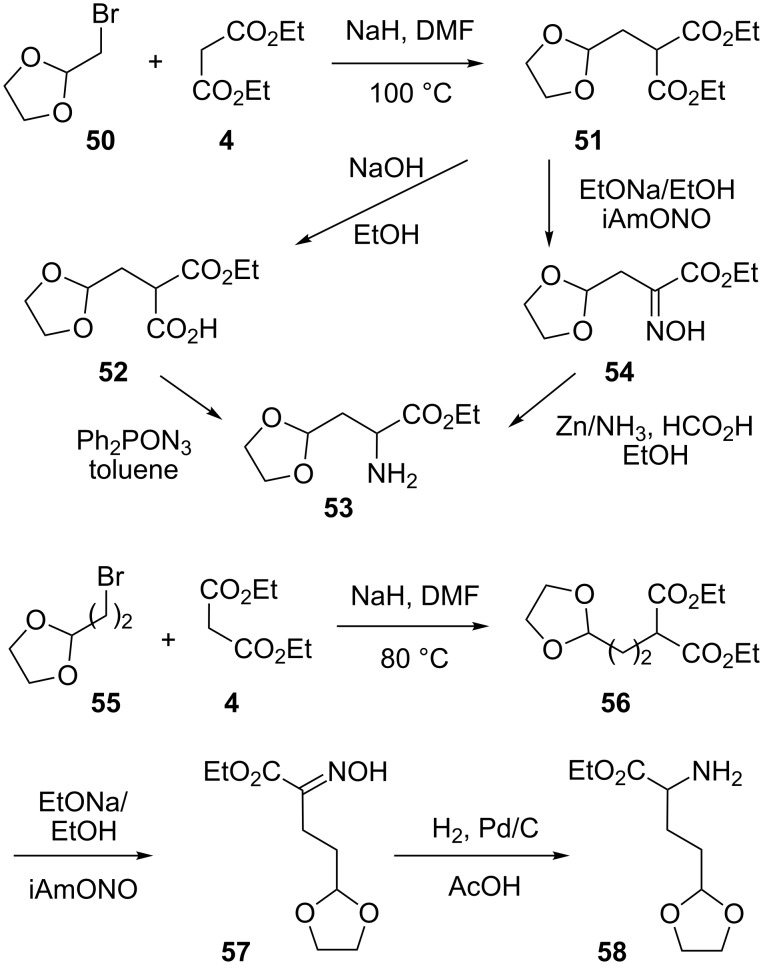
Syntheses of α-amino esters **53** and **58**.

Finally, as described in Scheme 5, we also prepared the dioxolane-bearing α-amino esters **53** and **58**. An approach involving a key Curtius reaction was first tried for the preparation of **53**. As reported [30], alkylation of diethyl malonate (**4**) with 2-(bromomethyl)-1,3-dioxolane (**50**) gave the diester **51**. A controlled saponification led to the mono acid **52** and its reaction with diphenylphosphoryl azide [31–33] gave 16% of the target aminoester **53** upon treatment with hydrochloric acid. Alternatively, oximation of the diester **51** gave 28% (from diethyl malonate) of the α-hydroxyimino ester **54**, which turned out to be not stable in CDCl_3_ because of hydrogen chloride traces. For this reason, no attempt was made to reduce its oxime function under the acidic conditions used above (hydrochloric and powdered zinc) and we successfully used instead the much milder [34] mixture of ammonium formate and zinc in ethanol at room temperature to give 43% of the target α-amino ester **53**. The same strategy gave the corresponding α-amino ester homolog **58** from the readily available dioxane derivative **56** [35] in a 46% overall yield. However, the final oxime reduction turned out to be much improved when using hydrogen and palladium over charcoal in acetic acid.

## Conclusion

As in our previous report [2], the goal of this investigation was to reach a very large variety of racemic α-amino esters. In the course of this study, we could point out that, in comparison with ethyl nitroacetate [2], the use of diethyl malonate (**4**) is at least as efficient and far more general, especially for the initial condensation step. However, as for the reduction of nitroacrylates [2], the use of borohydrides to reduce some of the resulting alkylidenemalonates **6** turned out to be limiting, even in some cases, tetramethylammonium borohydride instead of sodium borohydride could alleviate this limitation. Besides, out of the many preparations of α-hydroxyimino esters described above, another limitation was encountered when steric hindrance became a previously unreported limiting factor for the oximation of compound **34**, and to a lesser degree compounds **36** or **39**. Finally, the use of the ethyl β-bromo-α-hydroxyimino carboxylate **47** in a [2 + 4] cycloaddition reaction led us to suggest a simple, reliable and robust alternative for the preparation of many furan-bearing α-amino esters.

## Supporting Information

File 1 Characterization of all the compounds described, scans of ^1^H and ^13^C NMR spectra.
