# Supplementary material for: Unnatural α-amino ethyl esters from diethyl malonate or ethyl β-bromo-α-hydroxyiminocarboxylate
Source: Beilstein J Org Chem. 2018 Nov 16;14:2853–60. doi: 10.3762/bjoc.14.264 (PMC6244313; doi:10.3762/bjoc.14.264)
Supplement: File 1 — Characterization of all the compounds described, scans of 1H and 13C NMR spectra. [file Beilstein_J_Org_Chem-14-2853-s001.pdf]

BEILSTEIN JOURNAL OF ORGANIC CHEMISTRY

Supporting Information

for

Unnatural α-amino ethyl esters from diethyl malonate or ethyl β-bromo-α-hydroxyiminocarboxylate

Eloi P. Coutant, Vincent Hervin, Glwadys Gagnot, Candice Ford, Racha Baatallah and Yves L. Janin

Beilstein J. Org. Chem. **2018,** *14,* 2853–2860. doi:10.3762/bjoc.14.264

Characterization of all the compounds described, scans of ^{1}H and ^{13}C NMR spectra

License and Terms: This is a supporting information file under the terms of the Creative Commons Attribution License (http://creativecommons.org/licenses/by/4.0). Please note that the reuse, redistribution and reproduction in particular requires that the authors and source are credited.
The license is subject to the *Beilstein Journal of Organic Chemistry* terms and conditions: (https://www.beilstein-journals.org/bjoc)

Experimental

The ^{1}H NMR and ^{13}C NMR spectra were recorded on a Bruker Avance 400 spectrometer at 400 MHz and 100 MHz, respectively. Shifts (δ) are given in ppm with respect to the TMS signal and cross-coupling constants (J) are given in Hertz. Column chromatography were performed either on Merck silica gel 60 (0.035 - 0.070 mm) or neutral alumina using a solvent pump and an automated collecting system driven by a UV detector set to 254 nm unless required otherwise. Sample deposition was carried out by absorption of the mixture to be purified on a small amount of the solid phase followed by its deposition of the top of the column. The low resolution mass spectra were obtained on an Agilent 1200 series LC/MSD system using an Agilent Jet-Stream atmospheric electrospray ionization system and the high resolution mass spectra (HRMS) were obtained using a Waters Micromass Q-TOF with an electrospray ion source.

General preparation of α-hydroxyimino esters 2a–aj: Step 1, Knoevenagel condensation of diethyl malonate and aldehydes, preparation of substituted diethyl 2-methylenemalonate **6**. Under an inert atmosphere, the corresponding aldehyde (0.047 mol), diethyl malonate (7.54 g, 0.047 mol), acetic acid (0.14 g, 0.002 mol), piperidine (0.2 g, 0.002 mol) and 4 Å molecular sieve (15 g) were heated in dry ethanol (20 mL, dried over 4 Å molecular sieves) without stirring at 60 °C for 10 hours. Unless stated below in particle cases, the ^{1}H NMR spectra of a crude sample pointed out the complete disappearance of the starting aldehyde. Step 2, reduction of the substituted diethyl 2-methylenemalonates **6**, preparation of the substituted malonates **3**. The suspension containing the 4 Å molecular sieve described above was filtered and the solid washed with dry ethanol. When relevant, 10% palladium over charcoal (1.27 g, 0.001 mol) was added to the resulting ethanol solution (160 mL total volume) and this was stirred overnight under a hydrogen atmosphere (1 atm). The resulting suspension was filtered, the filtrate concentrated to dryness to give fairly pure substituted malonates **3**. Alternatively, this hydrogenation was achieved by the addition, at 0 °C, of

sodium boron hydride (1.1 equiv) in small portions to the ethanolic solution and stirring overnight at 0 °C. This was followed by its (cautious) acidification with acetic acid, dilution in an excess of water extraction with ethyl acetate. The organic layer was then washed with water, brine, dried over magnesium sulfate and concentrated to dryness to yield the crude substituted malonates **3**. Step 3, preparation of α-hydroxyimino esters **2**. The crude substituted malonate (0.0422 mol) was dissolved in ethanol (60 mL, dried over 4 Å molecular sieve) and cooled to 0 °C. A 21% solution of sodium ethoxide in ethanol was added (18.4 mL, 0.0493 mol) followed by a slow addition of isoamyl nitrite (6.2 mL, 0.046 mol). This was stirred at 0 °C for two hours, made slightly acid by the addition of 1 N hydrochloric acid and diluted in water (200 mL). The solution was extracted with ethyl acetate, the organic layer was washed with water, brine, dried over magnesium sulfate and concentrated to dryness to yield the crude α-hydroxyimino esters **2** which were further purified as described below.

Ethyl 2-(hydroxyimino)-3-phenylpropanoate (2a): Obtained as a white solid (3.55 g, 60% from 3 g of benzaldehyde) after a chromatography over silica gel (cyclohexane – ethyl acetate 4/1). ^{1}H NMR ($CDCl_3$): 9.76 (s, 1H, OH), 7.29 (m, 4H), 7.22 (m, 1H), 4.29 (q, 2H, *J* = 7.1 Hz), 3.99 (s, 2H), 1.32 (t, 3H, *J* = 8.0 Hz). ^{13}C NMR ($CDCl_3$): 163.2, 151.0, 135.7, 129.2, 128.5, 126.7, 61.9, 30.5, 26.9, 14.0. HRMS (*m/z*): $[M+Na]^+$ calcd for $C_{11}H_{13}NO_3Na$: 230.0793, found, 230.0800.

Ethyl 2-(hydroxyimino)-3-(o-tolyl)propanoate (2b): Obtained as a white solid (3.38 g, 61% from 3 g of 2-methylbenzaldehyde) after a chromatography over silica gel (cyclohexane – ethyl acetate 4/1). ^{1}H NMR ($CDCl_3$): 10.09 (s, 1H), 7.16 (m, 4H), 4.28 (q, 2H, *J* = 7.1 Hz), 4.00 (s, 2H), 2.41 (s, 3H), 1.31 (t, 3H, *J* = 7.1 Hz). ^{13}C NMR ($CDCl_3$): 163.4, 151.2, 136.5, 133.9, 130.3, 128.6, 126.7, 126.0, 61.9, 27.9, 19.7, 14.0. HRMS (*m/z*): $[M+Na]^+$ calcd for $C_{12}H_{15}NO_3Na$: 244.0950, found, 244.0942.

Ethyl 2-(hydroxyimino)-3-(m-tolyl)propanoate (2c): Obtained as an oil (3.71 g, 63% from 3.15 g of 3-methylbenzaldehyde) after a chromatography over silica gel (cyclohexane – ethyl acetate 6/1). ^{1}H NMR ($CDCl_3$): 9.59 (s, 1H), 7.16 (m, 3H), 7.05 (m, 1H), 4.31 (q, 2H, *J* = 7.1

Hz), 3.97 (s, 2H), 2.33 (s, 3H), 1.34 (t, 3H, J = 7.1 Hz). ^{13}C NMR ($CDCl_3$): 163.2, 151.1, 138.1, 135.6, 129.9, 128.4, 127.4, 126.2, 61.9, 30.4, 21.3, 14.0. HRMS (m/z): $[M+Na]^+$ calcd for $C_{12}H_{15}NO_3Na$: 244.0950, found, 244.0954.

Ethyl 2-(hydroxyimino)-3-(p-tolyl)propanoate (2d): Obtained as a white solid (3.31 g, 60% from 3 g of 4-methylbenzaldehyde) after a chromatography over silica gel (cyclohexane – ethyl acetate 4/1). ^{1}H NMR ($CDCl_3$): 10.07 (s, 1H), 7.24 (m, 2H), 7.11 (m, 2H), 4.31 (q, 2H, J = 7.1 Hz), 3.97 (s, 2H), 2.35 (s, 3H), 1.35 (t, 3H, J = 7.1 Hz). ^{13}C NMR ($CDCl_3$): 163.3, 151.1, 136.2, 132.7, 129.2, 129.1, 61.9, 30.1, 21.0, 14.0. HRMS (m/z): $[M+Na]^+$ calcd for $C_{12}H_{15}NO_3Na$: 244.0950, found, 244.0944.

Ethyl 2-(hydroxyimino)-3-(4-isopropylphenyl)propanoate (2e): Obtained as a solid (2.04 g, 48% from 2.5 g of 4-isopropylbenzaldehyde) after a chromatography over silica gel (cyclohexane – ethyl acetate 5/1). ^{1}H NMR ($CDCl_3$): 9.74 (s, 1H), 7.26 – 7.22 (m, 2H), 7.16 – 7.11 (m, 2H), 4.29 (q, 2H, J = 7.1 Hz), 3.95 (s, 2H), 2.87 (hept, 1H, J = 6.9 Hz), 1.32 (t, 3H, J = 7.1 Hz), 1.23 (d, 6H, J = 6.9 Hz). ^{13}C NMR ($CDCl_3$): 163.3, 151.2, 147.2, 133.0, 129.1, 126.6, 61.8, 33.7, 30.1, 24.0, 14.0. HRMS (m/z): $[M+Na]^+$ calcd for $C_{14}H_{19}NO_3Na$, 272.1263, found, 272.1258.

Ethyl 3-cyclopentyl-2-(hydroxyimino)propanoate (2f): Obtained as a white solid (2.50 g, 35% from 3.49 g of cyclopentanecarbaldehyde) after a chromatography over silica gel (cyclohexane – ethyl acetate 85/15). ^{1}H NMR ($CDCl_3$): 9.51 (s, 1H), 4.32 (q, 2H, J = 7.0 Hz), 2.67 (d, 2H, J = 7.6 Hz), 2.20 (m, 1H), 1.70 (m, 4H), 1.53 (m, 2H), 1.36 (t, 3H, J = 7.0 Hz), 1.25 (m, 2H). ^{13}C NMR ($CDCl_3$): 163.8, 152.9, 61.6, 37.4, 32.5, 29.9, 24.7, 14.0. HRMS (m/z): $[M+Na]^+$ calcd for $C_{10}H_{17}NO_3Na$: 222.1106, found, 222.1164.

Ethyl 3-cyclohexyl-2-(hydroxyimino)propanoate (2g): Obtained as a white solid (4.34 g, 50% from 4.50 g of cyclohexanecarbaldehyde) after a chromatography over silica gel (cyclohexane – ethyl acetate 9/1). ^{1}H NMR ($CDCl_3$): 9.85 (s, 1H), 4.31 (q, 2H, J = 7.2 Hz), 2.55 (d, 2H, J = 7.1 Hz), 1.70 (m, 6H), 1.36 (t, 3H, J = 7.0 Hz), 1.20 (m, 3H), 1.04 (m, 2H).

^{13}C NMR ($CDCl_3$): 163.8, 152.3, 61.6, 35.8, 33.3, 31.9, 26.2, 26.1, 14.0. HRMS (*m/z*): $[M+Na]^+$ calcd for $C_{11}H_{19}NO_3Na$: 236.1263, found, 236.1291.

Ethyl 2-(hydroxyimino)-3-(2-(trifluoromethyl)phenyl)propanoate (2h): Obtained as a white solid (4.65 g, 39% from 7.5 g of 2-(trifluoromethyl)benzaldehyde) after a recrystallization in n-heptane (plus charcoal decolourisation). ^1H NMR ($CDCl_3$): 9.66 (s, 1H), 7.68 (d(br), 1H, *J* = 7.5 Hz), 7.45 (m, 1H), 7.34 (m, 1H), 7.6 (d(br), 1H, *J* = 7.7 Hz), 4.29 (q, 2H, *J* = 7.2 Hz), 4.23 (s, 2H), 1.27 (t, 3H, *J* = 7.2 Hz). ^{13}C NMR ($CDCl_3$): 161.0, 150.5, 134.1, 131.9, 129.0, 128.5 (29 Hz), 126.6, 126.2 (6 Hz), 124.4 (273 Hz), 62.1, 27.3, 13.9. HRMS (*m/z*): $[M+Na]^+$ calcd for $C_{12}H_{12}F_3NO_3Na$: 298.0667, found, 298.0660.

Ethyl 2-(hydroxyimino)-3-(3-(trifluoromethyl)phenyl)propanoate (2i): Obtained as a white solid (1.3 g, 41% from 2.01 g of 3-(trifluoromethyl)benzaldehyde)) after a chromatography over silica gel (cyclohexane – ethyl acetate 4/1 to 3/1). ^1H NMR ($CDCl_3$): 9.87 (s, 1H), 7.62 (s(br), 1H), 7.50 (m, 2H), 7.42 (m, 1H), 4.32 (q, 2H, *J* = 7.0 Hz), 4.05 (s, 2H), 1.35 (t, 3H, *J* = 7.0 Hz). ^{13}C NMR ($CDCl_3$): 162.9, 150.2, 136.6, 132.6, 130.9 (32 Hz), 128.9, 126.1 (4 Hz), 124.1 (273 Hz), 123.6 (4 Hz), 62.1, 30.3, 13.9. HRMS (*m/z*): $[M+Na]^+$ calcd for $C_{12}H_{12}F_3NO_3Na$: 298.0667, found, 298.0643.

Ethyl 2-(hydroxyimino)-3-(4-(trifluoromethyl)phenyl)propanoate (2j): Obtained as a white solid (4.61 g, 62% from 4.71 g of 4-(trifluoromethyl)benzaldehyde)) after a chromatography over silica gel (dichloromethane – ethanol 99/1). ^1H NMR ($CDCl_3$): 9.76 (s, 1H), 7.55 (m, 2H), 7.45 (m, 2H), 4.32 (q, 2H, *J* = 7.1 Hz), 4.05 (s, 2H), 1.35 (t, 3H, *J* = 7.1 Hz). ^{13}C NMR ($CDCl_3$): 163.0, 150.2, 139.7, 129.5, 129.1 (32 Hz), 125.4 (4 Hz), 124.3 (272 Hz), 62.1, 30.4, 14.0. HRMS (*m/z*): $[M+H]^+$ calcd for $C_{12}H_{13}F_3NO_3$: 276.0847, found, 276.0834.

Ethyl 3-(2-fluorophenyl)-2-(hydroxyimino)propanoate (2k): Obtained as a white solid (3.11 g, 57% from 3 g of 2-fluorobenzaldehyde), after a chromatography over silica gel (cyclohexane – ethyl acetate 4/1). ^1H NMR ($CDCl_3$): 10.20 (s, 1H), 7.22 (m, 2H), 7.05 (m, 2H), 4.30 (q, 2H, *J* = 7.1 Hz), 4.04 (s, 2H), 1.31 (t, 3H, *J* = 7.1 Hz). ^{13}C NMR ($CDCl_3$): 163.1,

160.9 (247 Hz), 150.1, 130.6 (4 Hz), 128.3 (8 Hz), 124.0 (4 Hz), 122.8 (16 Hz), 115.3 (23 Hz), 62.0, 23.7 (4 Hz), 13.9. HRMS (*m/z*): $[M+Na]^+$ calcd for $C_{11}H_{12}FNO_3Na$: 248.0699, found, 248.0704.

Ethyl 3-(3-fluorophenyl)-2-(hydroxyimino)propanoate (2l): Obtained as a white solid (9.46 g, 65% from 7.95 g of 3-fluorobenzaldehyde) after a chromatography over silica gel (cyclohexane – ethyl acetate 5/1). ^{1}H NMR ($CDCl_3$): 10.02 (s, 1H), 7.25 (m, 1H), 7.10 (m, 1H), 7.06 (m, 1H), 6.92 (m, 1H), 4.32 (q, 2H, *J* = 7.2 Hz), 3.99 (s, 2H), 3.74 (m, 1H), 1.35 (t, 3H, *J* = 7.2 Hz). ^{13}C NMR ($CDCl_3$): 163.0, 162.9 (245 Hz), 150.3, 138.0 (6 Hz), 129.8 (8 Hz), 124.8 (3 Hz), 116.2 (22 Hz), 113.6 (21 Hz), 62.0, 30.2, 14.0. HRMS (*m/z*): $[M+Na]^+$ calcd for $C_{11}H_{12}FNO_3Na$: 248.0699, found, 248.0699.

Ethyl 3-(4-fluorophenyl)-2-(hydroxyimino)propanoate (2m): Out of a 14.80 g crude batch, only a sample (0.51 g) was purified by a chromatography over silica gel (dichloromethane – ethanol 99/1 to 98/2) to give a white solid (0.41 g). ^{1}H NMR ($CDCl_3$): 9.88 (s, 1H), 7.30 (m, 2H), 6.97 (m, 2H), 4.31 (q, 2H, *J* = 7.2 Hz), 3.91 (s, 2H), 3.74 (m, 1H), 1.34 (t, 3H, *J* = 7.2 Hz). ^{13}C NMR ($CDCl_3$): 163.1, 161.7 (245 Hz), 150.8, 131.3 (3 Hz), 130.7 (8 Hz), 115.3 (21 Hz), 62.0, 29.7, 14.0. HRMS (*m/z*): $[M+H]^+$ calcd for $C_{11}H_{13}FNO_3$: 226.0879, found, 226.0878.

Ethyl 3-(2,4-difluorophenyl)-2-(hydroxyimino)propanoate (2n): Obtained as a white solid (2.21 g, 50% from 2.57 g of 2,4-difluorobenzaldehyde) after a chromatography over silica gel (cyclohexane – ethyl acetate 6/1). ^{1}H NMR ($CDCl_3$): ^{1}H NMR ($CDCl_3$): 9.72 (s, 1H), 7.22 (m, 1H), 6.80 (m, 2H), 4.30 (q, 2H, *J* = 7.1 Hz), 3.98 (s, 2H), 1.33 (t, 3H, *J* = 7.1 Hz). ^{13}C NMR ($CDCl_3$): 163.1, 161.8 (12 and 248 Hz), 160.8 (12 and 249 Hz), 149.9, 131.2 (5 and 9 Hz), 118.5 (4 and 16 Hz), 111.1 (4 and 21 Hz), 103.7 (25 Hz), 62.1, 23.1 (4 Hz), 13.9. HRMS (*m/z*): $[M+Na]^+$ calcd for $C_{11}H_{11}F_2NO_3Na$: 266.0605, found, 266.0601.

Ethyl 3-(2,6-difluorophenyl)-2-(hydroxyimino)propanoate (2o): Obtained as a white solid (4.66 g, 54% from 5.03 g of 2,6-difluorobenzaldehyde) after a chromatography over silica gel (cyclohexane – ethyl acetate 4/1). ^{1}H NMR ($CDCl_3$): 9.81 (s, 1H), 7.18 (m, 1H), 6.85 (m, 2H),

4.29 (q, 2H, J = 7.1 Hz), 4.01 (s, 2H), 1.30 (t, 3H, J = 7.1 Hz). ^{13}C NMR ($CDCl_3$): 162.7, 161.4 (249 and 8 Hz), 148.8, 128.1 (10 Hz), 112.2 (19 Hz), 111.0 (two signals), 61.9, 18.5 (4 Hz), 13.8. HRMS (m/z): $[M+Na]^+$ calcd for $C_{11}H_{11}F_2NO_3Na$: 266.0605, found, 266.0613.

Ethyl 3-(2,3-difluorophenyl)-2-(hydroxyimino)propanoate (2p): Obtained as a white solid (4.36 g, 51% from 5 g of 2,3-difluorobenzaldehyde) after a chromatography over silica gel (cyclohexane – ethyl acetate 3.75/1.25). ^{1}H NMR ($CDCl_3$): 9.83 (s, 1H), 7.01 (m, 3H), 4.32 (q, 2H, J = 7.1 Hz), 4.06 (s, 2H), 1.33 (t, 3H, J = 7.1 Hz). ^{13}C NMR ($CDCl_3$): 162.8, 150.7 (248 and 13 Hz), 149.5, 149.0 (248 and 13 Hz), 125.2 (7 Hz), 125.1 (2 Hz), 123.8 (7 and 5 Hz), 115.6 (17 Hz), 62.1, 23.4 (4 and 3 Hz), 13.9. HRMS (m/z): $[M+Na]^+$ calcd for $C_{11}H_{11}F_2NO_3Na$: 266.0604, found, 266.0603.

Ethyl 3-(2,5-difluorophenyl)-2-(hydroxyimino)propanoate (2q): Obtained as a white solid (4.83 g, 50% from 5.17 g of 2,5-difluorobenzaldehyde, 96% pure) after a chromatography over silica gel (cyclohexane – ethyl acetate 4/1). ^{1}H NMR ($CDCl_3$): 9.66 (s, 1H), 6.95 (m, 3H), 4.32 (q, 2H, J = 7.1 Hz), 4.01 (s, 2H), 1.34 (t, 3H, J = 7.1 Hz). ^{13}C NMR ($CDCl_3$): 162.8, 158.6 (242 and 2 Hz), 156.8 (242 and 3 Hz), 149.6, 124.3 (19 and 8 Hz), 117.0 (25 and 4 Hz), 116.2 (25 and 9 Hz), 114.7 (24 and 8 Hz), 62.1, 23.7 (3 and 1 Hz), 13.9. HRMS (m/z): $[M+Na]^+$ calcd for $C_{11}H_{11}F_2NO_3Na$: 266.0605, found, 266.0615.

Ethyl 3-(3,5-difluorophenyl)-2-(hydroxyimino)propanoate (2r): Obtained as a white solid (4.18 g, 46% from 5.35 g of 3,5-difluorobenzaldehyde) after a chromatography over silica gel (cyclohexane – ethyl acetate 4/1). ^{1}H NMR ($CDCl_3$): 9.91 (s, 1H), 6.88 (m, 2H), 6.68 (m, 1H), 4.34 (q, 2H, J = 7.1 Hz), 3.97 (s, 2H), 1.36 (t, 3H, J = 7.1 Hz). ^{13}C NMR ($CDCl_3$): 163.0 (248 and 13 Hz), 162.9, 149.7, 139.2 (10 Hz), 112.2 (two signals), 102.3 (25 Hz), 62.2, 30.2, 14.0. HRMS (m/z): $[M+H]^+$ calcd for $C_{11}H_{12}F_2NO_3$: 244.0785, found, 244.0788.

Ethyl 2-(hydroxyimino)-3-(2,3,5-trifluorophenyl)propanoate (2s): Obtained as a white solid (3.02 g, 35% from 5.12 g of 2,3,5-trifluorobenzaldehyde) after a chromatography over silica gel (cyclohexane – ethyl acetate 4/1) and a recrystallization in cyclohexane. ^{1}H NMR ($CDCl_3$): 10.02 (s(br), 1H), 6.78 (m, 2H), 4.33 (q, 2H, J = 7.2 Hz), 4.03 (s, 2H), 1.34 (t, 3H, J

= 7.2 Hz). ^{13}C NMR ($CDCl_3$): 162.7, 157.4 (244, 10 and 3 Hz), 150.3 (250, 28 and 13 Hz), 148.9, 145.6 (244, 12 and 3 Hz), 126.0 (14 and 9 Hz), 103.9 (27 and 21 Hz), 62.3, 13.9.

Ethyl 3-(2-chlorophenyl)-2-(hydroxyimino)propanoate (2t): As described in the text, the preparation of oxime **2t** had to be optimized. The condensation of diethylmalonate was run as described above but in dry isopropanol, the reduction was undertaken as described above but with tetramethyl ammonium borohydride instead of sodium boron hydride. Pure oxime **2t** was obtained as a white solid (5.98 g, 49% from 7.01 g of 2-chlorobenzaldehyde) after a chromatography over silica gel (cyclohexane – ethyl acetate 4/1). ^{1}H NMR ($CDCl_3$): 9.47 (s, 1H), 7.38 (m, 1H), 7.18 (m, 3H), 4.30 (q, 2H, J = 7.1 Hz), 4.13 (s, 2H), 1.31 (t, 3H, J = 7.1 Hz). ^{13}C NMR ($CDCl_3$): 163.1, 150.5, 134.0, 133.5, 129.7, 129.5, 128.0, 126.8, 62.0, 28.5, 14.0. HRMS (*m/z*): $[M+Na]^+$ calcd for $C_{11}H_{12}ClNO_3Na$: 264.0403, found, 264.0401.

Ethyl-3-(3-chlorophenyl)-2-(hydroxyimino)propanoate (2u): The protocol described for the preparation of oxime **2t** was used to prepare compound **2u**, which was obtained as a light yellow solid (7.09 g, 59% from 7.01 g of 3-chlorobenzaldehyde) after a chromatography over silica gel (cyclohexane – ethyl acetate 4/1). ^{1}H NMR ($CDCl_3$): ^{1}H NMR ($CDCl_3$): 9.60 (s, 1H), 7.34 (m, 1H), 7.22 (m, 3H), 4.33 (q, 2H, J = 7.1 Hz), 3.97 (s, 2H), 1.36 (t, 3H, J = 7.1 Hz). ^{13}C NMR ($CDCl_3$): 163.0, 150.4, 137.6, 134.3, 129.7, 129.4, 127.4, 126.9, 62.1, 30.1, 14.0. HRMS (*m/z*): $[M+Na]^+$ calcd for $C_{11}H_{12}ClNO_3Na$: 264.0403, found, 264.0406. Note: ^{1}H NMR data for diethyl 2-((3-chlorophenyl)(ethoxy)methyl)malonate (**7**) which were extracted from the ^{1}H NMR analysis of a (concentrated to dryness) 3 weeks-old ethanol solution of compound **6u**: ^{1}H NMR ($CDCl_3$): 7.40 (m, 1H), 7.34 (m, 1H), 7.29 (m, 2H), 4.88 (d, 1H, J = 10.2 Hz), 4.29 (m, 2H), 3.98 (m, 2H), 3.70 (d, 1H, J = 10.2 Hz), 3.37 (m, 2H), 1.32 (t, 3H, J = 7.3 Hz), 1.13 (t, 3H, J = 7.1 Hz), 1.05 (t, 3H, J = 6.9 Hz).

Ethyl-3-(4-chlorophenyl)-2-(hydroxyimino)propanoate (2v): The protocol described for the preparation of oxime **2t** was used to prepare compound **2v**, which was obtained as a light yellow solid (8.4 g, 62% from 7.83 g of 4-chlorobenzaldehyde) after a chromatography over silica gel (cyclohexane – ethyl acetate 4/1). ^{1}H NMR ($CDCl_3$): 9.70 (s, 1H), 7.27 (m, 4H),

4.31 (q, 2H, J = 7.1 Hz), 3.96 (s, 2H), 1.35 (t, 3H, J = 7.1 Hz). ^{13}C NMR ($CDCl_3$): 163.0, 150.6, 134.1, 132.6, 130.6, 128.6, 62.0, 29.9, 14.0. HRMS (*m/z*): $[M+Na]^+$ calcd for $C_{11}H_{14}ClNO_2Na$, 264.0403, found, 264.0437. Spectroscopic data similar to the reported one [1].

Ethyl-3-(4-bromophenyl)-2-(hydroxyimino)propanoate (2w): Obtained as a white solid (1.43 g, 42% from 5.0 g of 4-bromobenzaldehyde), using sodium boron hydride at 4 °C overnight, after a chromatography over silica gel (cyclohexane – ethyl acetate 4/1). ^{1}H NMR ($CDCl_3$): 9.70 (s, 1H), 7.42 (m, 2H), 7.22 (m, 2H), 4.31 (q, 2H, J = 7.1 Hz), 3.96 (s, 2H), 1.32 (t, 3H, J = 7.1 Hz). ^{13}C NMR ($CDCl_3$): 163.0, 150.5, 134.7, 131.6, 130.9, 120.6, 62.0, 29.9, 14.0. HRMS (*m/z*): $[M+H]^+$ calcd for $C_{11}H_{13}BrNO_3$, 286.0079, found, 286.0056.

Ethyl 2-(hydroxyimino)-3-(2-methoxyphenyl)propanoate (2x): Obtained as a white solid (3.15 g, 60% from 3.0 g of 2-methoxybenzaldehyde) after a chromatography over silica gel (cyclohexane – ethyl acetate 4/1). ^{1}H NMR ($CDCl_3$): 9.65 (s, 1H), 7.22 (m, 1H), 7.14 (m, 1H), 6.88 (m, 2H), 4.29 (q, 2H, J = 7.1 Hz), 3.99 (s, 2H), 3.83 (s, 3H), 1.31 (t, 3H, J = 7.1 Hz). ^{13}C NMR ($CDCl_3$): 163.5, 157.2, 151.6, 129.7, 127.8, 124.3, 120.5, 110.4, 61.7, 55.4, 25.7, 14.0. HRMS (*m/z*): $[M+Na]^+$ calcd for $C_{12}H_{15}NO_4Na$: 260.0899, found, 260.0904.

Ethyl 2-(hydroxyimino)-3-(3-methoxyphenyl)propanoate (2y): Out of a 10.66 g batch, only a sample (0.43 g) was purified by a chromatography (dichloromethane – ethanol 98/2) to give a white solid (0.33 g). ^{1}H NMR ($CDCl_3$): 9.79 (s, 1H), 7.21 (t, 1H, J = 7.9 Hz), 6.93 (m, 1H), 6.91 (m, 1H), 6.77 (m, 1H), 4.30 (q, 2H, J = 7.1 Hz), 3.98 (s, 2H), 3.80 (s, 3H), 1.34 (t, 3H, J = 7.1 Hz). ^{13}C NMR ($CDCl_3$): 163.2, 159.7, 150.9, 137.2, 150.9, 137.2, 129.4, 121.5, 115.0, 112.1, 61.9, 55.1, 30.5, 14.0. HRMS (*m/z*): $[M+Na]^+$ calcd for $C_{12}H_{15}NO_4Na$: 260.0849, found, 260.0898.

Ethyl 3-(2-(benzyloxy)phenyl)-2-(hydroxyimino)propanoate (2z): Obtained as a white solid (10.78 g, 48% from 15.0 g of 2-(benzyloxy)benzaldehyde) after an initial recrystallization which gave 3.34 g of compound **2z** and a chromatography over silica gel of the filtrate (cyclohexane – ethyl acetate 4/1 to 7/3) followed by a recrystallization in

cyclohexane which gave an additional 7.44 g. Note: instead of 10 hours at 60 °C, for the condensation step, heating at 75 °C for 24 hours led to an almost complete disappearance of the starting aldehyde signal. ^{1}H NMR ($CDCl_3$) 9.14 (s(br), 1H), 7.46 (m, 2H), 7.39 (m, 2H), 7.32 (m, 1H) 6.90 (m, 2H), 5.11 (s, 2H), 4.15 (q, 2H, *J* = 7.2 Hz), 4.07 (s, 2H), 1.24 (t, 3H, *J* = 7.2 Hz). ^{13}C NMR ($CDCl_3$): 163.4, 156.4, 151.5, 137.1, 129.9, 128.5, 127.8, 127.7, 127.4, 124.7, 120.8, 111.8, 70.1, 61.6, 25.8, 13.9.

Ethyl 3-(3-(benzyloxy)phenyl)-2-(hydroxyimino)propanoate (2aa): Obtained as a white solid (8.09 g, 37% from 15.0 g of 3-(benzyloxy)benzaldehyde) after a chromatography over silica gel (cyclohexane – ethyl acetate 4/1 to 7/3) and a recrystallization in cyclohexane. Note: instead of 10 hours at 60 °C, for the condensation step, heating at 75 °C for 24 hours led to an almost complete disappearance of the starting aldehyde signal. ^{1}H NMR ($CDCl_3$) 9.41 (s(br), 1H), 7.46 – 7.31 (m, 5H), 7.27 (m, 1H), 6.99 (m, 1H), 6.94 (m, 1H), 6.85 (m, 1H), 5.06 (s, 2H), 4.31 (q, 2H, *J* = 7.2 Hz), 3.99 (s, 2H), 1.35 (t, 3H, *J* = 7.2 Hz). ^{13}C NMR ($CDCl_3$): 163.2, 159.0, 150.9, 137.2, 137.1, 129.5, 128.5, 127.9, 127.5, 121.9, 115.9, 113.0, 70.0, 61.9, 30.5, 14.0.

Ethyl 3-(4-(benzyloxy)phenyl)-2-(hydroxyimino)propanoate (2ab): Obtained as a white solid (5.99 g, 40% from 10.03 g of 4-(benzyloxy)benzaldehyde) after a chromatography over silica gel (cyclohexane – ethyl acetate 4/1 to 1/2). Note: instead of 10 hours at 60 °C, for the condensation step, heating at 75 °C for 12 hours led to an almost complete disappearance of the starting aldehyde signal. ^{1}H NMR ($CDCl_3$) 8.63 (s(br), 1H), 7.46 – 7.32 (m, 5H), 7.27 (m, 2H), 6.92 (m, 2H), 5.07 (s, 2H), 4.31 (q, 2H, *J* = 7.2 Hz), 3.94 (s, 2H), 1.34 (t, 3H, *J* = 7.2 Hz). ^{13}C NMR ($CDCl_3$): 163.3, 157.6, 151.3, 137.3, 128.7, 128.5, 128.0, 127.9, 127.4, 114.9, 70.0, 61.9, 29.6, 14.0.

Ethyl 2-(hydroxyimino)-3-(pyridin-2-yl)propanoate (2ac): Obtained as a solid (4.94 g, 49% from 7.10 g of picolinaldehyde) after a chromatography over silica gel (dichloromethane – ethanol 97/3). 10.53 (s, 1H), 8.53 (m, 1H), 7.61 (dt, 1H, *J* = 1.8, 7.5 Hz), 7.29 (m, 1H), 7.17 (m, 1H), 4.29 (q, 2H, *J* = 7.1 Hz), 4.23 (s, 2H), 1.30 (t, 3H, *J* = 7.1 Hz). ^{13}C NMR ($CDCl_3$):

163.6, 156.6, 150.0, 149.0, 137.0, 123.5, 121.7, 61.8, 33.2, 14.0. HRMS (*m/z*): $[M+H]^+$ calcd for $C_{10}H_{13}N_2O_3$: 209.0926, found, 209.0857.

Ethyl 2-(hydroxyimino)-3-(pyridin-3-yl)propanoate (2ad): Obtained as a tan solid (7.19 g, 43% from 8.47 g of nicotinaldehyde) after a chromatography over silica gel (dichloromethane – ethanol 97/3 to 96/4). ^{1}H NMR ($CDCl_3$): 12.79 (s, 1H), 8.65 (m, 1H), 8.46 (dd, 1H, J = 1.6, 4.9 Hz), 7.74 (m, 1H), 7.22 (m, 1H), 4.30 (q, 2H, J = 7.1 Hz), 4.01 (s, 2H), 1.32 (t, 3H, J = 7.1 Hz). ^{13}C NMR ($CDCl_3$): 163.7, 149.9, 149.6, 147.0, 137.6, 132.9, 123.7, 61.8, 28.1, 14.1. HRMS (*m/z*): $[M+H]^+$ calcd for $C_{10}H_{13}N_2O_3$: 209.0926, found, 209.0921.

Ethyl 3-(furan-2-yl)-2-(hydroxyimino)propanoate (2ae): As mentioned in the text, in this specific case the reduction step with sodium borohydride was run in ethanol at -10 °C for 1h30 and compound **2ae** was obtained as a white solid (7.90 g, 47% from 8.07 g of furfural) after a chromatography over silica gel (cyclohexane – ethyl acetate 4/1). ^{1}H NMR ($CDCl_3$): 9.72 (s, 1H), 7.31 (m, 1H), 6.30 (m, 1H), 6.13 (m, 1H), 4.33 (q, 2H, J = 7.1 Hz), 4.04 (s, 2H), 1.35 (t, 3H, J = 7.1 Hz), 1.25 (m, 2H). ^{13}C NMR ($CDCl_3$): 162.8, 148.7, 148.4, 141.5, 110.4, 107.0, 62.0, 23.5, 14.0. HRMS (*m/z*): $[M+H]^+$ calcd for $C_9H_{12}NO_4$: 198.0766, found, 198.0775. ^{1}H NMR similar to the reported one [2].

Ethyl 2-(hydroxyimino)-3-(5-methylfuran-2-yl)propanoate (2af): Obtained as an off white solid (5.99 g, 33% from 10.11 g of 5-methylfuran-2-carbaldehyde) after a chromatography over silica gel (cyclohexane – ethyl acetate 4/1). ^{1}H NMR ($CDCl_3$): 9.41 (s, 1H), 5.99 (m, 1H), 5.86 (m, 1H), 4.33 (q, 2H, J = 7.0 Hz), 3.98 (s, 2H), 2.25 (s, 3H), 1.35 (t, 3H, J = 7.0 Hz). ^{13}C NMR ($CDCl_3$): 162.9, 151.1, 148.8, 146.7, 107.6, 106.3, 61.9, 26.9, 14.0, 13.5. HRMS (*m/z*): $[M+Na]^+$ calcd for $C_{10}H_{13}NO_4Na$: 234.0742, found, 234.0744. ^{1}H NMR similar to the reported one [3].

Ethyl 3-(4,5-dimethylfuran-2-yl)-2-(hydroxyimino)propanoate (2ag): As mentioned in the text, in this specific case the reduction step with sodium borohydride was run in THF (20 mL) and ethanol (20 mL) at -10 °C for 1h30 and compound **2ag** was obtained as a solid (0.84 g, 23% from 2.03 g of aldehyde **5ag**), after a chromatography over silica gel (cyclohexane –

ethyl acetate 5/1). ^{1}H NMR ($CDCl_3$): 9.89 (s, 1H), 5.87 (s, 1H), 4.32 (q, 2H, *J* = 7.2 Hz), 3.94 (s, 2H), 2.15 (s, 3H), 1.88 (s, 3H), 1.35 (t, 3H, *J* = 7.2 Hz). ^{13}C NMR ($CDCl_3$): 163.0, 148.7, 146.3, 145.4, 114.6, 110.1, 61.9, 23.6, 14.0, 11.2, 9.8. HRMS (*m/z*): $[M+Na]^+$ calcd for $C_{11}H_{15}NO_4Na$: 248.0899, found, 248.0896.

Ethyl 3-(5-ethylfuran-2-yl)-2-(hydroxyimino)propanoate (2ah): Obtained as a white solid (3.17 g, 34% from 5.14 g of 5-ethylfuran-2-carbaldehyde) after a chromatography over silica gel (cyclohexane – ethyl acetate 5/1). ^{1}H NMR ($CDCl_3$): 9.79 (s, 1H), 5.98 (m, 1H), 5.86 (m, 1H), 4.33 (q, 2H, *J* = 7.1 Hz), 3.99 (s, 2H), 2.60 (q, 2H, *J* = 7.4 Hz), 1.35 (t, 3H, *J* = 7.1 Hz), 1.20 (t, 3H, *J* = 7.4 Hz). ^{13}C NMR ($CDCl_3$): 163.0, 156.8, 148.7, 146.6, 107.3, 104.6, 61.9, 23.7, 21.3, 14.0, 12.0. HRMS (*m/z*): $[M+Na]^+$ calcd for $C_{11}H_{15}NO_4Na$: 248.0899, found, 248.0868.

Ethyl 2-(hydroxyimino)-3-(5-(trifluoromethyl)furan-2-yl)propanoate (2ai): Obtained as a white solid (0.62 g, 36% from 1.06 g of 5-trifluoromethylfuran-2-carbaldehyde), using sodium boron hydride at 0 °C, after two chromatography over silica gel (dichloromethane – ethanol 99/1) and (cyclohexane – ethyl acetate 5/1). ^{1}H NMR ($CDCl_3$): 9.70 (s, 1H), 6.72 – 6.64 (m, 1H), 6.21 – 6.15 (m, 1H), 4.33 (q, 2H, *J* = 7.1 Hz), 4.05 (s, 2H), 1.34 (t, 3H, *J* = 7.1 Hz). ^{13}C NMR ($CDCl_3$): 162.7, 152.0, 147.6, 141.0 (q, *J* = 42.7 Hz), 119.2 (q, *J* = 266.7 Hz), 112.7 (q, *J* = 2.8 Hz), 108.18 (s), 62.4, 23.7, 14.1. HRMS (*m/z*): $[M+H]^+$ calcd for $C_{10}H_{11}F_3NO_4$: 266.0640, found, 266.0584.

Ethyl 3-(5-ethylthiophen-2-yl)-2-(hydroxyimino)propanoate (2aj): Obtained as an oil (1.36 g, 29% from 2.69 g of 5-ethylthiophene-2-carbaldehyde), using sodium boron hydride at 0 °C, after a chromatography over silica gel (cyclohexane – ethyl acetate 5/1). ^{1}H NMR ($CDCl_3$): 10.06 (s, 1H), 6.75 (m, 1H), 6.59 (m, 1H), 4.34 (q, 2H, *J* = 7.2 Hz), 4.09 (s, 2H), 2.78 (m, 2H), 1.38 (t, 3H, *J* = 7.2 Hz), 1.28 (t, 3H, *J* = 7.5 Hz). ^{13}C NMR ($CDCl_3$): 163.0, 150.1, 146.6, 133.9, 126.3, 122.8, 62.0, 24.8, 23.4, 15.8, 14.0. HRMS (*m/z*): $[M+Na]^+$ calcd for $C_{11}H_{15}NO_3SNa$: 264.0670, found, 264.0676.

Ethyl 2-(hydroxyimino)-3-(3-methylthiophen-2-yl)propanoate (2ak): Obtained as a yellow solid (1.38 g, 32% from 2.39 g of 3-methylthiophene-2-carbaldehyde), using sodium boron hydride at 0 °C, after a chromatography over silica gel (cyclohexane – ethyl acetate 5/1). ^{1}H NMR ($CDCl_3$): 9.88 (s, 1H), 7.06 (d, 1H, *J* = 5.1 Hz), 6.77 (d, 1H, *J* = 5.1 Hz), 4.33 (q, 2H, *J* = 7.2 Hz), 4.09 (s, 2H), 2.28 (s, 3H), 1.36 (t, 3H, *J* = 7.2 Hz). ^{13}C NMR ($CDCl_3$): 163.0, 150.1, 135.0, 130.3, 129.7, 122.8, 62.0, 22.9, 14.0, 13.7. HRMS (*m/z*): $[M+Na]^+$ calcd for $C_{10}H_{13}NO_3SNa$: 250.0514, found, 250.0513.

Ethyl 3-(4,5-dimethylthiophen-2-yl)-2-(hydroxyimino)propanoate (2al): Obtained as an oil (0.69 g, 31% from 1.29 g of 4,5-dimethylthiophene-2-carbaldehyde; 90 % pure), using sodium boron hydride at 0 °C, after a chromatography over silica gel (cyclohexane – ethyl acetate 5/1). ^{1}H NMR ($CDCl_3$): 9.77 (s, 1H), 6.62 (s, 1H), 4.34 (q, 2H, *J* = 7.2 Hz), 4.03 (s, 2H), 2.28 (s, 3H), 2.06 (s, 3H), 1.37 (t, 3H, *J* = 7.2 Hz). ^{13}C NMR ($CDCl_3$): 163.0, 150.1, 132.6, 131.7, 131.6, 129.6, 62.0, 24.6, 14.0, 13.4, 12.8. HRMS (*m/z*): $[M+Na]^+$ calcd for $C_{11}H_{15}NSO_3Na$: 264.0670, found, 264.0656.

General method for the preparation of the α-amino esters by zinc-based reduction of the α-hydroxyimino esters: The considered substituted α-hydroxyimino ester (0.065 mol) was dissolved in ethanol (70 mL) and 37% hydrochloric acid (49 mL, 0.58 mol) and the solution was cooled to 0 °C. Powdered zinc (12.9 g, 0.19 mol, less than 10 μm size) was added in one portion. The mixture was stirred for 3 hours, diluted with water, made basic with 22% ammonia and extracted with ethyl acetate. The organic layer was washed with water, brine, dried over sodium carbonate and concentrated to dryness to yield the α-amino esters **1** which were in some cases further purified as described below. Note: these oily α-amino esters are not very stable, and unless the corresponding hydrochloric salt is made (by adding 4 N HCl in dioxane and concentration to dryness), many of them will slowly dimerize into the corresponding solid piperazine-2,5-diones even when stored at −20 °C.

Ethyl 2-amino-3-(*o*-tolyl)propanoate (1b): Obtained as an oil (2.69 g, 85% from 3.38 g of oxime). ^{1}H NMR ($CDCl_3$): 7.16 (m, 4H), 4.17 (q, 2H, J = 7.2 Hz), 3.71 (dd, 1H, J = 8.7, 5.6 Hz), 3.14 (dd, 1H, J = 13.8, 5.6 Hz), 2.84 (dd, 1H, J = 13.8, 8.7 Hz), 2.82 (s, 3H), 1.24 (t, 3H, J = 7.2 Hz). ^{13}C NMR ($CDCl_3$): 175.3, 136.5, 135.9, 130.5, 130.0, 126.8, 125.9, 60.9, 55.0, 38.9, 19.5, 14.1. HRMS (*m/z*): $[M+H]^+$ calcd for $C_{12}H_{18}NO_2$: 208.1338, found, 208.1332.

Ethyl 2-amino-3-(*m*-tolyl)propanoate (1c): Obtained as an oil (3.07 g, 85% from 3.68 g of oxime). Analytical data identical with the one we reported previously [4].

Ethyl 2-amino-3-(*p*-tolyl)propanoate (1d): Obtained as an oil (2.70 g, 87% from 3.31 g of oxime). ^{1}H NMR ($CDCl_3$): 7.11 (m, 4H), 4.19 (q, 2H, J = 7.1 Hz), 3.70 (dd, 1H, J = 7.9, 5.3 Hz), 3.07 (dd, 1H, J = 13.6, 5.3 Hz), 2.84 (dd, 1H, J = 13.6, 7.9 Hz), 2.34 (s, 3H), 1.28 (t, 3H, J = 7.1 Hz). ^{13}C NMR ($CDCl_3$): 175.1, 136.3, 134.2, 129.4, 129.2 (two signals), 129.1, 60.9, 55.9, 40.7, 21.0, 14.2. HRMS (*m/z*): $[M+H]^+$ calcd for $C_{12}H_{18}NO_2$: 208.1338, found, 208.1333. Spectroscopic data similar to the reported one [5].

Ethyl 2-amino-3-(4-isopropylphenyl)propanoate hydrochloride (1e): Obtained from 2.02 g of the oxime as an oil which was immediately treated with a solution of hydrogen chloride in dioxane and concentrated to dryness to give the hydrochloride salt as a white powder (1.9 g, 91% pure). ^{1}H NMR (DMSO-d_6): 8.23 (s, 3H), 7.22 – 7.12 (m, 4H), 4.16 – 4.02 (m, 3H), 3.16 (dd, 1H, J = 13.9, 5.8 Hz), 3.01 (dd, 1H, J = 13.9, 7.8 Hz), 2.86 (hept, 1H, J = 6.9 Hz), 1.18 (d, 6H, J = 6.9 Hz), 1.06 (t, 3H, J = 7.1 Hz). ^{13}C NMR (DMSO-d_6): 169.3, 147.2, 132.2, 129.3, 126.3, 61.3, 53.4, 35.9, 33.0, 23.8, 13.7. HRMS (*m/z*): $[M+H]^+$ calcd for $C_{14}H_{22}NO_2$: 236.1651, found, 236.1651. ^{1}H NMR spectra not similar to the reported one [6].

Ethyl 2-amino-3-cyclopentylpropanoate (1f): Obtained as an oil (1.16 g, 85% from 1.46 g of oxime). ^{1}H NMR ($CDCl_3$): 4.17 (q, 2H, J = 7.2 Hz), 3.42 (dd, 1H, J = 5.8, 8.1 Hz), 1.93 (m, 1H), 1.80 (m, 2H), 1.75-1.48 (m, 8H), 1.27 (t, 3H, J = 7.2 Hz), 1.12 (m, 2H). ^{13}C NMR ($CDCl_3$): 174.5, 60.6, 54.1, 41.4, 36.7, 32.9, 32.3, 25.1, 24.9, 14.2. HRMS (*m/z*): $[M+H]^+$ calcd for $C_{10}H_{20}NO_2$: 186.1494, found, 186.1491.

Ethyl 2-amino-3-cyclohexylpropanoate (1g): Obtained as an oil (2.35 g, 92% from 2.73 g of oxime). ^{1}H NMR ($CDCl_3$): 4.17 (m, 2H), 3.48 (dd, 1H, J = 5.8, 8.7 Hz), 1.72 (m, 5H), 1.61-137 (m, 5H), 1.28 (t, 3H, J = 7.3 Hz), 1.28-1.10 (m, 3H), 0.92 (m, 2H). ^{13}C NMR ($CDCl_3$): 174.7, 60.6, 52.2, 42.7, 34.1, 33.7, 32.6, 26.5, 26.2, 26.1, 14.2. HRMS (*m/z*): $[M+H]^+$ calcd for $C_{11}H_{22}NO_2$: 200.1651, found, 200.1648. ^{1}H NMR spectra similar to the reported one [7].

Ethyl 2-amino-3-(2-(trifluoromethyl)phenyl)propanoate (1h): Obtained as an oil (7.22 g, 86% from 8.82 g of oxime). ^{1}H NMR ($CDCl_3$): 7.67 (m, 1H), 7.50 (m, 1H), 7.41 (m, 1H), 7.35 (m, 1H), 4.16 (q, 2H, J = 7.1 Hz), 3.75 (dd, 1H, J = 5.8, 8.7 Hz), 3.30 (dd, 1H, J = 5.8, 14.3 Hz), 2.97 (dd, 1H, J = 8.7, 14.3 Hz), 1.69 (s(br), 2H), 1.22 (t, 3H, J = 7.1 Hz). ^{13}C NMR ($CDCl_3$): 174.9, 136.4 (2 Hz), 131.8, 131.6, 129.1 (32 Hz), 126.8, 126.2 (5 Hz), 124.5 (274 Hz), 61.0, 55.6, 38.0, 14.0. HRMS (*m/z*): $[M+H]^+$ calcd for $C_{12}H_{15}F_3NO_2$: 262.1055, found, 262.1065.

Ethyl 2-amino-3-(3-(trifluoromethyl)phenyl)propanoate (1i): Obtained as an oil (0.75 g, 78% from 1.01 g of oxime) . ^{1}H NMR ($CDCl_3$): 7.51 (m, 1H), 7.48 (m, 1H), 7.43 (m, 2H), 4.17 (q, 2H, J = 7.3 Hz), 3.74 (dd, 1H, J = 5.5, 7.5 Hz), 3.13 (dd, 1H, J = 5.5, 13.8 Hz), 2.96 (dd, 1H, J = 7.5, 13.8 Hz), 1.51 (s, 2H), 1.24 (t, 3H, J = 7.3 Hz). ^{13}C NMR ($CDCl_3$): 174.7, 138.4, 132.8, 130.8 (32 Hz), 128.9, 126.0 (4 Hz), 124.2 (272 Hz), 123.6 (4 Hz), 61.1, 55.7, 40.9, 14.1. HRMS (*m/z*): $[M+H]^+$ calcd for $C_{12}H_{15}F_3NO_2$: 262.1055, found, 262.1054.

Ethyl 2-amino-3-(4-(trifluoromethyl)phenyl)propanoate (1j): Obtained as an oil (1.23 g, 91% from 1.41 g of oxime). ^{1}H NMR ($CDCl_3$): 7.58 (d, 2H, J = 8.3 Hz), 7.35 (d, 2H, J = 8.3 Hz), 4.18 (q, 2H, J = 7.3 Hz), 3.74 (dd, 1H, J = 5.8, 7.8 Hz), 3.14 (dd, 1H, J = 5.8, 13.4 Hz), 2.96 (dd, 1H, J = 7.8, 13.4 Hz), 1.50 (s, 2H), 1.25 (t, 3H, J = 7.3 Hz). ^{13}C NMR ($CDCl_3$): 174.7, 141.6, 129.6, 129.1 (32 Hz), 125.3 (4 Hz), 124.2 (270 Hz), 61.1, 55.6, 40.8, 14.1. HRMS (*m/z*): $[M+H]^+$ calcd for $C_{12}H_{15}F_3NO_2$: 262.1055, found, 262.1129. ^{1}H NMR spectra similar to the reported one [6].

Ethyl 2-amino-3-(2-fluorophenyl)propanoate (1k): Obtained as an oil (2.57 g, 88% from 3.11 g of oxime). Analytical data identical with the one we reported previously [4].

Ethyl 2-amino-3-(3-fluorophenyl)propanoate (1l): Obtained as an oil (4.89 g, 89% from 5.84 g of oxime). ^{1}H NMR ($CDCl_3$): 7.27 (m, 1H), 7.01-6.92 (m, 3H), 4.18 (q, 2H, *J* = 7.0 Hz), 3.72 (dd, 1H, *J* = 5.4, 7.9 Hz), 3.08 (dd, 1H, *J* = 5.4, 13.7 Hz), 2.88 (dd, 1H, *J* = 7.9, 13.7 Hz), 1.49 (s, 2H), 1.26 (t, 3H, *J* = 7.0 Hz). ^{13}C NMR ($CDCl_3$): 174.8, 162.9 (246 Hz), 139.9 (7 Hz), 129.9 (8 Hz), 125.0 (3 Hz), 116.2 (21 Hz), 113.7 (20 Hz), 61.0, 55.7, 40.8, 14.1. HRMS (*m/z*): $[M+H]^+$ calcd for $C_{11}H_{15}FNO_2$: 212.1087, found, 212.1088. ^{1}H NMR spectra similar to the reported one [5].

Ethyl 2-amino-3-(4-fluorophenyl)propanoate (1m): Obtained from 2.89 g of the crude oxime **2m** described above as an oil (2.21 g, 81% from 4-fluorobenzaldehyde) with analytical data identical with the one we reported previously [4].

Ethyl 2-amino-3-(2,4-difluorophenyl)propanoate (1n): Obtained as an oil (1.11 g, 85% from 1.38 g of oxime). ^{1}H NMR ($CDCl_3$): 7.19 (m, 1H), 6.80 (m, 2H), 4.15 (q, 2H, *J* = 7.1 Hz), 3.70 (dd, 1H, *J* = 7.9, 5.9 Hz), 3.04 (dd, 1H, *J* = 13.7, 5.9 Hz), 2.89 (dd, 1H, *J* = 13.7, 7.9 Hz), 1.52 (s, 2H), 1.23 (t, 3H, *J* = 7.1 Hz). ^{13}C NMR ($CDCl_3$): 174.8, 162.0 (11 and 247 Hz), 161.3 (12 and 249 Hz), 132.1 (6 and 9 Hz), 120.3 (4 and 16 Hz), 111.1, (3 and 21 Hz), 103.7 (26 Hz), 61.0, 54.7, 34.1, 10.1. HRMS (*m/z*): $[M+H]^+$ calcd for $C_{11}H_{14}F_2NO_2$, 230.0993, found, 230.1006.

Ethyl 2-amino-3-(2,6-difluorophenyl)propanoate (1o): Obtained as an oil (3.46 g, 94% from 3.89 g of oxime). ^{1}H NMR ($CDCl_3$): 7.21 (m, 1H), 6.89 (m, 2H), 4.18 (q, 2H, *J* = 7.1 Hz), 3.75 (dd, 1H, *J* = 8.1, 6.2 Hz), 3.14 (dd, 1H, *J* = 13.7, 6.1 Hz), 2.97 (dd, 1H, *J* = 13.7, 8.1 Hz), 1.54 (s, 2H), 1.25 (t, 3H, *J* = 7.1 Hz). ^{13}C NMR ($CDCl_3$): 174.8, 161.8 (247 and 8 Hz), 128.4 (10 Hz), 113.5 (20 Hz), 111.1, 61.1, 54.2, 28.3, 14.0. HRMS (*m/z*): $[M+H]^+$ calcd for $C_{11}H_{14}F_2NO_2$: 230.0993, found, 230.0995.

Ethyl 2-amino-3-(2,3-difluorophenyl)propanoate (1p): Obtained as an oil (3.36 g, 92% from 3.86 g of oxime). ^{1}H NMR ($CDCl_3$): 7.03 (m, 3H), 4.18 (q, 2H, *J* = 7.1 Hz), 3.76 (dd, 1H, *J* = 7.9, 5.9 Hz), 3.13 (ddd, 1H, *J* = 13.7, 5.9, 1.5 Hz), 2.97 (ddd, 1H, *J* = 13.7, 7.9, 1.3 Hz), 1.53 (s, 2H), 1.26 (t, 3H, *J* = 7.1 Hz). ^{13}C NMR ($CDCl_3$): 174.7, 150.6 (248 and 13 Hz), 149.4

(247 and 13 Hz), 127.1 (13 Hz), 126.2 (3 Hz), 123.8 (7, 5 Hz), 115.8 (17 Hz), 61.1, 54.7, 34.5, 14.1. HRMS (*m/z*): $[M+H]^+$ calcd for $C_{11}H_{14}F_2NO_2$: 230.0993, found, 230.1004.

Ethyl 2-amino-3-(2,5-difluorophenyl)propanoate (1q): Obtained as an oil (3.44 g, 95% from 3.85 g of oxime). ^{1}H NMR ($CDCl_3$): 6.96 (m, 3H), 4.19 (d, 2H, *J* = 7.1 Hz), 3.75 (dd, 1H, *J* = 7.9, 5.9 Hz), 3.08 (ddd, 1H, *J* = 13.7, 5.8, 1.2 Hz), 2.91 (ddd, 1H, *J* = 13.8, 7.9, 0.9 Hz), 1.52 (s, 2H), 1.26 (t, 3H, *J* = 7.1 Hz). ^{13}C NMR ($CDCl_3$): 163.0 (248 and 13 Hz), 162.9, 149.7, 139.2 (10 Hz), 112.2, 102.3 (25 Hz), 62.2, 30.2, 14.0. HRMS (*m/z*): $[M+H]^+$ calcd for $C_{11}H_{14}F_2NO_2$: 230.0993, found, 230.1000.

Ethyl 2-amino-3-(3,5-difluorophenyl)propanoate (1r): Obtained as an oil (3.33 g, 84% from 4.18 g of oxime). ^{1}H NMR ($CDCl_3$): 6.73 (m, 3H), 4.20 (q, 2H, *J* = 7.1 Hz), 3.71 (s, 1H), 3.06 (dd, 1H, *J* = 13.6, 5.4 Hz), 2.87 (dd, 1H, *J* = 13.6, 7.7 Hz), 1.49 (s, 2H), 1.28 (t, 3H, *J* = 7.1 Hz). ^{13}C NMR ($CDCl_3$): 174.5, 163.0 (248 and 13 Hz), 141.4 (9 Hz), 112.1, 102.3 (25 Hz), 61.1, 55.5, 40.7, 14.1. HRMS (*m/z*): $[M+H]^+$ calcd for $C_{11}H_{14}F_2NO_2$: 230.0993, found, 230.0995.

Ethyl 2-amino-3-(2,3,5-trifluorophenyl)propanoate (1s): Obtained as an oil (2.71 g, 94% from 3.04 g of oxime). ^{1}H NMR ($CDCl_3$): 6.86 – 6.75 (m, 2H), 4.18 (q, 2H, *J* = 7.1 Hz), 3.74 (dd, 1H, *J* = 5.9 and 7.8 Hz), 3.10 (ddd, 1H, *J* = 1.6, 5.9 and 13.7 Hz), 2.94 (ddd, 1H, *J* = 1.6, 7.8 and 13.7 Hz), 1.67 (s(br), 1H), 1.26 (t, 3H, *J* = 7.1 Hz). ^{13}C NMR ($CDCl_3$): 174.4, 157.4 (244, 10 and 3 Hz), 150.2 (251, 12 and 14 Hz), 145.9 (243, 13 and 4 Hz), 127.9 (9 and 15 Hz), 104.0 (21 and 27 Hz), 61.2, 54.5, 34.3, 14.0.

Ethyl 2-amino-3-(2-chlorophenyl)propanoate (1t): Obtained as an oil (4.18 g, 94% from 4.72 g of oxime). ^{1}H NMR ($CDCl_3$): 7.38 (m, 1H), 7.22 (m, 3H), 4.16 (m, 2H), 3.84 (dd, 1H, *J* = 8.5, 5.8 Hz), 3.24 (dd, 1H, *J* = 13.5, 5.8 Hz), 3.96 (dd, 1H, *J* = 13.5, 8.5 Hz), 1.23 (t, 3H, *J* = 7.2 Hz). ^{13}C NMR ($CDCl_3$): 175.0, 135.5, 134.4, 131.6, 129.6, 128.2, 126.7, 61.0, 54.4, 39.2, 14.1. HRMS (*m/z*): $[M+H]^+$ calcd for $C_{11}H_{15}ClNO_2$: 228.0791, found, 228.0791.

Ethyl 2-amino-3-(3-chlorophenyl)propanoate (1u): Obtained as an oil (2.31 g, 92% from 2.66 g of oxime). ^{1}H NMR ($CDCl_3$): 7.23 (td, 3H, *J* = 4.3, 2.5 Hz), 7.14–7.08 (m, 1H), 4.18 (q,

2H, J = 7.1 Hz), 3.71 (dd, 1H, J = 7.8, 5.5 Hz), 3.06 (dd, 1H, J = 13.6, 5.5 Hz), 2.86 (dd, 1H, J = 13.6, 7.8 Hz), 1.57 (s, 2H), 1.26 (t, 3H, J = 7.1 Hz). ^{13}C NMR ($CDCl_3$): 174.7, 139.5, 134.3, 129.7, 129.4, 127.5, 127.0, 61.0, 55.7, 40.7, 14.2. HRMS (*m/z*): $[M+H]^+$ calcd for $C_{11}H_{15}ClNO_2$: 228.0791, found, 228.0726.

Ethyl 2-amino-3-(4-chlorophenyl)propanoate (1v): Obtained as an oil (1.48 g, 85% from 1.84 g of oxime). ^{1}H NMR ($CDCl_3$): 7.29 (m, 2H), 7.16 (m, 2H), 4.18 (q, 2H, J = 7.3 Hz), 3.69 (dd, 1H, J = 5.6, 7.5 Hz), 3.05 (dd, 1H, J = 5.6, 13.7 Hz), 2.84 (dd, 1H, J = 7.5, 13.7 Hz), 1.47 (s(l), 2H), 1.26 (t, 3H, J = 7.1 Hz). ^{13}C NMR ($CDCl_3$): 174.8, 135.9, 132.7, 130.6, 128.6, 61.0, 55.7, 40.5, 14.2. HRMS (*m/z*): $[M+H]^+$ calcd for $C_{11}H_{15}ClNO_2$, 228.0791, found, 228.0806. Spectroscopic data similar to the reported one [5].

Ethyl 2-amino-3-(4-bromophenyl)propanoate (1w): Obtained as an oil (0.61 g, 86% from 0.74 g of oxime). ^{1}H NMR ($CDCl_3$): 7.44 (m, 2H), 7.10 (m, 2H), 4.18 (q, 2H, J = *7.3* Hz), 3.69 (dd, 1H, J = 5.3, 7.7 Hz), 3.04 (dd, 1H, J = 5.3, 13.7 Hz), 2.84 dd, 1H, J = 7.7, 13.7 Hz), 1.47 (s(l), 2H), 1.26 (t, 3H, J = *7.1* Hz). ^{13}C NMR ($CDCl_3$): 174.8, 136.4, 131.6, 131.0, 120.7, 61.0, 55.7, 40.5, 14.2. HRMS (*m/z*): $[M+H]^+$ calcd for $C_{11}H_{15}BrNO_2$, 272.0286, found, 272.0296. Spectroscopic data similar to the reported one [5].

Ethyl 2-amino-3-(2-methoxyphenyl)propanoate (1x): Obtained as an oil (2.47 g, 83% from 3.15 g of oxime). Analytical data identical with the one we reported previously [4].

Ethyl 2-amino-3-(3-methoxyphenyl)propanoate (1y): Obtained as an oil from 4.35 g of the crude compound **2y** described above (3.09 g, 75% from 3-methoxybenzaldehyde) with analytical data identical with the one we reported previously [4].

Ethyl 2-amino-3-(2-(benzyloxy)phenyl)propanoate (1z): Obtained as an oil (7.84 g, 91% from 8.94 g of oxime) ^{1}H NMR ($CDCl_3$): 7.46-7.33 (m, 5H), 7.24 (m, 1H), 6.87 (m, 3H), 5.08 (s, 2H), 4.19 (q, 2H, J = 7.1 Hz), 3.73 (dd, 1H, J = 5.1, 7.9 Hz), 3.09 (dd, 1H, J = 5.1, 13.4 Hz), 2.85 (dd, 1H, J = 7.9, 13.4 Hz), 1.52 (s (br), 2H), 1.27 (t, 3H, J = 7.1 Hz). ^{13}C NMR ($CDCl_3$): 174.9, 159.0, 139.0, 137.0, 129.5, 128.5, 127.9, 127.4, 121.9, 116.0, 113.1, 69.9, 60.9, 55.8, 41.2, 14.2. HRMS (*m/z*): $[M+H]^+$ calcd for $C_{18}H_{21}NO_3$: 300.1600, found, 300.1608.

Ethyl 2-amino-3-(3-(benzyloxy)phenyl)propanoate (1aa): Obtained as an oil (5.60 g, 92% from 6.33 g of oxime) ^{1}H NMR ($CDCl_3$): 7.48 (m, 2H), 7.41 (m, 2H), 7.34 (m, 1H), 7.25-7.18 (m, 2H), 6.94 (m, 2H), 5.13 (s, 2H), 4.12 (m, 2H), 3.88 (dd, 1H, *J* = 5.6, 8.5 Hz), 3.23 (dd, 1H, *J* = 5.6, 13.1 Hz), 2.88 (dd, 1H, *J* = 8.5, 13.1 Hz), 1.54 (s (br), 2H), 1.21 (t, 3H, *J* = 7.1 Hz). ^{13}C NMR ($CDCl_3$): 175.4, 156.8, 137.2, 131.3, 128.5, 128.1, 127.8, 127.0 (two signals), 126.4, 120.8, 111.8, 69.9, 60.7, 54.5, 36.8, 14.1. HRMS (*m/z*): $[M+H]^+$ calcd for $C_{18}H_{21}NO_3$: 300.1600, found, 300.1611.

Ethyl 2-amino-3-(4-(benzyloxy)phenyl)propanoate (1ab): Obtained as an oil (11.08 g, 95% from 12.13 g of oxime) with analytical data identical with the one we reported previously [4].

Ethyl 2-amino-3-(pyridin-2-yl)propanoate (1ac): Obtained as an oil (2.21 g, 56% from 4.28 g of oxime). ^{1}H NMR ($CDCl_3$): 8.55 (m, 1H), 7.61 (dt, 1H, *J* = 1.9, 7.6), 7.18 (m, 1H), 7.15 (m, 1H), 4.18 (q, 2H, *J* = 7.2 Hz), 3.98 (dd, 1H, *J* = 4.8, 8.2 Hz), 3.28 (dd, 1H, *J* = 4.8, 14.2 Hz), 3.04 (dd, 1H, *J* = 8.2, 14.2 Hz), 1.70 (s, 2H), 1.24 (t, 3H, *J* = 7.2 Hz). ^{13}C NMR ($CDCl_3$): 175.1, 158.1, 149.4, 136.3, 123.9, 121.6, 60.9, 54.5, 42.7, 14.1. HRMS (*m/z*): $[M+H]^+$ calcd for $C_{10}H_{15}N_2O_2$: 195.1134, found, 195.1062.

Ethyl 2-amino-3-(pyridin-3-yl)propanoate (1ad): Obtained as an oil (4.07 g, 63% from 6.87 g of oxime). ^{1}H NMR ($CDCl_3$): 8.49 (m, 2H), 7.56 (m, 1H), 7.22 (ddd, 1H, *J* = 0.8, 4.8, 5.6 Hz), 4.16 (q, 2H, *J* = 7.2 Hz), 3.70 (dd, 1H, *J* = 5.5, 7.5 Hz), 3.05 (dd, 1H, *J* = 5.5, 13.5 Hz), 2.88 (dd, 1H, *J* = 7.5, 13.5 Hz), 1.46 (s, 2H), 1.23 (t, 3H, *J* = 7.2 Hz). ^{13}C NMR ($CDCl_3$): 174.6, 150.6, 148.2, 136.7, 132.9, 123.2, 61.1, 55.5, 38.2, 14.1. HRMS (*m/z*): $[M+H]^+$ calcd for $C_{10}H_{15}N_2O_2$: 195.1134, found, 195.1131.

Ethyl 2-amino-3-(furan-2-yl)propanoate (1ae): Obtained as an oil (2.55 g, 89% from 3.08 g of oxime) with analytical data identical with the one we reported previously [4].

Ethyl 2-amino-3-(5-methylfuran-2-yl)propanoate (1af): Obtained as an oil (1.46 g, 88% from 1.76 g of oxime) with analytical data identical with the one we reported previously [4].

Ethyl 2-amino-3-(4,5-dimethylfuran-2-yl)propanoate (1ag): Obtained as an oil (0.79 g, 90% from 0.93 g of oxime). with analytical data identical with the one we reported previously [4].

Ethyl 2-amino-3-(5-ethylfuran-2-yl)propanoate (1ah): Obtained as an oil (1.87 g, 94% from 2.11 g of oxime) with analytical data identical with the one we reported previously [4].

Ethyl 2-amino-3-(5-(trifluoromethyl)furan-2-yl)propanoate (1ai): Obtained as a 95% pure oil (0.53 g, 93% from 0.60 g of oxime). ^{1}H NMR ($CDCl_3$): 6.66 (dd, 1H, *J* = 3.3, 1.2 Hz), 6.20 – 6.15 (m, 1H), 4.20 – 4.10 (m, 2H), 3.79 – 3.73 (m, 1H), 3.08 (dd, 1H, *J* = 15.0, 5.4 Hz), 2.99 (dd, 1H, *J* = 15.0, 7.1 Hz), 1.60 (s(br), 2H), 1.22 (t, 3H, *J* = 7.1 Hz). ^{13}C NMR ($CDCl_3$): 174.2, 154.7, 141.0 (q, *J* = 42.6 Hz), 119.0 (q, *J* = 266.6 Hz), 112.4 (q, *J* = 2.8 Hz), 108.5, 61.3, 53.3, 33.4, 14.0. HRMS (*m/z*): $[M+H]^+$ calcd for $C_{10}H_{13}F_3NO_3$, 252.0847, found, 252.0852.

Ethyl 2-amino-3-(5-ethylthiophen-2-yl)propanoate (1aj): Obtained as an oil (1.18 g, 92% from 1.36 g of oxime). ^{1}H NMR ($CDCl_3$): 6.66 (m, 1H), 6.61 (m, 1H), 4.21 (q, 2H, *J* = 5.1, 7.2 Hz), 3.68 (dd, 1H, *J* = 4.8, 7.3 Hz), 3.21 (dd, 1H, *J* = 4.8, 14.7 Hz), 3.04 (dd, 1H, *J* = 7.3, 14.7 Hz), 2.80 (m, 2H), 1.62 (s, 2H), 1.30 (t, 3H, *J* = 7.1 Hz), 1.29 (t, 3H, *J* = 7.5 Hz). ^{13}C NMR ($CDCl_3$): 174.6, 146.7, 136.2, 126.1, 123.0, 61.0, 55.7, 35.4, 23.4, 15.8, 14.2. HRMS (*m/z*): $[M+H]^+$ calcd for $C_{11}H_{18}NO_2S$: 228.1058, found, 228.1055.

Ethyl 2-amino-3-(3-methylthiophen-2-yl)propanoate (1ak): Obtained as an oil (1.10 g, 89% from 1.32 g of oxime). ^{1}H NMR ($CDCl_3$): 7.08 (d, 1H, *J* = 5.1 Hz), 6.80 (d, 1H, *J* = 5.1 Hz), 4.20 (m, 2H), 3.70 (dd, 1H, *J* = 5.1, 7.6 Hz), 3.23 (dd, 1H, *J* = 5.1, 14.7 Hz), 3.04 (dd, 1H, *J* = 7.6, 14.7 Hz), 2.20 (s, 3H), 1.59 (s, 2H), 1.28 (t, 3H, *J* = 7.6 Hz). ^{13}C NMR ($CDCl_3$): 174.6, 134.8, 132.5, 130.0, 122.6, 61.0, 55.8, 33.2, 14.1, 13.8. HRMS (*m/z*): $[M+H]^+$ calcd for $C_{10}H_{16}NO_2S$: 214.0902, found, 214.0901.

Ethyl 2-amino-3-(4,5-dimethylthiophen-2-yl)propanoate (1al): Obtained as an oil (0.46 g, 87%; 90% pure from 0.56 g of oxime). ^{1}H NMR ($CDCl_3$): 6.54 (s, 1H), 4.22 (d, 2H, *J* = 7.1 Hz), 3.66 (dd, 1H, *J* = 4.8, 7.4 Hz), 3.17 (dd, 1H, *J* = 4.8, 14.5 Hz), 3.03 (dd, 1H, *J* = 7.4, 14.5

Hz), 2.29 (s, 3H), 2.07 (s, 3H), 1.59 (s, 2H), 1.30 (t, 3H, J = 7.1 Hz). ^{13}C NMR ($CDCl_3$): 174.6, 133.9, 132.7, 131.7, 129.6, 61.0, 55.6, 35.1, 14.2, 13.4, 12.9. HRMS (*m/z*): $[M+H]^+$ calcd for $C_{11}H_{18}NO_2S$: 228.1058, found, 228.1053.

Ethyl 2-(hydroxyimino)-3-(tetrahydrofuran-2-yl)propanoate (9): This compound was obtained when using 10% Pd/C for the hydrogenation step described above for the general preparation of oximes **2** but letting it run 48 hours. Out a 12.8 g batch, only a sample (0.68 g) was purified by a chromatography (dichloromethane – ethanol 97/3) to give compound **9** an oil (0.56 g). ^{1}H NMR ($CDCl_3$): 9.74 (s, 1H), 4.32 (q, 2H, J = 7.2 Hz), 4.28 (m, 1H), 3.90 (m, 1H), 3.74 (m, 1H), 2.96 (dd, 1H, J = 5.5, 12.8 Hz), 2.80 (dd, 1H, J = 5.9, 12.8 Hz), 1.88 (m, 3H), 1.62 (m, 1H), 1.35 (t, 3H, J = 7.2 Hz). ^{13}C NMR ($CDCl_3$): 163.6, 150.4, 67.7, 61.7, 58.4, 31.2, 30.6, 25.3, 14.0. HRMS (*m/z*): $[M+Na]^+$ calcd for $C_9H_{15}NO_4Na$: 224.0899, found, 224.0874.

Ethyl 2-amino-3-(tetrahydrofuran-2-yl)propanoate (10): This compound was obtained by following the general reduction protocol for the preparation of compounds **1** described above, as an oil (5.68 g, 35% from 8.28 g of furfural) as a mixture of two diasteroisomers in a 3/2 ratio. ^{1}H NMR ($CDCl_3$): Major isomer: 4.17 (q, 2H, J = 7.3), 4.01 (m, 1H), 3.85 (m, 1H), 3.72 (m, 1H), 3.62 (dd, 1H, J = 8.9, 3.5 Hz), 2.03 (m, 1H), 1.89 (m, 2H), 1.68 (ddd, 2H, J = 3.5, 8.9, 13.8 Hz), 1.68 (s, 2H), 1.51 (m, 1H), 1.27 (t, 3H, J = 7.3 Hz). ^{13}C NMR ($CDCl_3$): 176.1, 75.8, 67.6, 60.8, 52.5, 40.2, 31.7, 25.6, 14.2. Minor isomer: 4.17 (q, 2H, J = 7.2), 4.01 (m, 1H), 3.85 (m, 1H), 3.72 (m, 1H), 3.56 (dd, 1H, J = 7.3, 5.4), 2.03 (m, 1H), 1.89 (m, 2H), 1.80 (m, 2H), 1.61 (s, 2H), 1.49 (m, 1H), 1.27 (t, 3H, J = 7.2 Hz). ^{13}C NMR ($CDCl_3$): 175.6, 76.9, 67.7, 60.8, 53.3, 40.5, 31.7, 25.4, 14.2. HRMS (*m/z*): $[M+H]^+$ calcd for $C_9H_{18}NO_3$: 188.1287, found, 188.1258.

Isolation of diethyl 2-(furan-2-ylmethyl)malonate (3ae): In this specific case, the following protocol was used: The ethanol solution obtained as described above and containing compound **6ae** was concentrated to dryness thoroughly. At 0 °C, under a calcium chloride-protected atmosphere, the resulting oil (5.86 g, 0.0246 mol) was dissolved in dry THF (100

mL) and sodium borohydride (0.65 g, 0.0172 mol) was added. This was stirred at 0 °C for 24 hours, diluted with water and ethyl acetate and made cautiously acidic with acetic acid. The resulting solution was extracted twice with ethyl acetate, the organic layer was washed with water, brine, dried over magnesium sulfate and concentrated to dryness. The residue was purified by column chromatography over silica gel (cyclohexane – dichloromethane 2/3 to 1/4) to yield compound **3ae** as an oil (2.91 g, 49%). ^{1}H NMR ($CDCl_3$): 7.31 (dd, 1H, *J* = 2.0, 0.9 Hz), 6.27 (dd, 1H, *J* = 3.2, 2.0 Hz), 6.08 (m, 1H), 4.21 (qd, 2H, *J* = 7.1, 1.5 Hz), 3.73 (t, 1H, *J* = 7.6 Hz), 3.06 (d, 2H, *J* = 7.6 Hz), 1.26 (t, 3H, *J* = 7.1 Hz).

Isolation of compounds 11 and 12: The crude mixtures resulting from the various trials to improve the reduction of **6ae** into **3ae** mentioned in the text and all containing ^{1}H NMR signals corresponding to compound **11** and **12** were gathered and purified by a chromatography over silica gel (cyclohexane – dichloromethane 2/1 to 0/1 to dichloromethane / ethanol 95/5) and the most polar fraction subjected to a second chromatography over silica gel (cyclohexane – ethyl acetate 4/1 to 1/2) to yield samples of compounds **11** (0.85 g, 9%) and **12** (0.21 g, 3%) as described below.

Ethyl 3-(furan-2-yl)-2-(hydroxymethyl)propanoate (11): ^{1}H NMR ($CDCl_3$): 7.33 (dd, 1H, *J* = 1.9, 0.9 Hz), 6.30 (dd, 1H, *J* = 3.2, 1.9 Hz), 6.09 (m, 1H), 4.20 (qd, 2H, *J* = 7.1, 2.1 Hz), 3.79 (m, 2H), 3.06 (m, 1H), 2.96 (m, 2H), 2.21 (t, 1H, *J* = 6.5 Hz), 1.28 (t, 3H, *J* = 7.1 Hz). ^{13}C NMR ($CDCl_3$): 174.1, 152.4, 141.5, 110.2, 106.7, 62.4, 60.9, 46.5, 26.8, 14.1. HRMS (*m/z*): $[M+Na]^+$ calcd for $C_{10}H_{14}O_4Na$: 221.0790, found, 221.0794.

2-(Furan-2-ylmethyl)propane-1,3-diol (12): ^{1}H NMR ($CDCl_3$): 7.33 (dd, 1H, *J* = 1.9, 0.9 Hz), 6.31 (dd, 1H, *J* = 3.2, 1.9 Hz), 6.07 (m, 1H), 3.82 (dd, 2H, *J* = 10.8, 4.2 Hz), 3.70 (dd, 2H, *J* = 10.8, 6.5 Hz), 2.73 (s, 1H), 2.71 (s, 1H), 2.34 (s, 2H), 2.15 (m, 1H). ^{13}C NMR ($CDCl_3$): 153.8, 141.3, 110.3, 106.3, 65.2, 41.7, 26.5. HRMS (*m/z*): $[M+Na]^+$ calcd for $C_8H_{12}O_3Na$: 179.0684, found, 179.0685.

Preparation of compounds 14, 18 or 30: Step 1 Preparation of the brominated arylidenemalonate **13**, **17** or **29**. By using the Knoevenagel condensation described above,

these compounds were obtained respectively from 3-bromobenzaldehyde, 4-bromobenzaldehyde or 5-bromofuran-2-carbaldehyde and following a concentration to dryness, used directly. Step 2: cyclopropanation. The considered brominated alkylidenemalonate (14.8 mmol), cyclopropylboronic acid (1.66 g, 19.3 mmol) and caesium carbonate (18.84 g, 57.8 mmol) were dispersed in a 95/5 mixture of toluene and water (120 mL). This was degassed by passing a gentle stream of argon through the suspension, [1,1'-bis(diphenylphosphino)ferrocene] dichloropalladium complexed with dichloromethane (0.30 g, 0.36 mmol) was then added and this was heated to reflux under argon for 50 minutes. The resulting dark solution was diluted in ethyl acetate, washed with water, brine, dried over magnesium sulfate and concentrated to dryness. The residues were purified as described below.

Diethyl 2-(3-cyclopropylbenzylidene)malonate (14): This compound was obtained as an oil (4.68 g, 60% from 3-bromobenzaldehyde; 90 % pure) after a chromatography over silica gel (cyclohexane – ethyl acetate 95/5). ^{1}H NMR ($CDCl_3$): 7.72 (s, 1H), 7.27 (m, 2H), 7.17 (m, 1H), 7.11 (m, 1H), 4.36 (q, 2H, *J* = 7.1 Hz), 4.32 (q, 2H, *J* = 7.1 Hz), 1.90 (m, 1H), 1.35 (t, 3H, *J* = 7.1 Hz), 1.32 (t, 3H, *J* = 7.1 Hz), 0.99 (m, 2H), 0.69 (m, 2H). ^{13}C NMR ($CDCl_3$): 166.7, 164.2, 144.6, 142.3, 132.9, 128.6, 128.1, 126.8, 128.5, 126.2, 61.6, 61.5, 15.2, 14.1, 13.9, 9.2.

Diethyl 2-(4-cyclopropylbenzylidene)malonate (18): This compound was obtained as an oil (2.05 g, 47% from 4-bromobenzaldehyde) after a chromatography over silica gel (cyclohexane – ethyl acetate 95/5). ^{1}H NMR ($CDCl_3$): 7.70 (s, 1H), 7.37 (m, 2H), 7.08 (m, 2H), 4.36 (q, 2H, *J* = 7.1 Hz), 4.31 (q, 2H, *J* = 7.1 Hz), 1.91 (m, 1H), 1.33 (t, 6H, *J* = 7.1 Hz), 1.04 (m, 2H), 0.75 (m, 2H). ^{13}C NMR ($CDCl_3$): 166.9, 164.3, 147.6, 142.0, 130.0, 129.7, 125.9, 124.9, 61.6, 61.5, 15.6, 14.1, 13.9, 10.0 (two signals missing). HRMS (*m/z*): $[M+Na]^+$ calcd for $C_{17}H_{20}O_4Na$, 311.1259, found, 311.1258.

Diethyl 2-((5-cyclopropylfuran-2-yl)methylene)malonate (30): This compound was obtained as an oil (3.12 g, 75% from 5-bromofuran-2-carbaldehyde) after a chromatography

over silica gel (cyclohexane – ethyl acetate 8/1). ^{1}H NMR ($CDCl_3$): 7.32 (s, 1H), 6.65 (d, 1H, *J* = 3.4 Hz), 6.09 (d, 1H, *J* = 3.4 Hz), 4.37 (q, 2H, *J* = 7.1 Hz), 4.25 (q, 2H, *J* = 7.1 Hz), 1.89 (tt, 1H, *J* = 8.4, 5.0 Hz), 1.37 (t, 3H, *J* = 7.1 Hz), 1.30 (t, 3H, *J* = 7.1 Hz), 1.01 – 0.93 (m, 2H), 0.88 – 0.79 (m, 2H). ^{13}C NMR ($CDCl_3$): 166.8, 164.7, 162.5, 147.0, 127.4, 120.1, 119.9, 107.3, 61.5, 61.4, 14.3, 14.2, 9.7, 8.3, (one signal missing). HRMS (*m/z*): $[M+Na]^+$ calcd for $C_{15}H_{18}O_5Na$, 301.1052, found, 301.1034.

Preparations of oximes 21 or 25: The considered cyclopropyl-bearing arylidenemalonate **14** or **18** were reduced using two days-long catalytic hydrogenation with 10% palladium over charcoal as described above and the resulting compounds were then used directly to prepare the corresponding oximes as described above for the preparation of compounds **2** to give the target oximes as further described below.

Ethyl-2-(hydroxyimino)-3-(3-propylphenyl)propanoate (21): This compound was obtained as a white solid (1.40 g, 36%, un-optimized) after a chromatography over silica gel (cyclohexane – ethyl acetate 5/1). ^{1}H NMR ($CDCl_3$): 9.74 (bs, 1H), 7.19 (m, 3H), 7.05 (m, 1H), 4.31 (q, 2H, *J* = 7.2 Hz), 3.99 (s, 2H), 2.58 (m, 2H), 1.65 (m, 2H), 1.34 (t, 3H, *J* = 7.2 Hz), 0.96 (t, 3H, *J* = 7.3 Hz). ^{13}C NMR ($CDCl_3$): 163.3, 151.6, 143.0, 135.5, 129.3, 128.3, 126.8, 126.4, 61.9, 38.0, 30.5, 24.5, 14.0, 13.8. HRMS (*m/z*): $[M+Na]^+$ calcd for $C_{14}H_{19}NO_3Na$, 272.1263, found, 272.1250.

Ethyl-2-(hydroxyimino)-3-(4-propylphenyl)propanoate (25): This compound was obtained as a white solid (2.64 g, 69%) after a chromatography over silica gel (cyclohexane – ethyl acetate 6/1). ^{1}H NMR ($CDCl_3$): 9.64 (s, 1H), 7.26 (m, 2H), 7.11 (m, 2H), 4.31 (q, 2H, *J* = 7.2 Hz), 3.98 (s, 2H), 2.57 (m, 2H), 1.64 (m, 2H), 1.34 (t, 3H, *J* = 7.2 Hz), 0.95 (t, 3H, *J* =7.3 Hz). ^{13}C NMR ($CDCl_3$): 163.3, 151.2, 141.0, 132.8, 129.0, 128.6, 61.8, 37.7, 30.1, 24.5, 14.0, 13.8. HRMS (*m/z*): $[M+Na]^+$ calcd for $C_{14}H_{19}NO_3Na$, 272.1263, found, 272.1276.

Preparations of oximes 23, 27 or 32: The considered cyclopropyl-bearing arylidenemalonates **14**, **18** or **30** were reduced using sodium borohydride overnight at 4 °C as described above and the resulting malonates **16**, **20** or **31** were then used directly to

prepare the corresponding oxime as described above for the preparation of compounds **2** to give the target oximes which were purified and characterized as described below.

Ethyl 3-(3-cyclopropylphenyl)-2-(hydroxyimino)propanoate (23): This compound was obtained as a colourless oil (1.72 g, 50% from compound **14**) after a chromatography over silica gel (cyclohexane – ethyl acetate 6/1). ^{1}H NMR ($CDCl_3$): 9.99 (bs, 1H), 7.20 (m, 3H), 6.91 (m, 1H), 4.31 (q, 2H, *J* = 7.2 Hz), 3.97 (s, 2H), 2.58 (m, 2H), 1.88 (m, 1H), 1.34 (t, 3H, *J* = 7.2 Hz), 0.96 (m, 2H), 0.69 (m, 2H). ^{13}C NMR ($CDCl_3$): 163.3, 151.0, 144.2, 135.6, 128.4, 126.8, 126.2, 123.8, 61.9, 30.4, 15.3, 14.0, 9.1. HRMS (*m/z*): $[M+Na]^+$ calcd for $C_{14}H_{17}NO_3Na$, 270.1106, found, 270.1112.

Ethyl 3-(4-cyclopropylphenyl)-2-(hydroxyimino)propanoate (27): This compound was obtained as a pale yellow solid (1.03 g, 88% from compound **18**) after a chromatography over silica gel (cyclohexane – ethyl acetate 6/1). ^{1}H NMR ($CDCl_3$): 9.31 (s, 1H), 7.22 (m, 2H), 6.99 (m, 2H), 4.29 (q, 2H, *J* = 7.2 Hz), 3.95 (s, 2H), 1.86 (m, 1H), 1.33 (t, 3H, *J* = 7.2 Hz), 0.93 (m, 2H), 0.67 (m, 2H). ^{13}C NMR ($CDCl_3$): 163.3, 151.2, 142.3, 132.6, 129.1, 125.8, 61.8, 30.0, 15.0, 14.0, 9.0. HRMS (*m/z*): $[M+Na]^+$ calcd for $C_{14}H_{17}NO_3Na$, 270.1106, found, 270.1109.

Ethyl 3-(5-cyclopropylfuran-2-yl)-2-(hydroxyimino)propanoate (32): This compound was obtained as an orange solid (1.09 g, 44% from compound **30**) after a chromatography over silica gel (cyclohexane – ethyl acetate 4/1). ^{1}H NMR ($CDCl_3$): ^{1}H NMR ($CDCl_3$): 9.42 (bs, 1H), 5.97 (m, 1H), 5.84 (d, 1H, *J* = 8.1 Hz), 4.33 (q, 2H, *J* = 7.1 Hz), 3.97 (s, 2H), 1.84 (m, 1H), 1.36 (t, 3H, *J* = 7.1 Hz), 0.84 (m, 2H), 0.73 (m, 2H). ^{13}C NMR ($CDCl_3$): 163.0, 156.5, 148.8, 146.3, 107.5, 104.2, 61.9, 23.7, 14.0, 8.7, 6.5. HRMS (*m/z*): HRMS (*m/z*): $[M+Na]^+$ calcd for $C_{12}H_{15}NO_4Na$, 260.0899, found, 260.0892.

Preparation of compounds 22, 24, 26, 28 and 33: By using the general protocol described above for the reduction of compounds **2** into **1**, compounds **22**, **24**, **26**, **28** and **33** were isolated and characterized as described below.

Ethyl 2-amino-3-(3-propylphenyl)propanoate hydrochloride (22): Obtained as an oil which was immediately treated with a solution of hydrogen chloride in dioxane and concentrated to dryness to give the hydrochloride salt as a white powder (1.89 g 90%; still containing some ethanol). ^{1}H NMR (DMSO-d_6): 8.72 (bs, 3H), 7.24 (m, 1H), 7.08 (m, 3H), 4.18 (dd, 1H, J = 7.9, 5.6 Hz), 4.08 (q, 2H, J = 7.2 Hz), 3.21 (dd, 1H, J = 13.9, 5.6 Hz), 3.03 (dd, 1H, J = 13.9, 7.9 Hz), 2.53 (m, 2H), 1.58 (m, 2H), 1.08 (t, 3H, J = 7.2 Hz), 0.89 (t, 3H, J = 7.3 Hz). ^{13}C NMR (DMSO-d6): 169.4, 142.8, 135.0, 129.9, 128.9, 127.7, 127.2, 62.0, 53.7, 37.6, 36.5, 24.4, 14.2, 14.1. HRMS (*m/z*): $[M+H]^+$ calcd for $C_{14}H_{22}NO_2$: 236.1651, found, 236.1637.

Ethyl 2-amino-3-(3-cyclopropylphenyl)propanoate (24): Obtained as an oil (1.66 g, 89%). ^{1}H NMR ($CDCl_3$): 7.19 (m, 1H), 6.95 (m, 3H), 4.19 (q, 2H, J = 7.0 Hz), 3.71 (dd, 1H, J = 5.2, 7.9 Hz), 3.05 (dd, 1H, J = 5.2, 13.5 Hz), 2.83 (dd, 1H, J = 7.9, 13.5 Hz), 1.88 (m, 1H), 1.53 (s, 2H), 1.28 (t, 3H, J = 7.0 Hz), 0.95 (m, 2H), 0.68 (m, 2H). ^{13}C NMR ($CDCl_3$): 175.0, 144.3, 137.2, 128.4, 126.8, 126.3, 124.0, 60.9, 55.8, 41.2, 15.3, 14.2, 9.2, 9.1. HRMS (m/z): $[M+H]^+$ calcd for $C_{14}H_{20}NO_2$: 234.1494, found, 234.1496.

Ethyl 2-amino-3-(4-propylphenyl)propanoate hydrochloride (26): Obtained as an oil which was immediately treated with a solution of hydrogen chloride in dioxane and concentrated to dryness to give the hydrochloride salt as a white powder (2.46 g, 85%; still containing some ethanol). ^{1}H NMR (DMSO-d_6): 8.59 (bs, 3H), 7.15 (s, 4H), 4.20 (dd, 1H, J = 7.5, 5.9 Hz), 4.10 (m, 2H), 3.16 (dd, 1H, J = 7.9, 5.9 Hz), 3.02 (dd, 1H, J = 14.2, 7.9 Hz), 2.54 (m, 2H), 1.58 (m, 2H), 1.09 (t, 3H, J = 7.2 Hz), 0.89 (t, 3H, J = 7.3 Hz). ^{13}C NMR (DMSO-d6): 169.5, 142.6, 132.3, 129.8, 129.0, 62.0, 53.7, 37.3, 36.2, 24.5, 14.2, 14.0. HRMS (*m/z*): $[M+H]^+$ calcd for $C_{14}H_{22}NO_2$: 236.1651, found, 236.1659.

Ethyl 2-amino-3-(4-cyclopropylphenyl)propanoate (28): Obtained as an oil (0.71 g, 80%). ^{1}H NMR ($CDCl_3$): 7.09 (m, 2H), 7.01 (m, 2H), 4.18 (q, 2H, J = 7.0 Hz), 3.70 (dd, 1H, J = 5.2, 7.9 Hz), 3.06 (dd, 1H, J = 5.2, 13.7 Hz), 2.83 (dd, 1H, J = 7.9, 13.7 Hz), 1.87 (m, 1H), 1.54 (s, 2H), 1.28 (t, 3H, J = 7.0 Hz), 0.95 (m, 2H), 0.68 (m, 2H). ^{13}C NMR ($CDCl_3$): 175.0, 142.4,

134.2, 129.2, 125.9, 60.9, 55.9, 40.6, 15.0, 14.2, 9.0. HRMS (*m/z*): $[M+H]^+$ calcd for $C_{14}H_{20}NO_2$: 234.1494, found, 234.1490.

Ethyl 2-amino-3-(5-cyclopropylfuran-2-yl)propanoate (33): Obtained as an oil (0.70 g, 88%; 93% pure). ^1H NMR ($CDCl_3$): 5.99 (d, 1H, *J* = 3.1 Hz), 5.85 (d, 1H, *J* = 3.1 Hz), 4.20 (q, 2H, *J* = 7.2 Hz), 3.74 (dd, 1H, *J* = 7.1, 5.1 Hz), 3.03 (dd, 1H, *J* = 14.8, 5.1 Hz), 2.93 (dd, 1H, *J* = 14.8, 7.1 Hz), 1.84 (m, 1H), 1.36 (t, 3H, *J* = 7.2 Hz), 0.84 (m, 2H), 0.73 (m, 2H). ^{13}C NMR ($CDCl_3$): 174.7, 156.7, 149.0, 108.2, 104.0, 60.9, 53.7, 33.3, 14.1, 8.7, 6.4. HRMS (*m/z*): $[M+H]^+$ calcd for $C_{12}H_{17}NO_3$, 224.1287, found, 224.1282.

Diethyl 2-(1-(furan-2-yl)ethyl)malonate (34): The ethanol solution containing compound **6ae** and obtained as described above was concentrated to dryness thoroughly to give an oil (4.51 g, 0.0189 mol). This was dissolved under argon in dry THF (30 mL), cooled to 0 °C and a 3 M solution of methyl magnesium chloride in THF (16 mL, 0.0473 mol) was slowly added. This was stirred for 15 minutes at 0 °C, cautiously quenched with ethanol, diluted in water made acid with a 1 N solution of hydrochloric acid and extracted with ethyl acetate. The organic layer was washed with water, brine, dried over magnesium sulfate, concentrated to dryness and the residue purified by a chromatography over silica gel (cyclohexane – dichloromethane 4/1) to yield compound **34** as a 95% pure oil (3.41 g, 70%). ^1H NMR ($CDCl_3$): 7.32 (t, 1H, *J* = 1.8, 0.9 Hz), 6.28 (dd, 1H, *J* = 3.2, 1.8 Hz), 6.10 – 6.03 (m, 1H), 4.22 (q, 2H, *J* = 7.1 Hz), 4.10 (m, 2H), 3.70 – 3.64 (m, 2H), 1.42 – 1.30 (m, 3H), 1.28 (t, 3H, *J* = 7.1 Hz), 1.18 (t, 3H, *J* = 7.1 Hz). ^{13}C NMR ($CDCl_3$): 168.1, 168.0, 156.1, 141.3, 110.0, 105.4, 61.4, 61.3, 57.0, 33.5, 17.0, 14.0, 13.9. HRMS (*m/z*): $[M+Na]^+$ calcd for $C_{13}H_{18}O_5Na$, 277.1052, found, 277.1052. ^1H NMR data similar to the reported one [8].

Ethyl 2-(hydroxyimino)-3-phenylbutanoate (37): Step 1: preparation of diethyl 2-(1-phenylethyl)malonate (**36**): under a calcium chloride guard, diethyl malonate (5.32 g, 0.033 mol) was dissolved in dry DMF (50 mL, dried over 4 Å molecular sieves) and 60% sodium hydride in mineral oil (1.39 g, 0.0348 mol) was added portion-wise while maintaining the solution temperature at 20 °C with a water bath. This was stirred until the end of hydrogen

evolution and (1-chloroethyl)benzene 4.8 mL, 0.036 mol) was added. This was stirred for 7 days, diluted in water and ethyl acetate, the organic layer was washed 5 times with water, brine, dried over magnesium sulfate and concentrated to dryness under high vacuum to remove most of the unreacted diethyl malonate and (1-chloroethyl)benzene to give an oil (6.22 g) pure enough for the next step. Step 2: reaction with isoamylnitrite. Under an inert atmosphere, 4.68 g of the resulting oil was dissolved in dry ethanol (50 mL, dried over 4 Å molecular sieves) and cooled at 0 °C with ice. A solution of 21% sodium ethoxide in ethanol (7.7 mL, 0.0207 mol) was added followed by isoamylnitrite (2.6 mL, 0.0194 mol). This was allowed to warm to room temperature and stirred for 16 hours. The resulting solution was then treated as described above for the general preparation of α-hydroxyimino esters **2** and compound **37** was obtained as an oil (1.07 g, 14% from diethylmalonate) after a chromatography over silica gel (cyclohexane – ethyl acetate 5/1). ^{1}H NMR ($CDCl_3$, for once it is a separable but slowly equilibrating mixture of the two oxime isomers): 10.69 (s, 0.2H), 9.72 (s, 0.8H), 4.82 (q, 0.8H, *J* = 7.2 Hz), 4.21 (m, 2H), 4.02 (q, 0.2H, *J* = 7.2 Hz), 1.68 (d, 2.4H, *J* = 7.2 Hz), 1.54 (d, 0.6H, *J* = 7.2 Hz), 1.26 (t, 2.4H, *J* = 7.3 Hz), 1.15 (t, 0.6H, *J* = 7.3 Hz). ^{13}C NMR ($CDCl_3$, major/minor: 162.6/163.3, 154.3/152.3, 140.6/141.2, 128.5/128.6, 127.6/127.7, 126.6/127.0, 61.5/61.6, 35.0/42.1, 15.5/18.7, 13.9/13.7. HRMS (*m/z*): $[M+Na]^+$ calcd for $C_{12}H_{15}NO_3Na$, 244.0950, found, 244.0939.

Ethyl 2-amino-3-phenylbutanoate (38): By using the general protocol described above for the reduction of compounds **2** into **1**, this compound was obtained as an oil (0.76 g, 88%) containing a 1/2 mixture of two diasteroisomers. ^{1}H NMR ($CDCl_3$): 7.39-7.21 (m, 5H), 4.20 (q, 1.3H, *J* = 7.1 Hz), 4.20 (q, 0.7H, *J* = 7.1 Hz), 3.62 (d, 0.3H, *J* = 6.0 Hz), 3.56 (d, 0.7H, *J* = 7.2 Hz), 3.18 (m, 0.3H), 3.12 (m, 0.3H), 1.44 (s, 2H), 1.36 (d, 2H, *J* = 7.2 Hz), 1.34 (d, 1H, *J* = 6.0 Hz), 1.29 (t, 2H, *J* = 7.1 Hz), 1.16 (t, 1H, *J* = 7.1 Hz). ^{13}C NMR ($CDCl_3$) major diastereoisomer: 174.7, 142.2, 128.5, 127.9, 126.9, 60.8, 60.6, 44.4, 18.4, 14.2. minor diastereoisomer: 174.6, 143.0, 128.3, 127.7, 126.7, 60.7, 60.4, 43.5, 15.1, 14.0. HRMS (*m/z*): $[M+H]^+$ calcd for $C_{12}H_{18}NO_2$, 208.1338, found, 208.1331.

Ethyl 2-(hydroxyimino)-3-methyl-3-phenylbutanoate (40): Under an inert atmosphere, ethyl 3-methyl-3-phenylbutanoate [9,10] (1.38 g, 6.68 mmol) was dissolved in dry THF (20 mL). This solution was cooled to −78 °C and a 2 N solution of lithium diisopropylamide in THF (3.5 mL, 7.02 mmol) was added. This was stirred for 5 minutes and isoamylnitrite (1 mL, 7.35 mmol) was added. The solution was allowed to warm to room temperature and stirred for 24 hours. This was diluted in water, made acid with 1 N hydrochloric acid and extracted with ethyl acetate. The organic layer was washed with water, brine, dried over magnesium sulfate and concentrated to dryness. Under an inert atmosphere, this residue was diluted in dry ethanol (10 mL, dried over 4 Å molecular sieves), a 21% solution of sodium ethoxide in ethanol (1 mL) was added and this was heated to reflux for 7 hours. The resulting "transesterified" solution was diluted with water, made acidic with 1 N hydrochloric acid and extracted with ethyl acetate. The organic layer was washed with water, brine, dried over magnesium sulfate and concentrated to dryness and purified by a chromatography over silica gel (cyclohexane – ethyl acetate 7/1 to 4/1) to yield the target oxime as an oil (0.24 g, 15%). ^{1}H NMR ($CDCl_3$): 8.57 (s, 1H), 7.35 (m, 4H), 7.21 (m, 1H), 4.11 (q, 2H, J = 7.1 Hz), 1.62 (s, 6H), 1.06 (t, 3H, J = 7.1 Hz). ^{13}C NMR ($CDCl_3$): 163.4, 158.6, 144.3, 128.2, 126.7, 126.4, 61.2, 42.9, 27.1, 13.7. HRMS (*m/z*): $[M+H]^+$ calcd for $C_{13}H_{17}NO_3Na$, 258.1106, found, 258.1087.

Ethyl 2-amino-3-methyl-3-phenylbutanoate (41): Compound **40** (0.23 g, 0.97 mmol) was dissolved in ethanol (5 mL); To this solution was added 37% hydrochloric acid (0.72 mL, 8.79 mmol) and powdered zinc (0.19 g, 2.93 mmol). This was stirred for 12 hours and another portion of 37% hydrochloric acid (0.72 mL, 8.79 mmol) followed by powdered zinc (0.19 g, 2.93 mmol) were added. Stirring at room temperature was resumed for 8 hours, the resulting solution was diluted in water, made basic with 33% ammonia, extracted with ethyl acetate, the organic layer was washed with water, brine, dried over sodium carbonate and concentrated to dryness to yield compound **41** as an oil (0.20 g, 92%). ^{1}H NMR ($CDCl_3$): 7.37 (m, 4H), 7.24 (m, 1H), 4.07 (q, 2H, J = 7.2 Hz), 3.65 (s, 1H), 1.43 (s, 6H), 1.15 (t, 3H, J

= 7.2 Hz). ^{13}C NMR ($CDCl_3$): 173.9, 146.3, 128.2, 126.4, 126.3, 64.0, 60.5, 41.9, 25.5, 23.3, 14.0. HRMS (*m/z*): $[M+H]^+$ calcd for $C_{13}H_{20}NO_2$, 222.1494, found, 222.1490.

Ethyl hydroxyphenylalaninate (42): Compound **2a** (0.1 g, 0.483 mmol) and trifluoromethane sulfonic acid (0.64 mL, 7.2 mmol) were dissolved in diethyl ether (10 mL). To this was added an initial 2 equivalents of triethylsilane (0.15 mL, 0.965 mmol) and following the monitoring of the reaction by LC/MS, additional equivalent of triethylsilane were added to reach a sum of 6 equivalents on day six. Following another day of stirring, water and 1 N hydrochloric acid were added to the resulting solution. This was stirred for 15 minutes made basic by adding 1 N sodium bicarbonate and extracted with ethyl acetate. The organic layer was washed with water, brine, concentrated to dryness and purified by a chromatography over silica gel (dichloromethane – ethanol 98/2, TLC detection using a $KMnO_4$ solution) to yield compound **42** as a film (0.07 g, 69%). ^{1}H NMR ($CDCl_3$): 7.34-7.21 (m, 5H), 5.87 (s, 1H), 4.21 (q, 2H, *J* = 7.2 Hz), 3.89 (dd, 1H, *J* = 5.9 and 8.5 Hz), 3.01 (dd, 1H, *J* = 6.0 et 14.1 Hz), 2.92 (dd, 1H, *J* = 8.2 et 14.0 Hz), 1.24 (t, 3H, *J* = 8.0). ^{13}C NMR ($CDCl_3$): 172.9, 136.6, 129.1, 128.6, 126.9, 66.2, 61.1, 35.5, 14.1. HRMS (*m/z*): $[M+H]^+$ calcd for $C_{11}H_{16}NO_3$, 210.1130, found, 210.1136. ^{1}H NMR data similar to the reported one [11].

General protocol for the preparation of malonates 44a and 44b: Step 1: preparation of diethyl 2-(prop-2-yn-1-yl)malonate (**43**). Diethylmalonate (9.16 g, 0.057 mol), 80% propargyl bromide in toluene (6.36 mL, 0.057 mol) and potassium carbonate (8.7 g, 0.063 mol) were stirred in dry acetone (100 mL, dried over 4 Å molecular sieves) for 48 hours. The suspension was filtered and concentrated to dryness to give and oil still (15.52 g) containing about 55% of unreacted diethyl malonate. Since all our trials failed to purify this mixture, this was used directly. Step 2: In a calcium chloride-protected atmosphere, the crude diethyl 2-(prop-2-yn-1-yl)malonate described above (15.5 g), the relevant nitroalkane (0.078 mol) and phenylisocyanate (15.3 mL, 0.14 mol) were dissolved in dichloromethane (200 mL). To this solution were added 10 drops of triethylamine and this was stirred for 48 hours at 20 °C. The

resulting suspension was filtered, the filtrate concentrate to dryness and the residue further purified as described below.

Diethyl 2-((3-methylisoxazol-5-yl)methyl)malonate (44a): This compound was obtained as an oil (3.47 g, 23% from 1-nitroethane; still containing some aryl-bearing impurity) after a chromatography over silica gel (cyclohexane – ethyl acetate 5/1) and extensive drying under vacuum at 80 °C to distil off some 3,4-dimethyl-1,2,5-oxadiazole 2-oxide. ^{1}H NMR ($CDCl_3$) 5.90 (s, 1H), 4.22 (m, 4H), 3.77 (t, 1H, J = 7.6 Hz), 3.33 (d, 2H, J = 7.6 Hz), 2.26 (s,3H), 1.27 (t, 6H, J = 7.1 Hz). ^{13}C NMR ($CDCl_3$): 168.9, 168.0, 159.8, 102.9, 61.9, 50.2, 25.9, 13.9, 11.3. HRMS (*m/z*): $[M+Na]^+$ calcd for $C_{12}H_{17}NO_5Na$, 278.1004, found, 278.1003.

Diethyl 2-((3-ethylisoxazol-5-yl)methyl)malonate (44b): This compound was obtained as an oil (5.8 g, 33% from 1-nitropropane; still containing some aryl-bearing impurity) after a chromatography over silica gel (cyclohexane – ethyl acetate 5/1). ^{1}H NMR ($CDCl_3$) 5.93 (s, 1H), 4.22 (m, 4H), 3.78 (t, 1H, J = 7.6 Hz), 3.34 (d, 2H, J = 7.6 Hz), 2.66 (q, 2H, J = 7.6 Hz), 1.27 (m, 9H). ^{13}C NMR ($CDCl_3$): 168.9, 168.0, 159.8, 102.9, 61.9, 50.2, 25.9, 13.9, 11.3. ^{13}C NMR ($CDCl_3$): 168.9, 168.0, 159.8, 102.9, 61.9, 50.2, 25.9, 13.9, 11.3. HRMS (*m/z*): $[M+Na]^+$ calcd for $C_{13}H_{19}NO_5Na$, 292.1161, found, 292.1161.

Ethyl 2-(hydroxyimino)-3-(3-methylisoxazol-5-yl)propanoate (45a): This compound was obtained when following the general protocol for the transformation of compounds **3** into **2** as a solid (3.39 g, 50%) after a chromatography over silica gel (cyclohexane – ethyl acetate 9/1). ^{1}H NMR ($CDCl_3$): 10.17 (s(br), 1H), 5.92 (s, 1H), 4.34 (q, 2H, J = 7.2 Hz), 4.10 (s, 2H), 2.26 (s, 3H), 1.35 (t, 3H, J = 7.2 Hz). ^{13}C NMR ($CDCl_3$): 166.2, 162.6, 160.0, 146.8, 103.1, 62.3, 22.5, 14.0, 11.3. HRMS (*m/z*): $[M+Na]^+$ calcd for $C_9H_{12}N_2O_4Na$: 262.1055, found, 262.1147.

Ethyl 3-(3-ethylisoxazol-5-yl)-2-(hydroxyimino)propanoate (45b): This compound was obtained when following the general protocol for the transformation of compounds **3** into **2** as a solid (2.85 g, 58%). ^{1}H NMR ($CDCl_3$): 10.22 (s(br), 1H), 5.94 (s, 1H), 4.34 (q, 2H, J = 7.2 Hz), 4.10 (s, 2H), 2.65 (q, 2H, J = 7.6 Hz), 1.35 (t, 3H, J = 7.2 Hz), 1.24 (t, 3H, J = 7.6 Hz).

^{13}C NMR ($CDCl_3$): 165.2, 165.4, 162.6, 146.8, 101.7, 60.3, 22.5, 19.5, 14.0, 12.5. HRMS (*m/z*): $[M+Na]^+$ calcd for $C_{10}H_{14}N_2O_4Na$: 249.0851, found, 249.0854.

Ethyl 2-amino-3-(3-methylisoxazol-5-yl)propanoate (46a): By using the general protocol described above for the reduction of compounds **2** into **1**, this compound was obtained as an oil (0.43 g, 35%). ^1H NMR ($CDCl_3$): 5.96 (s, 1H), 4.22 (qd, 2H, *J* = 7.2, 1.9 Hz), 3.84 (dd, 1H, *J* = 7.7, 5.0 Hz), 3.20 (dd, 1H, *J* = 15.0, 5.0 Hz), 3.05 (dd, 1H, *J* = 15.1, 7.7 Hz), 2.29 (s, 3H), 1.30 (t, 3H, *J* = 7.1 Hz). ^{13}C NMR ($CDCl_3$): 173.9, 168.9, 159.9, 103.5, 61.4, 53.1, 32.1, 14.2, 11.4. HRMS (*m/z*): $[M+H]^+$ calcd for $C_9H_{15}N_2O_3$: 199.1083, found, 199.1085.

Ethyl 2-amino-3-(3-ethylisoxazol-5-yl)propanoate (46b): By using the general protocol described above for the reduction of compounds **2** into **1**, this compound was obtained as an oil (2.26 g, 84%).^1H NMR ($CDCl_3$): 5.98 (s, 1H), 4.22 (qd, 2H, *J* = 7.1, 2.3 Hz), 3.84 (dd, 1H, *J* = 7.7, 5.1 Hz), 3.21 (ddd, 1H, *J* = 15.0, 5.1, 0.7 Hz), 3.06 (ddd, 1H, *J* = 15.1, 7.7, 0.7 Hz), 2.68 (q, 2H, *J* = 7.6 Hz), 1.29 (t, 4H, *J* = 7.2 Hz), 1.27 (t, 2H, *J* = 7.6 Hz). ^{13}C NMR ($CDCl_3$): 173.9, 168.7, 165.2, 102.1, 61.4, 53.1, 32.2, 19.6, 14.2, 12.6. HRMS (*m/z*): $[M+H]^+$ calcd for $C_{10}H_{17}N_2O_3$: 213.1239, found, 213.1237.

Ethyl 3-bromo-2-(hydroxyimino)propanoate (47): Two preparations were used to synthesize compound **47**. The first one is very much the protocol described in a patent [12]: 75% pure ethyl bromopyruvate (5 mL, 0.0299 mol) was dissolved in water (10 mL) and chloroform (10 mL). To this was added hydroxylamine hydrochloride (2.52 g, 0.036 mol) and this was stirred in a closed vessel overnight. The resulting solution was diluted in dichloromethane, the organic layer was washed with water, brine, dried over magnesium sulfate, concentrated to dryness and recrystallized from *n*-heptane to yield compound **47** (still containing traces of ethyl 2-(hydroxyimino)propanoate; δ = 2.12 ppm) as white needles (5.43 g, 64%). Note: an improved yield (19.74 g, 77%) was obtained when following the same procedure but using 90% pure ethyl bromopyruvate. Alternatively, we used the following original protocol (very much inspired by a report [13] mentioning a one pot synthesis of ethyl 2-(hydroxyimino)acetate from glyoxylic acid): 3-bromo-2-oxopropanoic acid (5.85 g, 0.0316

mol), hydroxylamine hydrochloride (2.2 g, 0.0316 mol) and sodium bromide (6.5 g, 0.063 mol) were stirred overnight at 20 °C in dry ethanol (60 mL) and then heated to reflux for 30 minutes. This was dispersed in water and ethyl acetate, the organic layer was washed with water, brine, concentrated to dryness and the residue recrystallized from *n*-heptane to yield compound **47** (4.67 g, 70%) still containing some amount of its (oily) chlorinated homolog. ^{1}H NMR data similar to the reported one [12].

3-Ethyl-2-methylfuran (48ao): In a 500 mL flask fitted with a condenser and a dropping funnel, lithium aluminium hydride (0.91 g, 0.024 mol) was weighted. Under an argon atmosphere, dry ether (20 mL) was added and the suspension stirred. In another flask, dry aluminium trichloride (3.2 g, 0.024 mol) was dissolved in dry ether (25 mL); note: a slight heating can be observed. This solution was then added to the suspension described above (no or little heating occurred). To this suspension 1-(2-methylfuran-3-yl)ethan-1-one, prepared from acetylacetone and chloroacetaldehyde as described below for the preparation of 6,7-dihydrobenzofuran-4(5*H*)-one (2.97 g, 0.024 mol) dissolved in dry ether (50 mL) was added drop-wise, slowly enough to avoid too much reflux. The resulting solution was stirred for 2 hours, cautiously quenched with water, and cautiously diluted with a 3 N solution of sulfuric acid. This was diluted in water and ether, the organic layer was washed with water, brine, dried over magnesium sulfate and concentrated at atmospheric pressure to yield an oil containing compound **48ao** and some ether which was directly used for the cycloaddition reaction as described below.

4,5,6,7-Tetrahydrobenzofuran (48ap): Step 1, preparation of 6,7-dihydrobenzofuran-4(5*H*)-one [14]: under argon, cyclohexanedione (20.71 g, 0.18 mol) was dissolved in methanol (100 mL) and cooled to 0 °C. To this was added potassium hydroxide (12.2 g, 0.18 mol) and after stirring 30 minutes at 0 °C the 50% solution of chloroacetaldehyde in water (26.3 mL, 0.203 mol) was added. This was stirred overnight, the resulting solution made acidic with 1 N hydrochloric acid, extracted with ethyl acetate, the organic layer was washed with water, brine, dried over magnesium sulfate and concentrated to dryness to give the volatile 6,7-

dihydrobenzofuran-4(5*H*)-one (16.5 g) clean enough for the next step. Step 2, reduction with $AlLiH_4/AlCl_3$: from 6,7-dihydrobenzofuran-4(5*H*)-one (3.62 g, 0.026 mol) the procedure is identical to the one described above for the preparation of **48ao**. Note: but for the purification, this reduction protocol is pretty much identical to the one reported previously [15]. Again the oil containing **48ap** and some ether was used directly for the cycloaddition reaction as described below.

2-Ethyl-3-methylfuran (48aq): Step 1: preparation of ethyl 2-ethylfuran-3-carboxylate. By using the protocol described above for the synthesis of 6,7-dihydrobenzofuran-4(5H)-one, this volatile compound was obtained from ethyl 3-oxopentanoate (9.88 g, 0.068 mol) and chloroacetaldehyde after a chromatography over silica gel (cyclohexane – dichloromethane) as an oil still containing some cyclohexane (5.21 g). Step 2, reduction with $AlLiH_4/AlCl_3$: the procedure is identical to the one described above for the preparation of 3-ethyl-2-methylfuran (**48ao**) but for the differences that two equivalent of $AlCl_3$ and two equivalent of $AlLiH_4$ were used and that the reaction was left to stir for 50 hours before undertaking the work up procedure described above. This gave the volatile compound **48aq** as an oil still containing ether and cyclohexane which was used directly for the cycloaddition reaction as described below.

2-isopropyl-3-methylfuran (48ar): Step 1: preparation of ethyl 2-isopropylfuran-3-carboxylate. By using the protocol described above for the synthesis of 6,7-dihydrobenzofuran-4(5*H*)-one, this volatile compound was obtained from ethyl 4-methyl-3-oxopentanoate (6.92 g, 0.0437 mol) and chloroacetaldehyde after a chromatography over silica gel (cyclohexane – dichloromethane) as an oil still containing some cyclohexane (1.8 g). Step 2, reduction with $AlLiH_4/AlCl_3$: the procedure is identical to the one described above for the preparation of 3-ethyl-2-methylfuran (**48ao**) but for the differences that 2 equivalent of $AlCl_3$ and two equivalent of $AlLiH_4$ were used and that the reaction was left to stir for 50 hours before undertaking the work up procedure described above. This gave the volatile

compound **48ar** as an oil still containing ether and cyclohexane which was used directly for the cycloaddition reaction as described below.

Protocols for the synthesis of furan-bearing α-hydroxyimino esters by [2 + 4] cycloaddition Protocol b: The considered furan (0.013 mol), compound **47** (2.75 g, 0.013 mol) and sodium carbonate (1.5 g, 0.0144 mol) were dispersed in toluene (15 mL) before adding tetrabutylammonium bromide (0.042 g, 13 mmol). This was stirred for 1 hour, diluted in water and ethyl acetate, the organic layer was washed with water, brine, dried over magnesium sulfate, concentrated to dryness and further purified as described below. Protocol c: The considered furan (0.01 mol), compound **47** (2.10 g, 0.01 mol) and ammonium bicarbonate (0.86 g, 0.011 mol) were dispersed in ethyl acetate (10 mL) before adding water (2 mL). This was stirred for 1 hour, diluted in water and ethyl acetate, the organic layer was washed with water, brine, dried over magnesium sulfate, concentrated to dryness and further purified as described below. Protocol d: The considered furan (6.09 mmol), compound **47** (1.28 g, 6.09 mmol) and lithium carbonate (0.49 g, 6.69 mmol) were dispersed in ethyl acetate (10 mL) before adding water (2 mL). This was stirred for 1 hour, diluted in water and ethyl acetate made, the organic layer was washed with water, brine, dried over magnesium sulfate, concentrated to dryness and the residue further purified as described below. Protocol e: The considered furan (0.0117 mol), compound **47** (2.56 g, 0.0117 mol) and sodium carbonate (1.42 g, 0.0129 mol) were dispersed in ethyl acetate (6 mL) and water (3 mL). This was stirred for 1 hour, diluted in water and ethyl acetate, the organic layer was washed with water, brine, dried over magnesium sulfate, concentrated to dryness and further purified as described below.

Ethyl 2-(hydroxyimino)-3-(5-propylfuran-2-yl)propanoate (2am): This compound was obtained in the yield reported in Table 2, as a solid after a chromatography over silica gel (cyclohexane - ethyl acetate 5/1). ^{1}H NMR ($CDCl_3$): 9.15 (s(br), 1H), 5.98 (d, 1H, *J* = 3.0 Hz), 5.88 (m, 1H), 4.32 (q, 2H, *J* = 7.0 Hz), 3.99 (s, 2H), 2.55 (q, 2H, *J* = 7.7 Hz), 1.65 (m, 2H), 1.35 (t, 3H, *J* = 7.1 Hz), 0.95 (t, 3H, *J* = 7.5 Hz). ^{13}C NMR ($CDCl_3$): 163.0, 155.5, 148.8,

146.8, 107.3, 105.5, 61.9, 30.0, 23.7, 21.3, 14.0, 13.7. HRMS (*m/z*): $[M+Na]^+$ calcd for $C_{12}H_{17}NO_4Na$: 262.1055, found, 262.1047.

Ethyl 2-(hydroxyimino)-3-(5-pentylfuran-2-yl)propanoate (2an): This compound was obtained in the yield reported in Table 2, as a solid after a chromatography over silica gel (cyclohexane - ethyl acetate 5/1). ^{1}H NMR ($CDCl_3$): 8.33 (s(br), 1H), 5.98 (d, 1H, *J* = 3.0 Hz), 5.87 (m, 1H), 4.32 (q, 2H, *J* = 7.0 Hz), 3.99 (s, 2H), 2.57 (q, 2H, *J* = 7.7 Hz), 1.65 (m, 2H), 1.35 (t, 3H, *J* = 7.1 Hz), 1.32 (m, 4H), 0.91 (t, 3H, *J* = 7.5 Hz). ^{13}C NMR ($CDCl_3$): 162.9, 155.7, 148.7, 146.5, 107.3, 105.3, 61.9, 31.3, 30.0, 27.6, 23.7, 22.3, 14.0, 13.9. HRMS (*m/z*): $[M+Na]^+$ calcd for $C_{14}H_{121}NO_4Na$: 290.1368, found, 290.1366.

Ethyl 3-(4-ethyl-5-methylfuran-2-yl)-2-(hydroxyimino)propanoate (2ao): This compound was obtained in the yield reported in Table 2, as a solid after a chromatography over silica gel (cyclohexane - ethyl acetate 5/1). ^{1}H NMR ($CDCl_3$): 5.93 (s, 1H), 4.33 (q, 2H, *J* = 7.2 Hz), 3.95 (s, 2H), 2.29 (q, 2H, *J* = 7.4 Hz), 2.16 (s, 3H), 1.35 (t, 3H *J* = 7.2 Hz), 1.09 (t, 3H, *J* = 7.4 Hz). ^{13}C NMR ($CDCl_3$): 163.0, 148.9, 145.5, 145.4, 121.4, 108.5, 61.9, 23.6, 18.0, 14.7, 14.0, 11.3. HRMS (*m/z*): $[M+Na]^+$ calcd for $C_{12}H_{17}NO_4Na$: 262.1055, found, 262.1053.

Ethyl 2-(hydroxyimino)-3-(4,5,6,7-tetrahydrobenzofuran-2-yl)propanoate (2ap): This compound was obtained in the yield reported in Table 2, as a solid after a chromatography over silica gel (cyclohexane - ethyl acetate 5/1). ^{1}H NMR ($CDCl_3$): 5.91 (s, 1H), 4.33 (q, 2H, *J* = 7.2 Hz), 3.97 (s, 2H), 2.54 (m, 2H), 2.36 (m, 2H), 1.80 (m, 2H), 1.70 (m, 2H), 1.35 (t, 3H, *J* = 7.2 Hz). ^{13}C NMR ($CDCl_3$): 163.0, 149.7, 149.0, 146.0, 117.6, 107.8, 61.9, 23.7, 23.2, 23.1, 23.0, 22.0, 14.0. HRMS (*m/z*): $[M+Na]^+$ calcd for $C_{13}H_{17}NO_4Na$: 274.1054, found, 274.1057.

Ethyl-3-(5-ethyl-4-methylfuran-2-yl)-2-(hydroxyimino)propanoate (2aq): This compound was obtained in the yield reported in Table 2, as a solid after a chromatography over silica gel (cyclohexane - ethyl acetate 5/1). ^{1}H NMR ($CDCl_3$): 8.56 (s(br), 1H), 5.86 (s, 1H), 4.33 (q, 2H, *J* = 7.2 Hz), 3.95 (s, 2H), 2.54 (q, 2H, *J* = 7.6 Hz), 1.89 (s, 3H), 1.35 (t, 3H *J* = 7.2 Hz), 1.16 (t, 3H, *J* = 7.6 Hz). ^{13}C NMR ($CDCl_3$): 163.0, 151.5, 148.9, 145.3, 113.7, 110.0, 61.9,

23.6, 19.3, 14.0, 12.9, 9.7. HRMS (*m/z*): $[M+Na]^+$ calcd for $C_{12}H_{17}NO_4Na$: 262.1055, found, 262.1053.

Ethyl 2-(hydroxyimino)-3-(5-isopropyl-4-methylfuran-2-yl)propanoate (2ar): This compound (90% pure) was obtained in the yield reported in Table 2, as a solid after a chromatography over silica gel (cyclohexane - ethyl acetate 5/1). ^{1}H NMR ($CDCl_3$): 5.83 (s, 1H), 4.32 (q, 2H, *J* = 7.2 Hz), 3.95 (s, 2H), 2.94 (sept, 1H), *J* = 7.0 Hz), 1.91 (s, 3H), 1.35 (t, 3H, *J* = 7.2 Hz), 1.20 (d, 6H, *J* = 7.0 Hz). ^{13}C NMR ($CDCl_3$): 163.0, 154.5, 149.0, 145.0, 112.6, 110.0, 61.9, 26.2, 23.6, 21.3, 14.0, 9.7. HRMS (*m/z*): $[M+Na]^+$ calcd for $C_{13}H_{19}NO_4Na$: 276.1212, found, 276.1213.

Ethyl 2-amino-3-(5-propylfuran-2-yl)propanoate (1am): By using the general protocol described above for the reduction of compounds **2** into **1**, this compound was obtained as an oil (0.93 g, 79%). ^{1}H NMR ($CDCl_3$): 6.00 (d, 1H, *J* = 3.0 Hz), 5.88 (dt, 1H, *J* = 3.0, 1.0 Hz), 4.20 (qd, 2H, *J* = 7.1, 2.6 Hz), 3.75 (dd, 1H, *J* = 7.1, 5.1 Hz), 3.05 (dd, 1H, *J* = 14.9, 5.1 Hz), 2.95 (dd, 1H, *J* = 14.9, 7.1 Hz), 2.55 (t, 3H, *J* = 7.4 Hz), 1.65 (m, 2H), 1.29 (t, 3H, *J* = 7.1 Hz), 0.96 (t, 3H, *J* = 7.4 Hz). ^{13}C NMR ($CDCl_3$): 174.7, 155.7, 149.3, 108.1, 105.3, 60.9, 53.8, 33.7, 30.0, 21.3, 14.2, 13.7. HRMS (*m/z*): $[M+H]^+$ calcd for $C_{12}H_{20}NO_3$: 226.1443, found, 226.1449.

Ethyl 2-amino-3-(5-pentylfuran-2-yl)propanoate (1an): By using the general protocol described above for the reduction of compounds **2** into **1**, this compound was obtained as an oil (0.54 g, 90%). ^{1}H NMR ($CDCl_3$): 6.00 (d, 1H, *J* = 3.0 Hz), 5.88 (m, 1H), 4.20 (qd, 2H, *J* = 7.1, 2.6 Hz), 3.75 (dd, 1H, *J* = 7.1, 5.1 Hz), 3.07 (dd, 1H, *J* = 14.9, 5.1 Hz), 2.96 (dd, 1H, *J* = 14.9, 7.1 Hz), 2.56 (t, 3H, *J* = 7.4 Hz), 19.6 (s(br), 2H), 1.61 (m, 2H), 1.33 (m, 4H), 1.29 (t, 3H, *J* = 7.1 Hz), 0.91 (t, 3H, *J* = 7.4 Hz). ^{13}C NMR ($CDCl_3$): 174.5, 156.0, 149.1, 108.2, 105.2, 61.0, 53.7, 33.5, 31.3, 28.0, 27.6, 22.4, 14.2, 13.9. HRMS (*m/z*): $[M+H]^+$ calcd for $C_{14}H_{24}NO_3$: 254.1756, found, 254.1756.

Ethyl 2-amino-3-(4-ethyl-5-methylfuran-2-yl)propanoate (1ao): By using the general protocol described above for the reduction of compounds **2** into **1**, this compound was

obtained as an oil (0.95 g, 82%). ^{1}H NMR ($CDCl_3$): 5.94 (s, 1H), 4.20 (m, 2H), 3.73 (dd, 1H, J = 4.9 and 7.4 Hz), 3.02 (dd, 1H, J = 4.9 and 14.7 Hz), 2.90 (dd, 1H, J = 7.4 and 14.7 Hz), 2.29 (q, 2H, J = 7.5), 2.16 (s, 3H), 1.83 (s (br), 2H), 1.27 (d, 3H J = 7.1 Hz), 1.05 (d, 3H J = 7.5 Hz). ^{13}C NMR ($CDCl_3$): 174.7, 148.2, 145.7, 121.2, 109.2, 60.9, 53.8, 33.6, 18.0, 14.8, 14.2, 11.3. HRMS (*m/z*): $[M+H]^+$ calcd for $C_{12}H_{20}NO_3$: 226.1443, found, 226.1446.

Ethyl 2-amino-3-(4,5,6,7-tetrahydrobenzofuran-2-yl)propanoate (1ap): By using the general protocol described above for the reduction of compounds **2** into **1**, this compound was obtained as an oil (1.57 g, 82%). ^{1}H NMR ($CDCl_3$): 5.91 (s, 1H), 4.20 (m, 2H), 3.74 (dd, 1H, J = 5.0 and 7.4 Hz), 3.05 (dd, 1H, J = 5.0 and 14.8 Hz), 2.90 (dd, 1H, J = 7.4 and 14.8 Hz), 2.54 (m, 2H), 2.37 (m, 2H), 1.87 (s (br), 2H), 1.81 (m, 2H), 1.70 (m, 2H), 1.29 (d, 3H J = 7.2 Hz). ^{13}C NMR ($CDCl_3$): 174.7, 150.0, 148.7, 117.4, 108.6, 61.1, 53.8, 33.7, 21.1 (two signals), 23.1, 22.0, 14.1. HRMS (*m/z*): $[M+H]^+$ calcd for $C_{13}H_{20}NO_3$: 238.1443, found, 238.1438.

Ethyl 2-amino-3-(5-ethyl-4-methylfuran-2-yl)propanoate (1aq): By using the general protocol described above for the reduction of compounds **2** into **1**, this compound was obtained as an oil (0.95 g, 82%). ^{1}H NMR ($CDCl_3$): 5.94 (s, 1H), 4.20 (m, 2H), 3.73 (dd, 1H, J = 4.9 and 7.4 Hz), 3.02 (dd, 1H, J = 4.9 and 14.7 Hz), 2.90 (dd, 1H, J = 7.4 and 14.7 Hz), 2.29 (q, 2H, J = 7.5), 2.16 (s, 3H), 1.83 (s (br), 2H), 1.27 (d, 3H J = 7.1 Hz), 1.05 (d, 3H J = 7.5 Hz). ^{13}C NMR ($CDCl_3$): 174.7, 148.2, 145.7, 121.2, 109.2, 60.9, 53.8, 33.6, 18.0, 14.8, 14.2, 11.3.

Ethyl 2-amino-3-(5-isopropyl-4-methylfuran-2-yl)propanoate (1ar): By using the general protocol described above for the reduction of compounds **2** into **1**, this compound was obtained as an oil (0.41 g, 86%; 90% pure). ^{1}H NMR ($CDCl_3$): 5.87 (s, 1H), 4.21 (m, 2H), 3.73 (dd, 1H, J = 5.1 and 6.8 Hz), 3.01 (dd, 1H, J = 5.1 and 14.8 Hz), 2.94 (sept, 1H, J = 7.0 Hz), 2.90 (dd, 1H, J = 6.8 and 14.8 Hz), 1.91 (s, 3H), 1.84 (s, 2H), 1.29 (q, 2H, J = 7.1), 1.20 (d, 6H J = 7.0 Hz).^{13}C NMR ($CDCl_3$): 174.6, 154.8, 147.6, 112.3, 111.1, 60.9, 53.6, 33.4,

26.1, 21.3, 21.2, 14.1, 9.7. HRMS (*m/z*): $[M+H]^+$ calcd for $C_{13}H_{22}NO_3$: 240.1600, found, 240.1602.

Ethyl 2-amino-3-(1,3-dioxolan-2-yl)propanoate (53): Step 1: the previously described [16] diethyl 2-((1,3-dioxolan-2-yl)methyl)malonate (**51**) (7.8 g, 0.317 mol) was dissolved in ethanol (40 mL) and potassium hydroxide (1.78 g, 0.0317 mol) dissolved in ethanol (60 mL) was added dropwise. The solution was stirred overnight at room temperature and then briefly heated to reflux. This was concentrated, dissolved in water which was made more basic with 22% ammonia. The aqueous phase was washed with dichloromethane, made acidic with 37% hydrochloric acid, extracted with ethyl acetate, the organic layer was washed with water, brine, dried over magnesium sulfate and concentrated to dryness to yield 90% pure 2-((1,3-dioxolan-2-yl)methyl)-3-ethoxy-3-oxopropanoic acid (**52**) (3.85 g, 55%). ^{1}H NMR ($CDCl_3$): 5.05 (t, 1H, *J* = 3.7 Hz), 4.24 (q, 2H, *J* = 7.1 Hz), 3.98 (m, 2H), 3.82 (m, 2H), 3.64 (t, 1H, *J* = 6.9 Hz), 2.40 (m, 2H), 1.30 (t, 3H, *J* = 7.1 Hz). Step 2: Under argon, this compound (3.85 g, 0.0176 mol) was dissolved in toluene (50 mL, dried over 4 Å molecular). Triethylamine (2.95 mL, 0.021 mol) was added and then diphenylphosphoryl azide (4.18 mL, 0.021 mol). This was stirred at 80 °C for 1.5 h, the toluene was removed under vacuum and the resulting oil repeatedly extracted with cyclohexane. Concentration to dryness of this extract led to an oil which was stirred at room temperature in 4% hydrochloric acid (50 mL) overnight. The resulting solution was diluted in more acid, washed with ethyl acetate, the aqueous phase was made basic with 22% ammonia, extracted with ethyl acetate, and this organic layer was washed with water, brine, dried over sodium carbonate and concentrated to dryness to yield the α-amino ester **53** as an oil (0.14 g, 16%). Alternatively, the same compound (0.44 g, 43%) was obtained from **53**, using the very mild reduction protocol described below for the preparation of α-amino ester **56**. ^{1}H NMR ($CDCl_3$): 5.03 (dd, 1H, *J* = 4.3, 5.0 Hz), 4.17 (q, 2H, *J* = 7.0 Hz), 3.96 (m, 2H), 3.85 (m, 2H), 3.66 (dd, 1H, *J* = 4.5, 8.3 Hz), 2.14 (ddd, 1H, *J* = 4.5, 5.0, 14.4 Hz), 1.93 (ddd, 1H, *J* = 4.3, 8.3, 14.4 Hz), 1.86 (s, 2H),

1.26 (t, 3H, J = 7.0 Hz). ^{13}C NMR ($CDCl_3$): 175.3, 102.6, 64.9, 64.8, 61.0, 51.2, 38.3, 14.2. HRMS (m/z): $[M+H]^+$ calcd for $C_8H_{16}NO_4$, 190.1079, found, 190.1061.

Ethyl 3-(1,3-dioxolan-2-yl)-2-(hydroxyimino)propanoate (54): This compound was obtained from compound **51** [16] as a solid (3.81 g, 28% from diethyl malonate) using the general preparation of oximes **2** from malonates described above after a chromatography over silica gel (cyclohexane – ethyl acetate 3/2) and a recrystallisation from cyclohexane (two crops). ^{1}H NMR (DMSO-d_6, not stable in $CDCl_3$): 12.34 (s (br), 1H), 5.11 (t, 1H, J = 5.5 Hz), 4.19 (q, 2H, J = 7.1 Hz), 3.87 (m, 2H), 3.75 (m, 2H), 2.83 (d, 2H, J = 5.2 Hz), 1.24 (t, 3H, J = 7.1 Hz). ^{13}C NMR (DMSO-d_6): 164.1, 147.3, 100.5, 64.5, 61.3, 30.1, 14.4. HRMS (m/z): $[M+Na]^+$ calcd for $C_8H_{13}NO_5Na$, 226.0691, found, 226.0698.

Diethyl 2-(2-(1,3-dioxolan-2-yl)ethyl)malonate (56): The following protocol is inspired from the reported procedure [17]. Under a calcium chloride-protected atmosphere, diethylmalonate (13.77 g, 0.0859 mol) was dissolved in dry DMF (110 mL, dried over 4 Å molecular sieves), This was cooled to 0 °C and 60% sodium hydride suspended in oil (3.33 g, 0.0832 mol) was added by portion. At the end of the hydrogen evolution, 2-(2-bromoethyl)-1,3-dioxolane (9.8 mL, 0.0834 mol) was added. The reaction wessel was brought back to room temperature and then heated at 80 °C for 4 hours. The resulting suspension was concentrated, the residue dissolved in a mixture of water and diethyether, the organic layer was washed with water four times, brine, dried over magnesium sulfate and concentrated to dryness. A distillation under a 5 mbar vaccum led to a fraction boiling at 148-150 °C which contained pure compound **56** (13.28 g, 59 %). ^{1}H NMR (DMSO-d_6) : 4.79 (t, 1H, J = 4.8 Hz), 4.12 (m, 2H), 3.87 (m, 2H), 3.76 (m, 2H), 3.51 (t, 1H, J = 7.6 Hz), 1.87 (m, 2H), 1.58 (m, 2H), 1.18 (t, 3H, J = 7.2 Hz).^{13}C NMR (DMSO-d_6): 169.3, 103.4, 64.7, 61.3, 51.2, 31.0, 23.3, 14.4. ^{1}H NMR data identical with the one reported previously [17].

Ethyl 4-(1,3-dioxolan-2-yl)-2-(hydroxyimino)butanoate (57): This compound was obtained from compound **56** (5.46 g) as a white powder (3.27 g, 71%) using the general preparation of oximes **2** from malonates described above and a chromatography over silica gel

(dichloromethane – ethanol 98/2). ^{1}H NMR (DMSO-d_6, not stable in $CDCl_3$): 12.14 (s (br), 1H), 4.79 (t, 1H, J = 4.4 Hz), 4.18 (q, 2H, J = 7.1 Hz), 3.85 (m, 2H), 3.75 (m, 2H), 2.53 (m, 2H), 1.73 (m, 2H), 1.23 (t, 3H, J = 7.1 Hz). ^{13}C NMR (DMSO-d_6): 164.2, 151.4, 103.4, 64.8, 61.1, 29.8, 19.4, 14.5. HRMS (m/z): $[M+Na]^+$ calcd for $C_9H_{15}NO_5Na$, 240.0848, found, 240.0845.

Ethyl 2-amino-4-(1,3-dioxolan-2-yl)butanoate (58): Compound **57** (0.26 g, 1.19 mmol) and 10 % palladium over charcoal (0.12 g, 0.119 mmol) were dispersed in acetic acid (10 mL). This was charged with hydrogen (1 atm) and stirred for 24 hours. The resulting solution was filtered, concentrated to dryness, the residue was dispersed in water, made basic with 30% ammonia and this was extracted with ethyl acetate. The organic layer was washed with brine, dried over sodium carbonate and cocnetrated to dryness to give compound **58** as a volatile oil (0.16 g, 65 %). ^{1}H NMR (DMSO-d_6): 4.77 (t, 1H, J = 4.6 Hz), 4.08 (m, 2H), 3.86 (m, 2H), 3.75 (m, 2H), 3.29 (m, 1H), 1.76-1.45 (m, 6H), 1.19 (t, 3H, J = 7.06 Hz). ^{13}C NMR (DMSO-d_6): 176.1, 103.9, 64.7, 64.6, 60.4, 54.2, 30.1, 29.5, 14.6. HRMS (m/z): $[M+H]^+$ calcd for $C_9H_{18}NO_4$, 204.1236, found, 204.1235.

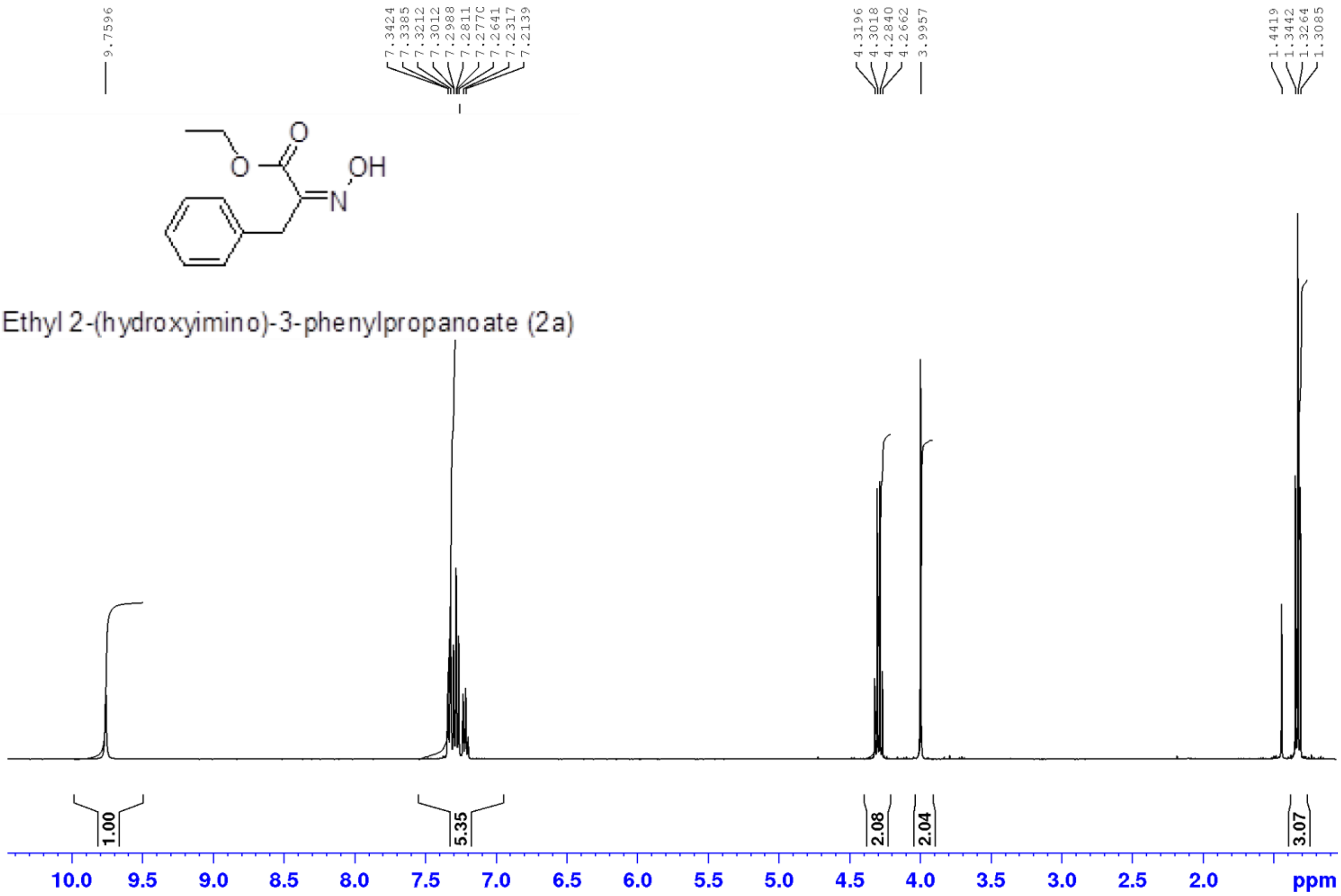

Ethyl 2-(hydroxyimino)-3-phenylpropanoate (2a)

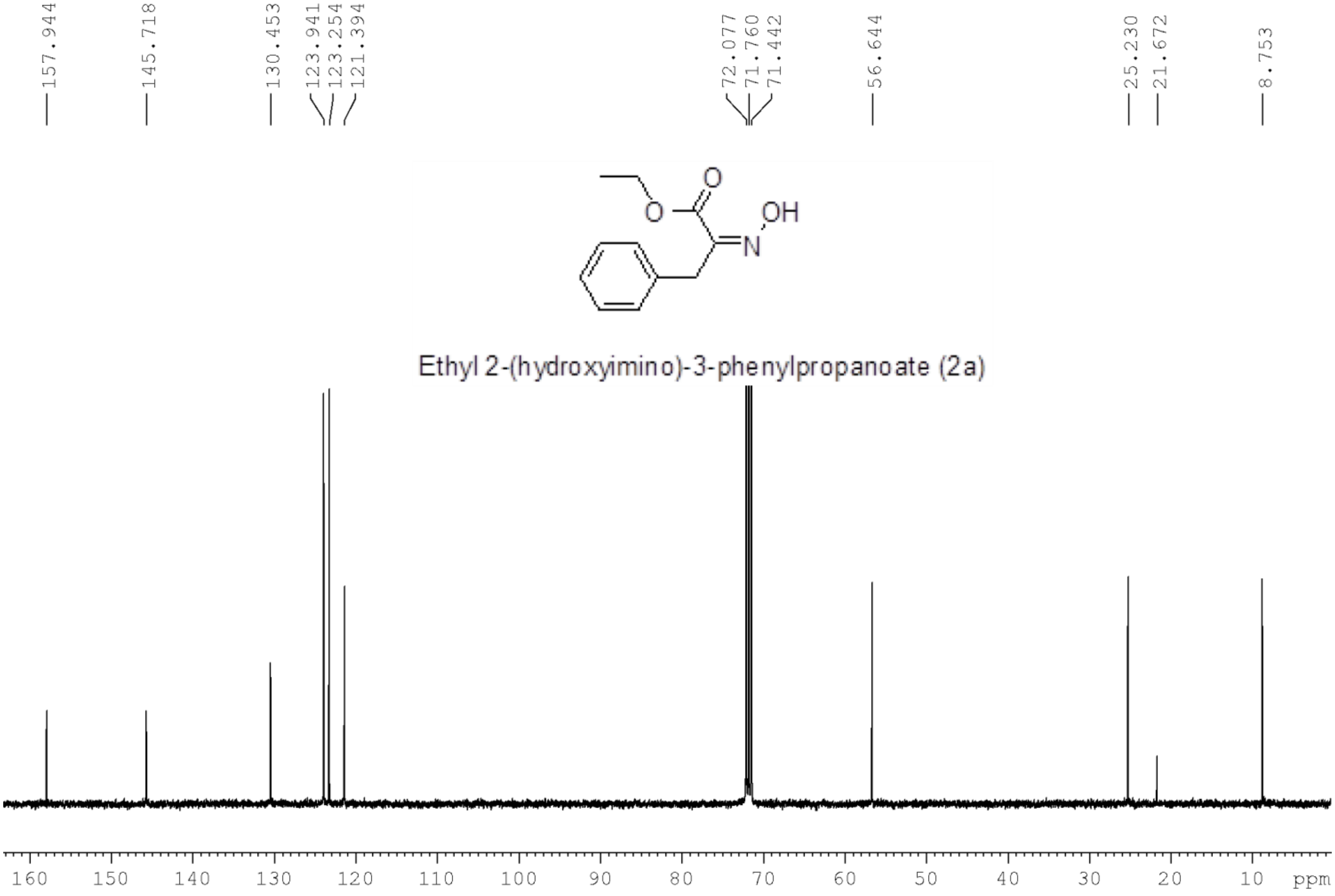

Ethyl 2-(hydroxyimino)-3-phenylpropanoate (2a)

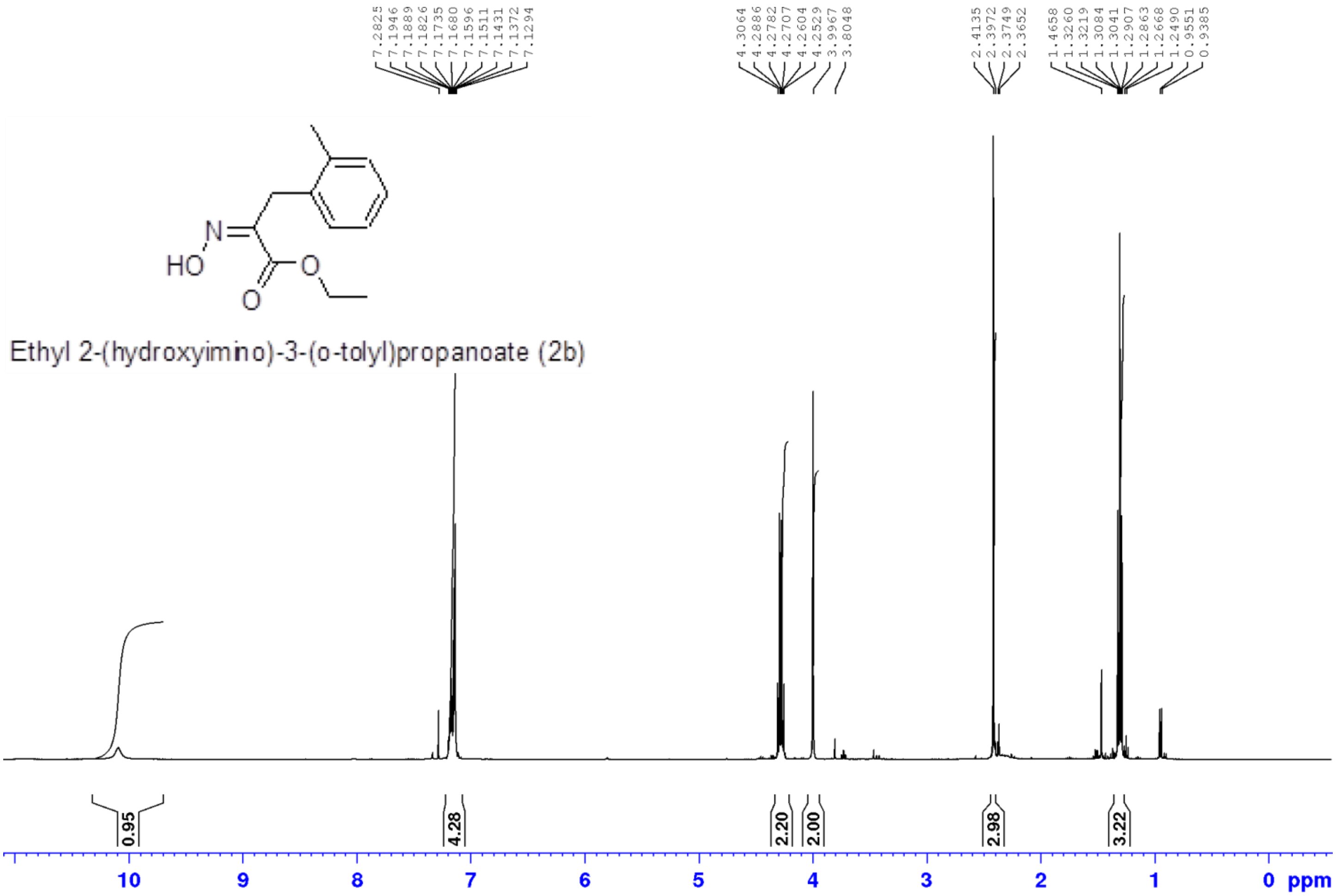
7.2825 7.1946 7.1889 7.1826 7.1735 7.1680 7.1596 7.1511 7.1431 7.1372 7.1294
4.3064 4.2886 4.2782 4.2707 4.2604 4.2529 3.9967 3.8048
2.4135 2.3972 2.3749 2.3652
1.4658 1.3260 1.3219 1.3084 1.3041 1.2907 1.2863 1.2668 1.2490 0.9551 0.9385
N
HO
O
O
Ethyl 2-(hydroxyimino)-3-(o-tolyl)propanoate (2b)
0.95
4.28
2.20
2.00
2.98
3.22
10 9 8 7 6 5 4 3 2 1 0 ppm

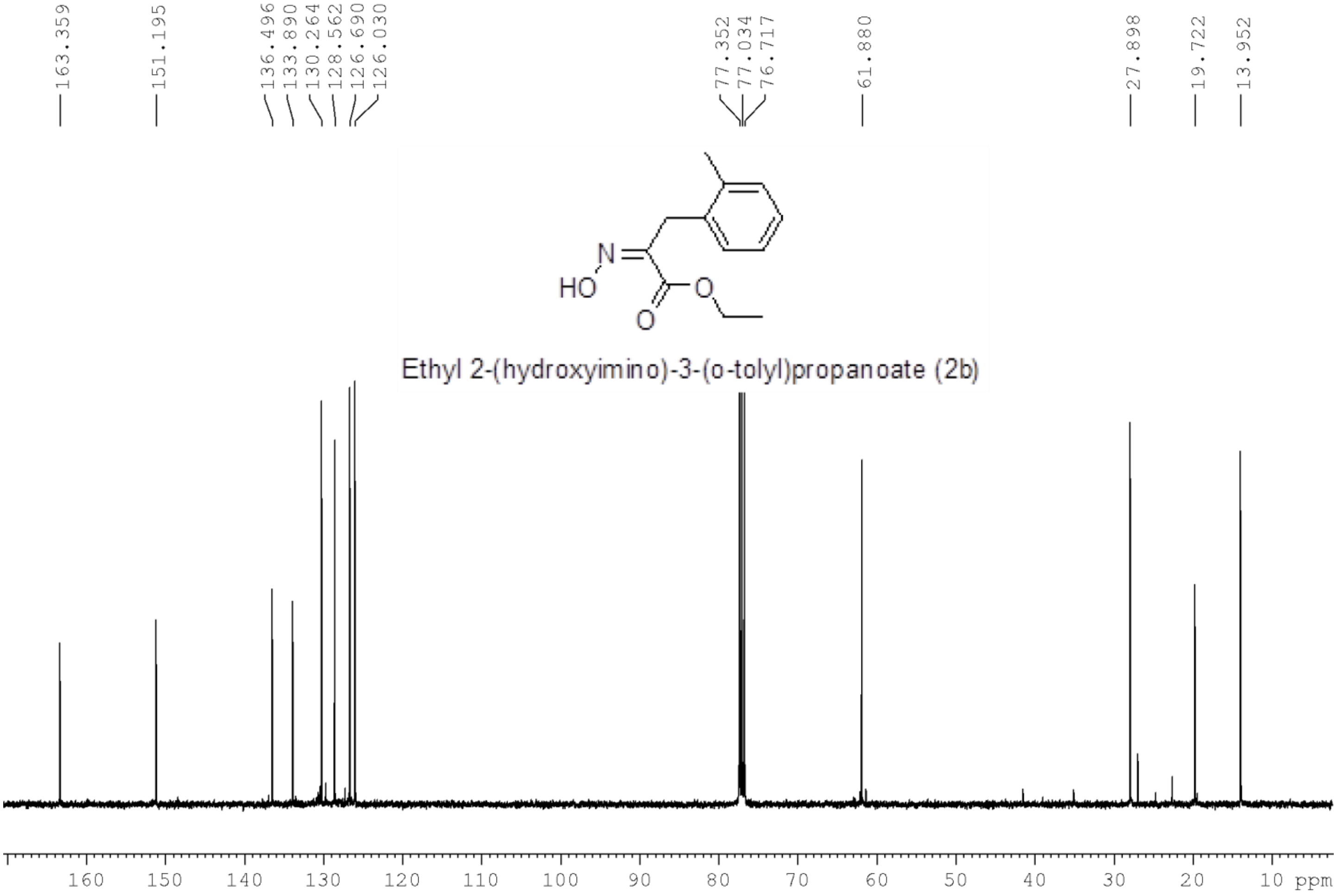
163.359
151.195
136.496 133.890 130.264 128.562 126.690 126.030
77.352 77.034 76.717
61.880
27.898
19.722
13.952
N
HO
O
O
Ethyl 2-(hydroxyimino)-3-(o-tolyl)propanoate (2b)
160 150 140 130 120 110 100 90 80 70 60 50 40 30 20 10 ppm

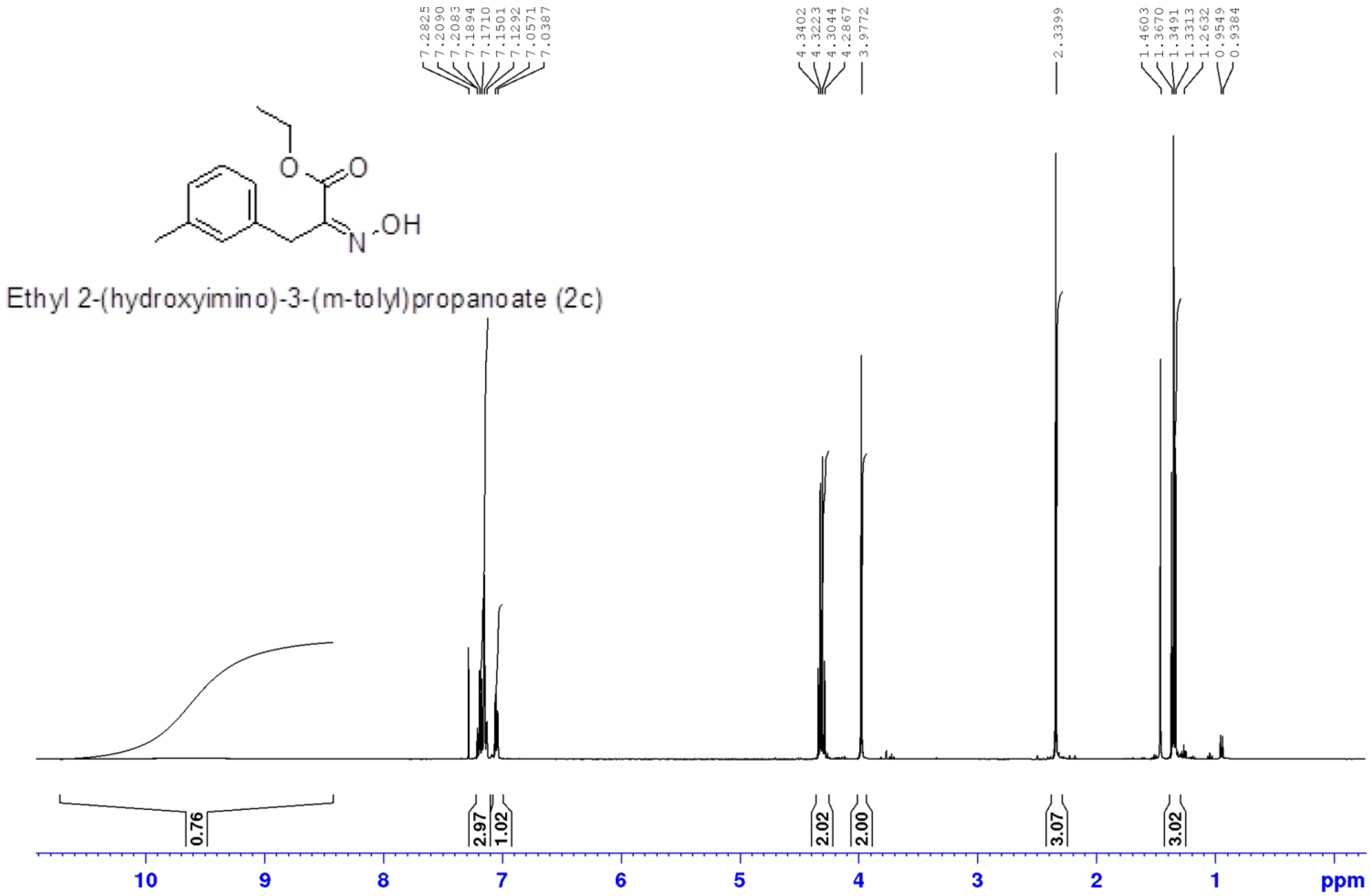
7.2825
7.2090
7.2083
7.1894
7.1710
7.1501
7.1292
7.0571
7.0387
4.3402
4.3223
4.3044
4.2867
3.9772
2.3399
1.4603
1.3670
1.3491
1.3313
1.2632
0.9549
0.9384
Ethyl 2-(hydroxyimino)-3-(m-tolyl)propanoate (2c)
0.76
2.97
1.02
2.02
2.00
3.07
3.02
10
9
8
7
6
5
4
3
2
1
ppm

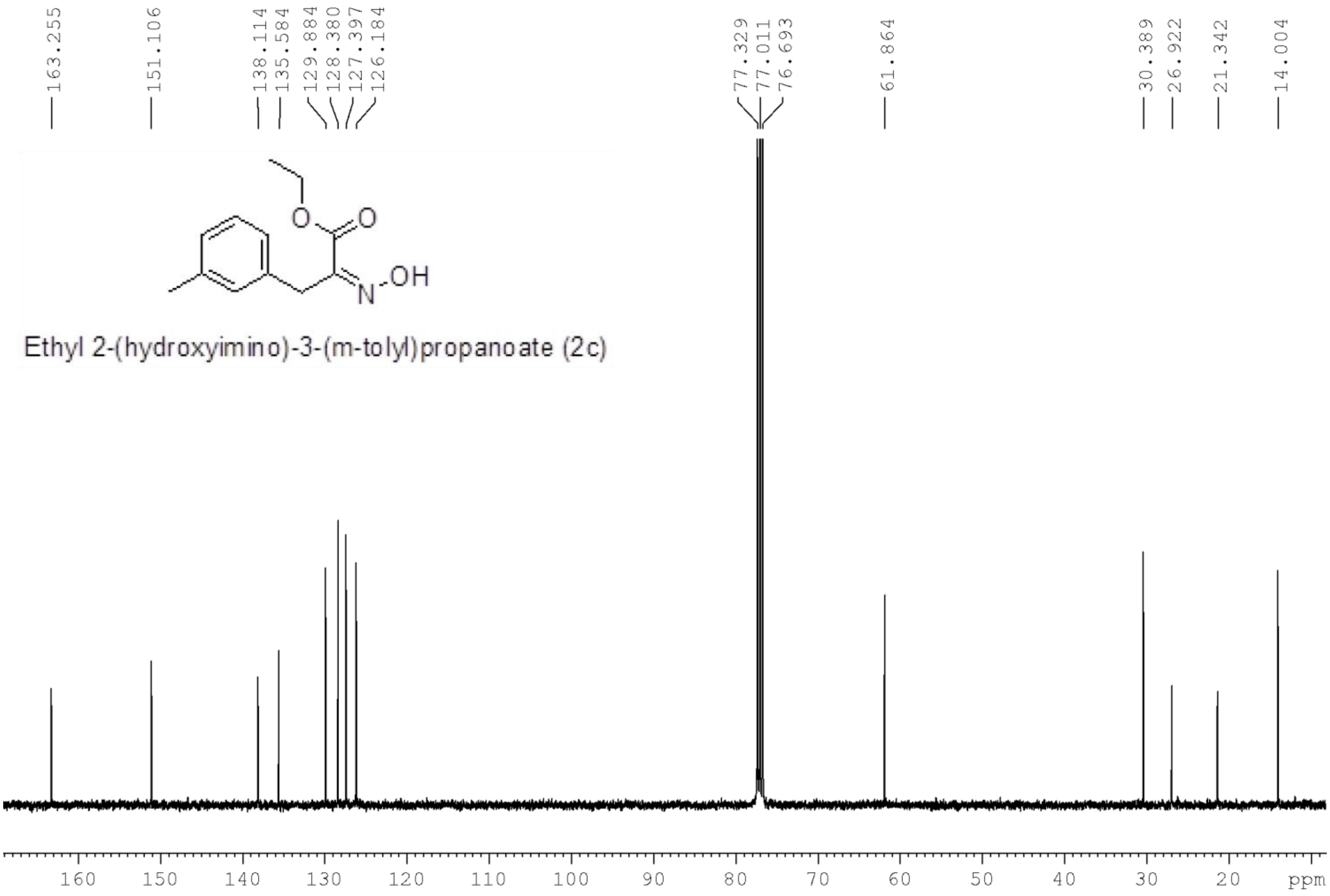
163.255
151.106
138.114
135.584
129.884
128.380
127.397
126.184
77.329
77.011
76.693
61.864
30.389
26.922
21.342
14.004
Ethyl 2-(hydroxyimino)-3-(m-tolyl)propanoate (2c)
160
150
140
130
120
110
100
90
80
70
60
50
40
30
20
ppm

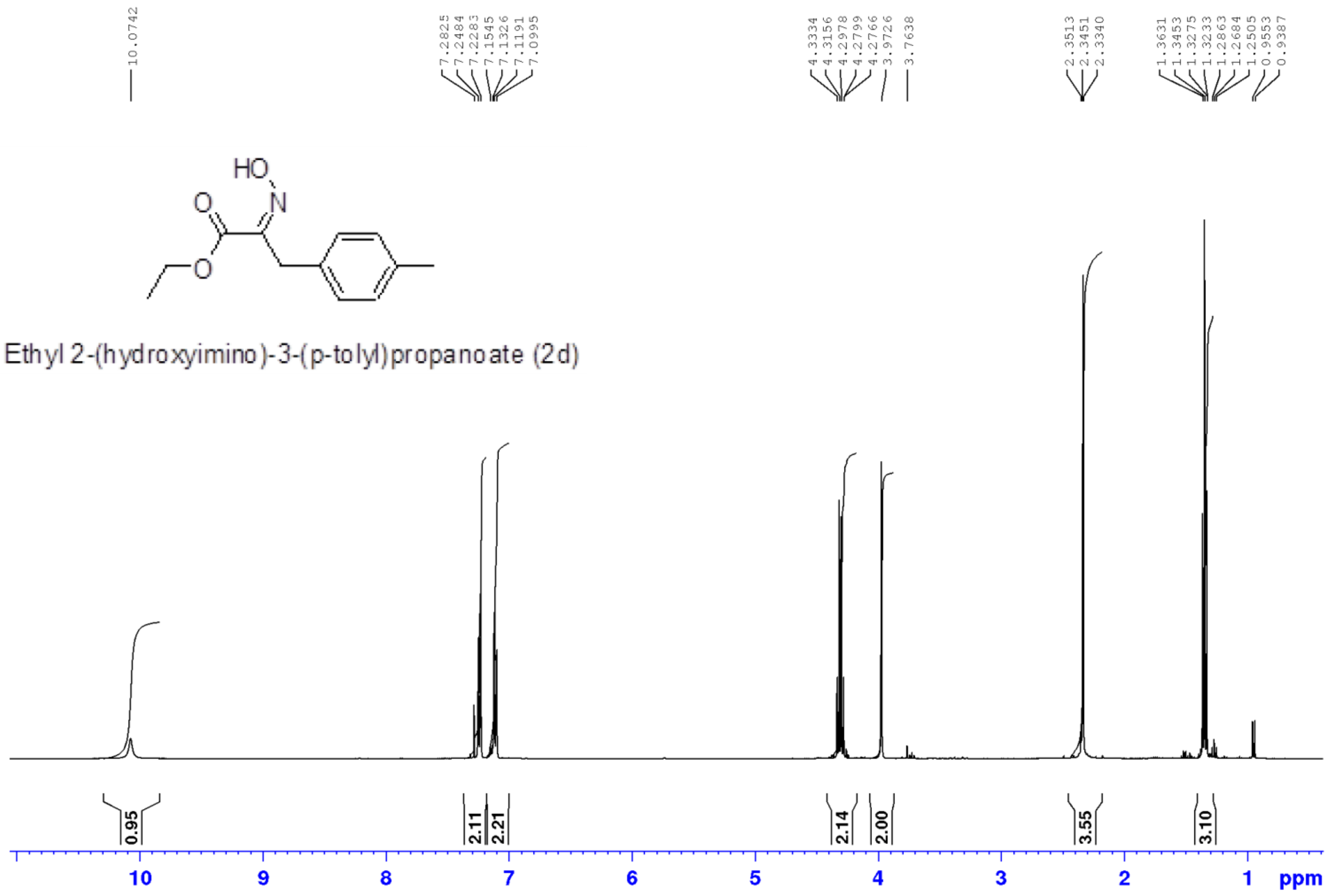
10.0742
7.2825 7.2484 7.2283 7.1545 7.1326 7.1191 7.0995
4.3334 4.3156 4.2978 4.2799 4.2766 3.9726 3.7638
2.3513 2.3451 2.3340
1.3631 1.3453 1.3275 1.3233 1.2863 1.2684 1.2505 0.9553 0.9387
HO
N
O
O
Ethyl 2-(hydroxyimino)-3-(p-tolyl)propanoate (2d)
0.95
2.11
2.21
2.14
2.00
3.55
3.10
10
9
8
7
6
5
4
3
2
1
ppm

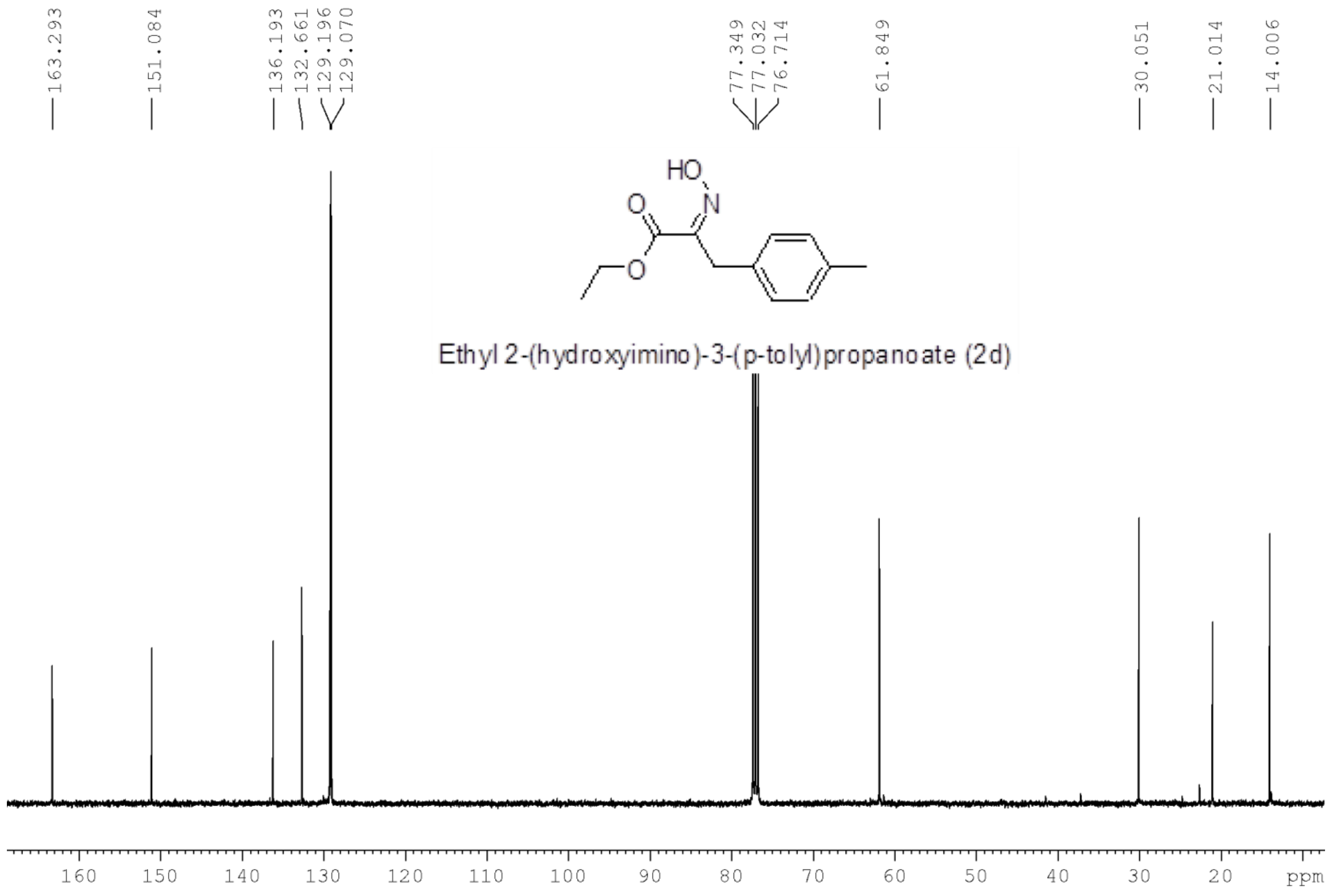
163.293
151.084
136.193 132.661 129.196 129.070
77.349 77.032 76.714
61.849
30.051
21.014
14.006
HO
N
O
O
Ethyl 2-(hydroxyimino)-3-(p-tolyl)propanoate (2d)
160
150
140
130
120
110
100
90
80
70
60
50
40
30
20
ppm

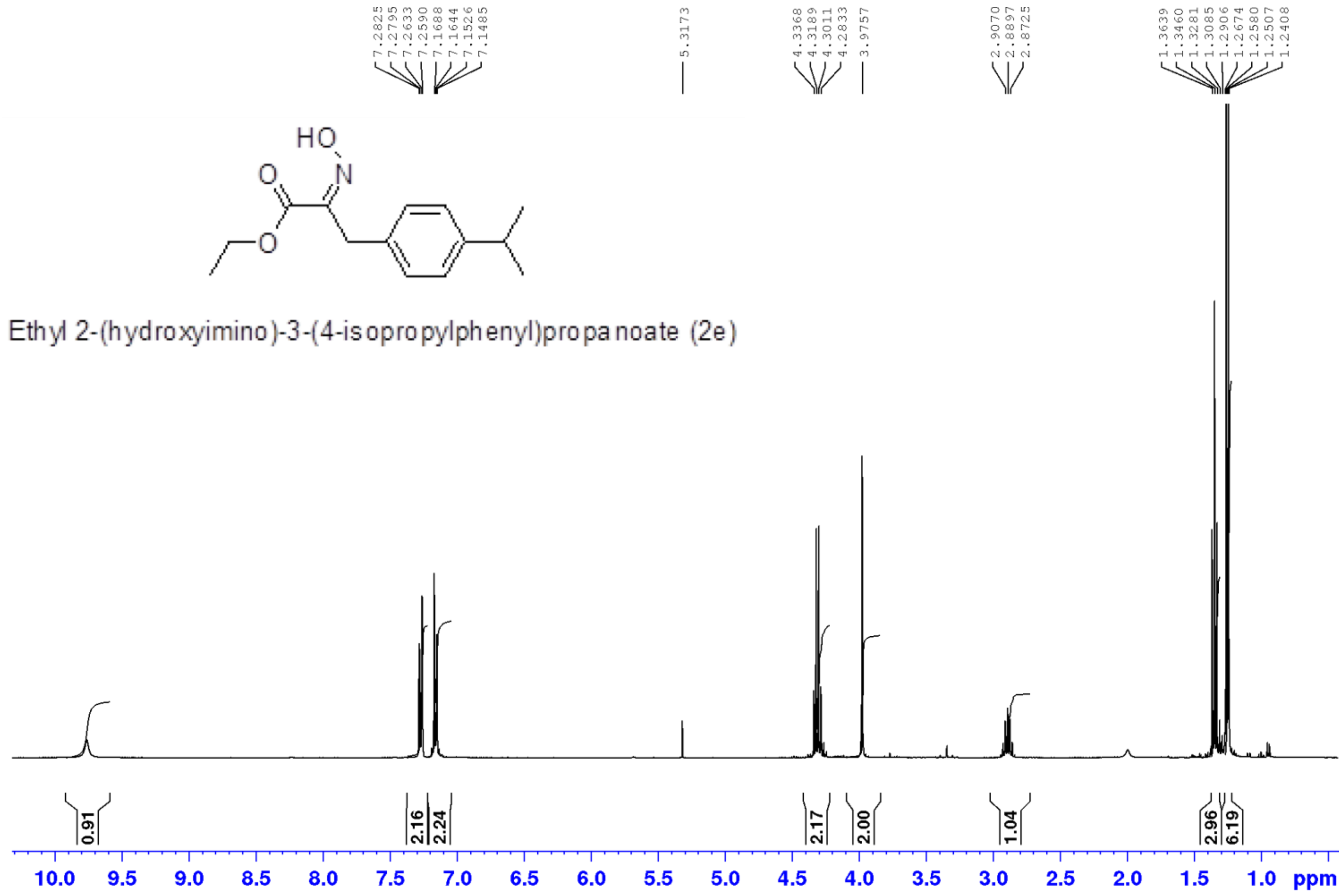
7.2825
7.2795
7.2633
7.2590
7.1688
7.1644
7.1526
7.1485
5.3173
4.3368
4.3189
4.3011
4.2833
3.9757
2.9070
2.8897
2.8725
1.3639
1.3460
1.3281
1.3085
1.2906
1.2674
1.2580
1.2507
1.2408
HO
N
O
O
Ethyl 2-(hydroxyimino)-3-(4-isopropylphenyl)propanoate (2e)
0.91
2.16
2.24
2.17
2.00
1.04
2.96
6.19
10.0 9.5 9.0 8.5 8.0 7.5 7.0 6.5 6.0 5.5 5.0 4.5 4.0 3.5 3.0 2.5 2.0 1.5 1.0 ppm

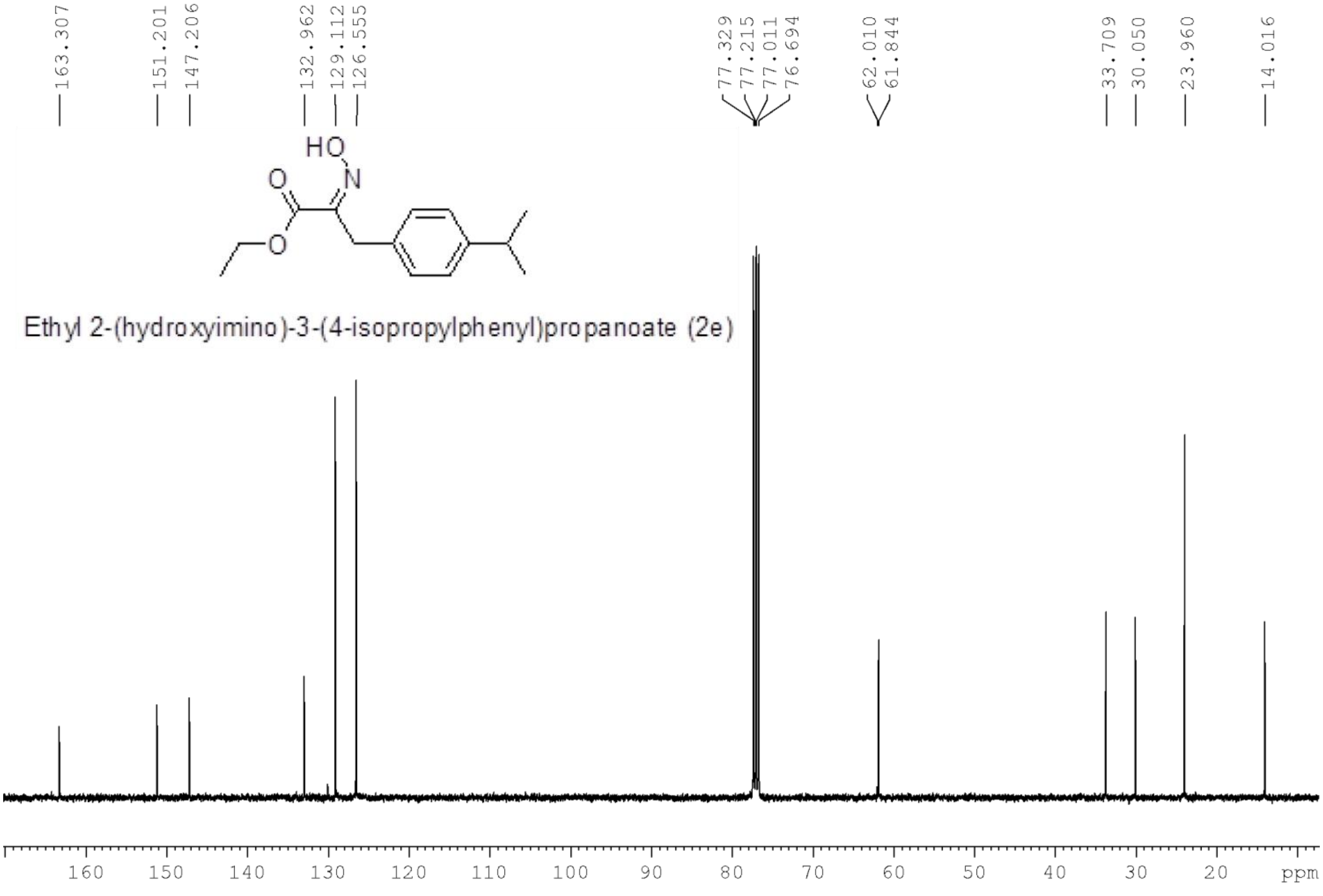
163.307
151.201
147.206
132.962
129.112
126.555
77.329
77.215
77.011
76.694
62.010
61.844
33.709
30.050
23.960
14.016
HO
N
O
O
Ethyl 2-(hydroxyimino)-3-(4-isopropylphenyl)propanoate (2e)
160 150 140 130 120 110 100 90 80 70 60 50 40 30 20 ppm

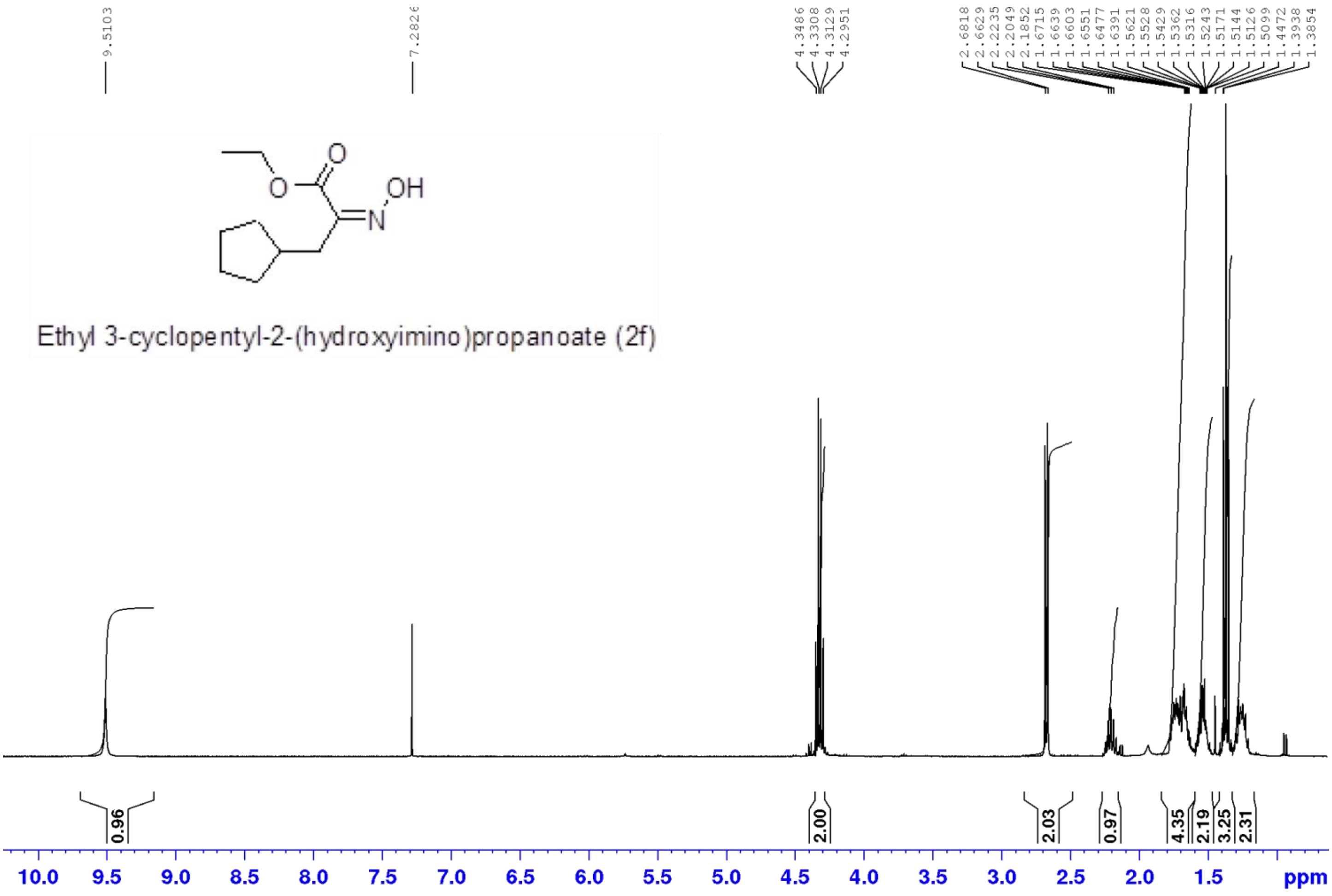

9.5103
7.2826
4.3486
4.3308
4.3129
4.2951
2.6818
2.6629
2.2235
2.2049
2.1852
1.6715
1.6639
1.6603
1.6551
1.6477
1.6391
1.5621
1.5528
1.5429
1.5362
1.5316
1.5243
1.5171
1.5144
1.5126
1.5099
1.4472
1.3938
1.3854
O
O
OH
N
Ethyl 3-cyclopentyl-2-(hydroxyimino)propanoate (2f)
0.96
2.00
2.03
0.97
4.35
2.19
3.25
2.31
10.0
9.5
9.0
8.5
8.0
7.5
7.0
6.5
6.0
5.5
5.0
4.5
4.0
3.5
3.0
2.5
2.0
1.5
ppm

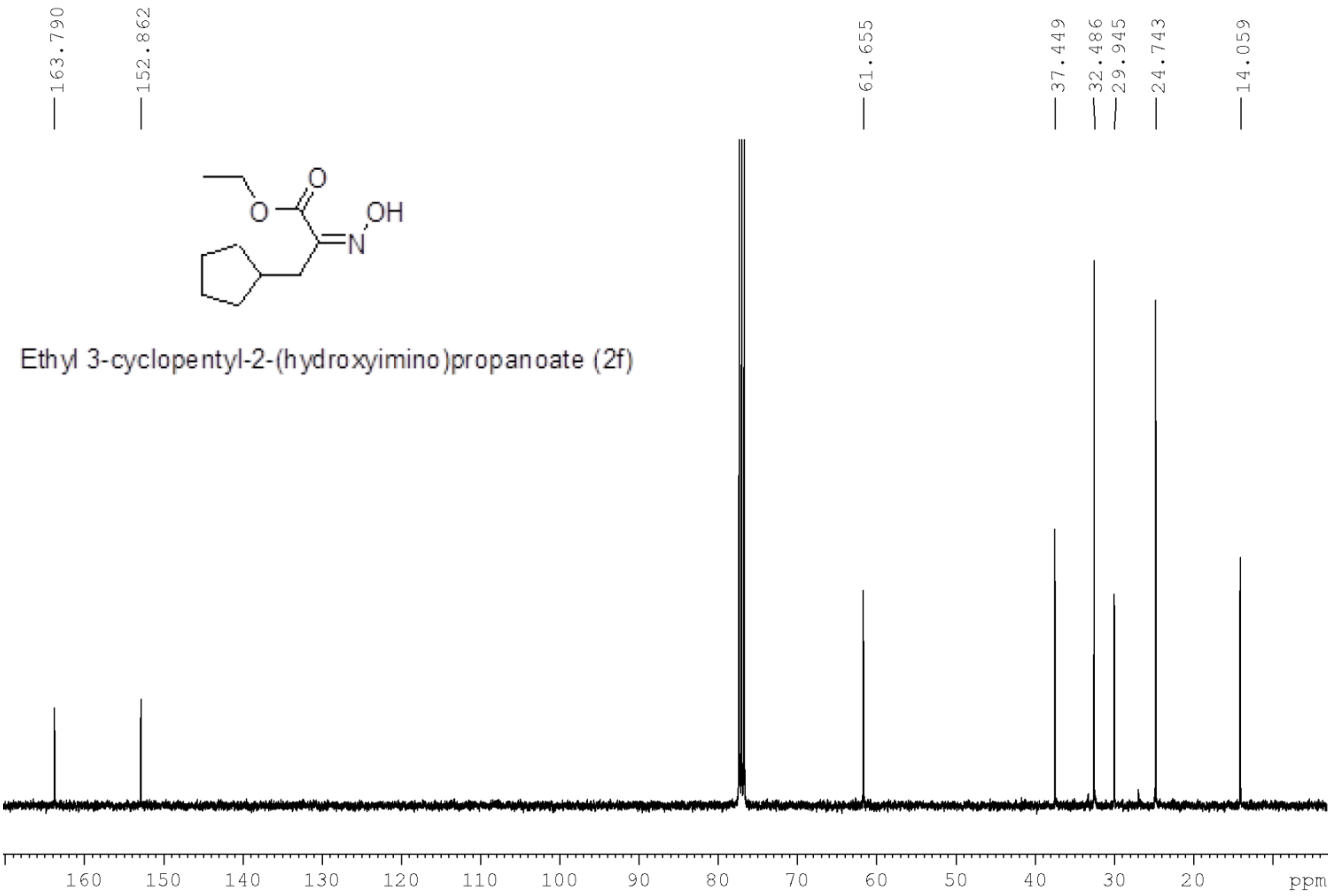

163.790
152.862
61.655
37.449
32.486
29.945
24.743
14.059
O
O
OH
N
Ethyl 3-cyclopentyl-2-(hydroxyimino)propanoate (2f)
160
150
140
130
120
110
100
90
80
70
60
50
40
30
20
ppm

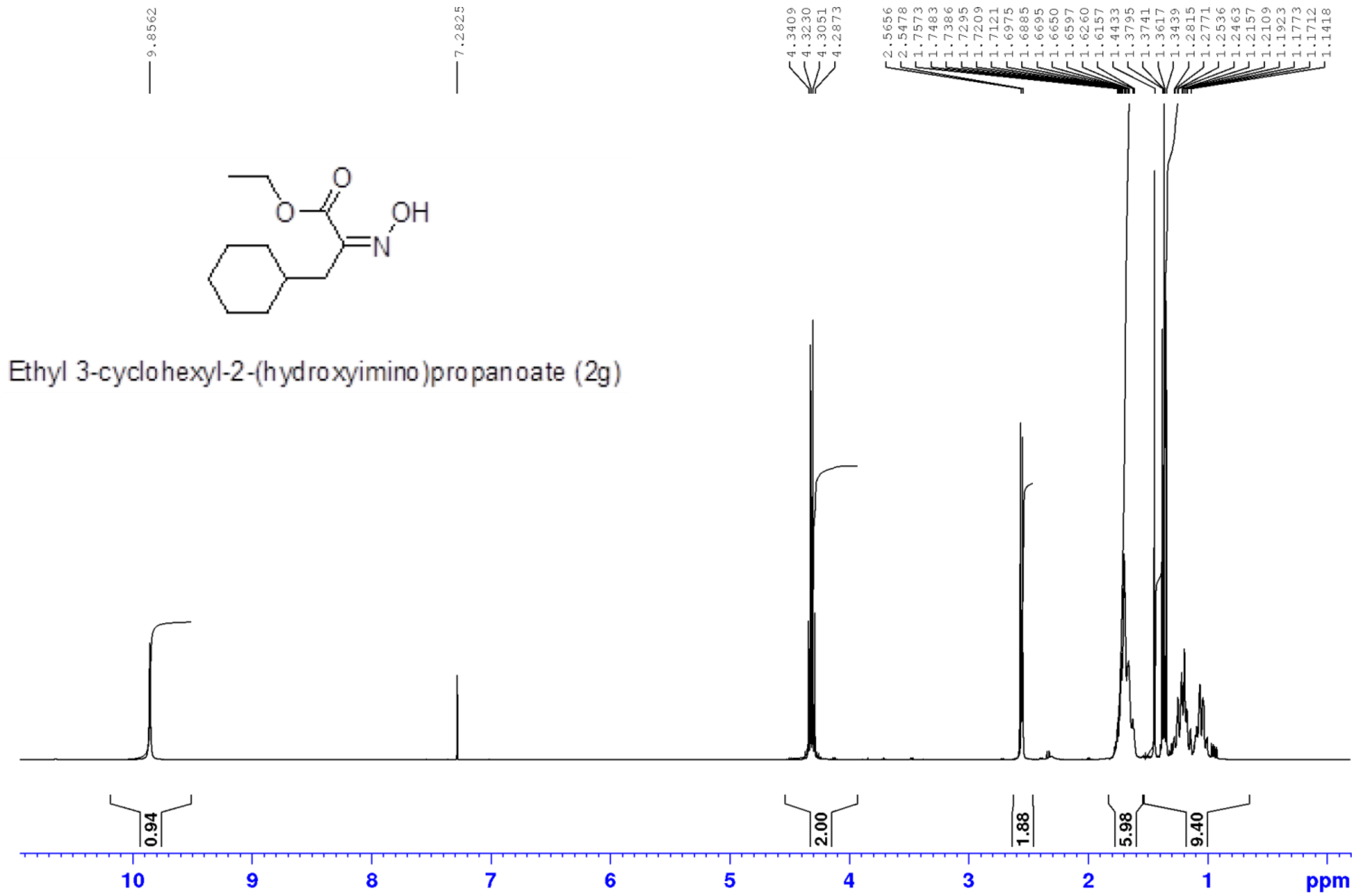
9.8562
7.2825
4.3409
4.3230
4.3051
4.2873
2.5656
2.5478
1.7573
1.7483
1.7386
1.7295
1.7209
1.7121
1.6975
1.6885
1.6695
1.6650
1.6597
1.6260
1.6157
1.4433
1.3795
1.3741
1.3617
1.3439
1.2815
1.2771
1.2536
1.2463
1.2157
1.2109
1.1923
1.1773
1.1712
1.1418
O
OH
N
Ethyl 3-cyclohexyl-2-(hydroxyimino)propanoate (2g)
0.94
2.00
1.88
5.98
9.40
10
9
8
7
6
5
4
3
2
1
ppm

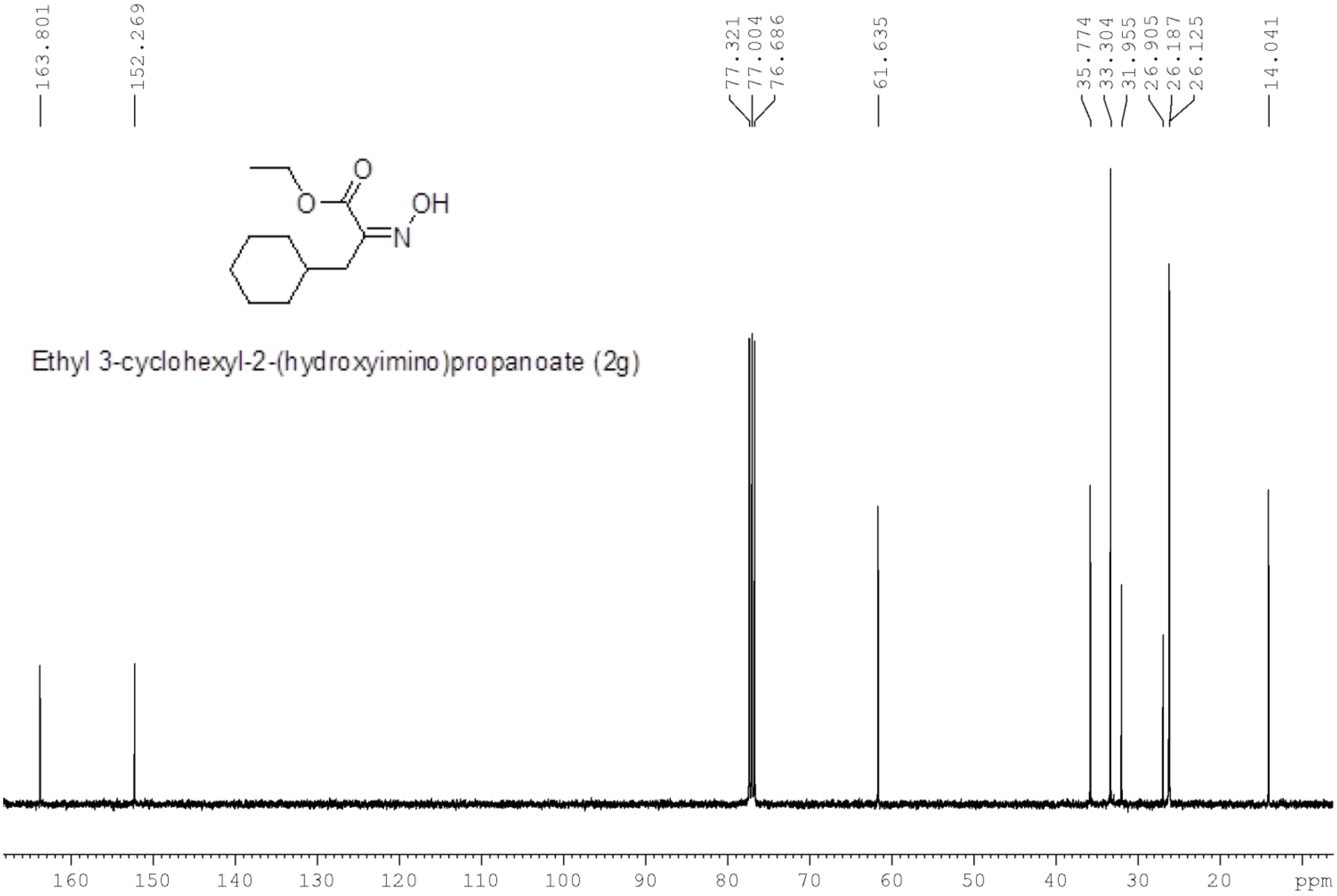
163.801
152.269
77.321
77.004
76.686
61.635
35.774
33.304
31.955
26.905
26.187
26.125
14.041
O
OH
N
Ethyl 3-cyclohexyl-2-(hydroxyimino)propanoate (2g)
160
150
140
130
120
110
100
90
80
70
60
50
40
30
20
ppm

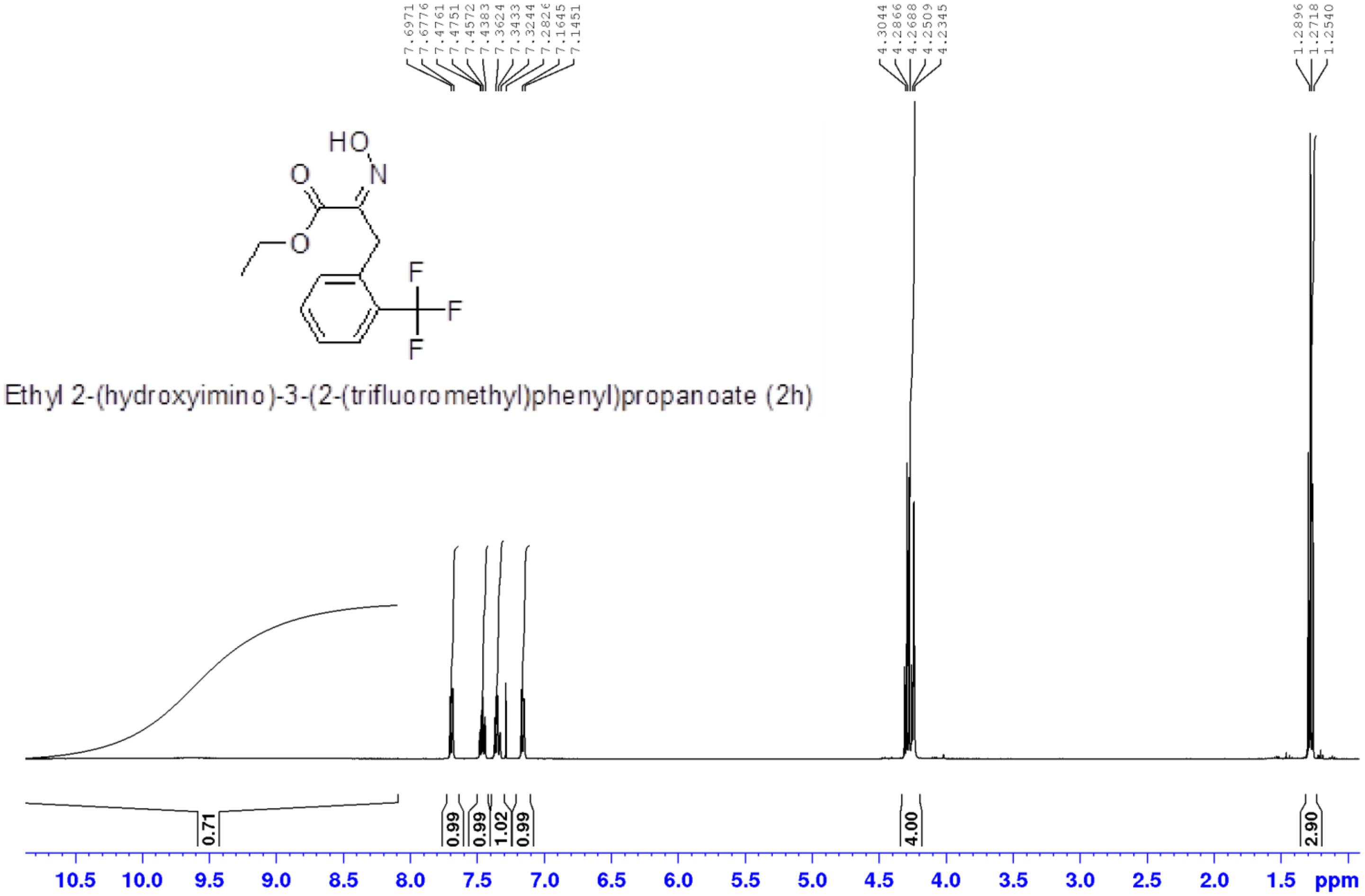
7.6971
7.6776
7.4761
7.4751
7.4572
7.4383
7.3624
7.3433
7.3244
7.2826
7.1645
7.1451
4.3044
4.2866
4.2688
4.2509
4.2345
1.2896
1.2718
1.2540
HO
N
O
O
F
F
F
Ethyl 2-(hydroxyimino)-3-(2-(trifluoromethyl)phenyl)propanoate (2h)
0.71
0.99
0.99
1.02
0.99
4.00
2.90
10.5 10.0 9.5 9.0 8.5 8.0 7.5 7.0 6.5 6.0 5.5 5.0 4.5 4.0 3.5 3.0 2.5 2.0 1.5 ppm

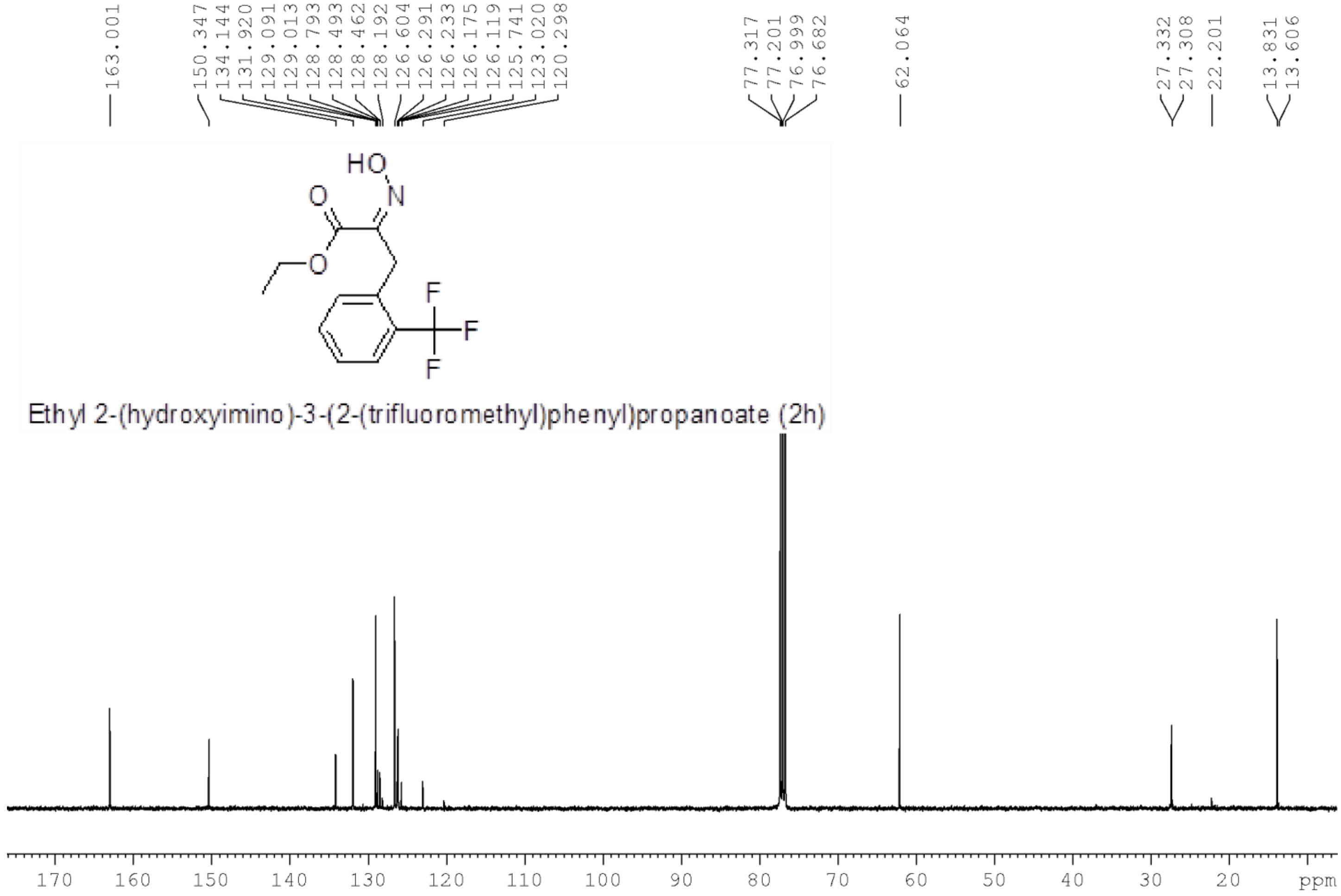
163.001
150.347
134.144
131.920
129.091
129.013
128.793
128.493
128.462
128.192
126.604
126.291
126.233
126.175
126.119
125.741
123.020
120.298
77.317
77.201
76.999
76.682
62.064
27.332
27.308
22.201
13.831
13.606
HO
N
O
O
F
F
F
Ethyl 2-(hydroxyimino)-3-(2-(trifluoromethyl)phenyl)propanoate (2h)
170 160 150 140 130 120 110 100 90 80 70 60 50 40 30 20 ppm

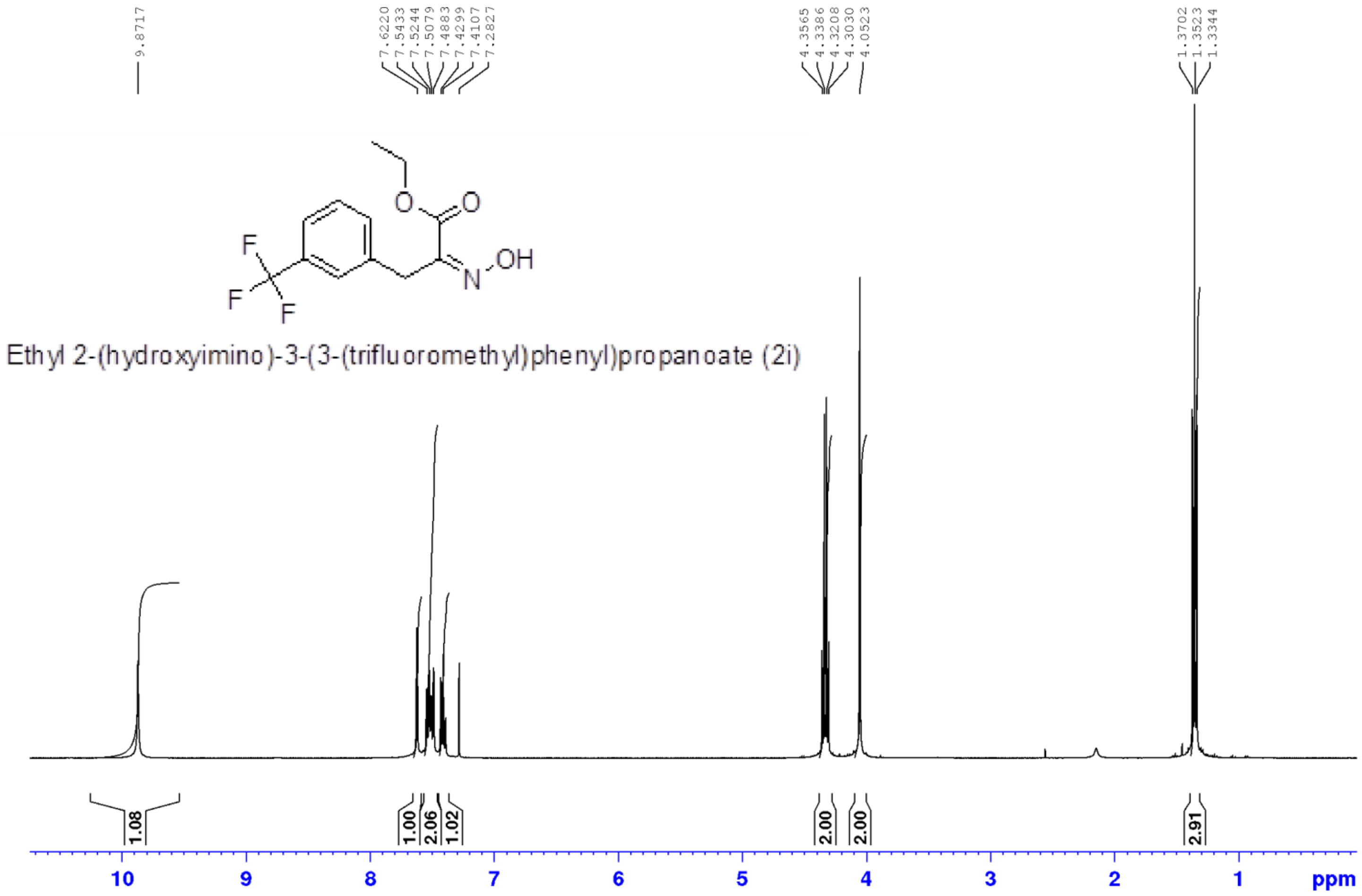
9.8717
7.6220 7.5433 7.5244 7.5079 7.4883 7.4299 7.4107 7.2827
4.3565 4.3386 4.3208 4.3030 4.0523
1.3702 1.3523 1.3344
Ethyl 2-(hydroxyimino)-3-(3-(trifluoromethyl)phenyl)propanoate (2i)
1.08
1.00 2.06 1.02
2.00 2.00
2.91
ppm

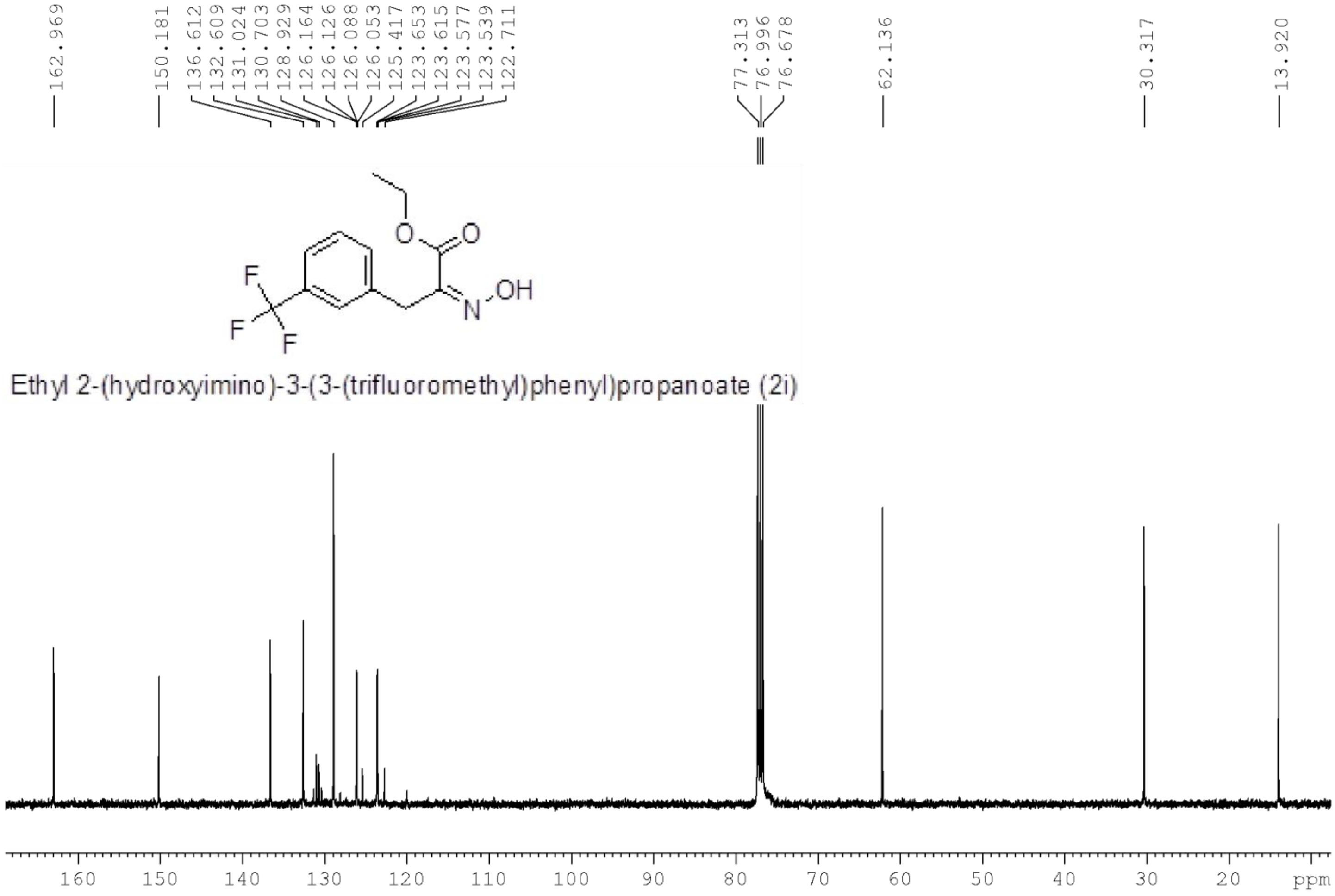
162.969
150.181
136.612 132.609 131.024 130.703 128.929 126.164 126.126 126.088 126.053 125.417 123.653 123.615 123.577 123.539 122.711
77.313 76.996 76.678
62.136
30.317
13.920
Ethyl 2-(hydroxyimino)-3-(3-(trifluoromethyl)phenyl)propanoate (2i)
ppm

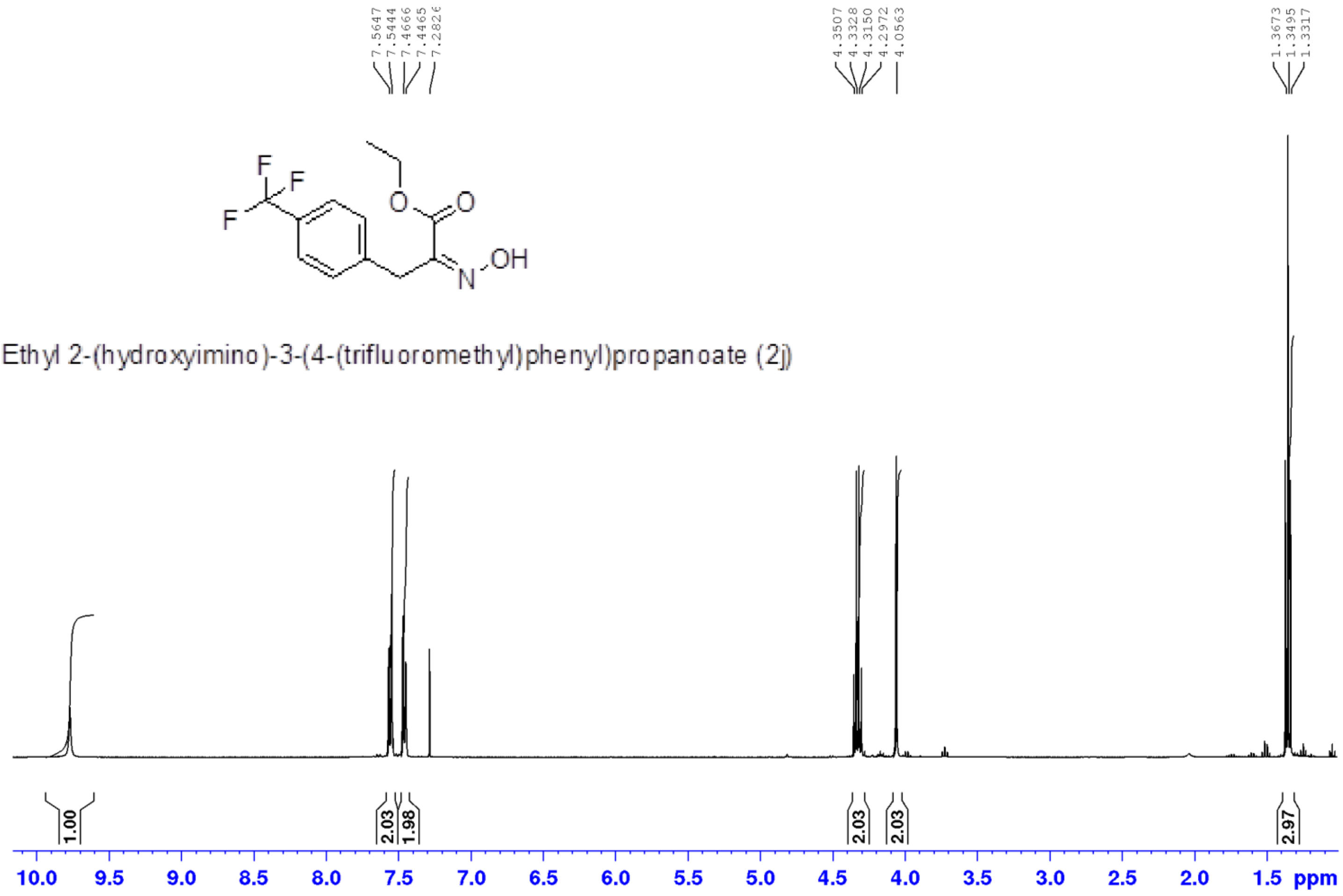
7.5647
7.5444
7.4666
7.4465
7.2826
4.3507
4.3328
4.3150
4.2972
4.0563
1.3673
1.3495
1.3317
Ethyl 2-(hydroxyimino)-3-(4-(trifluoromethyl)phenyl)propanoate (2j)
1.00
2.03
1.98
2.03
2.03
2.97
10.0 9.5 9.0 8.5 8.0 7.5 7.0 6.5 6.0 5.5 5.0 4.5 4.0 3.5 3.0 2.5 2.0 1.5 ppm

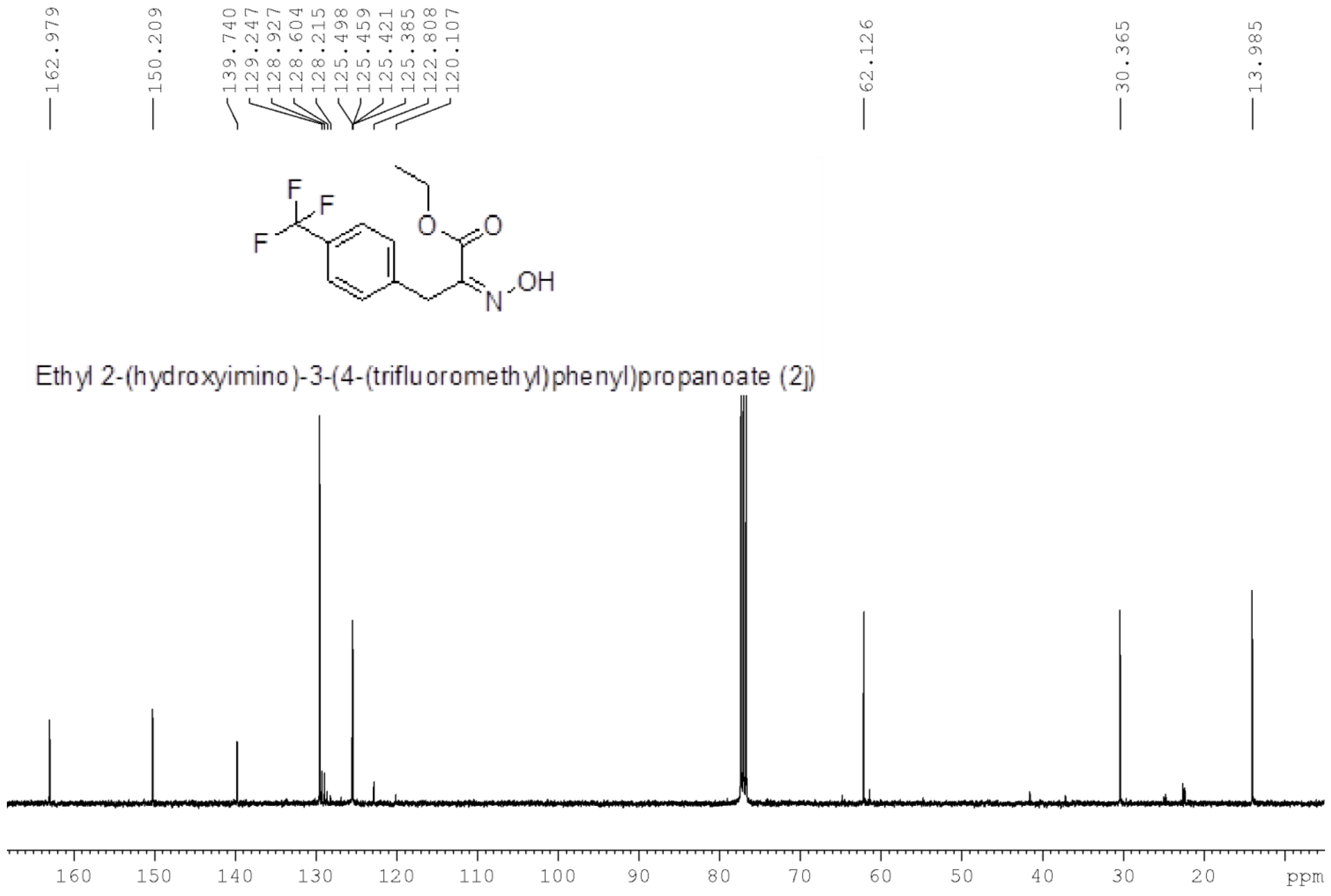
162.979
150.209
139.740
129.247
128.927
128.604
128.215
125.498
125.459
125.421
125.385
122.808
120.107
62.126
30.365
13.985
Ethyl 2-(hydroxyimino)-3-(4-(trifluoromethyl)phenyl)propanoate (2j)
160 150 140 130 120 110 100 90 80 70 60 50 40 30 20 ppm

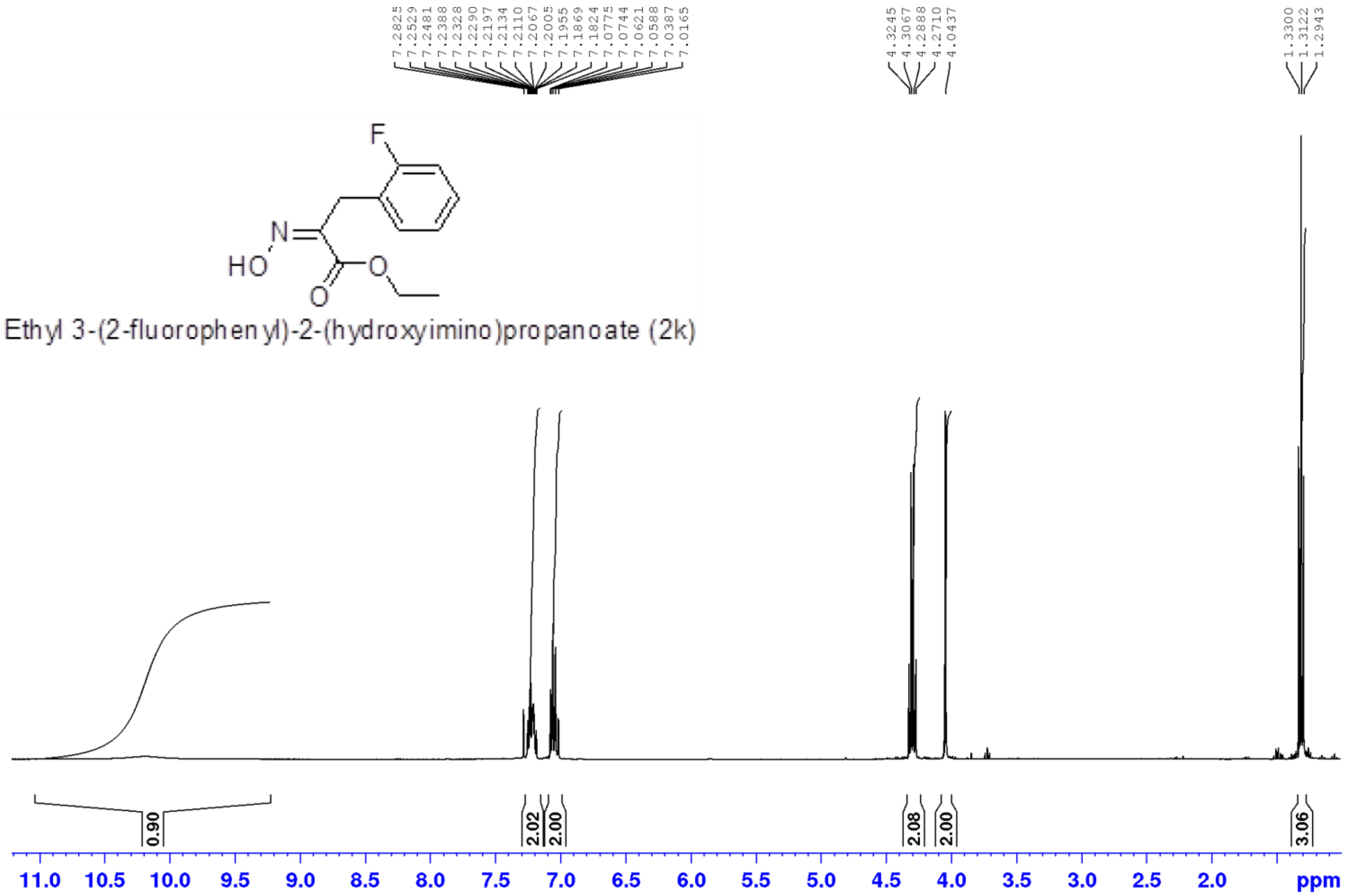
7.2825
7.2529
7.2481
7.2388
7.2328
7.2290
7.2197
7.2134
7.2110
7.2067
7.2005
7.1955
7.1869
7.1824
7.0775
7.0744
7.0621
7.0588
7.0387
7.0165
4.3245
4.3067
4.2888
4.2710
4.0437
1.3300
1.3122
1.2943
Ethyl 3-(2-fluorophenyl)-2-(hydroxyimino)propanoate (2k)
0.90
2.02
2.00
2.08
2.00
3.06
ppm

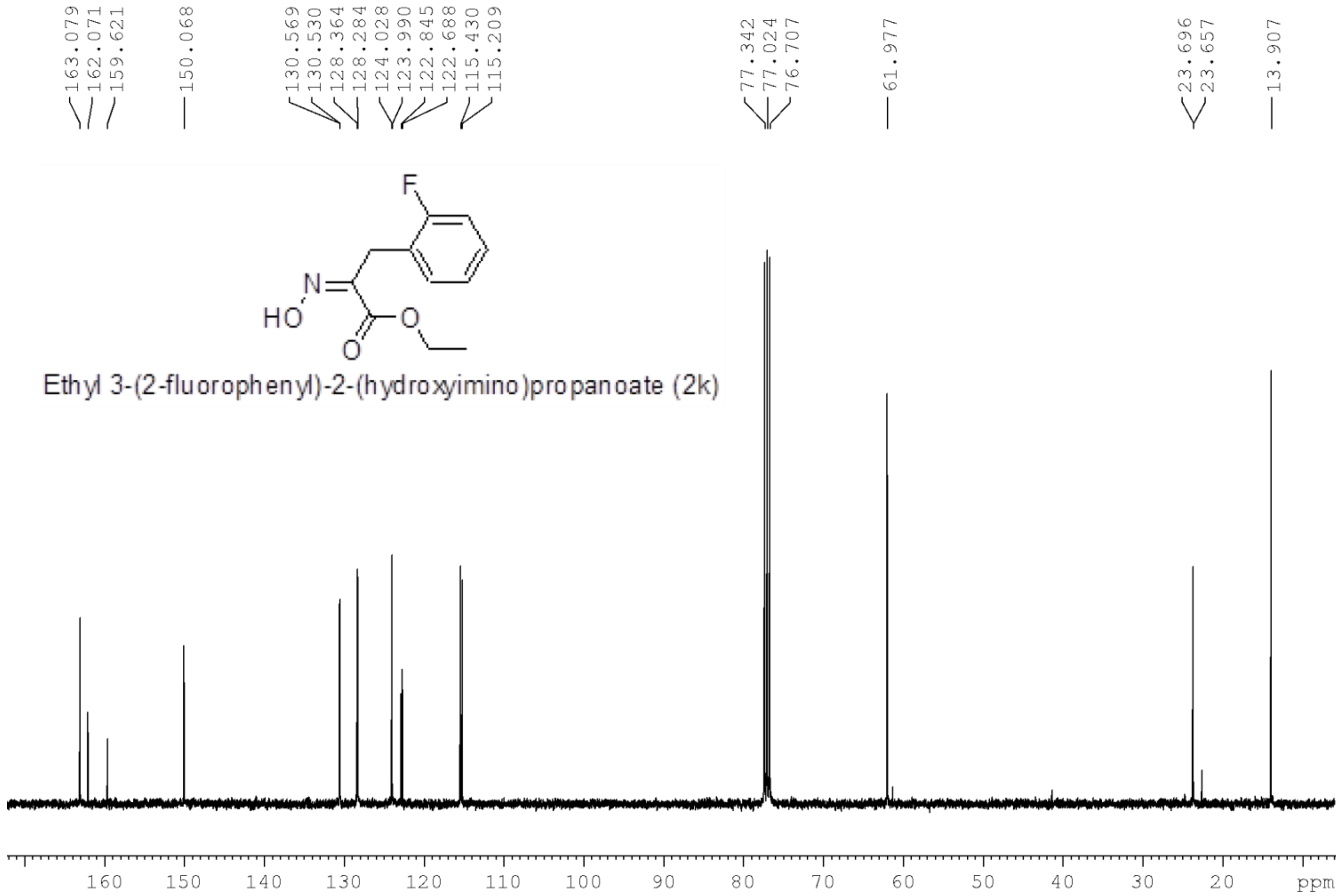
163.079
162.071
159.621
150.068
130.569
130.530
128.364
128.284
124.028
123.990
122.845
122.688
115.430
115.209
77.342
77.024
76.707
61.977
23.696
23.657
13.907
Ethyl 3-(2-fluorophenyl)-2-(hydroxyimino)propanoate (2k)
ppm

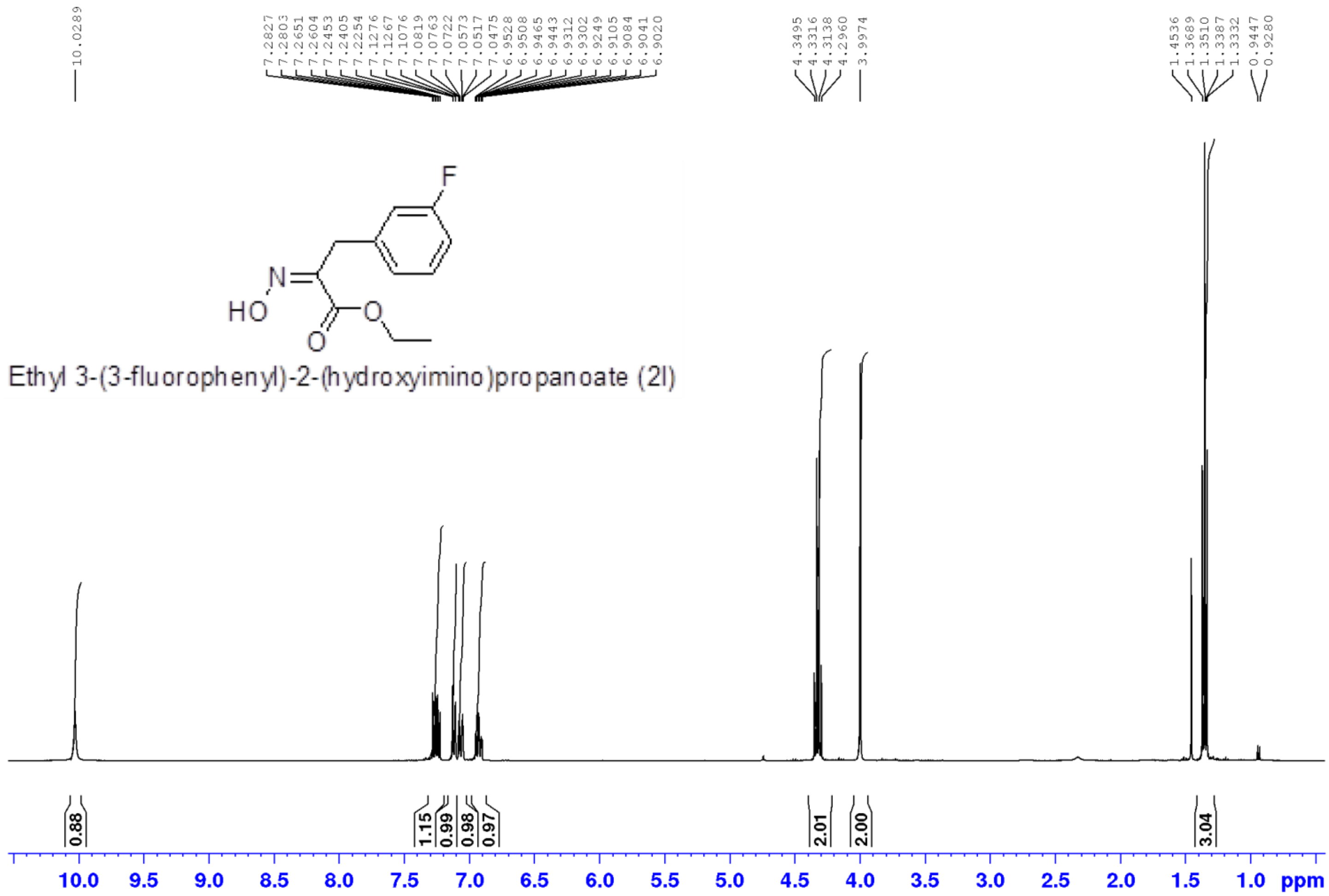
10.0289
7.2827 7.2803 7.2651 7.2604 7.2453 7.2405 7.2254 7.1276 7.1267 7.1076 7.0819 7.0763 7.0722 7.0573 7.0517 7.0475 6.9528 6.9508 6.9465 6.9443 6.9312 6.9302 6.9249 6.9105 6.9084 6.9041 6.9020
4.3495 4.3316 4.3138 4.2960 3.9974
1.4536 1.3689 1.3510 1.3387 1.3332 0.9447 0.9280
F
N
HO
O
O
Ethyl 3-(3-fluorophenyl)-2-(hydroxyimino)propanoate (2l)
0.88
1.15 0.99 0.98 0.97
2.01 2.00
3.04
10.0 9.5 9.0 8.5 8.0 7.5 7.0 6.5 6.0 5.5 5.0 4.5 4.0 3.5 3.0 2.5 2.0 1.5 1.0 ppm

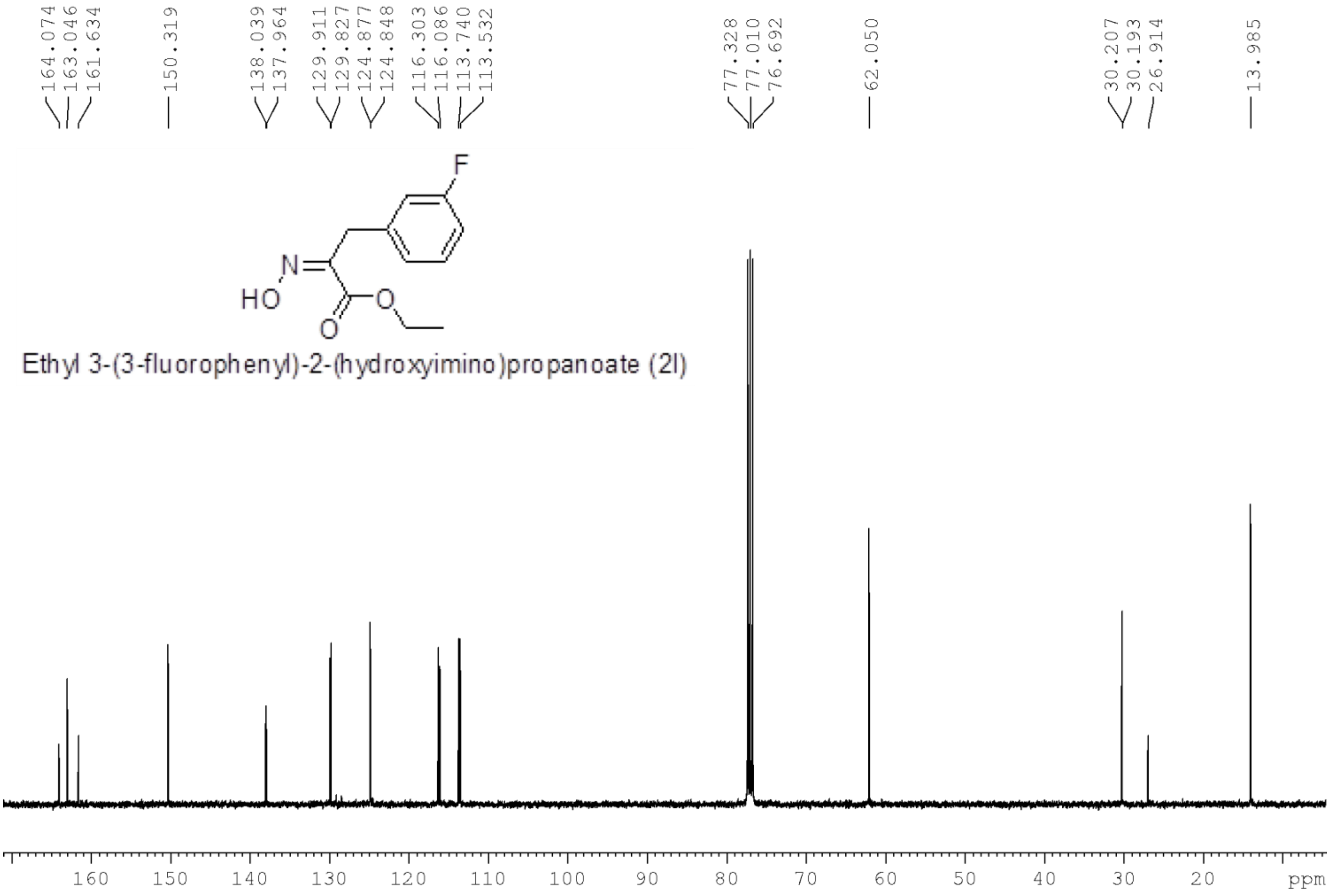
164.074 163.046 161.634
150.319
138.039 137.964
129.911 129.827 124.877 124.848
116.303 116.086 113.740 113.532
77.328 77.010 76.692
62.050
30.207 30.193 26.914
13.985
F
N
HO
O
O
Ethyl 3-(3-fluorophenyl)-2-(hydroxyimino)propanoate (2l)
160 150 140 130 120 110 100 90 80 70 60 50 40 30 20 ppm

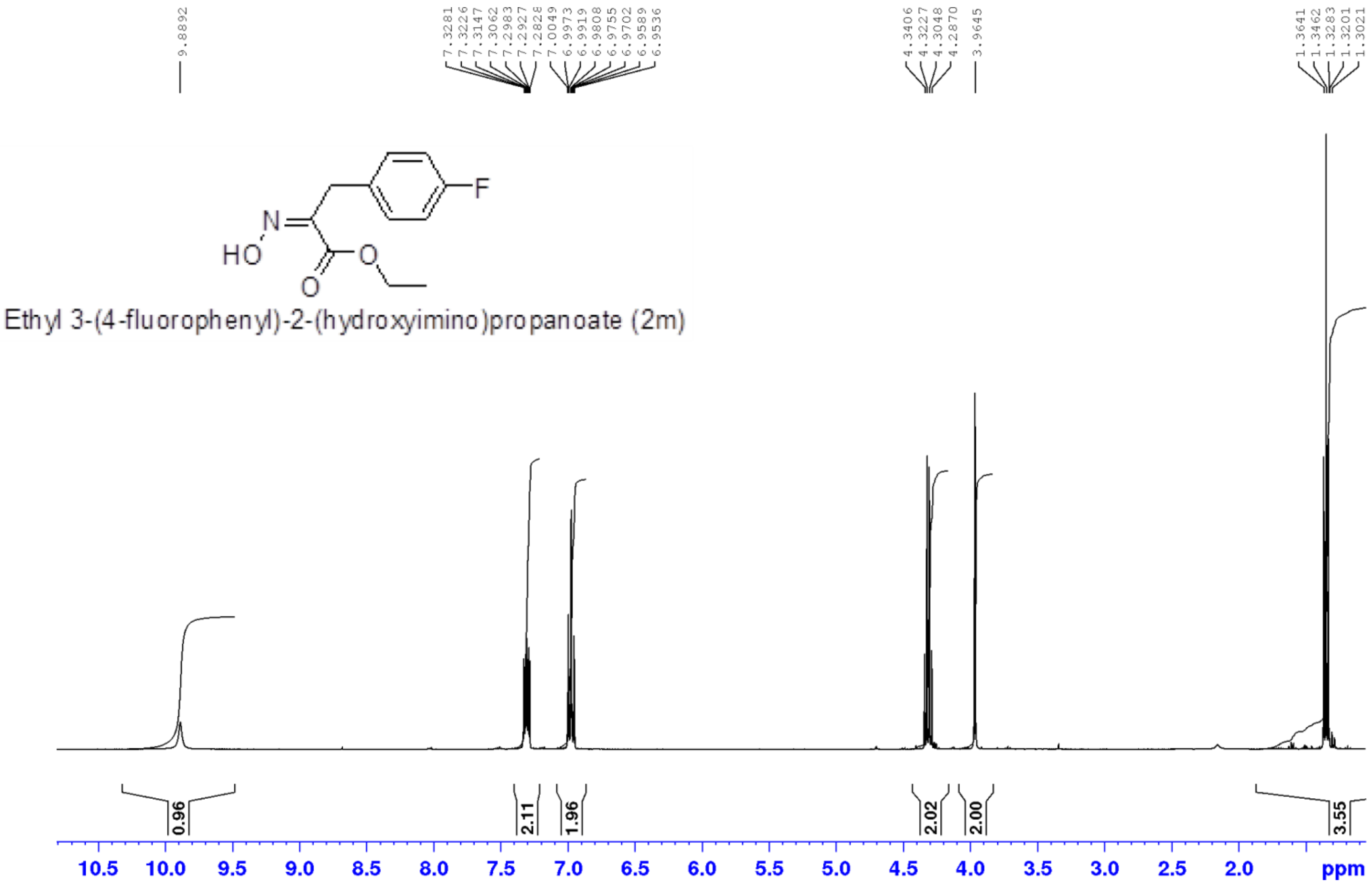
9.8892
7.3281
7.3226
7.3147
7.3062
7.2983
7.2927
7.2828
7.0049
6.9973
6.9919
6.9808
6.9755
6.9702
6.9589
6.9536
4.3406
4.3227
4.3048
4.2870
3.9645
1.3641
1.3462
1.3283
1.3201
1.3021
N
HO
O
O
F
Ethyl 3-(4-fluorophenyl)-2-(hydroxyimino)propanoate (2m)
0.96
2.11
1.96
2.02
2.00
3.55
10.5
10.0
9.5
9.0
8.5
8.0
7.5
7.0
6.5
6.0
5.5
5.0
4.5
4.0
3.5
3.0
2.5
2.0
ppm

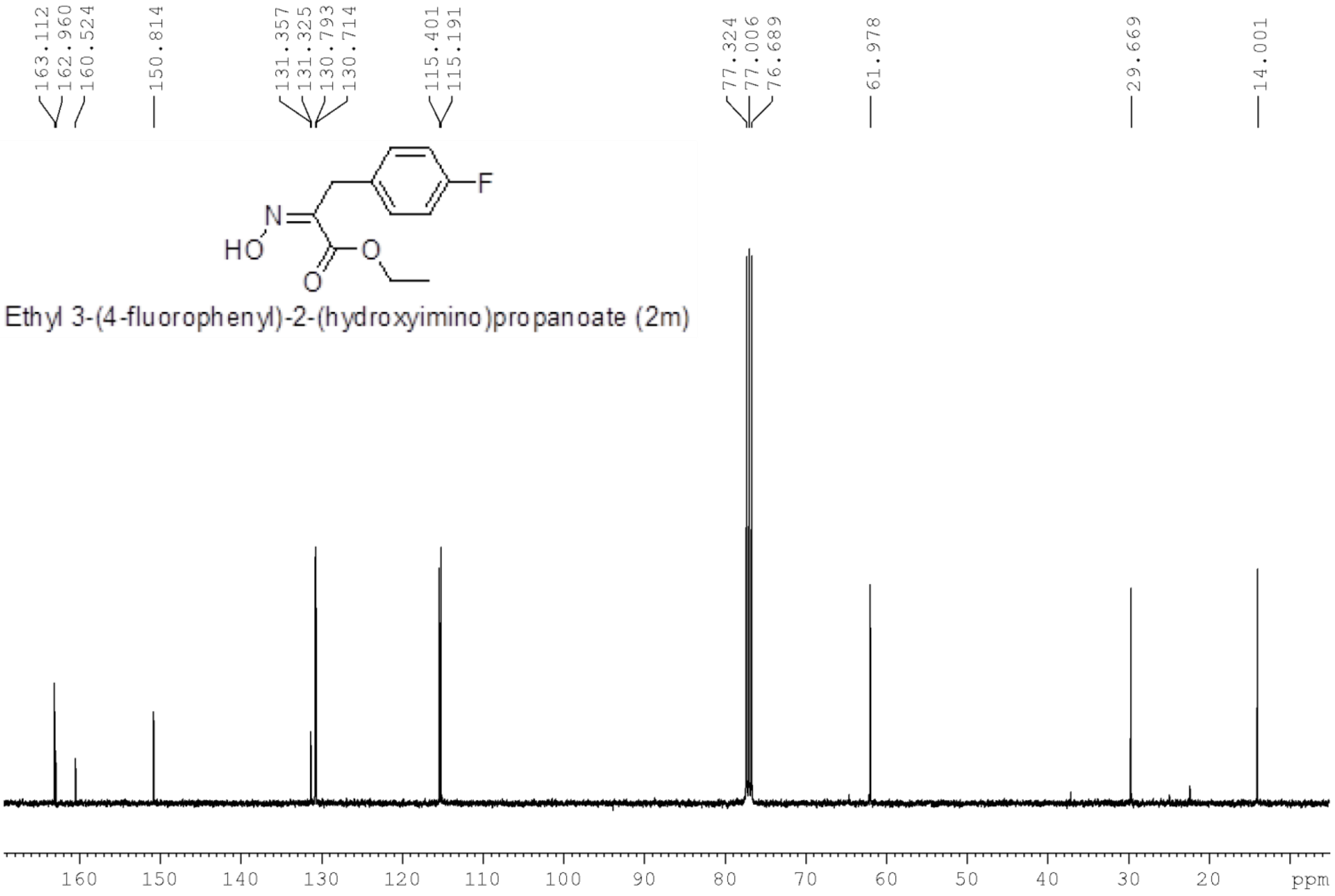
163.112
162.960
160.524
150.814
131.357
131.325
130.793
130.714
115.401
115.191
77.324
77.006
76.689
61.978
29.669
14.001
N
HO
O
O
F
Ethyl 3-(4-fluorophenyl)-2-(hydroxyimino)propanoate (2m)
160
150
140
130
120
110
100
90
80
70
60
50
40
30
20
ppm

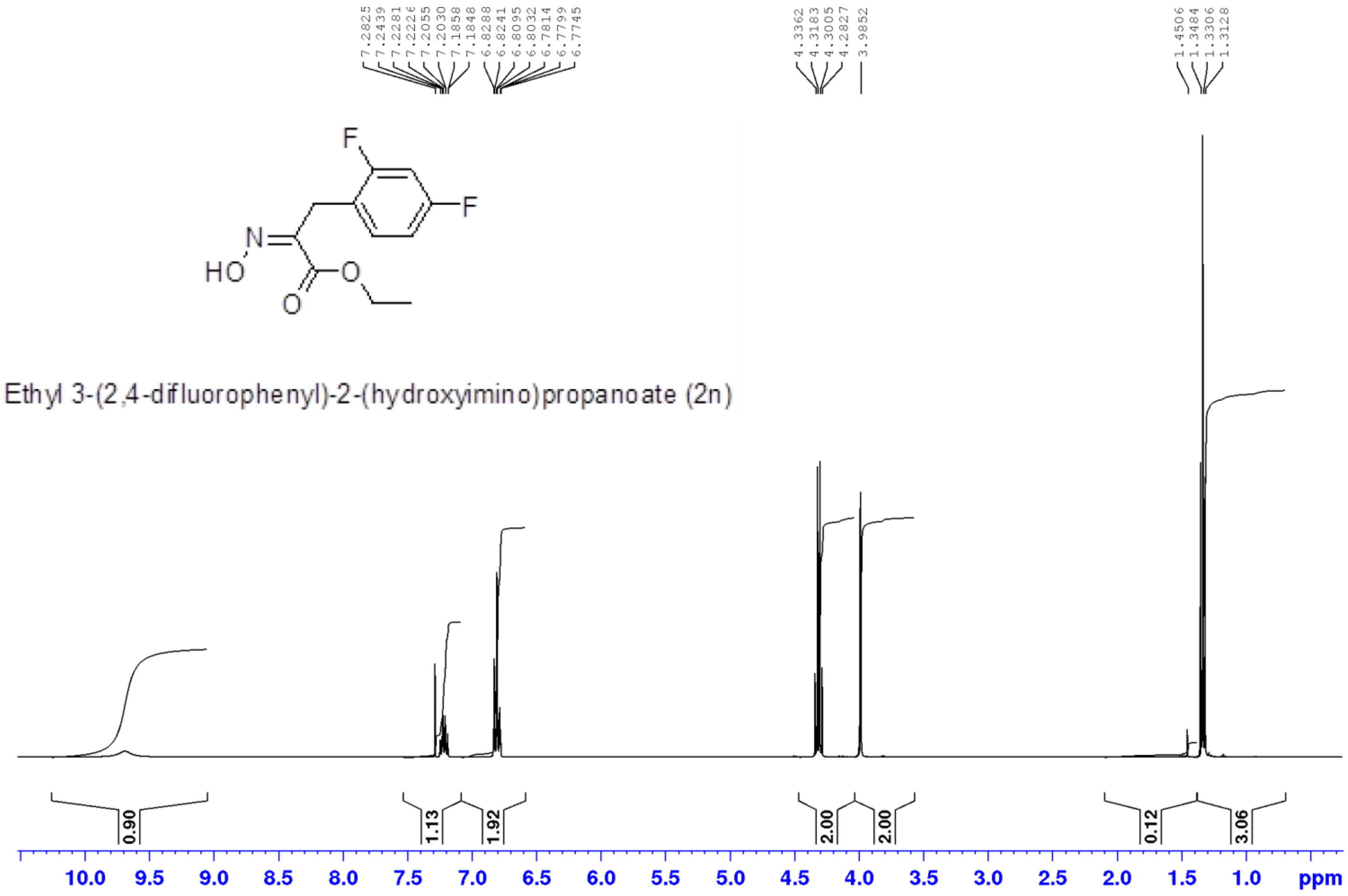
Ethyl 3-(2,4-difluorophenyl)-2-(hydroxyimino)propanoate (2n)
ppm

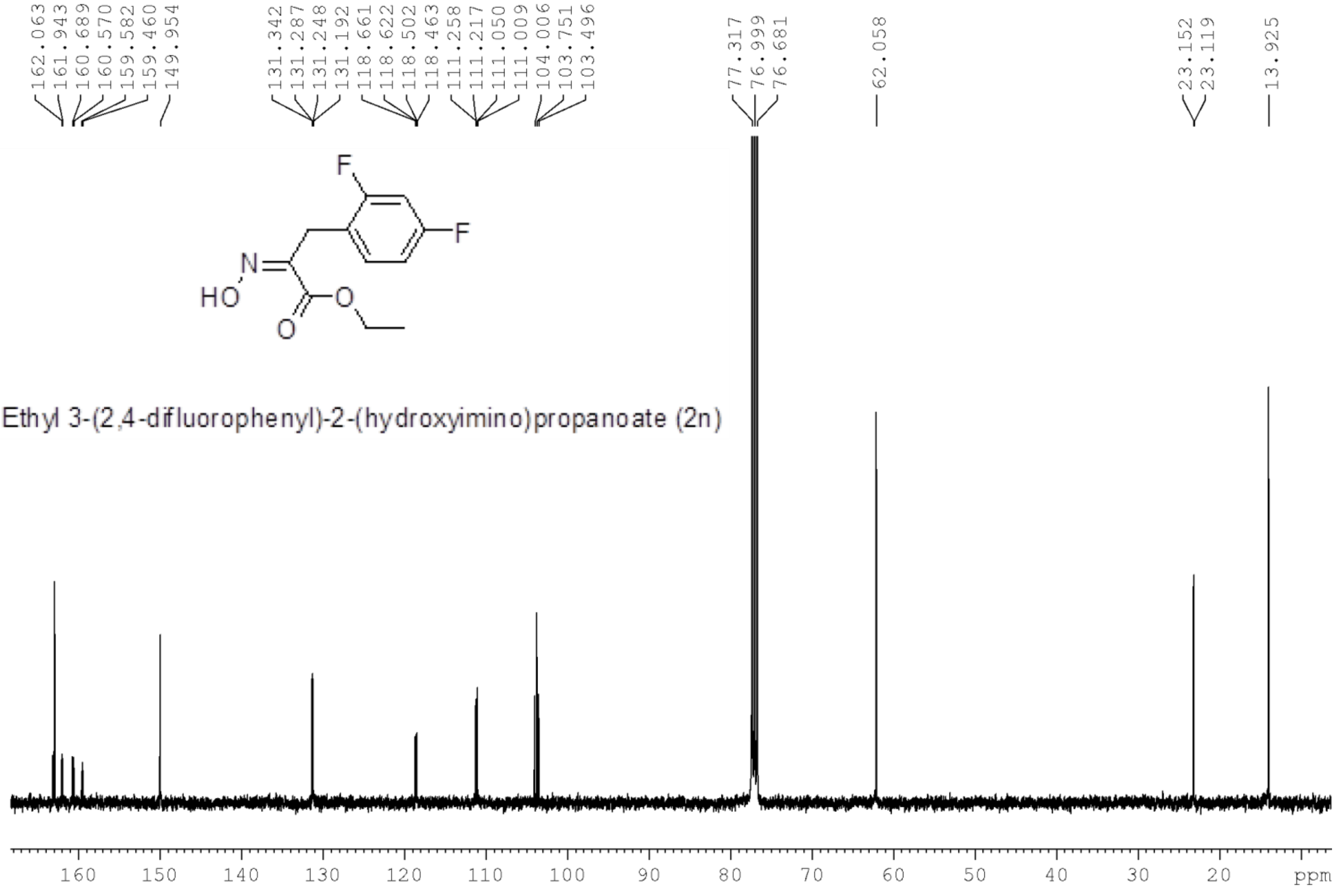
Ethyl 3-(2,4-difluorophenyl)-2-(hydroxyimino)propanoate (2n)
ppm

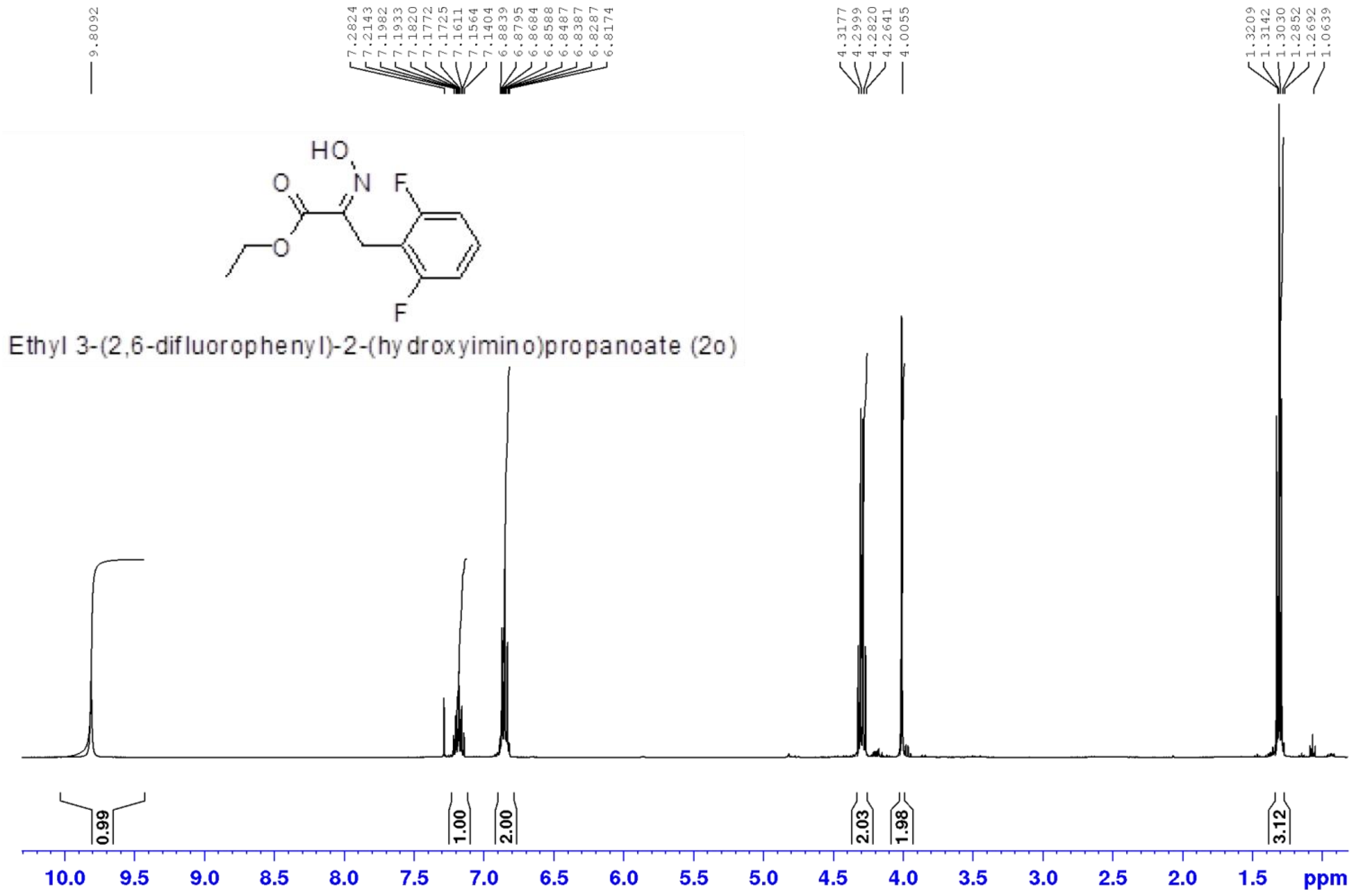

Ethyl 3-(2,6-difluorophenyl)-2-(hydroxyimino)propanoate (2o)

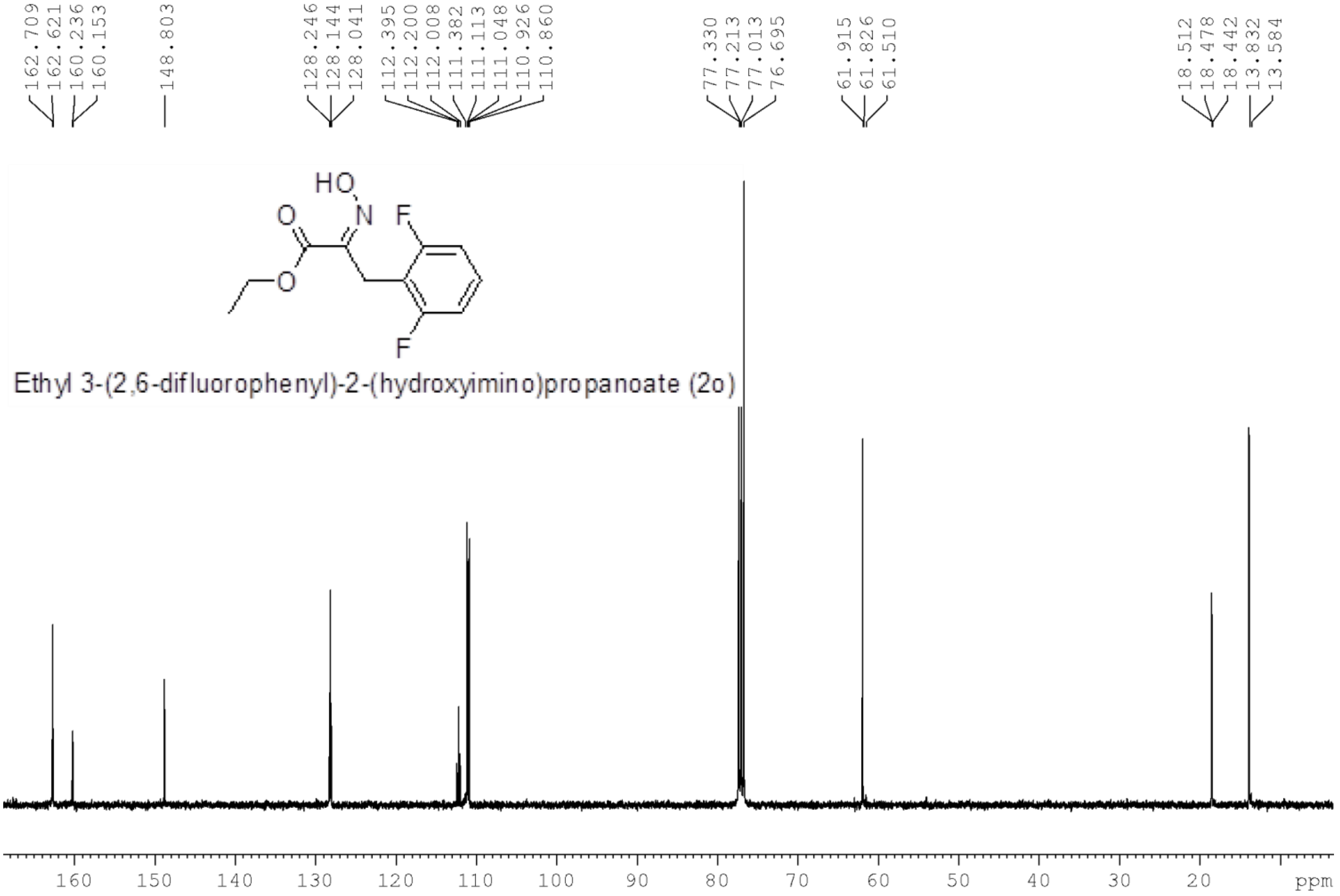

Ethyl 3-(2,6-difluorophenyl)-2-(hydroxyimino)propanoate (2o)

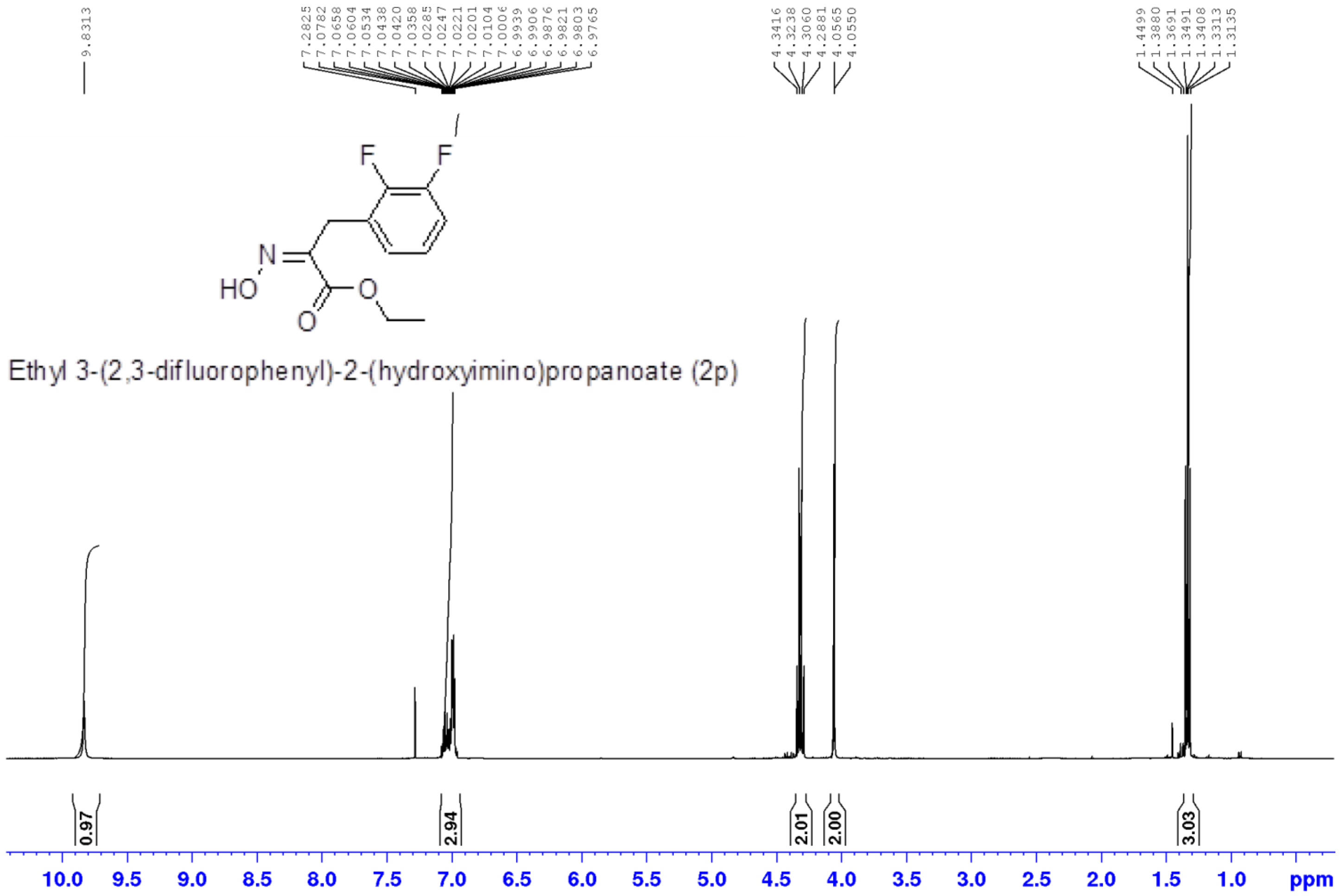
9.8313
7.2825
7.0782
7.0658
7.0604
7.0534
7.0438
7.0420
7.0358
7.0285
7.0247
7.0221
7.0201
7.0104
7.0006
6.9939
6.9906
6.9876
6.9821
6.9803
6.9765
4.3416
4.3238
4.3060
4.2881
4.0565
4.0550
1.4499
1.3880
1.3691
1.3491
1.3408
1.3313
1.3135
Ethyl 3-(2,3-difluorophenyl)-2-(hydroxyimino)propanoate (2p)
0.97
2.94
2.01
2.00
3.03
ppm

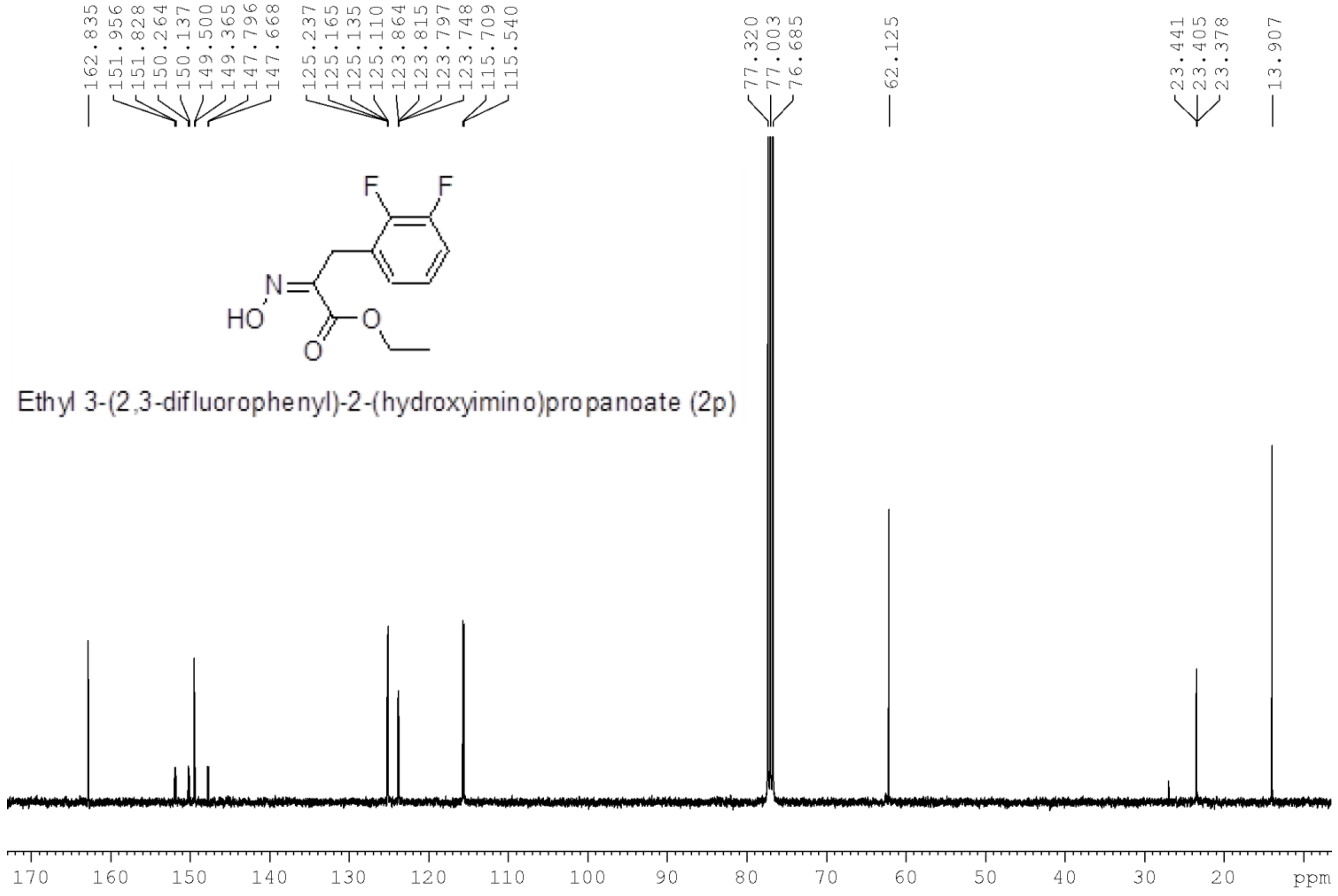
162.835
151.956
151.828
150.264
150.137
149.500
149.365
147.796
147.668
125.237
125.165
125.135
125.110
123.864
123.815
123.797
123.748
115.709
115.540
77.320
77.003
76.685
62.125
23.441
23.405
23.378
13.907
Ethyl 3-(2,3-difluorophenyl)-2-(hydroxyimino)propanoate (2p)
ppm

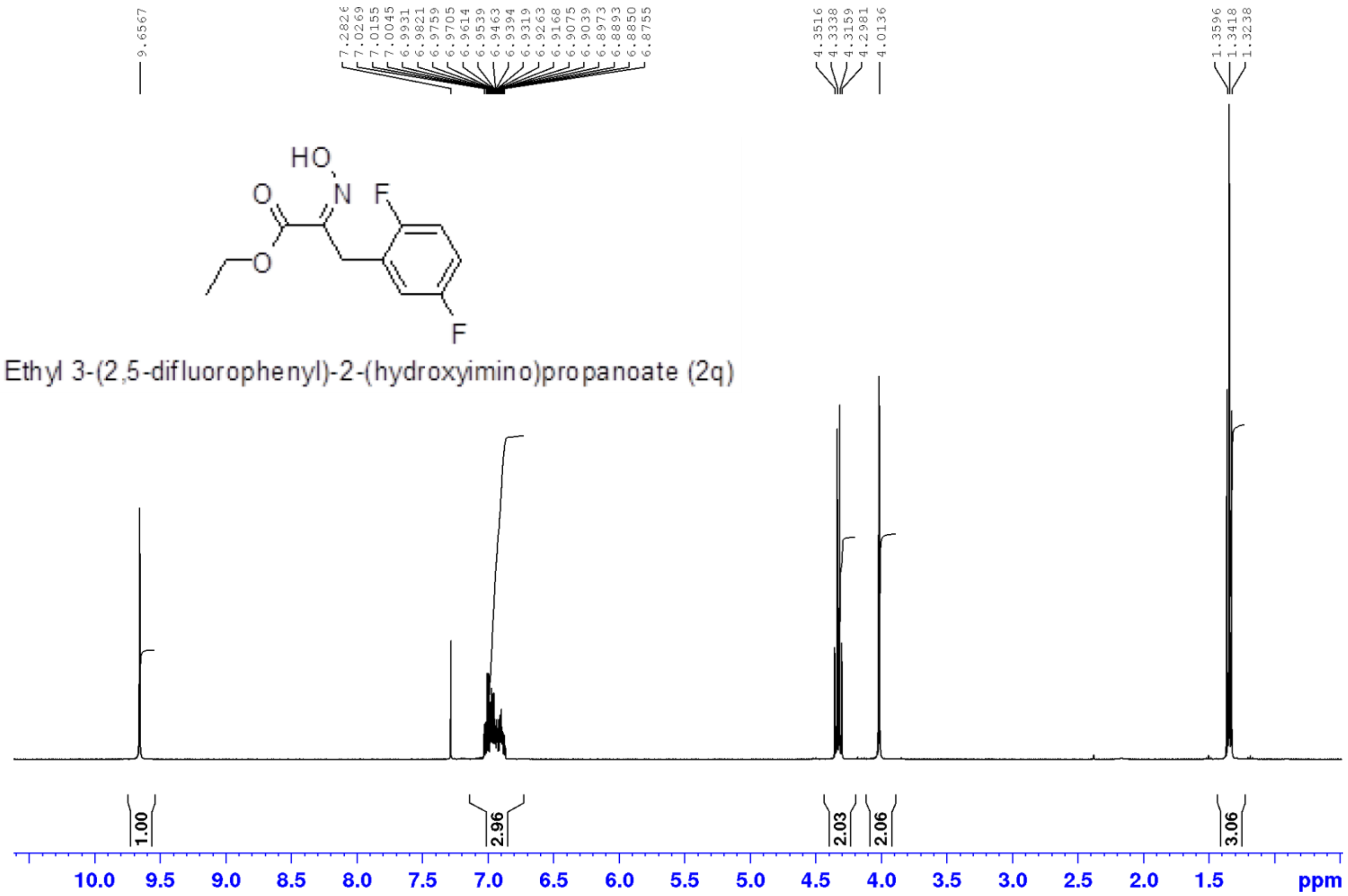

Ethyl 3-(2,5-difluorophenyl)-2-(hydroxyimino)propanoate (2q)

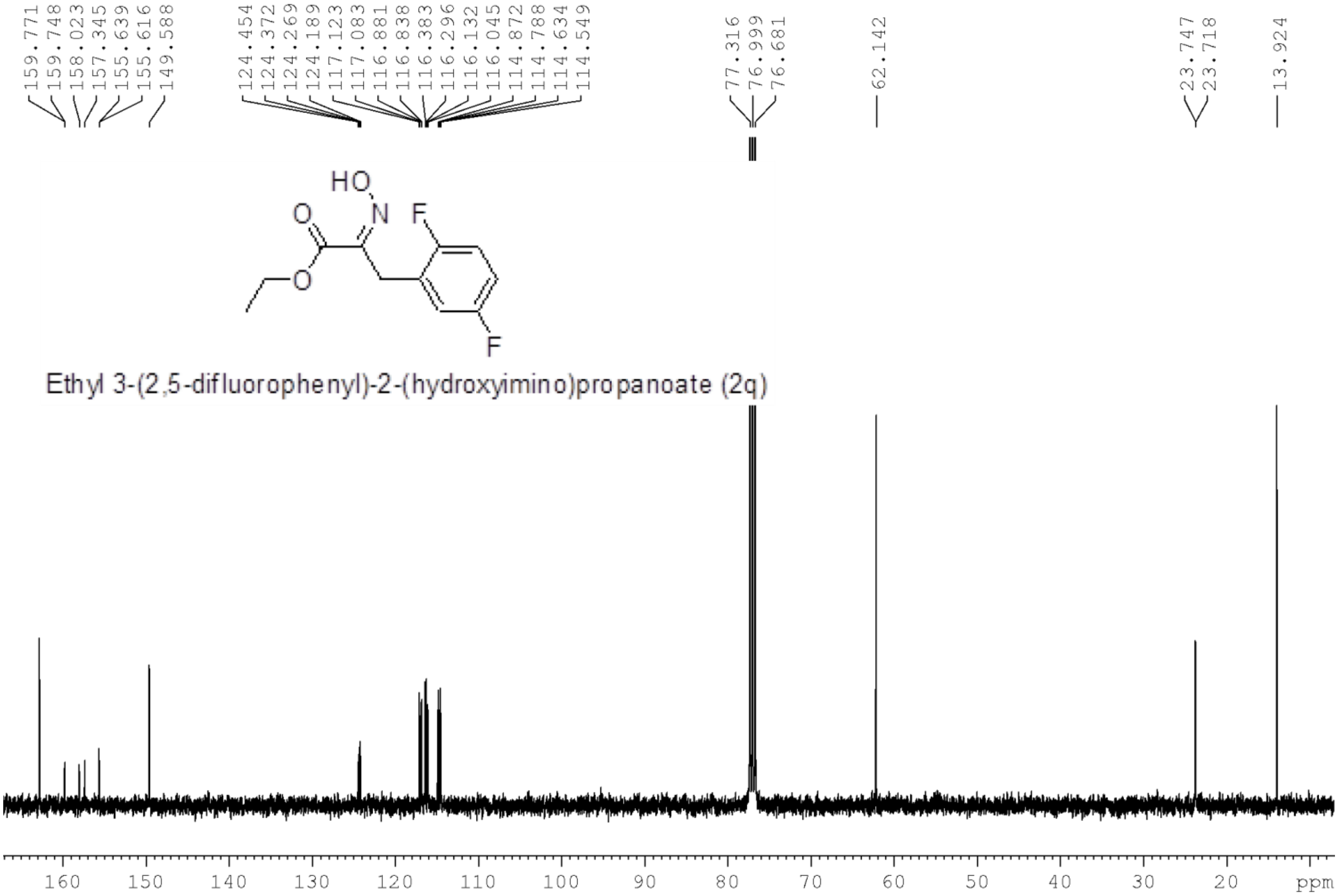

Ethyl 3-(2,5-difluorophenyl)-2-(hydroxyimino)propanoate (2q)

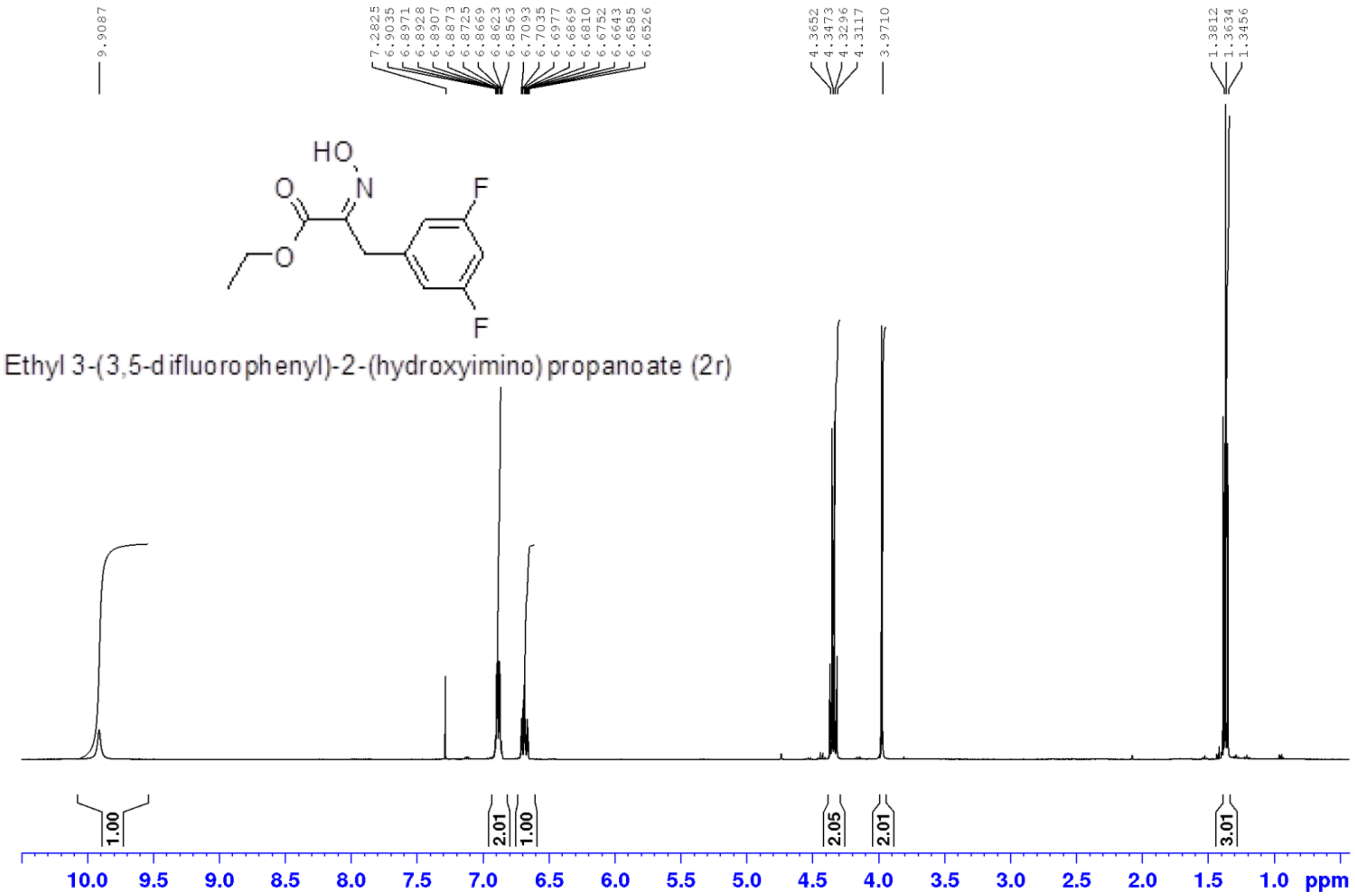
Ethyl 3-(3,5-difluorophenyl)-2-(hydroxyimino)propanoate (2r)
ppm

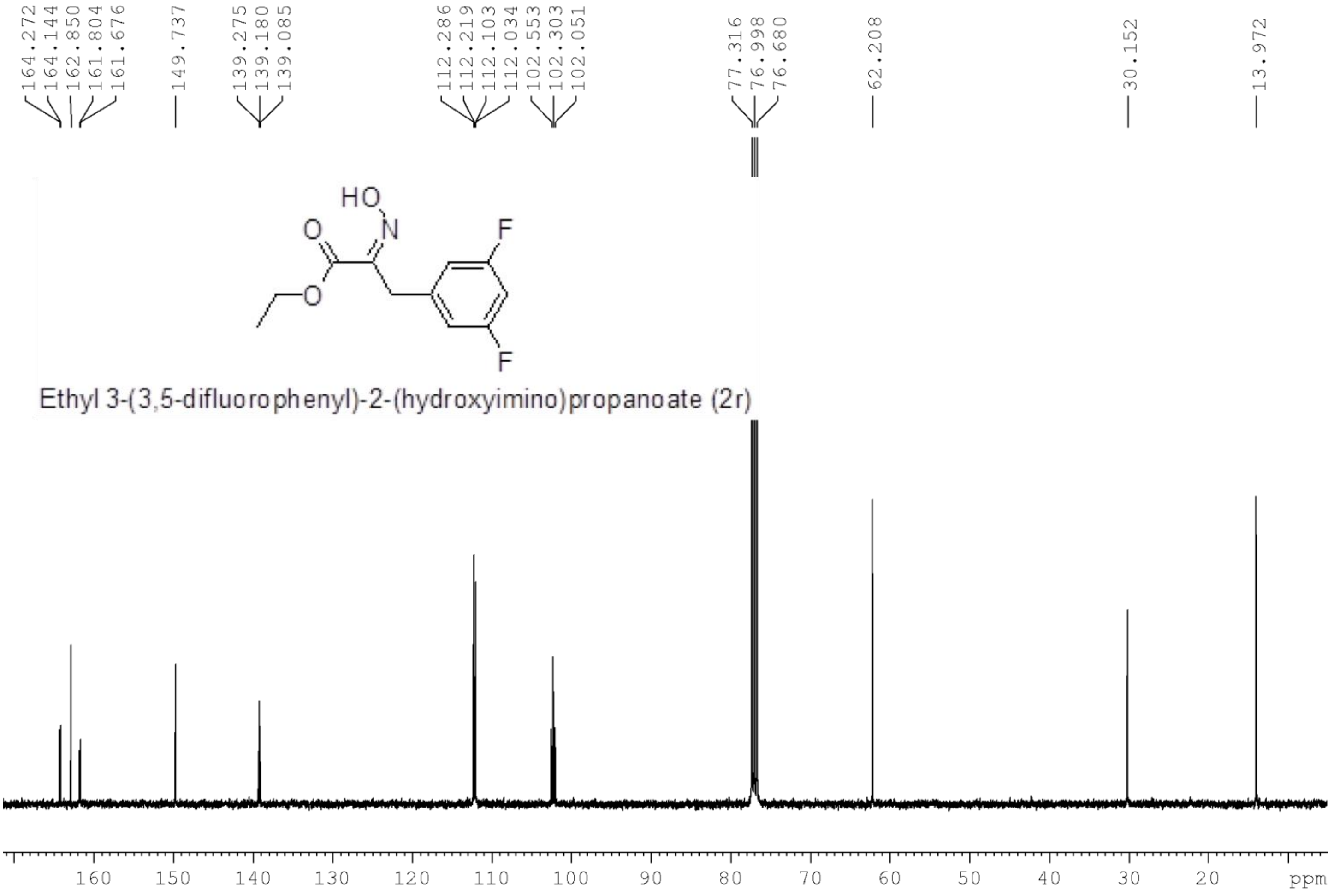
Ethyl 3-(3,5-difluorophenyl)-2-(hydroxyimino)propanoate (2r)
ppm

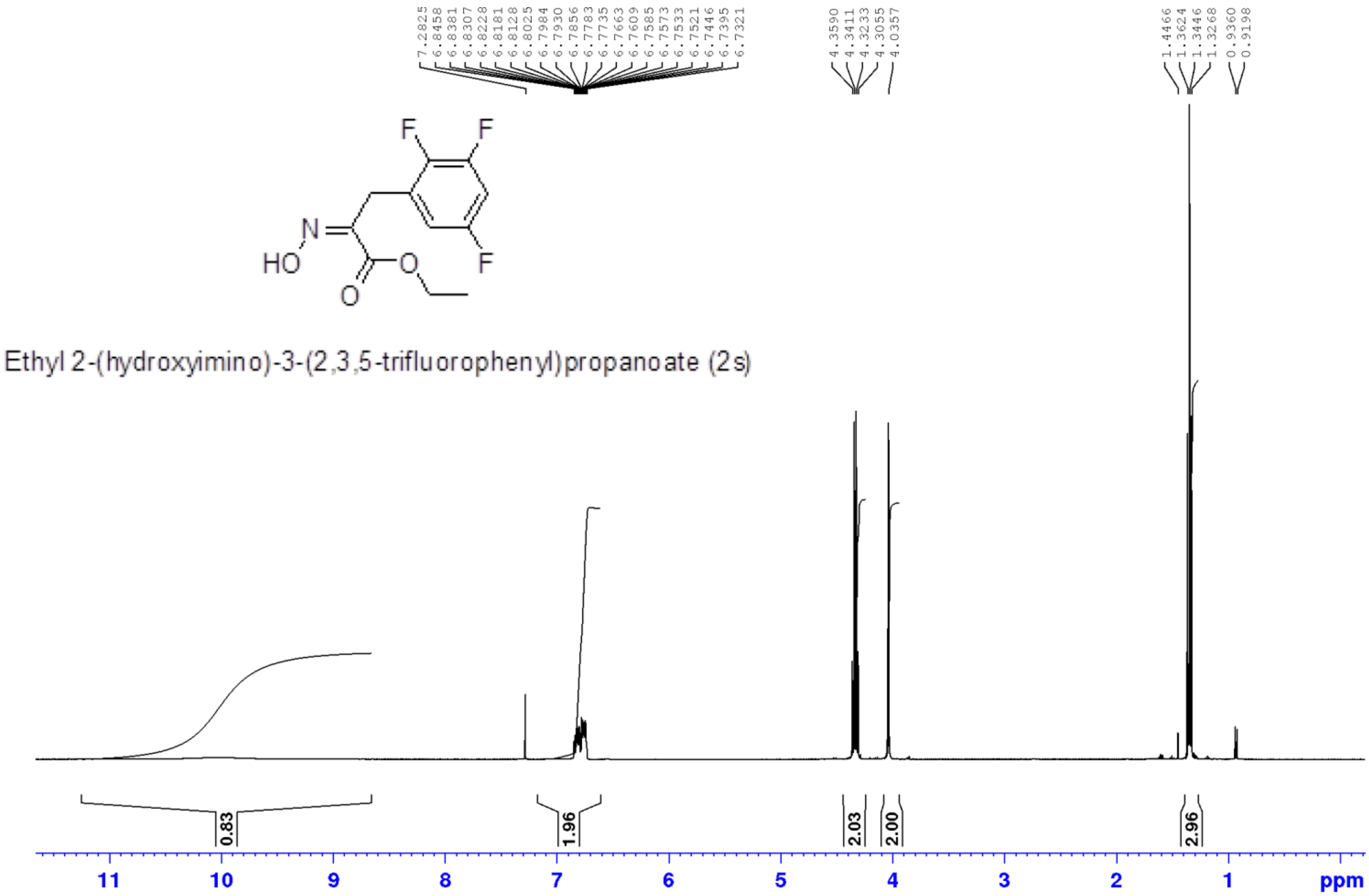

Ethyl 2-(hydroxyimino)-3-(2,3,5-trifluorophenyl)propanoate (2s)
F
F
F
N
HO
O
O
7.2825
6.8458
6.8381
6.8307
6.8228
6.8181
6.8128
6.8025
6.7984
6.7930
6.7856
6.7783
6.7735
6.7663
6.7609
6.7585
6.7573
6.7533
6.7521
6.7446
6.7395
6.7321
4.3590
4.3411
4.3233
4.3055
4.0357
1.4466
1.3624
1.3446
1.3268
0.9360
0.9198
0.83
1.96
2.03
2.00
2.96
11
10
9
8
7
6
5
4
3
2
1
ppm

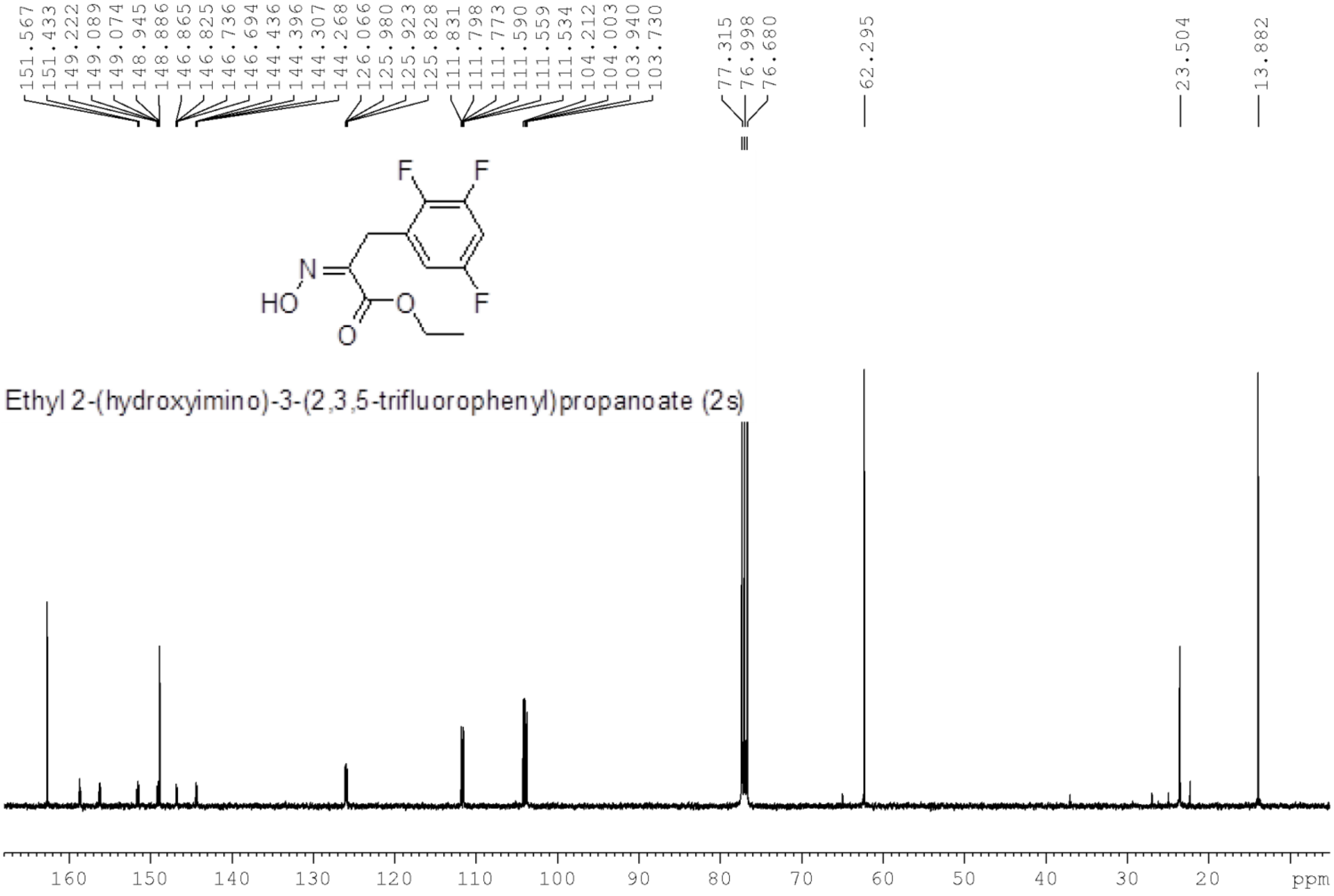

151.567
151.433
149.222
149.089
149.074
148.945
148.886
146.865
146.825
146.736
146.694
144.436
144.396
144.307
144.268
126.066
125.980
125.923
125.828
111.831
111.798
111.773
111.590
111.559
111.534
104.212
104.003
103.940
103.730
77.315
76.998
76.680
62.295
23.504
13.882
F
F
F
N
HO
O
O
Ethyl 2-(hydroxyimino)-3-(2,3,5-trifluorophenyl)propanoate (2s)
160
150
140
130
120
110
100
90
80
70
60
50
40
30
20
ppm

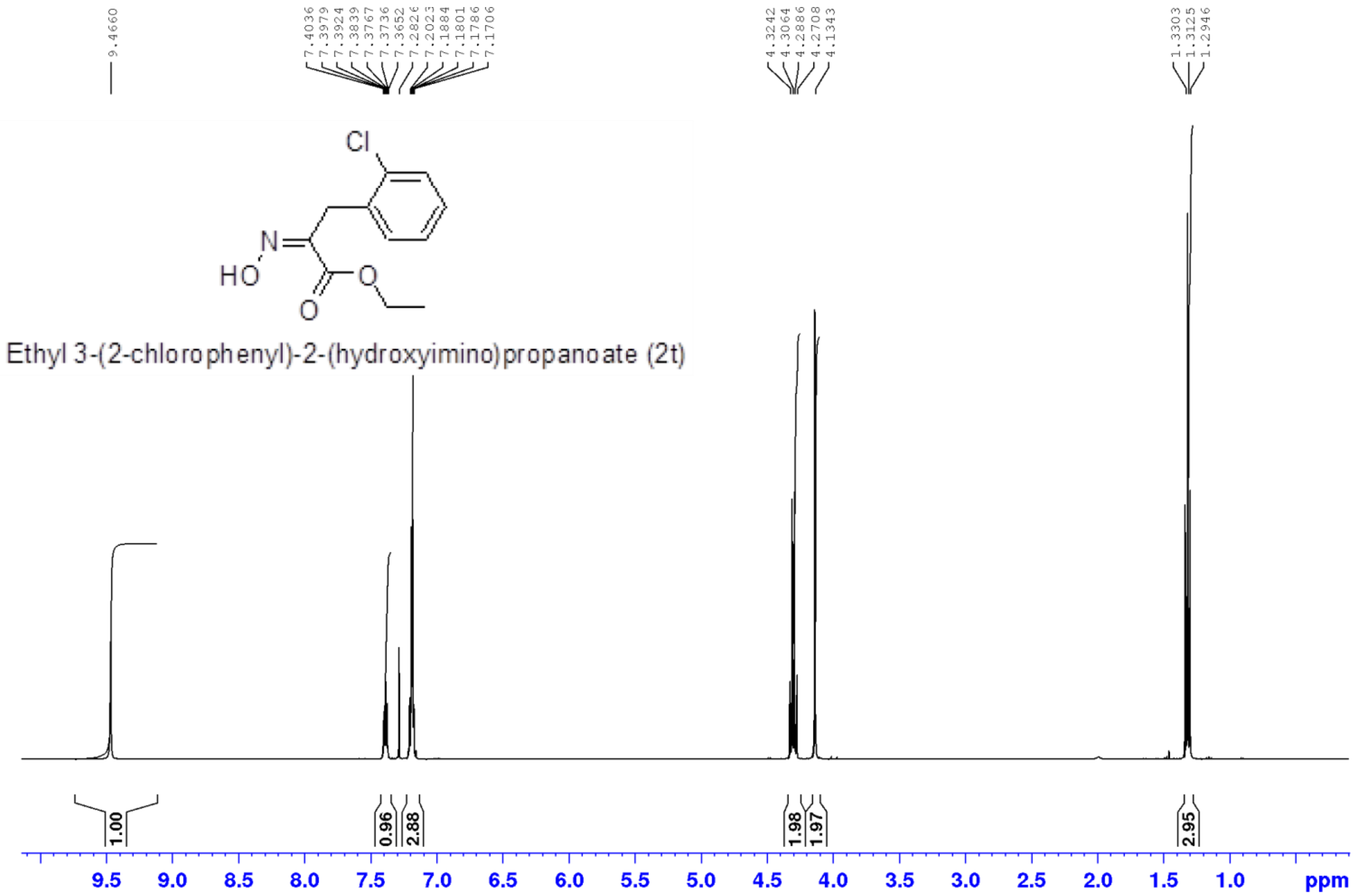
9.4660
7.4036
7.3979
7.3924
7.3839
7.3767
7.3736
7.3652
7.2826
7.2023
7.1884
7.1801
7.1786
7.1706
4.3242
4.3064
4.2886
4.2708
4.1343
1.3303
1.3125
1.2946
Cl
N
HO
O
O
Ethyl 3-(2-chlorophenyl)-2-(hydroxyimino)propanoate (2t)
1.00
0.96
2.88
1.98
1.97
2.95
9.5
9.0
8.5
8.0
7.5
7.0
6.5
6.0
5.5
5.0
4.5
4.0
3.5
3.0
2.5
2.0
1.5
1.0
ppm

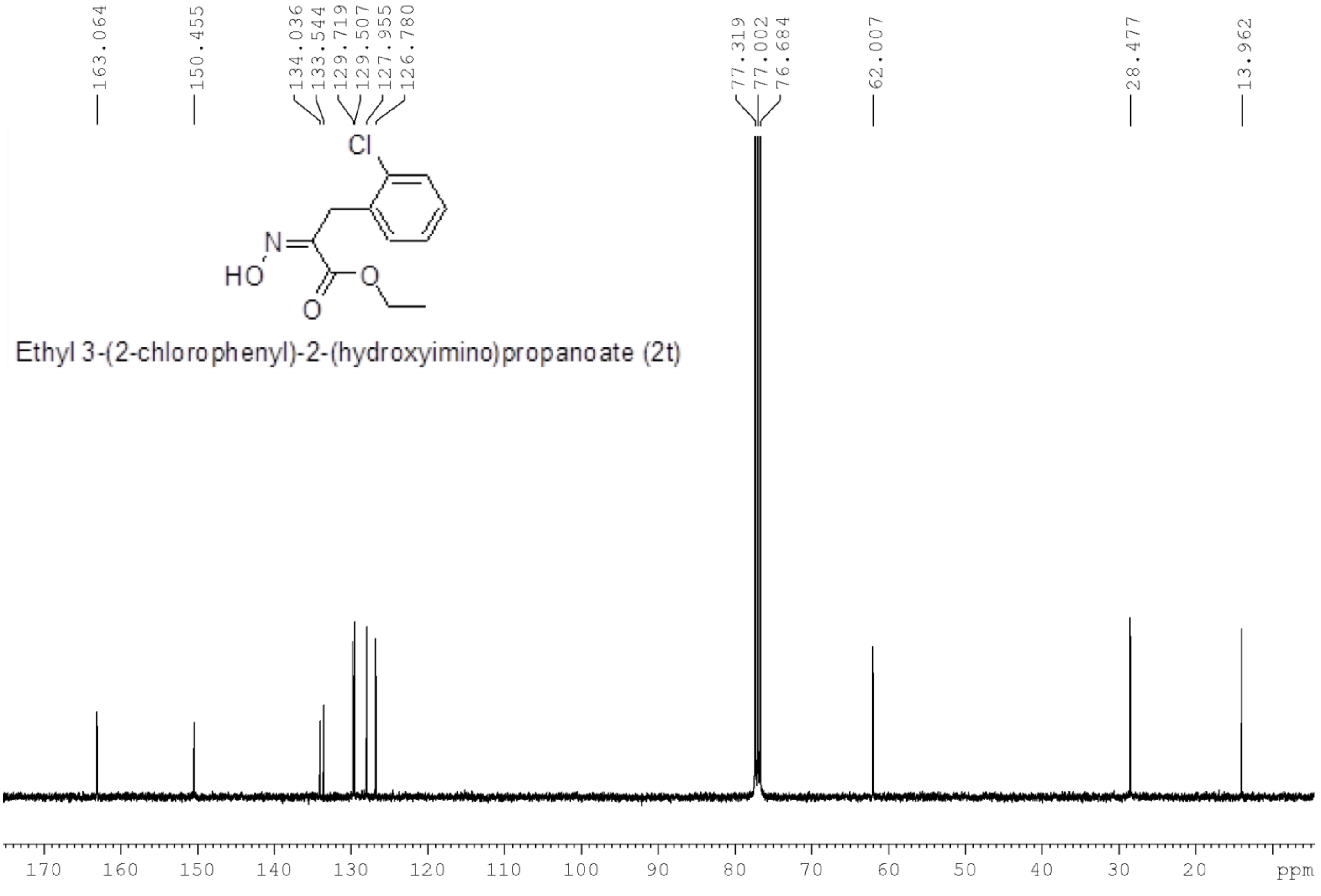
163.064
150.455
134.036
133.544
129.719
129.507
127.955
126.780
77.319
77.002
76.684
62.007
28.477
13.962
Cl
N
HO
O
O
Ethyl 3-(2-chlorophenyl)-2-(hydroxyimino)propanoate (2t)
170
160
150
140
130
120
110
100
90
80
70
60
50
40
30
20
ppm

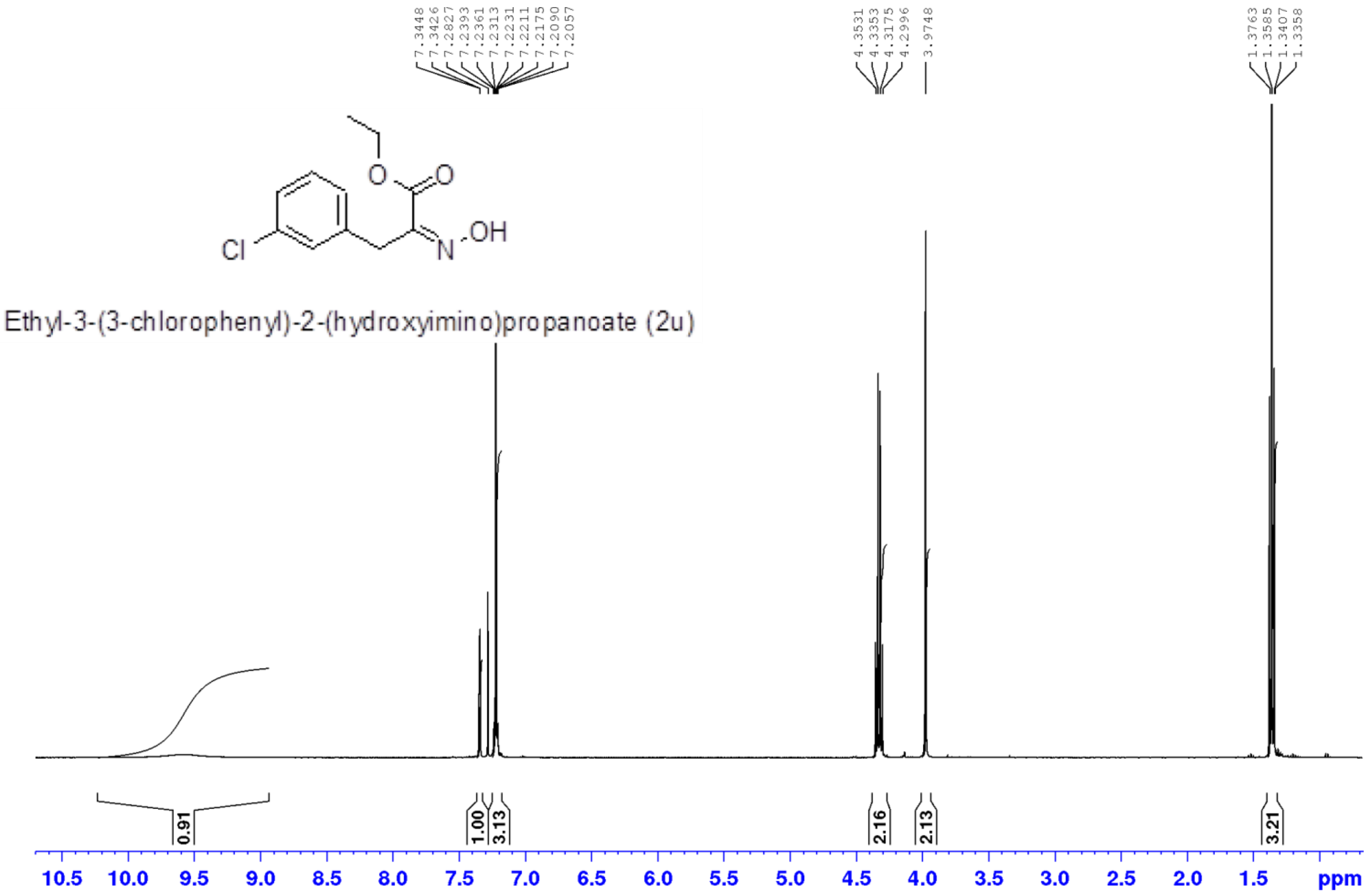

7.3448
7.3426
7.2827
7.2393
7.2361
7.2313
7.2231
7.2211
7.2175
7.2090
7.2057
4.3531
4.3353
4.3175
4.2996
3.9748
1.3763
1.3585
1.3407
1.3358
Cl
O
O
N
OH
Ethyl-3-(3-chlorophenyl)-2-(hydroxyimino)propanoate (2u)
0.91
1.00
3.13
2.16
2.13
3.21
10.5 10.0 9.5 9.0 8.5 8.0 7.5 7.0 6.5 6.0 5.5 5.0 4.5 4.0 3.5 3.0 2.5 2.0 1.5 ppm

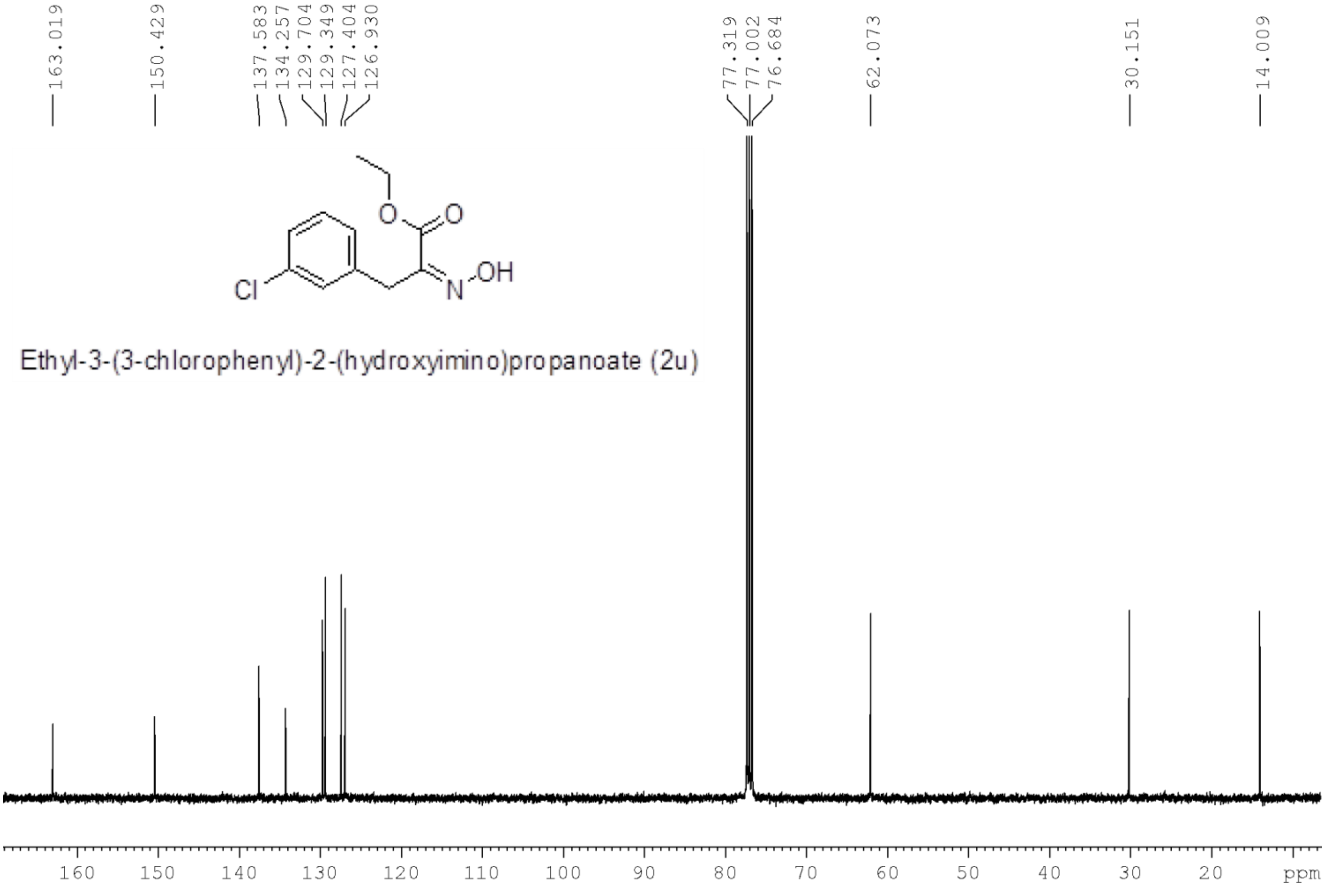

163.019
150.429
137.583
134.257
129.704
129.349
127.404
126.930
77.319
77.002
76.684
62.073
30.151
14.009
Cl
O
O
N
OH
Ethyl-3-(3-chlorophenyl)-2-(hydroxyimino)propanoate (2u)
160 150 140 130 120 110 100 90 80 70 60 50 40 30 20 ppm

^{1}H NMR of a new and an old (15 days) sample of **6u** (with ethanol), the second one (concentrated to dryness) is also containing the ethanol adduct **7** :

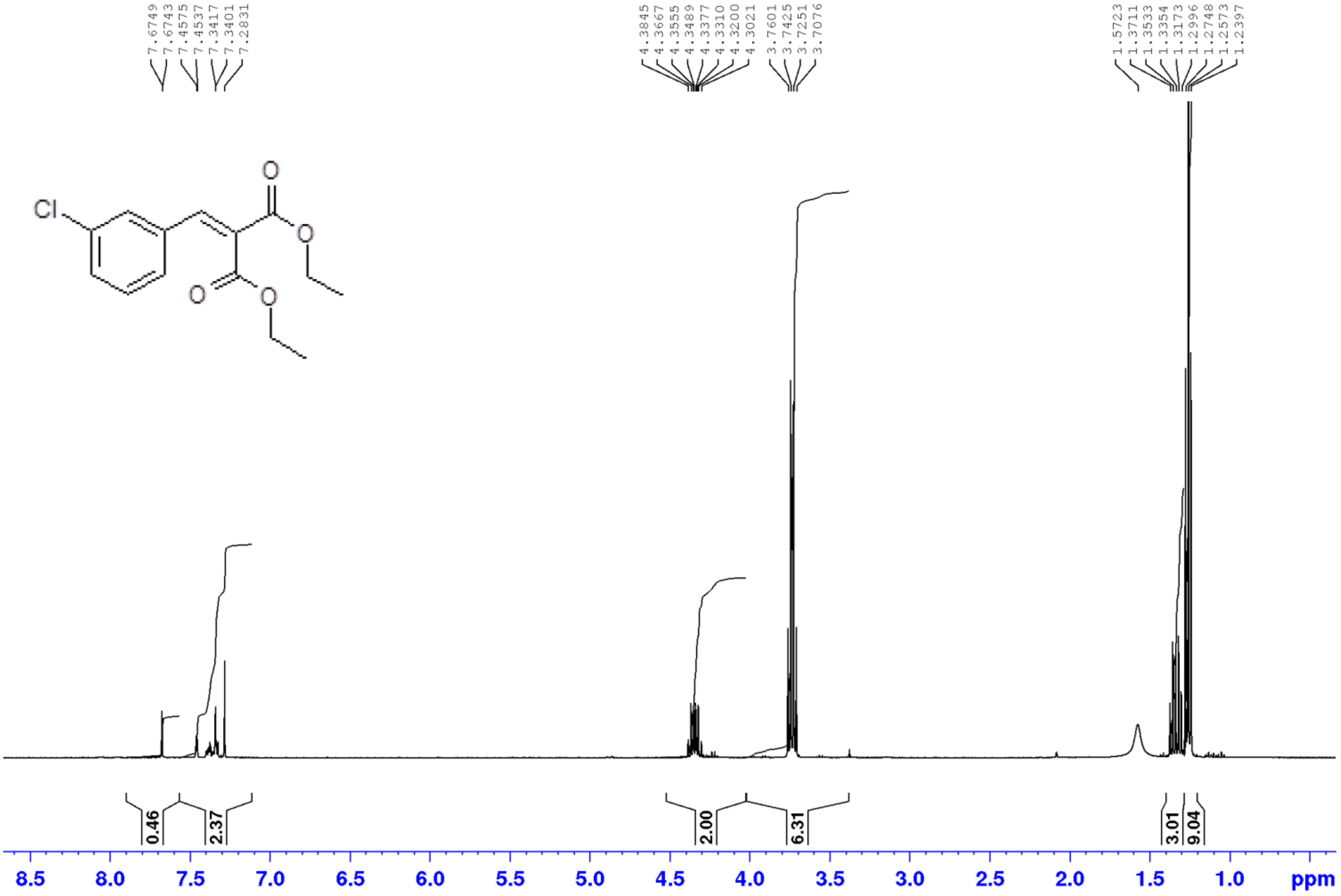

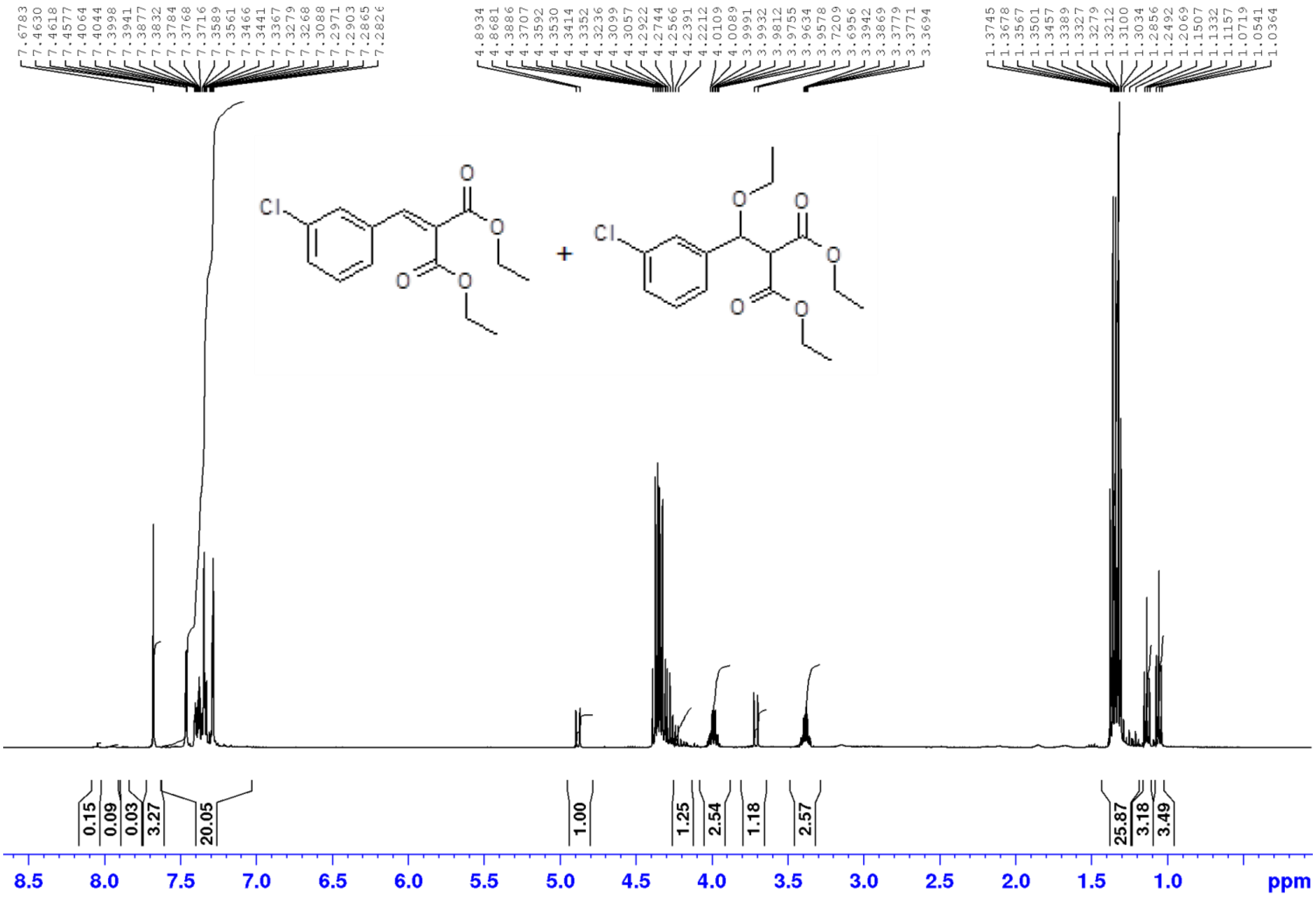

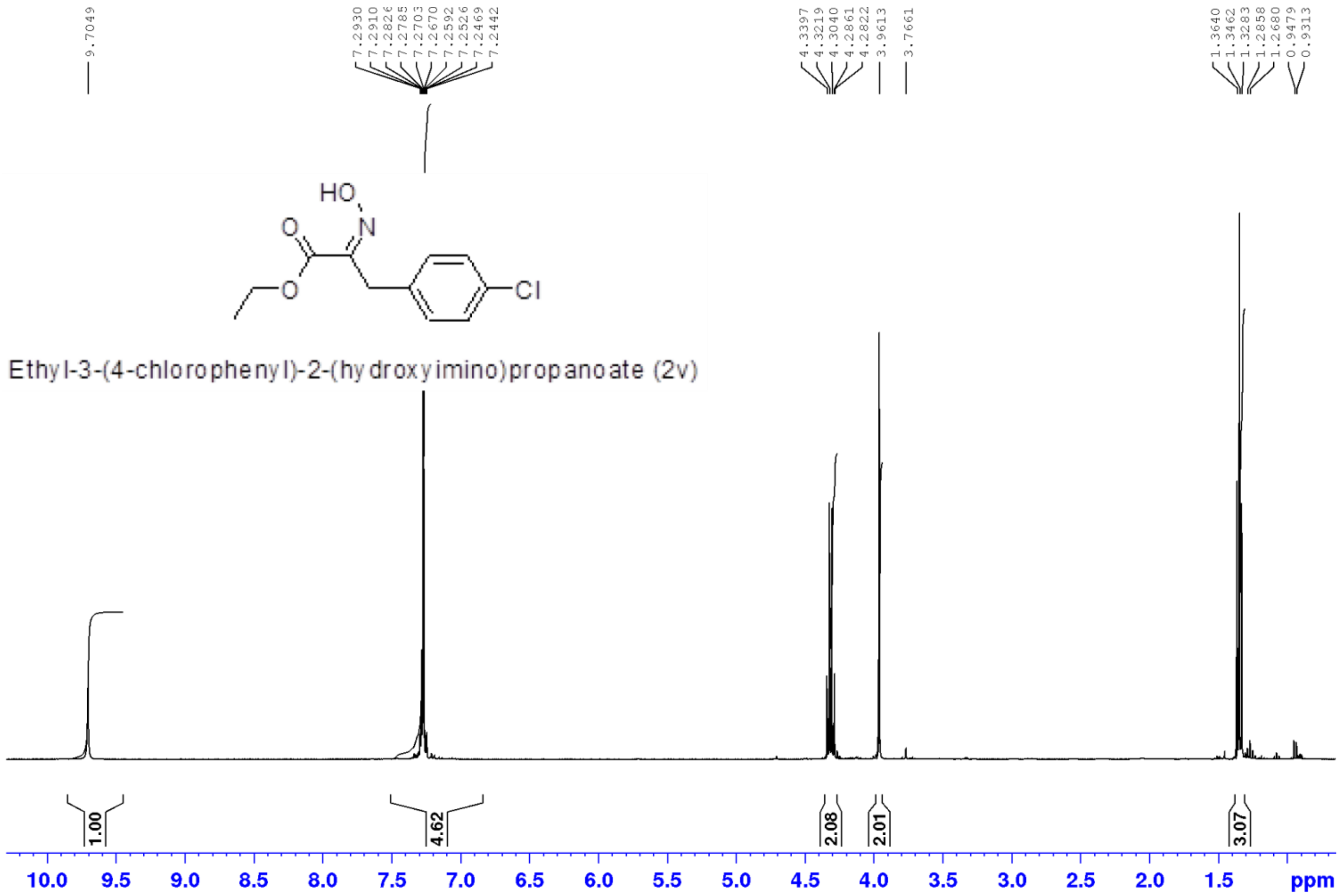
9.7049
7.2930
7.2910
7.2826
7.2785
7.2703
7.2670
7.2592
7.2526
7.2469
7.2442
4.3397
4.3219
4.3040
4.2861
4.2822
3.9613
3.7661
1.3640
1.3462
1.3283
1.2858
1.2680
0.9479
0.9313
HO
N
O
O
Cl
Ethyl-3-(4-chlorophenyl)-2-(hydroxyimino)propanoate (2v)
1.00
4.62
2.08
2.01
3.07
10.0 9.5 9.0 8.5 8.0 7.5 7.0 6.5 6.0 5.5 5.0 4.5 4.0 3.5 3.0 2.5 2.0 1.5 ppm

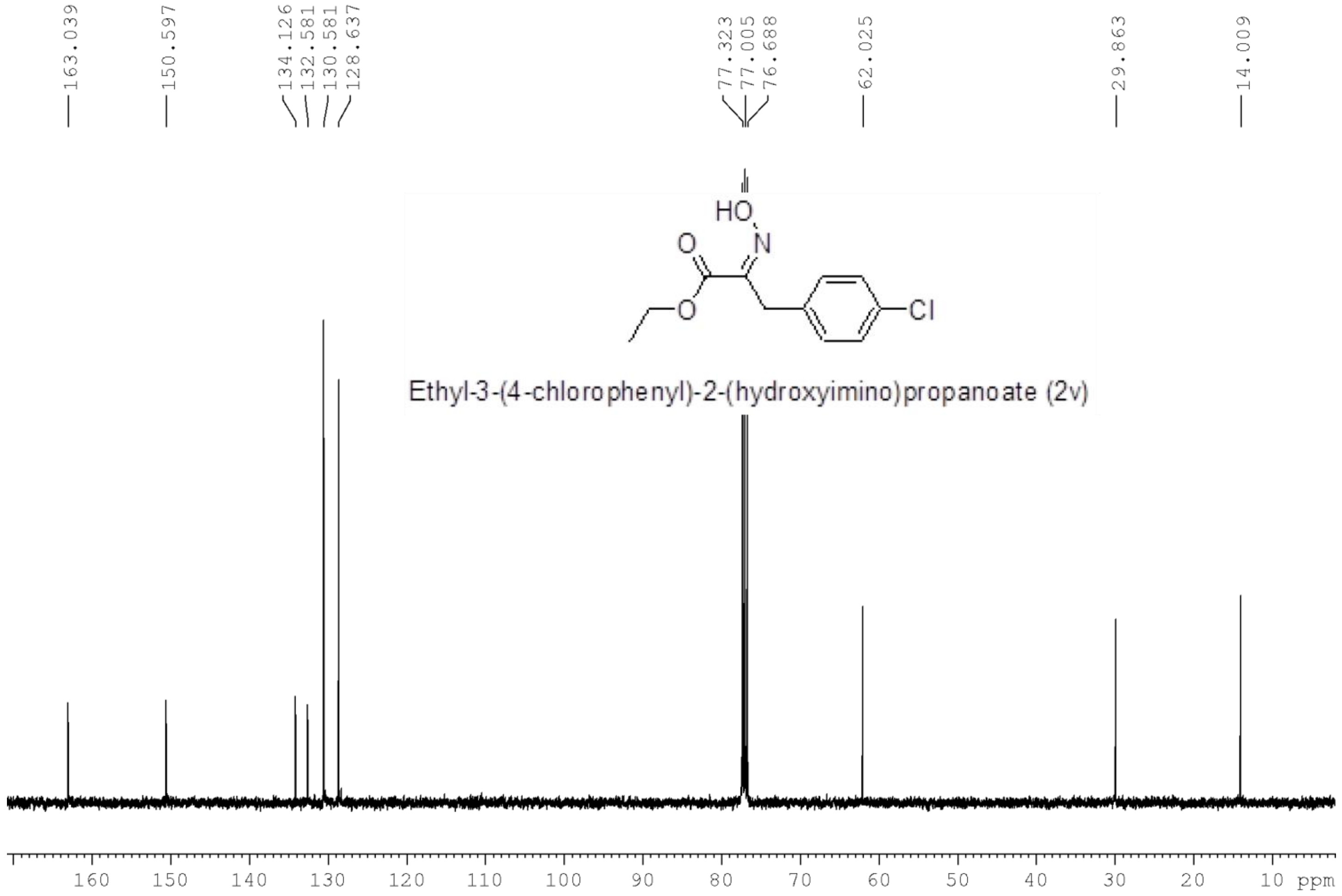
163.039
150.597
134.126
132.581
130.581
128.637
77.323
77.005
76.688
62.025
29.863
14.009
HO
N
O
O
Cl
Ethyl-3-(4-chlorophenyl)-2-(hydroxyimino)propanoate (2v)
160 150 140 130 120 110 100 90 80 70 60 50 40 30 20 10 ppm

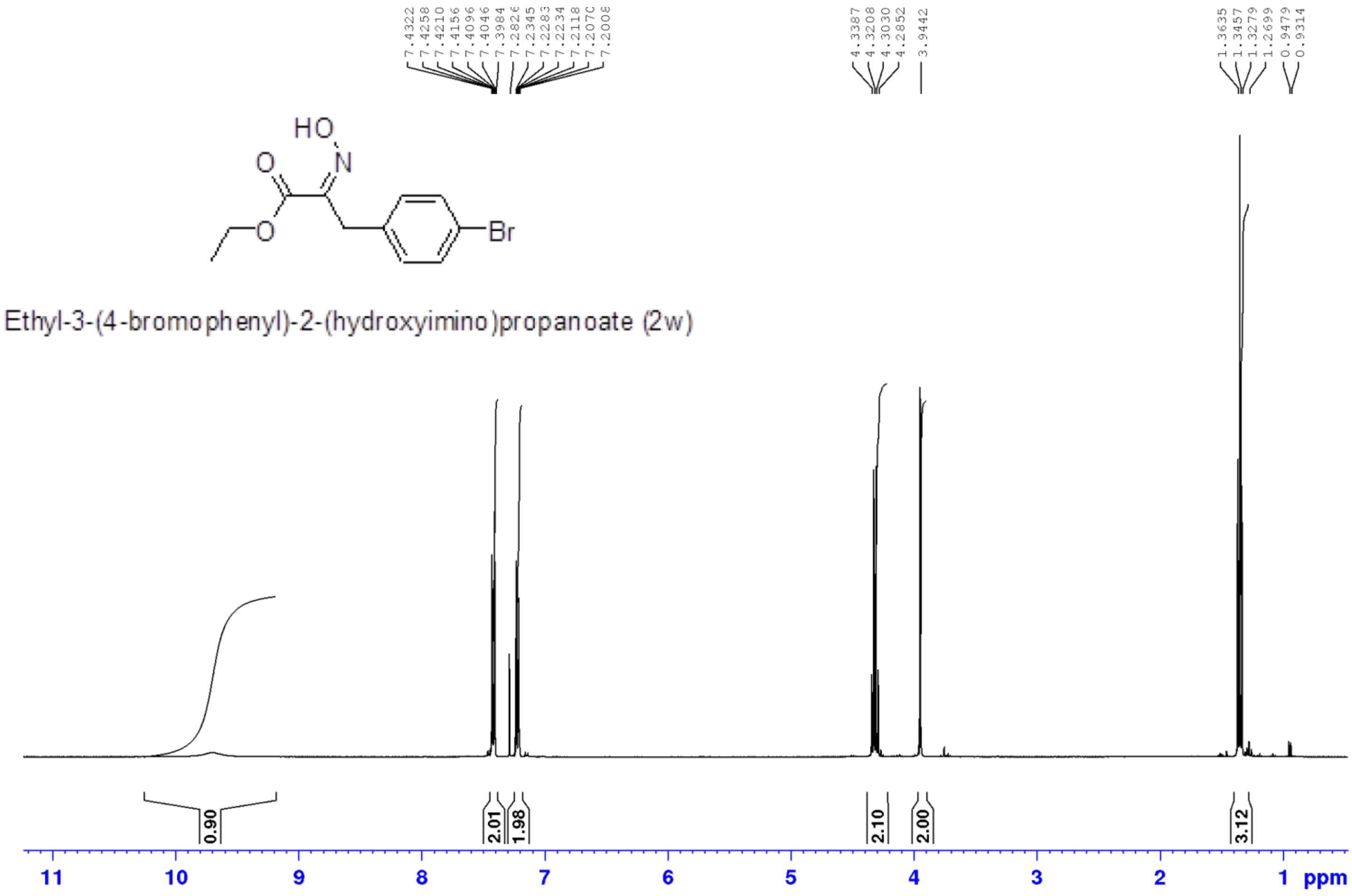

Ethyl-3-(4-bromophenyl)-2-(hydroxyimino)propanoate (2w)

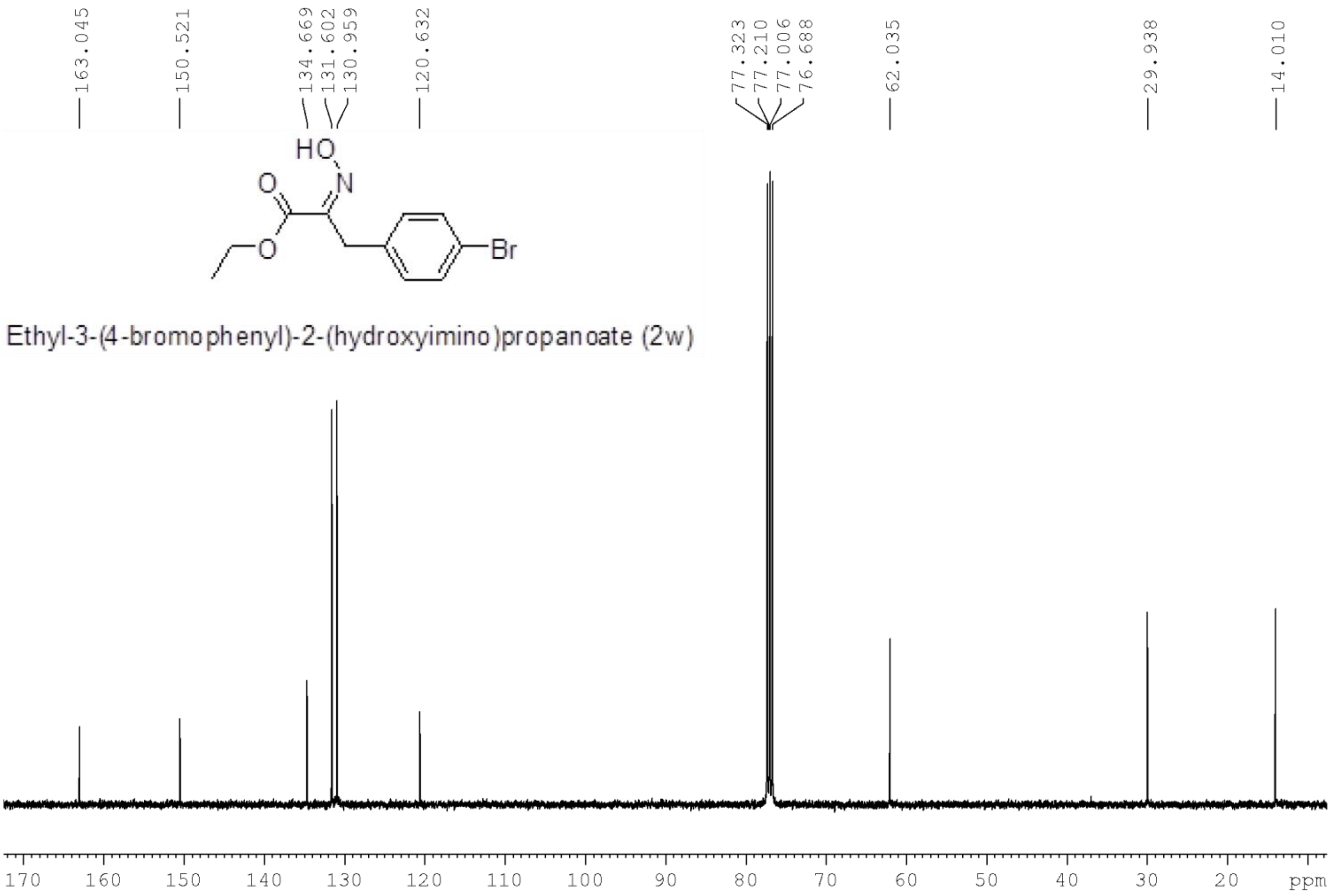

Ethyl-3-(4-bromophenyl)-2-(hydroxyimino)propanoate (2w)

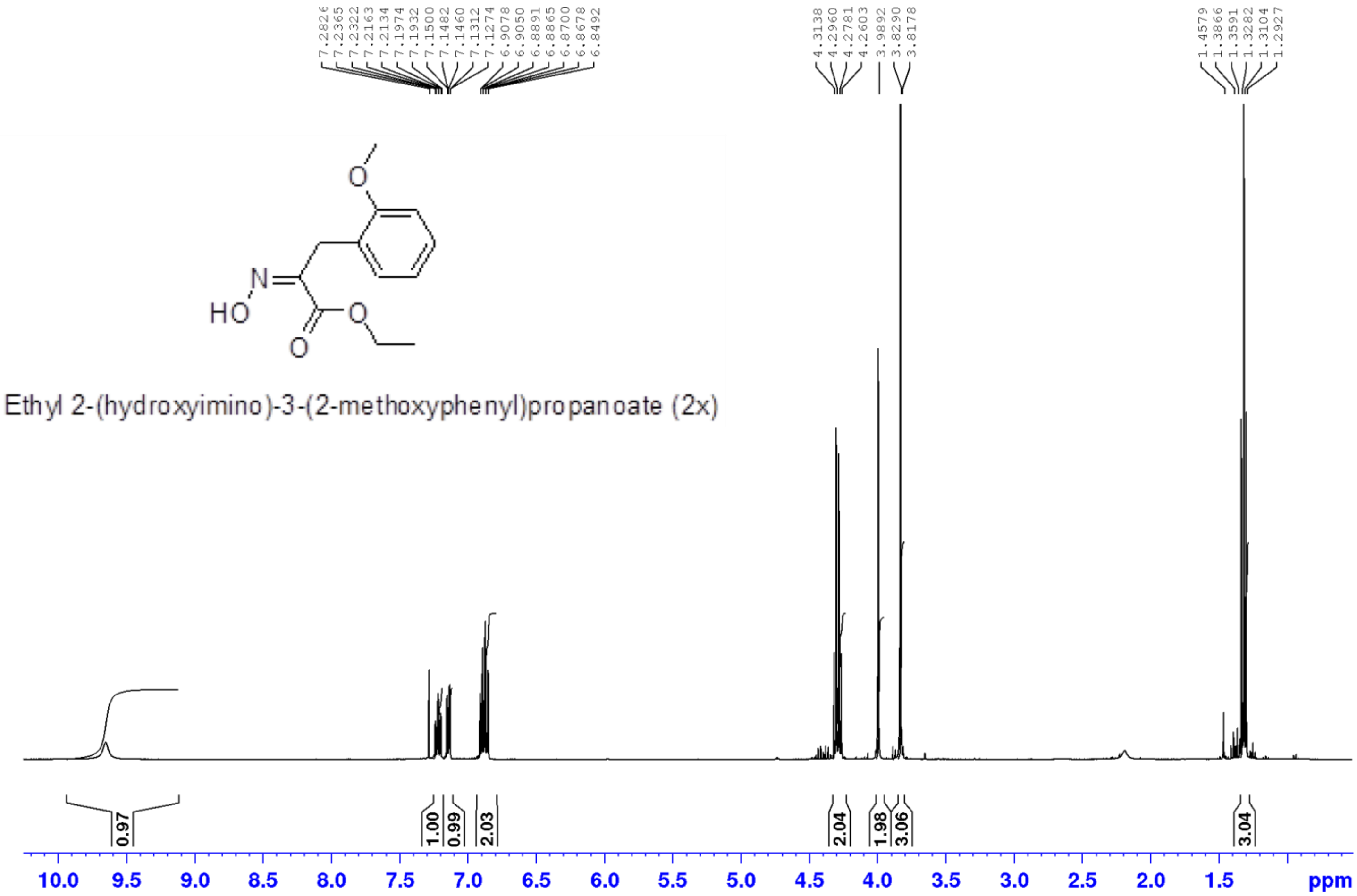
7.2826
7.2365
7.2322
7.2163
7.2134
7.1974
7.1932
7.1500
7.1482
7.1460
7.1312
7.1274
6.9078
6.9050
6.8891
6.8865
6.8700
6.8678
6.8492
4.3138
4.2960
4.2781
4.2603
3.9892
3.8290
3.8178
1.4579
1.3866
1.3591
1.3282
1.3104
1.2927
Ethyl 2-(hydroxyimino)-3-(2-methoxyphenyl)propanoate (2x)
0.97
1.00
0.99
2.03
2.04
1.98
3.06
3.04
10.0 9.5 9.0 8.5 8.0 7.5 7.0 6.5 6.0 5.5 5.0 4.5 4.0 3.5 3.0 2.5 2.0 1.5 ppm

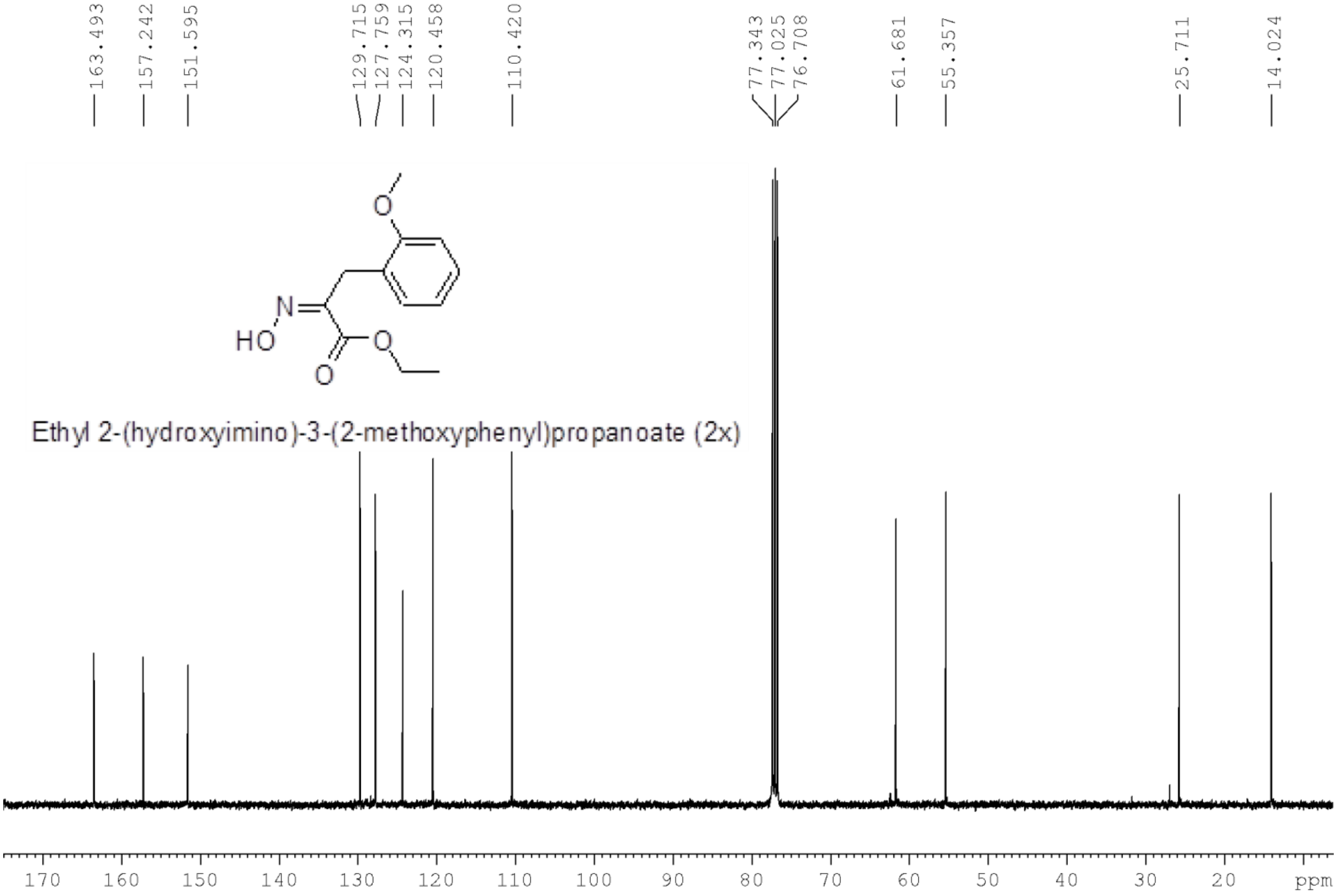
163.493
157.242
151.595
129.715
127.759
124.315
120.458
110.420
77.343
77.025
76.708
61.681
55.357
25.711
14.024
Ethyl 2-(hydroxyimino)-3-(2-methoxyphenyl)propanoate (2x)
170 160 150 140 130 120 110 100 90 80 70 60 50 40 30 20 ppm

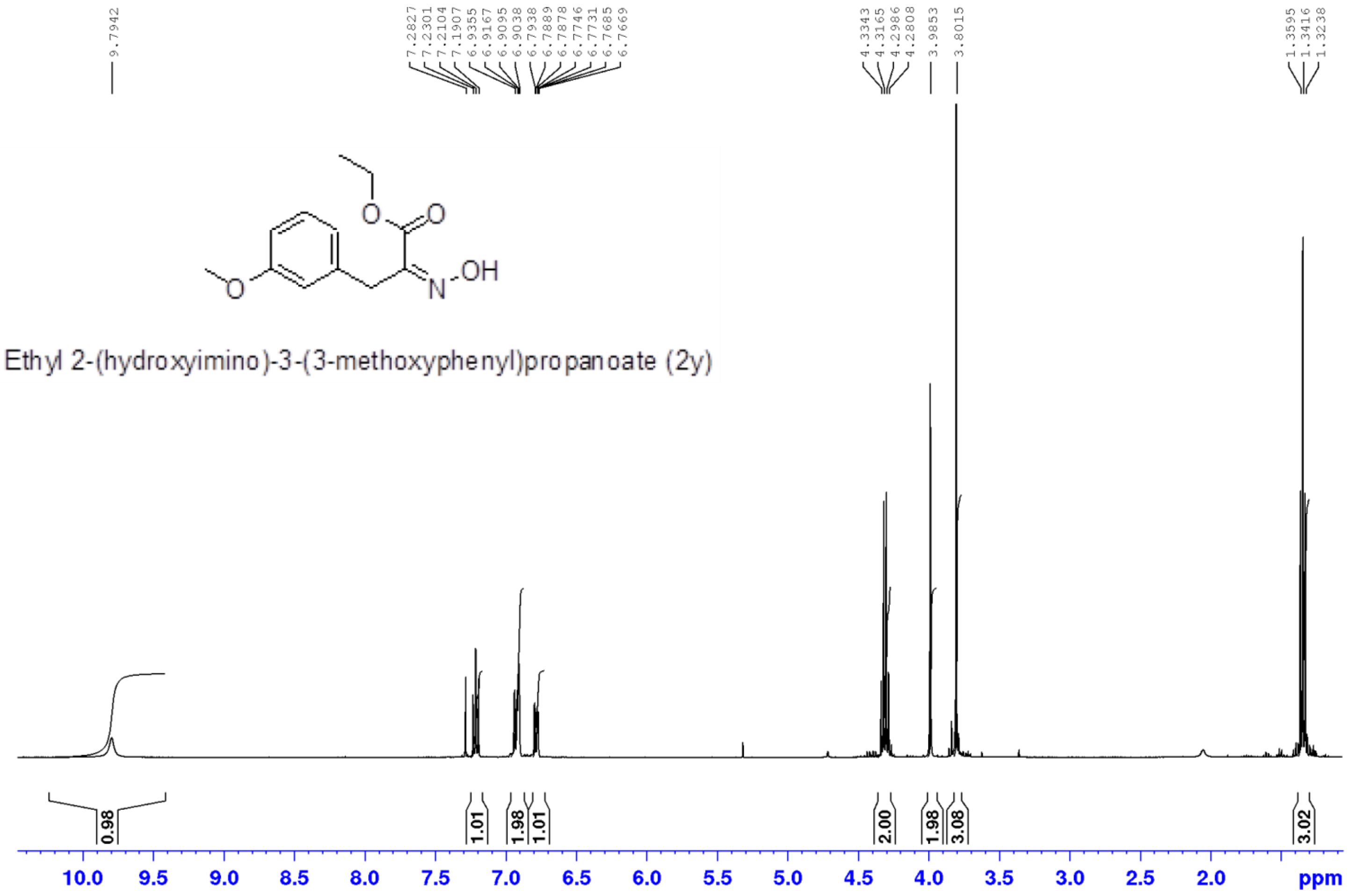
9.7942
7.2827 7.2301 7.2104 7.1907 6.9355 6.9167 6.9095 6.9038 6.7938 6.7889 6.7878 6.7746 6.7731 6.7685 6.7669
4.3343 4.3165 4.2986 4.2808 3.9853 3.8015
1.3595 1.3416 1.3238
O
O
N
OH
O
Ethyl 2-(hydroxyimino)-3-(3-methoxyphenyl)propanoate (2y)
0.98
1.01
1.98
1.01
2.00
1.98
3.08
3.02
10.0 9.5 9.0 8.5 8.0 7.5 7.0 6.5 6.0 5.5 5.0 4.5 4.0 3.5 3.0 2.5 2.0 ppm

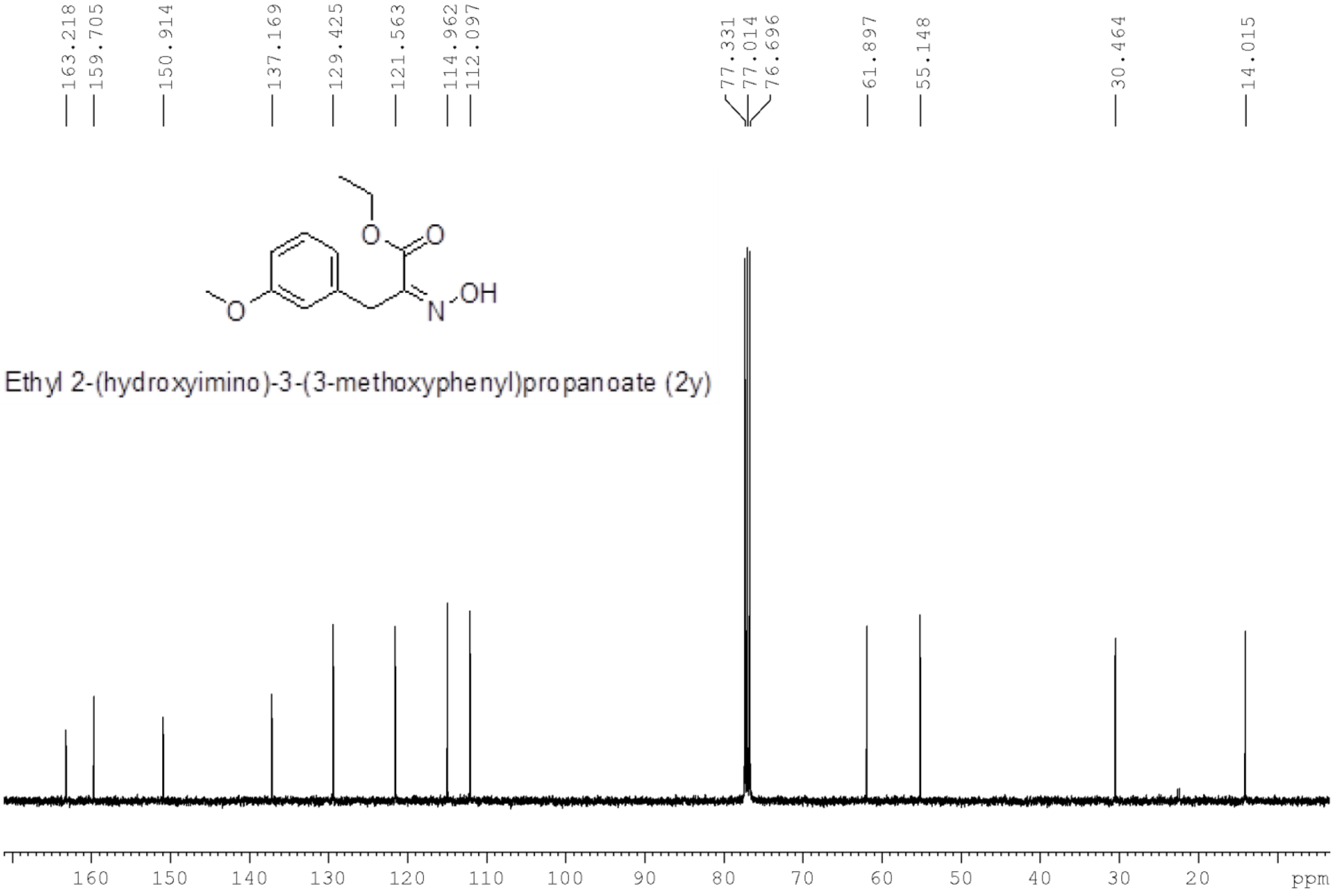
163.218 159.705 150.914 137.169 129.425 121.563 114.962 112.097
77.331 77.014 76.696
61.897 55.148 30.464 14.015
O
O
N
OH
O
Ethyl 2-(hydroxyimino)-3-(3-methoxyphenyl)propanoate (2y)
160 150 140 130 120 110 100 90 80 70 60 50 40 30 20 ppm

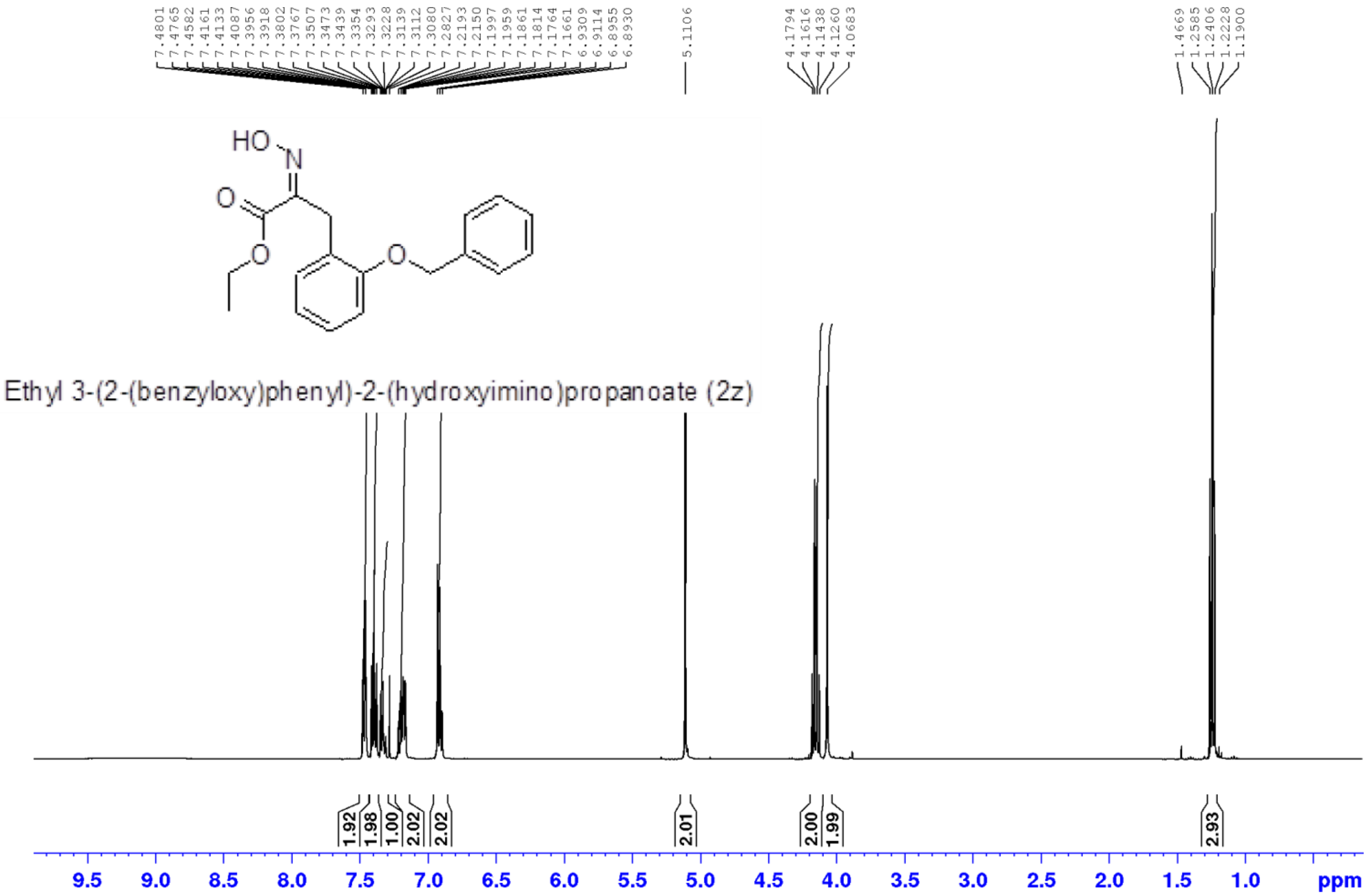
Ethyl 3-(2-(benzyloxy)phenyl)-2-(hydroxyimino)propanoate (2z)
7.4801 7.4765 7.4582 7.4161 7.4133 7.4087 7.3956 7.3918 7.3802 7.3767 7.3507 7.3473 7.3439 7.3354 7.3293 7.3228 7.3139 7.3112 7.3080 7.2827 7.2193 7.2150 7.1997 7.1959 7.1861 7.1814 7.1764 7.1661 6.9309 6.9114 6.8955 6.8930
5.1106
4.1794 4.1616 4.1438 4.1260 4.0683
1.4669 1.2585 1.2406 1.2228 1.1900
1.92 1.98 1.00 2.02 2.02 2.01 2.00 1.99 2.93
ppm

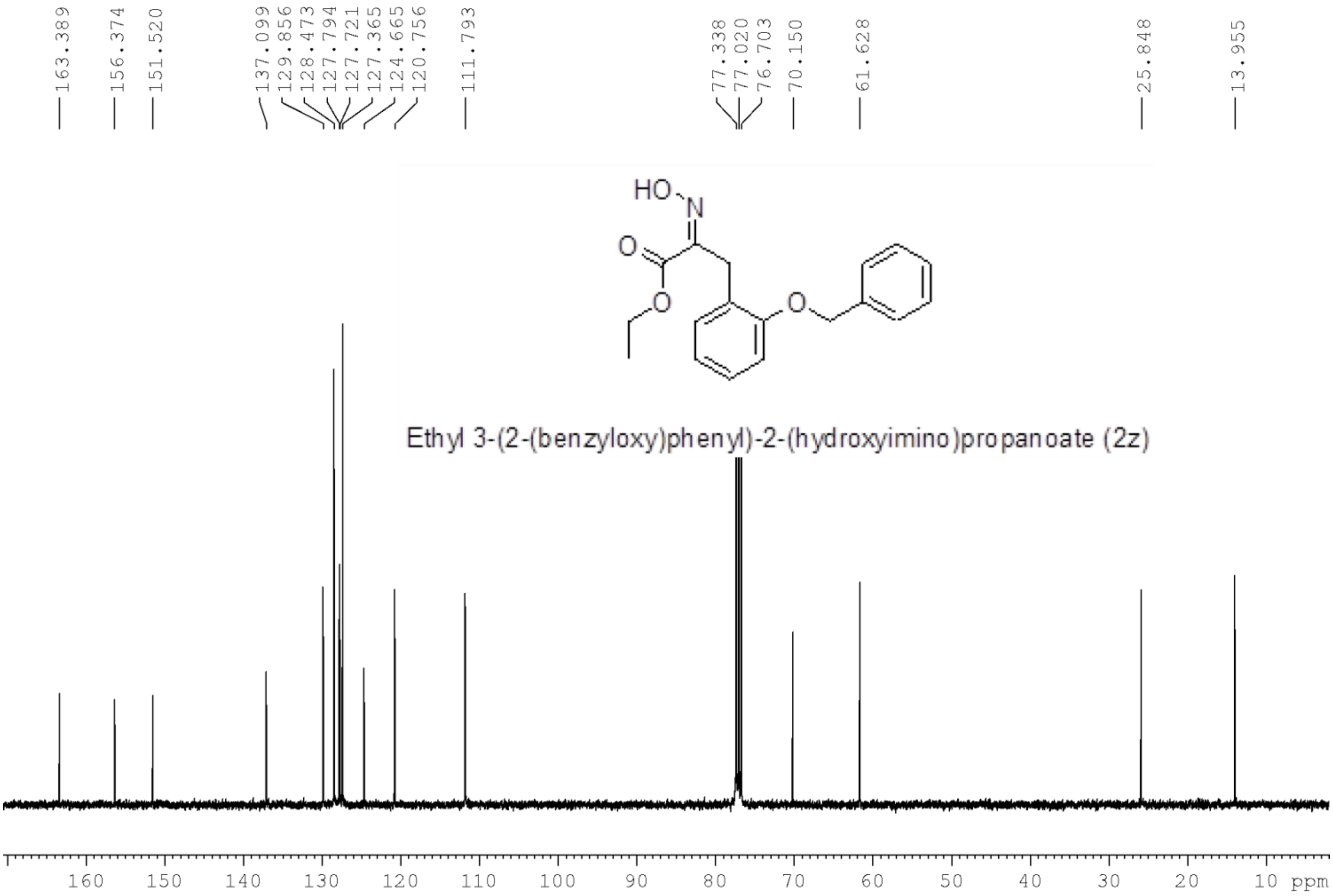
Ethyl 3-(2-(benzyloxy)phenyl)-2-(hydroxyimino)propanoate (2z)
163.389 156.374 151.520 137.099 129.856 128.473 127.794 127.721 127.365 124.665 120.756 111.793 77.338 77.020 76.703 70.150 61.628 25.848 13.955
ppm

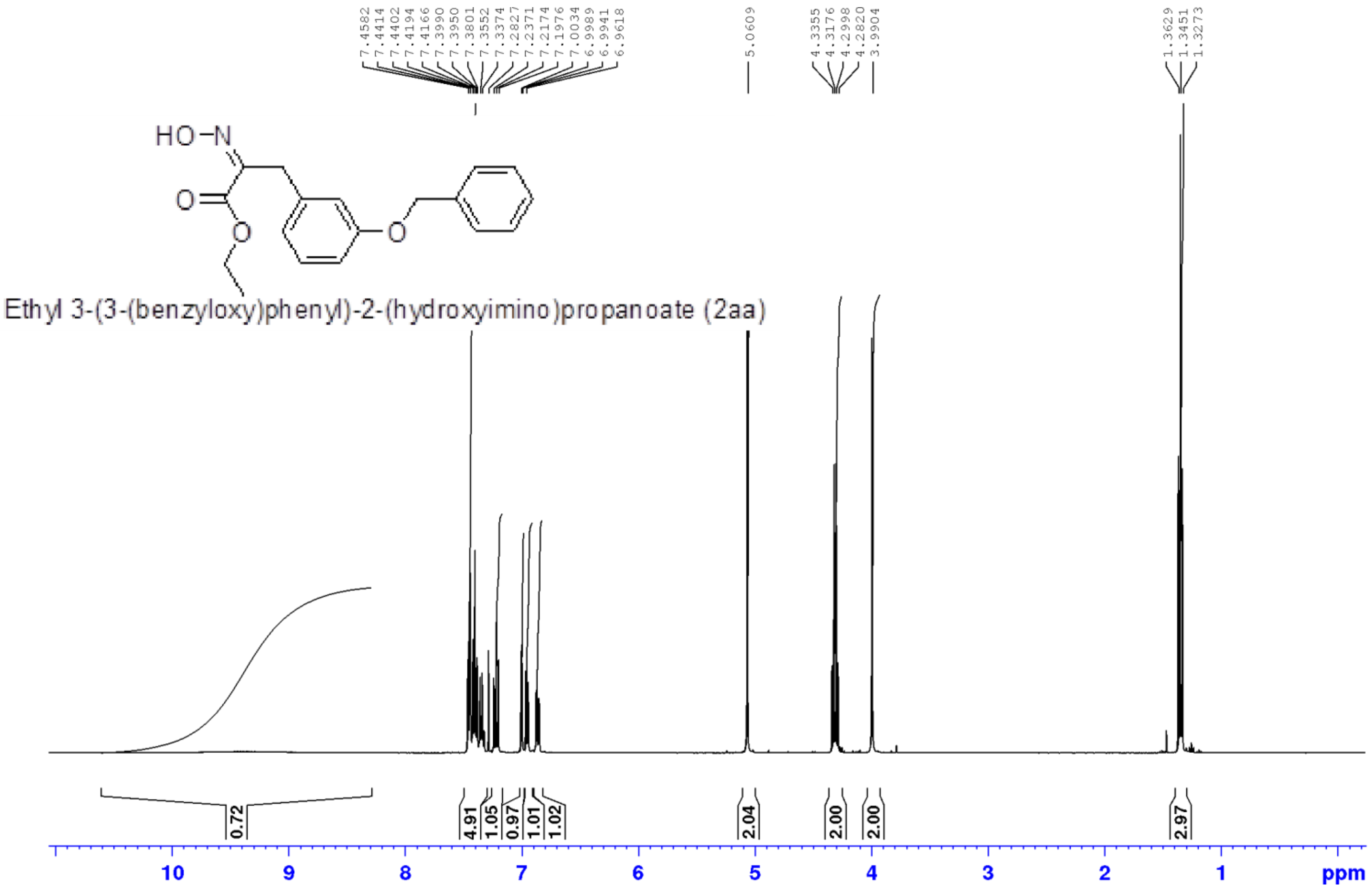
7.4582
7.4414
7.4402
7.4194
7.4166
7.3990
7.3950
7.3801
7.3552
7.3374
7.2827
7.2371
7.2174
7.1976
7.0034
6.9989
6.9941
6.9618
5.0609
4.3355
4.3176
4.2998
4.2820
3.9904
1.3629
1.3451
1.3273
HO−N
O
O
O
Ethyl 3-(3-(benzyloxy)phenyl)-2-(hydroxyimino)propanoate (2aa)
0.72
4.91
1.05
0.97
1.01
1.02
2.04
2.00
2.00
2.97
10
9
8
7
6
5
4
3
2
1
ppm

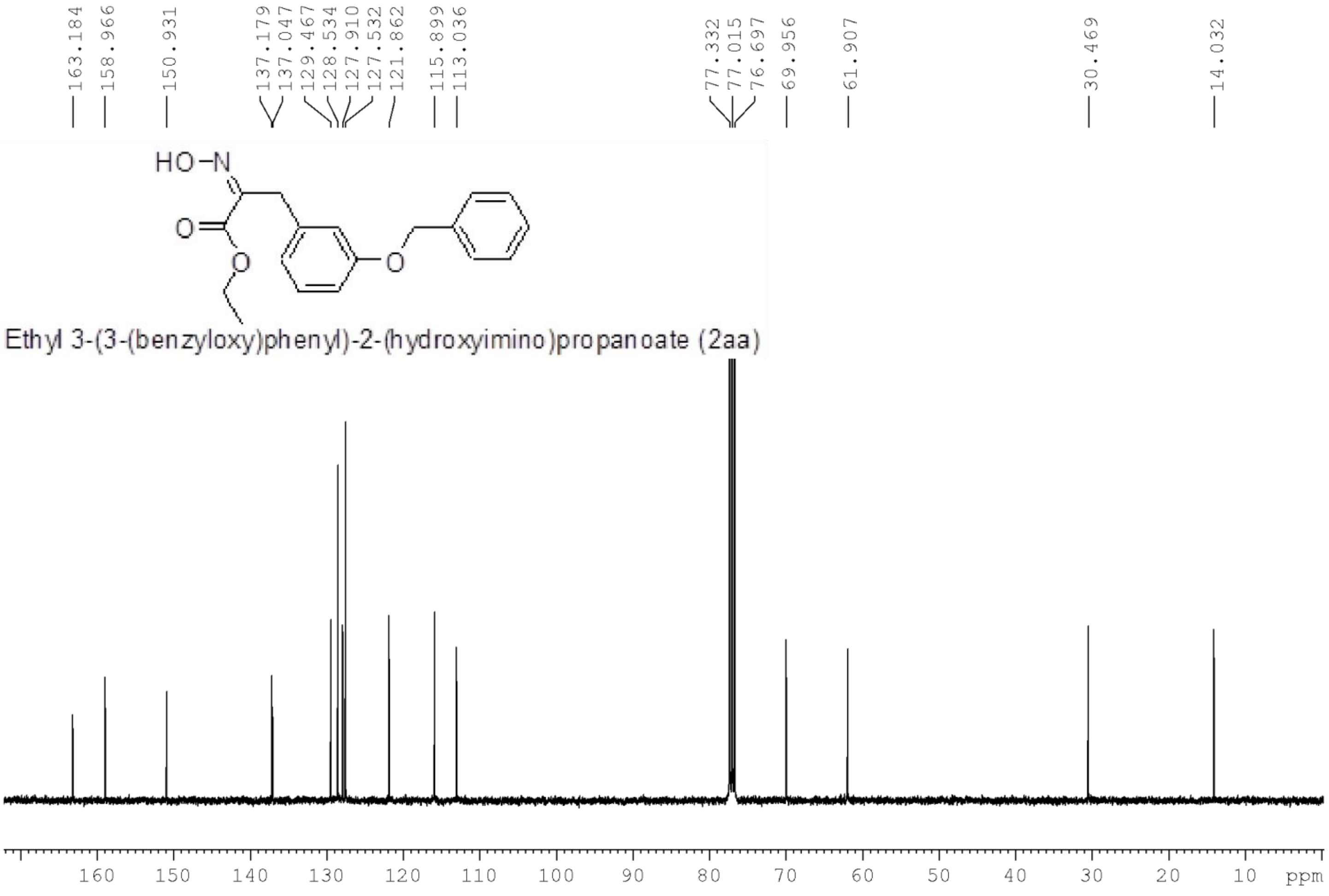
163.184
158.966
150.931
137.179
137.047
129.467
128.534
127.910
127.532
121.862
115.899
113.036
77.332
77.015
76.697
69.956
61.907
30.469
14.032
HO−N
O
O
O
Ethyl 3-(3-(benzyloxy)phenyl)-2-(hydroxyimino)propanoate (2aa)
160
150
140
130
120
110
100
90
80
70
60
50
40
30
20
10
ppm

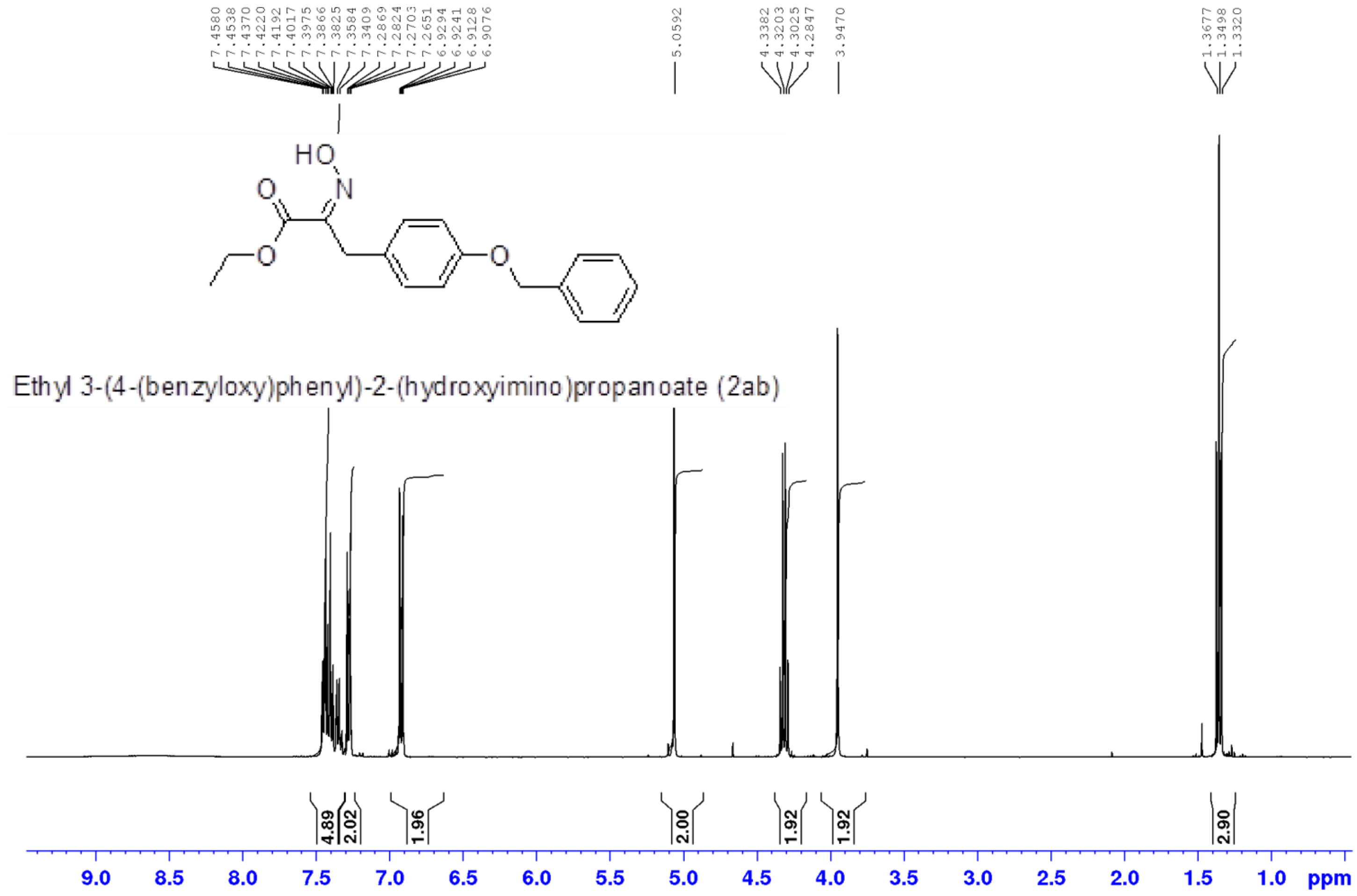
Ethyl 3-(4-(benzyloxy)phenyl)-2-(hydroxyimino)propanoate (2ab)

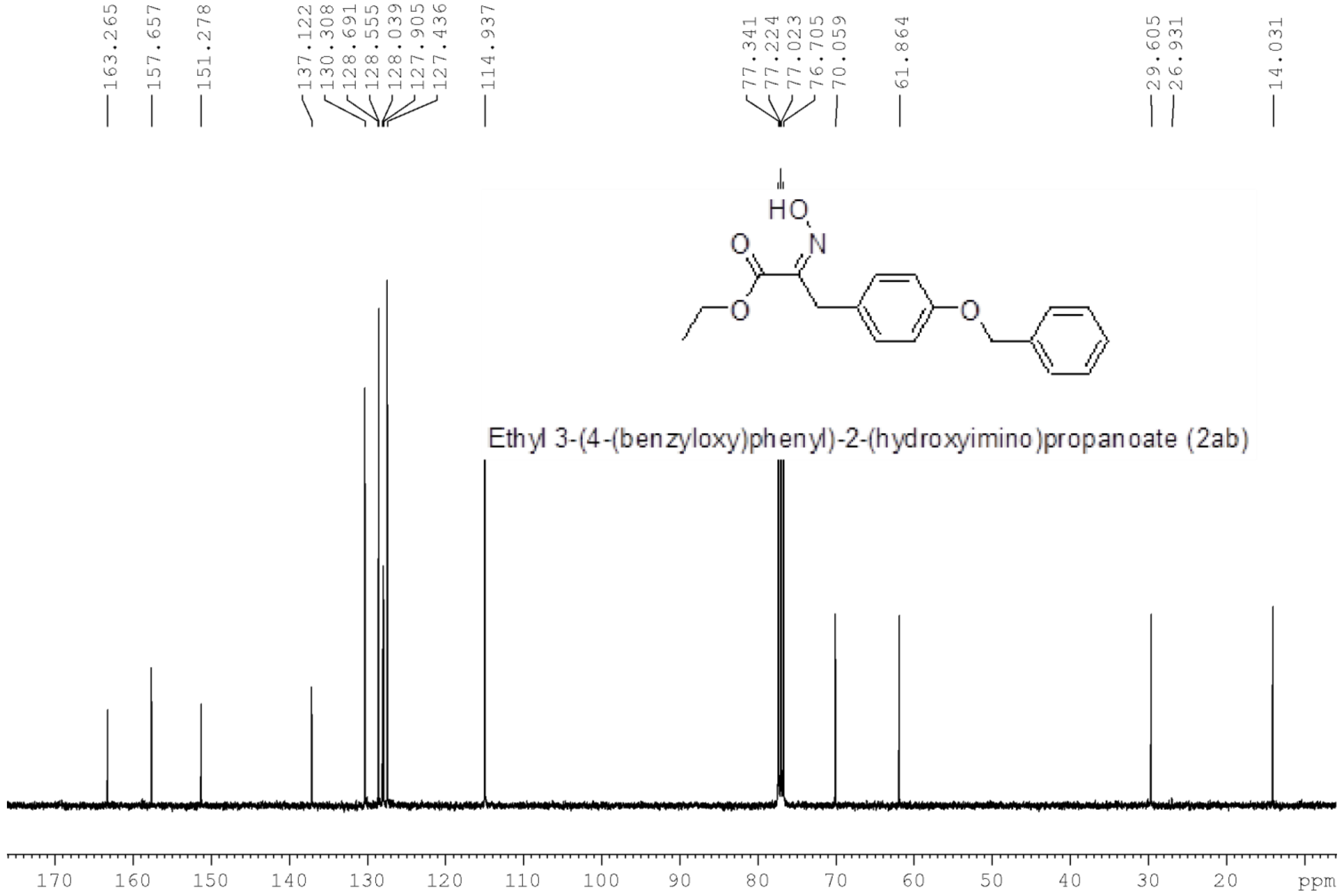
Ethyl 3-(4-(benzyloxy)phenyl)-2-(hydroxyimino)propanoate (2ab)

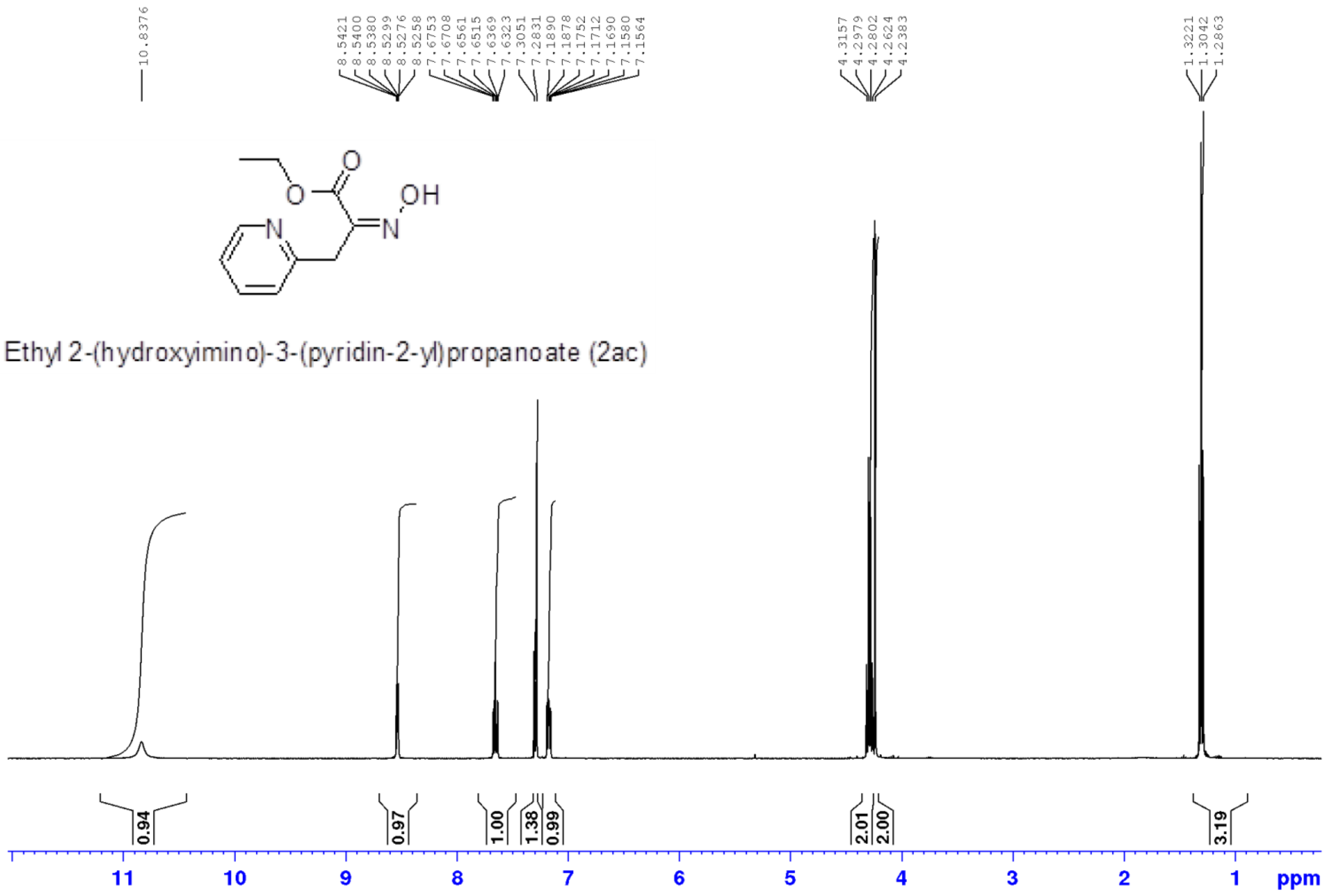
10.8376
8.5421 8.5400 8.5380 8.5299 8.5276 8.5258 7.6753 7.6708 7.6561 7.6515 7.6369 7.6323 7.3051 7.2831 7.1890 7.1878 7.1752 7.1712 7.1690 7.1580 7.1564
4.3157 4.2979 4.2802 4.2624 4.2383
1.3221 1.3042 1.2863
O
OH
N
N
Ethyl 2-(hydroxyimino)-3-(pyridin-2-yl)propanoate (2ac)
0.94
0.97
1.00
1.38
0.99
2.01
2.00
3.19
11 10 9 8 7 6 5 4 3 2 1 ppm

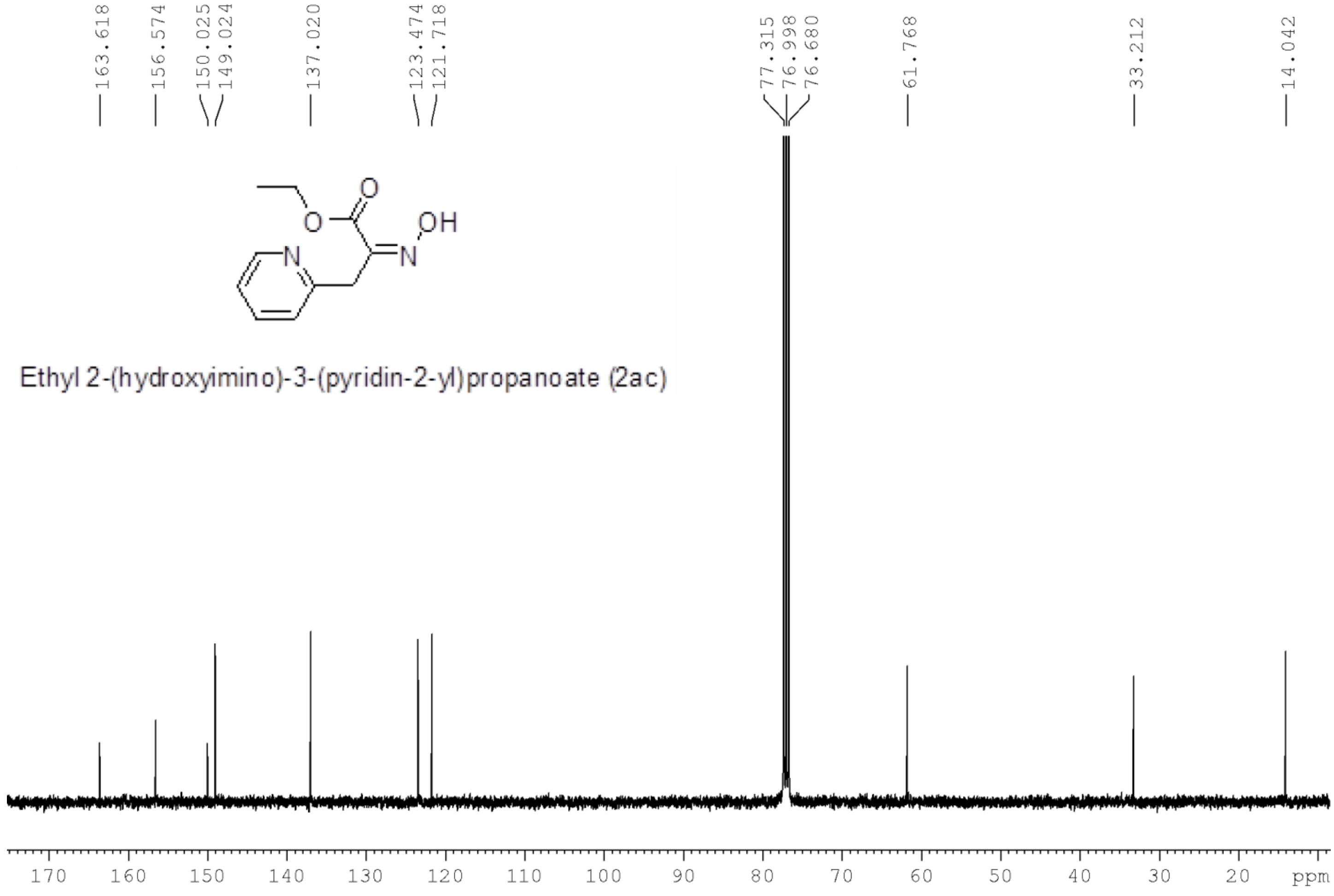
163.618
156.574
150.025
149.024
137.020
123.474
121.718
77.315 76.998 76.680
61.768
33.212
14.042
O
OH
N
N
Ethyl 2-(hydroxyimino)-3-(pyridin-2-yl)propanoate (2ac)
170 160 150 140 130 120 110 100 90 80 70 60 50 40 30 20 ppm

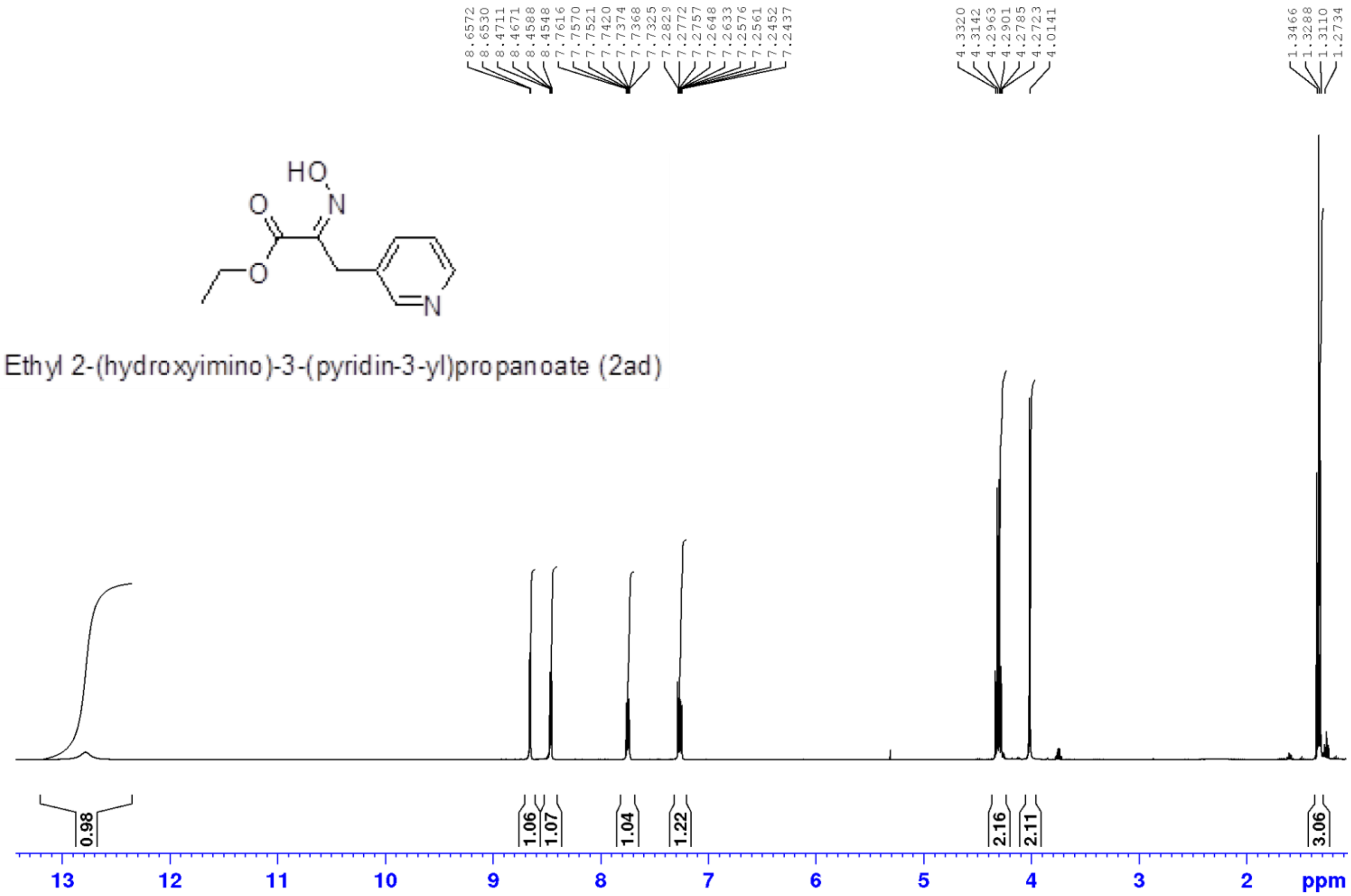
8.6572
8.6530
8.4711
8.4671
8.4588
8.4548
7.7616
7.7570
7.7521
7.7420
7.7374
7.7368
7.7325
7.2829
7.2772
7.2757
7.2648
7.2633
7.2576
7.2561
7.2452
7.2437
4.3320
4.3142
4.2963
4.2901
4.2785
4.2723
4.0141
1.3466
1.3288
1.3110
1.2734
HO
N
O
O
N
Ethyl 2-(hydroxyimino)-3-(pyridin-3-yl)propanoate (2ad)
0.98
1.06
1.07
1.04
1.22
2.16
2.11
3.06
13
12
11
10
9
8
7
6
5
4
3
2
ppm

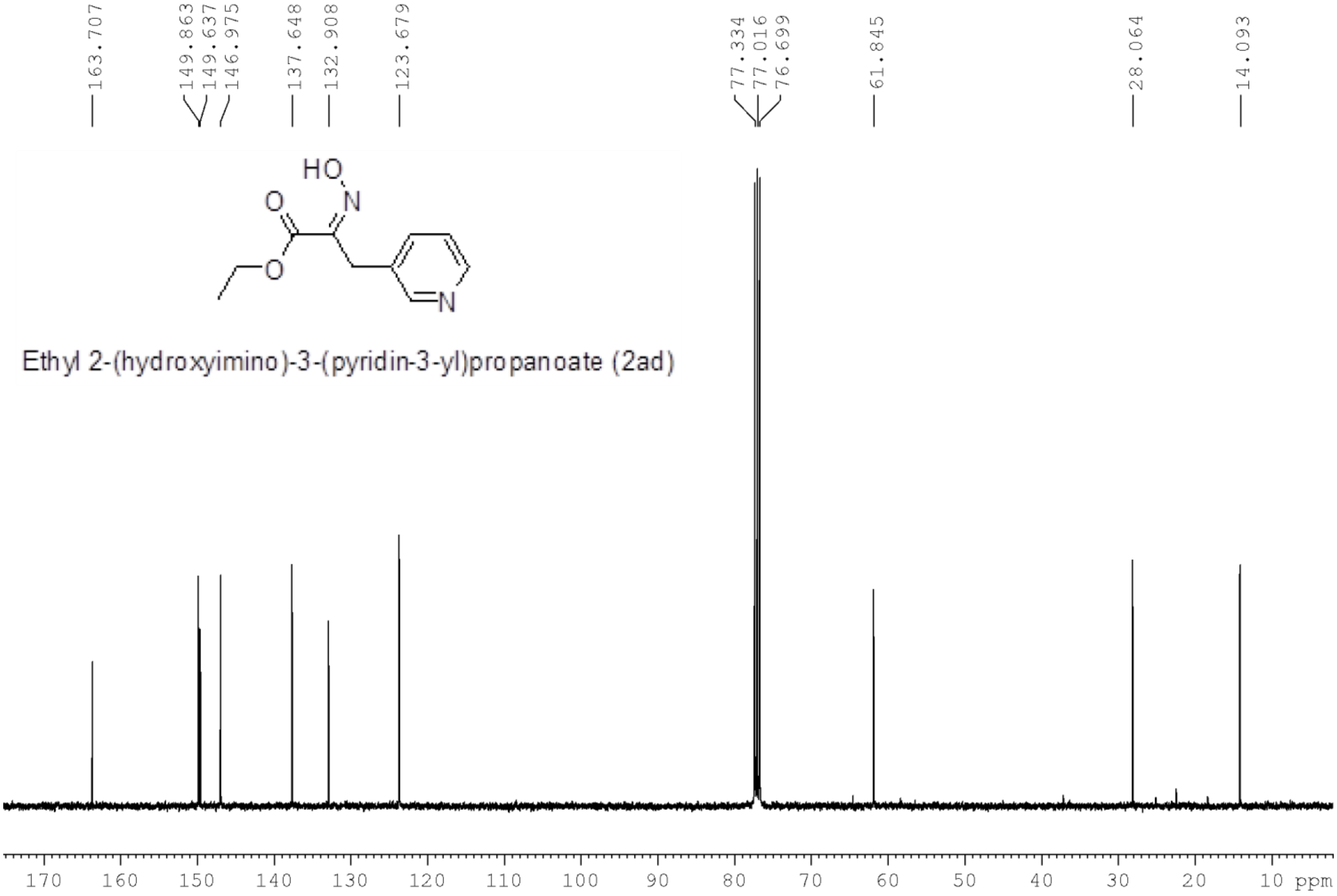
163.707
149.863
149.637
146.975
137.648
132.908
123.679
77.334
77.016
76.699
61.845
28.064
14.093
HO
N
O
O
N
Ethyl 2-(hydroxyimino)-3-(pyridin-3-yl)propanoate (2ad)
170
160
150
140
130
120
110
100
90
80
70
60
50
40
30
20
10 ppm

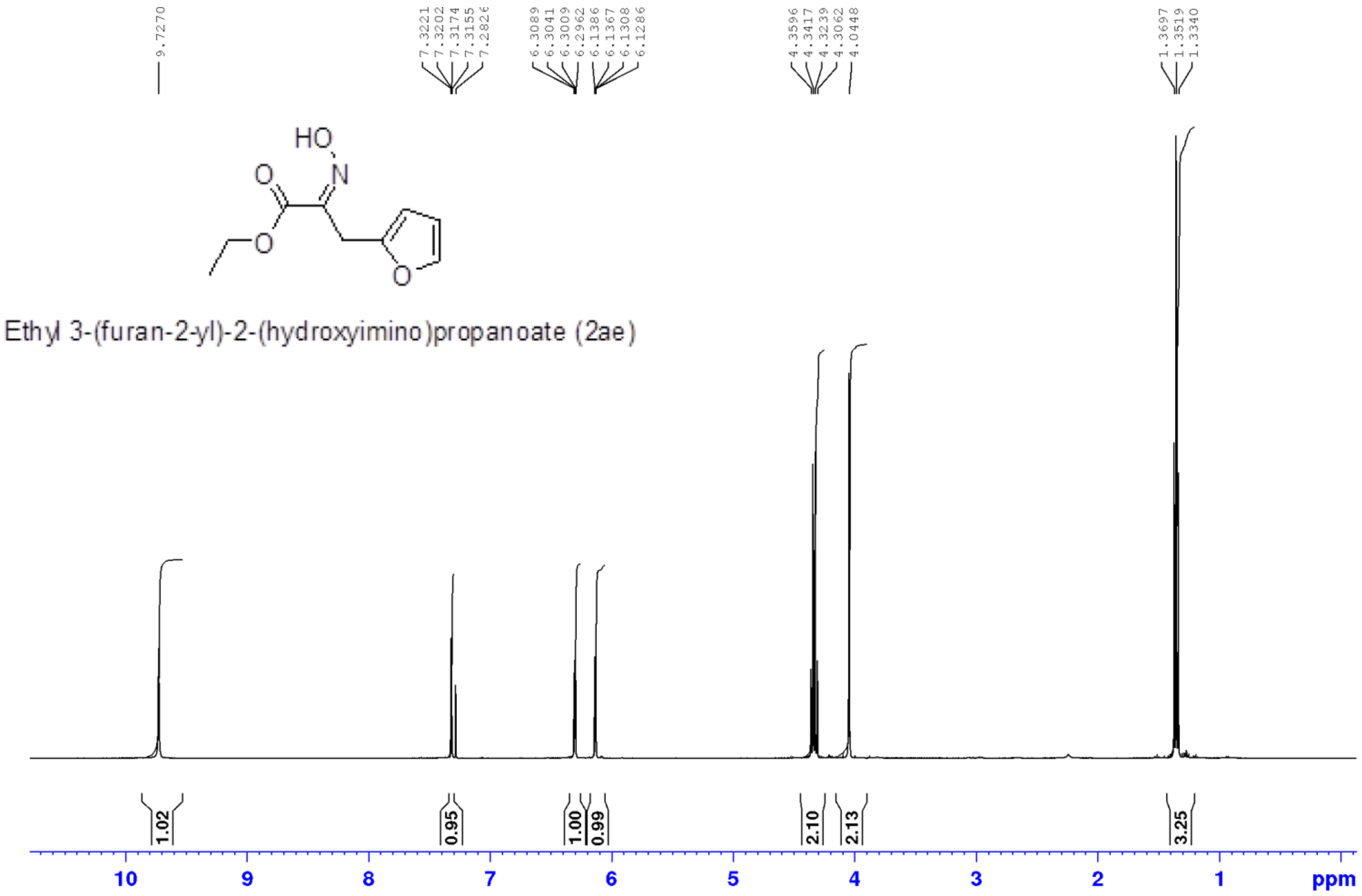
9.7270
7.3221
7.3202
7.3174
7.3155
7.2826
6.3089
6.3041
6.3009
6.2962
6.1386
6.1367
6.1308
6.1286
4.3596
4.3417
4.3239
4.3062
4.0448
1.3697
1.3519
1.3340
HO
N
O
O
O
Ethyl 3-(furan-2-yl)-2-(hydroxyimino)propanoate (2ae)
1.02
0.95
1.00
0.99
2.10
2.13
3.25
10
9
8
7
6
5
4
3
2
1
ppm

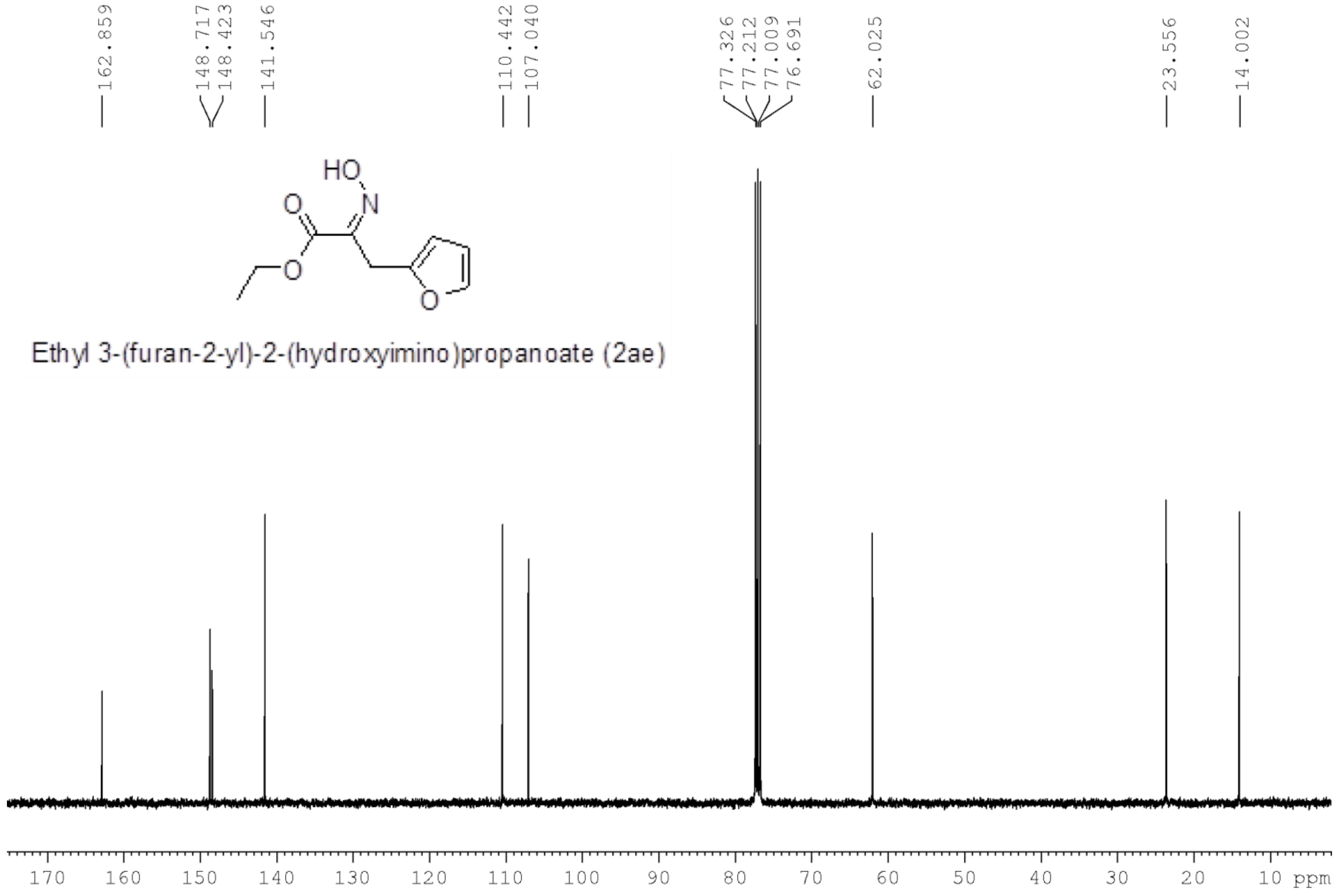
162.859
148.717
148.423
141.546
110.442
107.040
77.326
77.212
77.009
76.691
62.025
23.556
14.002
HO
N
O
O
O
Ethyl 3-(furan-2-yl)-2-(hydroxyimino)propanoate (2ae)
170
160
150
140
130
120
110
100
90
80
70
60
50
40
30
20
10 ppm

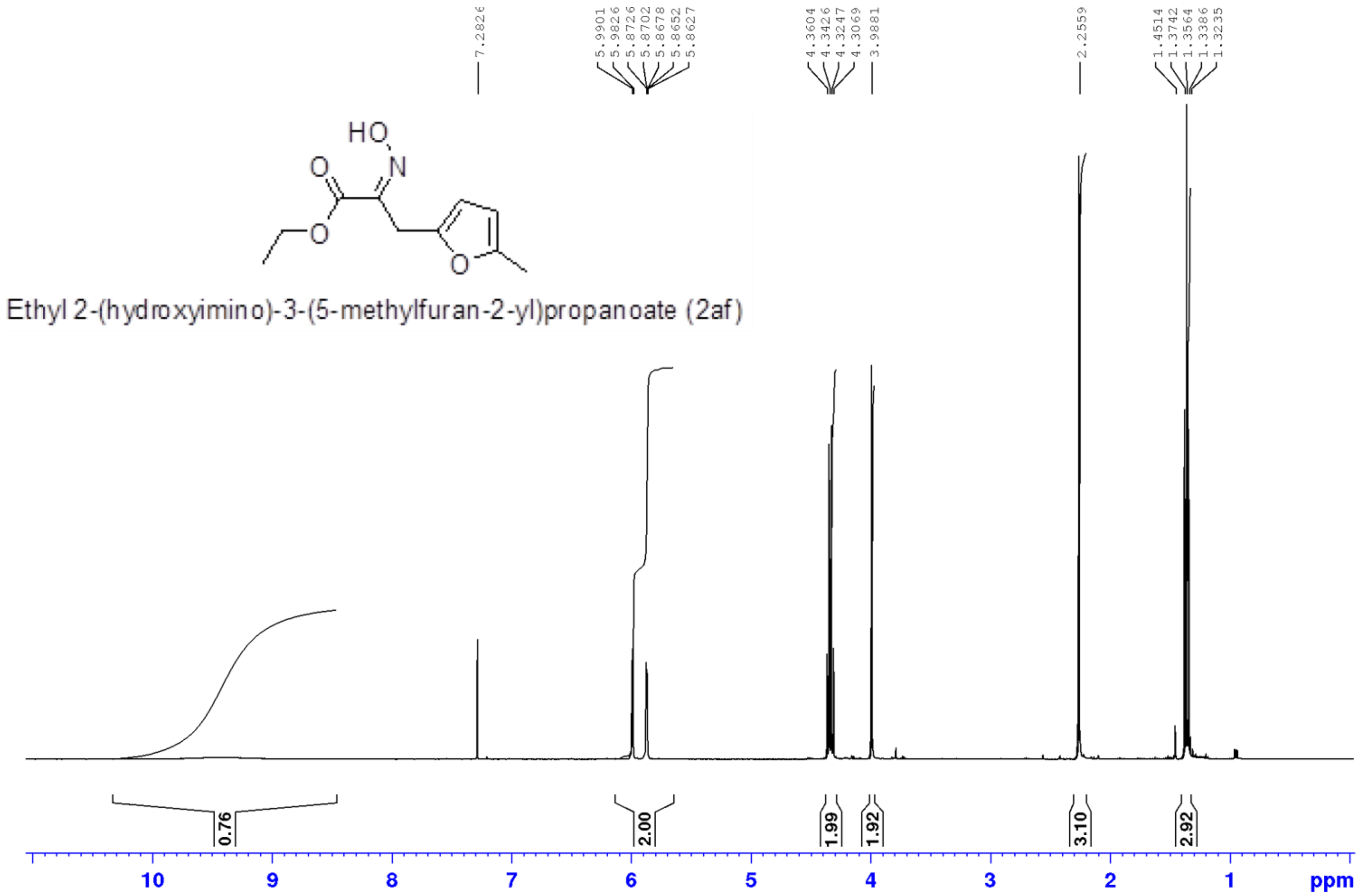
Ethyl 2-(hydroxyimino)-3-(5-methylfuran-2-yl)propanoate (2af)
7.2826
5.9901
5.9826
5.8726
5.8702
5.8678
5.8652
5.8627
4.3604
4.3426
4.3247
4.3069
3.9881
2.2559
1.4514
1.3742
1.3564
1.3386
1.3235
0.76
2.00
1.99
1.92
3.10
2.92
ppm

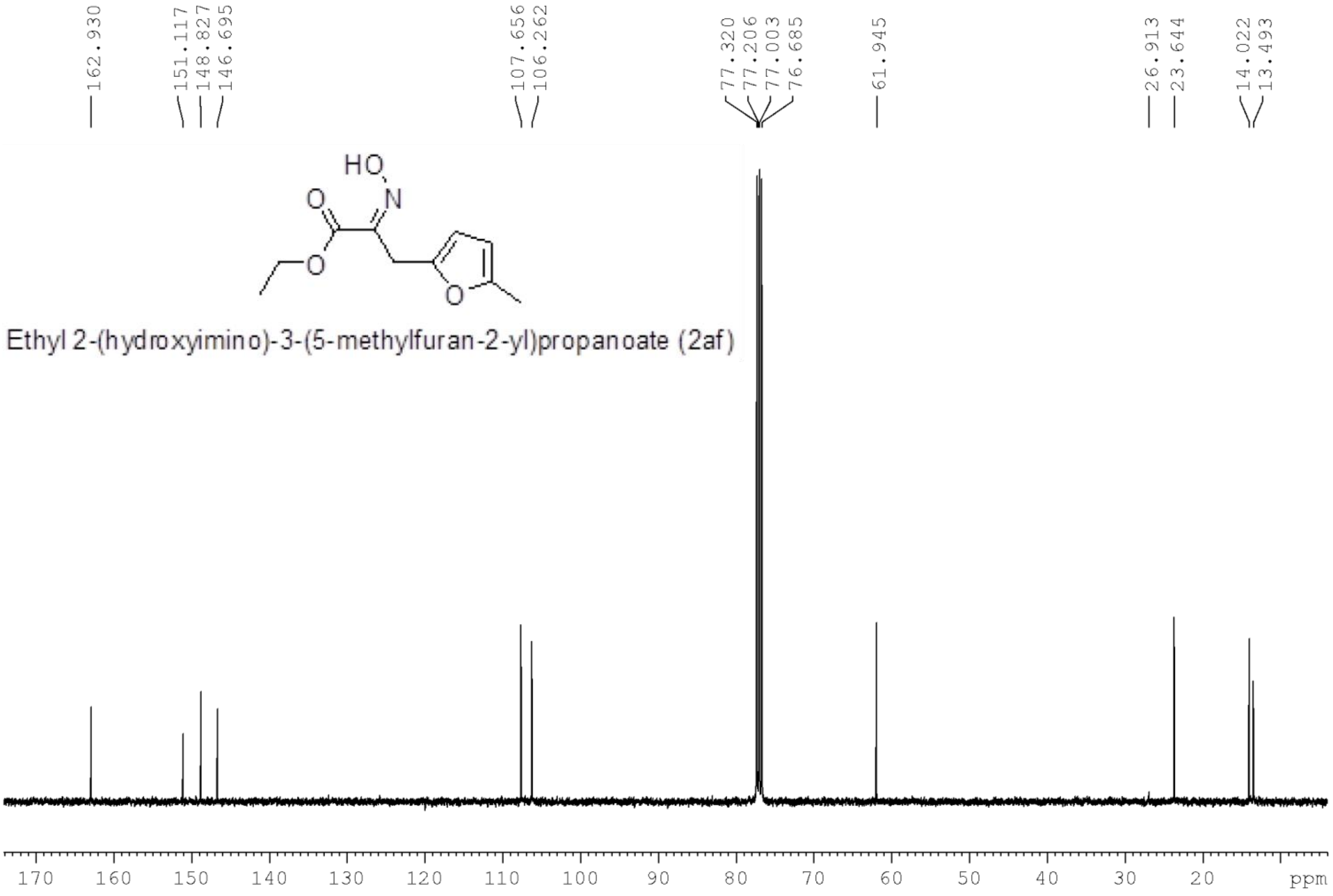
Ethyl 2-(hydroxyimino)-3-(5-methylfuran-2-yl)propanoate (2af)
162.930
151.117
148.827
146.695
107.656
106.262
77.320
77.206
77.003
76.685
61.945
26.913
23.644
14.022
13.493
ppm

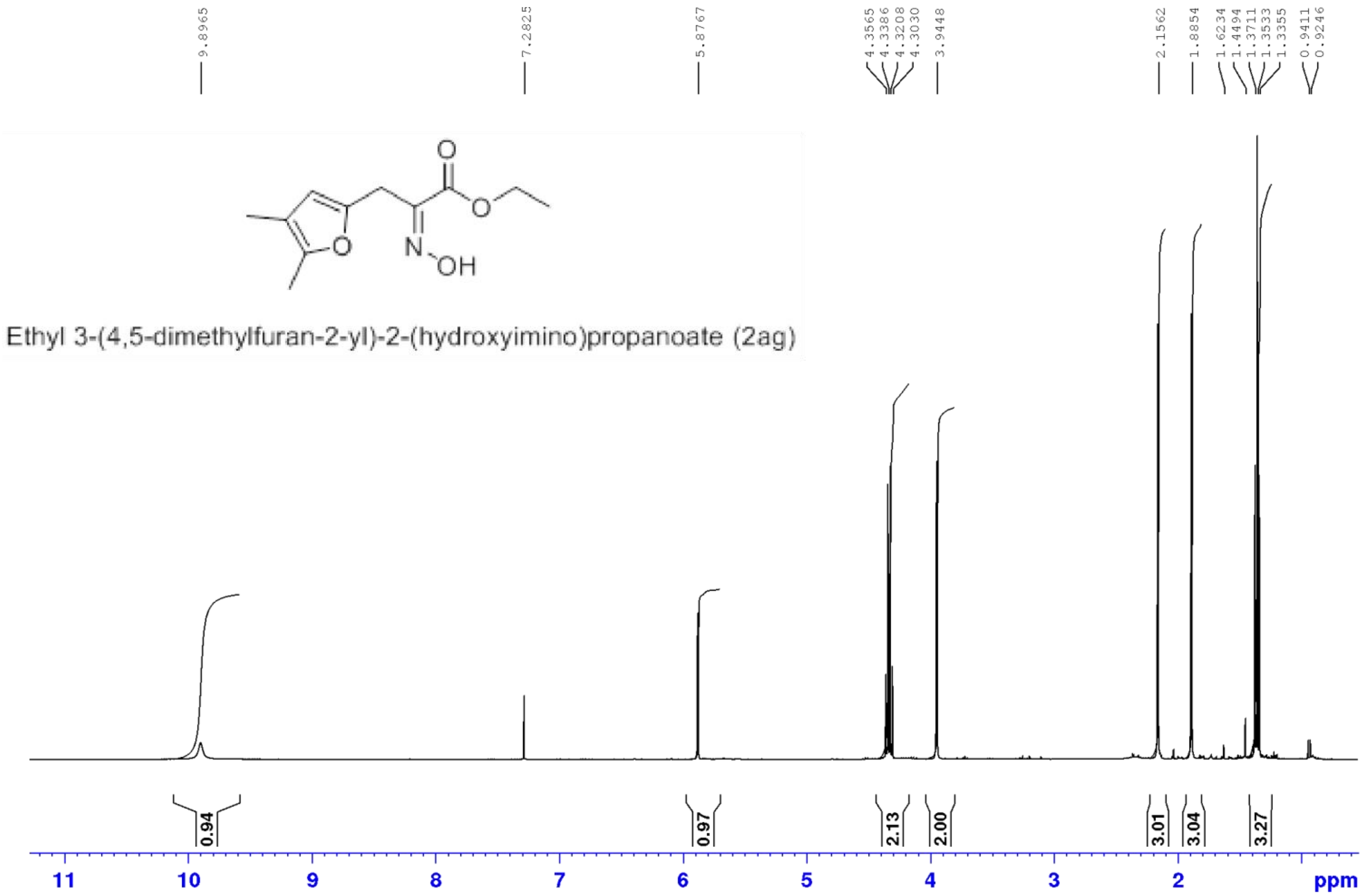
9.8965
7.2825
5.8767
4.3565
4.3386
4.3208
4.3030
3.9448
2.1562
1.8854
1.6234
1.4494
1.3711
1.3533
1.3355
0.9411
0.9246
O
N
OH
Ethyl 3-(4,5-dimethylfuran-2-yl)-2-(hydroxyimino)propanoate (2ag)
0.94
0.97
2.13
2.00
3.01
3.04
3.27
11
10
9
8
7
6
5
4
3
2
ppm

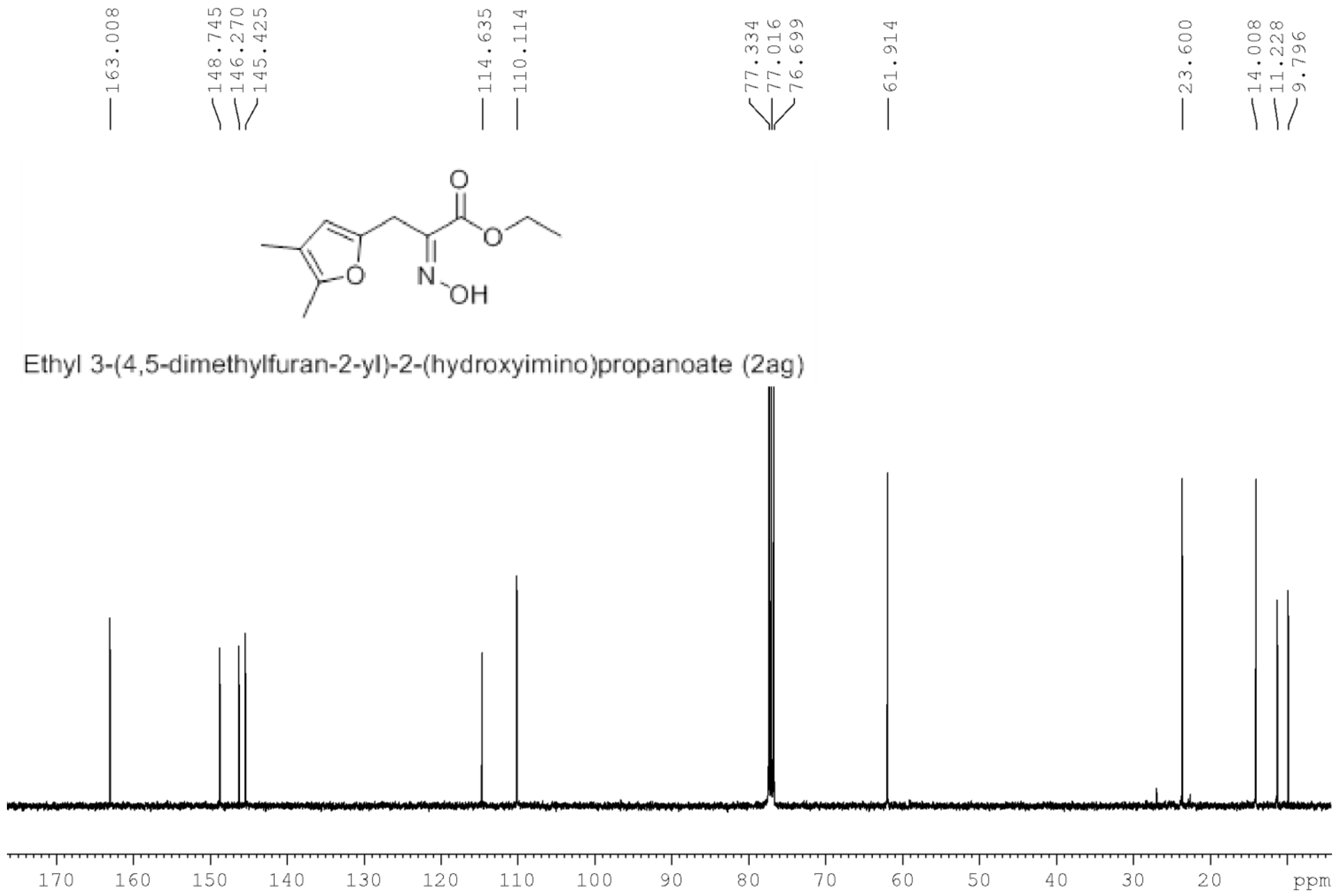
163.008
148.745
146.270
145.425
114.635
110.114
77.334
77.016
76.699
61.914
23.600
14.008
11.228
9.796
O
N
OH
Ethyl 3-(4,5-dimethylfuran-2-yl)-2-(hydroxyimino)propanoate (2ag)
170
160
150
140
130
120
110
100
90
80
70
60
50
40
30
20
ppm

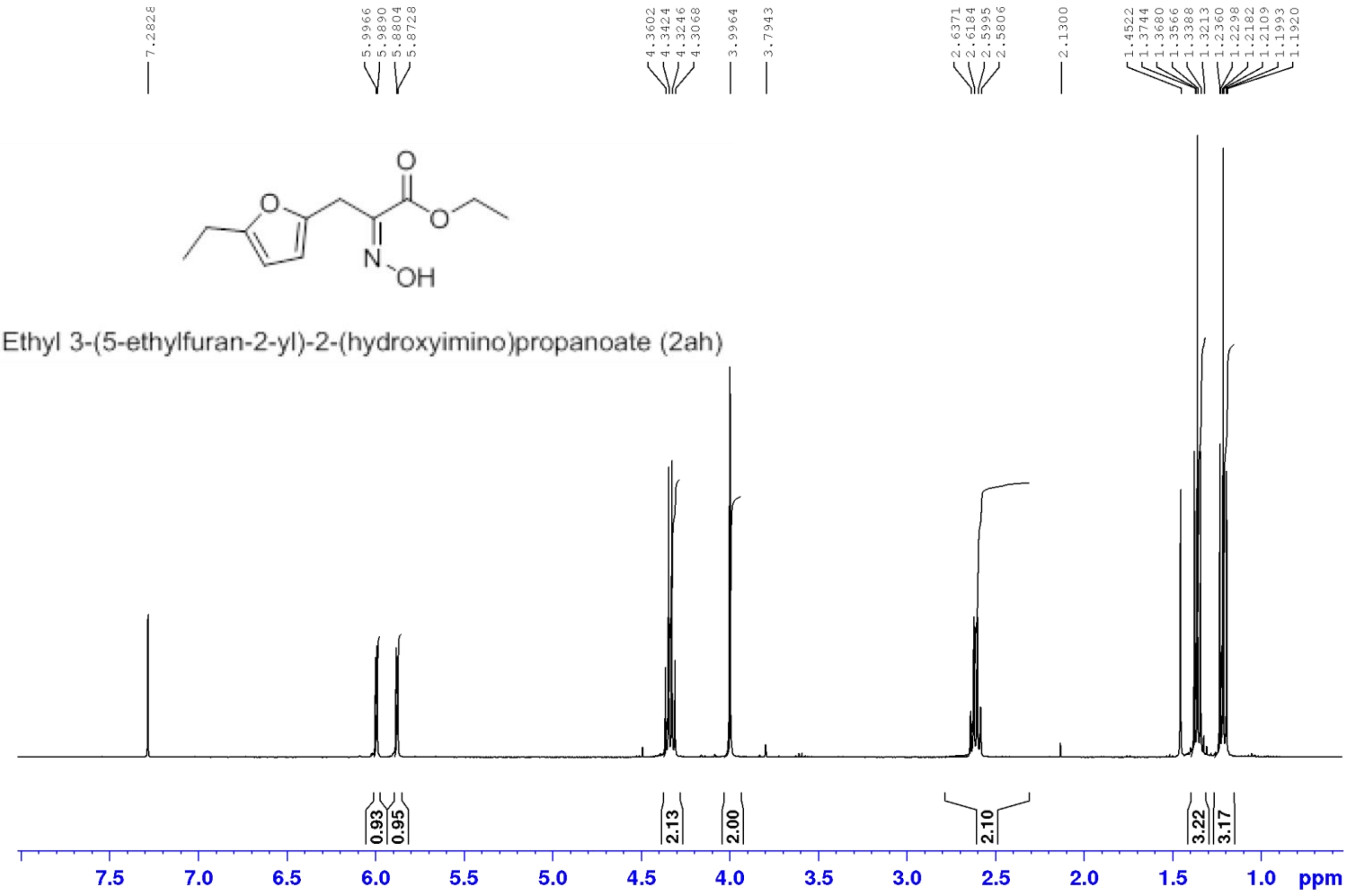
7.2828
5.9966
5.9890
5.8804
5.8728
4.3602
4.3424
4.3246
4.3068
3.9964
3.7943
2.6371
2.6184
2.5995
2.5806
2.1300
1.4522
1.3744
1.3680
1.3566
1.3388
1.3213
1.2360
1.2298
1.2182
1.2109
1.1993
1.1920
O
N
OH
Ethyl 3-(5-ethylfuran-2-yl)-2-(hydroxyimino)propanoate (2ah)
0.93
0.95
2.13
2.00
2.10
3.22
3.17
7.5
7.0
6.5
6.0
5.5
5.0
4.5
4.0
3.5
3.0
2.5
2.0
1.5
1.0
ppm

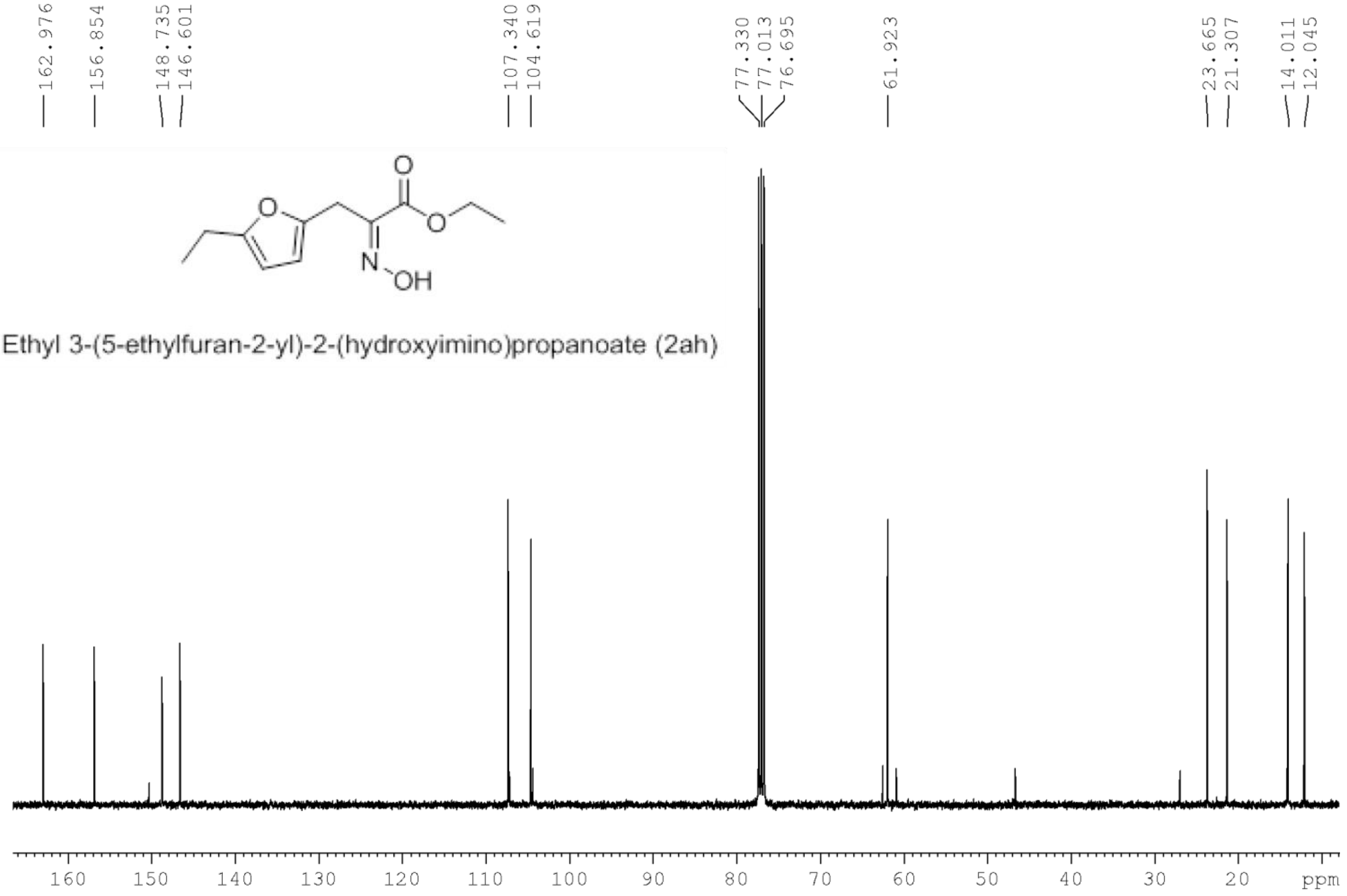
162.976
156.854
148.735
146.601
107.340
104.619
77.330
77.013
76.695
61.923
23.665
21.307
14.011
12.045
O
N
OH
Ethyl 3-(5-ethylfuran-2-yl)-2-(hydroxyimino)propanoate (2ah)
160
150
140
130
120
110
100
90
80
70
60
50
40
30
20
ppm

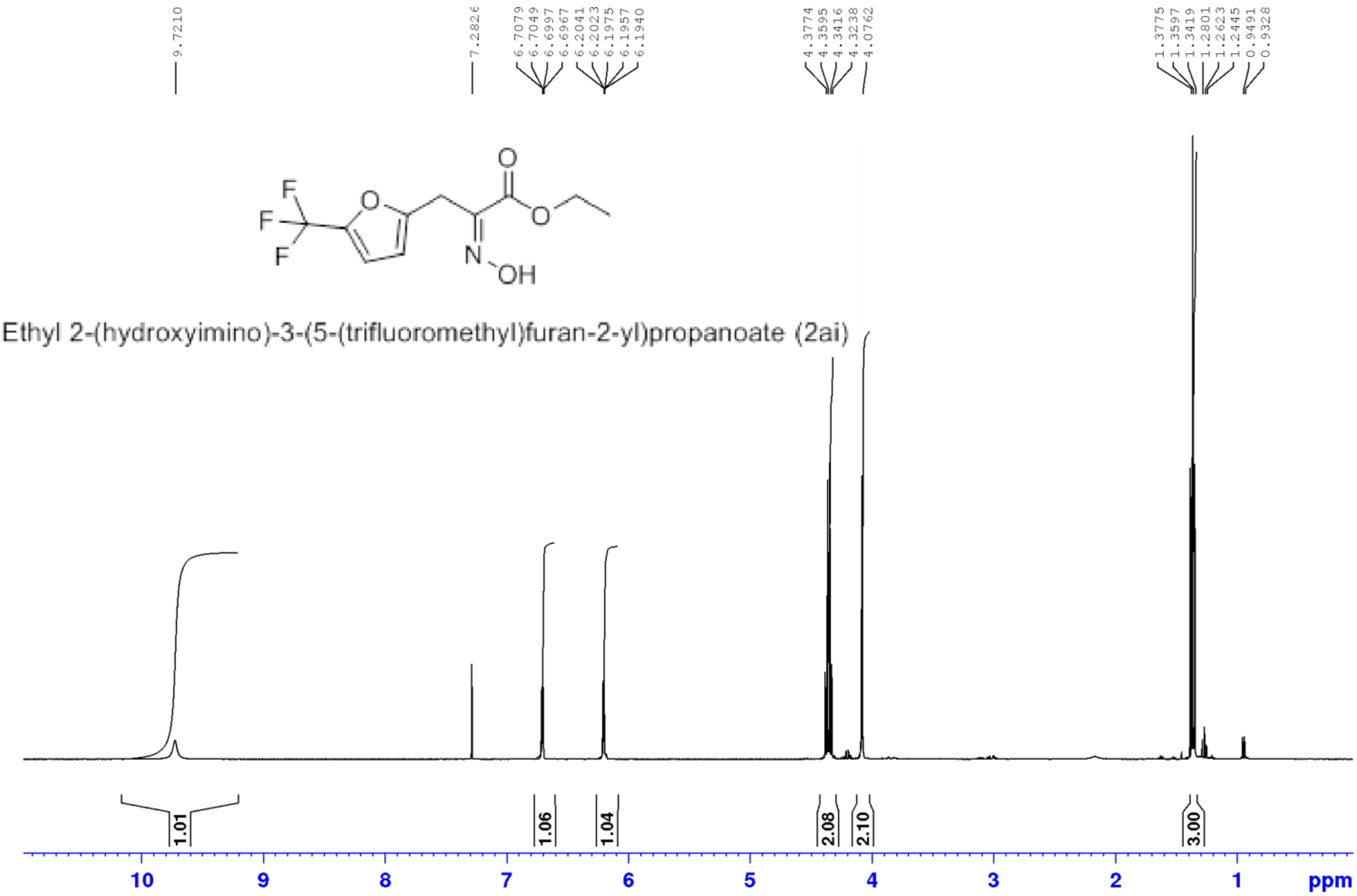
9.7210
7.2826
6.7079
6.7049
6.6997
6.6967
6.2041
6.2023
6.1975
6.1957
6.1940
4.3774
4.3595
4.3416
4.3238
4.0762
1.3775
1.3597
1.3419
1.2801
1.2623
1.2445
0.9491
0.9328
Ethyl 2-(hydroxyimino)-3-(5-(trifluoromethyl)furan-2-yl)propanoate (2ai)
1.01
1.06
1.04
2.08
2.10
3.00
ppm

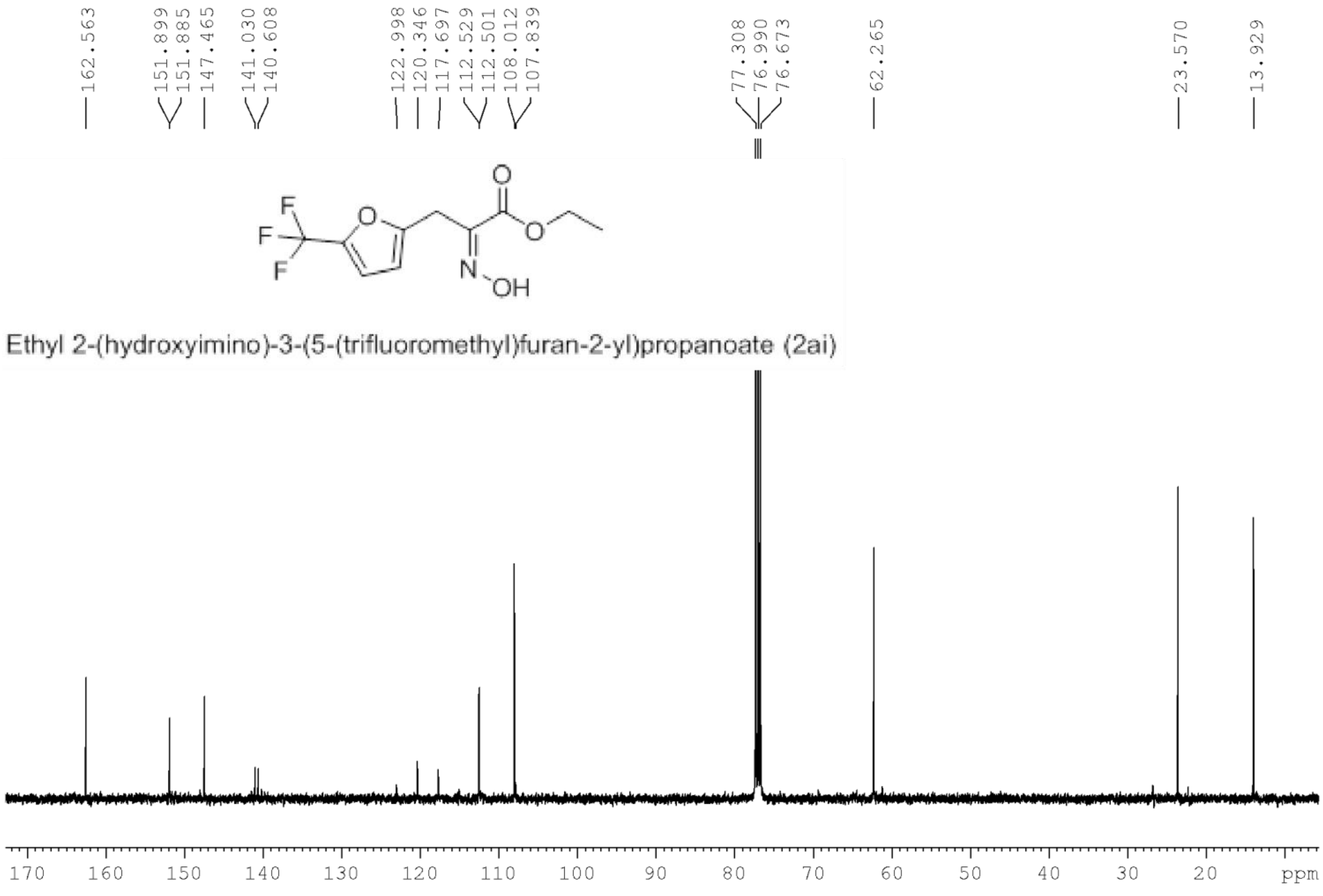
162.563
151.899
151.885
147.465
141.030
140.608
122.998
120.346
117.697
112.529
112.501
108.012
107.839
77.308
76.990
76.673
62.265
23.570
13.929
Ethyl 2-(hydroxyimino)-3-(5-(trifluoromethyl)furan-2-yl)propanoate (2ai)
ppm

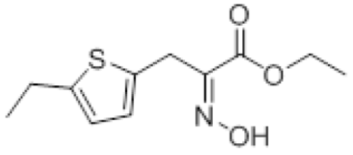

Ethyl 3-(5-ethylthiophen-2-yl)-2-(hydroxyimino)propanoate (2aj)

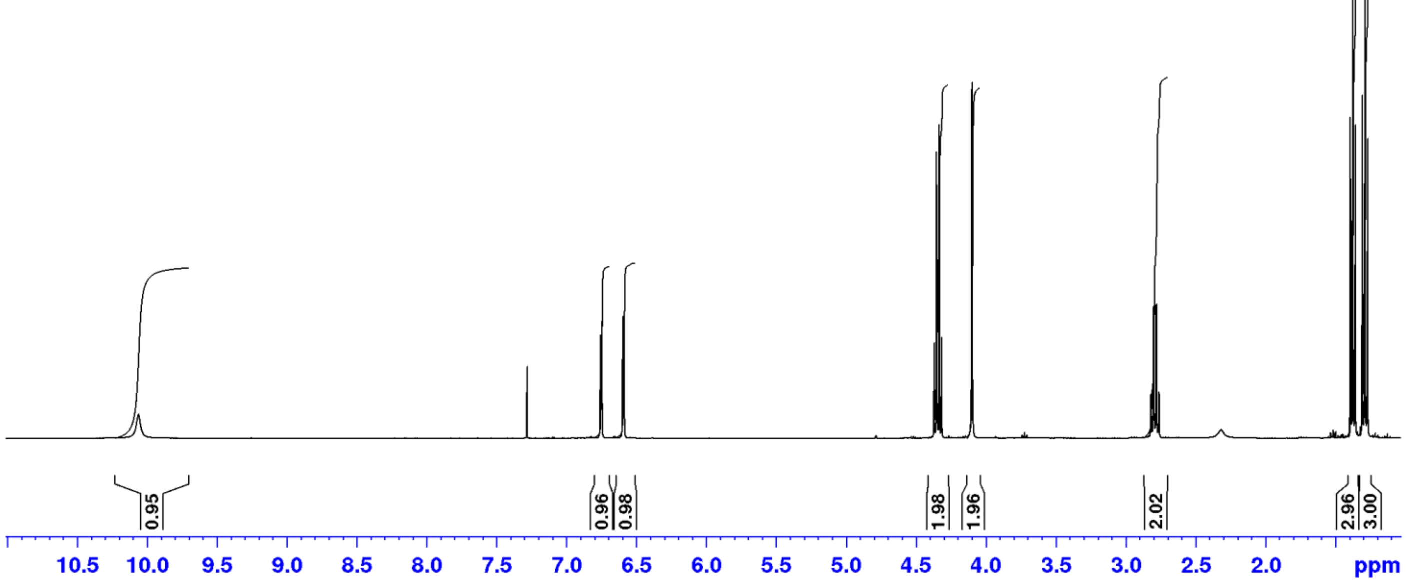

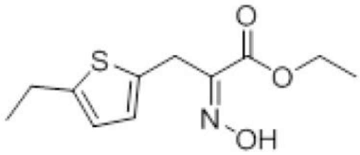

Ethyl 3-(5-ethylthiophen-2-yl)-2-(hydroxyimino)propanoate (2aj)

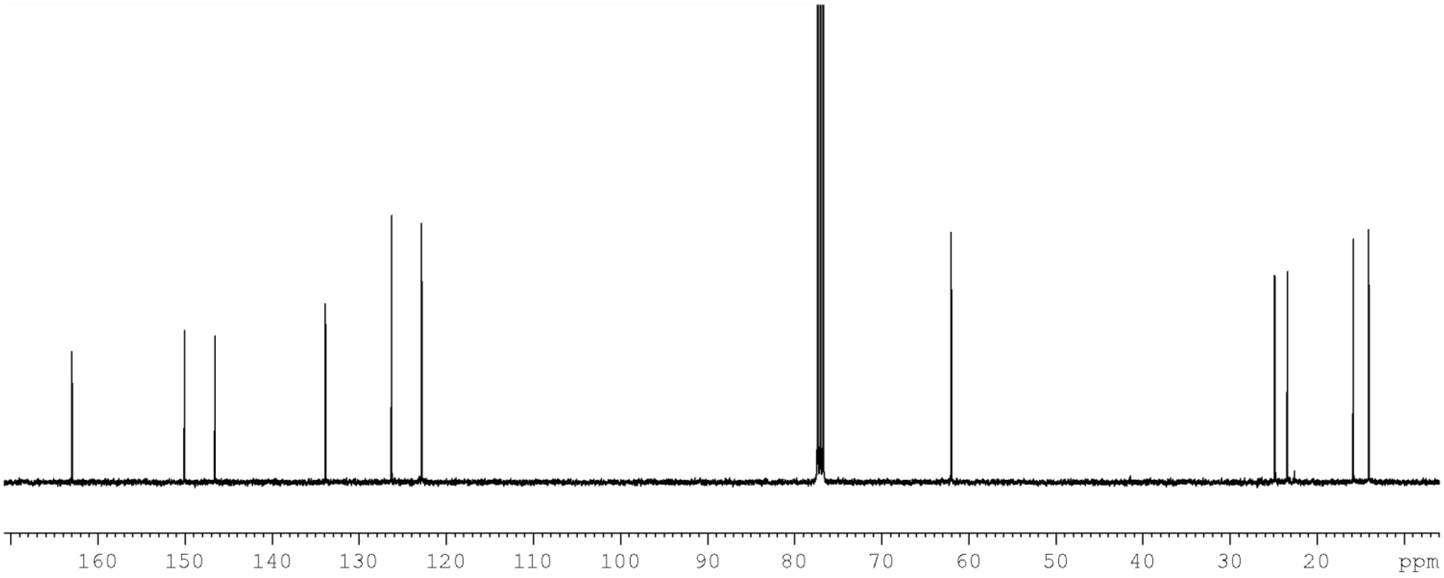

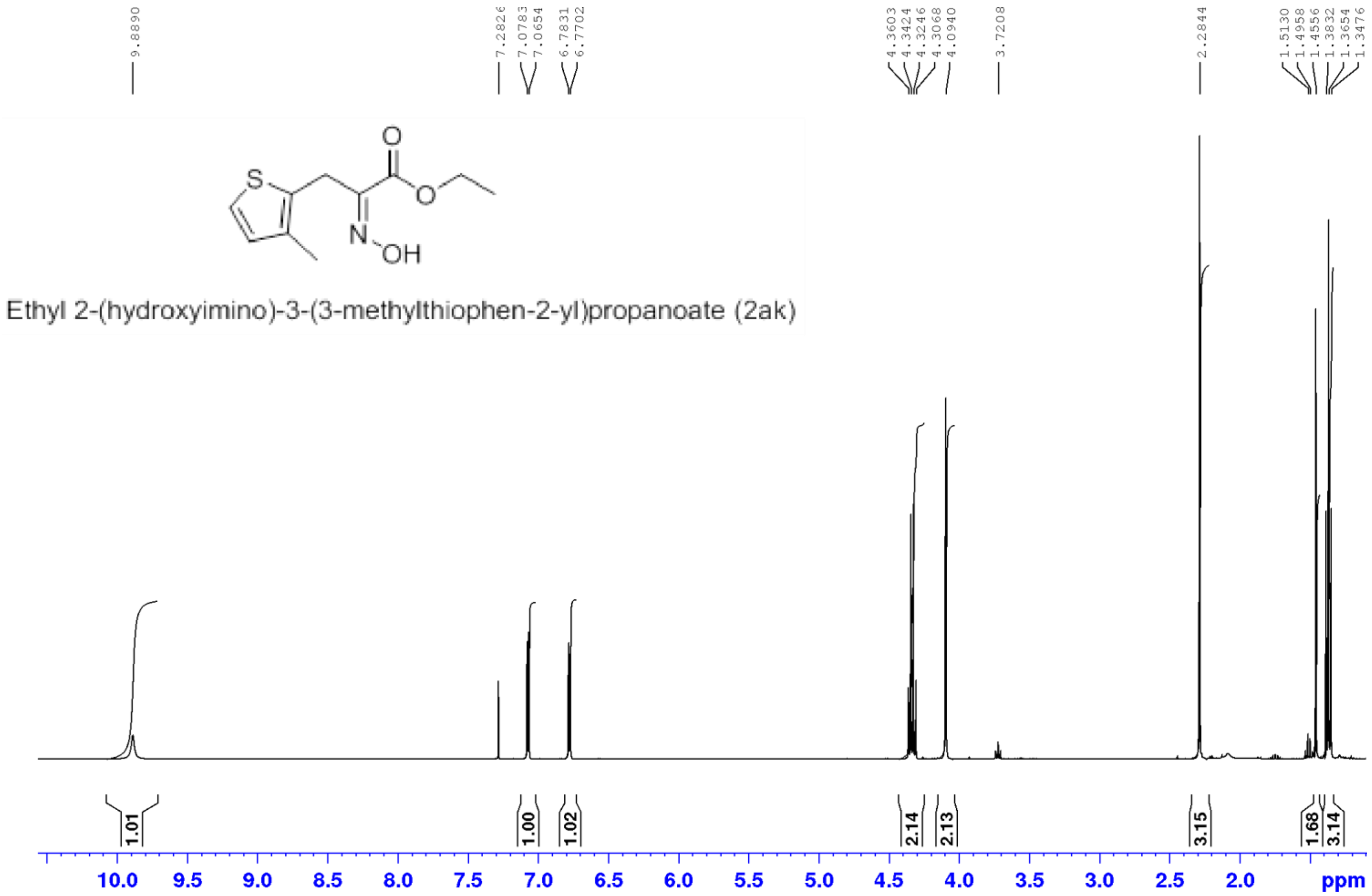
9.8890
7.2826
7.0783
7.0654
6.7831
6.7702
4.3603
4.3424
4.3246
4.3068
4.0940
3.7208
2.2844
1.5130
1.4958
1.4556
1.3832
1.3654
1.3476
Ethyl 2-(hydroxyimino)-3-(3-methylthiophen-2-yl)propanoate (2ak)
1.01
1.00
1.02
2.14
2.13
3.15
1.68
3.14
10.0
9.5
9.0
8.5
8.0
7.5
7.0
6.5
6.0
5.5
5.0
4.5
4.0
3.5
3.0
2.5
2.0
ppm

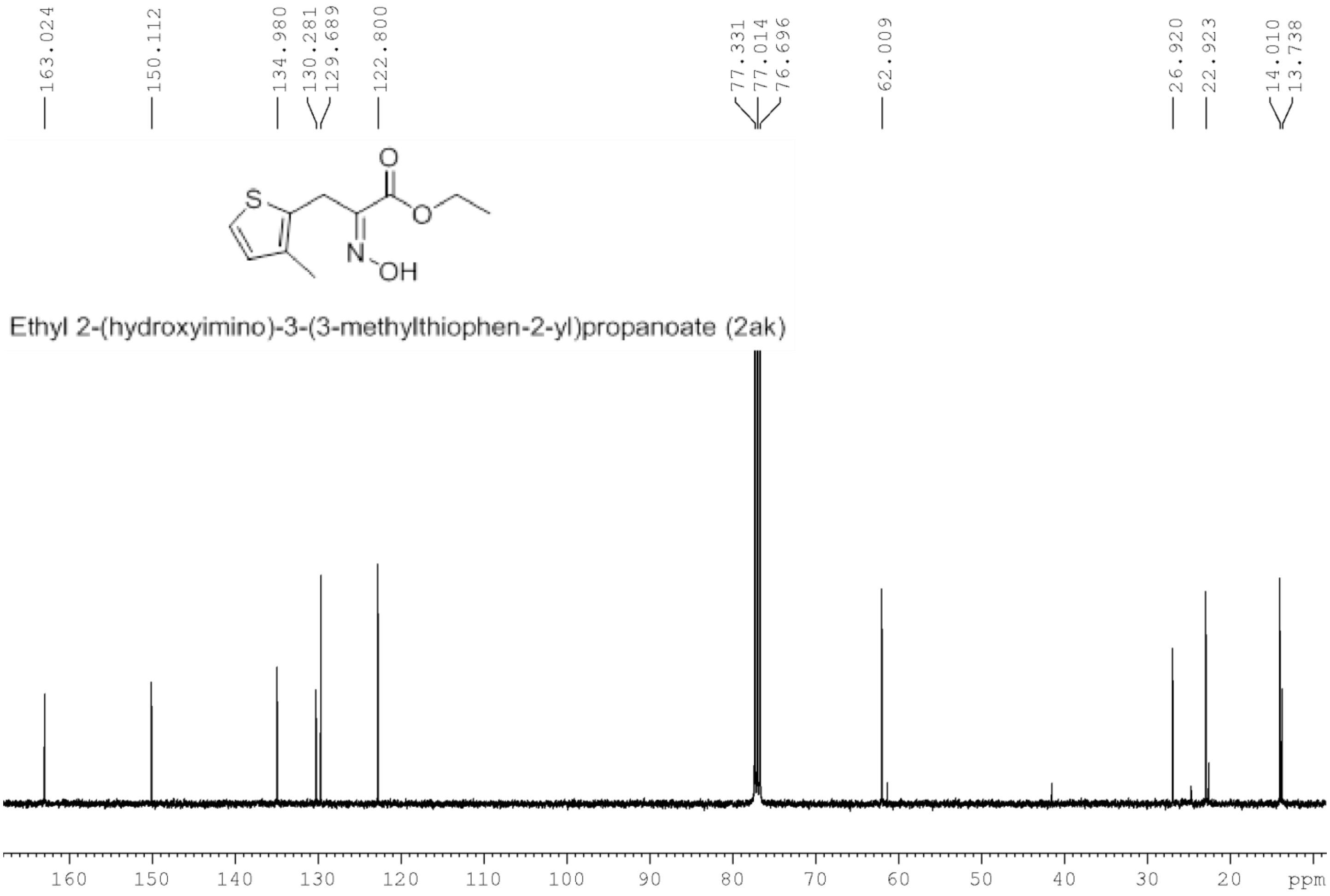
163.024
150.112
134.980
130.281
129.689
122.800
77.331
77.014
76.696
62.009
26.920
22.923
14.010
13.738
Ethyl 2-(hydroxyimino)-3-(3-methylthiophen-2-yl)propanoate (2ak)
160
150
140
130
120
110
100
90
80
70
60
50
40
30
20
ppm

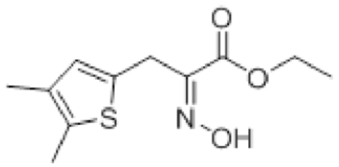

Ethyl 3-(4,5-dimethylthiophen-2-yl)-2-(hydroxyimino)propanoate (2al)

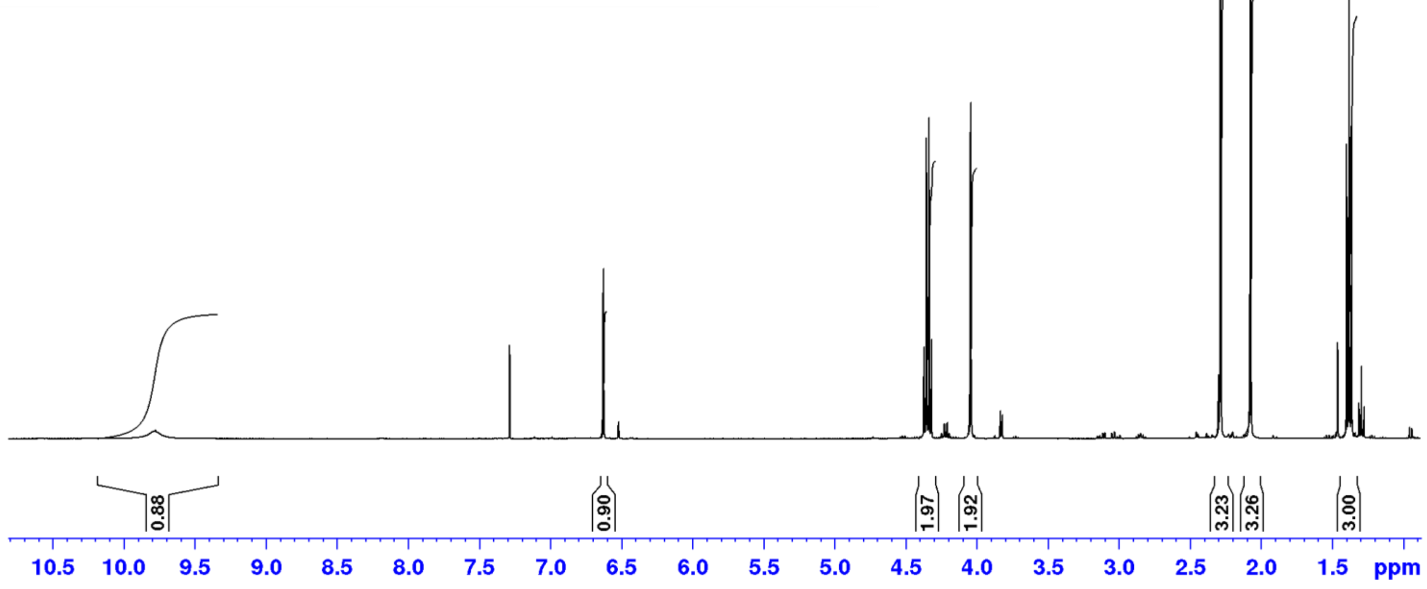

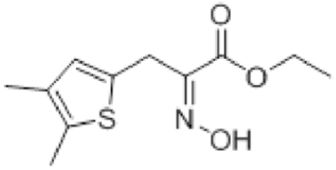

Ethyl 3-(4,5-dimethylthiophen-2-yl)-2-(hydroxyimino)propanoate (2al)

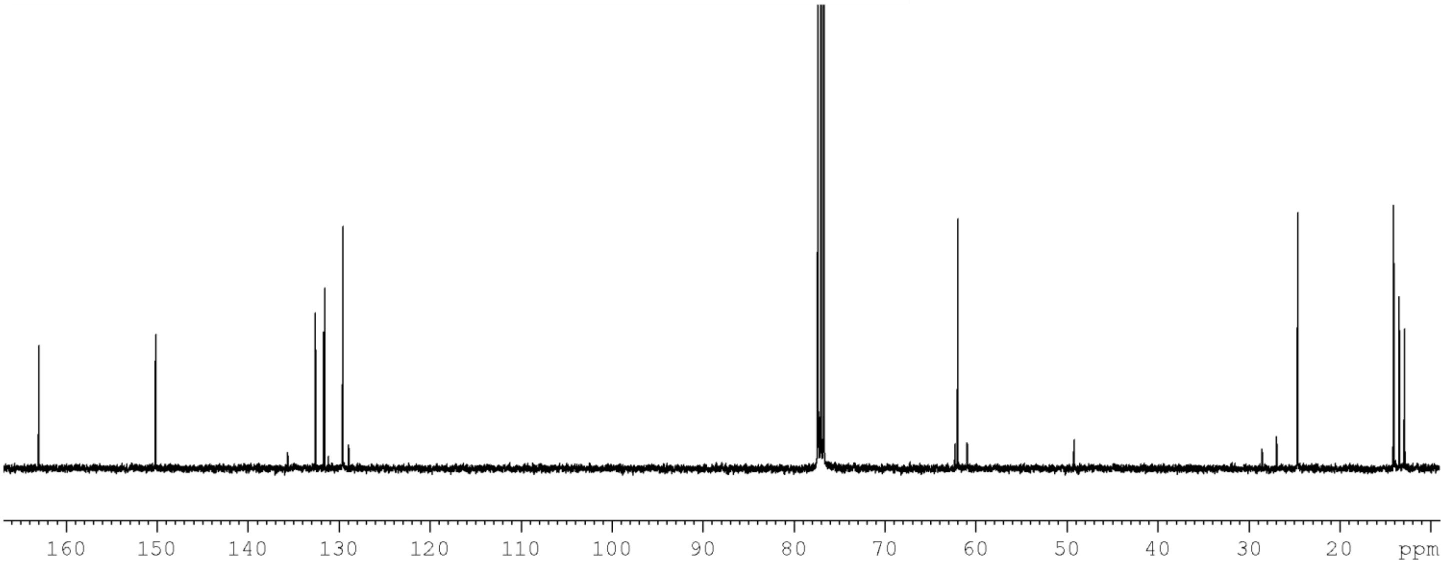

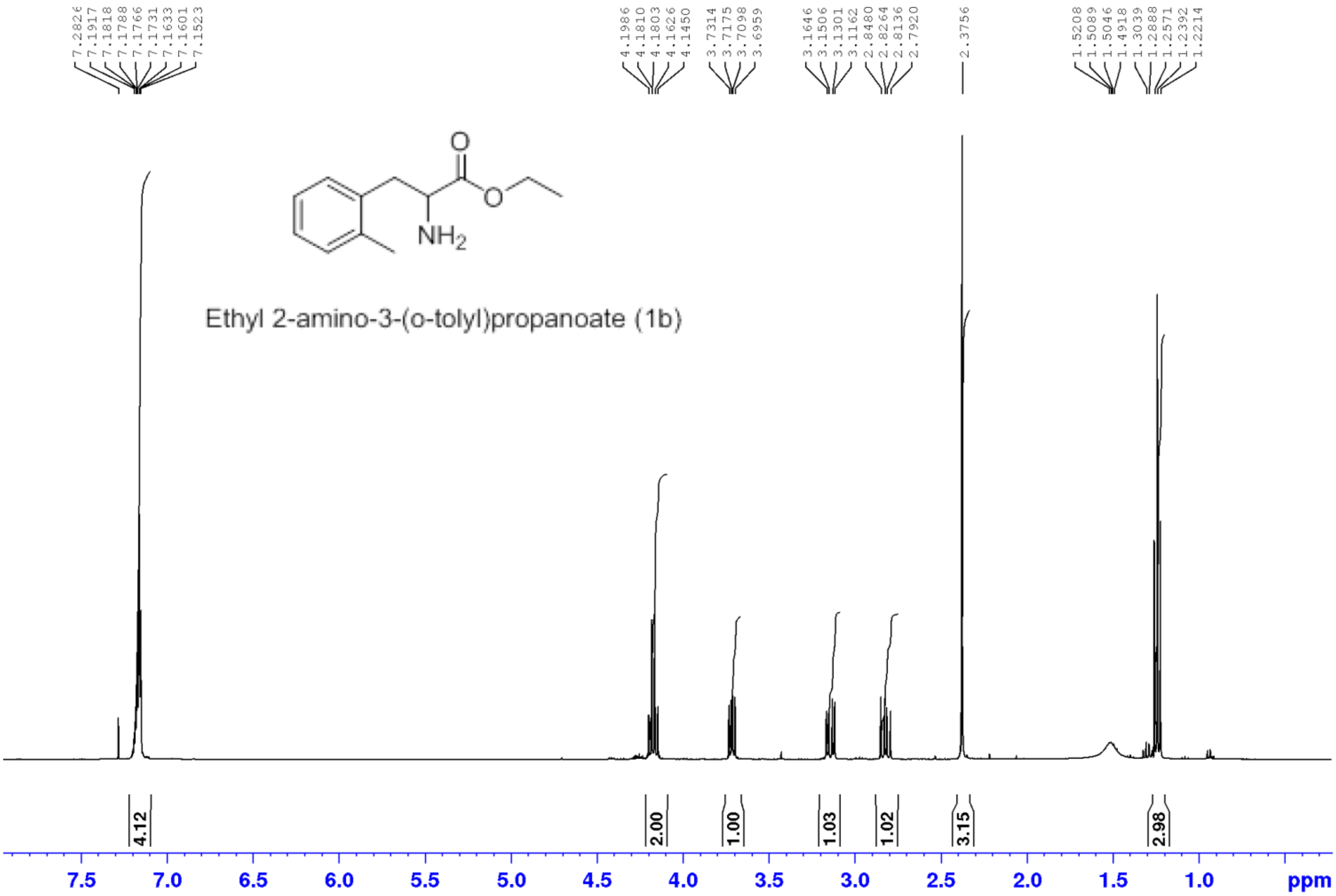
7.2826
7.1917
7.1818
7.1788
7.1766
7.1731
7.1633
7.1601
7.1523
4.1986
4.1810
4.1803
4.1626
4.1450
3.7314
3.7175
3.7098
3.6959
3.1646
3.1506
3.1301
3.1162
2.8480
2.8264
2.8136
2.7920
2.3756
1.5208
1.5089
1.5046
1.4918
1.3039
1.2888
1.2571
1.2392
1.2214
O
O
NH2
Ethyl 2-amino-3-(o-tolyl)propanoate (1b)
4.12
2.00
1.00
1.03
1.02
3.15
2.98
7.5
7.0
6.5
6.0
5.5
5.0
4.5
4.0
3.5
3.0
2.5
2.0
1.5
1.0
ppm

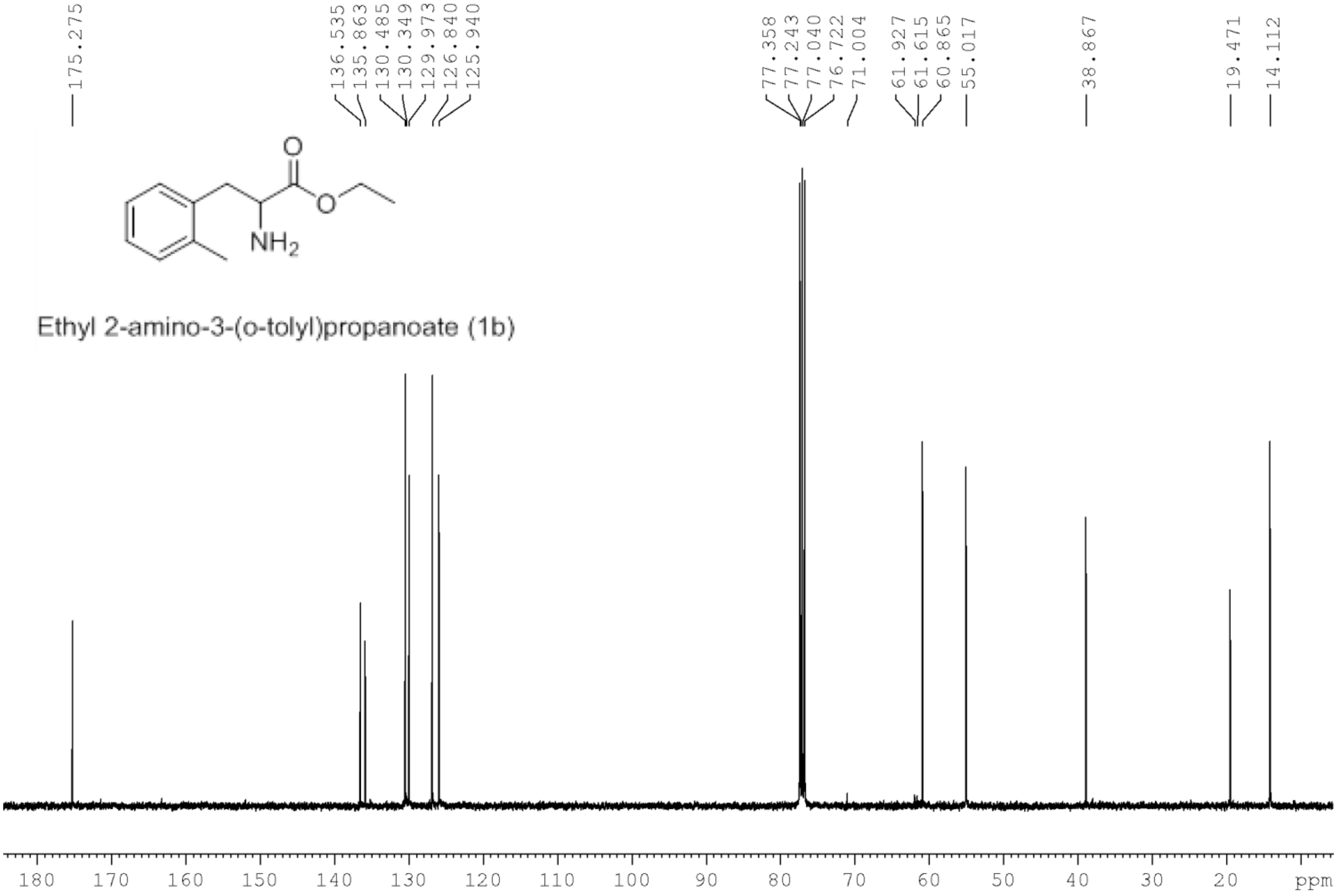
175.275
136.535
135.863
130.485
130.349
129.973
126.840
125.940
77.358
77.243
77.040
76.722
71.004
61.927
61.615
60.865
55.017
38.867
19.471
14.112
O
O
NH2
Ethyl 2-amino-3-(o-tolyl)propanoate (1b)
180
170
160
150
140
130
120
110
100
90
80
70
60
50
40
30
20
ppm

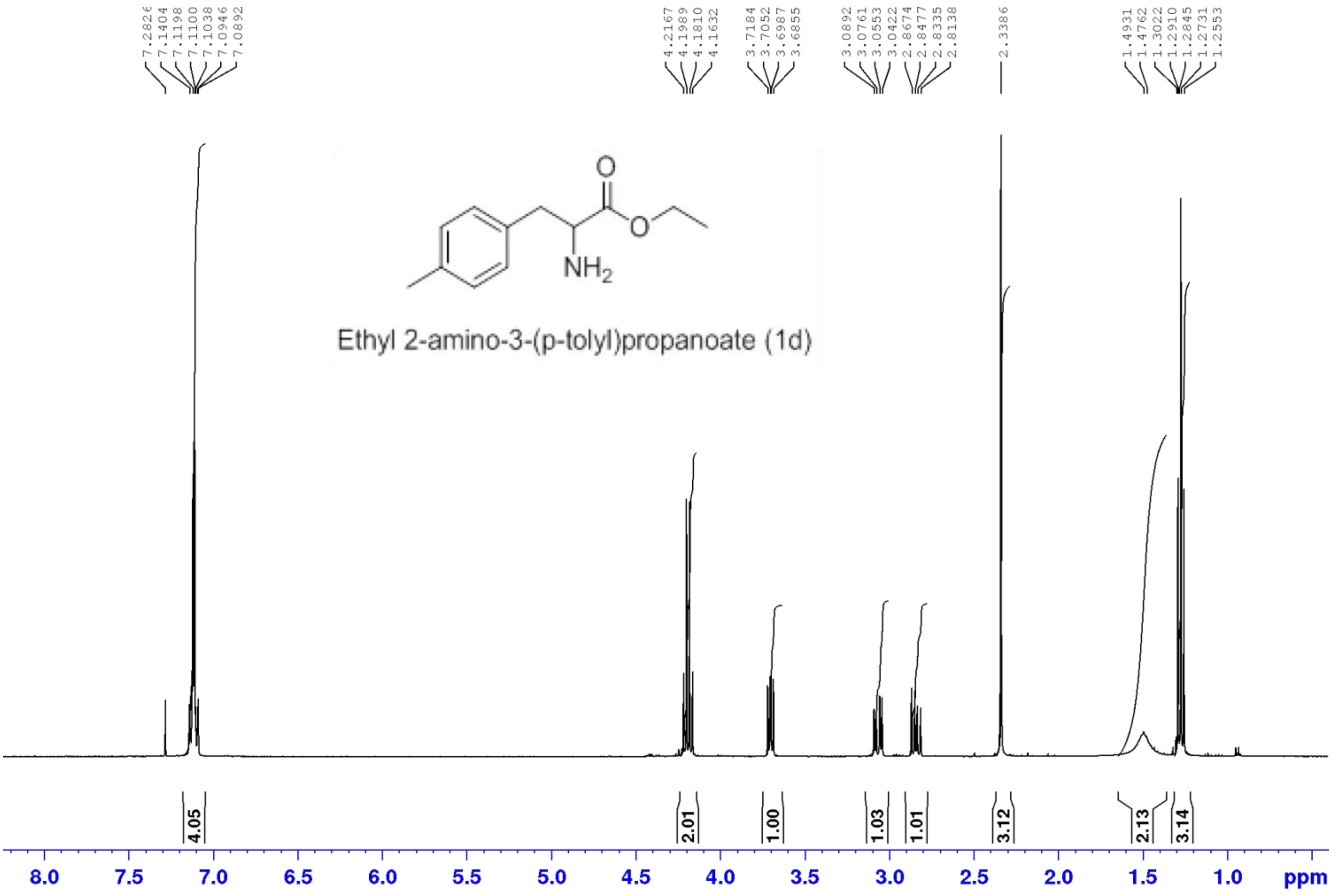
7.2826
7.1404
7.1198
7.1100
7.1038
7.0946
7.0892
4.2167
4.1989
4.1810
4.1632
3.7184
3.7052
3.6987
3.6855
3.0892
3.0761
3.0553
3.0422
2.8674
2.8477
2.8335
2.8138
2.3386
1.4931
1.4762
1.3022
1.2910
1.2845
1.2731
1.2553
NH2
Ethyl 2-amino-3-(p-tolyl)propanoate (1d)
4.05
2.01
1.00
1.03
1.01
3.12
2.13
3.14
ppm

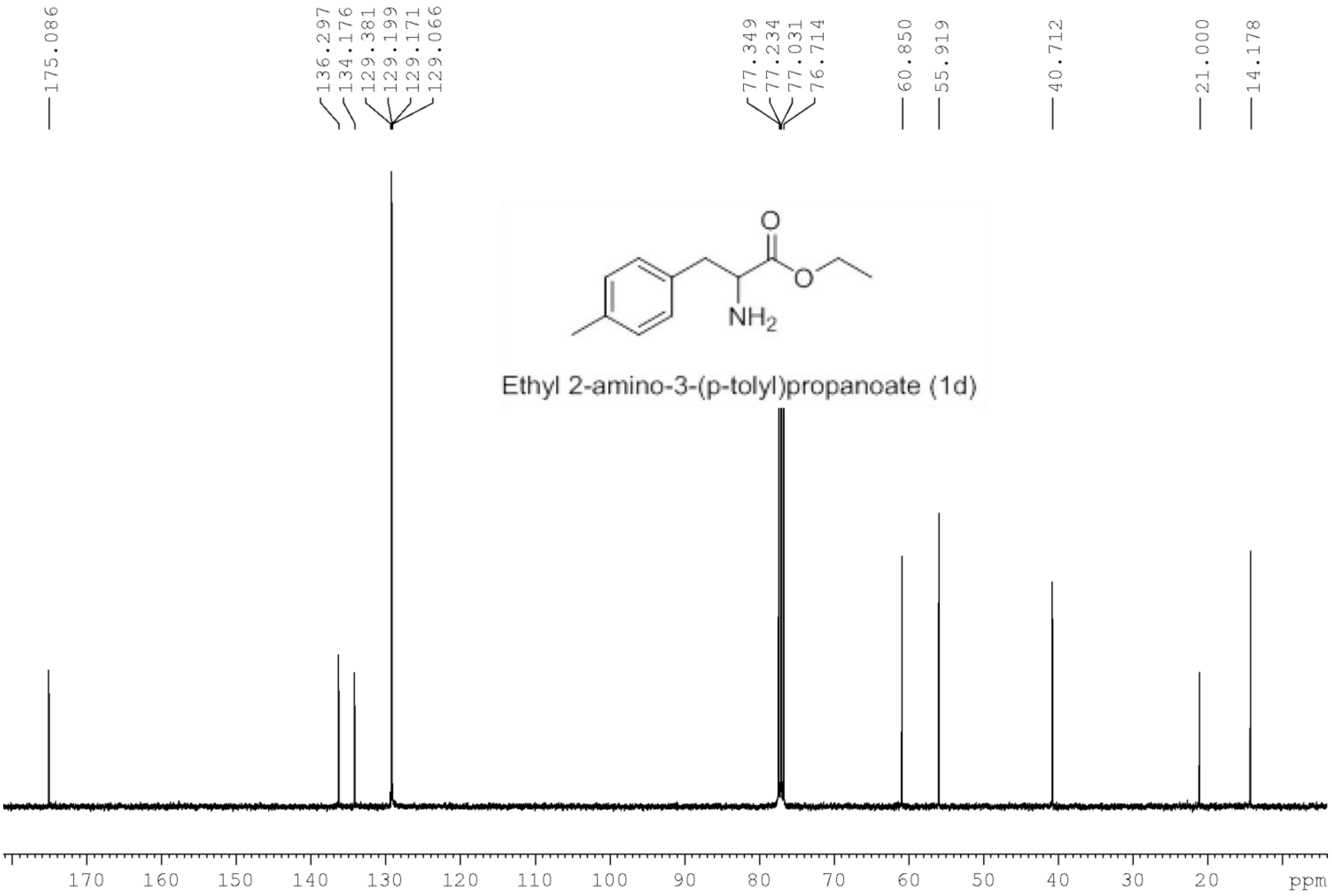
175.086
136.297
134.176
129.381
129.199
129.171
129.066
77.349
77.234
77.031
76.714
60.850
55.919
40.712
21.000
14.178
NH2
Ethyl 2-amino-3-(p-tolyl)propanoate (1d)
ppm

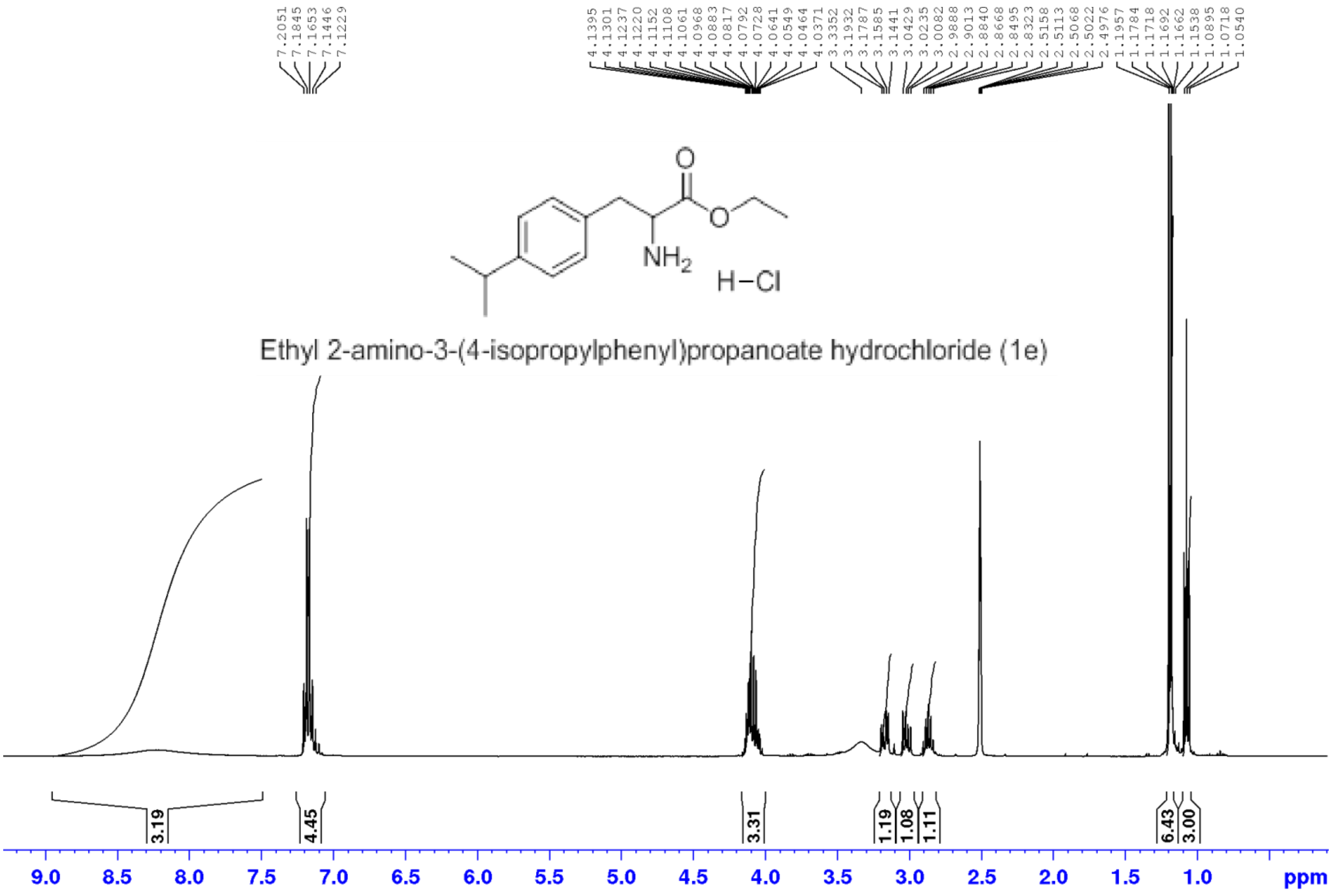
7.2051
7.1845
7.1653
7.1446
7.1229
4.1395
4.1301
4.1237
4.1220
4.1152
4.1108
4.1061
4.0968
4.0883
4.0817
4.0792
4.0728
4.0641
4.0549
4.0464
4.0371
3.3352
3.1932
3.1787
3.1585
3.1441
3.0429
3.0235
3.0082
2.9888
2.9013
2.8840
2.8668
2.8495
2.8323
2.5158
2.5113
2.5068
2.5022
2.4976
1.1957
1.1784
1.1718
1.1692
1.1662
1.1538
1.0895
1.0718
1.0540
O
O
NH2
H−Cl
Ethyl 2-amino-3-(4-isopropylphenyl)propanoate hydrochloride (1e)
3.19
4.45
3.31
1.19
1.08
1.11
6.43
3.00
9.0
8.5
8.0
7.5
7.0
6.5
6.0
5.5
5.0
4.5
4.0
3.5
3.0
2.5
2.0
1.5
1.0
ppm

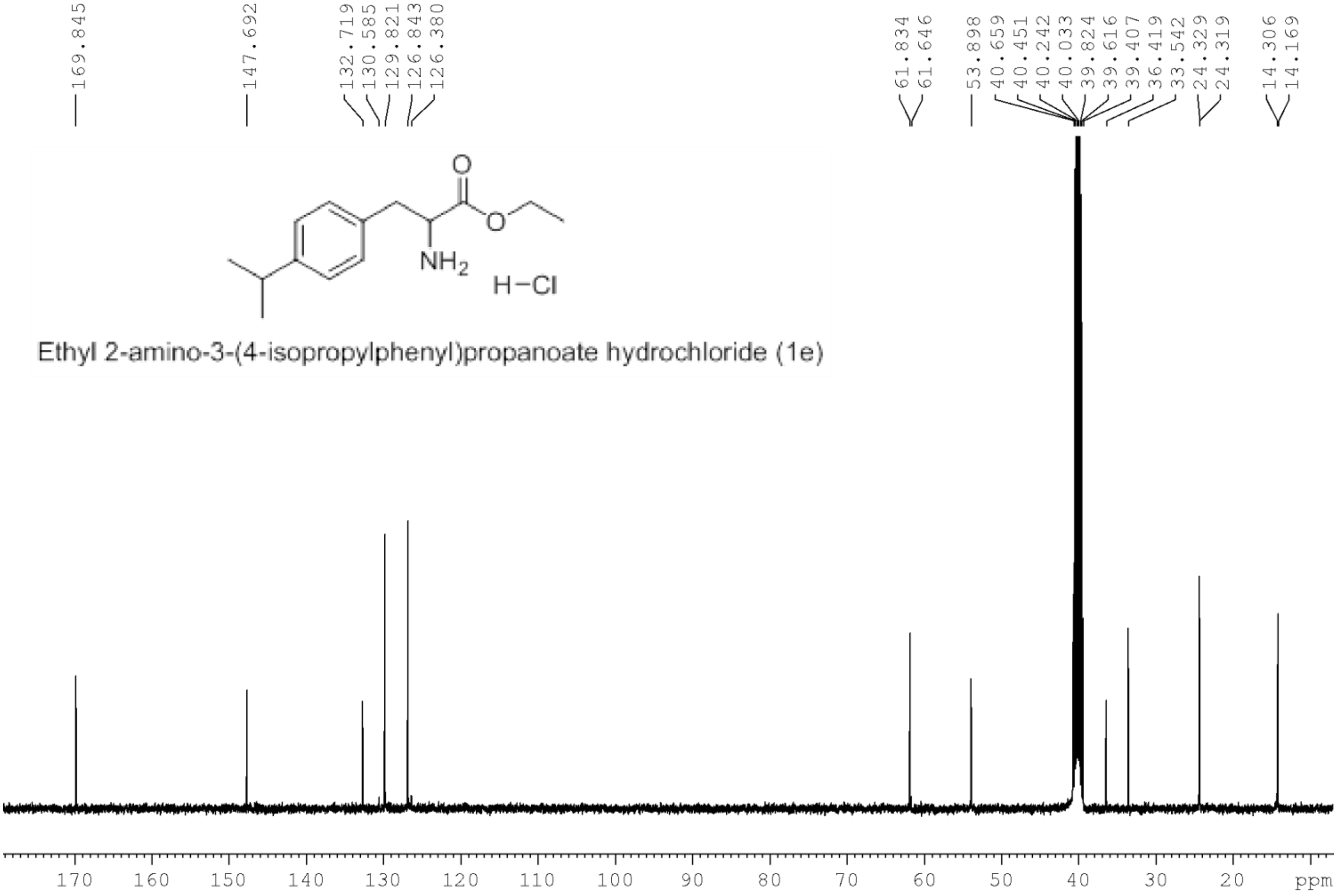
169.845
147.692
132.719
130.585
129.821
126.843
126.380
61.834
61.646
53.898
40.659
40.451
40.242
40.033
39.824
39.616
39.407
36.419
33.542
24.329
24.319
14.306
14.169
O
O
NH2
H−Cl
Ethyl 2-amino-3-(4-isopropylphenyl)propanoate hydrochloride (1e)
170
160
150
140
130
120
110
100
90
80
70
60
50
40
30
20
ppm

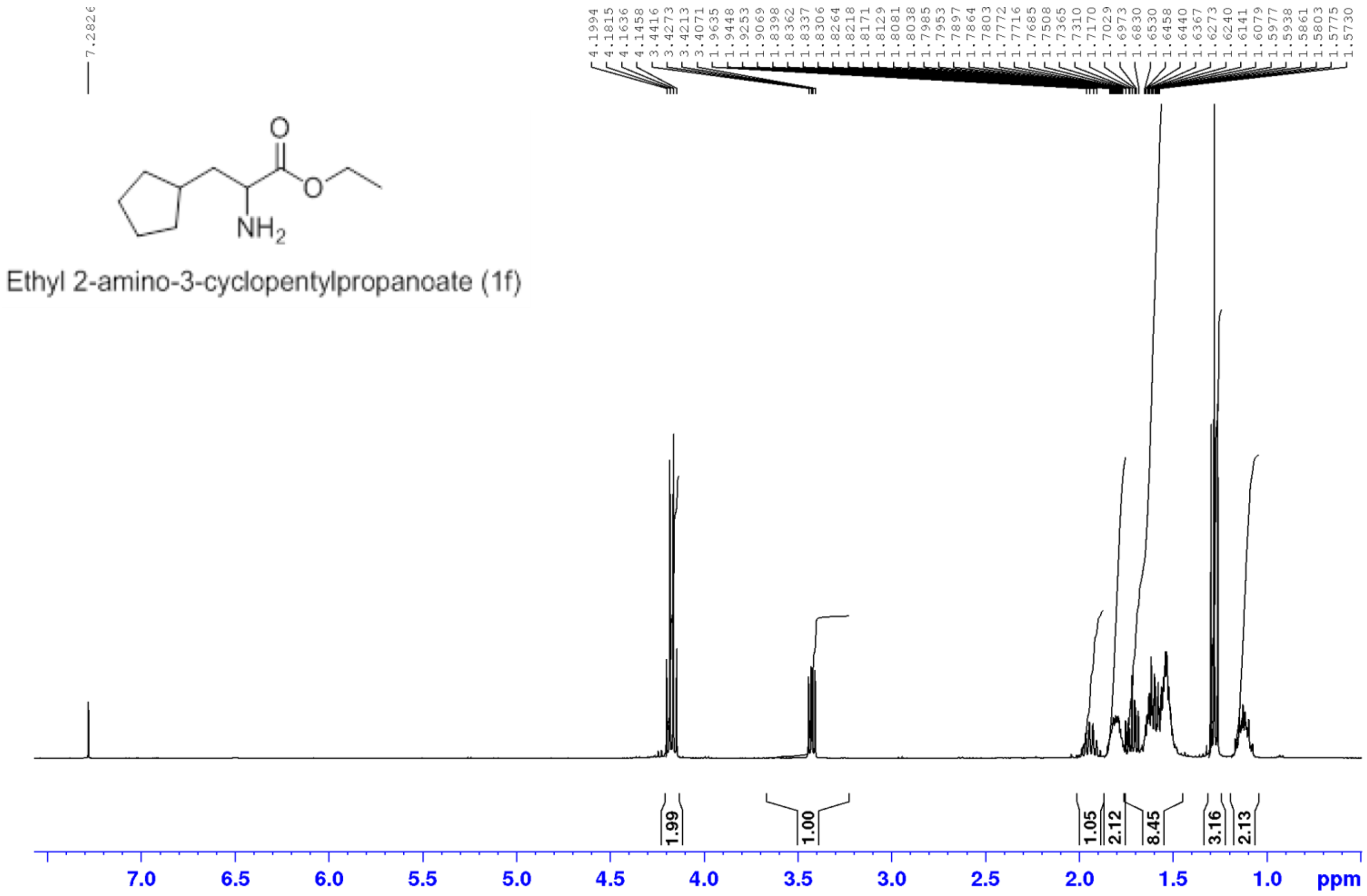
Ethyl 2-amino-3-cyclopentylpropanoate (1f)
1.99
1.00
1.05
2.12
8.45
3.16
2.13
ppm

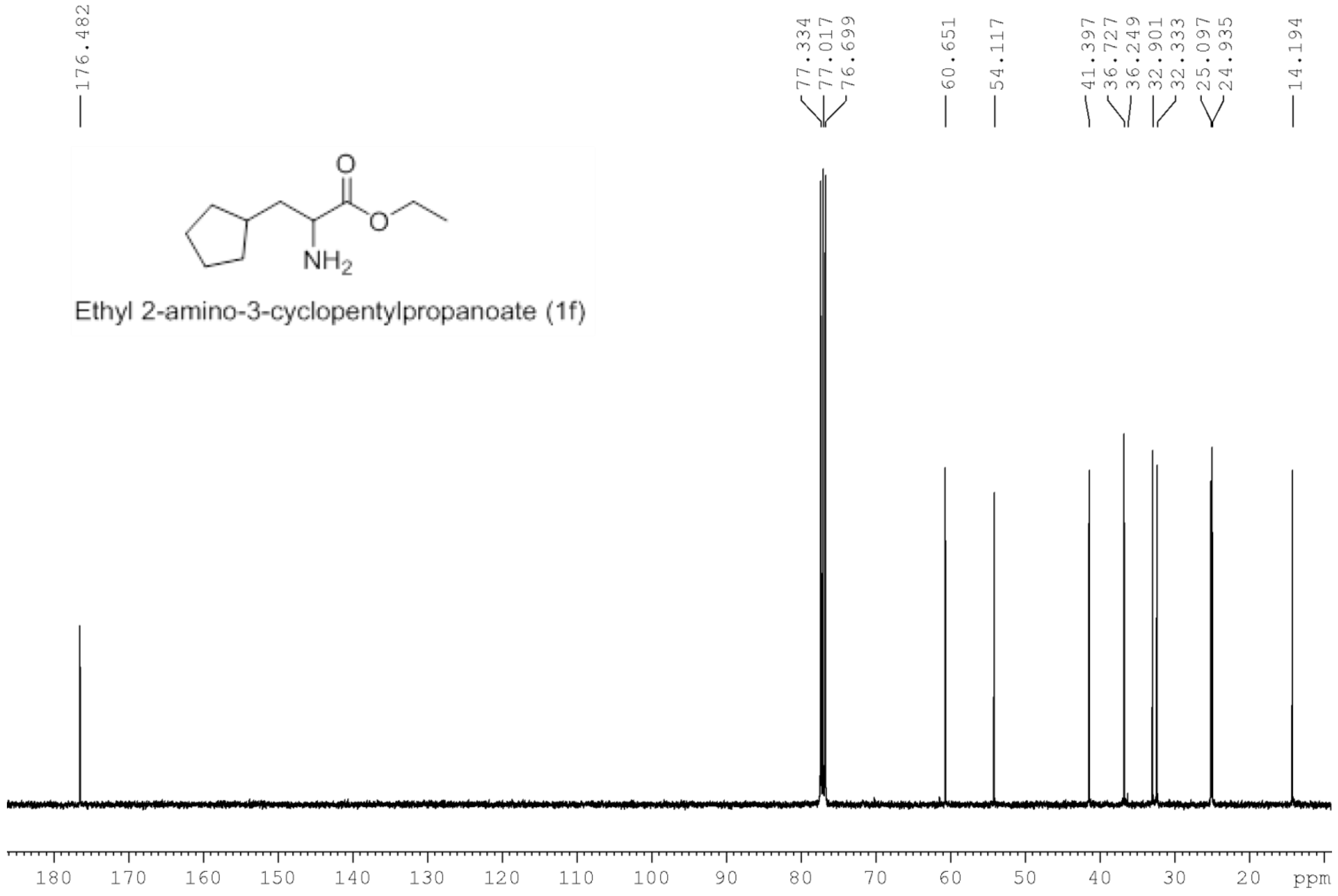
176.482
77.334
77.017
76.699
60.651
54.117
41.397
36.727
36.249
32.901
32.333
25.097
24.935
14.194
Ethyl 2-amino-3-cyclopentylpropanoate (1f)
ppm

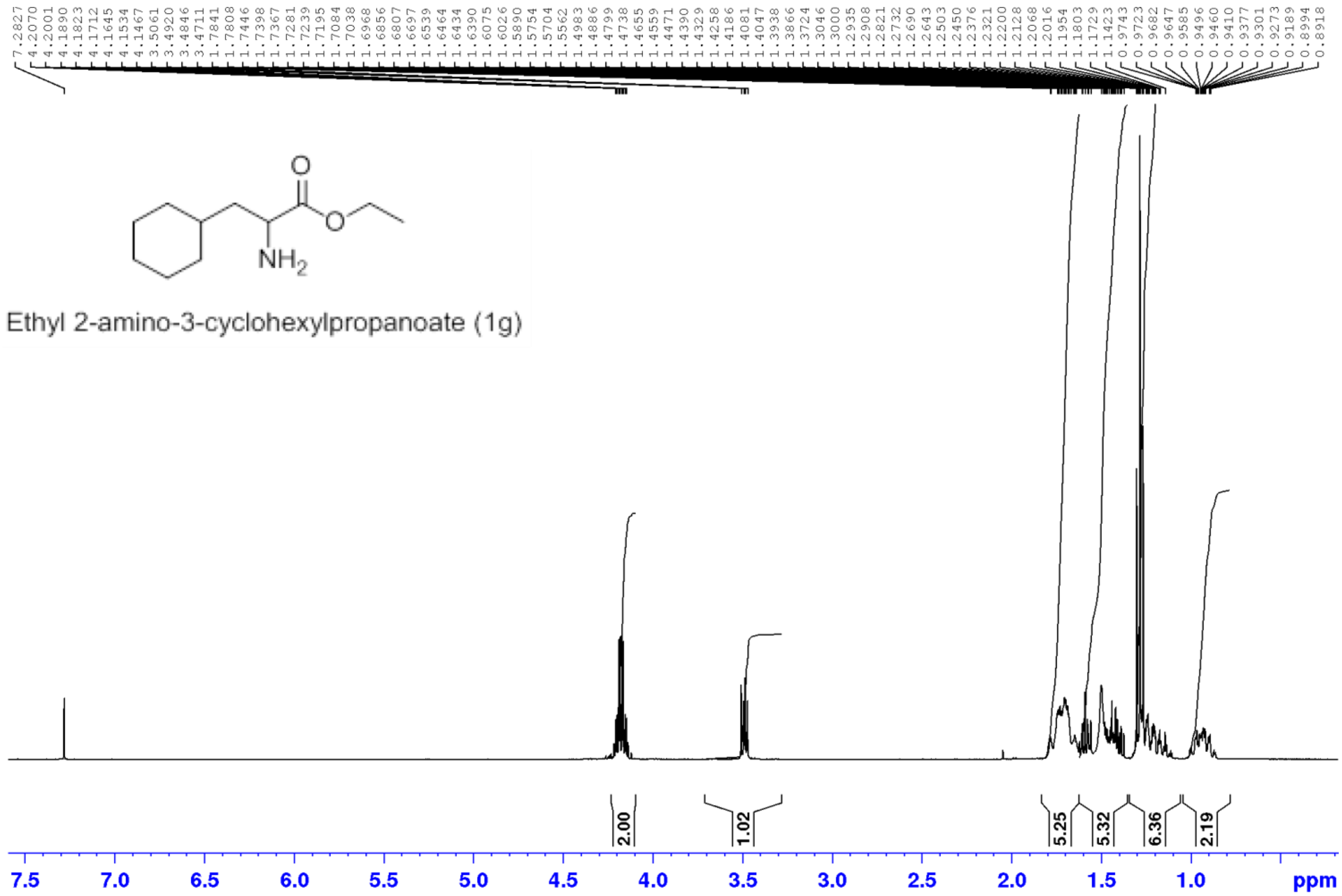

7.2827
4.2070
4.2001
4.1890
4.1823
4.1712
4.1645
4.1534
4.1467
3.5061
3.4920
3.4846
3.4711
1.7841
1.7808
1.7446
1.7398
1.7367
1.7281
1.7239
1.7195
1.7084
1.7038
1.6968
1.6856
1.6807
1.6697
1.6539
1.6464
1.6434
1.6390
1.6075
1.6026
1.5890
1.5754
1.5704
1.5562
1.4983
1.4886
1.4799
1.4738
1.4655
1.4559
1.4471
1.4390
1.4329
1.4258
1.4186
1.4081
1.4047
1.3938
1.3866
1.3724
1.3046
1.3000
1.2935
1.2908
1.2821
1.2732
1.2690
1.2643
1.2503
1.2450
1.2376
1.2321
1.2200
1.2128
1.2068
1.2016
1.1954
1.1803
1.1729
1.1423
0.9743
0.9723
0.9682
0.9647
0.9585
0.9496
0.9460
0.9410
0.9377
0.9301
0.9273
0.9189
0.8994
0.8918
O
O
NH2
Ethyl 2-amino-3-cyclohexylpropanoate (1g)
2.00
1.02
5.25
5.32
6.36
2.19
7.5
7.0
6.5
6.0
5.5
5.0
4.5
4.0
3.5
3.0
2.5
2.0
1.5
1.0
ppm

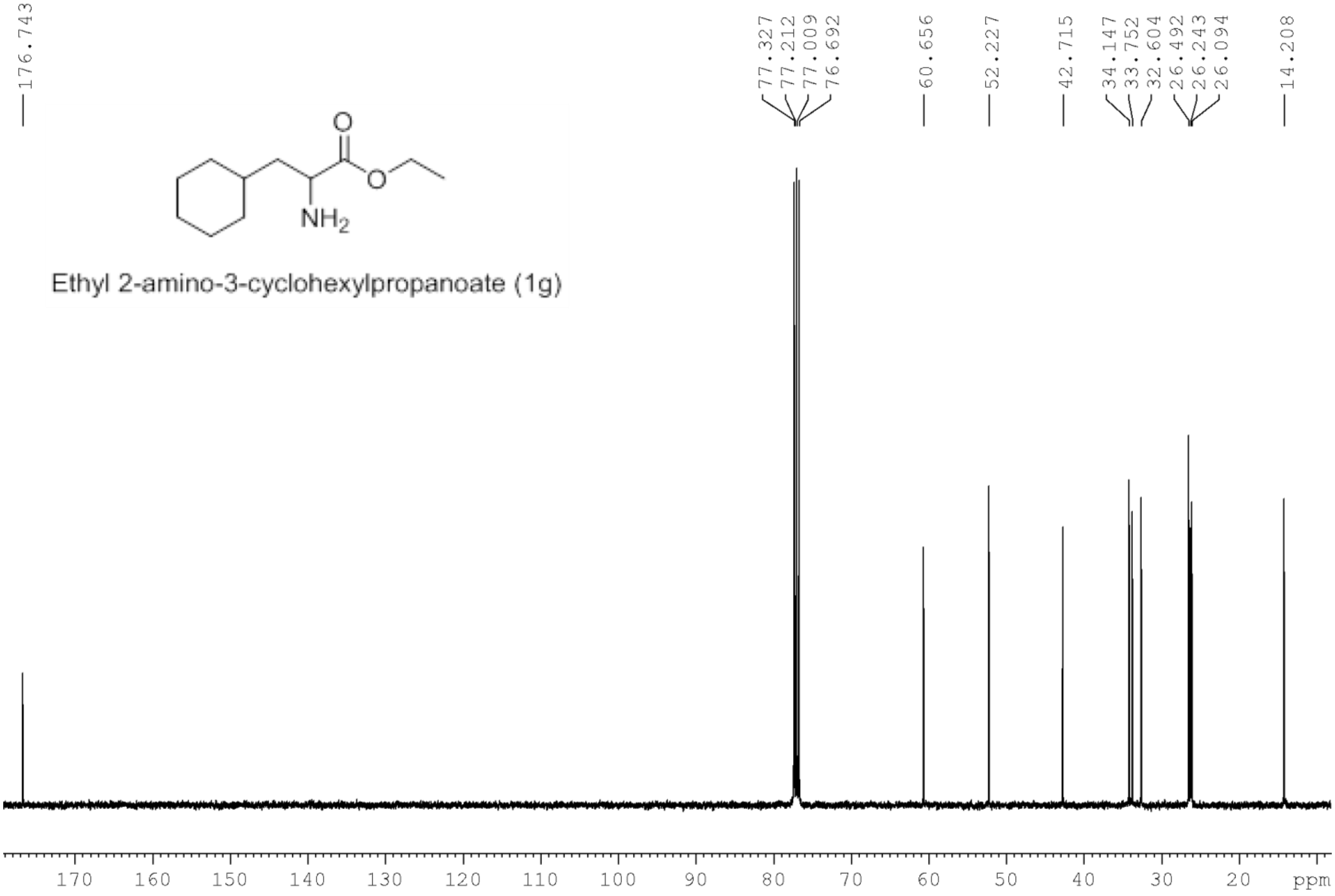

176.743
77.327
77.212
77.009
76.692
60.656
52.227
42.715
34.147
33.752
32.604
26.492
26.243
26.094
14.208
O
O
NH2
Ethyl 2-amino-3-cyclohexylpropanoate (1g)
170
160
150
140
130
120
110
100
90
80
70
60
50
40
30
20
ppm

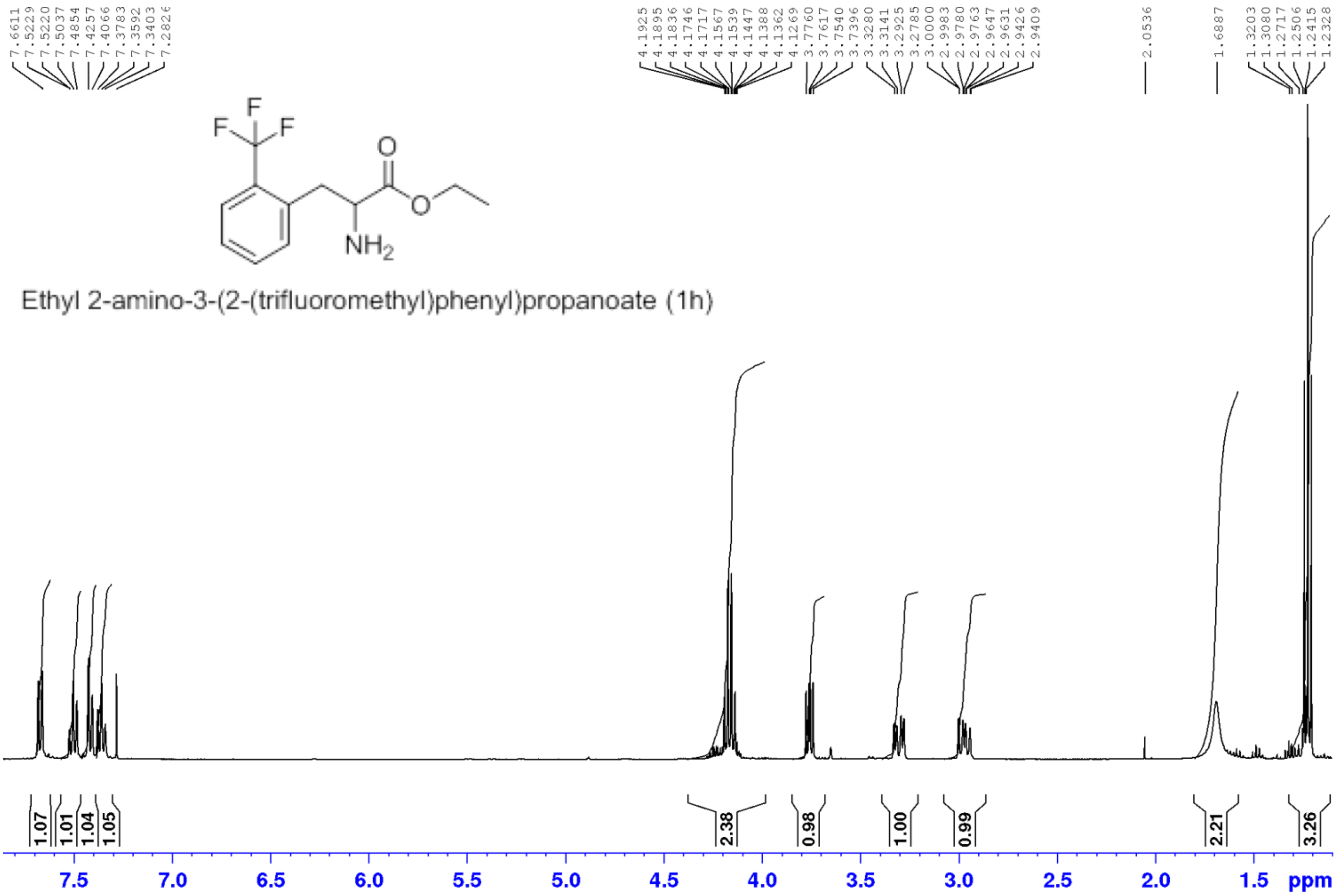

Ethyl 2-amino-3-(2-(trifluoromethyl)phenyl)propanoate (1h)

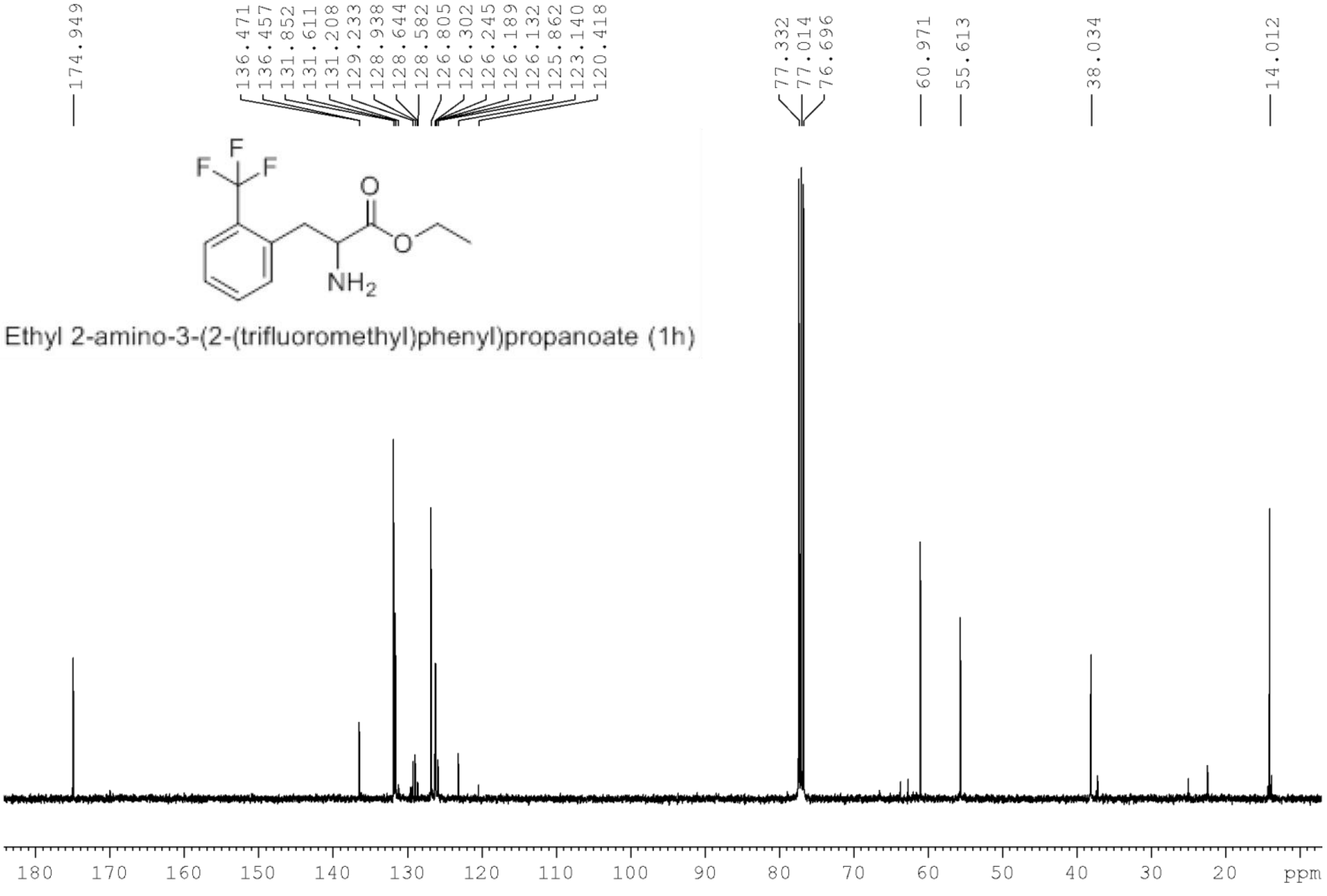

Ethyl 2-amino-3-(2-(trifluoromethyl)phenyl)propanoate (1h)

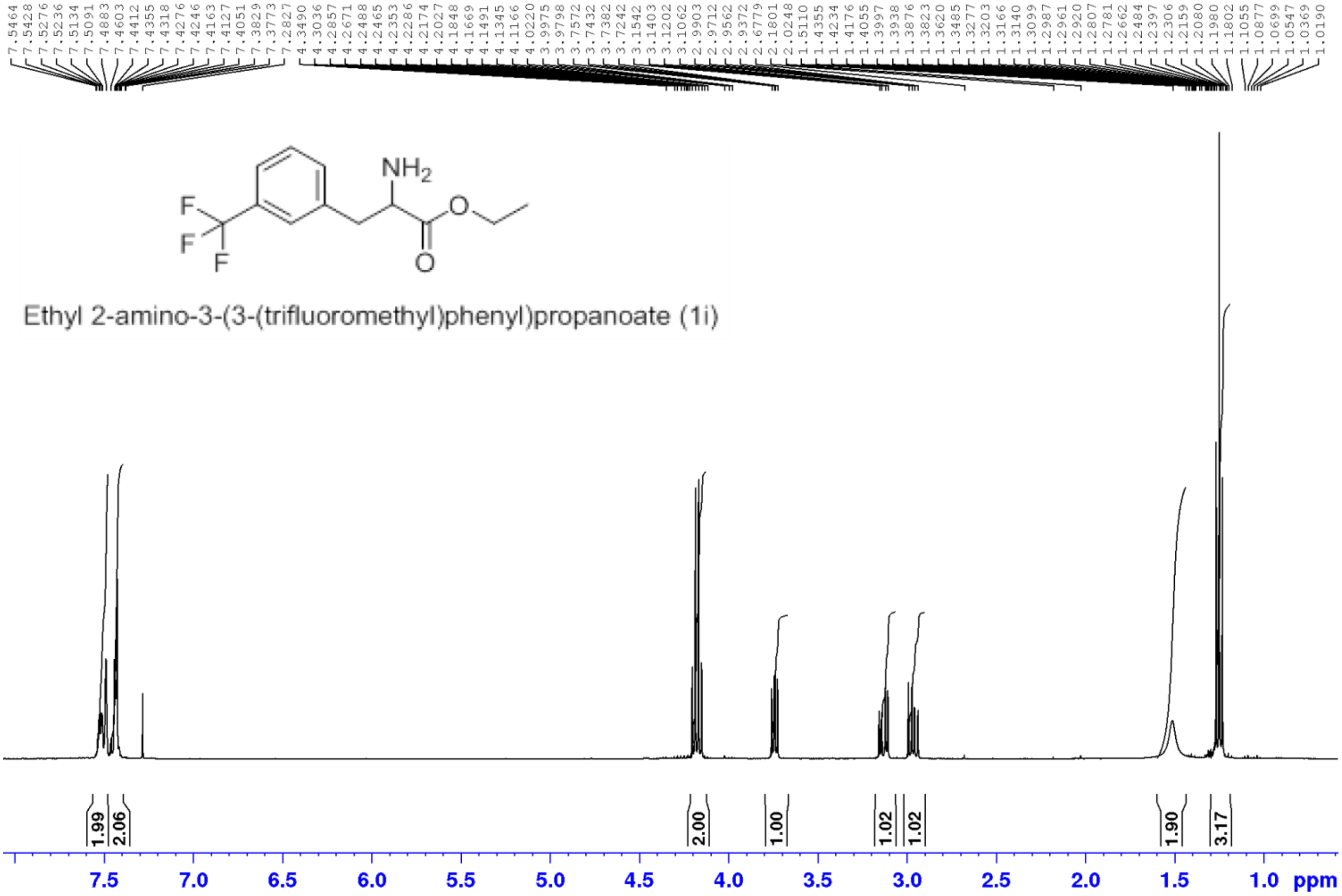
7.5464 7.5428 7.5276 7.5236 7.5134 7.5091 7.4883 7.4603 7.4412 7.4355 7.4318 7.4276 7.4246 7.4163 7.4127 7.4051 7.3829 7.3773 7.2827 4.3490 4.3036 4.2857 4.2671 4.2488 4.2465 4.2353 4.2286 4.2174 4.2027 4.1848 4.1669 4.1491 4.1345 4.1166 4.0220 3.9975 3.9798 3.7572 3.7432 3.7382 3.7242 3.1542 3.1403 3.1202 3.1062 2.9903 2.9712 2.9562 2.9372 2.6779 2.1801 2.0248 1.5110 1.4355 1.4234 1.4176 1.4055 1.3997 1.3938 1.3876 1.3823 1.3620 1.3485 1.3277 1.3203 1.3166 1.3140 1.3099 1.2987 1.2961 1.2920 1.2807 1.2781 1.2662 1.2484 1.2397 1.2306 1.2159 1.2080 1.1980 1.1802 1.1055 1.0877 1.0699 1.0547 1.0369 1.0190
NH2
F
F
F
O
O
Ethyl 2-amino-3-(3-(trifluoromethyl)phenyl)propanoate (1i)
1.99
2.06
2.00
1.00
1.02
1.02
1.90
3.17
7.5 7.0 6.5 6.0 5.5 5.0 4.5 4.0 3.5 3.0 2.5 2.0 1.5 1.0 ppm

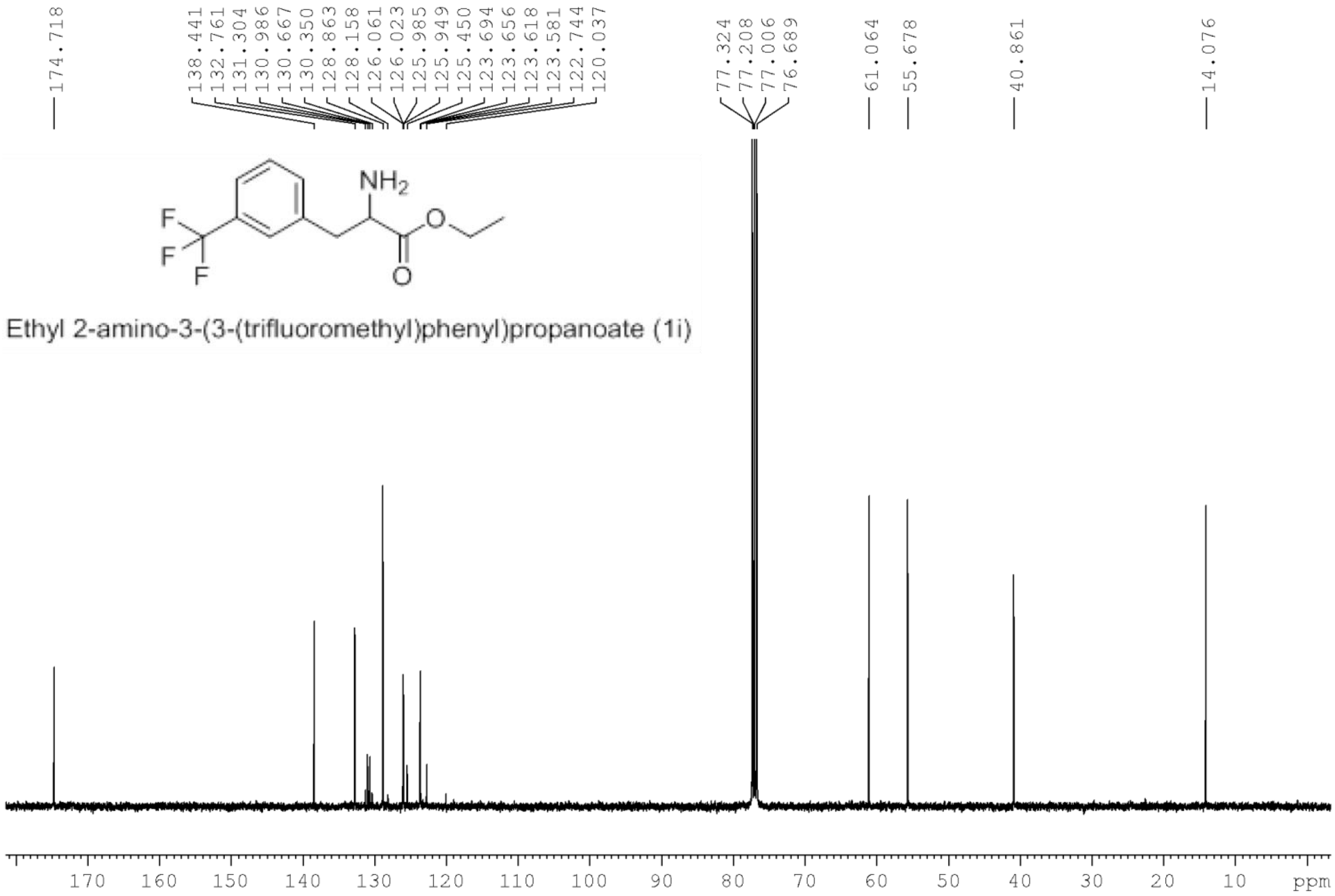
174.718
138.441 132.761 131.304 130.986 130.667 130.350 128.863 128.158 126.061 126.023 125.985 125.949 125.450 123.694 123.656 123.618 123.581 122.744 120.037
77.324 77.208 77.006 76.689
61.064
55.678
40.861
14.076
NH2
F
F
F
O
O
Ethyl 2-amino-3-(3-(trifluoromethyl)phenyl)propanoate (1i)
170 160 150 140 130 120 110 100 90 80 70 60 50 40 30 20 10 ppm

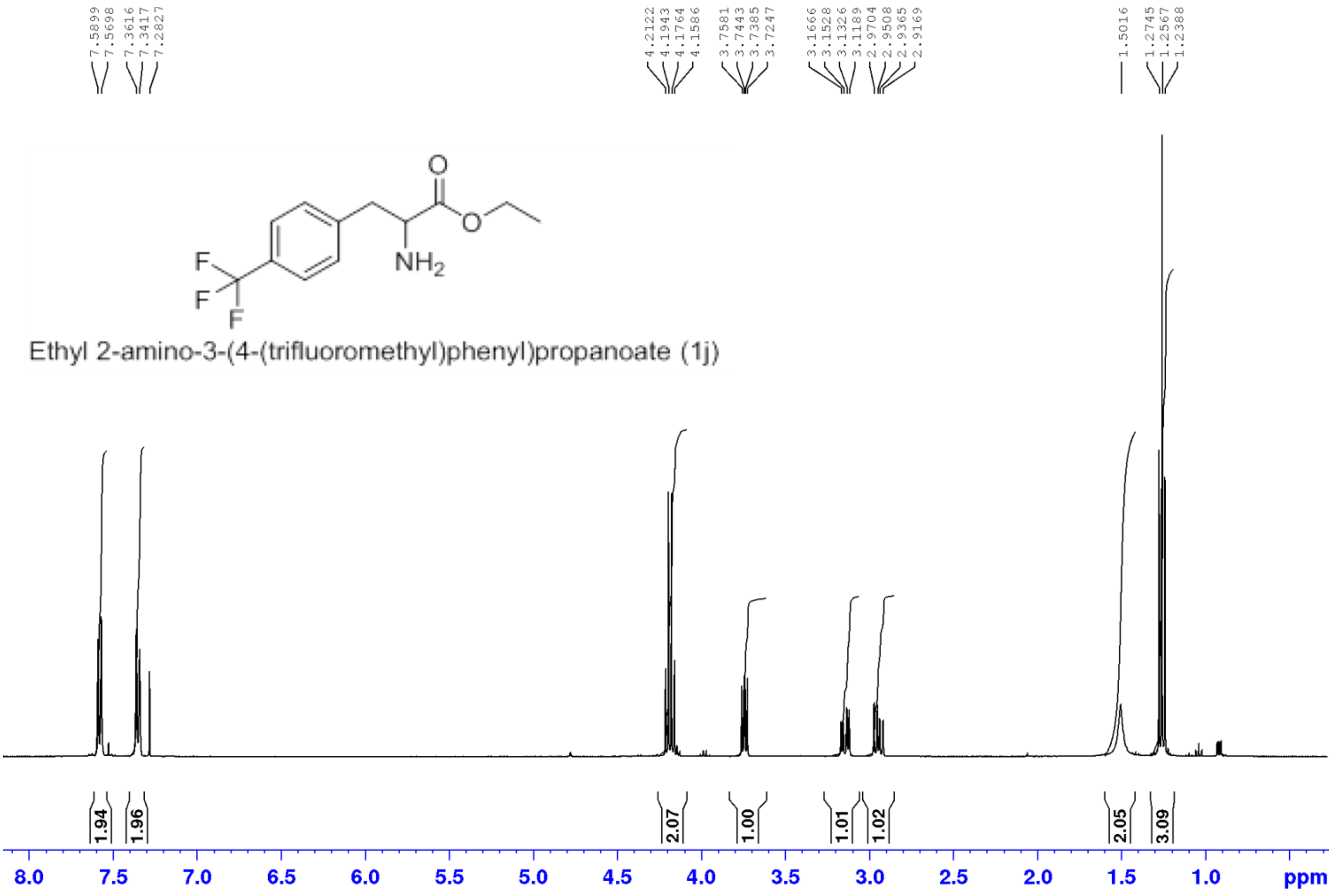
7.5899
7.5698
7.3616
7.3417
7.2827
4.2122
4.1943
4.1764
4.1586
3.7581
3.7443
3.7385
3.7247
3.1666
3.1528
3.1326
3.1189
2.9704
2.9508
2.9365
2.9169
1.5016
1.2745
1.2567
1.2388
F
F
F
O
O
NH2
Ethyl 2-amino-3-(4-(trifluoromethyl)phenyl)propanoate (1j)
1.94
1.96
2.07
1.00
1.01
1.02
2.05
3.09
8.0
7.5
7.0
6.5
6.0
5.5
5.0
4.5
4.0
3.5
3.0
2.5
2.0
1.5
1.0
ppm

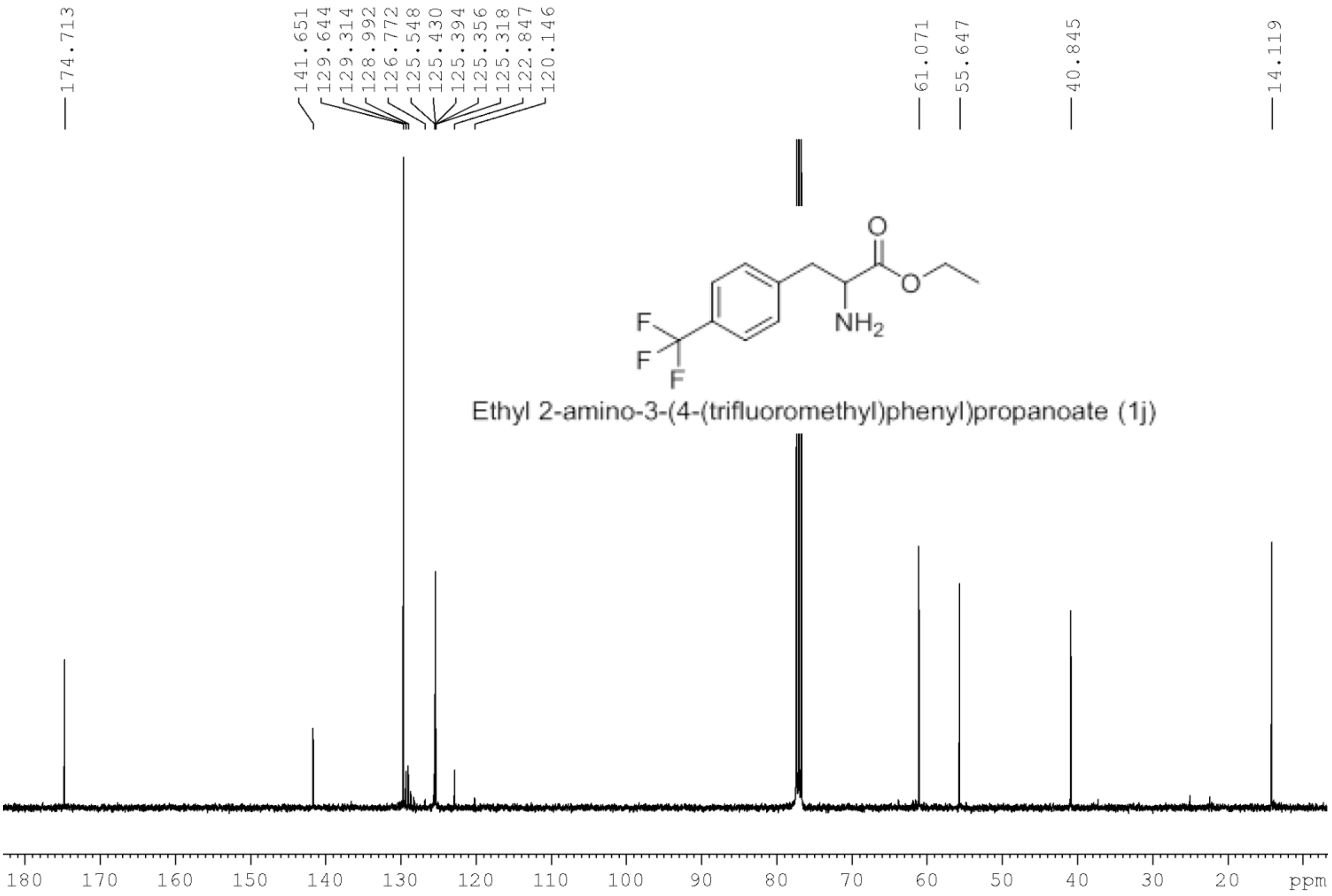
174.713
141.651
129.644
129.314
128.992
126.772
125.548
125.430
125.394
125.356
125.318
122.847
120.146
61.071
55.647
40.845
14.119
F
F
F
O
O
NH2
Ethyl 2-amino-3-(4-(trifluoromethyl)phenyl)propanoate (1j)
180
170
160
150
140
130
120
110
100
90
80
70
60
50
40
30
20
ppm

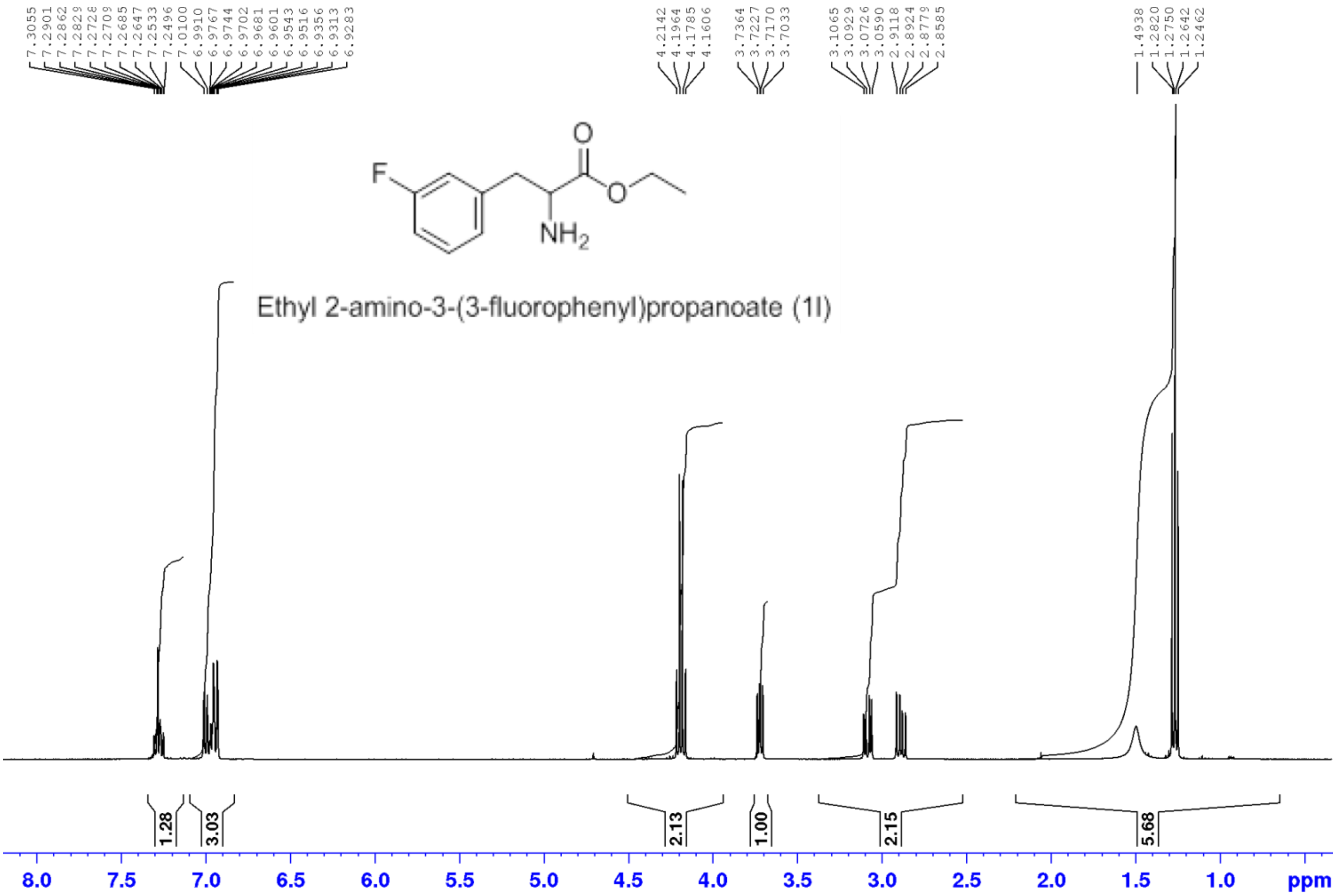
7.3055
7.2901
7.2862
7.2829
7.2728
7.2709
7.2685
7.2647
7.2533
7.2496
7.0100
6.9910
6.9767
6.9744
6.9702
6.9681
6.9601
6.9543
6.9516
6.9356
6.9313
6.9283
4.2142
4.1964
4.1785
4.1606
3.7364
3.7227
3.7170
3.7033
3.1065
3.0929
3.0726
3.0590
2.9118
2.8924
2.8779
2.8585
1.4938
1.2820
1.2750
1.2642
1.2462
F
O
O
NH2
Ethyl 2-amino-3-(3-fluorophenyl)propanoate (1l)
1.28
3.03
2.13
1.00
2.15
5.68
8.0 7.5 7.0 6.5 6.0 5.5 5.0 4.5 4.0 3.5 3.0 2.5 2.0 1.5 1.0 ppm

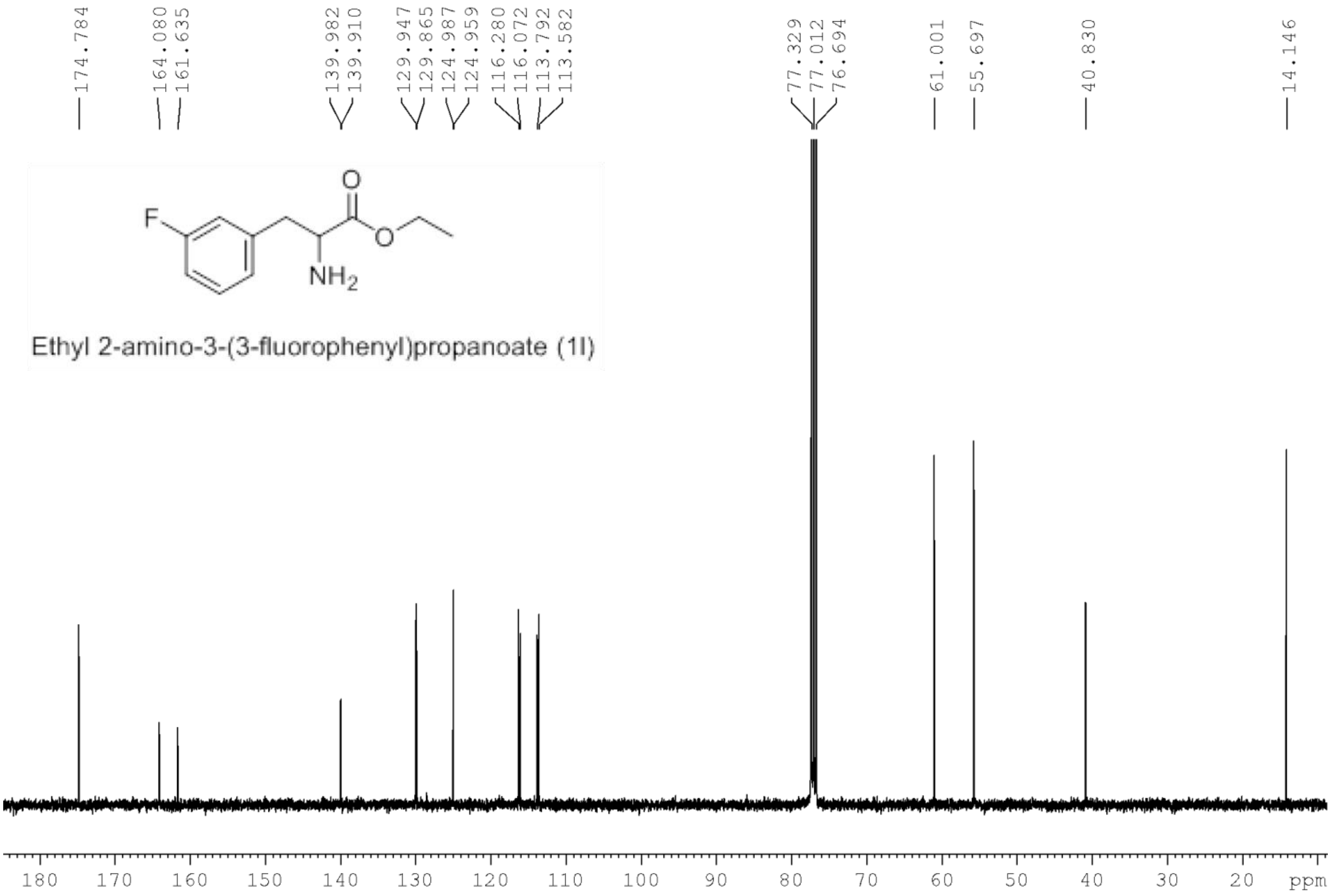
174.784
164.080
161.635
139.982
139.910
129.947
129.865
124.987
124.959
116.280
116.072
113.792
113.582
77.329
77.012
76.694
61.001
55.697
40.830
14.146
F
O
O
NH2
Ethyl 2-amino-3-(3-fluorophenyl)propanoate (1l)
180 170 160 150 140 130 120 110 100 90 80 70 60 50 40 30 20 ppm

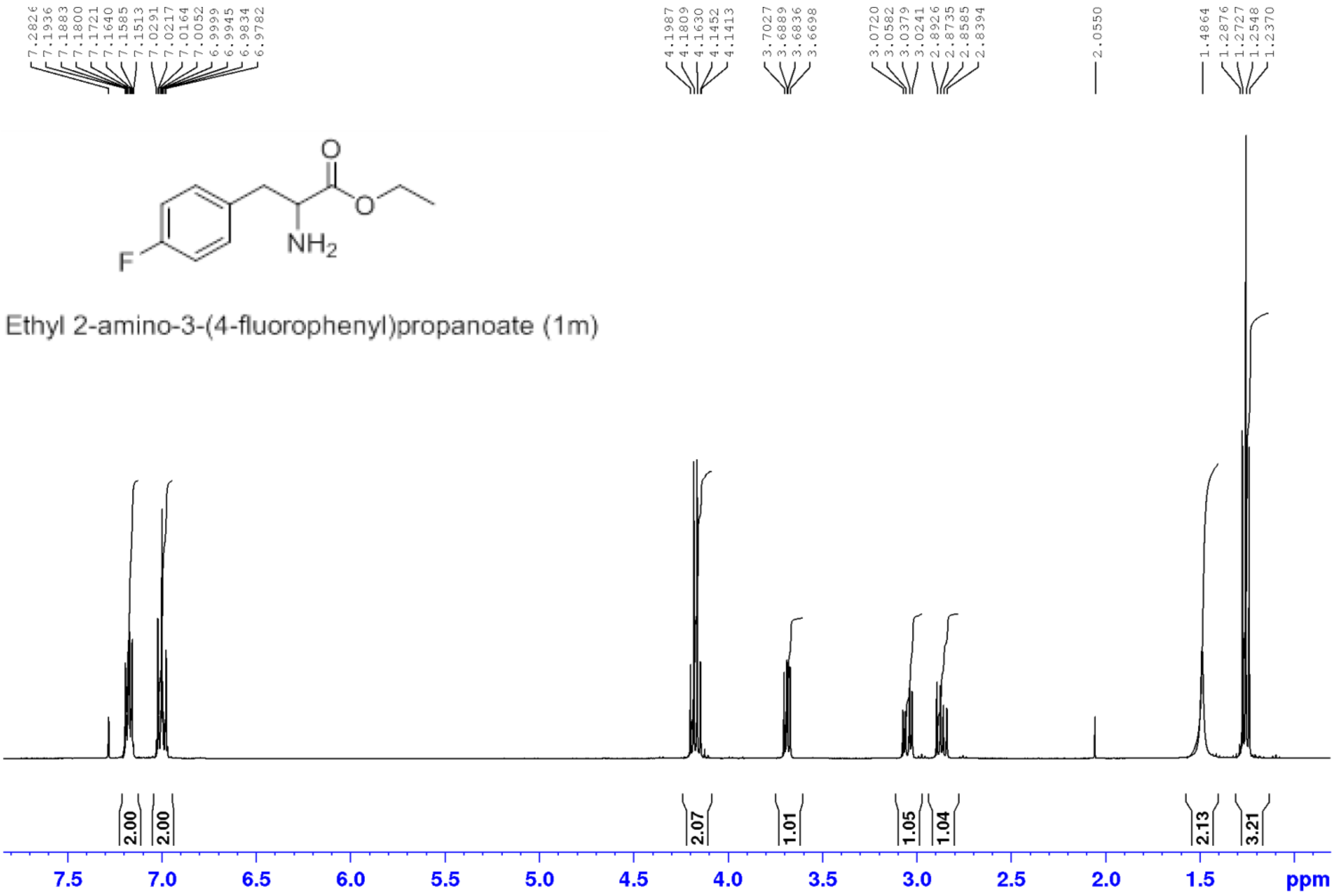
Ethyl 2-amino-3-(4-fluorophenyl)propanoate (1m)
7.2826
7.1936
7.1883
7.1800
7.1721
7.1640
7.1585
7.1513
7.0291
7.0217
7.0164
7.0052
6.9999
6.9945
6.9834
6.9782
4.1987
4.1809
4.1630
4.1452
4.1413
3.7027
3.6889
3.6836
3.6698
3.0720
3.0582
3.0379
3.0241
2.8926
2.8735
2.8585
2.8394
2.0550
1.4864
1.2876
1.2727
1.2548
1.2370
2.00
2.00
2.07
1.01
1.05
1.04
2.13
3.21
7.5
7.0
6.5
6.0
5.5
5.0
4.5
4.0
3.5
3.0
2.5
2.0
1.5
ppm

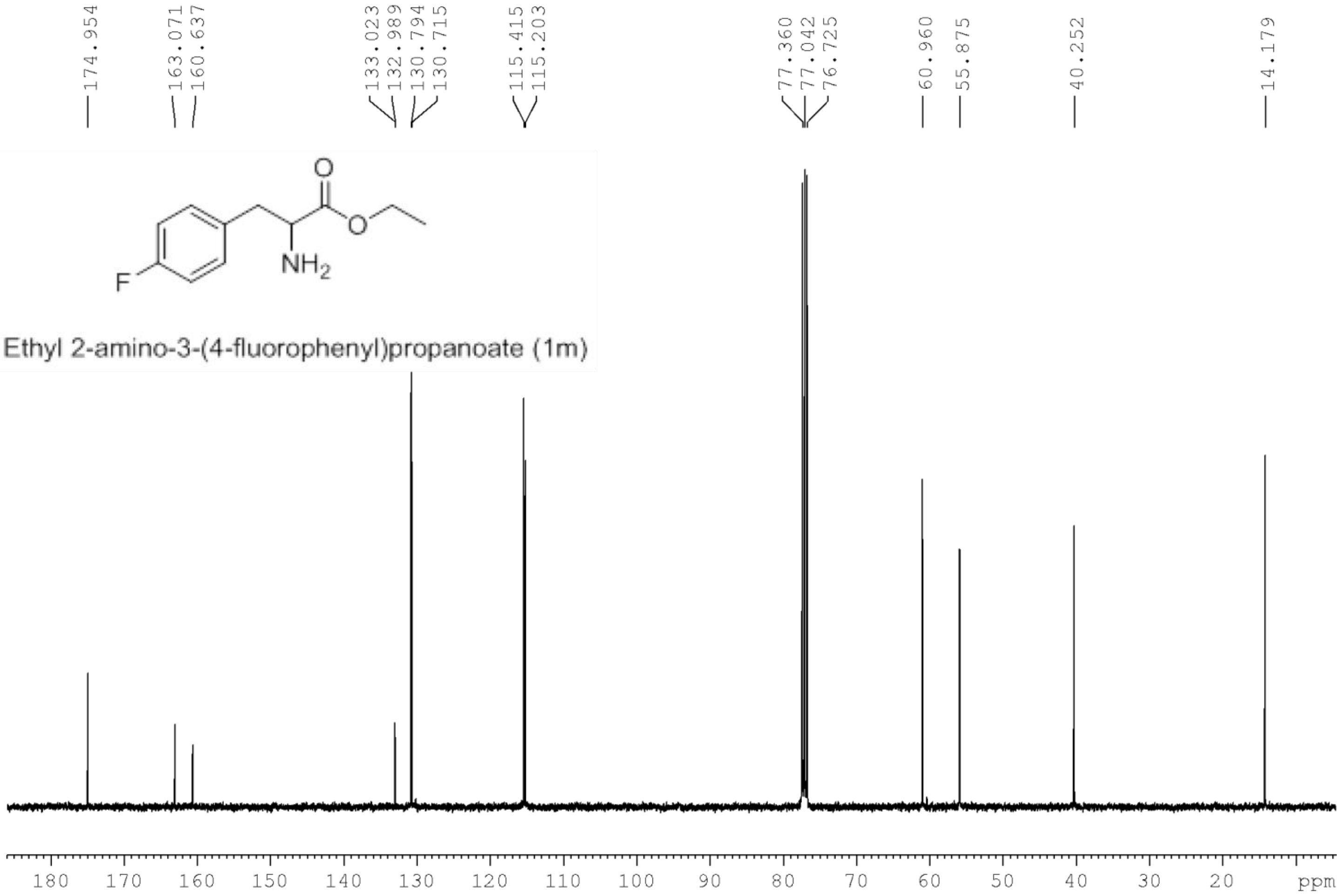
Ethyl 2-amino-3-(4-fluorophenyl)propanoate (1m)
174.954
163.071
160.637
133.023
132.989
130.794
130.715
115.415
115.203
77.360
77.042
76.725
60.960
55.875
40.252
14.179
180
170
160
150
140
130
120
110
100
90
80
70
60
50
40
30
20
ppm

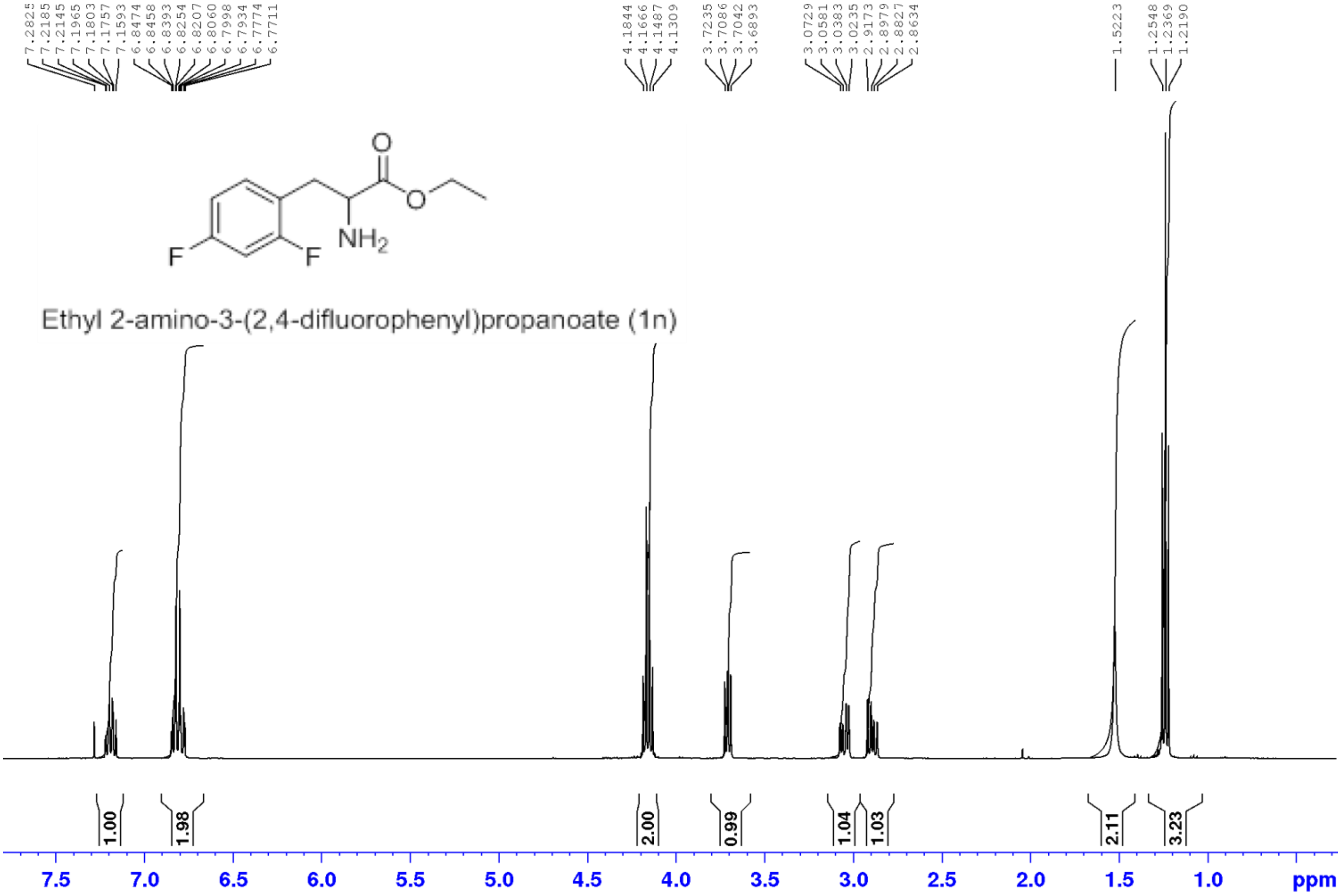
7.2825
7.2185
7.2145
7.1965
7.1803
7.1757
7.1593
6.8474
6.8458
6.8393
6.8254
6.8207
6.8060
6.7998
6.7934
6.7774
6.7711
4.1844
4.1666
4.1487
4.1309
3.7235
3.7086
3.7042
3.6893
3.0729
3.0581
3.0383
3.0235
2.9173
2.8979
2.8827
2.8634
1.5223
1.2548
1.2369
1.2190
O
F
F
NH2
Ethyl 2-amino-3-(2,4-difluorophenyl)propanoate (1n)
1.00
1.98
2.00
0.99
1.04
1.03
2.11
3.23
7.5
7.0
6.5
6.0
5.5
5.0
4.5
4.0
3.5
3.0
2.5
2.0
1.5
1.0
ppm

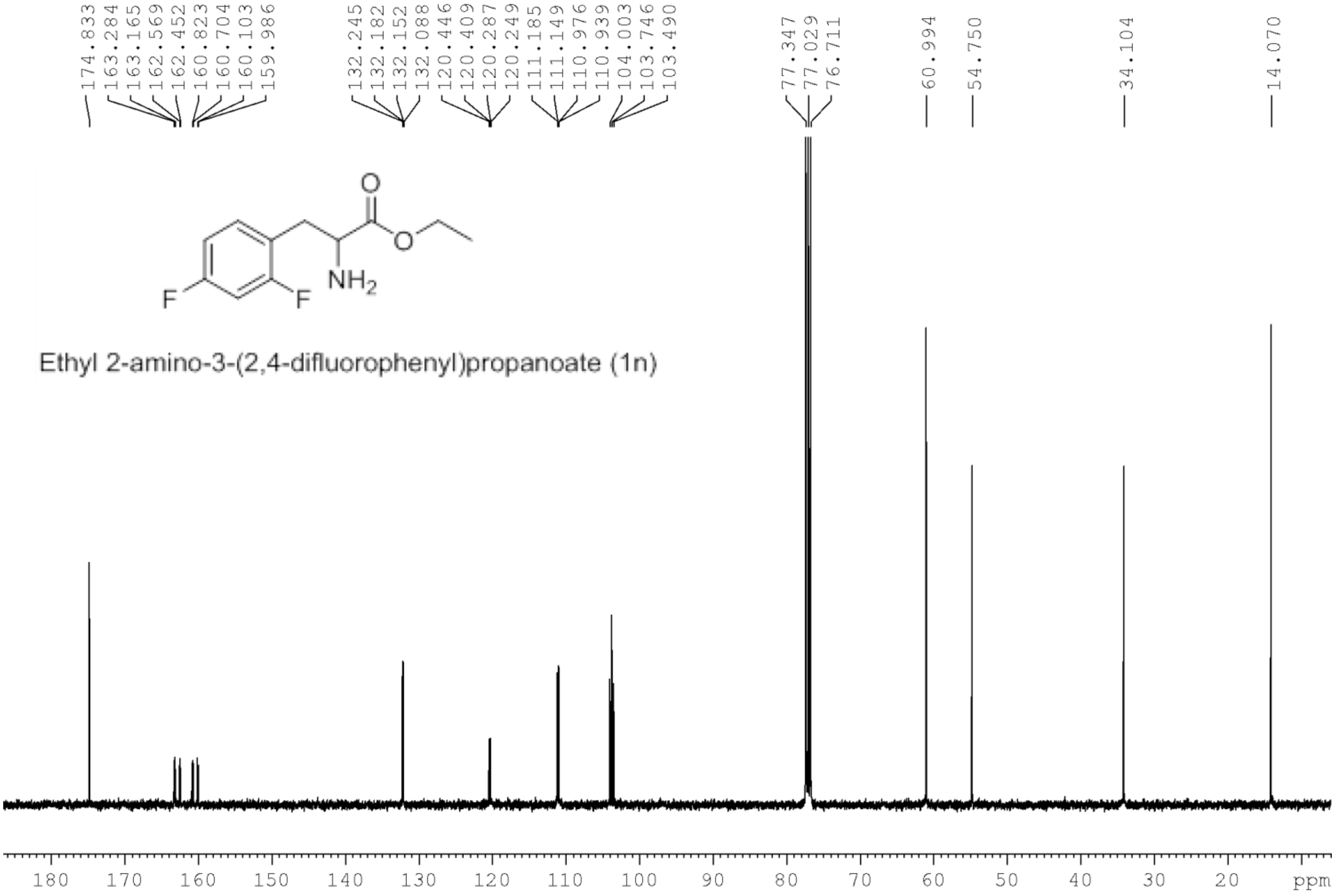
174.833
163.284
163.165
162.569
162.452
160.823
160.704
160.103
159.986
132.245
132.182
132.152
132.088
120.446
120.409
120.287
120.249
111.185
111.149
110.976
110.939
104.003
103.746
103.490
77.347
77.029
76.711
60.994
54.750
34.104
14.070
O
F
F
NH2
Ethyl 2-amino-3-(2,4-difluorophenyl)propanoate (1n)
180
170
160
150
140
130
120
110
100
90
80
70
60
50
40
30
20
ppm

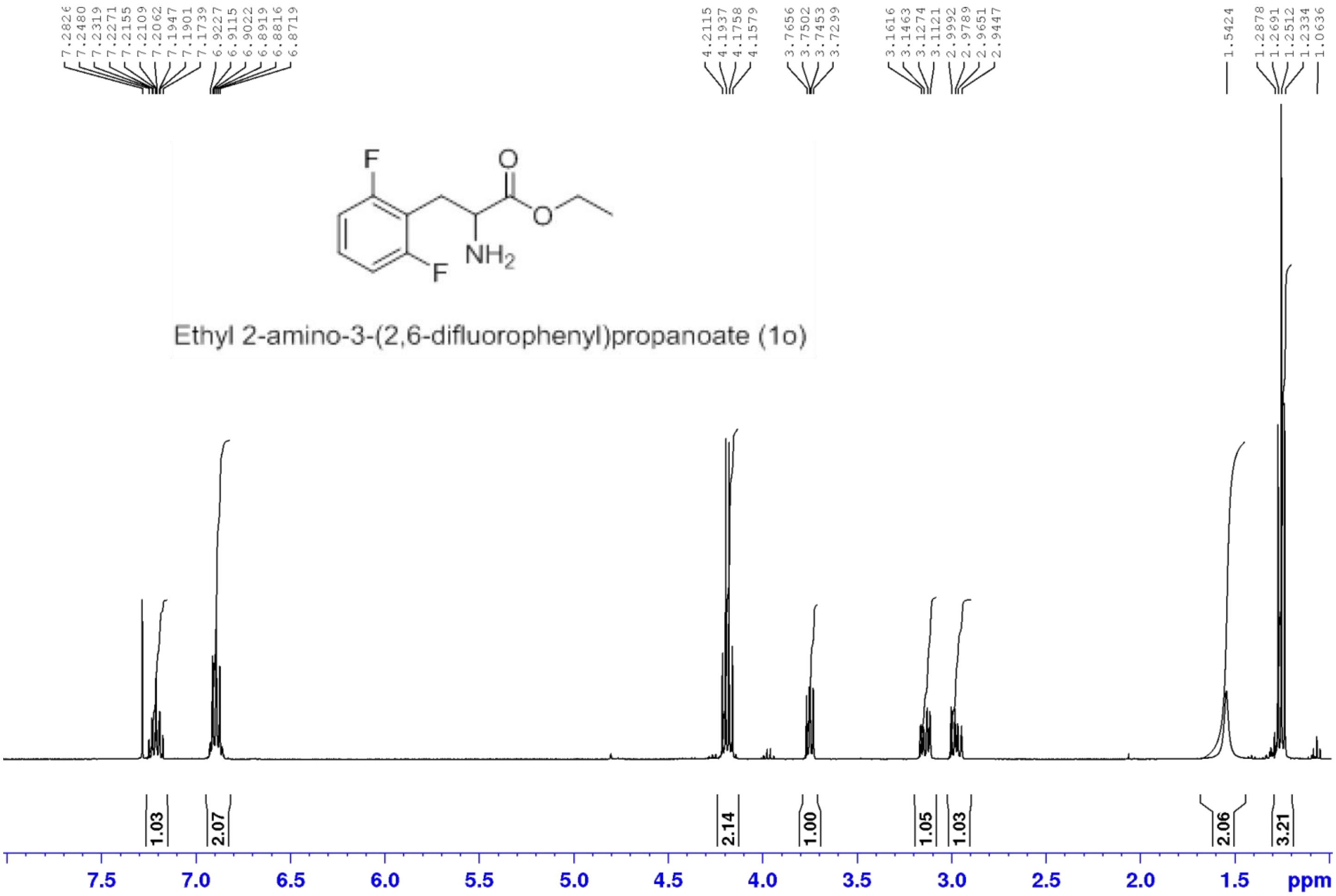
7.2826
7.2480
7.2319
7.2271
7.2155
7.2109
7.2062
7.1947
7.1901
7.1739
6.9227
6.9115
6.9022
6.8919
6.8816
6.8719
4.2115
4.1937
4.1758
4.1579
3.7656
3.7502
3.7453
3.7299
3.1616
3.1463
3.1274
3.1121
2.9992
2.9789
2.9651
2.9447
1.5424
1.2878
1.2691
1.2512
1.2334
1.0636
F
O
O
NH2
F
Ethyl 2-amino-3-(2,6-difluorophenyl)propanoate (1o)
1.03
2.07
2.14
1.00
1.05
1.03
2.06
3.21
7.5
7.0
6.5
6.0
5.5
5.0
4.5
4.0
3.5
3.0
2.5
2.0
1.5
ppm

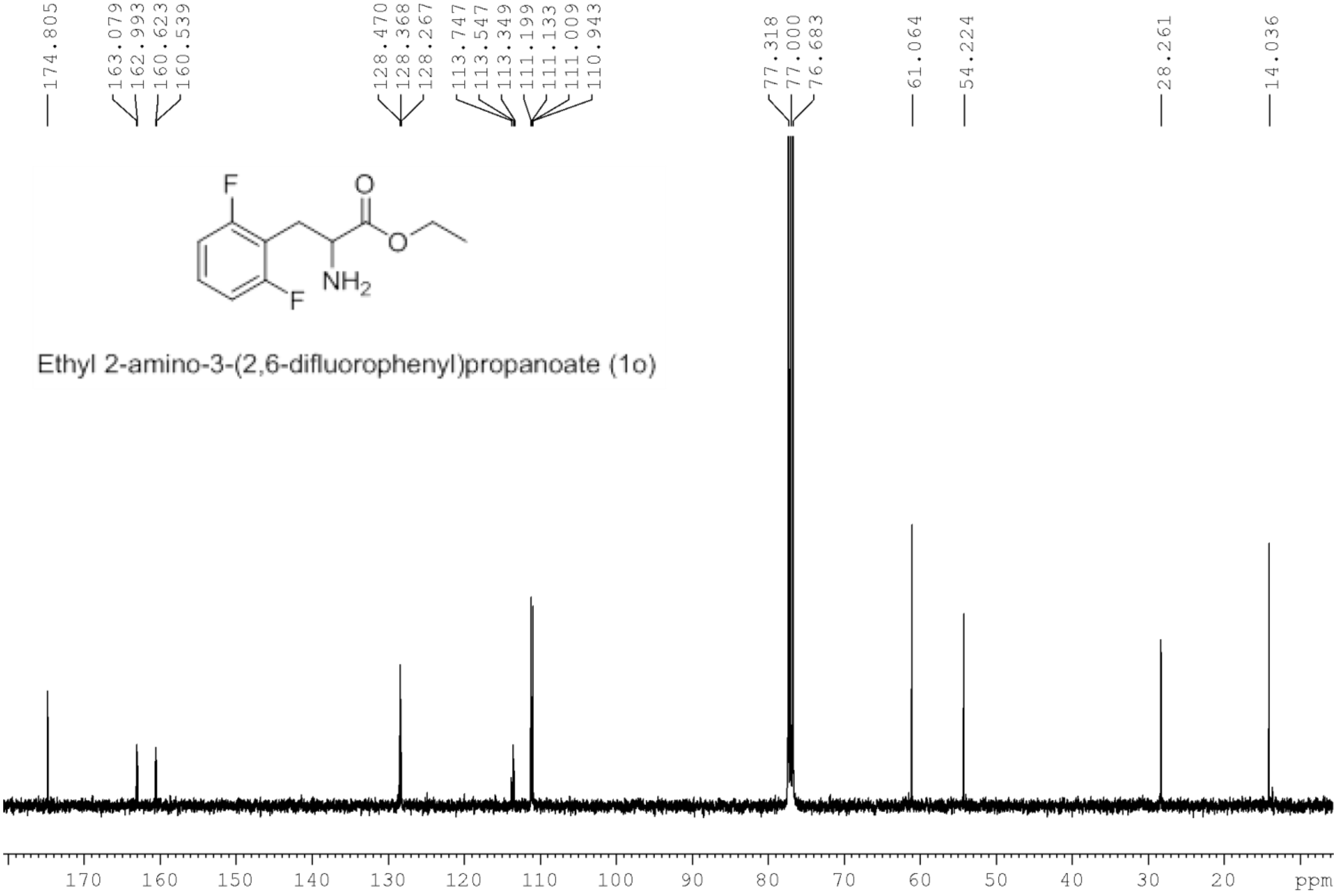
174.805
163.079
162.993
160.623
160.539
128.470
128.368
128.267
113.747
113.547
113.349
111.199
111.133
111.009
110.943
77.318
77.000
76.683
61.064
54.224
28.261
14.036
F
O
O
NH2
F
Ethyl 2-amino-3-(2,6-difluorophenyl)propanoate (1o)
170
160
150
140
130
120
110
100
90
80
70
60
50
40
30
20
ppm

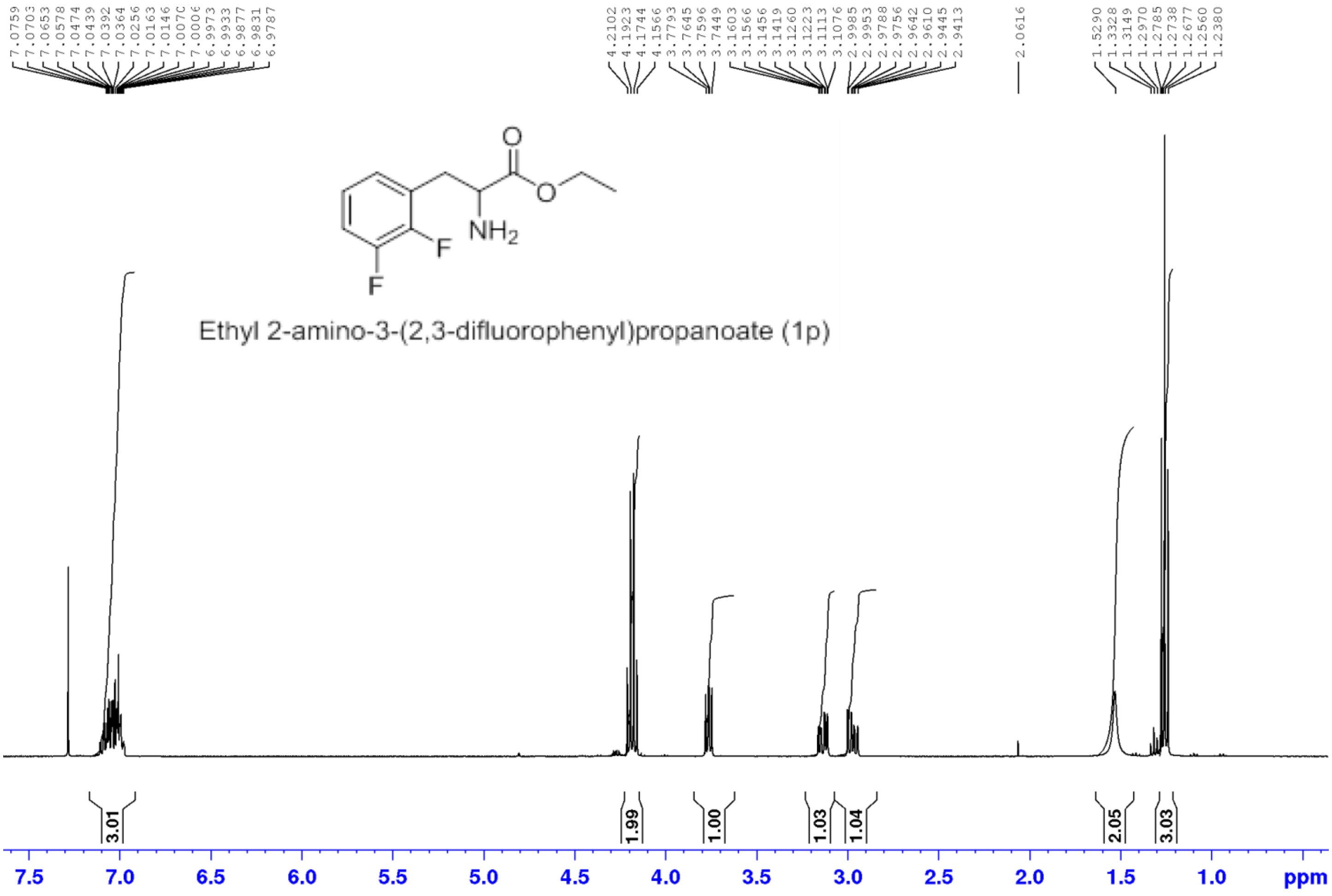
Ethyl 2-amino-3-(2,3-difluorophenyl)propanoate (1p)
7.5
7.0
6.5
6.0
5.5
5.0
4.5
4.0
3.5
3.0
2.5
2.0
1.5
1.0
ppm
3.01
1.99
1.00
1.03
1.04
2.05
3.03

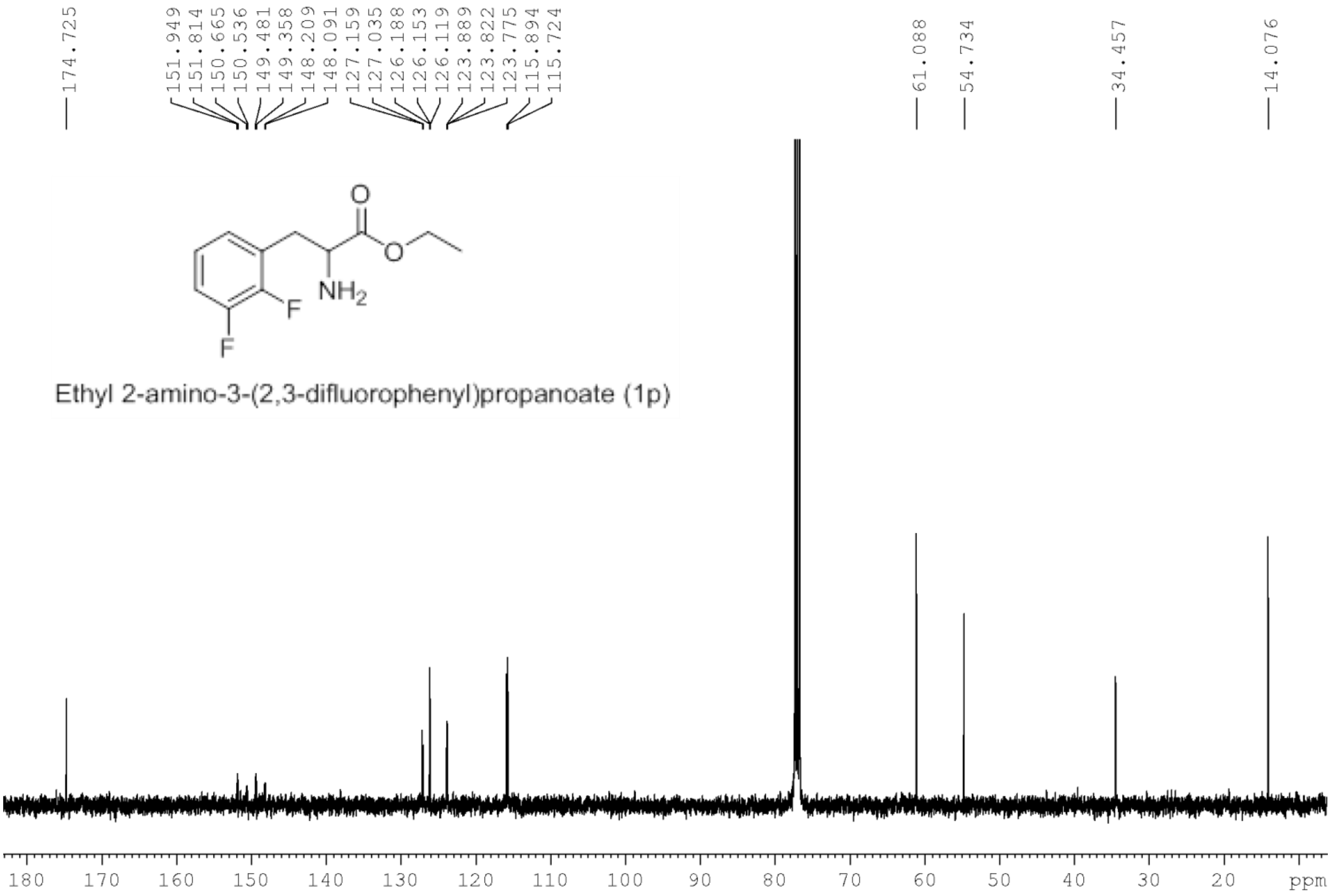
Ethyl 2-amino-3-(2,3-difluorophenyl)propanoate (1p)
174.725
61.088
54.734
34.457
14.076
180
170
160
150
140
130
120
110
100
90
80
70
60
50
40
30
20
ppm

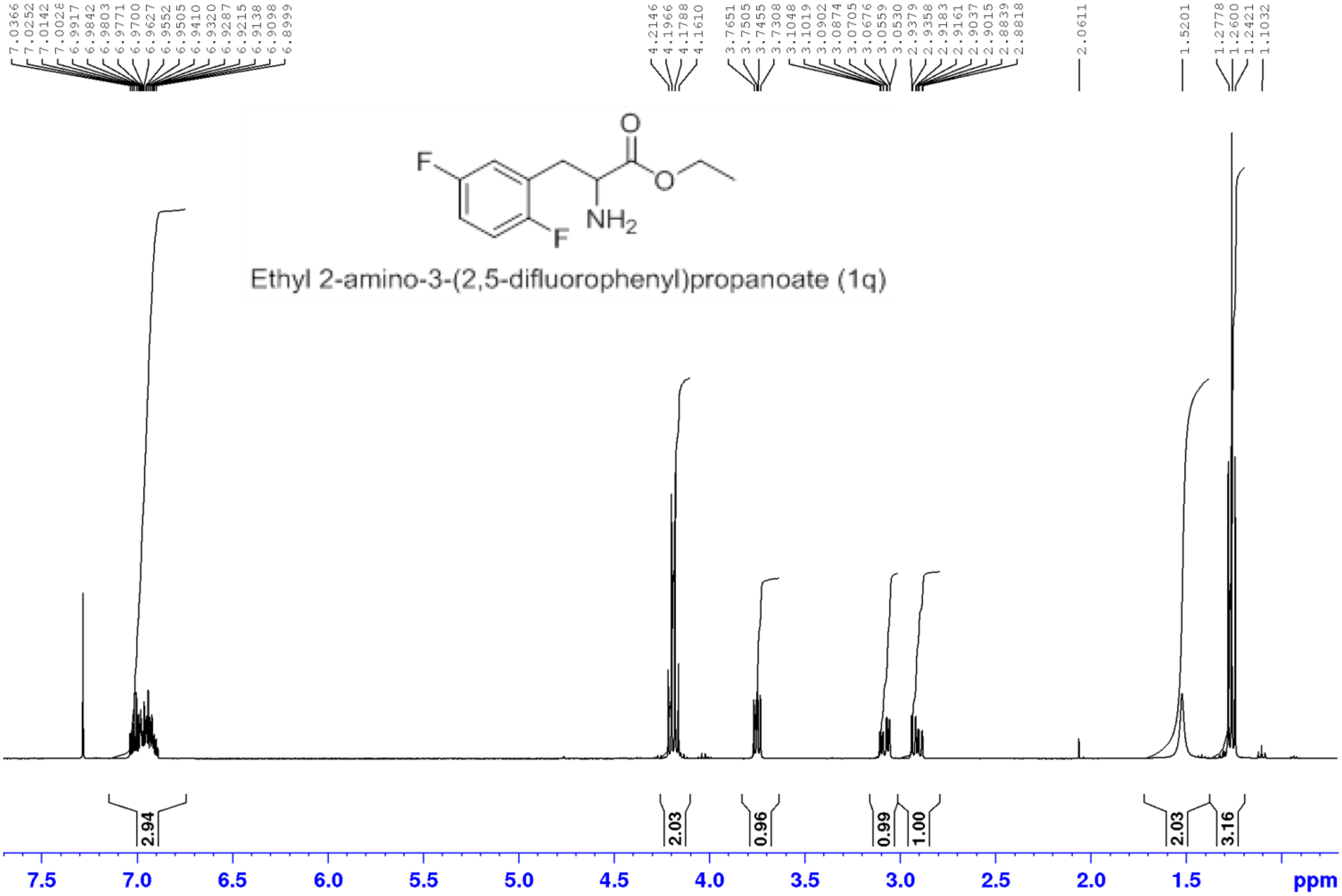
7.0366 7.0252 7.0142 7.0028 6.9917 6.9842 6.9803 6.9771 6.9700 6.9627 6.9552 6.9505 6.9410 6.9320 6.9287 6.9215 6.9138 6.9098 6.8999
4.2146 4.1966 4.1788 4.1610
3.7651 3.7505 3.7455 3.7308
3.1048 3.1019 3.0902 3.0874 3.0705 3.0676 3.0559 3.0530
2.9379 2.9358 2.9183 2.9161 2.9037 2.9015 2.8839 2.8818
2.0611
1.5201
1.2778 1.2600 1.2421 1.1032
F
F
O
O
NH2
Ethyl 2-amino-3-(2,5-difluorophenyl)propanoate (1q)
2.94 2.03 0.96 0.99 1.00 2.03 3.16
7.5 7.0 6.5 6.0 5.5 5.0 4.5 4.0 3.5 3.0 2.5 2.0 1.5 ppm

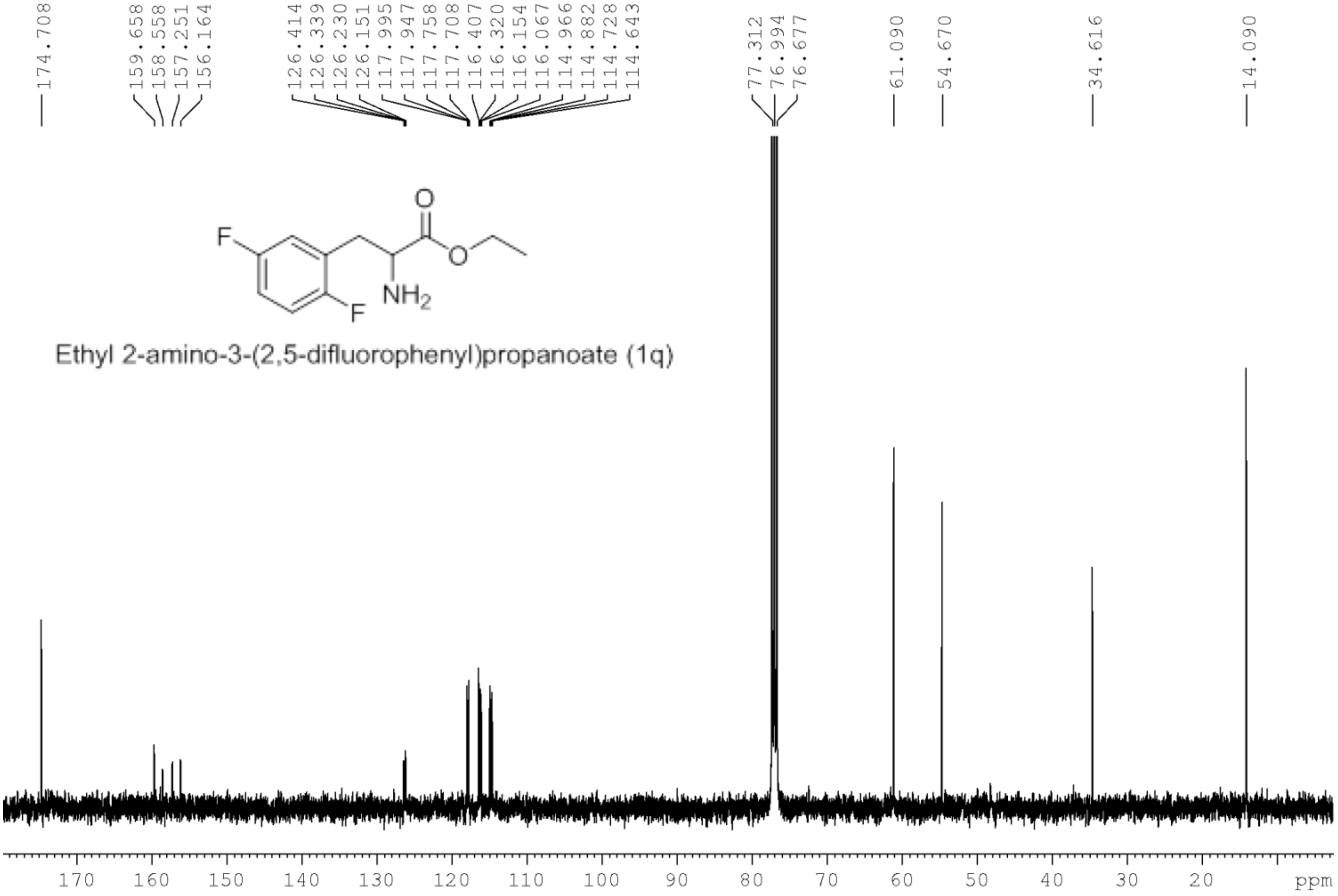
174.708
159.658 158.558 157.251 156.164
126.414 126.339 126.230 126.151 117.995 117.947 117.758 117.708 116.407 116.320 116.154 116.067 114.966 114.882 114.728 114.643
77.312 76.994 76.677
61.090
54.670
34.616
14.090
F
F
O
O
NH2
Ethyl 2-amino-3-(2,5-difluorophenyl)propanoate (1q)
170 160 150 140 130 120 110 100 90 80 70 60 50 40 30 20 ppm

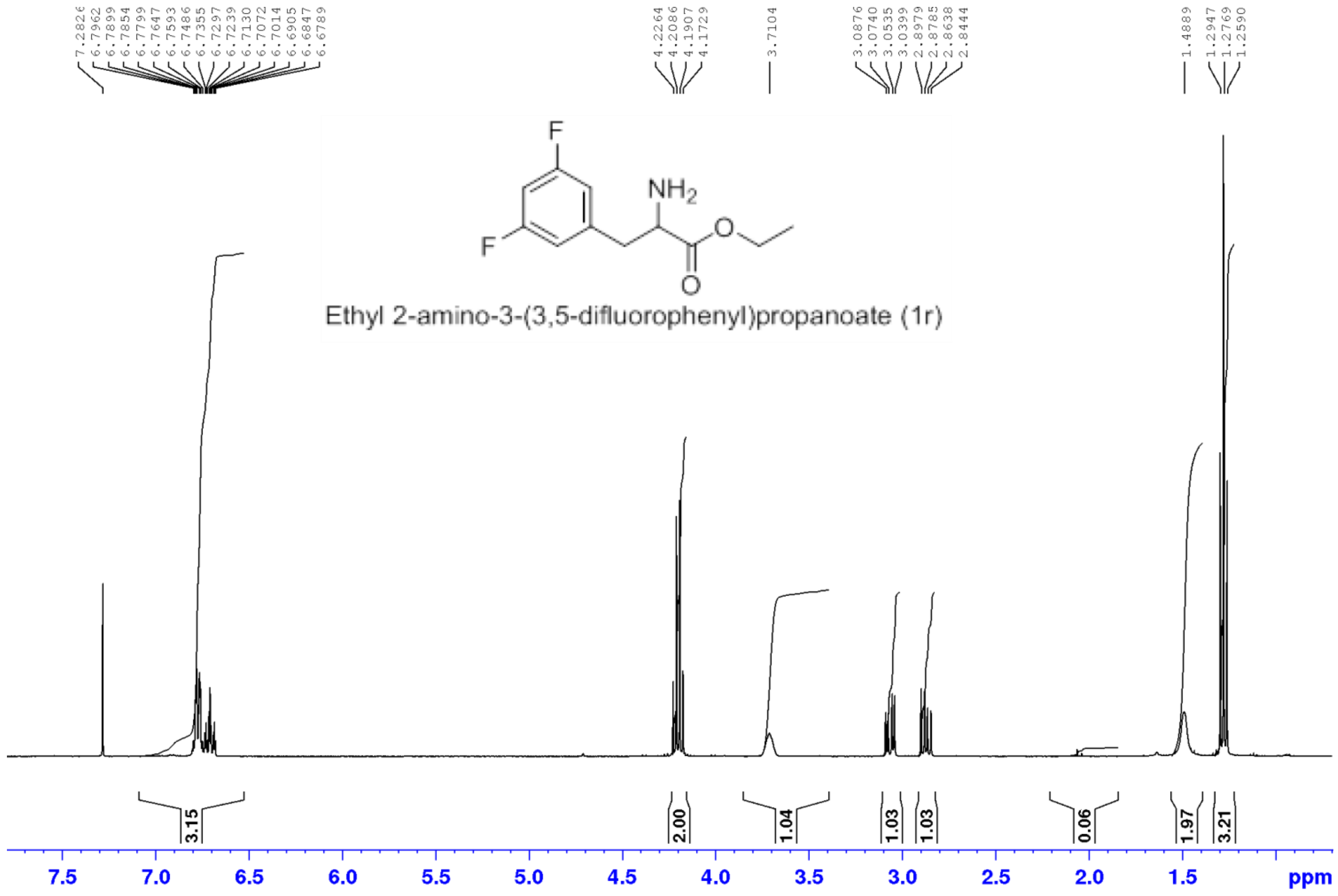
7.282[illegible]
6.7962
6.7899
6.7854
6.7799
6.7647
6.7593
6.7486
6.7355
6.7297
6.7239
6.7130
6.7072
6.7014
6.6905
6.6847
6.6789
4.2264
4.2086
4.1907
4.1729
3.7104
3.0876
3.0740
3.0535
3.0399
2.8979
2.8785
2.8638
2.8444
1.4889
1.2947
1.2769
1.2590
F
F
NH2
O
O
Ethyl 2-amino-3-(3,5-difluorophenyl)propanoate (1r)
3.15
2.00
1.04
1.03
1.03
0.06
1.97
3.21
7.5
7.0
6.5
6.0
5.5
5.0
4.5
4.0
3.5
3.0
2.5
2.0
1.5
ppm

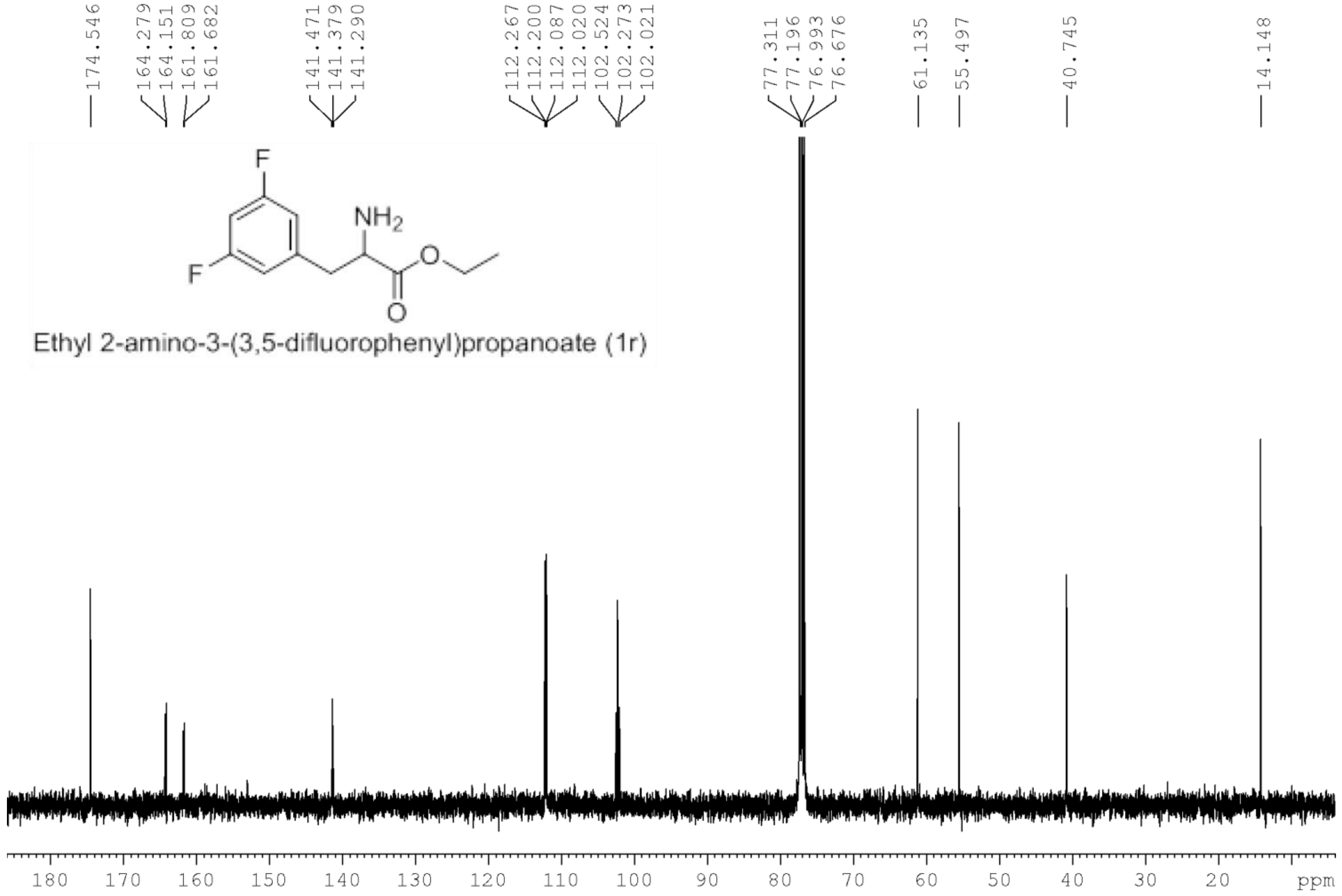
174.546
164.279
164.151
161.809
161.682
141.471
141.379
141.290
112.267
112.200
112.087
112.020
102.524
102.273
102.021
77.311
77.196
76.993
76.676
61.135
55.497
40.745
14.148
F
F
NH2
O
O
Ethyl 2-amino-3-(3,5-difluorophenyl)propanoate (1r)
180
170
160
150
140
130
120
110
100
90
80
70
60
50
40
30
20
ppm

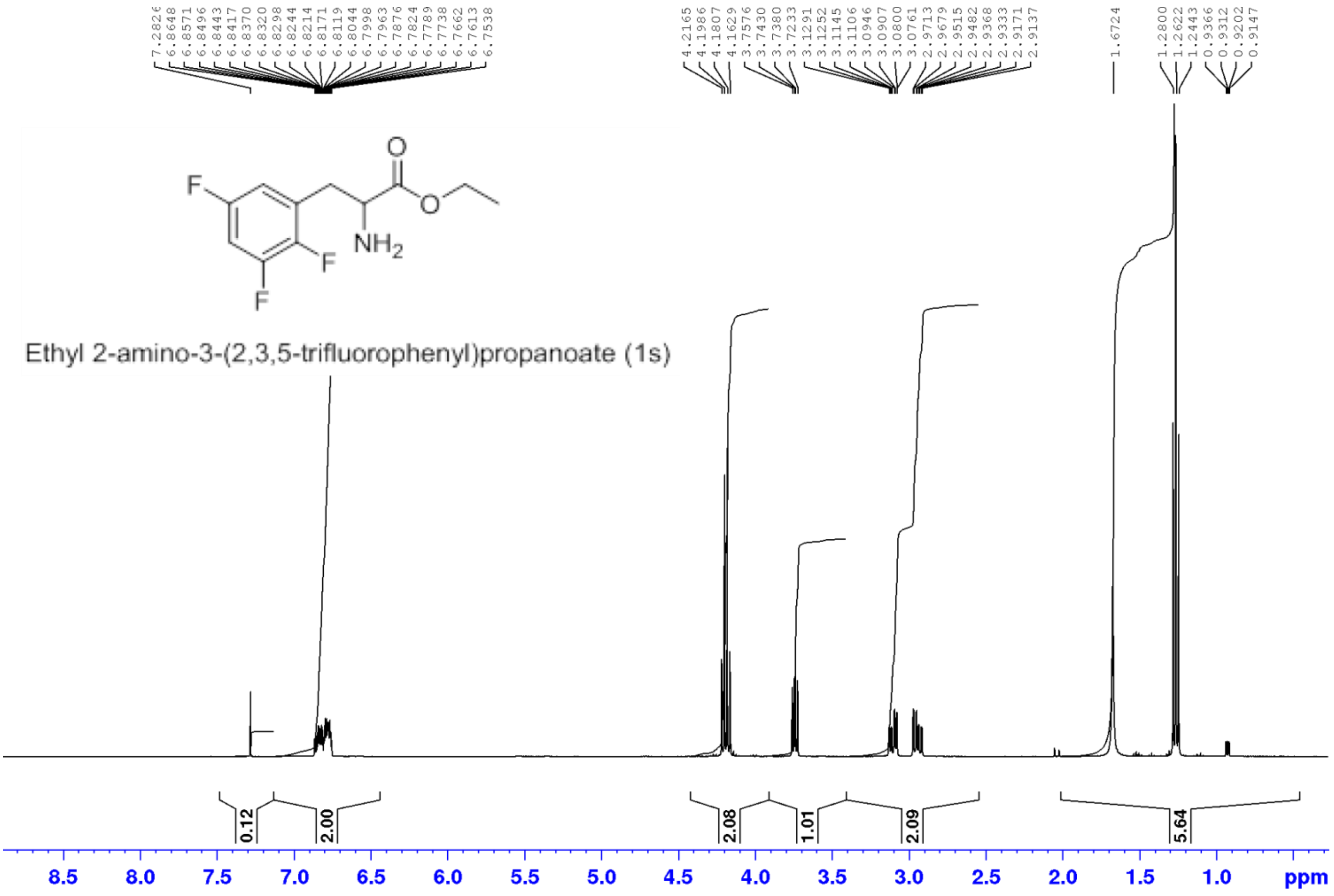
7.2826 6.8648 6.8571 6.8496 6.8443 6.8417 6.8370 6.8320 6.8298 6.8244 6.8214 6.8171 6.8119 6.8044 6.7998 6.7963 6.7876 6.7824 6.7789 6.7738 6.7662 6.7613 6.7538
4.2165 4.1986 4.1807 4.1629 3.7576 3.7430 3.7380 3.7233 3.1291 3.1252 3.1145 3.1106 3.0946 3.0907 3.0800 3.0761 2.9713 2.9679 2.9515 2.9482 2.9368 2.9333 2.9171 2.9137
1.6724
1.2800 1.2622 1.2443 0.9366 0.9312 0.9202 0.9147
F
O
O
NH2
F
F
Ethyl 2-amino-3-(2,3,5-trifluorophenyl)propanoate (1s)
0.12
2.00
2.08
1.01
2.09
5.64
8.5 8.0 7.5 7.0 6.5 6.0 5.5 5.0 4.5 4.0 3.5 3.0 2.5 2.0 1.5 1.0 ppm

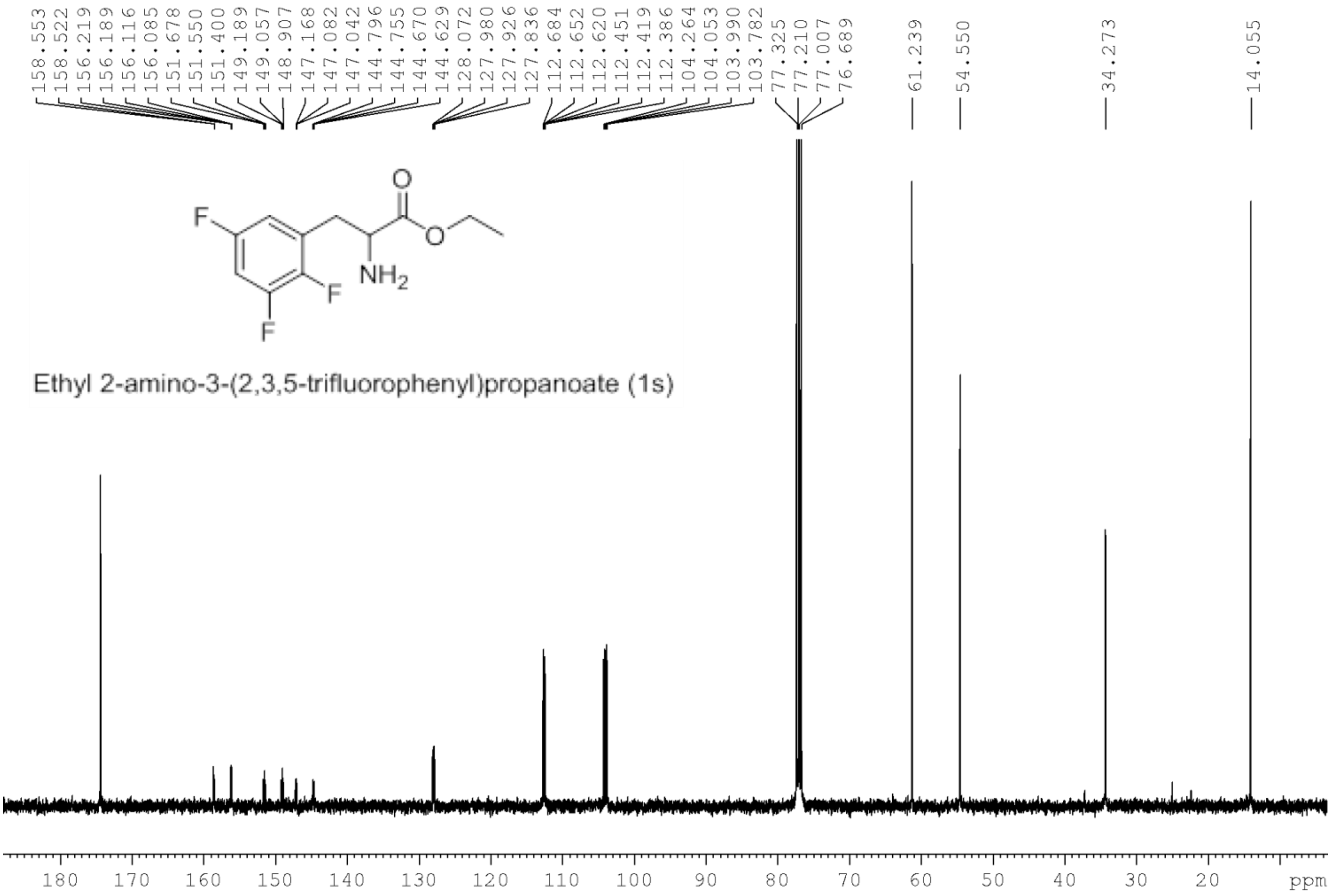
158.553 158.522 156.219 156.189 156.116 156.085 151.678 151.550 151.400 149.189 149.057 148.907 147.168 147.082 147.042 144.796 144.755 144.670 144.629 128.072 127.980 127.926 127.836 112.684 112.652 112.620 112.451 112.419 112.386 104.264 104.053 103.990 103.782 77.325 77.210 77.007 76.689
61.239
54.550
34.273
14.055
F
O
O
NH2
F
F
Ethyl 2-amino-3-(2,3,5-trifluorophenyl)propanoate (1s)
180 170 160 150 140 130 120 110 100 90 80 70 60 50 40 30 20 ppm

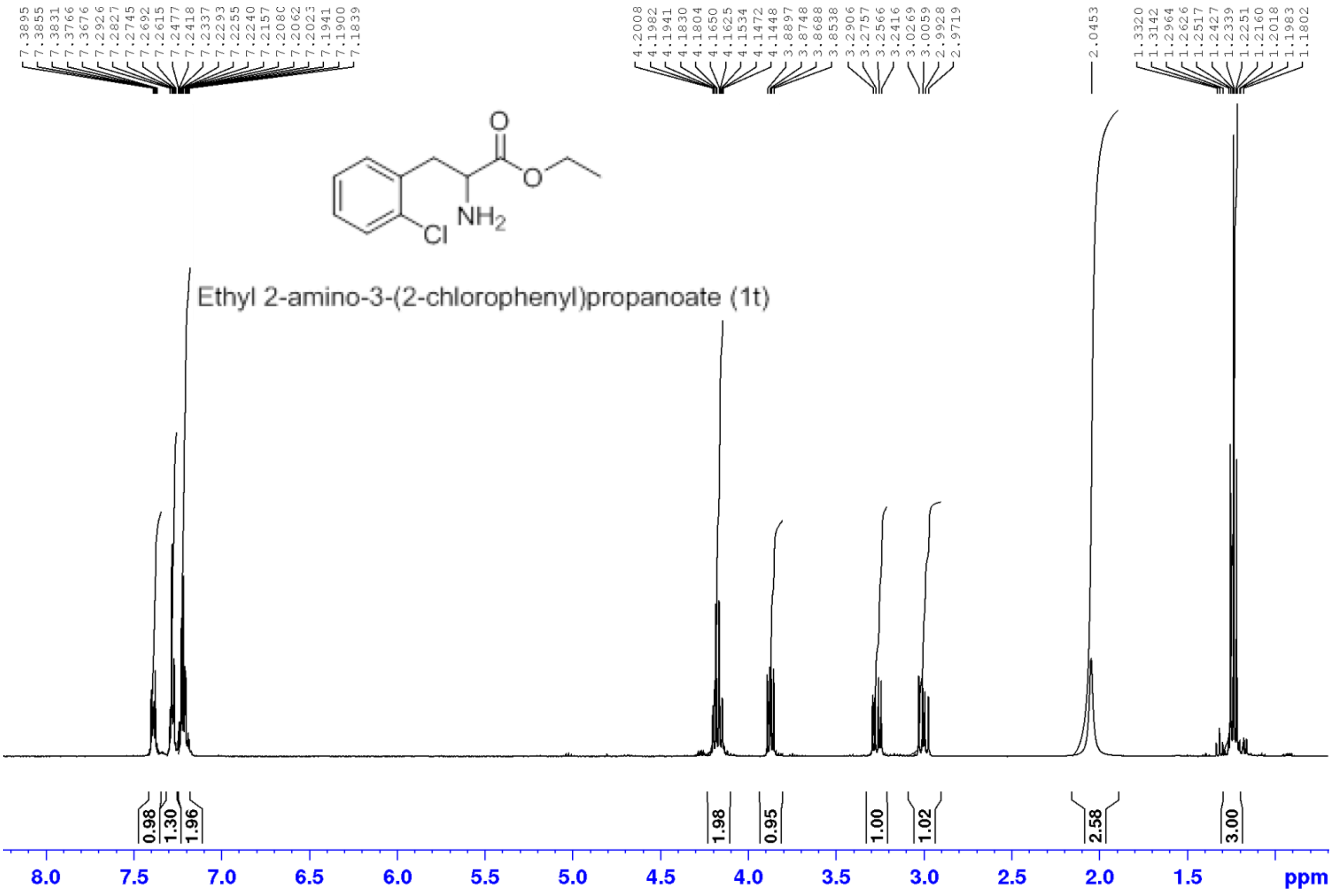

7.3895 7.3855 7.3831 7.3766 7.3676 7.2926 7.2827 7.2745 7.2692 7.2615 7.2477 7.2418 7.2337 7.2293 7.2255 7.2240 7.2157 7.208C 7.2062 7.2023 7.1941 7.1900 7.1839
4.2008 4.1982 4.1941 4.1830 4.1804 4.1650 4.1625 4.1534 4.1472 4.1448 3.8897 3.8748 3.8688 3.8538 3.2906 3.2757 3.2566 3.2416 3.0269 3.0059 2.9928 2.9719
2.0453
1.3320 1.3142 1.2964 1.2626 1.2517 1.2427 1.2339 1.2251 1.2160 1.2018 1.1983 1.1802
O
O
NH2
Cl
Ethyl 2-amino-3-(2-chlorophenyl)propanoate (1t)
0.98 1.30 1.96 1.98 0.95 1.00 1.02 2.58 3.00
8.0 7.5 7.0 6.5 6.0 5.5 5.0 4.5 4.0 3.5 3.0 2.5 2.0 1.5 ppm

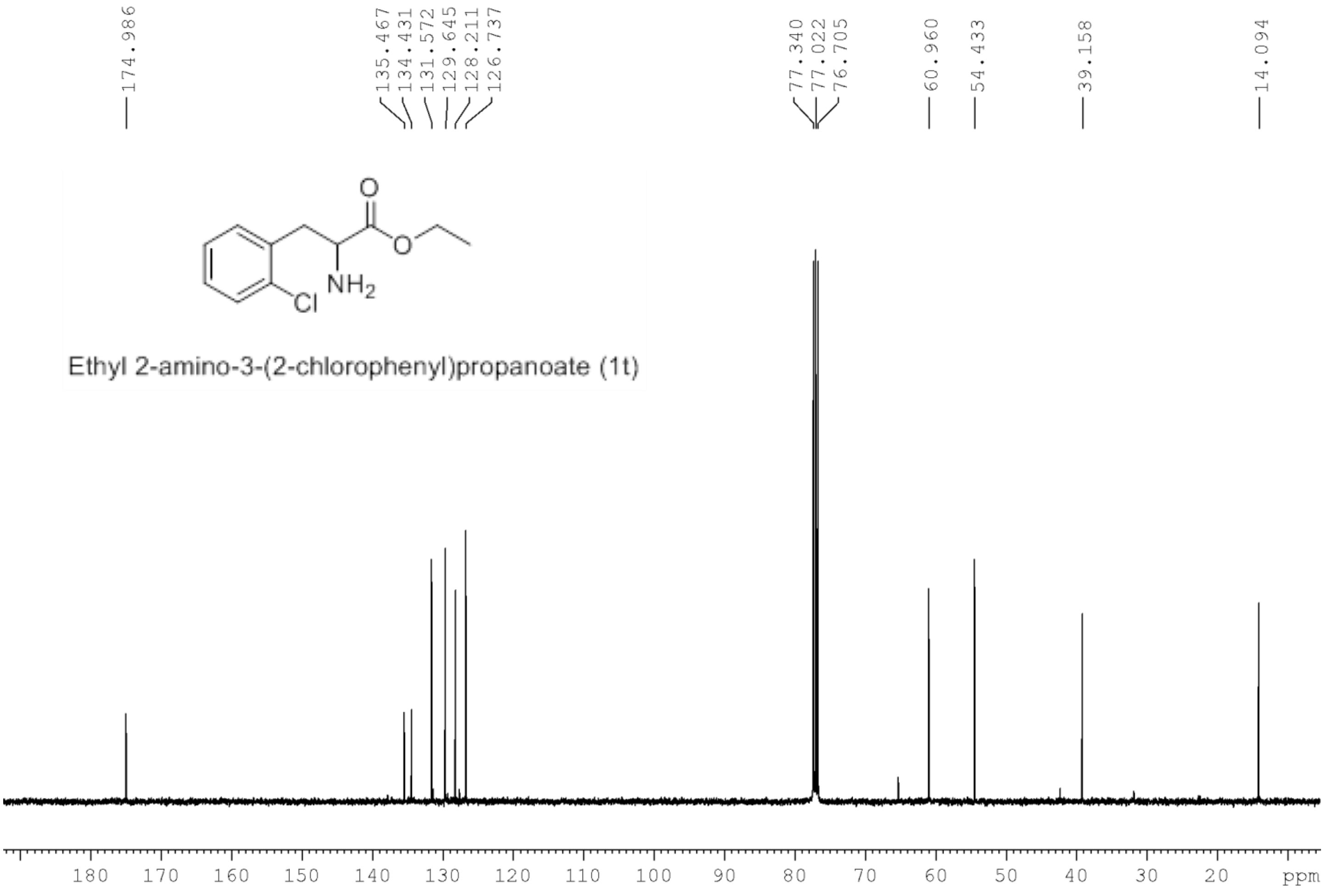

174.986
135.467 134.431 131.572 129.645 128.211 126.737
77.340 77.022 76.705
60.960
54.433
39.158
14.094
O
O
NH2
Cl
Ethyl 2-amino-3-(2-chlorophenyl)propanoate (1t)
180 170 160 150 140 130 120 110 100 90 80 70 60 50 40 30 20 ppm

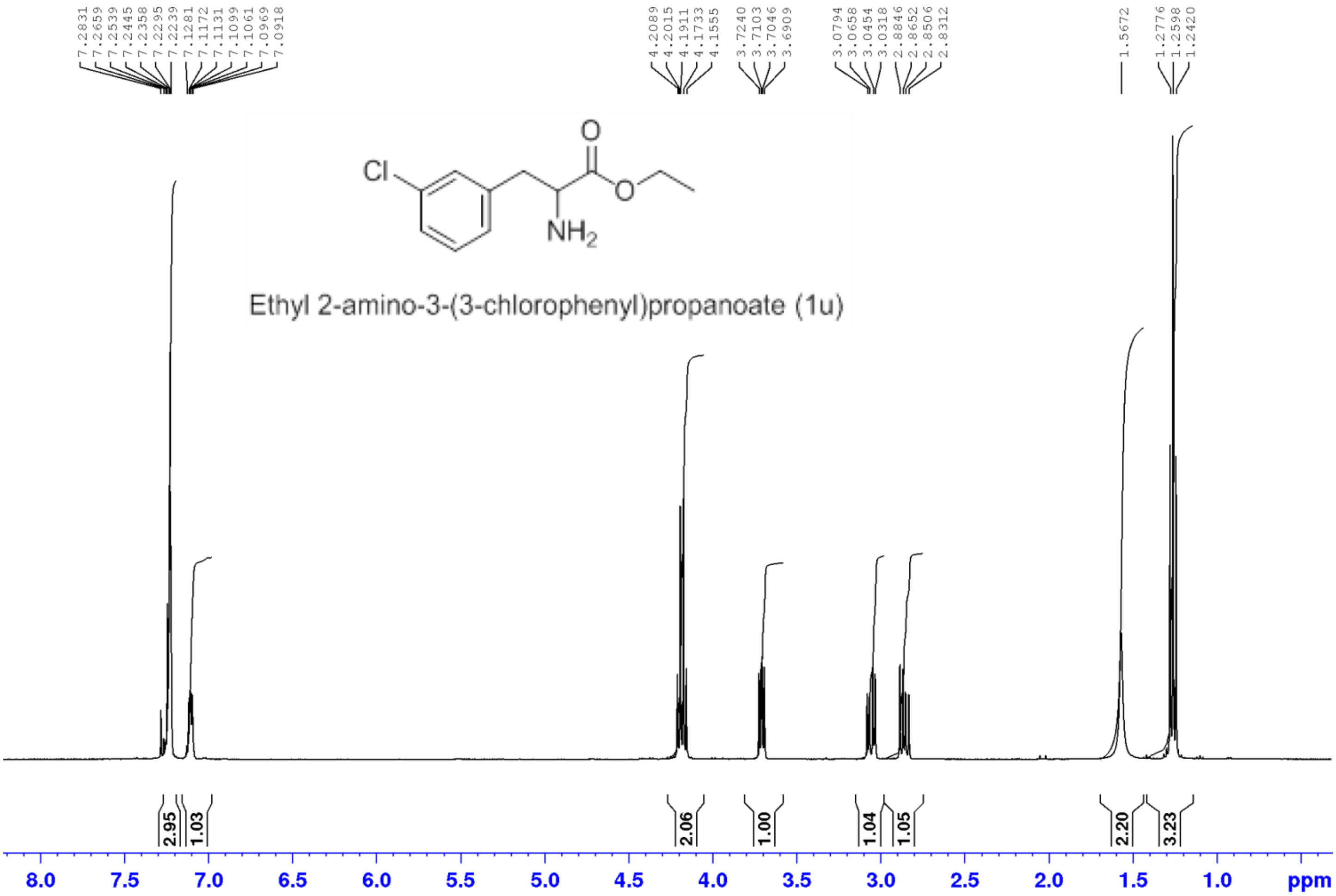
7.2831 7.2659 7.2539 7.2445 7.2358 7.2295 7.2239 7.1281 7.1172 7.1131 7.1099 7.1061 7.0969 7.0918
4.2089 4.2015 4.1911 4.1733 4.1555 3.7240 3.7103 3.7046 3.6909
3.0794 3.0658 3.0454 3.0318 2.8846 2.8652 2.8506 2.8312
1.5672 1.2776 1.2598 1.2420
Cl
O
O
NH2
Ethyl 2-amino-3-(3-chlorophenyl)propanoate (1u)
2.95 1.03 2.06 1.00 1.04 1.05 2.20 3.23
8.0 7.5 7.0 6.5 6.0 5.5 5.0 4.5 4.0 3.5 3.0 2.5 2.0 1.5 1.0 ppm

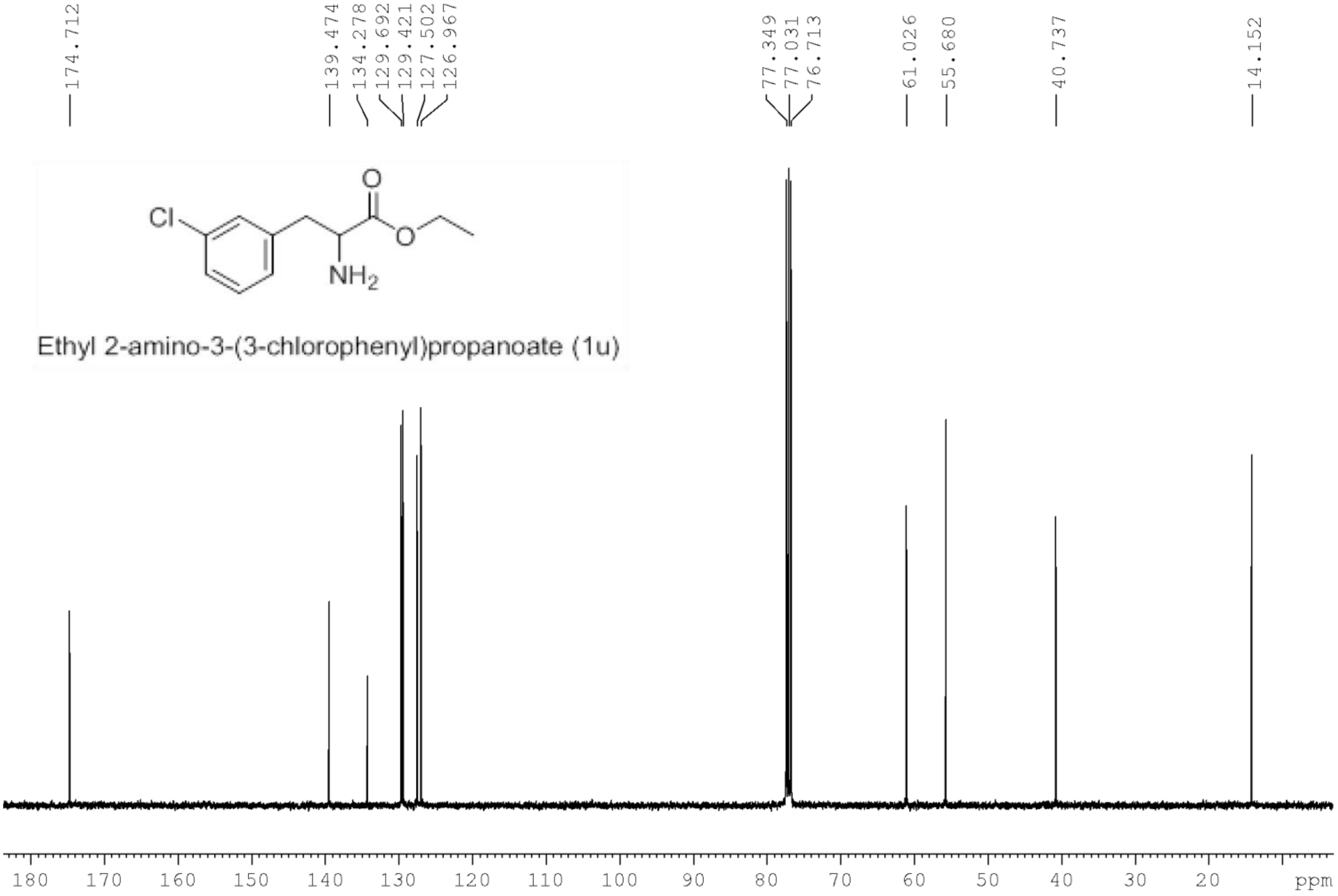
174.712
139.474 134.278 129.692 129.421 127.502 126.967
77.349 77.031 76.713
61.026 55.680 40.737 14.152
Cl
O
O
NH2
Ethyl 2-amino-3-(3-chlorophenyl)propanoate (1u)
180 170 160 150 140 130 120 110 100 90 80 70 60 50 40 30 20 ppm

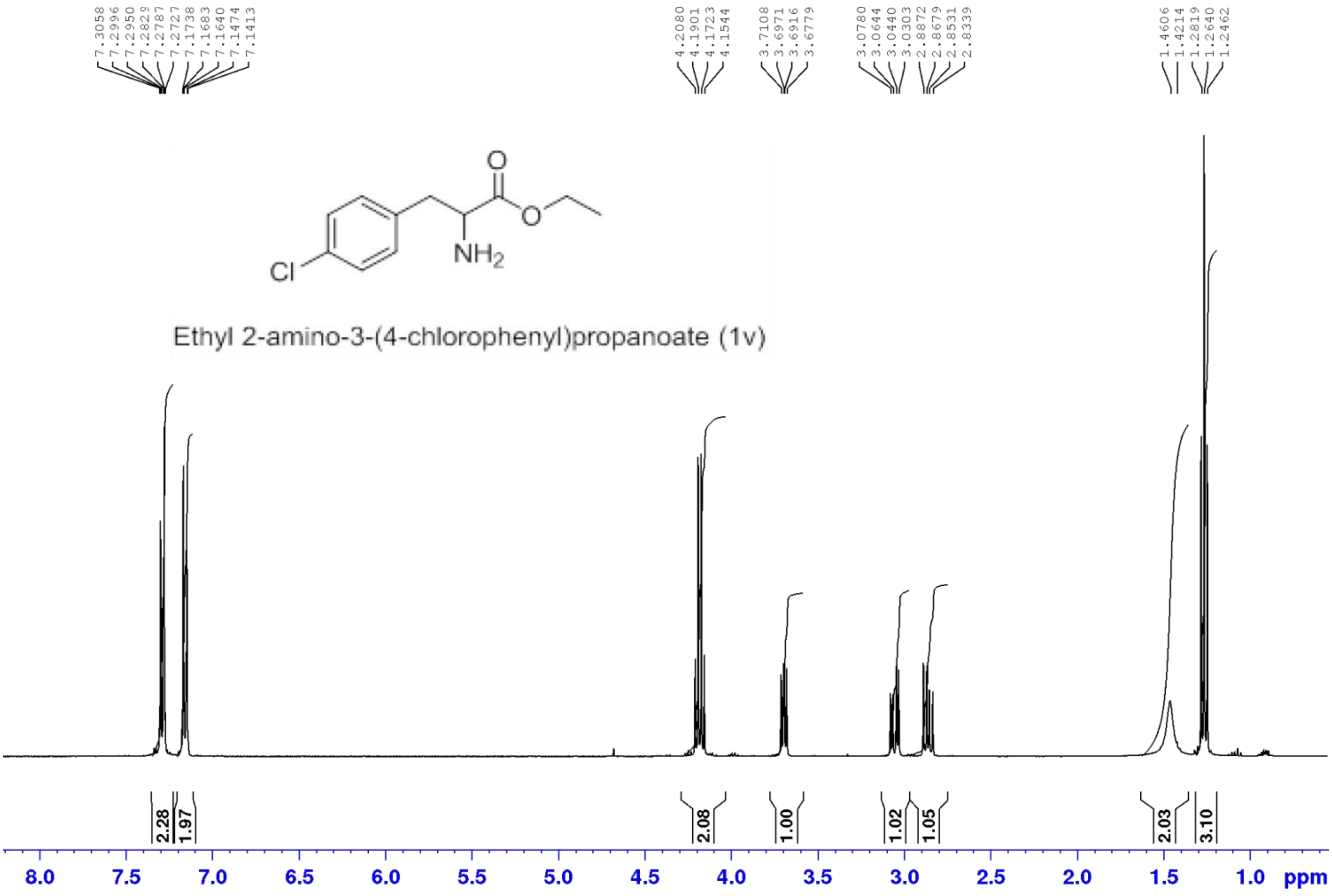
7.3058
7.2996
7.2950
7.2829
7.2787
7.2727
7.1738
7.1683
7.1640
7.1474
7.1413
4.2080
4.1901
4.1723
4.1544
3.7108
3.6971
3.6916
3.6779
3.0780
3.0644
3.0440
3.0303
2.8872
2.8679
2.8531
2.8339
1.4606
1.4214
1.2819
1.2640
1.2462
Cl
NH_2
O
Ethyl 2-amino-3-(4-chlorophenyl)propanoate (1v)
2.28
1.97
2.08
1.00
1.02
1.05
2.03
3.10
8.0 7.5 7.0 6.5 6.0 5.5 5.0 4.5 4.0 3.5 3.0 2.5 2.0 1.5 1.0 ppm

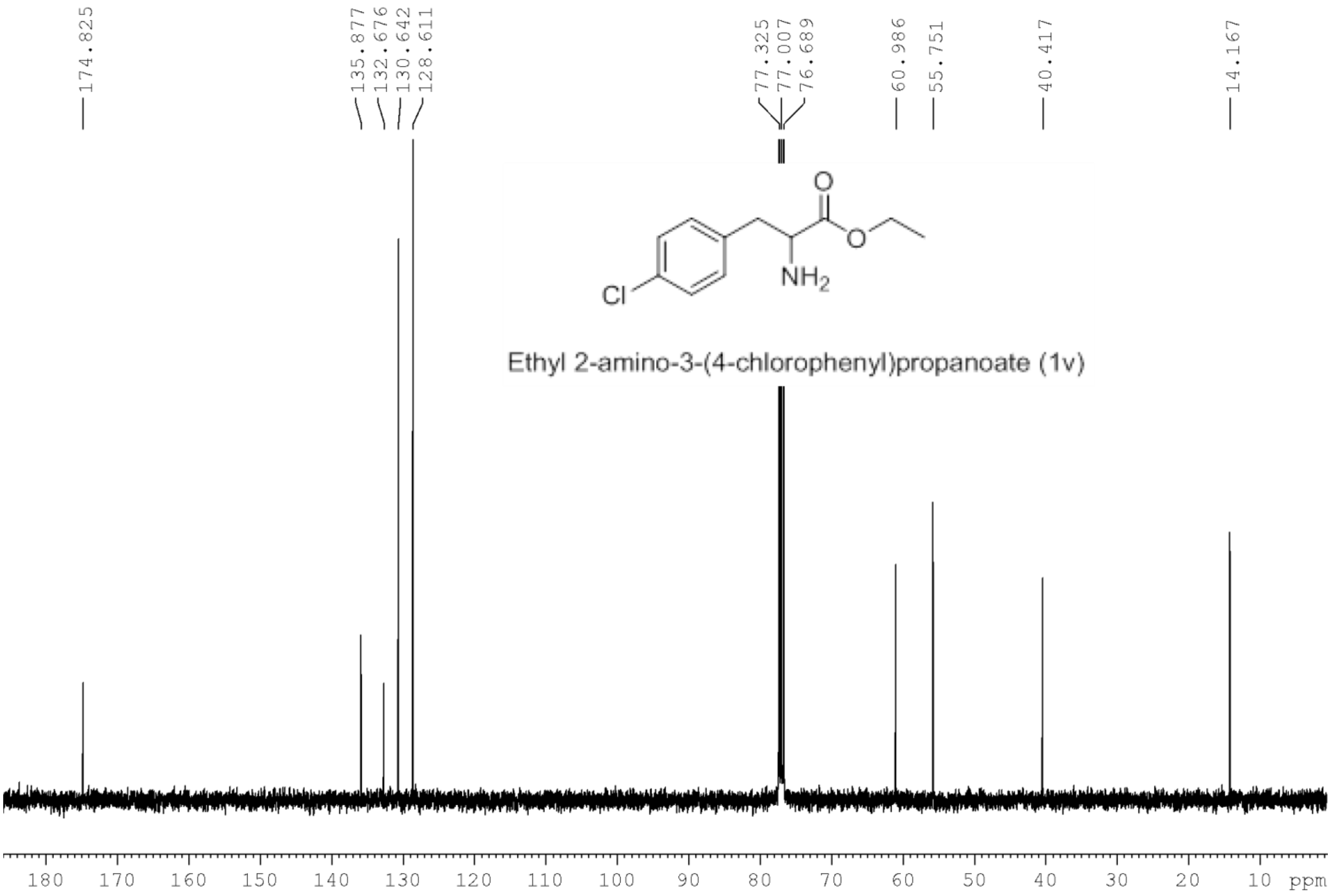
174.825
135.877
132.676
130.642
128.611
77.325
77.007
76.689
60.986
55.751
40.417
14.167
Cl
NH_2
O
Ethyl 2-amino-3-(4-chlorophenyl)propanoate (1v)
180 170 160 150 140 130 120 110 100 90 80 70 60 50 40 30 20 10 ppm

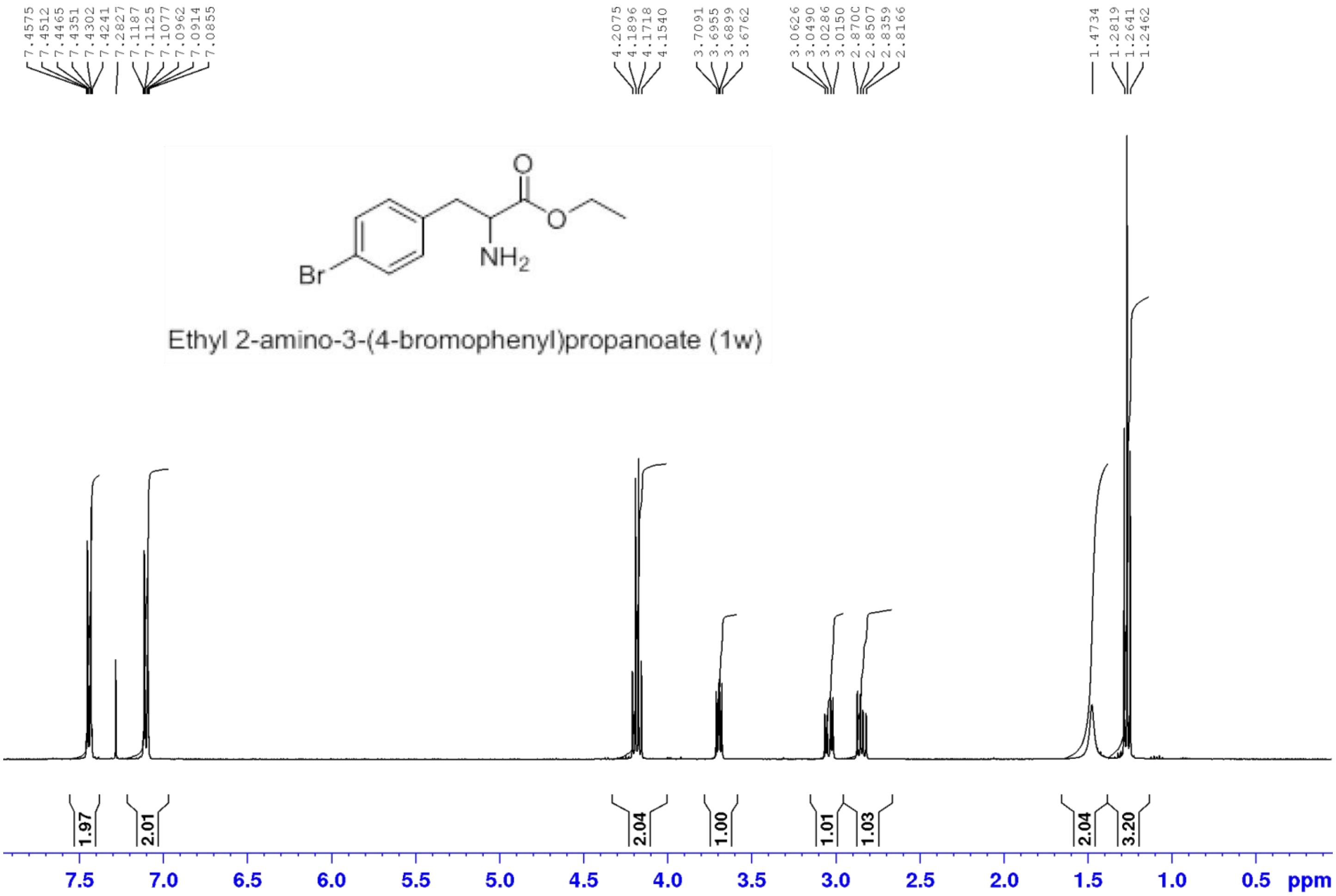
7.4575
7.4512
7.4465
7.4351
7.4302
7.4241
7.2827
7.1187
7.1125
7.1077
7.0962
7.0914
7.0855
4.2075
4.1896
4.1718
4.1540
3.7091
3.6955
3.6899
3.6762
3.0626
3.0490
3.0286
3.0150
2.8700
2.8507
2.8359
2.8166
1.4734
1.2819
1.2641
1.2462
Br
NH2
O
Ethyl 2-amino-3-(4-bromophenyl)propanoate (1w)
1.97
2.01
2.04
1.00
1.01
1.03
2.04
3.20
7.5
7.0
6.5
6.0
5.5
5.0
4.5
4.0
3.5
3.0
2.5
2.0
1.5
1.0
0.5
ppm

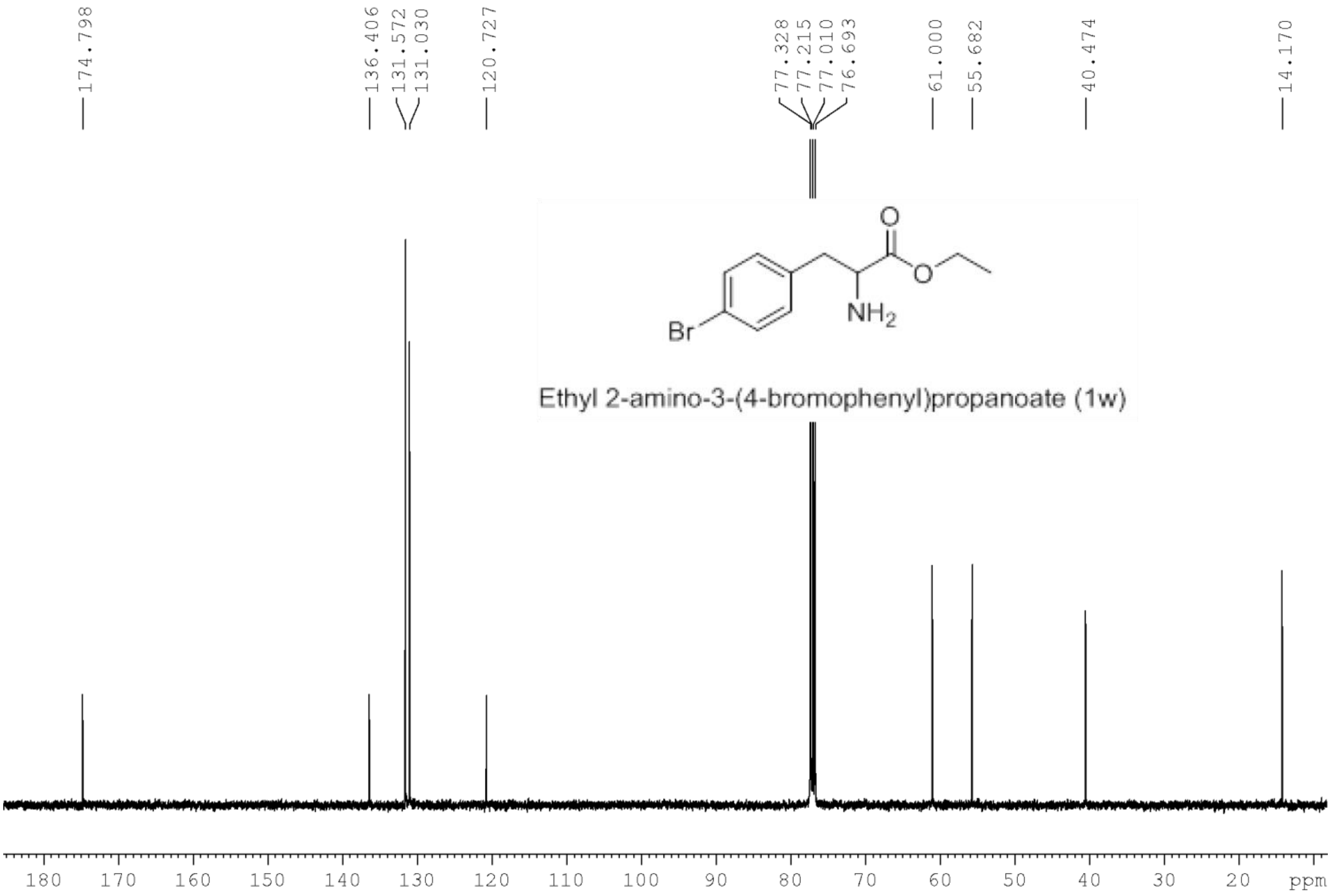
174.798
136.406
131.572
131.030
120.727
77.328
77.215
77.010
76.693
61.000
55.682
40.474
14.170
Br
NH2
O
Ethyl 2-amino-3-(4-bromophenyl)propanoate (1w)
180
170
160
150
140
130
120
110
100
90
80
70
60
50
40
30
20
ppm

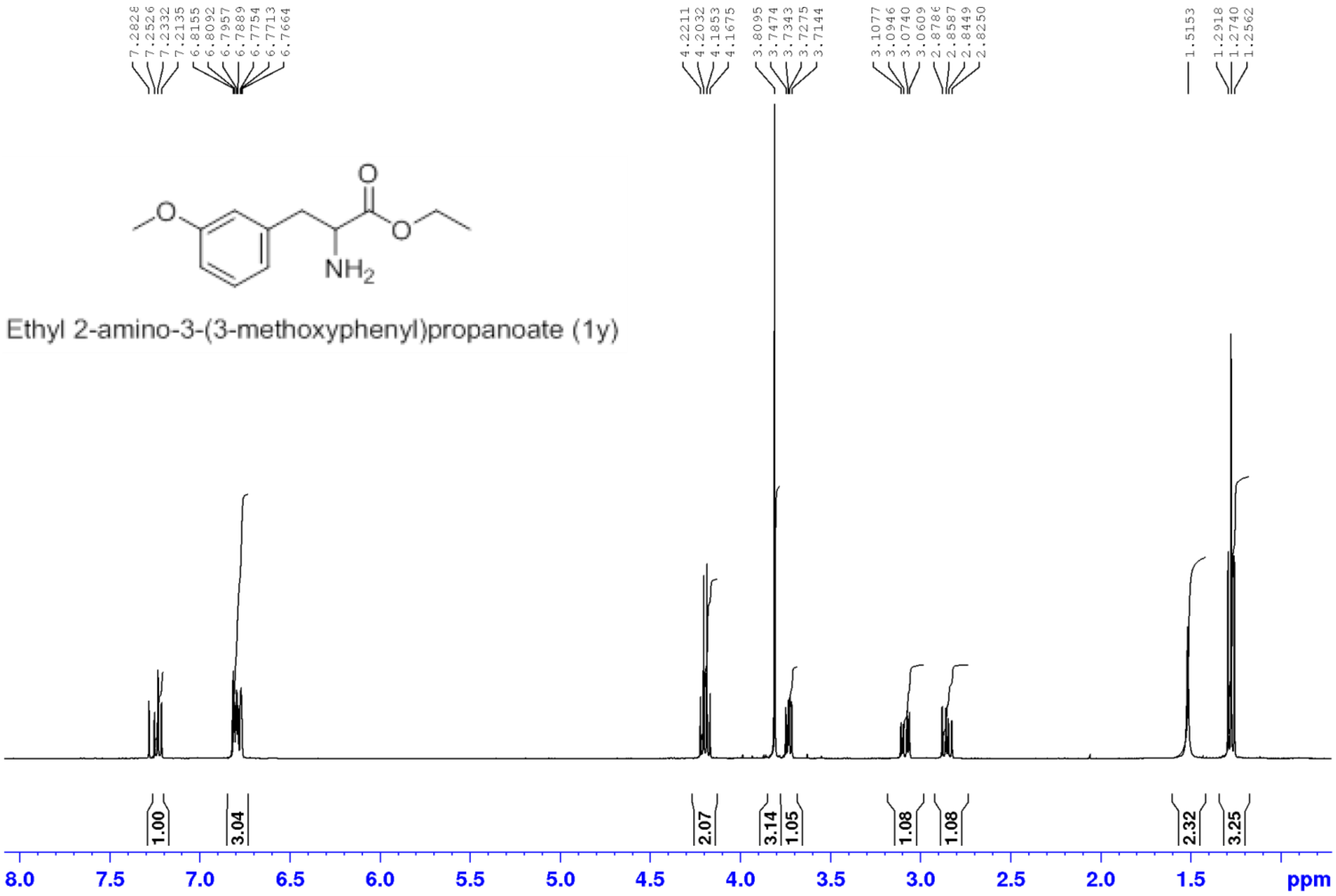
7.2828
7.2526
7.2332
7.2135
6.8155
6.8092
6.7957
6.7889
6.7754
6.7713
6.7664
4.2211
4.2032
4.1853
4.1675
3.8095
3.7474
3.7343
3.7275
3.7144
3.1077
3.0946
3.0740
3.0609
2.8786
2.8587
2.8449
2.8250
1.5153
1.2918
1.2740
1.2562
O
O
O
NH2
Ethyl 2-amino-3-(3-methoxyphenyl)propanoate (1y)
1.00
3.04
2.07
3.14
1.05
1.08
1.08
2.32
3.25
8.0
7.5
7.0
6.5
6.0
5.5
5.0
4.5
4.0
3.5
3.0
2.5
2.0
1.5
ppm

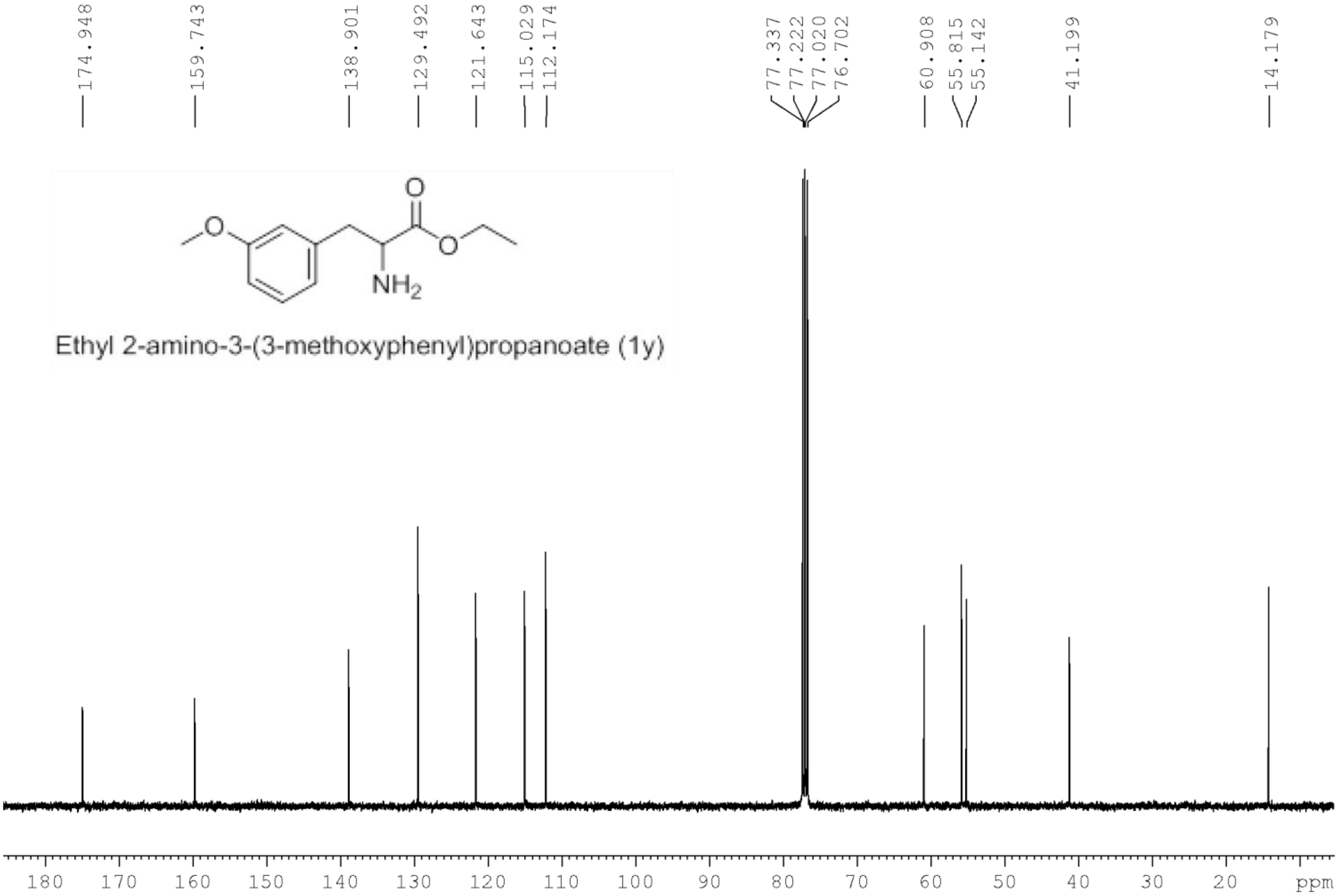
174.948
159.743
138.901
129.492
121.643
115.029
112.174
77.337
77.222
77.020
76.702
60.908
55.815
55.142
41.199
14.179
O
O
O
NH2
Ethyl 2-amino-3-(3-methoxyphenyl)propanoate (1y)
180
170
160
150
140
130
120
110
100
90
80
70
60
50
40
30
20
ppm

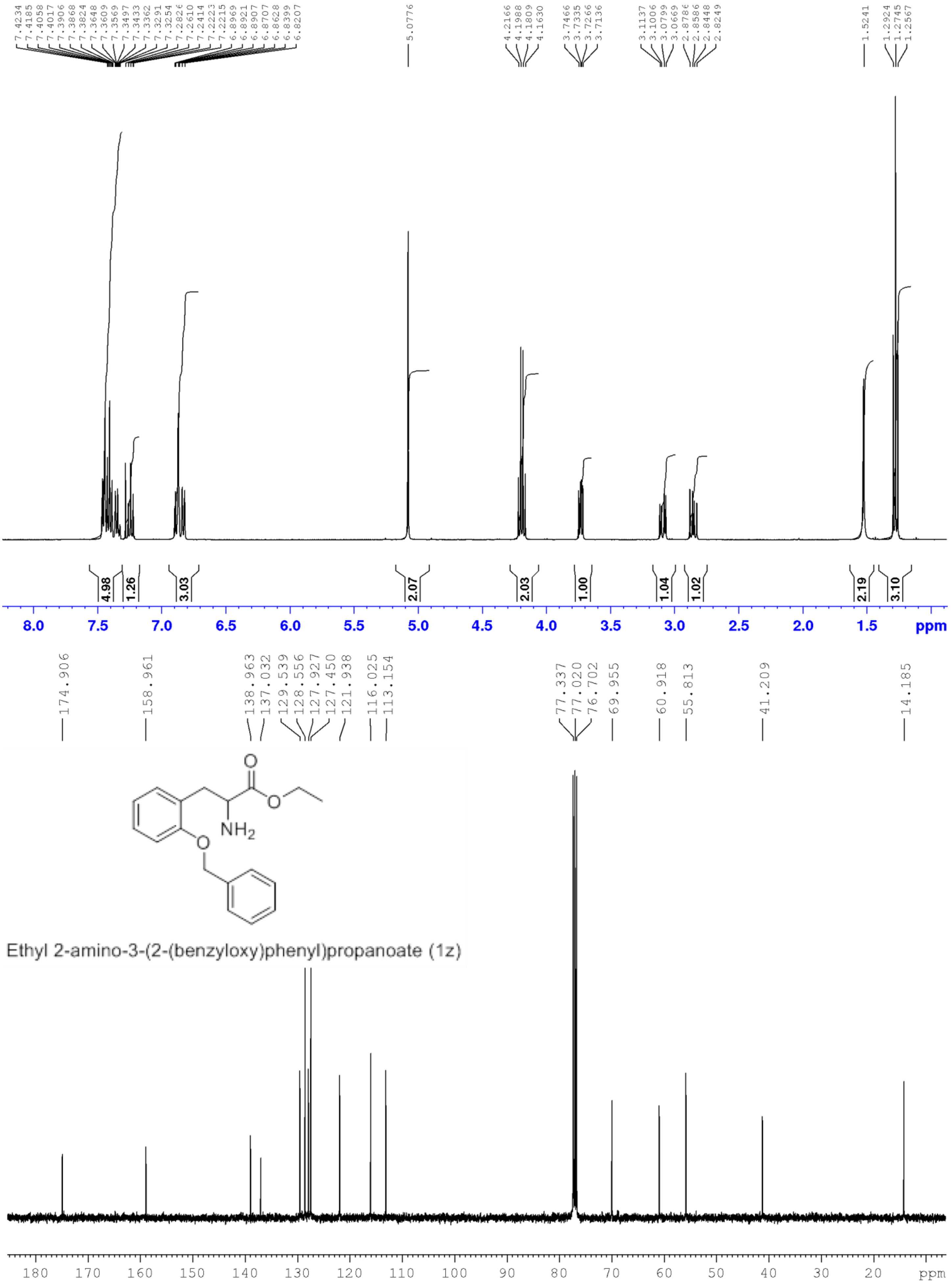

Ethyl 2-amino-3-(2-(benzyloxy)phenyl)propanoate (1z)

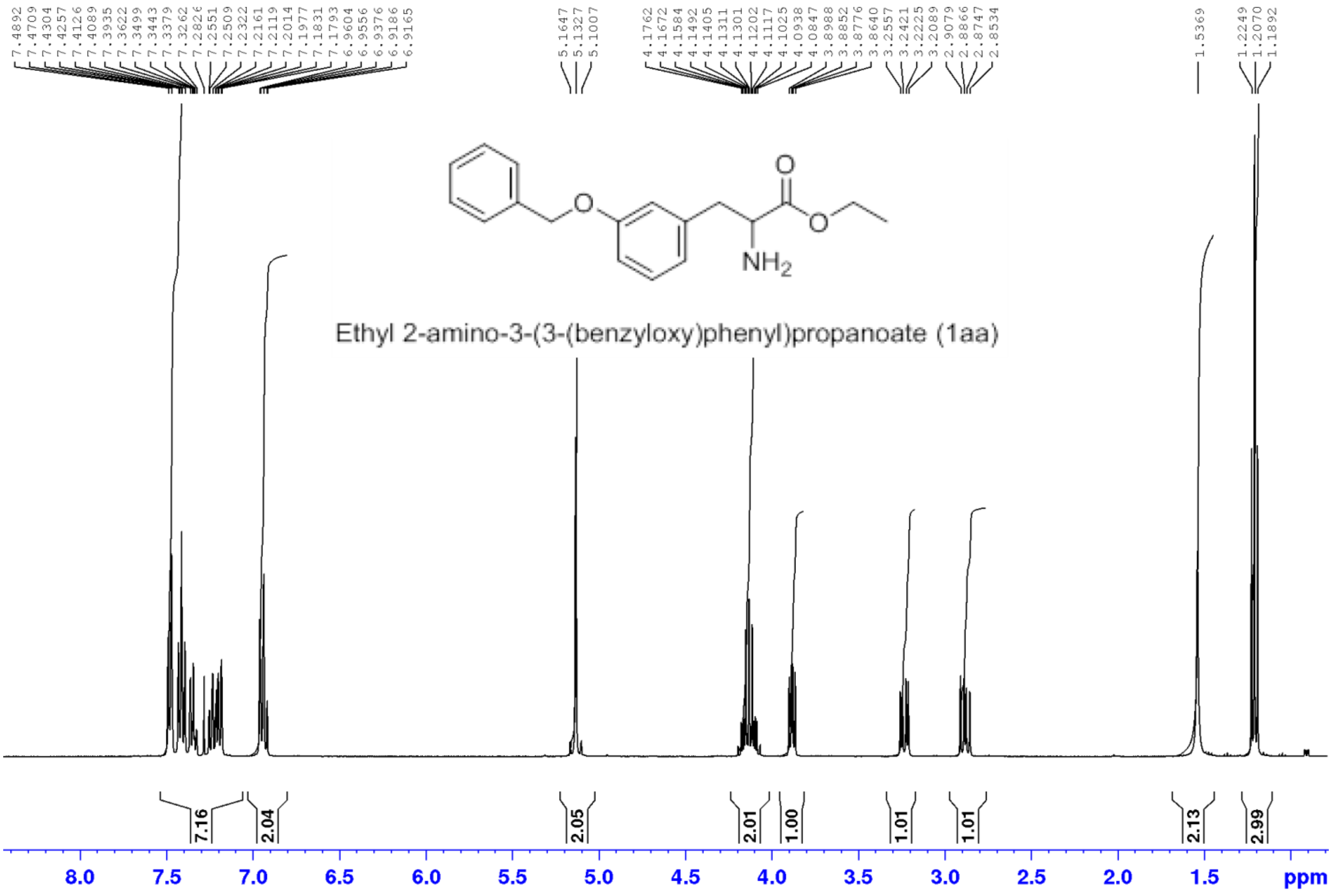
Ethyl 2-amino-3-(3-(benzyloxy)phenyl)propanoate (1aa)
ppm

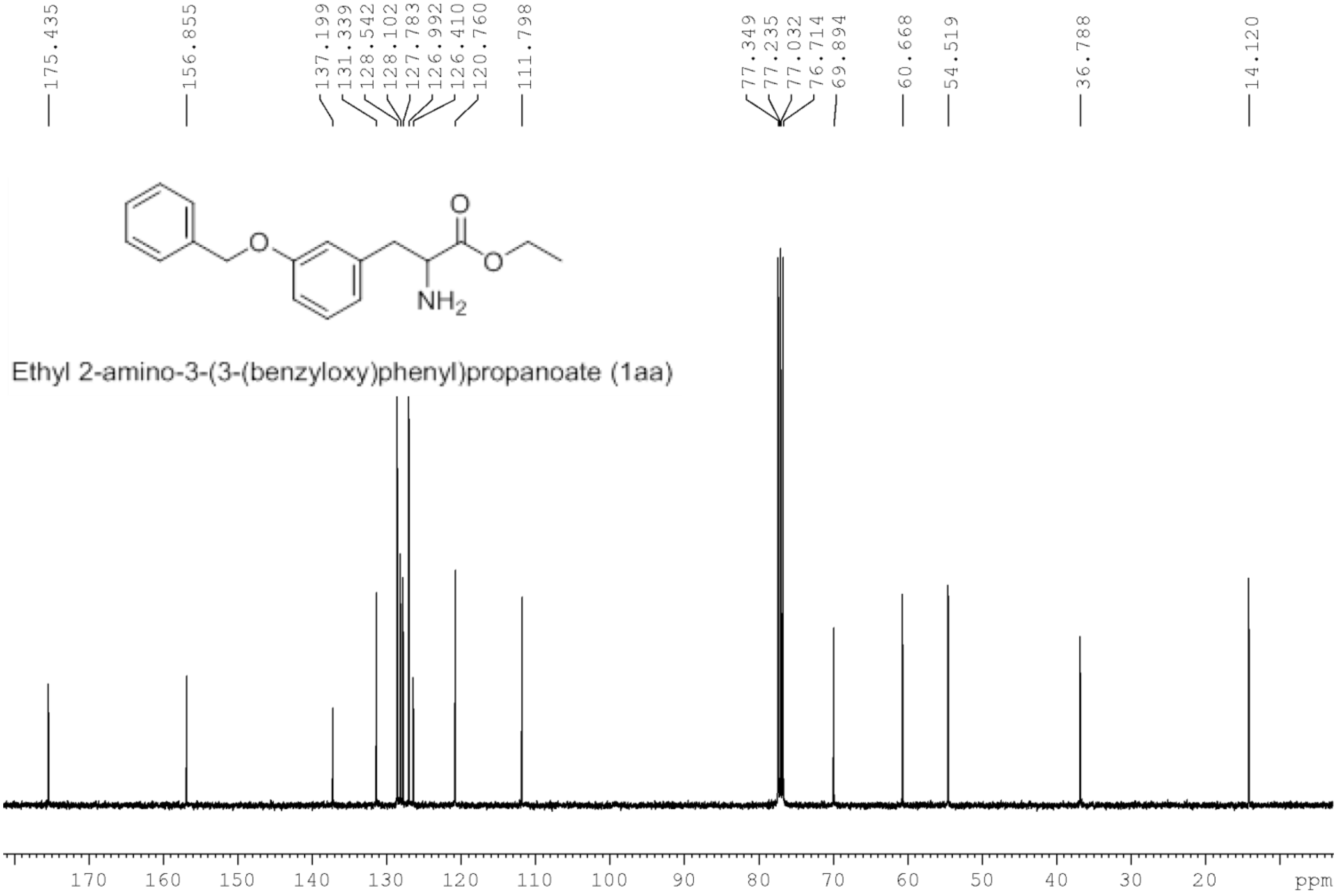
Ethyl 2-amino-3-(3-(benzyloxy)phenyl)propanoate (1aa)
ppm

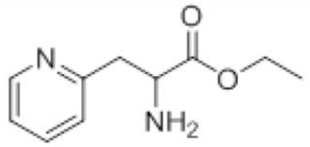

Ethyl 2-amino-3-(pyridin-2-yl)propanoate (1ac)

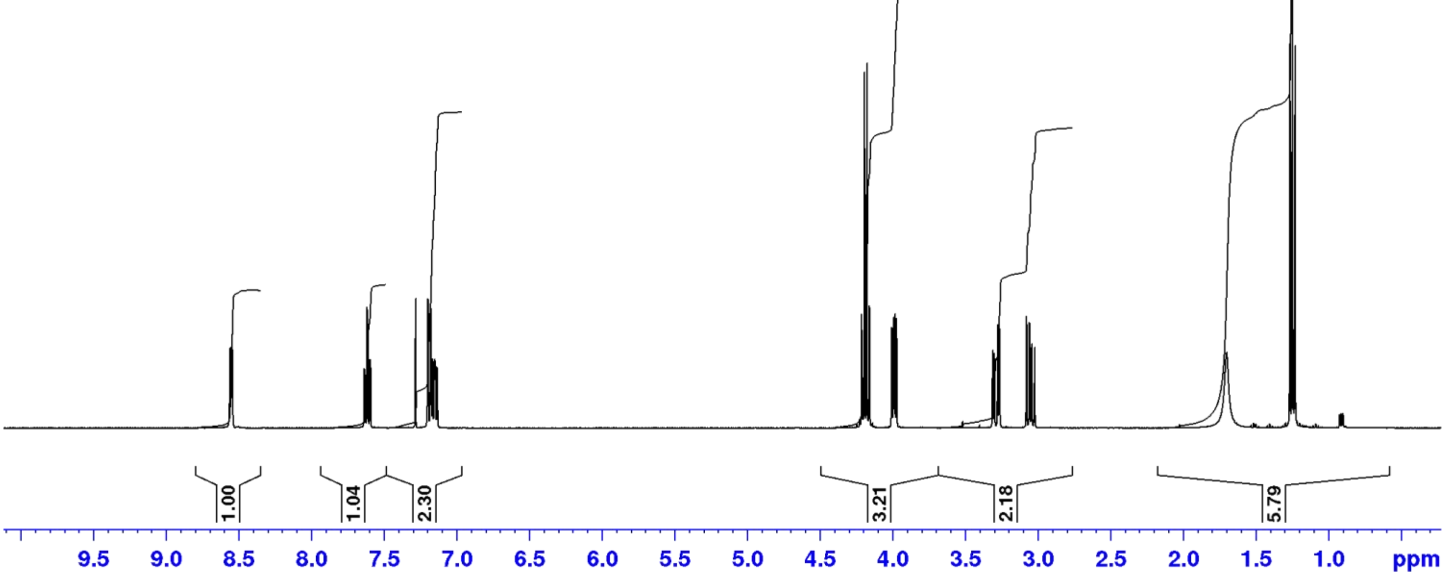

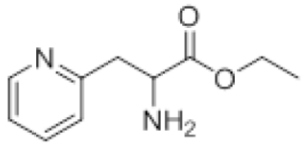

Ethyl 2-amino-3-(pyridin-2-yl)propanoate (1ac)

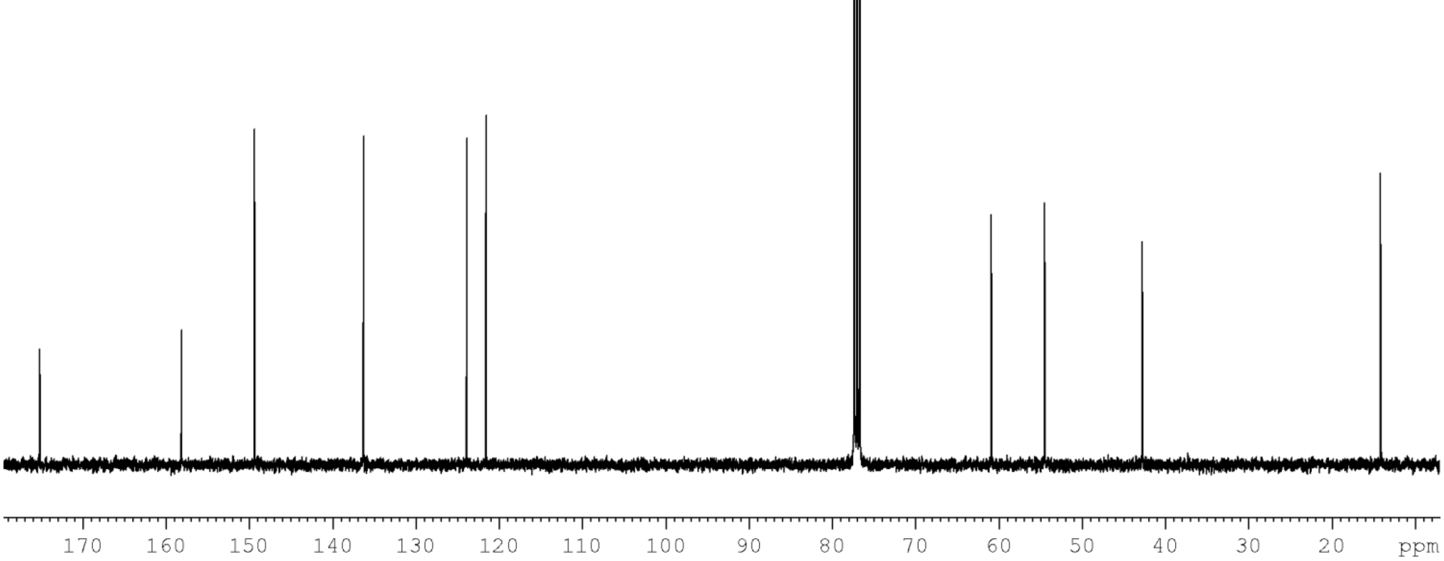

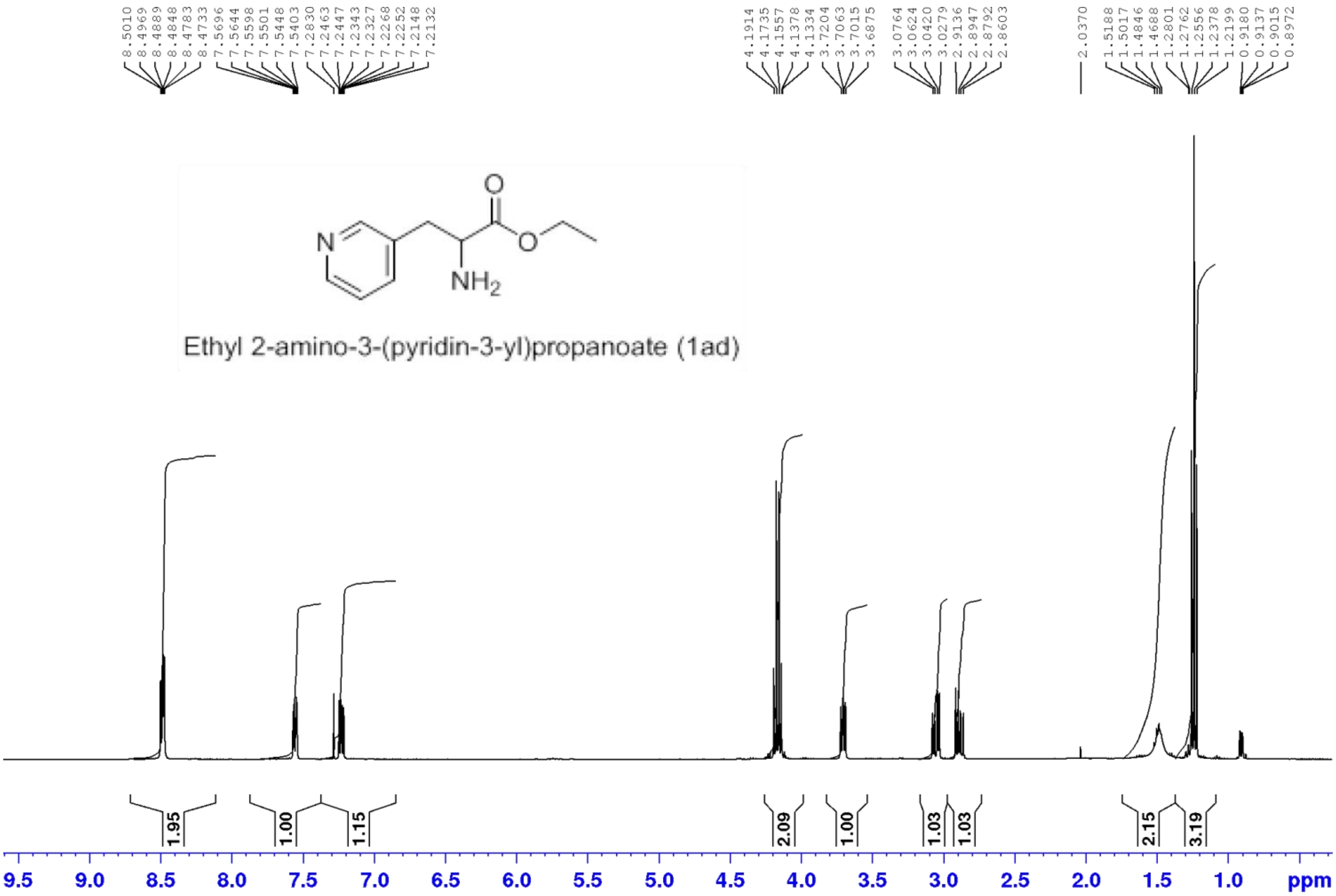

8.5010
8.4969
8.4889
8.4848
8.4783
7.5696
7.5644
7.5598
7.5501
7.5448
7.5403
7.2830
7.2463
7.2447
7.2343
7.2327
7.2268
7.2252
7.2148
7.2132
4.1914
4.1735
4.1557
4.1378
4.1334
3.7204
3.7063
3.7015
3.6875
3.0764
3.0624
3.0420
3.0279
2.9136
2.8947
2.8792
2.8603
2.0370
1.5188
1.5017
1.4846
1.4688
1.2801
1.2762
1.2556
1.2378
1.2199
0.9180
0.9137
0.9015
0.8972
NH2
Ethyl 2-amino-3-(pyridin-3-yl)propanoate (1ad)
1.95
1.00
1.15
2.09
1.00
1.03
1.03
2.15
3.19
9.5 9.0 8.5 8.0 7.5 7.0 6.5 6.0 5.5 5.0 4.5 4.0 3.5 3.0 2.5 2.0 1.5 1.0 ppm

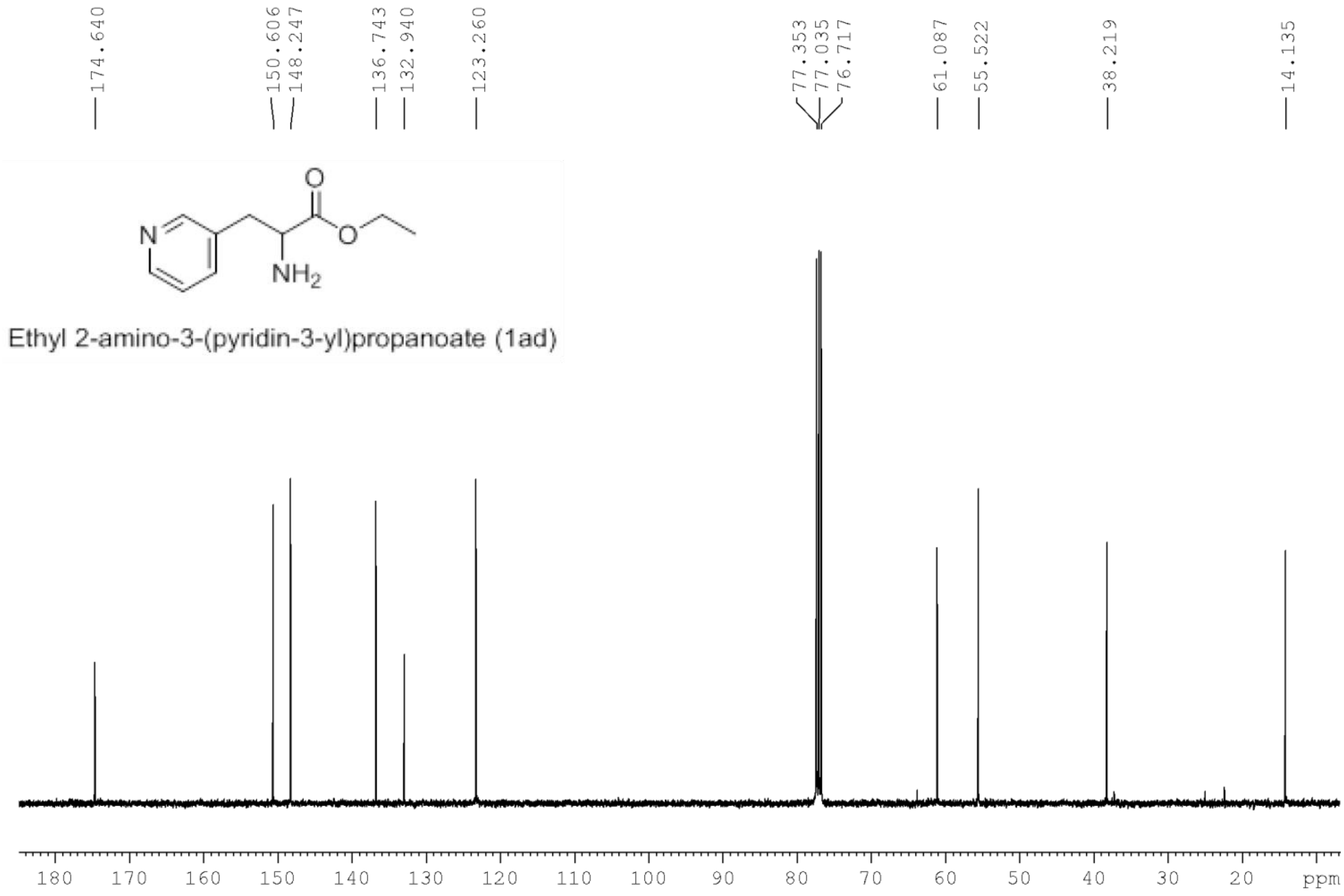

174.640
150.606
148.247
136.743
132.940
123.260
77.353
77.035
76.717
61.087
55.522
38.219
14.135
NH2
Ethyl 2-amino-3-(pyridin-3-yl)propanoate (1ad)
180 170 160 150 140 130 120 110 100 90 80 70 60 50 40 30 20 ppm

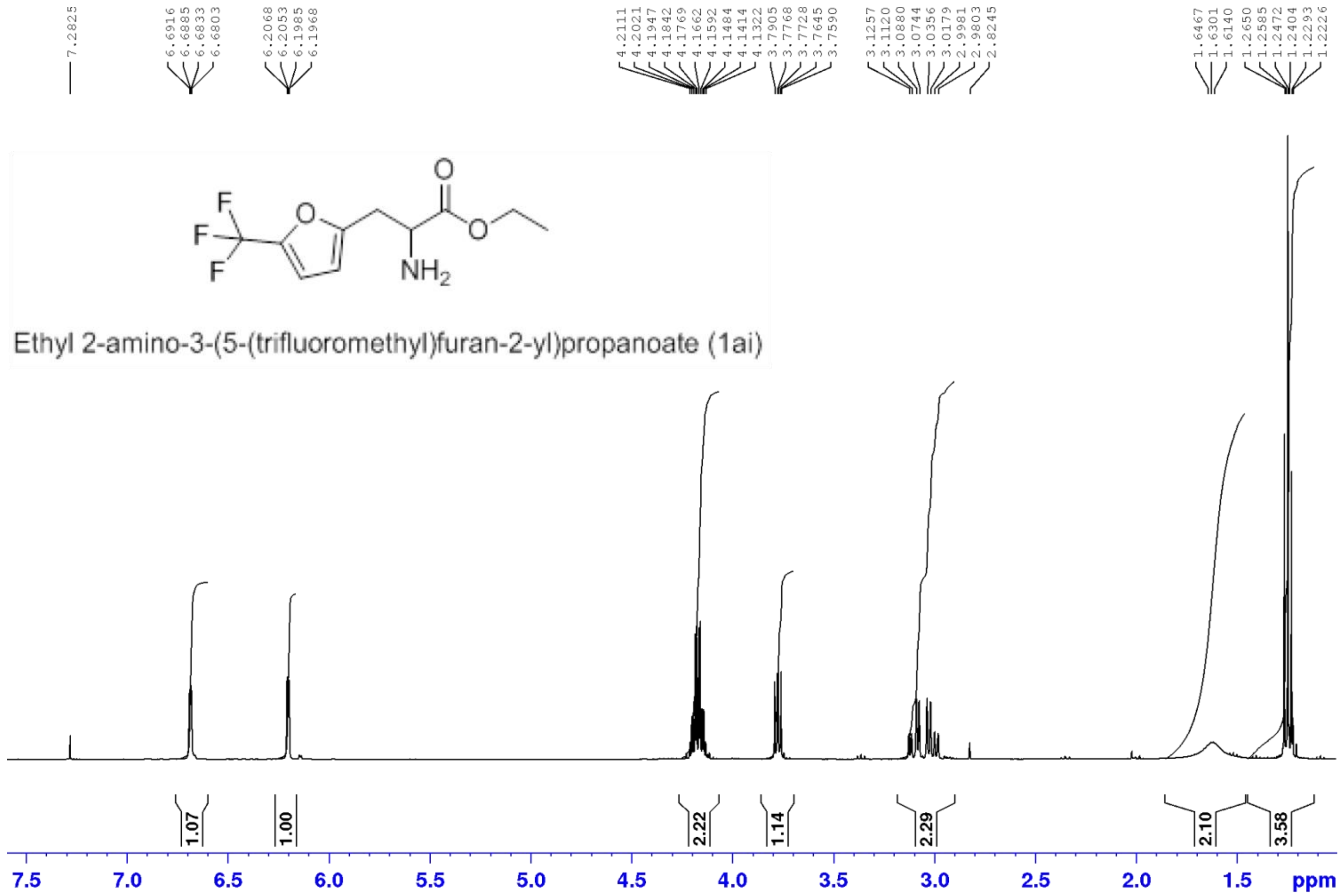
7.2825
6.6916
6.6885
6.6833
6.6803
6.2068
6.2053
6.1985
6.1968
4.2111
4.2021
4.1947
4.1842
4.1769
4.1662
4.1592
4.1484
4.1414
4.1322
3.7905
3.7768
3.7728
3.7645
3.7590
3.1257
3.1120
3.0880
3.0744
3.0356
3.0179
2.9981
2.9803
2.8245
1.6467
1.6301
1.6140
1.2650
1.2585
1.2472
1.2404
1.2293
1.2226
Ethyl 2-amino-3-(5-(trifluoromethyl)furan-2-yl)propanoate (1ai)
1.07
1.00
2.22
1.14
2.29
2.10
3.58
7.5
7.0
6.5
6.0
5.5
5.0
4.5
4.0
3.5
3.0
2.5
2.0
1.5
ppm

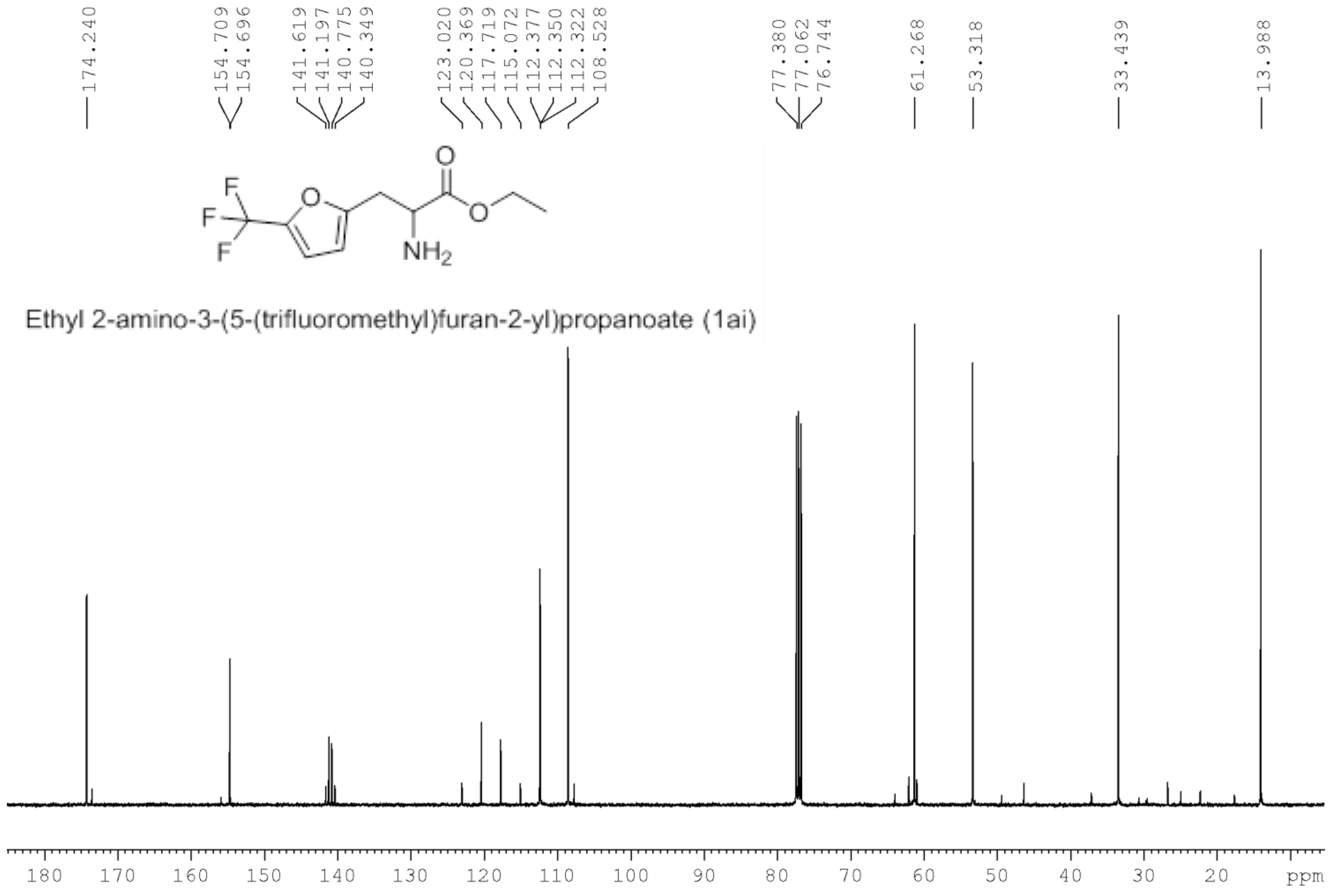
174.240
154.709
154.696
141.619
141.197
140.775
140.349
123.020
120.369
117.719
115.072
112.377
112.350
112.322
108.528
77.380
77.062
76.744
61.268
53.318
33.439
13.988
Ethyl 2-amino-3-(5-(trifluoromethyl)furan-2-yl)propanoate (1ai)
180
170
160
150
140
130
120
110
100
90
80
70
60
50
40
30
20
ppm

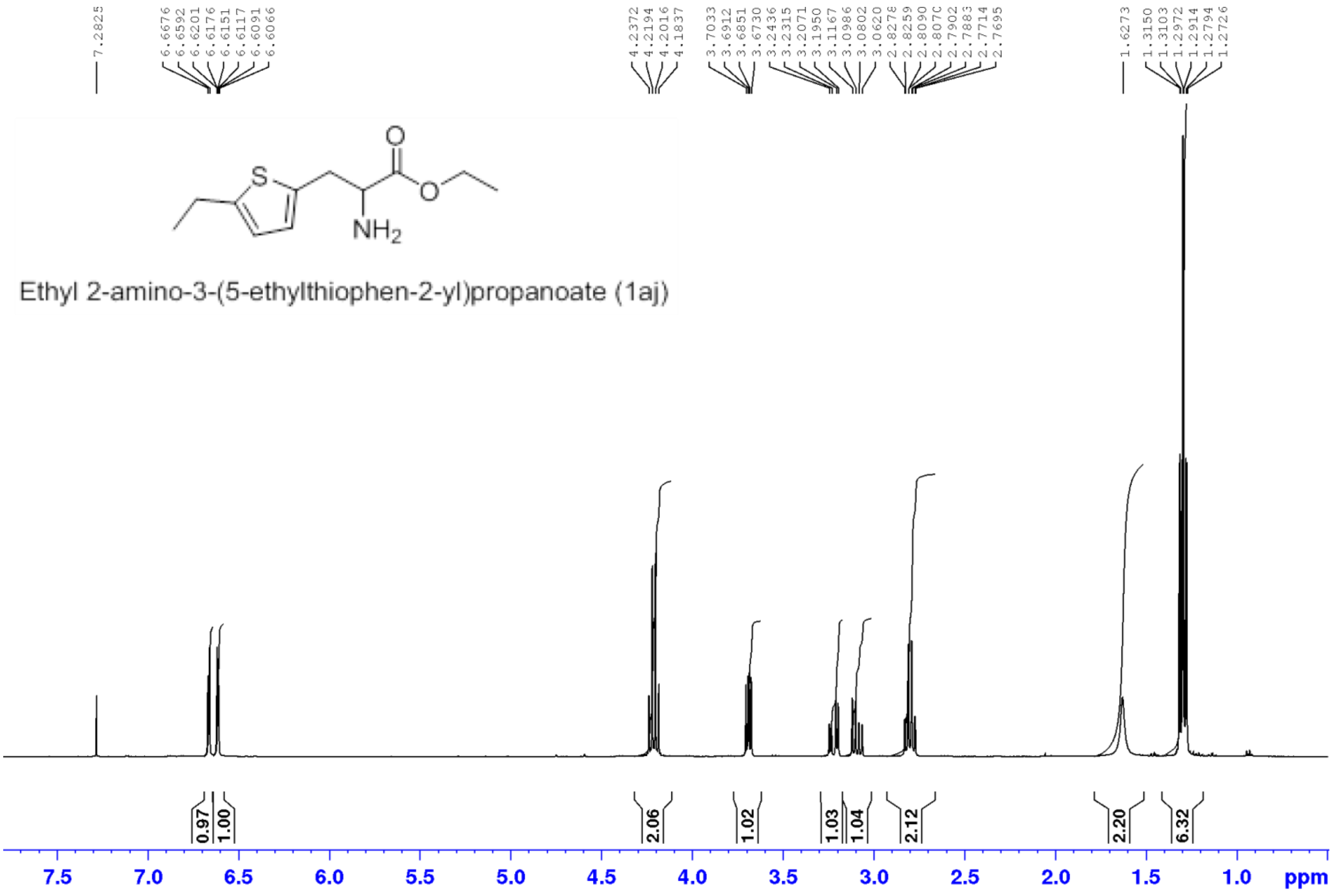

Ethyl 2-amino-3-(5-ethylthiophen-2-yl)propanoate (1aj)

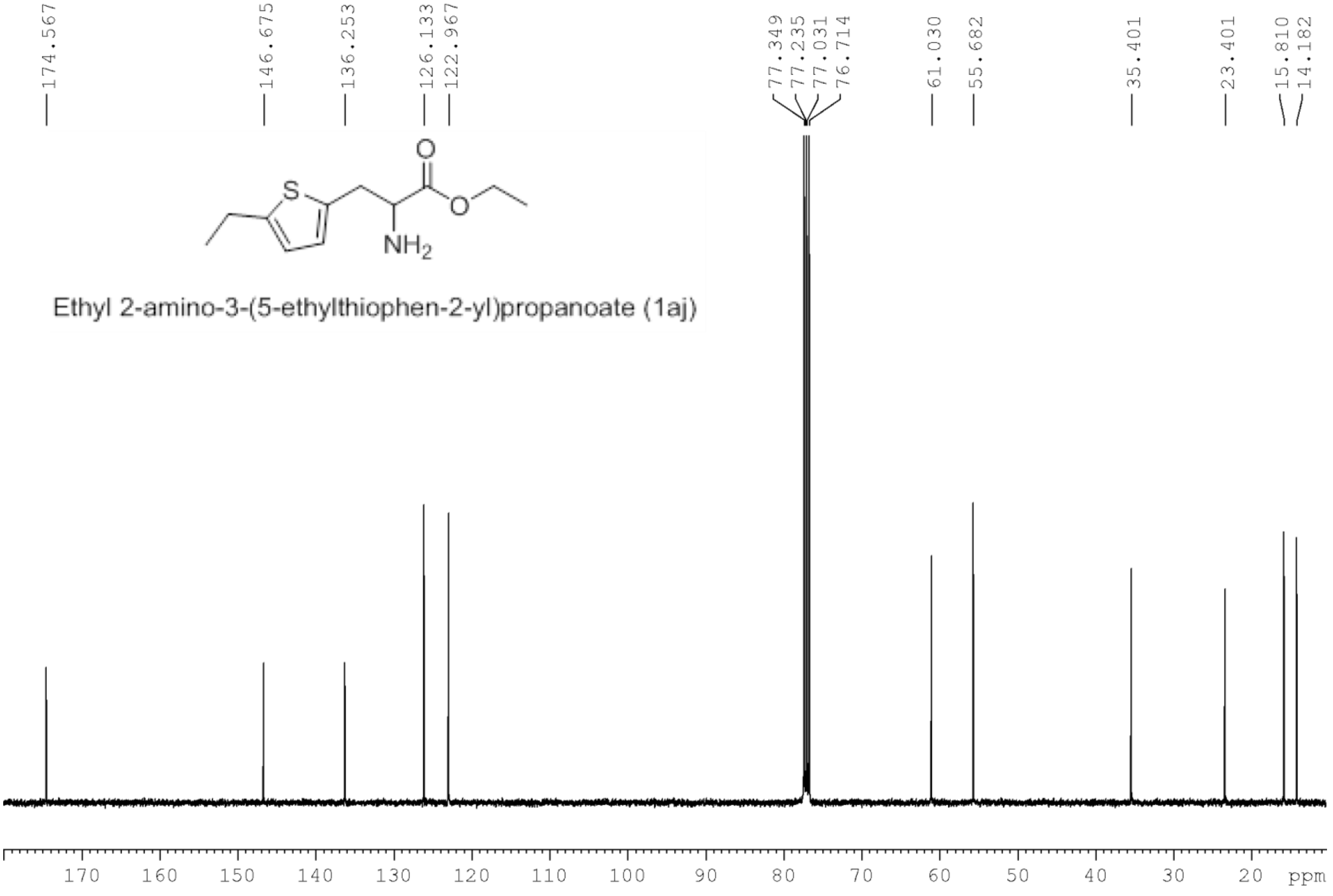

Ethyl 2-amino-3-(5-ethylthiophen-2-yl)propanoate (1aj)

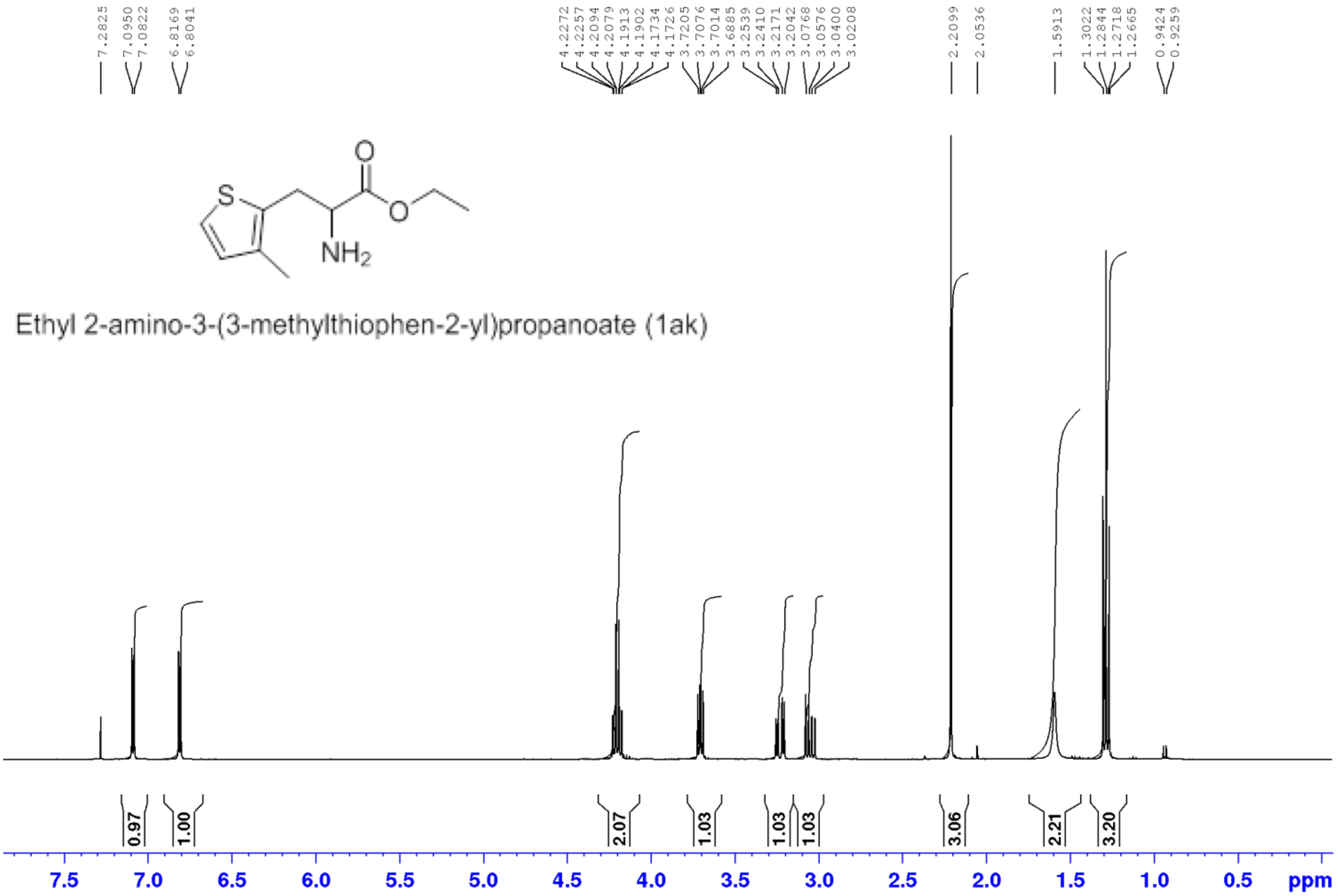
Ethyl 2-amino-3-(3-methylthiophen-2-yl)propanoate (1ak)
7.2825
7.0950
7.0822
6.8169
6.8041
4.2272
4.2257
4.2094
4.2079
4.1913
4.1902
4.1734
4.1726
3.7205
3.7076
3.7014
3.6885
3.2539
3.2410
3.2171
3.2042
3.0768
3.0576
3.0400
3.0208
2.2099
2.0536
1.5913
1.3022
1.2844
1.2718
1.2665
0.9424
0.9259
0.97
1.00
2.07
1.03
1.03
1.03
3.06
2.21
3.20
ppm

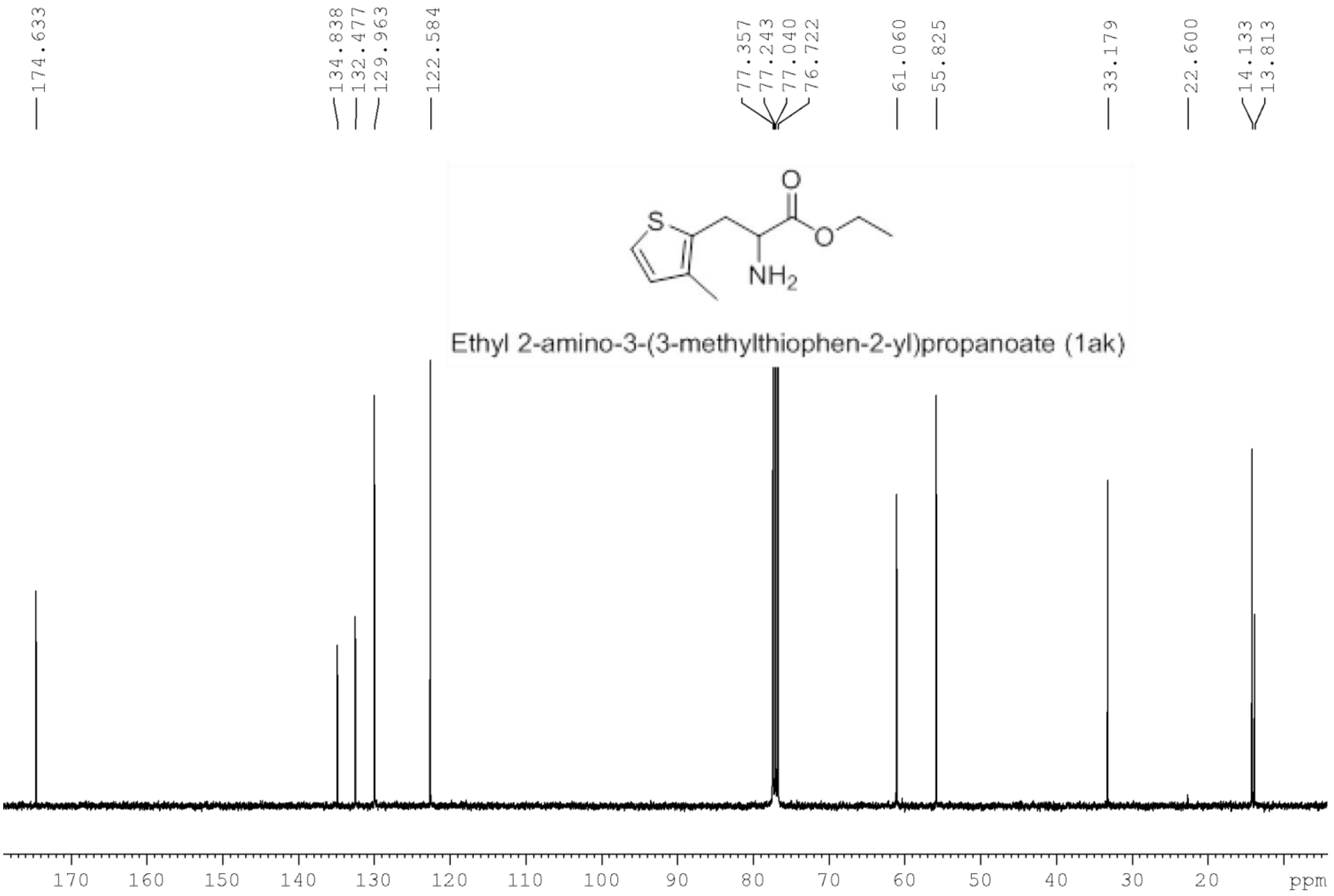
Ethyl 2-amino-3-(3-methylthiophen-2-yl)propanoate (1ak)
174.633
134.838
132.477
129.963
122.584
77.357
77.243
77.040
76.722
61.060
55.825
33.179
22.600
14.133
13.813
ppm

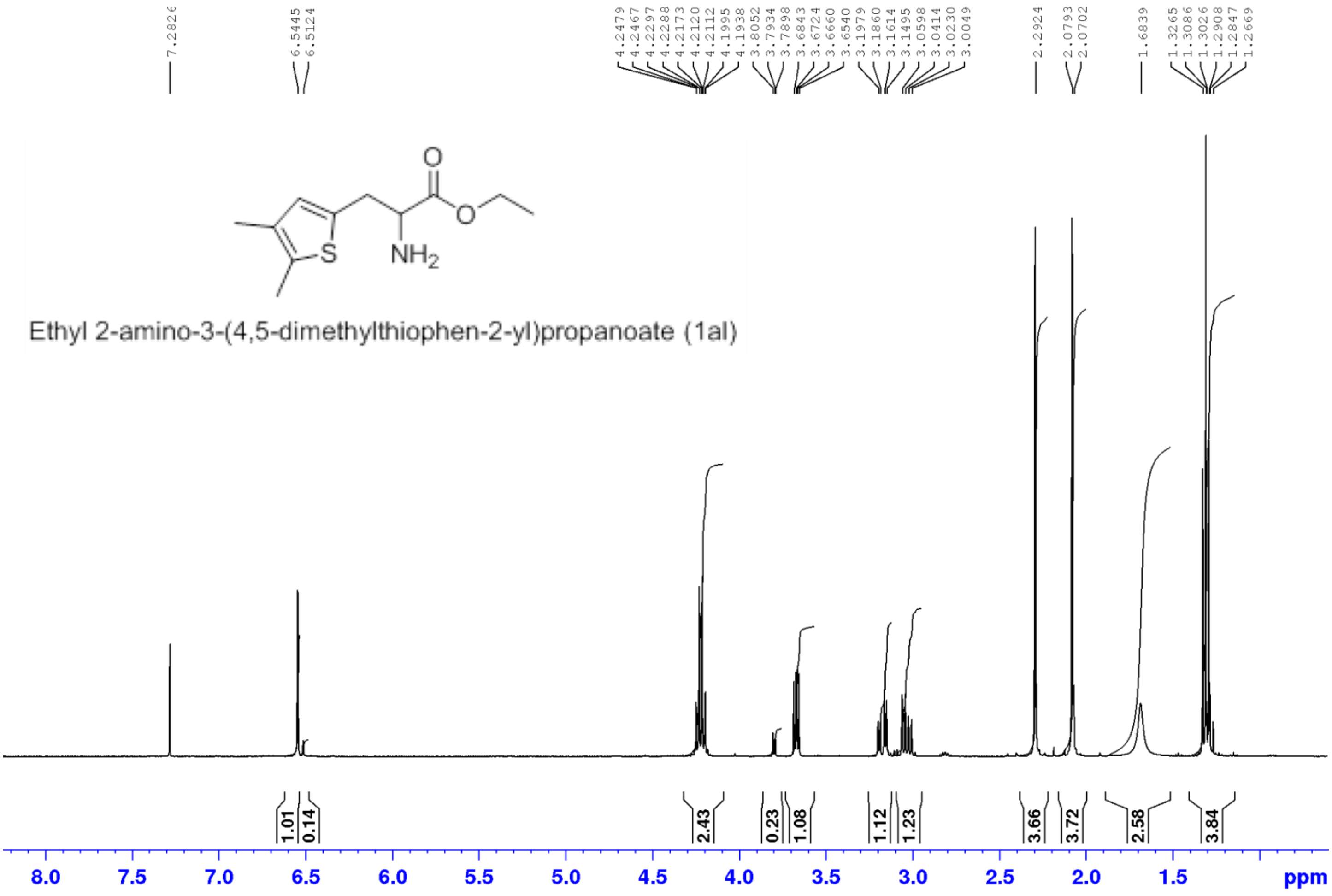
Ethyl 2-amino-3-(4,5-dimethylthiophen-2-yl)propanoate (1al)
S
NH2
O
7.2826
6.5445
6.5124
4.2479
4.2467
4.2297
4.2288
4.2173
4.2120
4.2112
4.1995
4.1938
3.8052
3.7934
3.7898
3.6843
3.6724
3.6660
3.6540
3.1979
3.1860
3.1614
3.1495
3.0598
3.0414
3.0230
3.0049
2.2924
2.0793
2.0702
1.6839
1.3265
1.3086
1.3026
1.2908
1.2847
1.2669
1.01
0.14
2.43
0.23
1.08
1.12
1.23
3.66
3.72
2.58
3.84
8.0
7.5
7.0
6.5
6.0
5.5
5.0
4.5
4.0
3.5
3.0
2.5
2.0
1.5
ppm

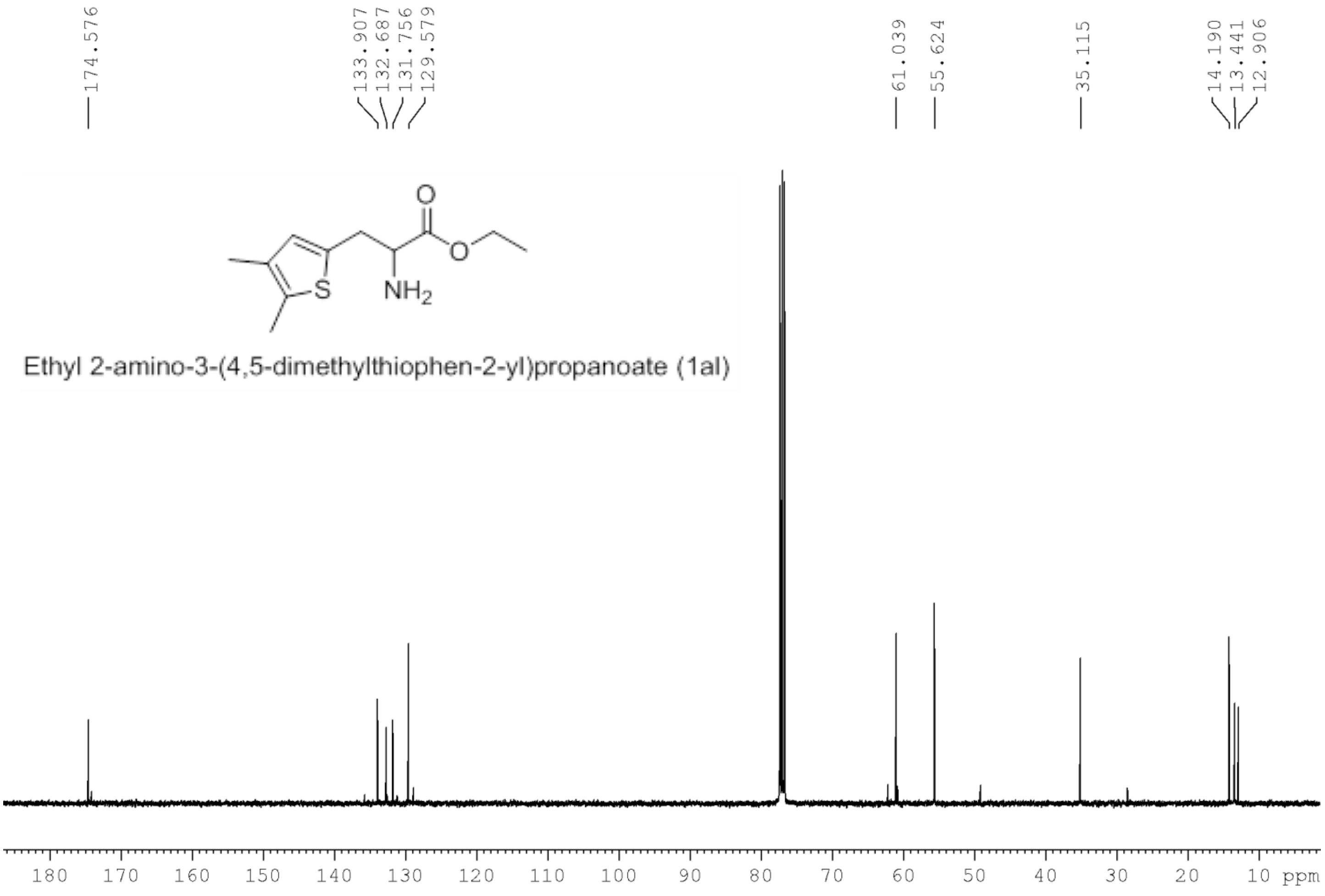
Ethyl 2-amino-3-(4,5-dimethylthiophen-2-yl)propanoate (1al)
S
NH2
O
174.576
133.907
132.687
131.756
129.579
61.039
55.624
35.115
14.190
13.441
12.906
180
170
160
150
140
130
120
110
100
90
80
70
60
50
40
30
20
10 ppm

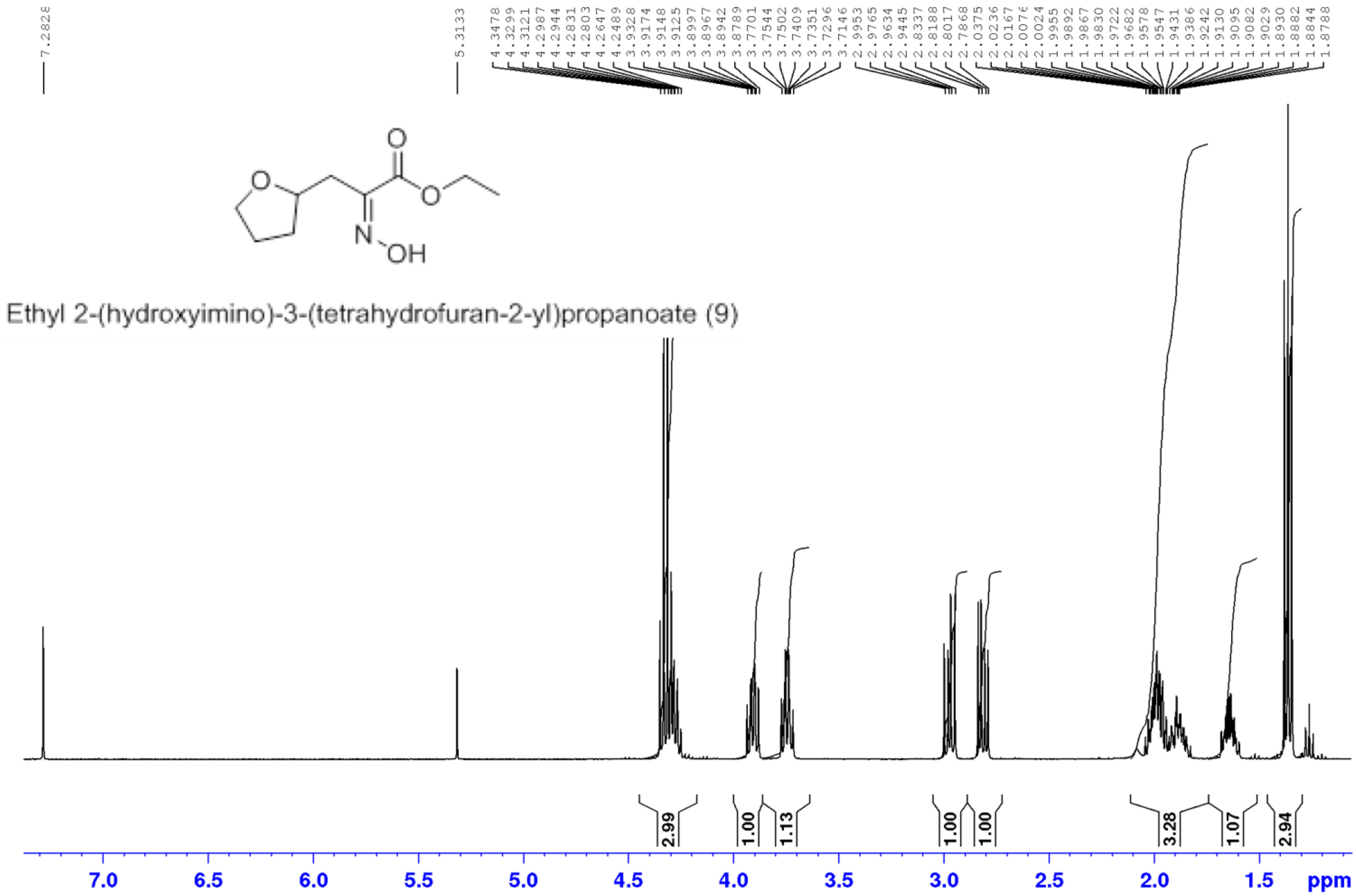
Ethyl 2-(hydroxyimino)-3-(tetrahydrofuran-2-yl)propanoate (9)
7.2828
5.3133
4.3478 4.3299 4.3121 4.2987 4.2944 4.2831 4.2803 4.2647 4.2489 3.9328 3.9174 3.9148 3.9125 3.8997 3.8967 3.8942 3.8789 3.7701 3.7544 3.7502 3.7409 3.7351 3.7296 3.7146 2.9953 2.9765 2.9634 2.9445 2.8337 2.8188 2.8017 2.7868 2.0375 2.0236 2.0167 2.0076 2.0024 1.9955 1.9892 1.9867 1.9830 1.9722 1.9682 1.9578 1.9547 1.9431 1.9386 1.9242 1.9130 1.9095 1.9082 1.9029 1.8930 1.8882 1.8844 1.8788
2.99
1.00
1.13
1.00
1.00
3.28
1.07
2.94
7.0 6.5 6.0 5.5 5.0 4.5 4.0 3.5 3.0 2.5 2.0 1.5 ppm

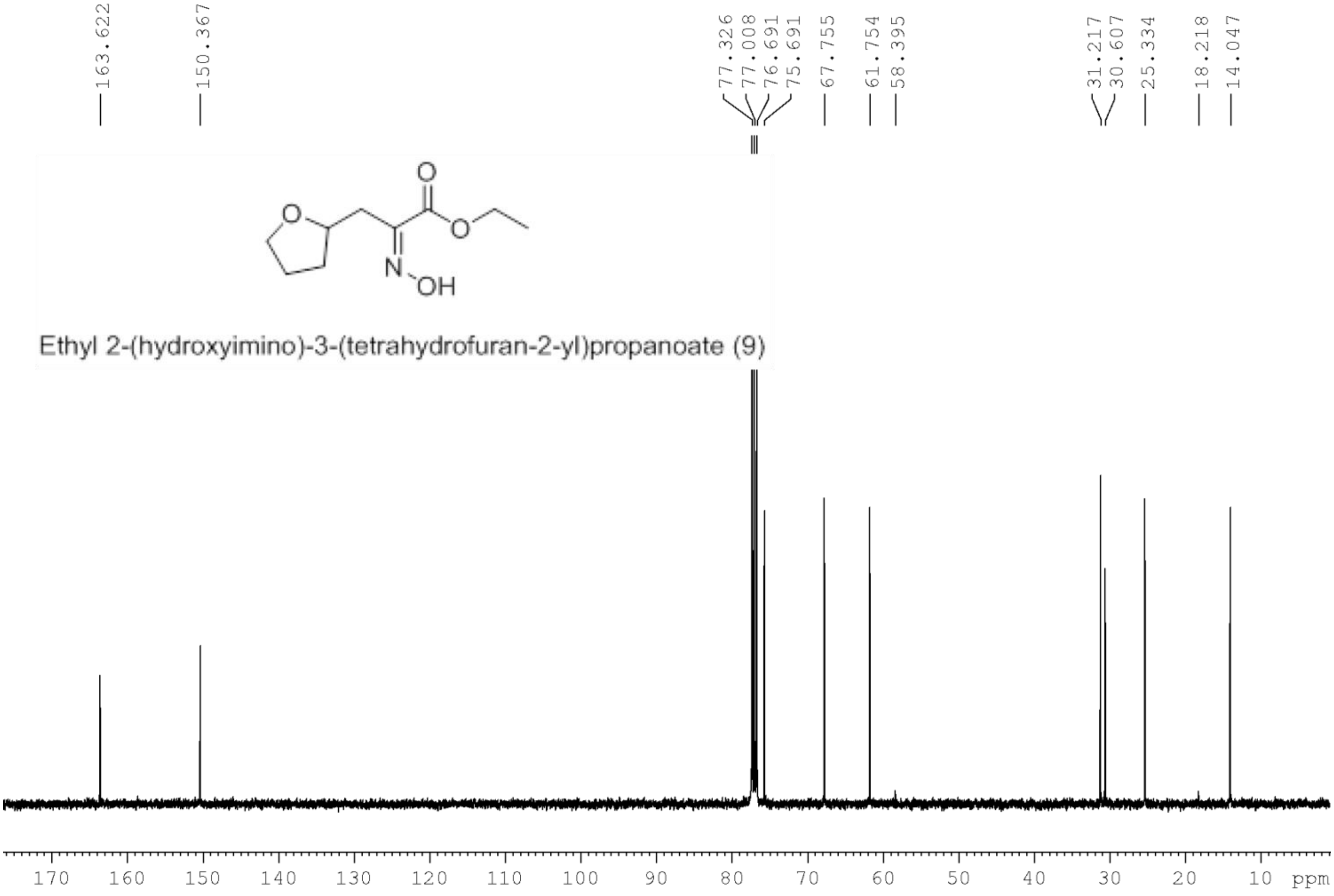
163.622
150.367
77.326 77.008 76.691 75.691
67.755
61.754 58.395
31.217 30.607
25.334
18.218
14.047
Ethyl 2-(hydroxyimino)-3-(tetrahydrofuran-2-yl)propanoate (9)
170 160 150 140 130 120 110 100 90 80 70 60 50 40 30 20 10 ppm

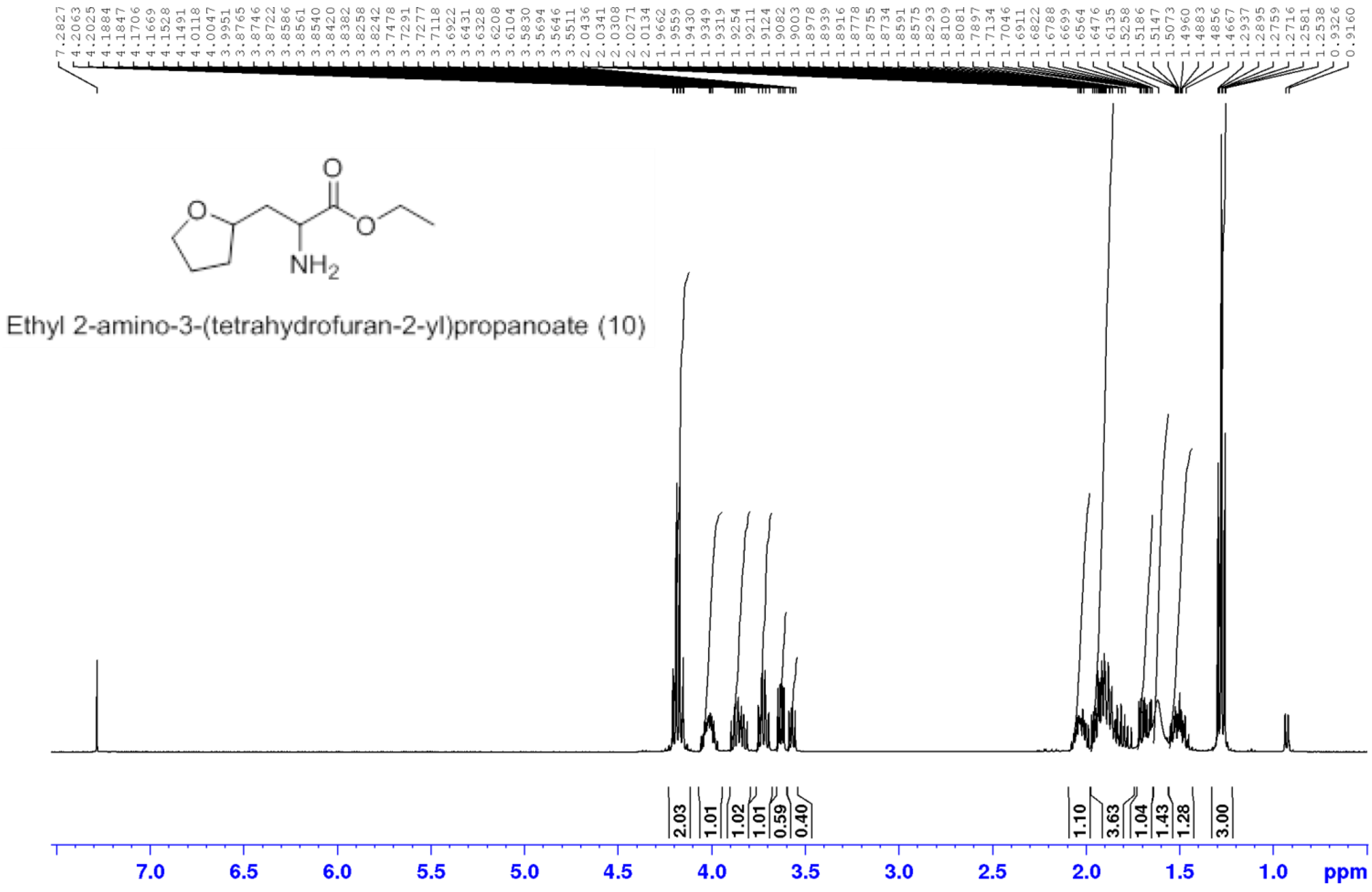
Ethyl 2-amino-3-(tetrahydrofuran-2-yl)propanoate (10)
ppm

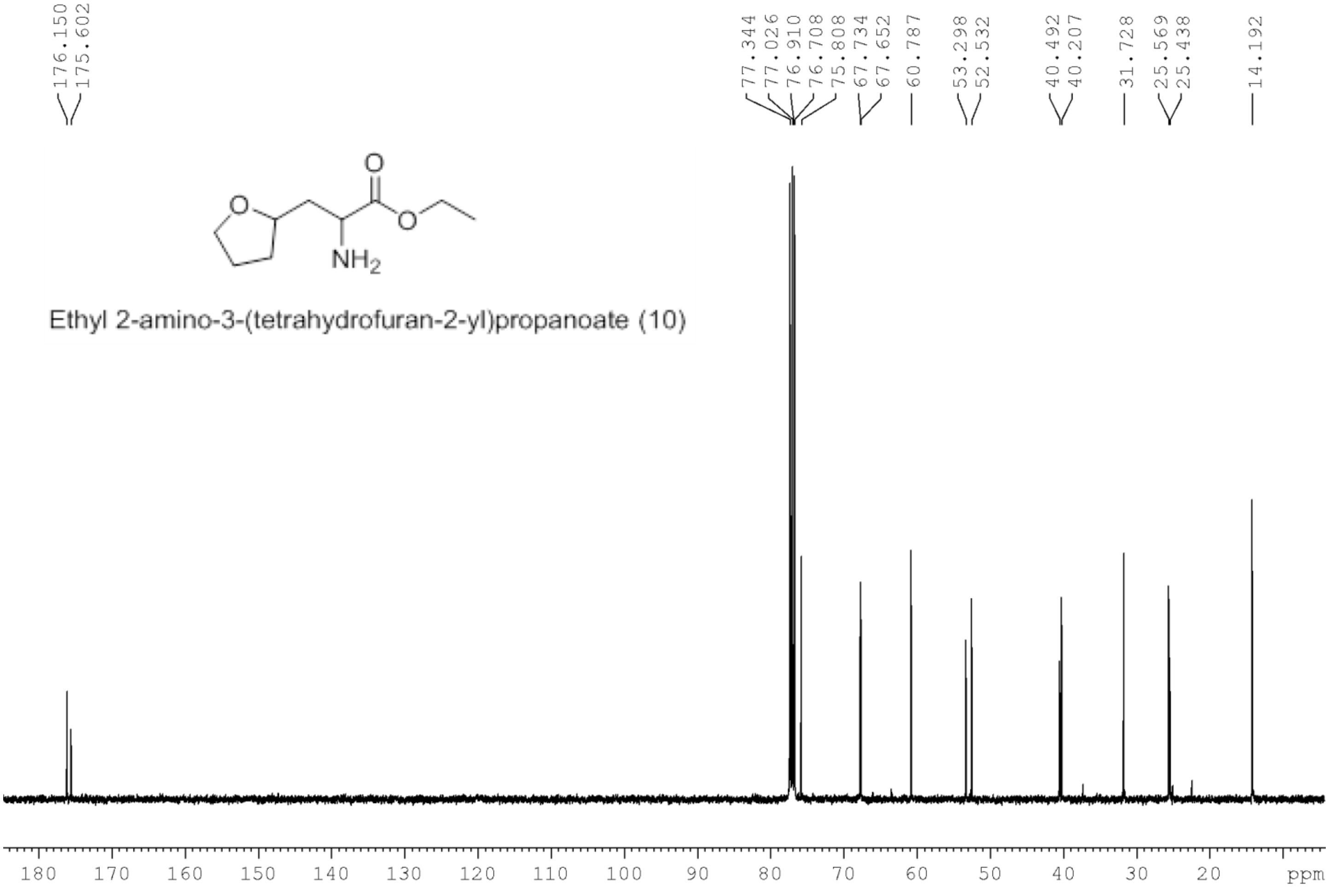
Ethyl 2-amino-3-(tetrahydrofuran-2-yl)propanoate (10)
ppm

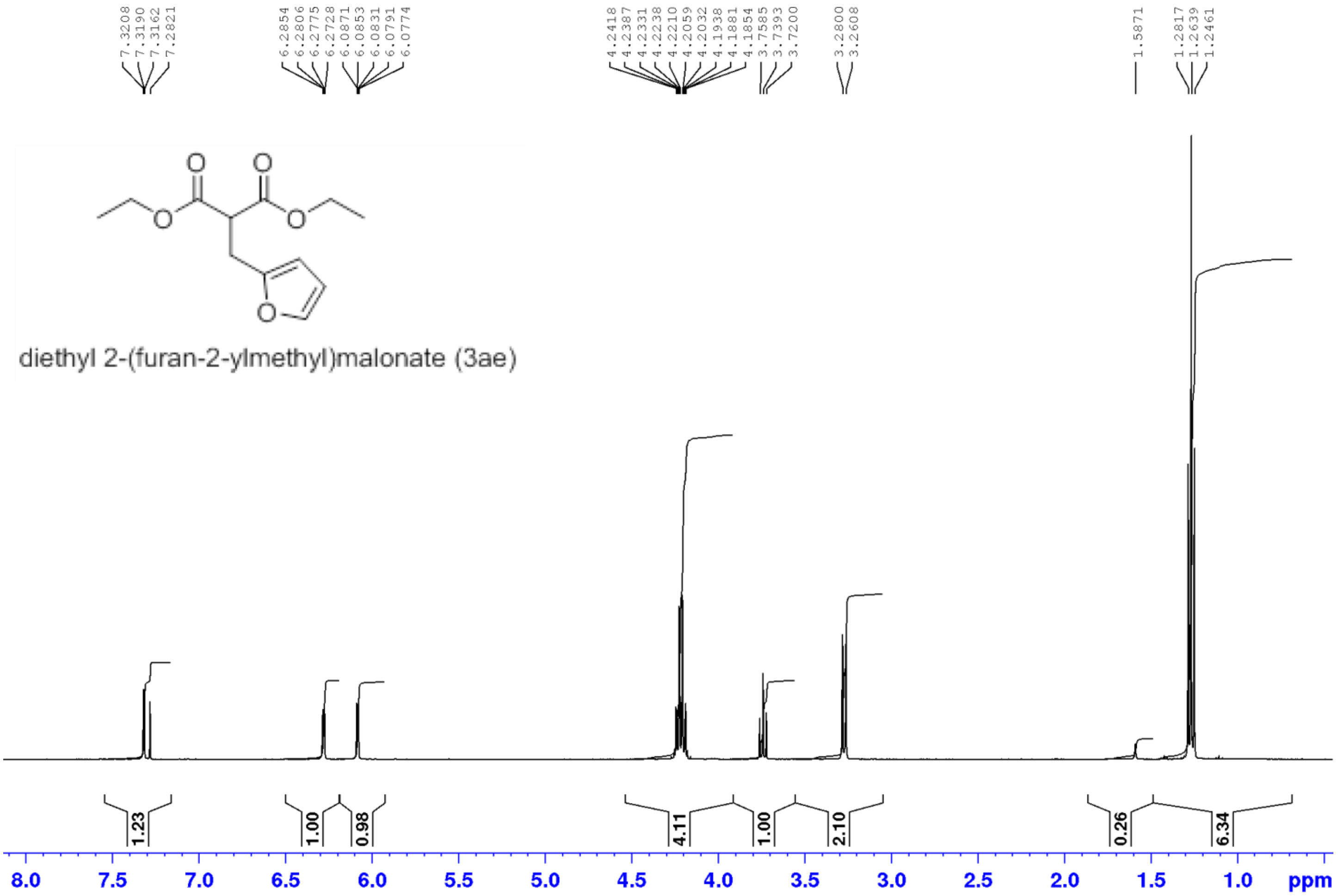

diethyl 2-(furan-2-ylmethyl)malonate (3ae)

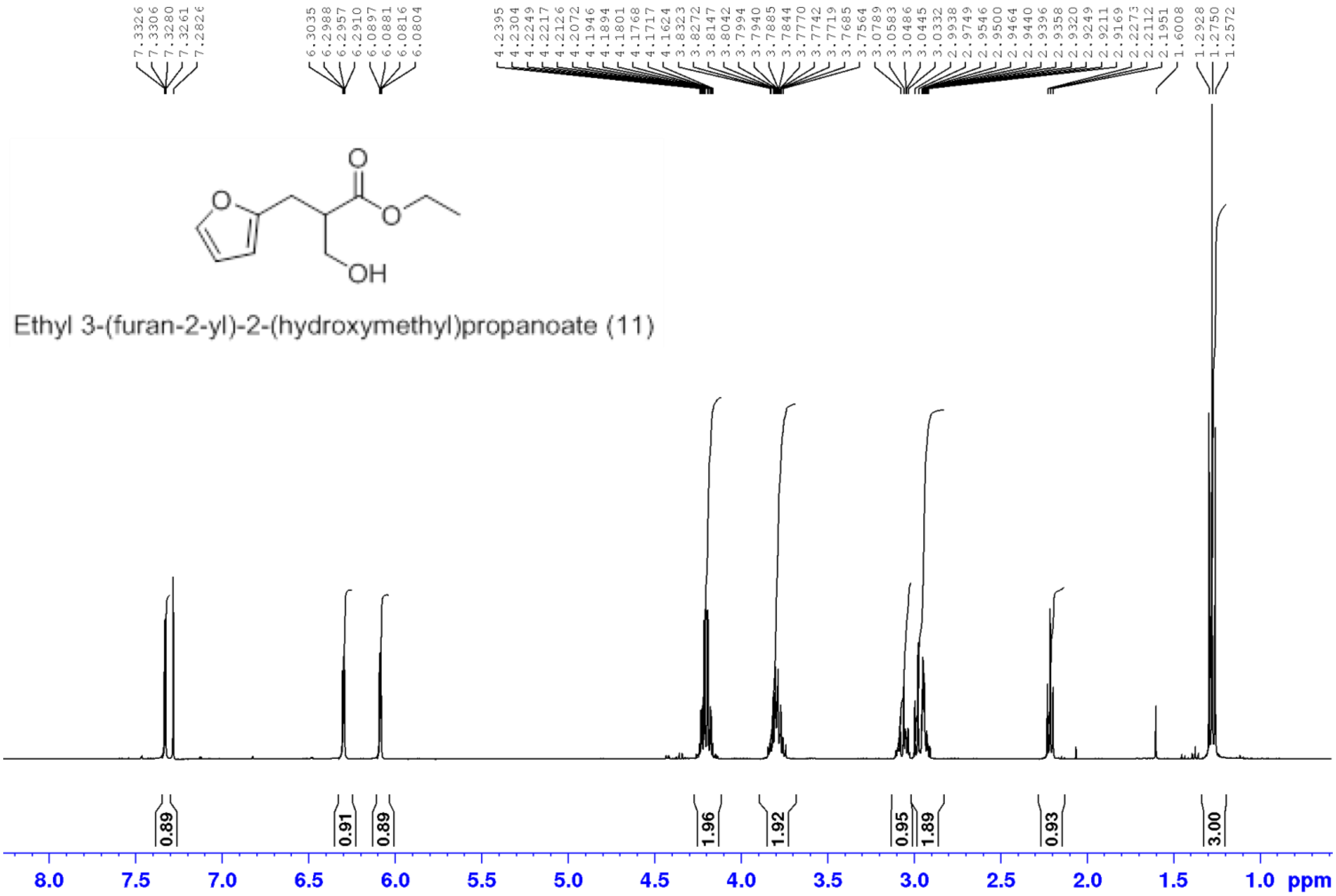

Ethyl 3-(furan-2-yl)-2-(hydroxymethyl)propanoate (11)

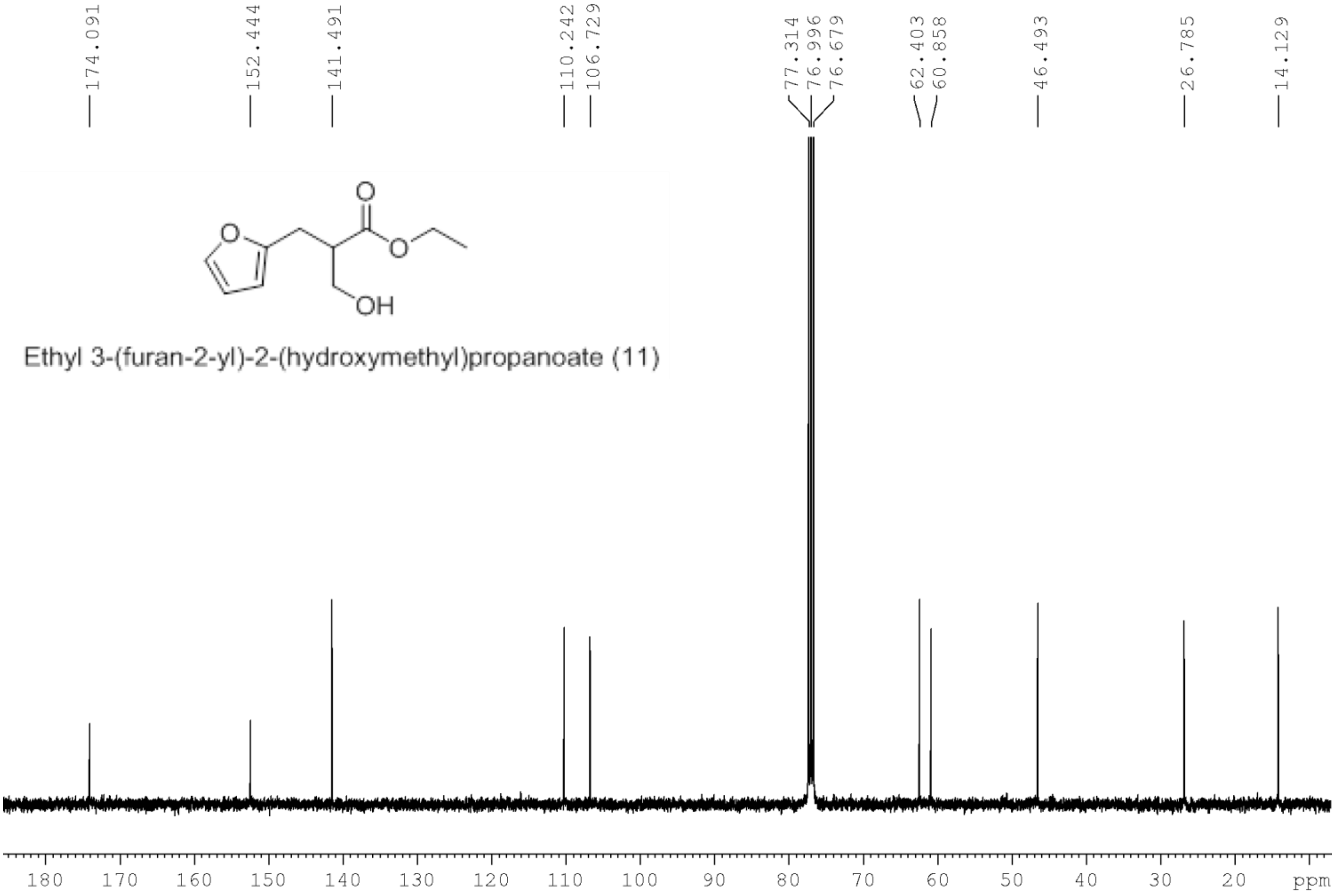

Ethyl 3-(furan-2-yl)-2-(hydroxymethyl)propanoate (11)

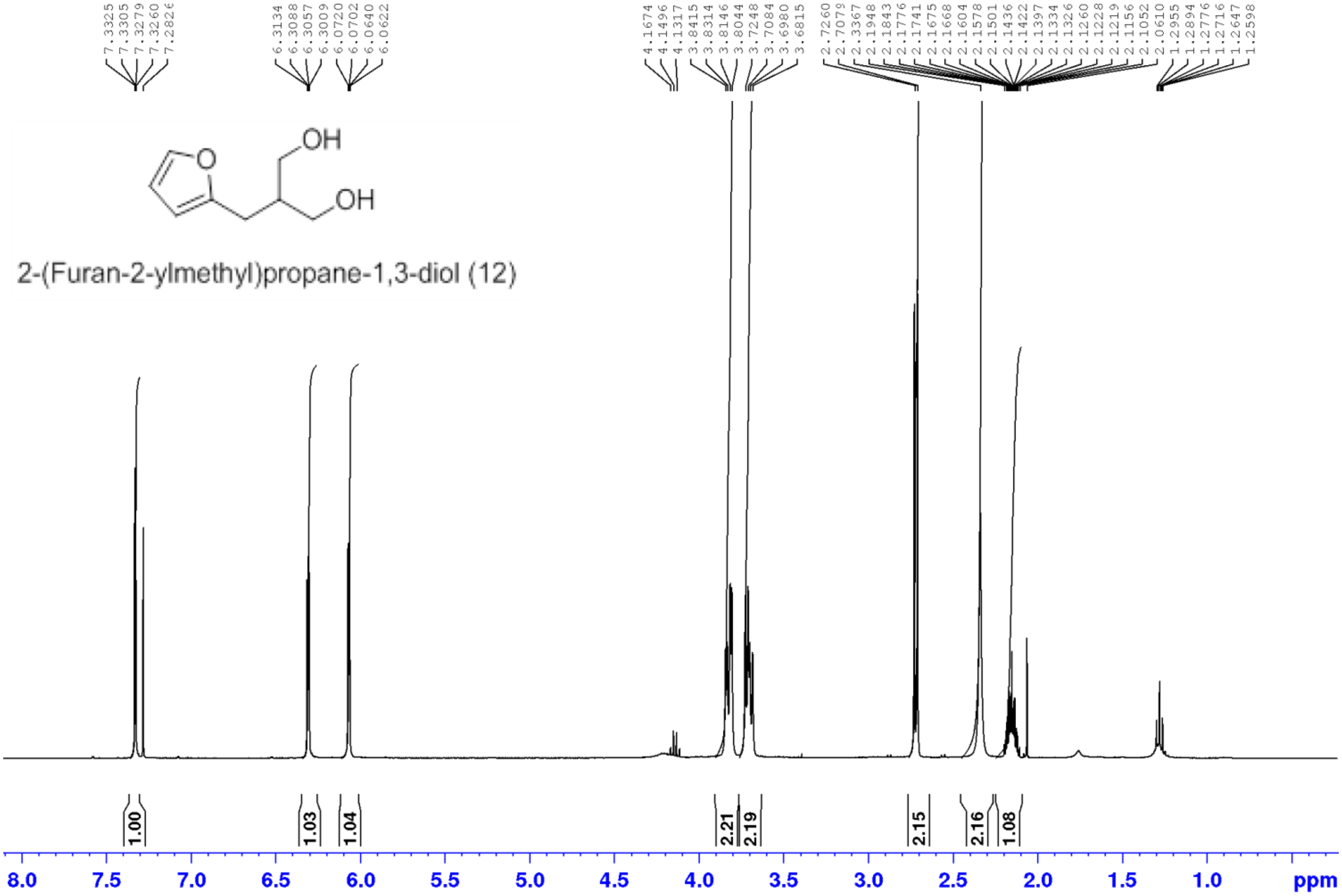

2-(Furan-2-ylmethyl)propane-1,3-diol (12)

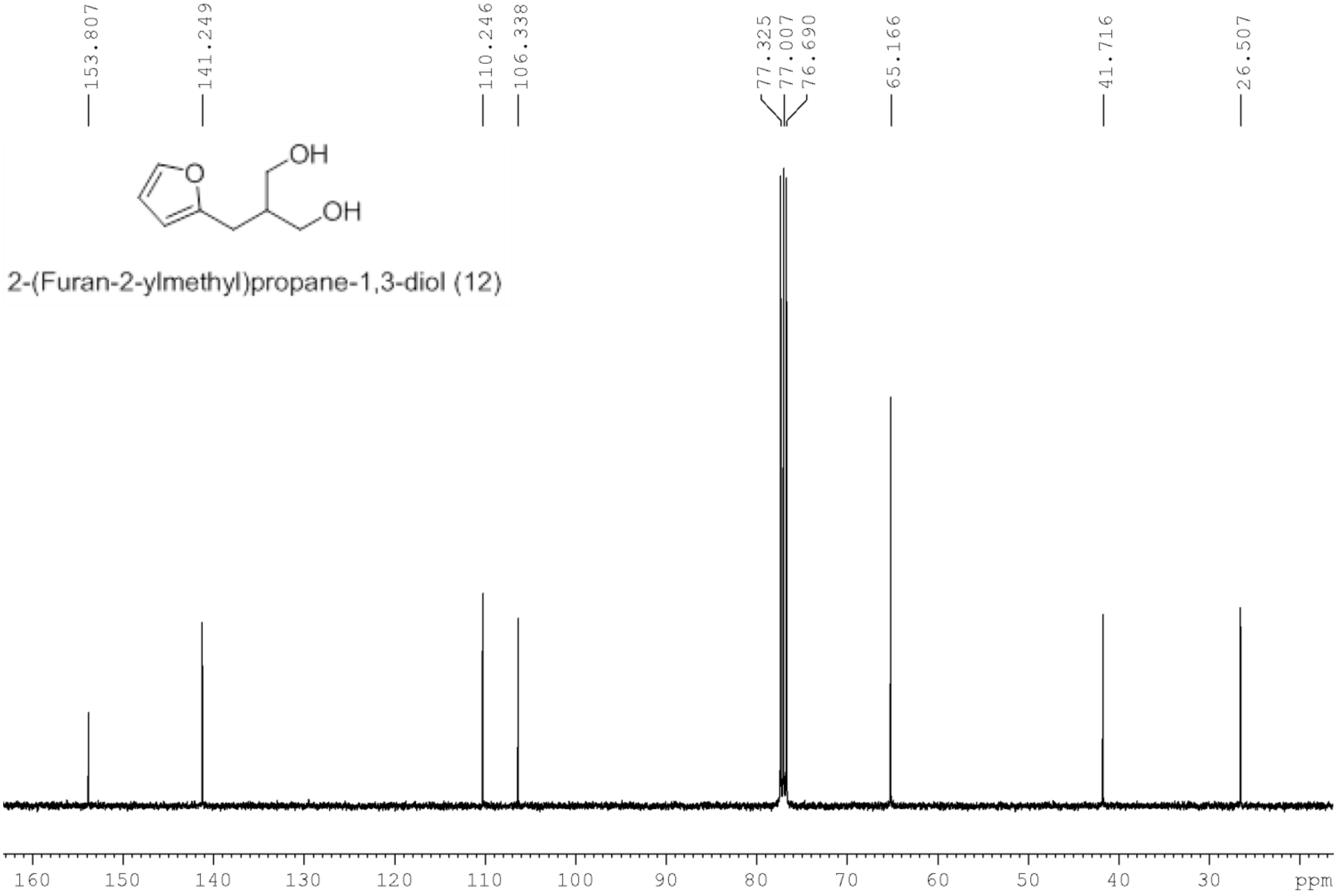

2-(Furan-2-ylmethyl)propane-1,3-diol (12)

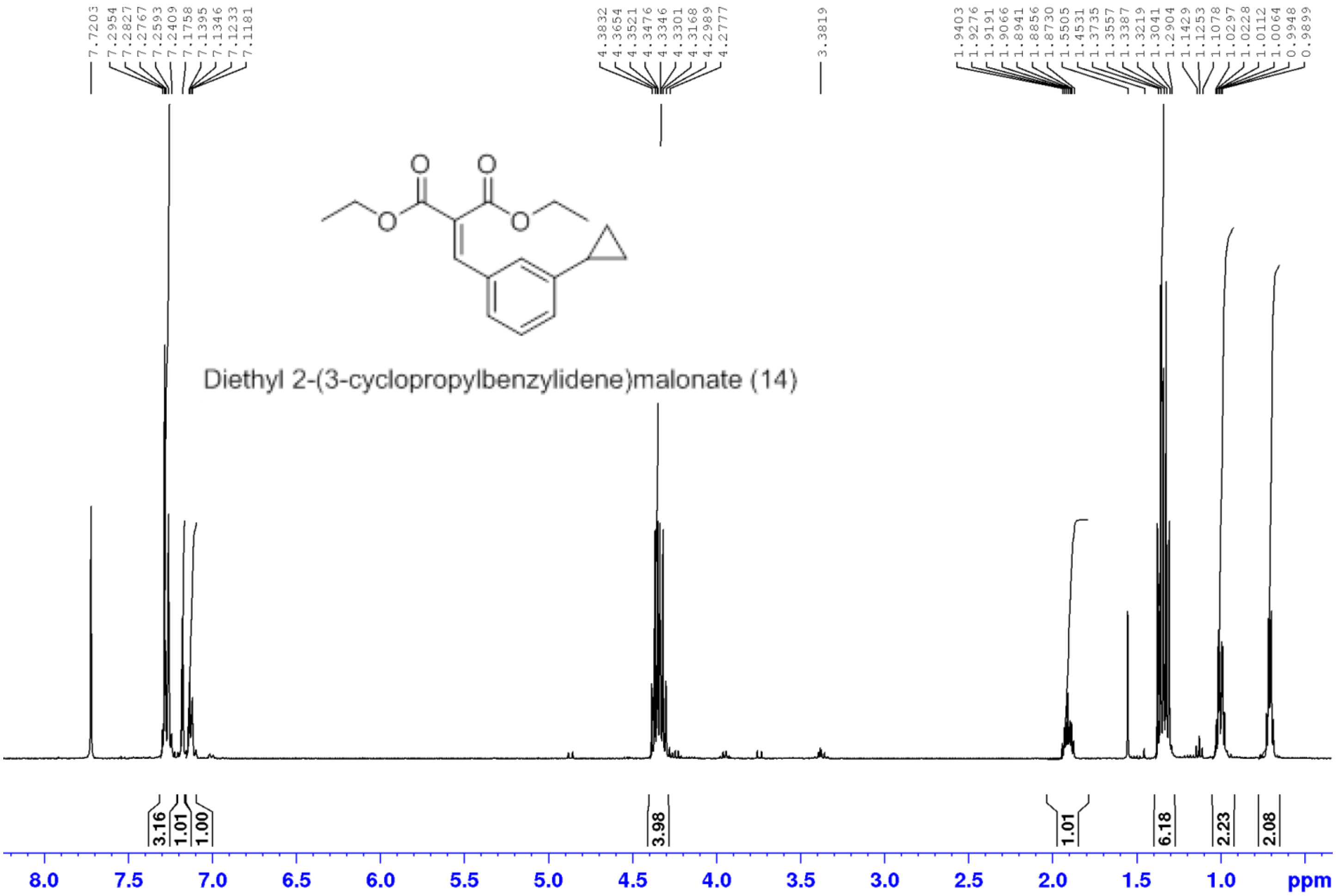
7.7203
7.2954
7.2827
7.2767
7.2593
7.2409
7.1758
7.1395
7.1346
7.1233
7.1181
4.3832
4.3654
4.3521
4.3476
4.3346
4.3301
4.3168
4.2989
4.2777
3.3819
1.9403
1.9276
1.9191
1.9066
1.8941
1.8856
1.8730
1.5505
1.4531
1.3735
1.3557
1.3387
1.3219
1.3041
1.2904
1.1429
1.1253
1.1078
1.0297
1.0228
1.0112
1.0064
0.9948
0.9899
Diethyl 2-(3-cyclopropylbenzylidene)malonate (14)
3.16
1.01
1.00
3.98
1.01
6.18
2.23
2.08
8.0
7.5
7.0
6.5
6.0
5.5
5.0
4.5
4.0
3.5
3.0
2.5
2.0
1.5
1.0
ppm

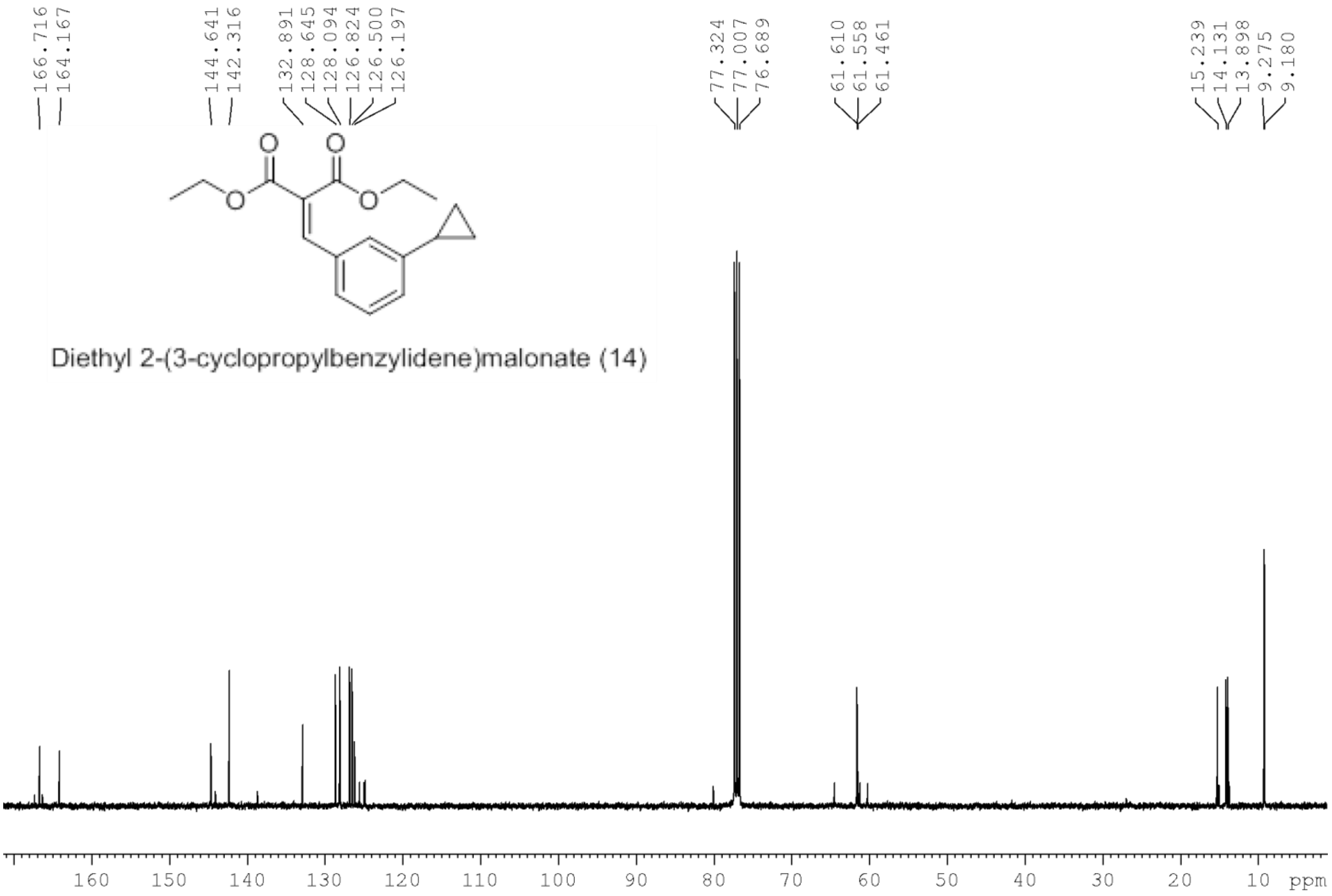
166.716
164.167
144.641
142.316
132.891
128.645
128.094
126.824
126.500
126.197
77.324
77.007
76.689
61.610
61.558
61.461
15.239
14.131
13.898
9.275
9.180
Diethyl 2-(3-cyclopropylbenzylidene)malonate (14)
160
150
140
130
120
110
100
90
80
70
60
50
40
30
20
10
ppm

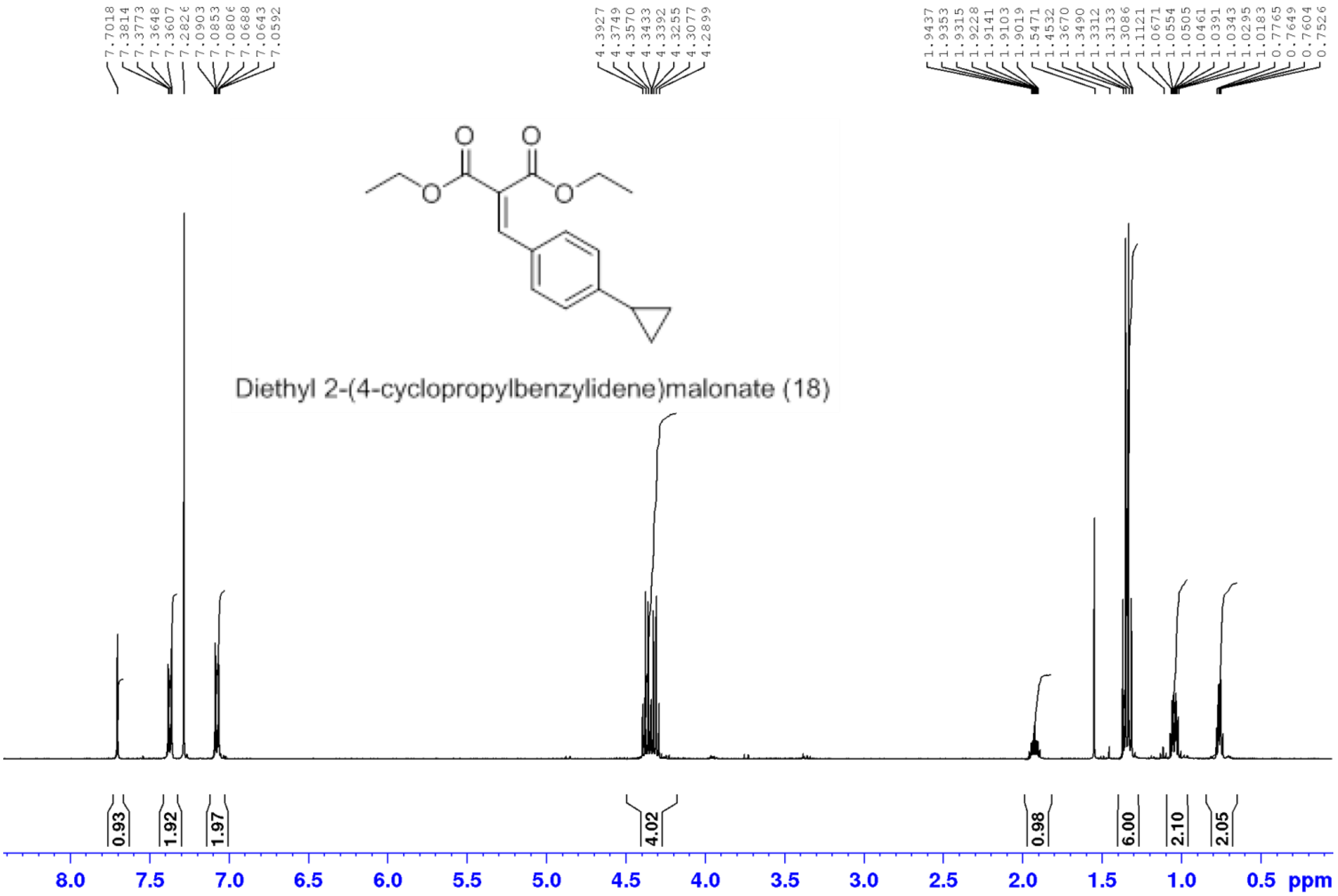
7.7018
7.3814
7.3773
7.3648
7.3607
7.2826
7.0903
7.0853
7.0806
7.0688
7.0643
7.0592
4.3927
4.3749
4.3570
4.3433
4.3392
4.3255
4.3077
4.2899
1.9437
1.9353
1.9315
1.9228
1.9141
1.9103
1.9019
1.5471
1.4532
1.3670
1.3490
1.3312
1.3133
1.3086
1.1121
1.0671
1.0554
1.0505
1.0461
1.0391
1.0343
1.0295
1.0183
0.7765
0.7649
0.7604
0.7526
Diethyl 2-(4-cyclopropylbenzylidene)malonate (18)
0.93
1.92
1.97
4.02
0.98
6.00
2.10
2.05
8.0
7.5
7.0
6.5
6.0
5.5
5.0
4.5
4.0
3.5
3.0
2.5
2.0
1.5
1.0
0.5 ppm

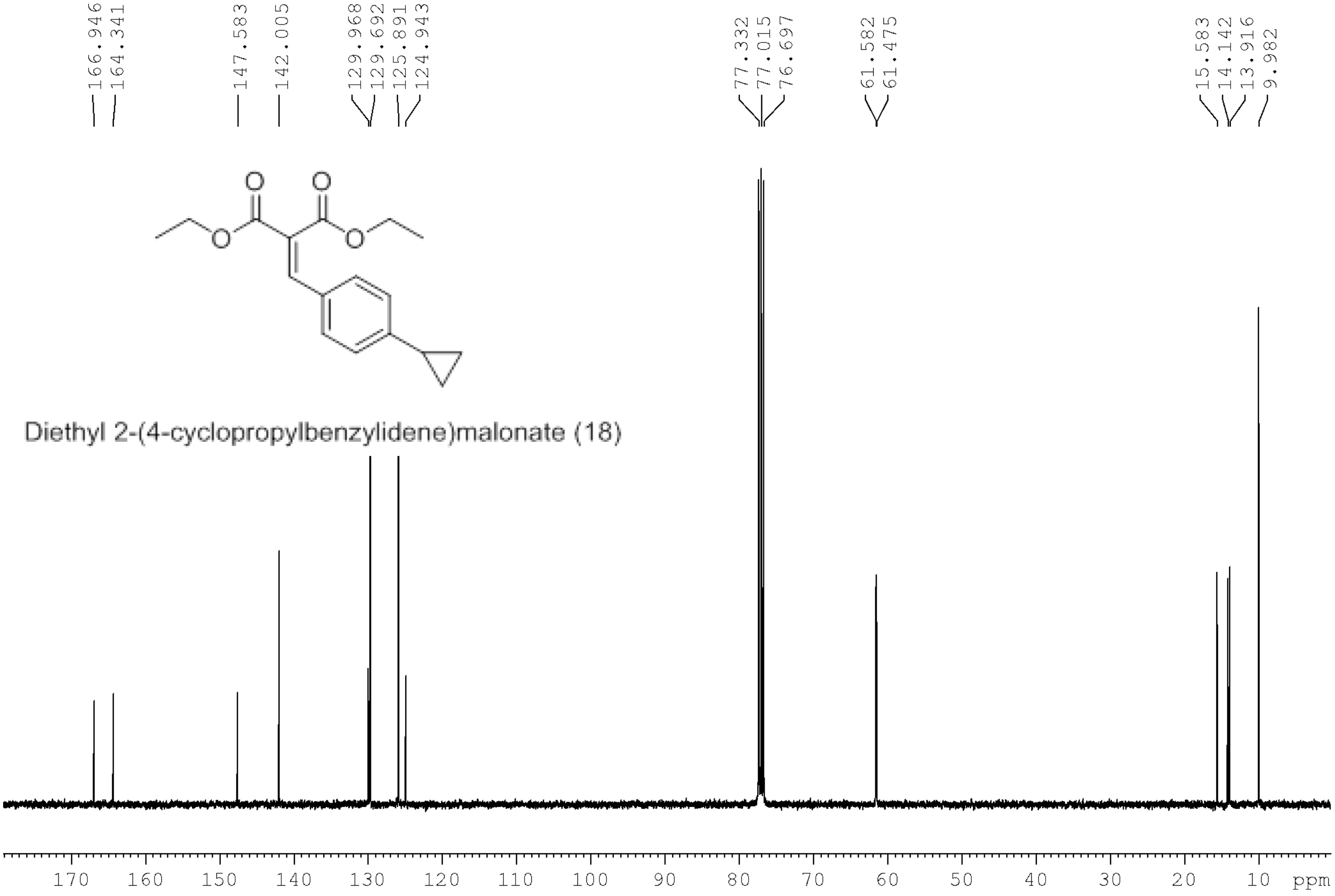
166.946
164.341
147.583
142.005
129.968
129.692
125.891
124.943
77.332
77.015
76.697
61.582
61.475
15.583
14.142
13.916
9.982
Diethyl 2-(4-cyclopropylbenzylidene)malonate (18)
170
160
150
140
130
120
110
100
90
80
70
60
50
40
30
20
10 ppm

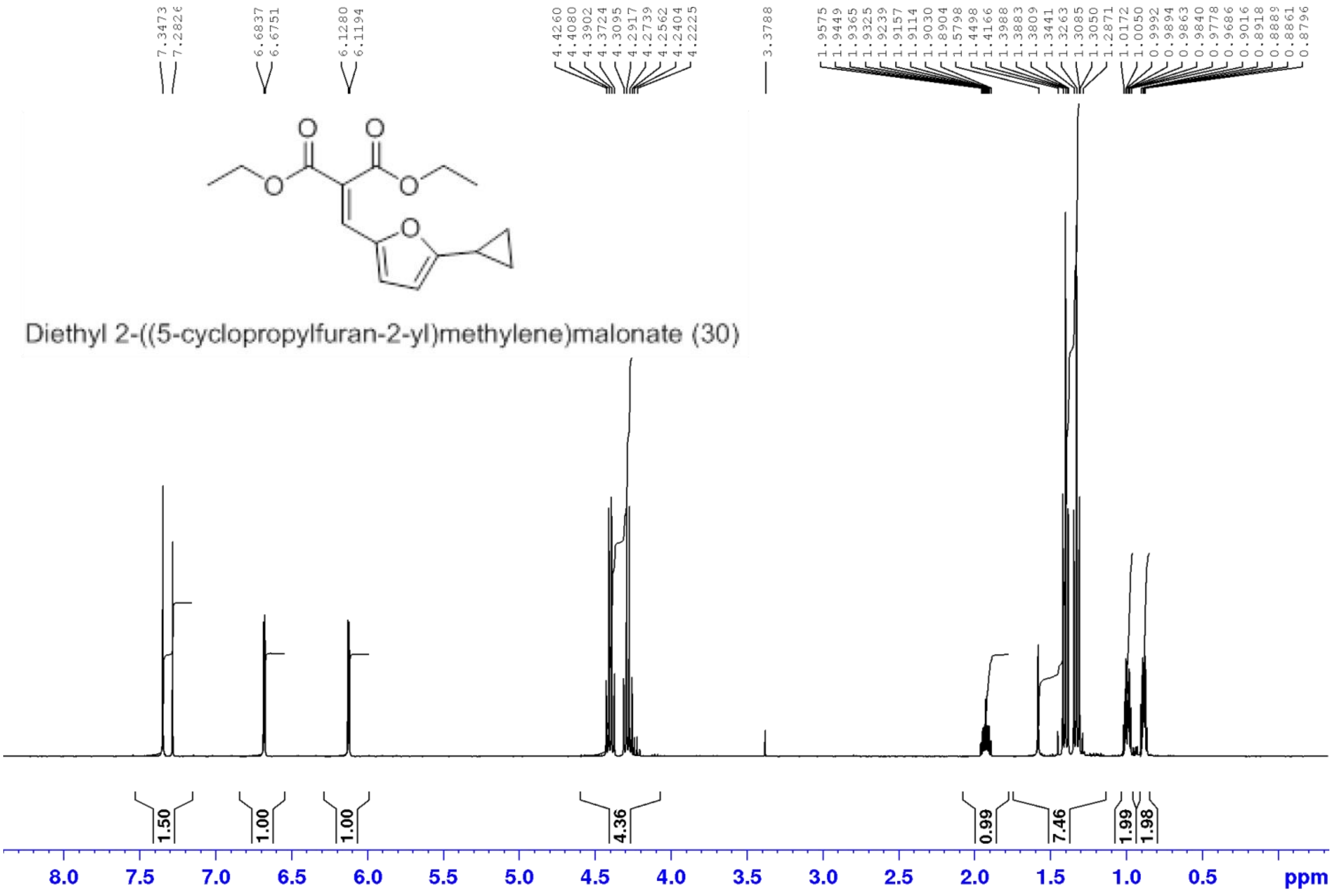

Diethyl 2-((5-cyclopropylfuran-2-yl)methylene)malonate (30)

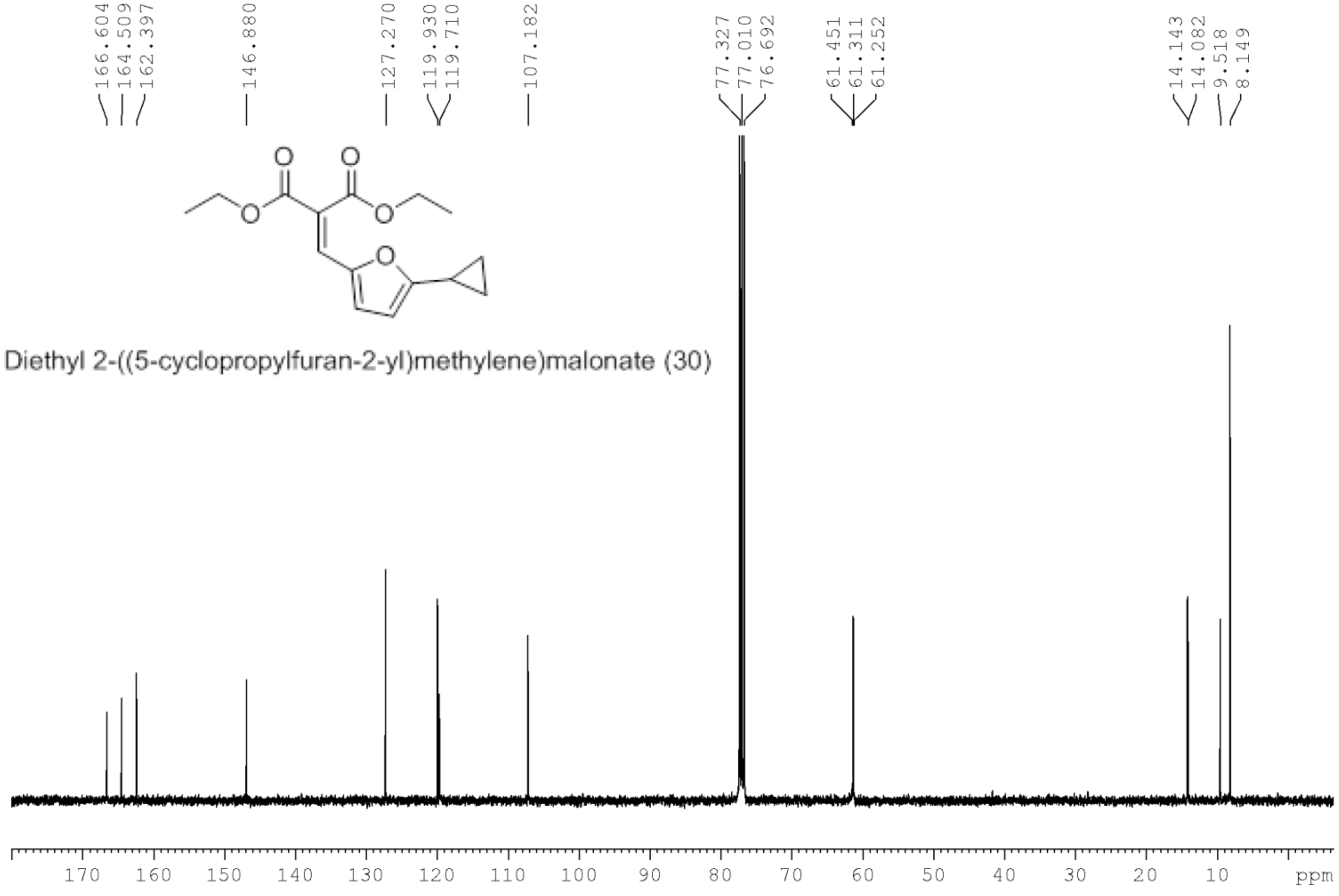

Diethyl 2-((5-cyclopropylfuran-2-yl)methylene)malonate (30)

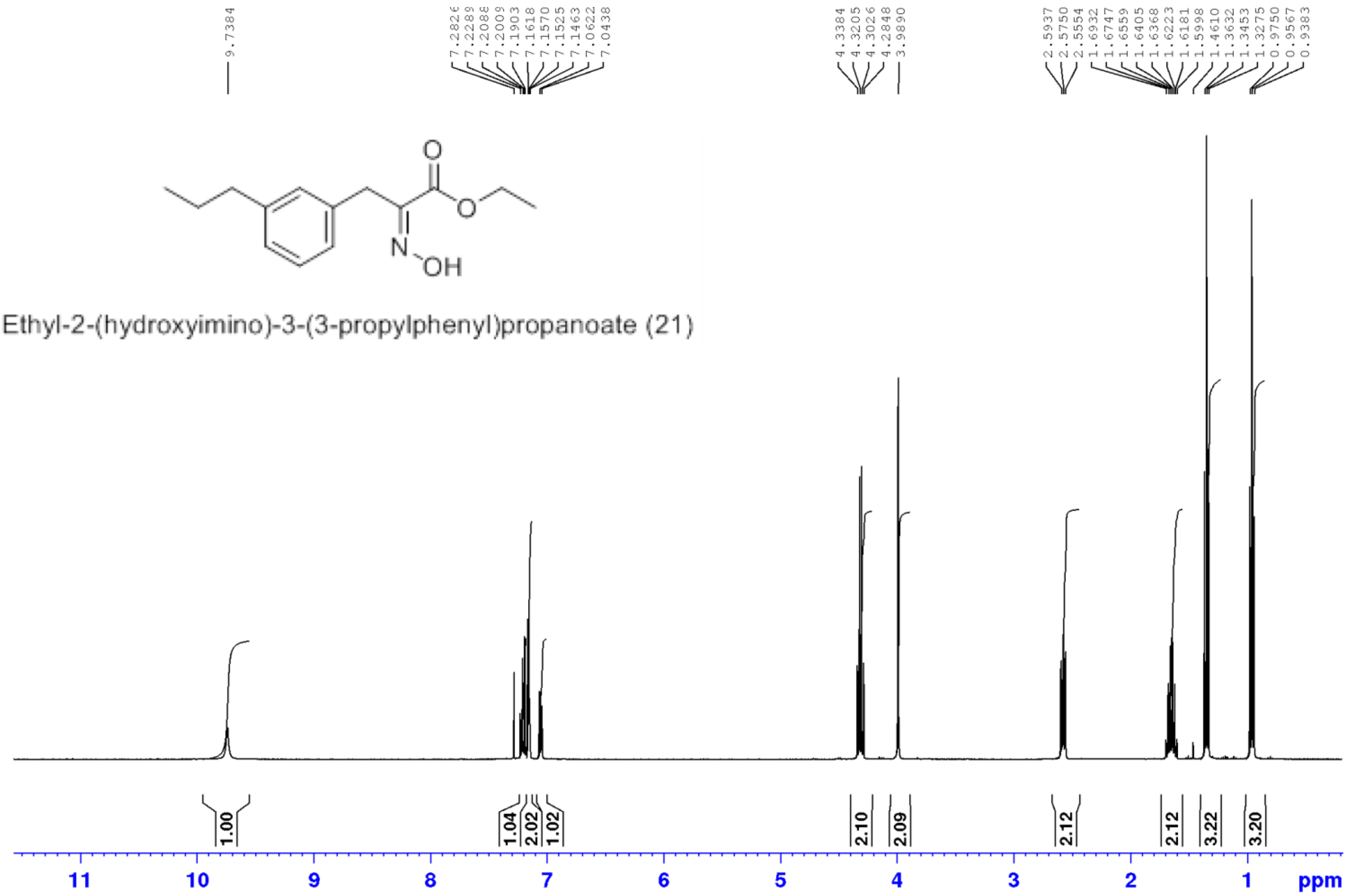
Ethyl-2-(hydroxyimino)-3-(3-propylphenyl)propanoate (21)

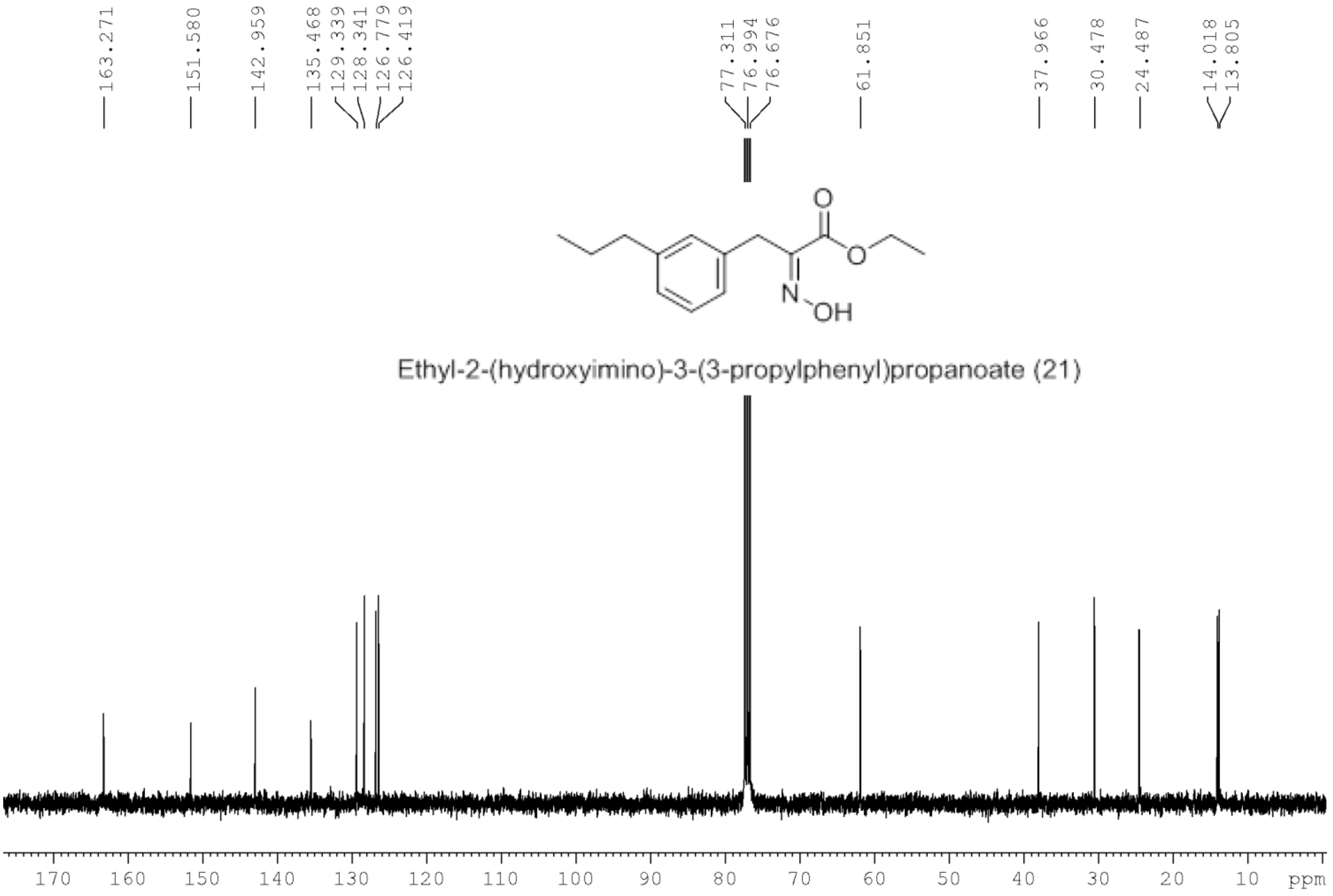
Ethyl-2-(hydroxyimino)-3-(3-propylphenyl)propanoate (21)

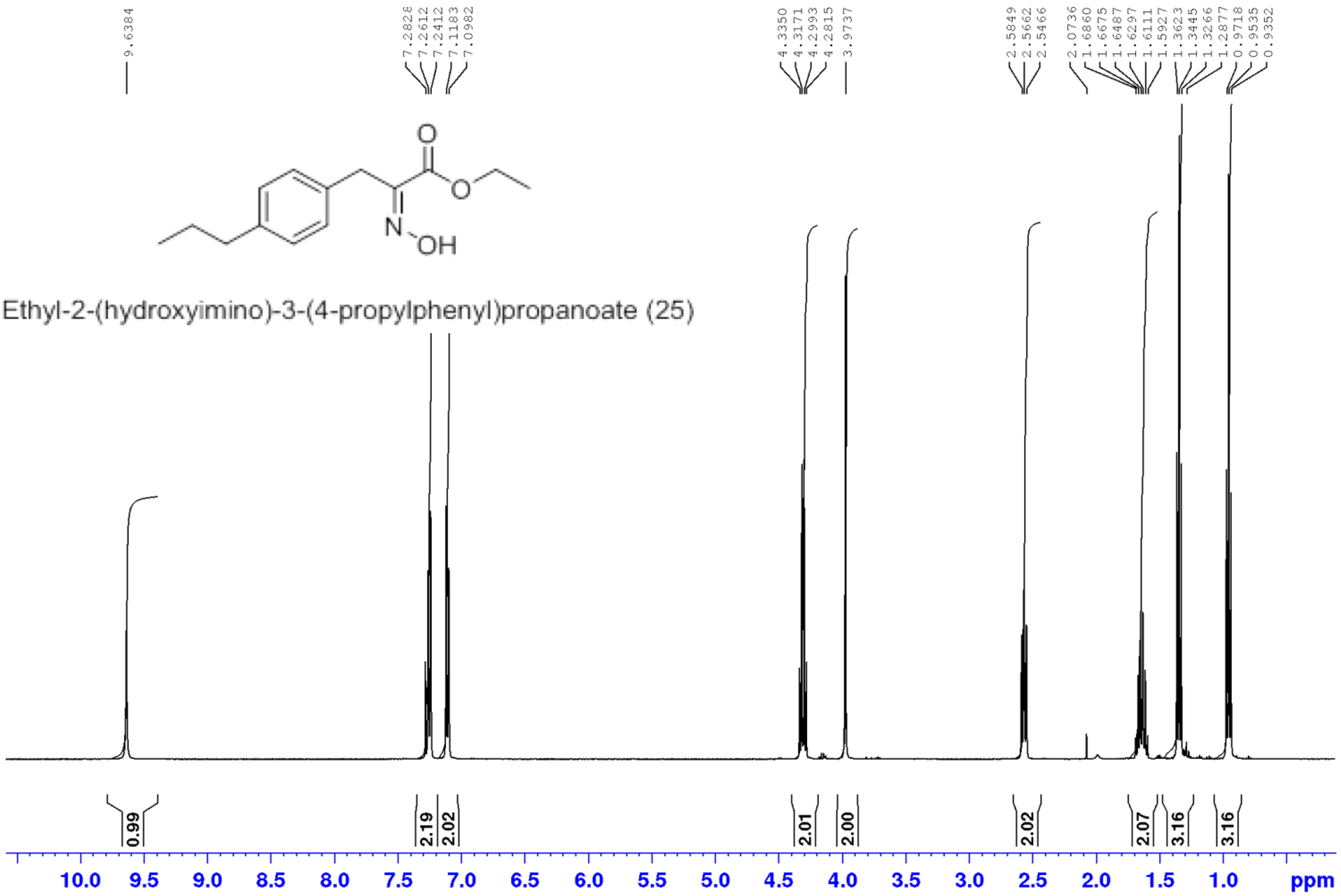
9.6384
7.2826 7.2612 7.2412 7.1183 7.0982
4.3350 4.3171 4.2993 4.2815 3.9737
2.5849 2.5662 2.5466
2.0736 1.6860 1.6675 1.6487 1.6297 1.6111 1.5927 1.3623 1.3445 1.3266 1.2877 0.9718 0.9535 0.9352
Ethyl-2-(hydroxyimino)-3-(4-propylphenyl)propanoate (25)
0.99
2.19
2.02
2.01
2.00
2.02
2.07
3.16
3.16
10.0 9.5 9.0 8.5 8.0 7.5 7.0 6.5 6.0 5.5 5.0 4.5 4.0 3.5 3.0 2.5 2.0 1.5 1.0 ppm

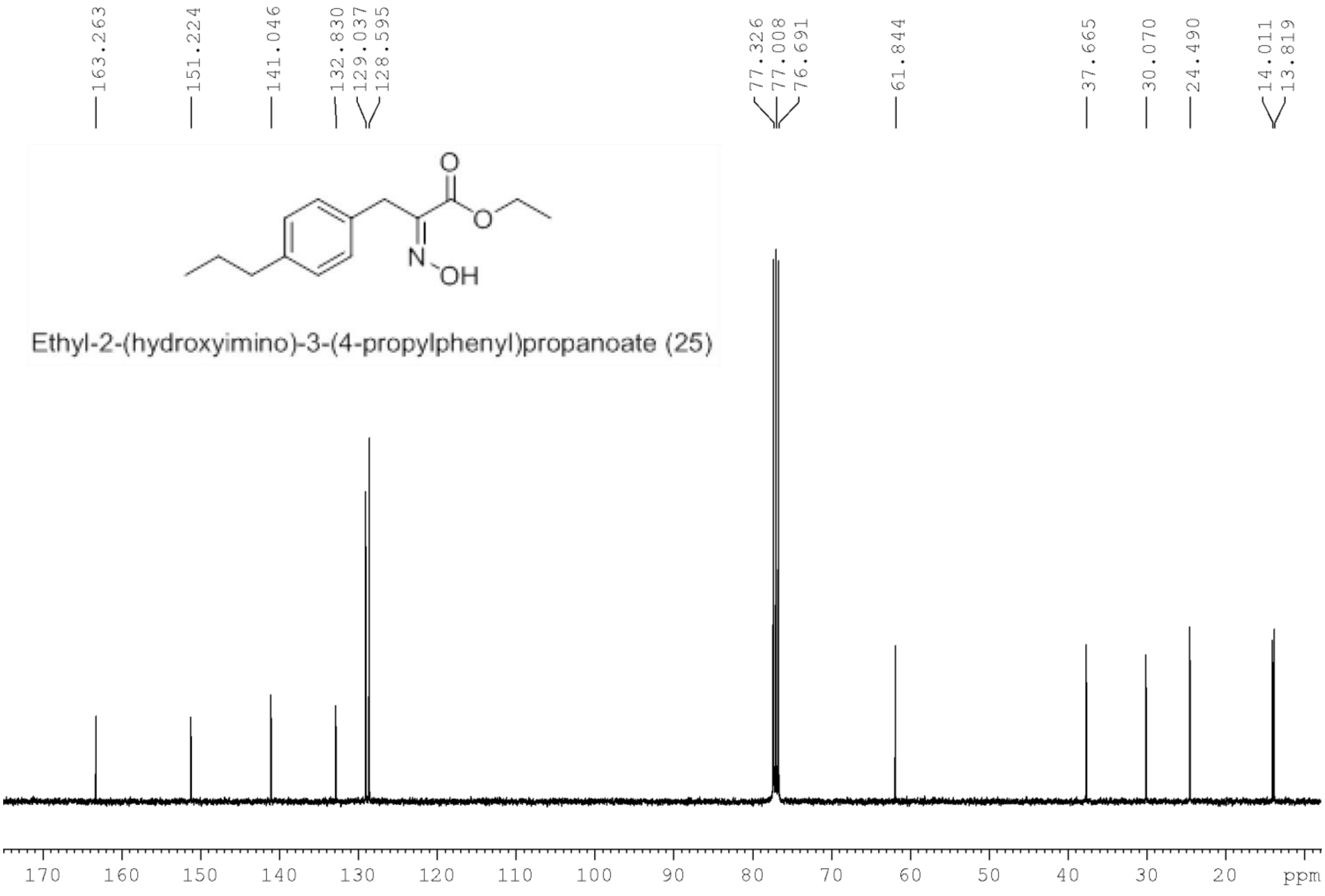
163.263
151.224
141.046
132.830 129.037 128.595
77.326 77.008 76.691
61.844
37.665
30.070
24.490
14.011 13.819
Ethyl-2-(hydroxyimino)-3-(4-propylphenyl)propanoate (25)
170 160 150 140 130 120 110 100 90 80 70 60 50 40 30 20 ppm

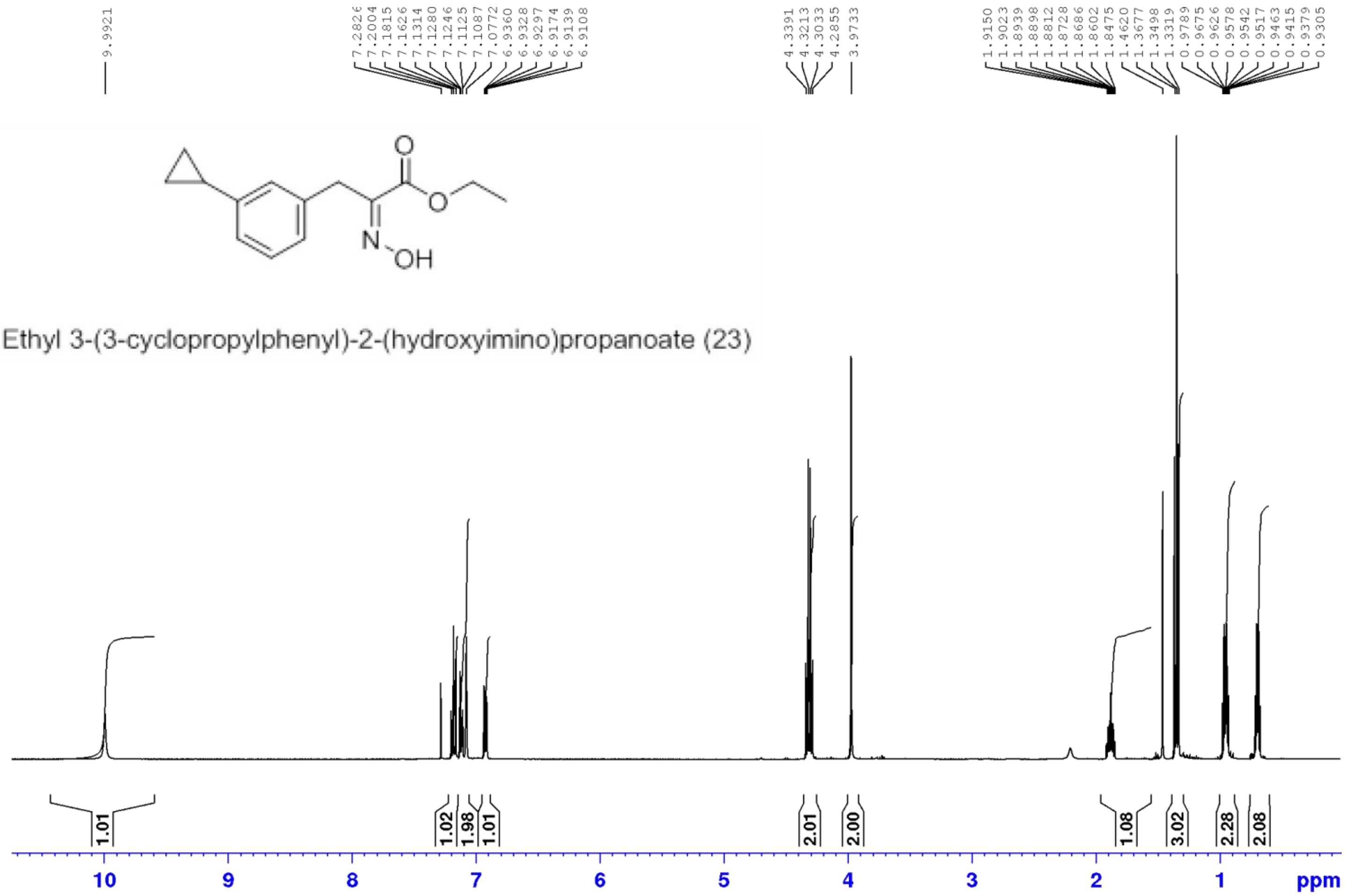
9.9921
7.2826
7.2004
7.1815
7.1626
7.1314
7.1280
7.1246
7.1125
7.1087
7.0772
6.9360
6.9328
6.9297
6.9174
6.9139
6.9108
4.3391
4.3213
4.3033
4.2855
3.9733
1.9150
1.9023
1.8939
1.8898
1.8812
1.8728
1.8686
1.8602
1.8475
1.4620
1.3677
1.3498
1.3319
0.9789
0.9675
0.9626
0.9578
0.9542
0.9517
0.9463
0.9415
0.9379
0.9305
Ethyl 3-(3-cyclopropylphenyl)-2-(hydroxyimino)propanoate (23)
1.01
1.02
1.98
1.01
2.01
2.00
1.08
3.02
2.28
2.08
10
9
8
7
6
5
4
3
2
1
ppm

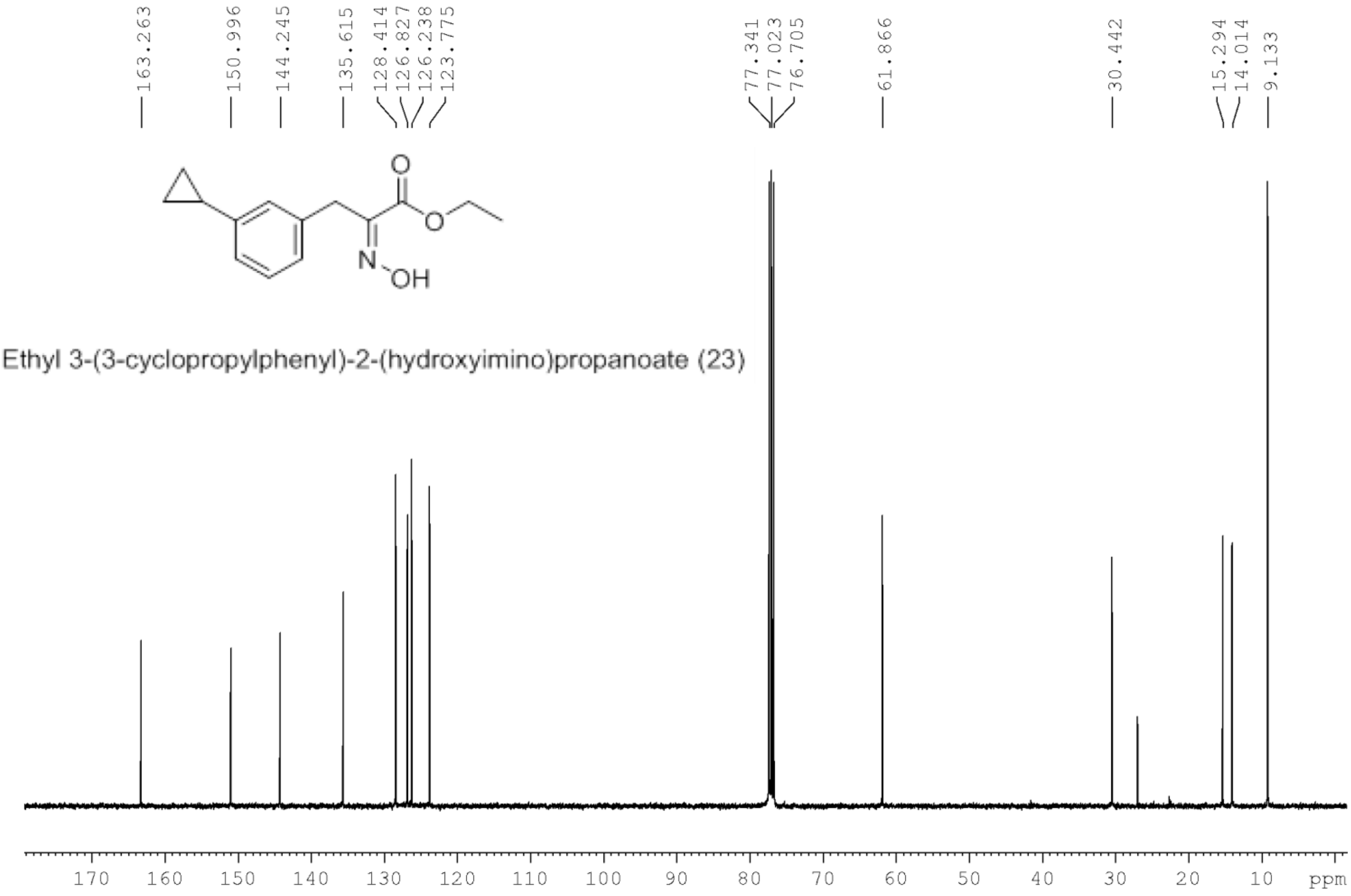
163.263
150.996
144.245
135.615
128.414
126.827
126.238
123.775
77.341
77.023
76.705
61.866
30.442
15.294
14.014
9.133
Ethyl 3-(3-cyclopropylphenyl)-2-(hydroxyimino)propanoate (23)
170
160
150
140
130
120
110
100
90
80
70
60
50
40
30
20
10
ppm

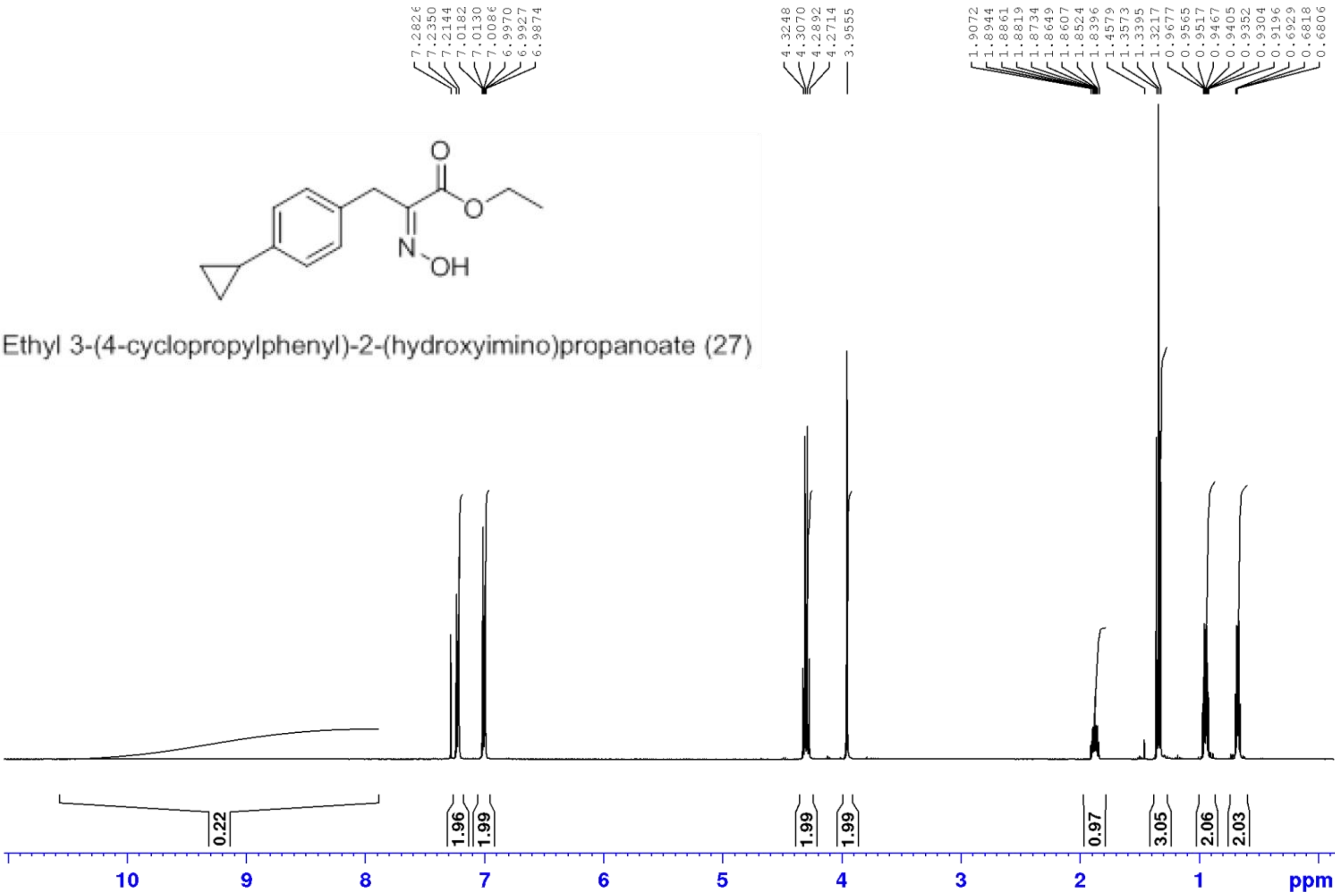
7.2826
7.2350
7.2144
7.0182
7.0130
7.0086
6.9970
6.9927
6.9874
4.3248
4.3070
4.2892
4.2714
3.9555
1.9072
1.8944
1.8861
1.8819
1.8734
1.8649
1.8607
1.8524
1.8396
1.4579
1.3573
1.3395
1.3217
0.9677
0.9565
0.9517
0.9467
0.9405
0.9352
0.9304
0.9196
0.6929
0.6818
0.6806
O
O
N
OH
Ethyl 3-(4-cyclopropylphenyl)-2-(hydroxyimino)propanoate (27)
0.22
1.96
1.99
1.99
1.99
0.97
3.05
2.06
2.03
10
9
8
7
6
5
4
3
2
1
ppm

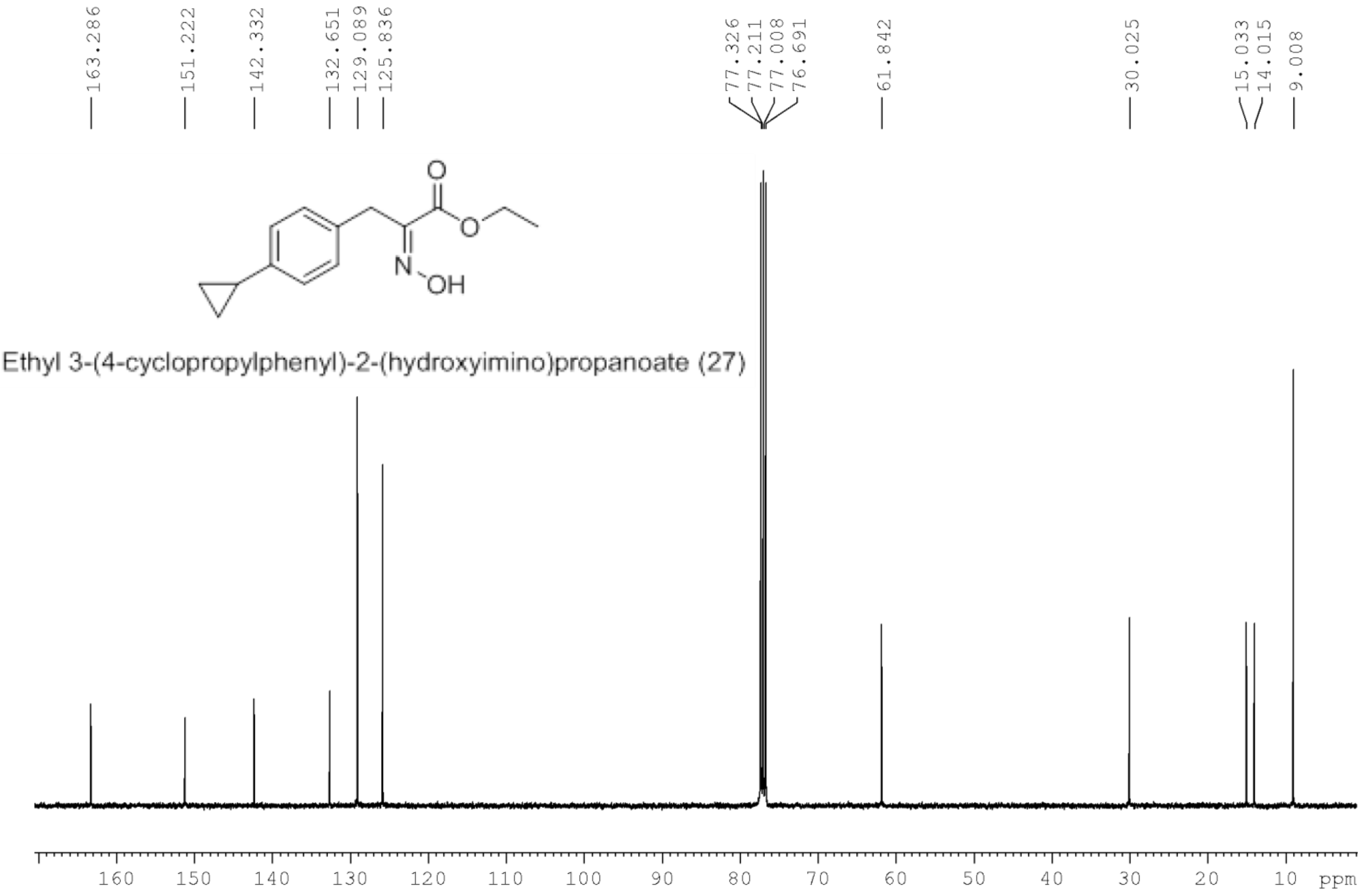
163.286
151.222
142.332
132.651
129.089
125.836
77.326
77.211
77.008
76.691
61.842
30.025
15.033
14.015
9.008
O
O
N
OH
Ethyl 3-(4-cyclopropylphenyl)-2-(hydroxyimino)propanoate (27)
160
150
140
130
120
110
100
90
80
70
60
50
40
30
20
10
ppm

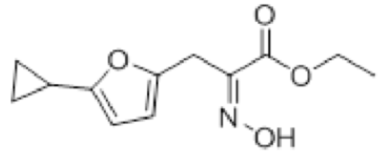

Ethyl 3-(5-cyclopropylfuran-2-yl)-2-(hydroxyimino)propanoate (32)

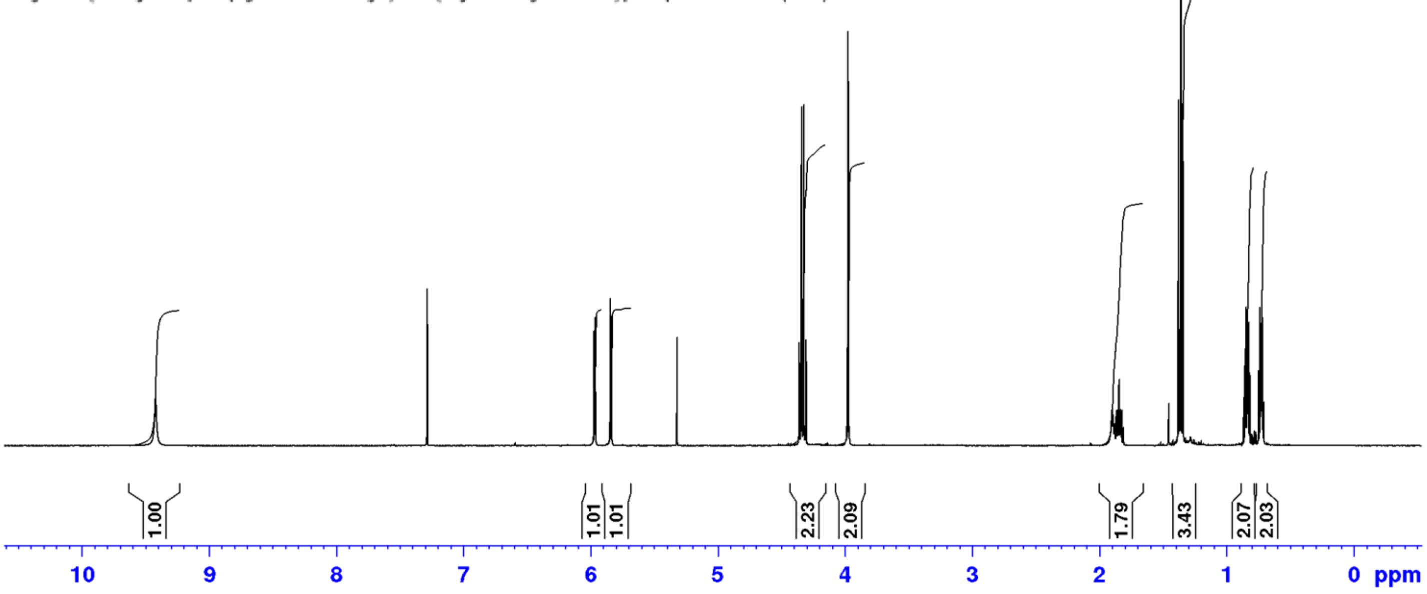

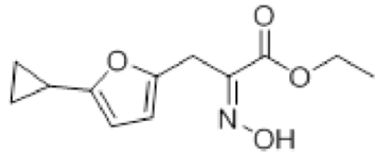

Ethyl 3-(5-cyclopropylfuran-2-yl)-2-(hydroxyimino)propanoate (32)

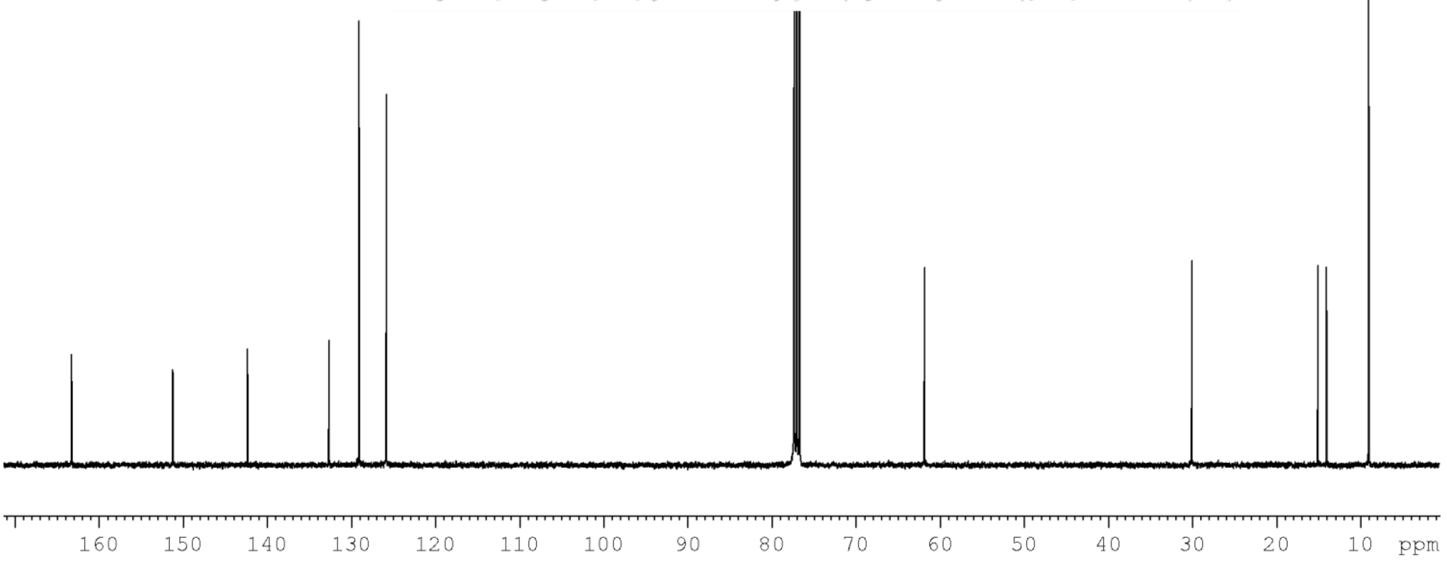

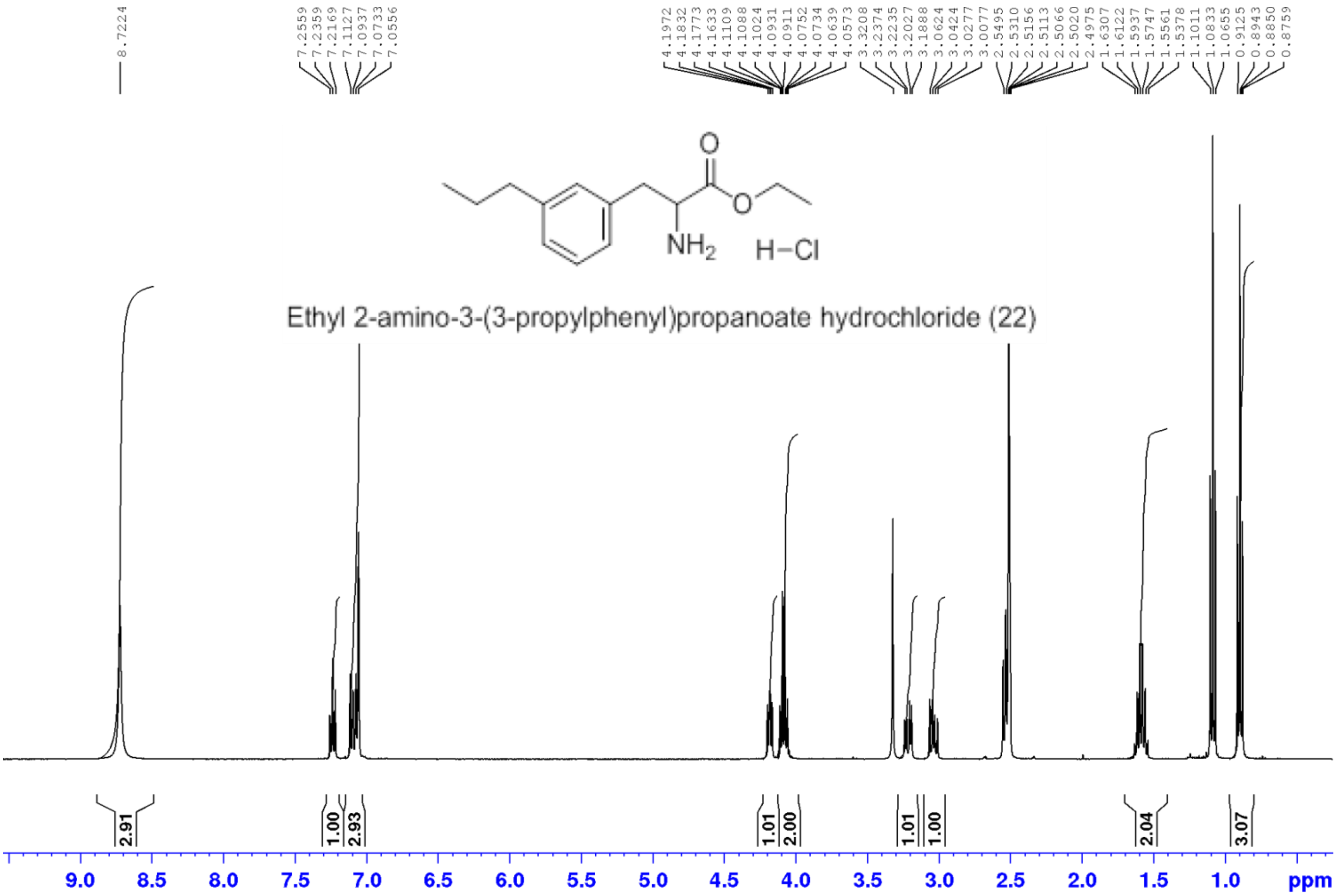
8.7224
7.2559
7.2359
7.2169
7.1127
7.0937
7.0733
7.0556
4.1972
4.1832
4.1773
4.1633
4.1109
4.1088
4.1024
4.0931
4.0911
4.0752
4.0734
4.0639
4.0573
3.3208
3.2374
3.2235
3.2027
3.1888
3.0624
3.0424
3.0277
3.0077
2.5495
2.5310
2.5156
2.5113
2.5066
2.5020
2.4975
1.6307
1.6122
1.5937
1.5747
1.5561
1.5378
1.1011
1.0833
1.0655
0.9125
0.8943
0.8850
0.8759
NH2
H−Cl
Ethyl 2-amino-3-(3-propylphenyl)propanoate hydrochloride (22)
2.91
1.00
2.93
1.01
2.00
1.01
1.00
2.04
3.07
9.0 8.5 8.0 7.5 7.0 6.5 6.0 5.5 5.0 4.5 4.0 3.5 3.0 2.5 2.0 1.5 1.0 ppm

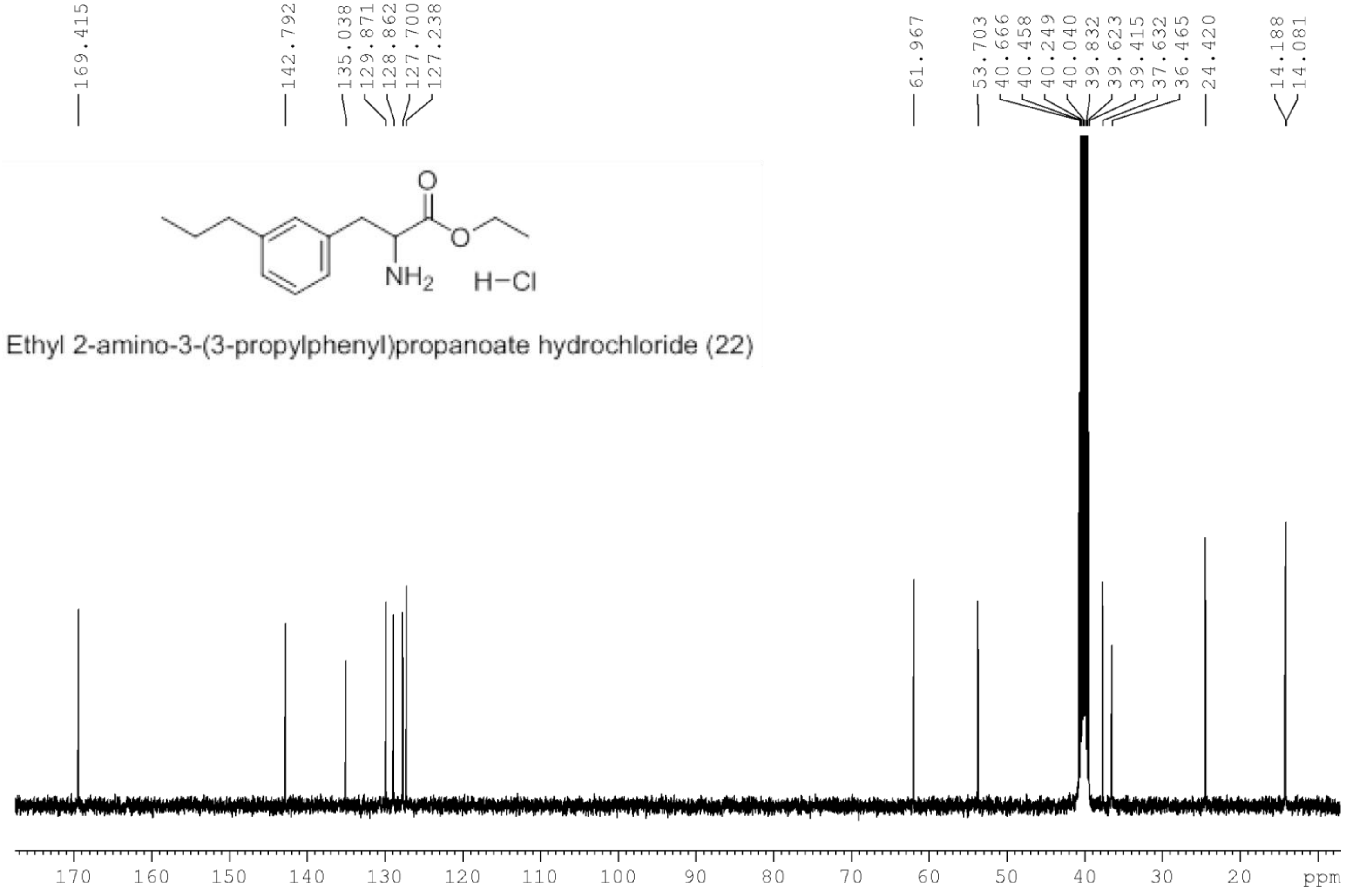
169.415
142.792
135.038
129.871
128.862
127.700
127.238
61.967
53.703
40.666
40.458
40.249
40.040
39.832
39.623
39.415
37.632
36.465
24.420
14.188
14.081
NH2
H−Cl
Ethyl 2-amino-3-(3-propylphenyl)propanoate hydrochloride (22)
170 160 150 140 130 120 110 100 90 80 70 60 50 40 30 20 ppm

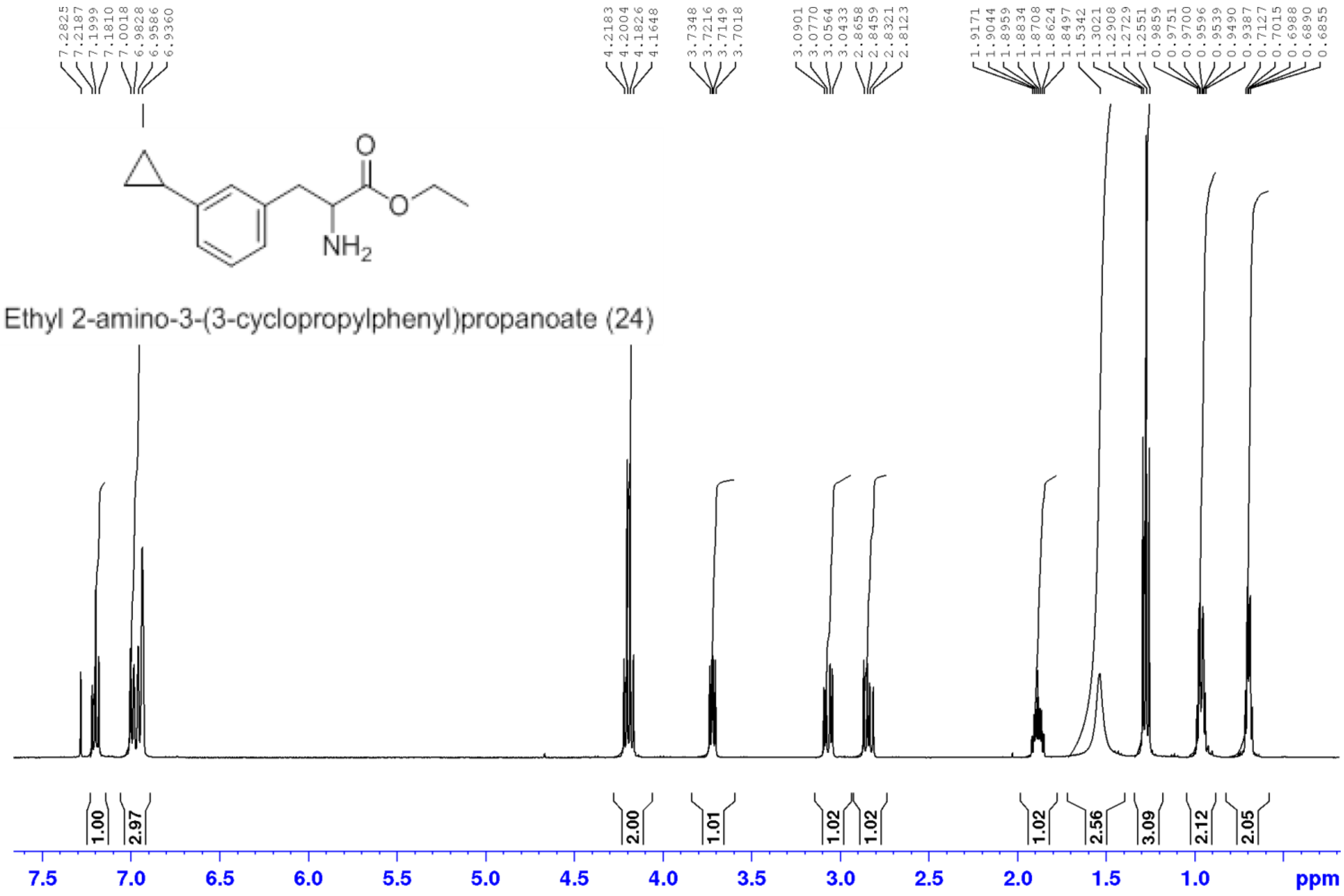
7.2825
7.2187
7.1999
7.1810
7.0018
6.9828
6.9586
6.9360
4.2183
4.2004
4.1826
4.1648
3.7348
3.7216
3.7149
3.7018
3.0901
3.0770
3.0564
3.0433
2.8658
2.8459
2.8321
2.8123
1.9171
1.9044
1.8959
1.8834
1.8708
1.8624
1.8497
1.5342
1.3021
1.2908
1.2729
1.2551
0.9859
0.9751
0.9700
0.9596
0.9539
0.9490
0.9387
0.7127
0.7015
0.6988
0.6890
0.6855
O
O
NH2
Ethyl 2-amino-3-(3-cyclopropylphenyl)propanoate (24)
1.00
2.97
2.00
1.01
1.02
1.02
1.02
2.56
3.09
2.12
2.05
7.5
7.0
6.5
6.0
5.5
5.0
4.5
4.0
3.5
3.0
2.5
2.0
1.5
1.0
ppm

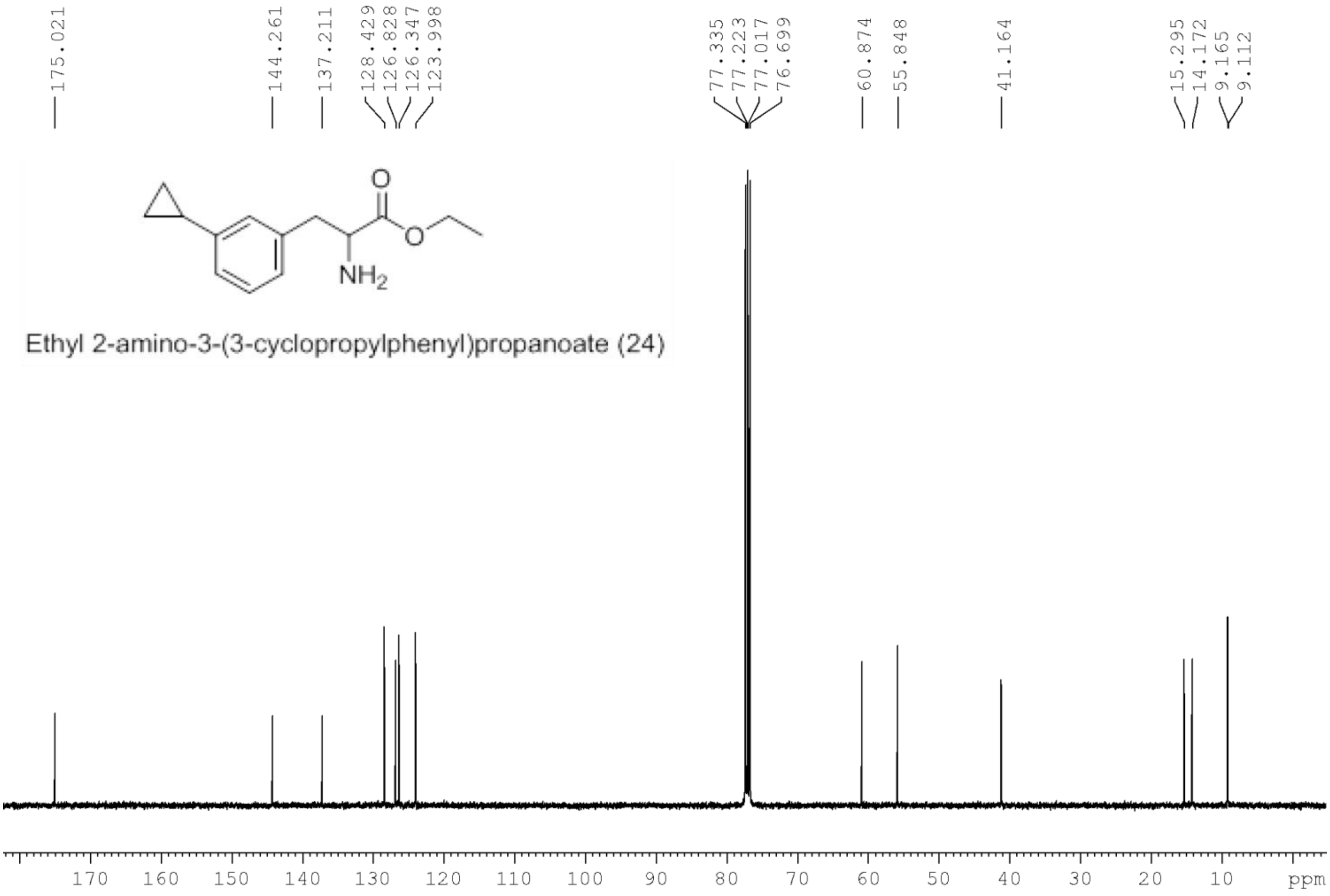
175.021
144.261
137.211
128.429
126.828
126.347
123.998
77.335
77.223
77.017
76.699
60.874
55.848
41.164
15.295
14.172
9.165
9.112
O
O
NH2
Ethyl 2-amino-3-(3-cyclopropylphenyl)propanoate (24)
170
160
150
140
130
120
110
100
90
80
70
60
50
40
30
20
10
ppm

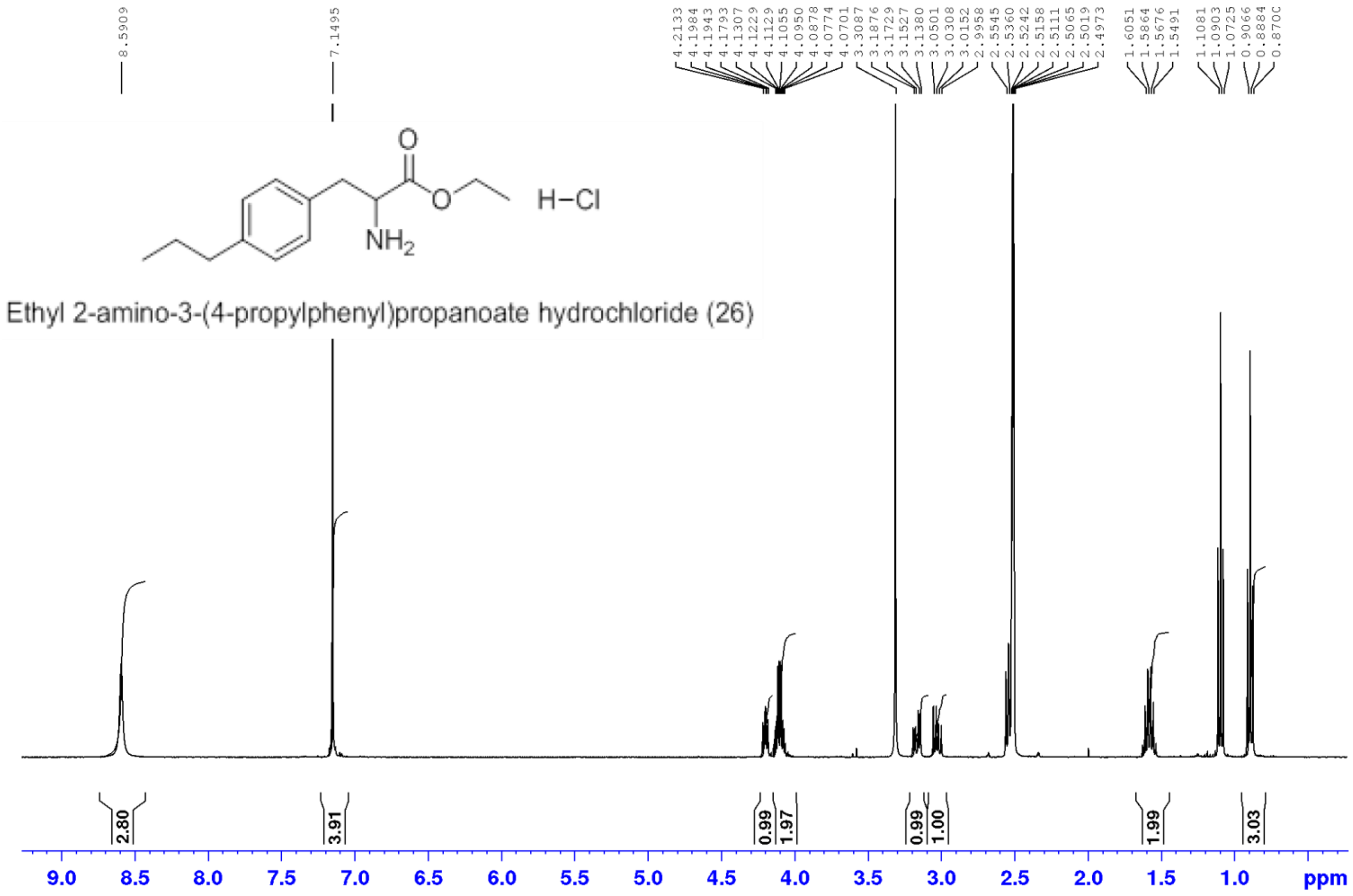
8.5909
7.1495
4.2133
4.1984
4.1943
4.1793
4.1307
4.1229
4.1129
4.1055
4.0950
4.0878
4.0774
4.0701
3.3087
3.1876
3.1729
3.1527
3.1380
3.0501
3.0308
3.0152
2.9958
2.5545
2.5360
2.5242
2.5158
2.5111
2.5065
2.5019
2.4973
1.6051
1.5864
1.5676
1.5491
1.1081
1.0903
1.0725
0.9066
0.8884
0.8700
O
O
NH2
H−Cl
Ethyl 2-amino-3-(4-propylphenyl)propanoate hydrochloride (26)
2.80
3.91
0.99
1.97
0.99
1.00
1.99
3.03
9.0
8.5
8.0
7.5
7.0
6.5
6.0
5.5
5.0
4.5
4.0
3.5
3.0
2.5
2.0
1.5
1.0
ppm

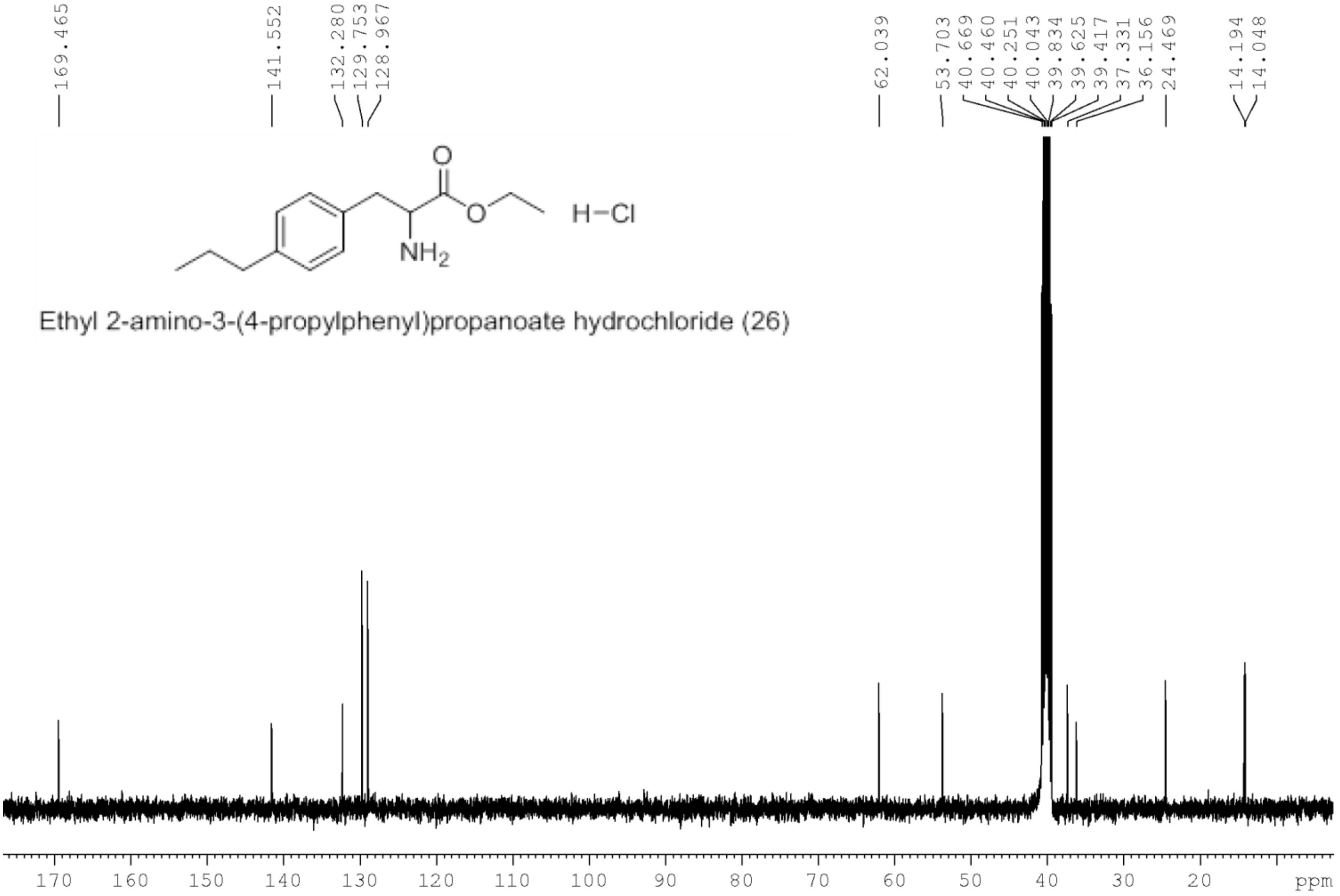
169.465
141.552
132.280
129.753
128.967
62.039
53.703
40.669
40.460
40.251
40.043
39.834
39.625
39.417
37.331
36.156
24.469
14.194
14.048
O
O
NH2
H−Cl
Ethyl 2-amino-3-(4-propylphenyl)propanoate hydrochloride (26)
170
160
150
140
130
120
110
100
90
80
70
60
50
40
30
20
ppm

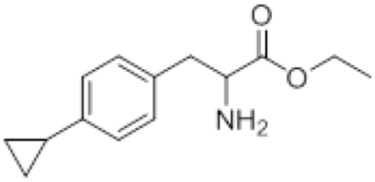

Ethyl 2-amino-3-(4-cyclopropylphenyl)propanoate (28)

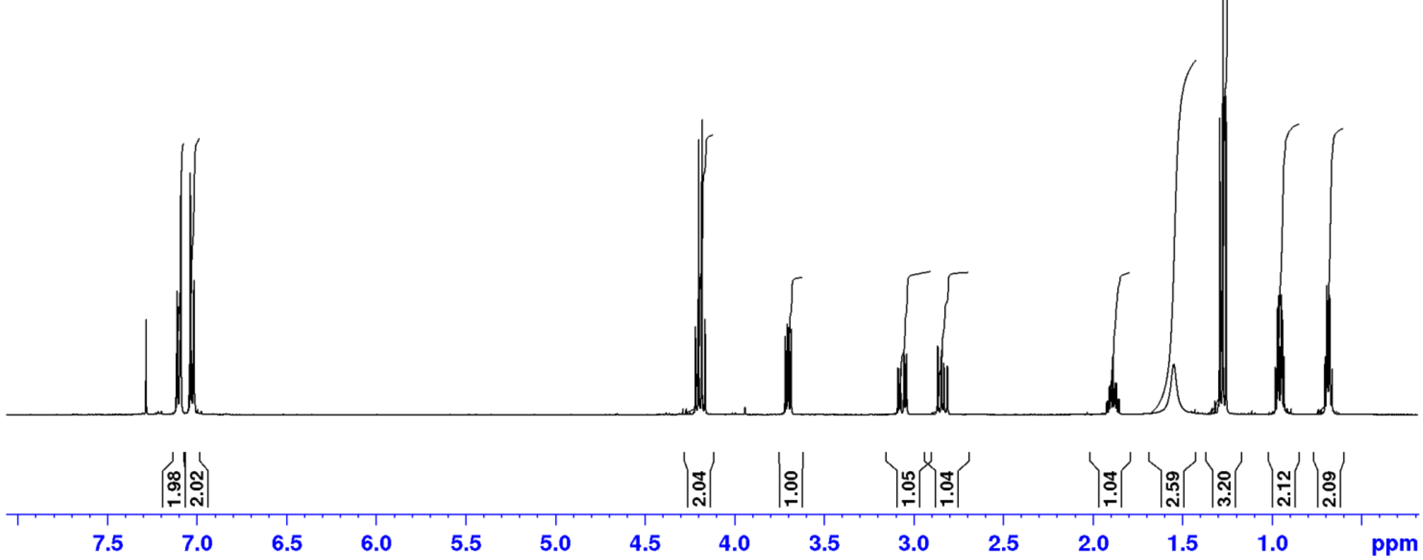

Ethyl 2-amino-3-(4-cyclopropylphenyl)propanoate (28)

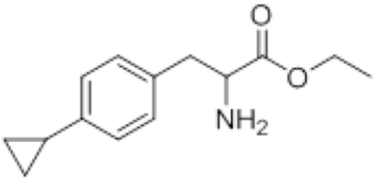

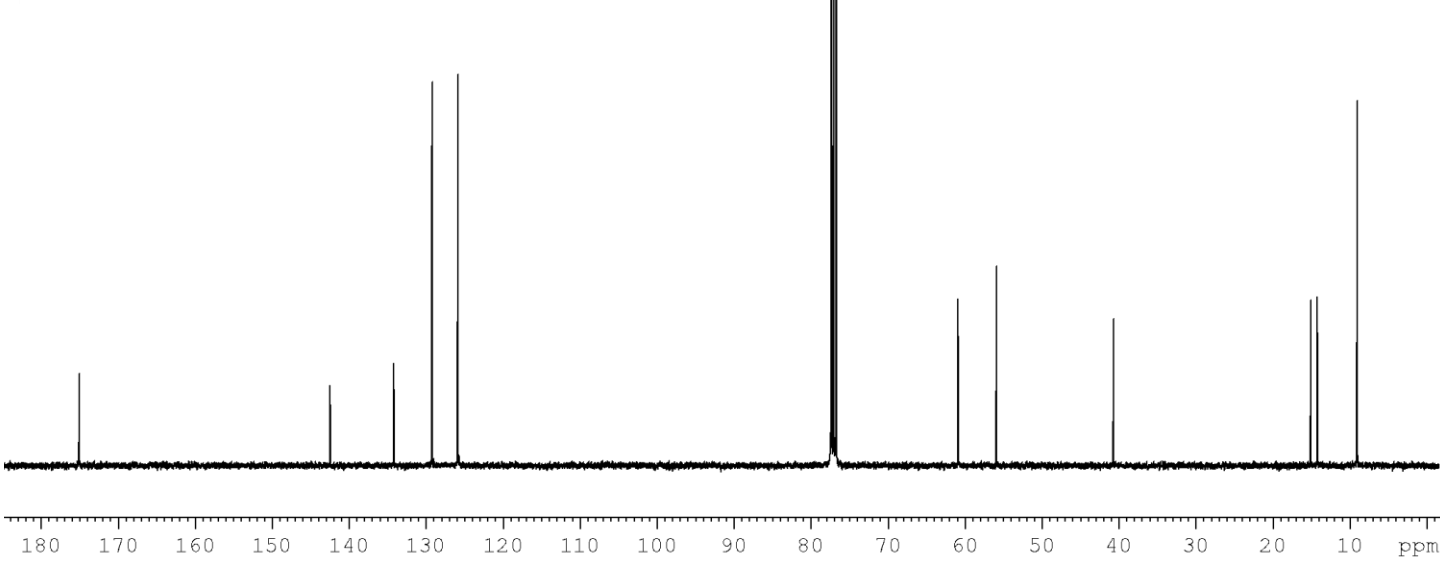

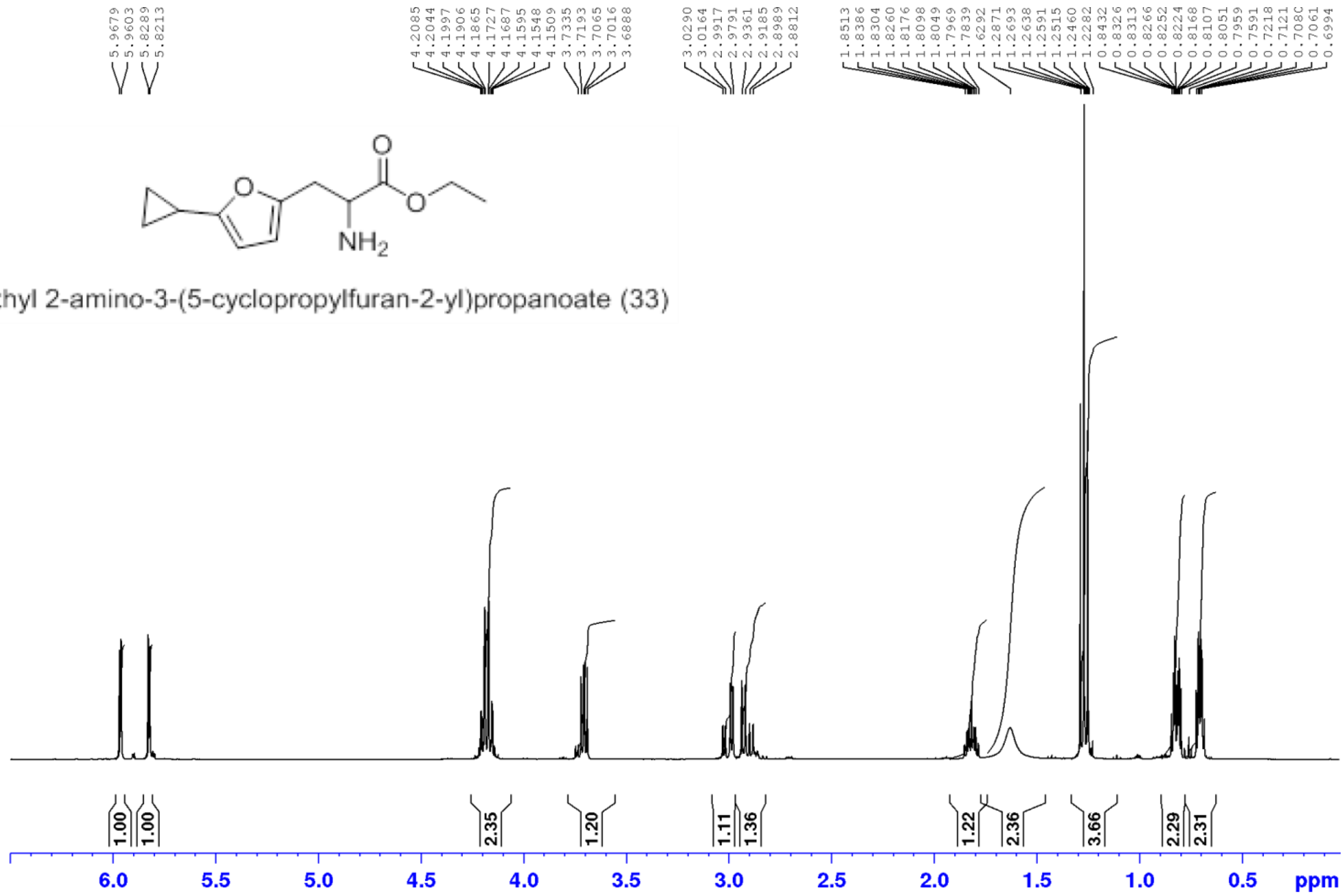

Ethyl 2-amino-3-(5-cyclopropylfuran-2-yl)propanoate (33)

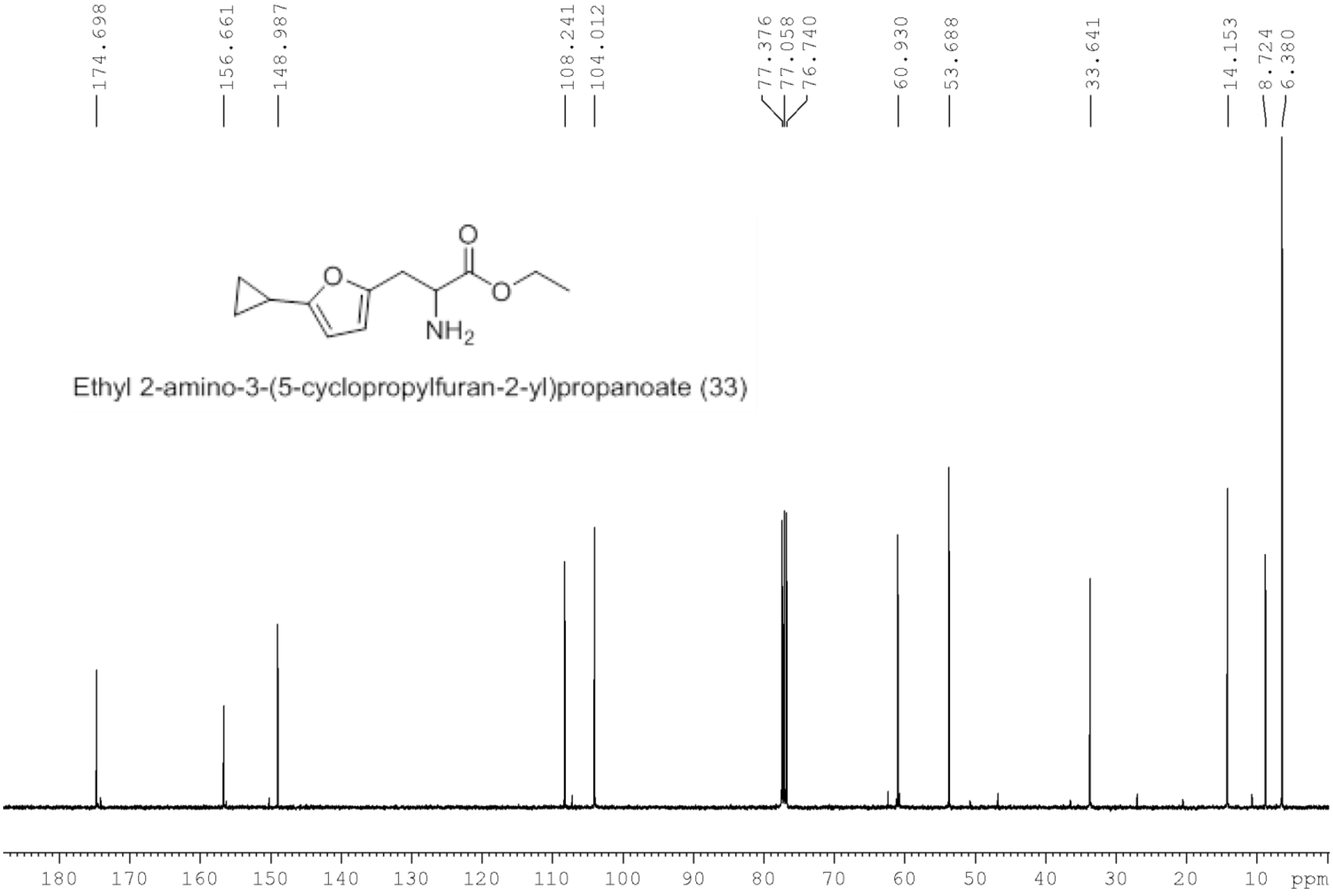

Ethyl 2-amino-3-(5-cyclopropylfuran-2-yl)propanoate (33)

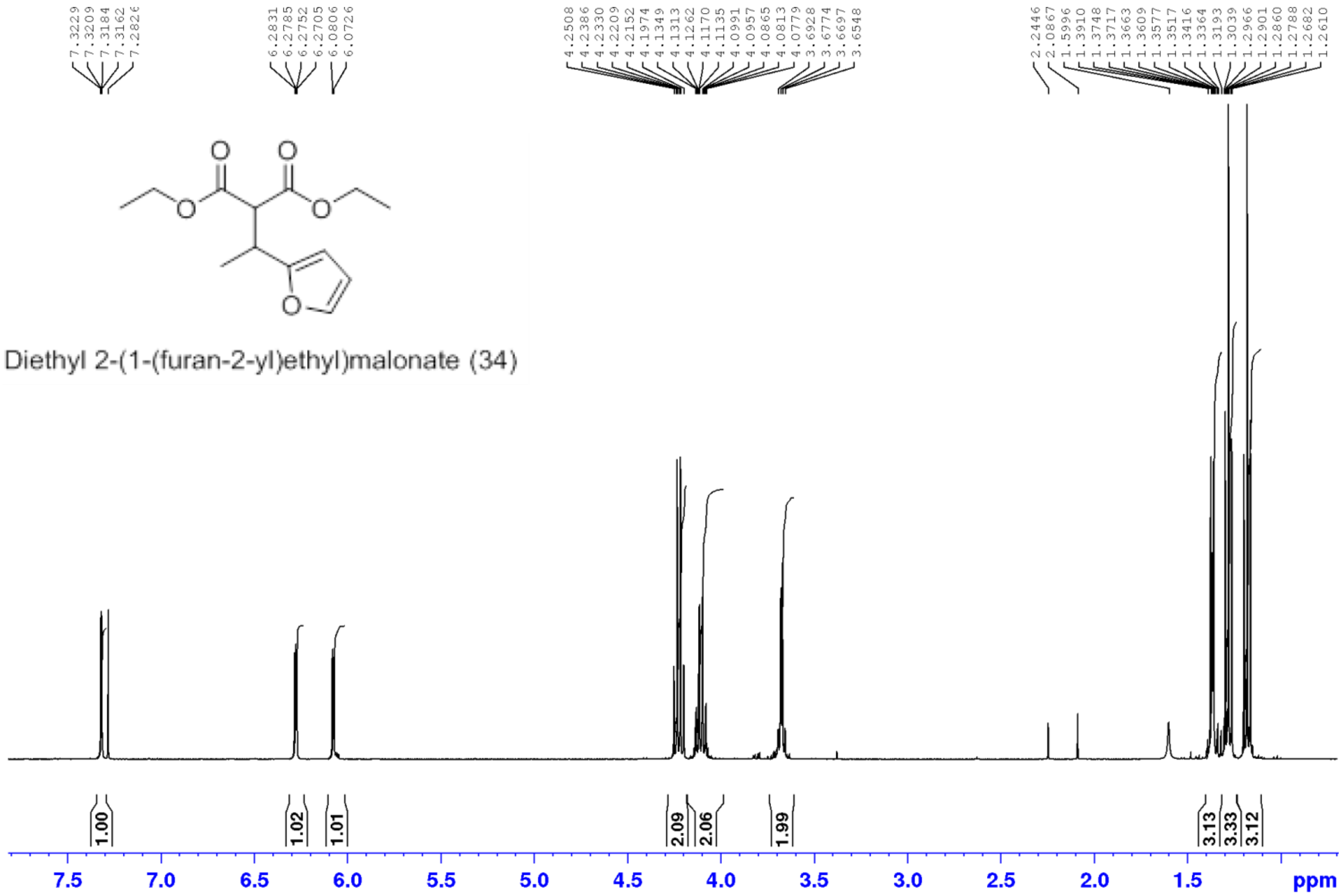
7.3229
7.3209
7.3184
7.3162
7.2826
6.2831
6.2785
6.2752
6.2705
6.0806
6.0726
4.2508
4.2386
4.2330
4.2209
4.2152
4.1974
4.1349
4.1313
4.1262
4.1170
4.1135
4.0991
4.0957
4.0865
4.0813
4.0779
3.6928
3.6774
3.6697
3.6548
2.2446
2.0867
1.5996
1.3910
1.3748
1.3717
1.3663
1.3609
1.3577
1.3517
1.3416
1.3364
1.3193
1.3039
1.2966
1.2901
1.2860
1.2788
1.2682
1.2610
O
O
O
O
O
Diethyl 2-(1-(furan-2-yl)ethyl)malonate (34)
1.00
1.02
1.01
2.09
2.06
1.99
3.13
3.33
3.12
7.5
7.0
6.5
6.0
5.5
5.0
4.5
4.0
3.5
3.0
2.5
2.0
1.5
ppm

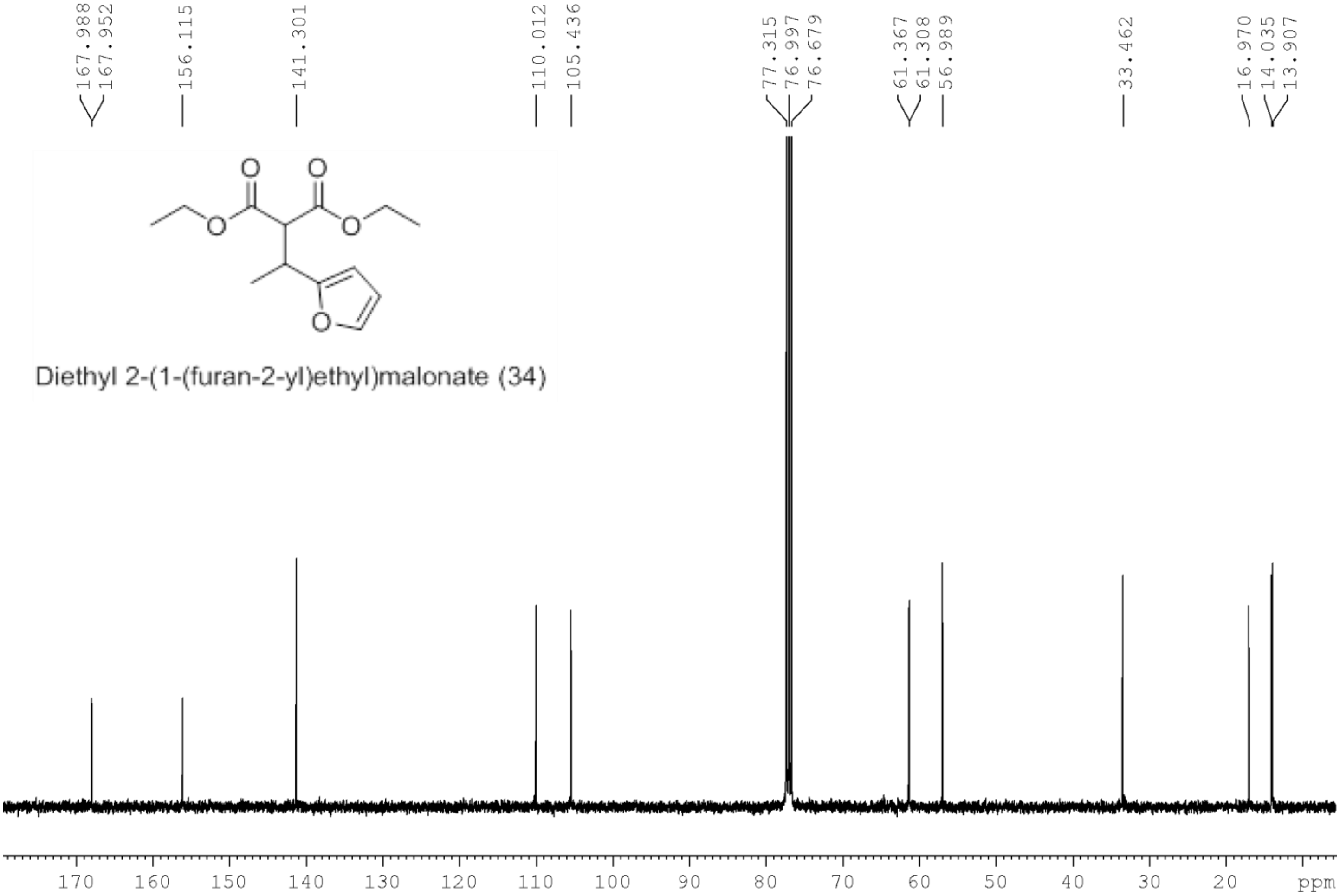
167.988
167.952
156.115
141.301
110.012
105.436
77.315
76.997
76.679
61.367
61.308
56.989
33.462
16.970
14.035
13.907
O
O
O
O
O
Diethyl 2-(1-(furan-2-yl)ethyl)malonate (34)
170
160
150
140
130
120
110
100
90
80
70
60
50
40
30
20
ppm

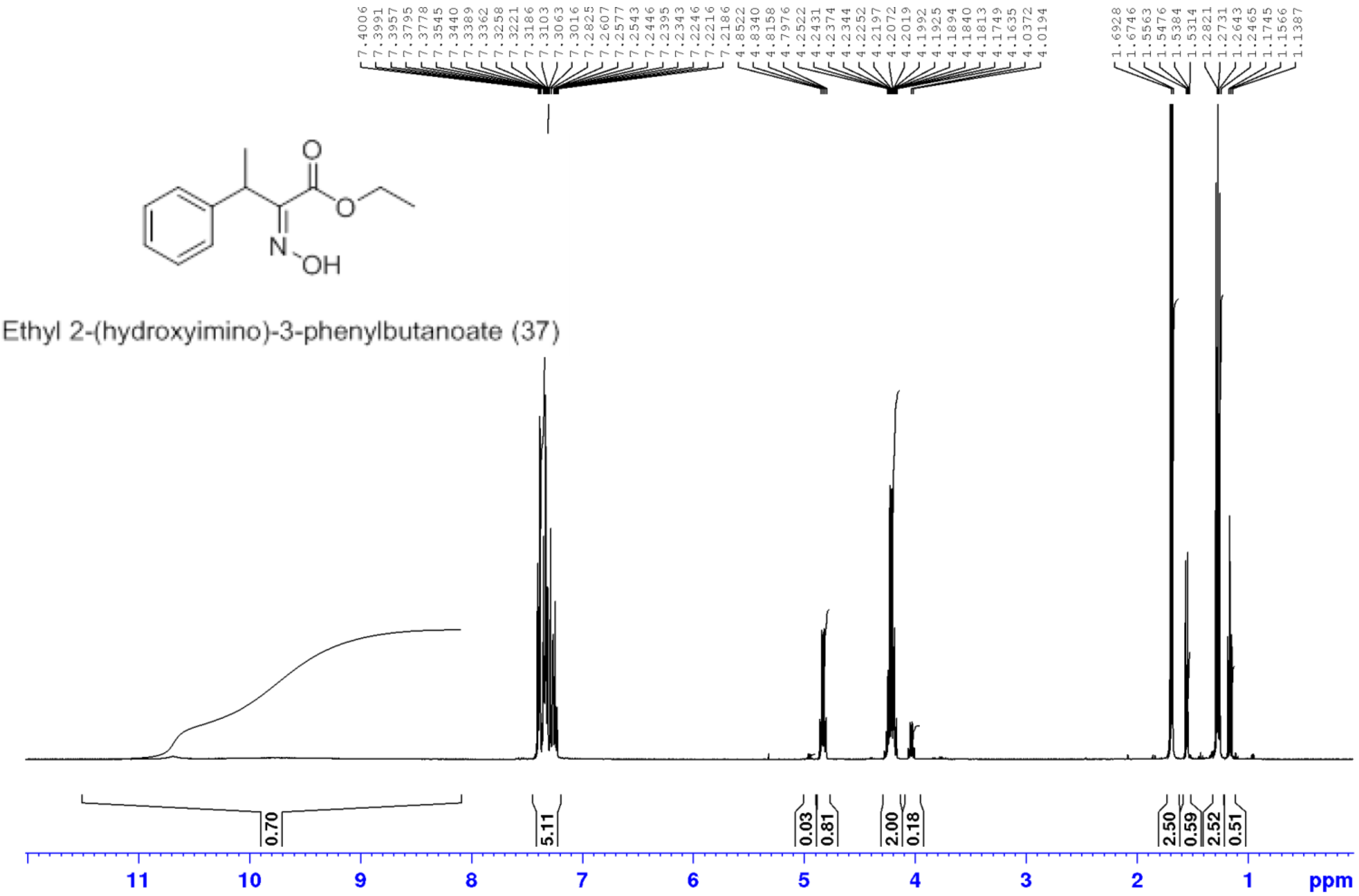

Ethyl 2-(hydroxyimino)-3-phenylbutanoate (37)

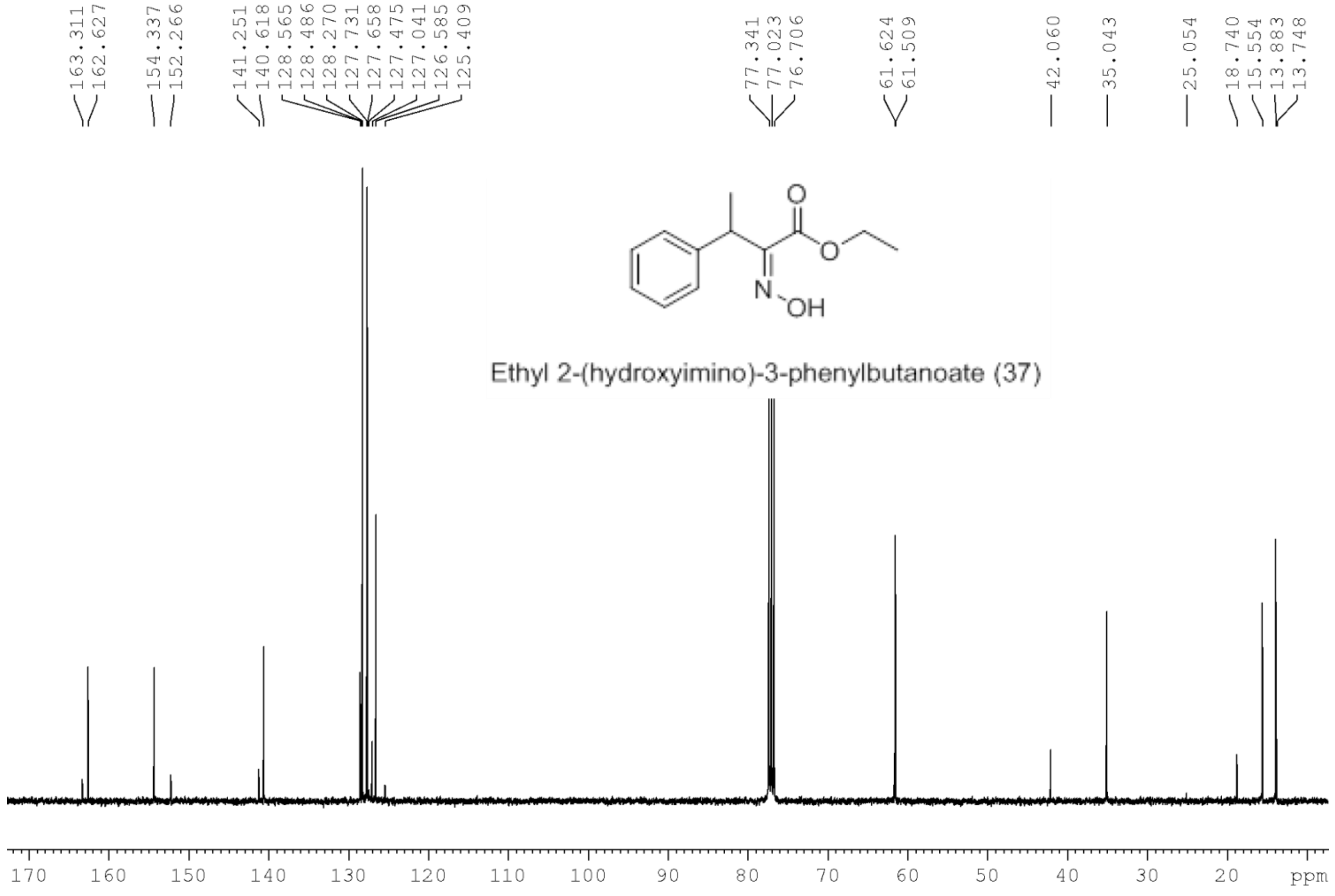

Ethyl 2-(hydroxyimino)-3-phenylbutanoate (37)

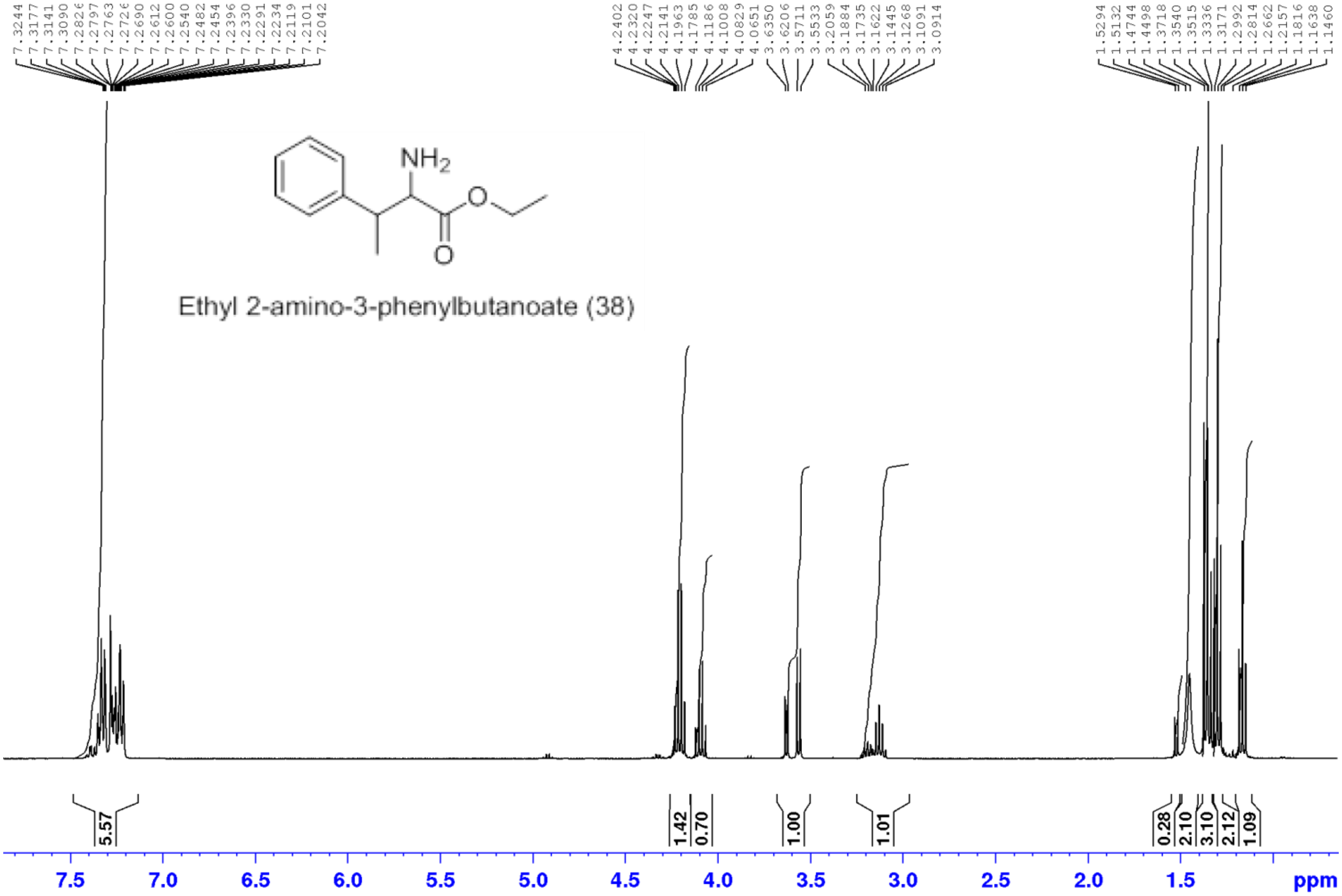
7.3244
7.3177
7.3141
7.3090
7.2826
7.2797
7.2763
7.2726
7.2690
7.2612
7.2600
7.2540
7.2482
7.2454
7.2396
7.2330
7.2291
7.2234
7.2119
7.2101
7.2042
4.2402
4.2320
4.2247
4.2141
4.1963
4.1785
4.1186
4.1008
4.0829
4.0651
3.6350
3.6206
3.5711
3.5533
3.2059
3.1884
3.1735
3.1622
3.1445
3.1268
3.1091
3.0914
1.5294
1.5132
1.4744
1.4498
1.3718
1.3540
1.3515
1.3336
1.3171
1.2992
1.2814
1.2662
1.2157
1.1816
1.1638
1.1460
NH2
O
O
Ethyl 2-amino-3-phenylbutanoate (38)
5.57
1.42
0.70
1.00
1.01
0.28
2.10
3.10
2.12
1.09
7.5
7.0
6.5
6.0
5.5
5.0
4.5
4.0
3.5
3.0
2.5
2.0
1.5
ppm

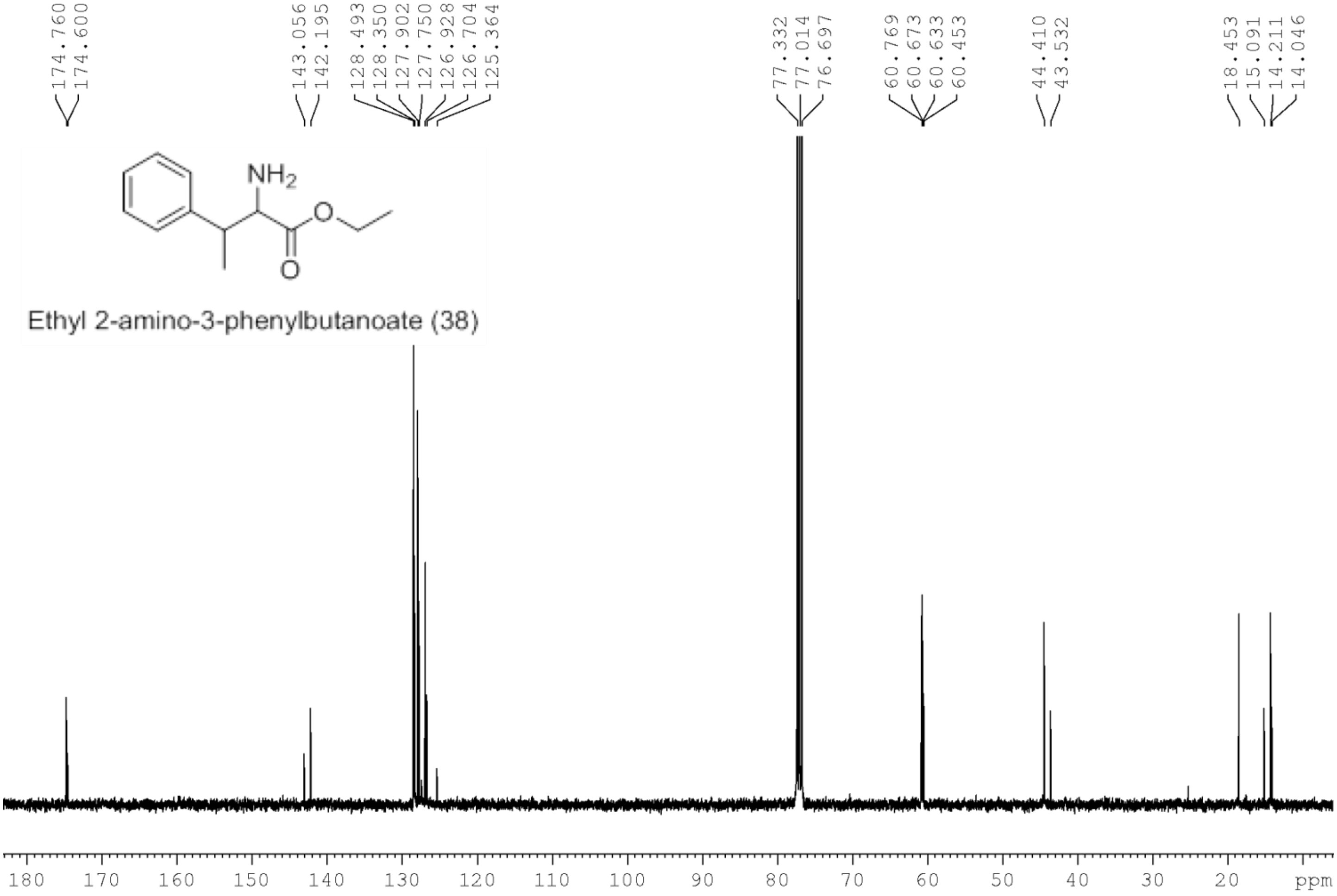
174.760
174.600
143.056
142.195
128.493
128.350
127.902
127.750
126.928
126.704
125.364
77.332
77.014
76.697
60.769
60.673
60.633
60.453
44.410
43.532
18.453
15.091
14.211
14.046
NH2
O
O
Ethyl 2-amino-3-phenylbutanoate (38)
180
170
160
150
140
130
120
110
100
90
80
70
60
50
40
30
20
ppm

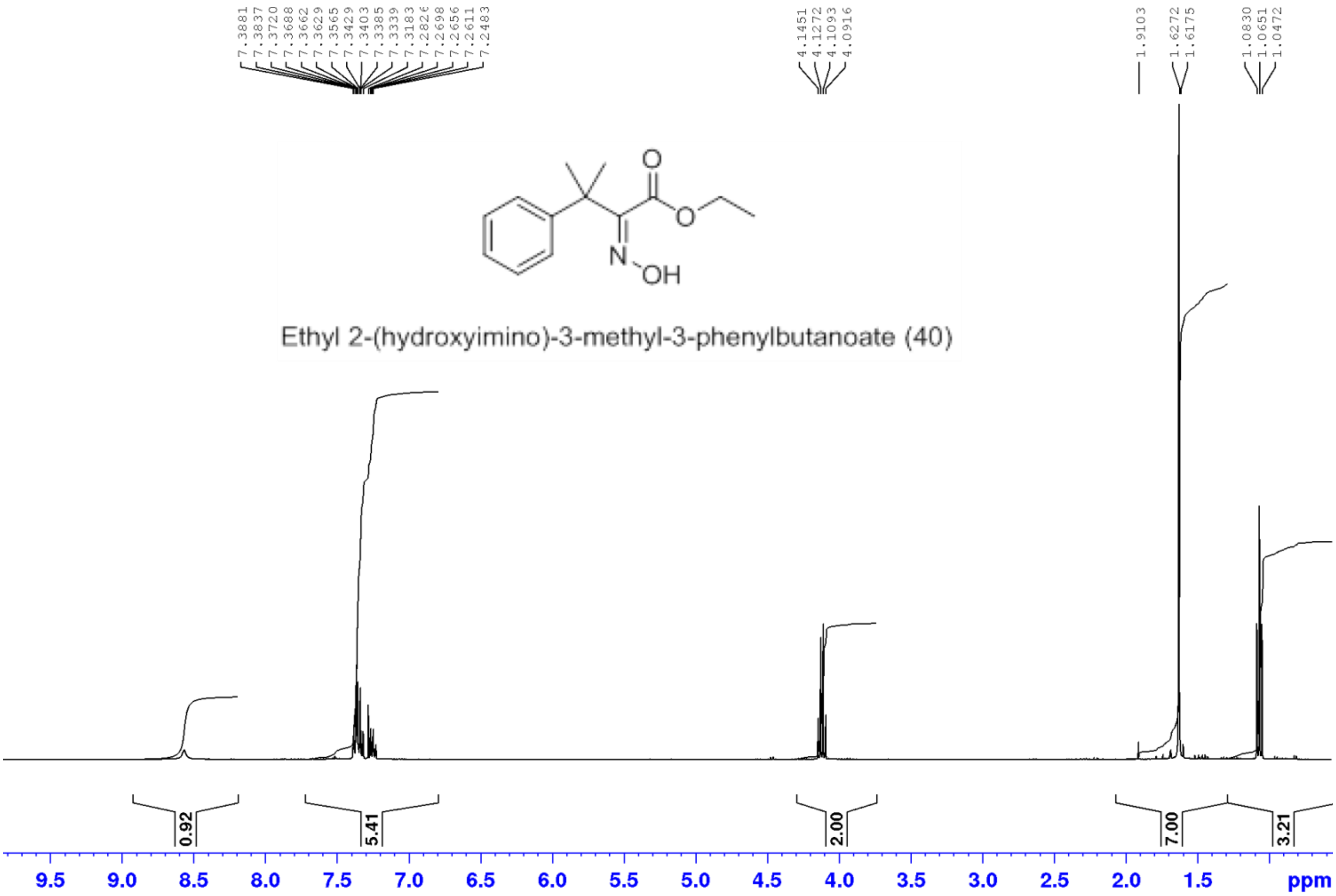
7.3881
7.3837
7.3720
7.3688
7.3662
7.3629
7.3565
7.3429
7.3403
7.3385
7.3339
7.3183
7.2826
7.2698
7.2656
7.2611
7.2483
4.1451
4.1272
4.1093
4.0916
1.9103
1.6272
1.6175
1.0830
1.0651
1.0472
O
O
N
OH
Ethyl 2-(hydroxyimino)-3-methyl-3-phenylbutanoate (40)
0.92
5.41
2.00
7.00
3.21
9.5
9.0
8.5
8.0
7.5
7.0
6.5
6.0
5.5
5.0
4.5
4.0
3.5
3.0
2.5
2.0
1.5
ppm

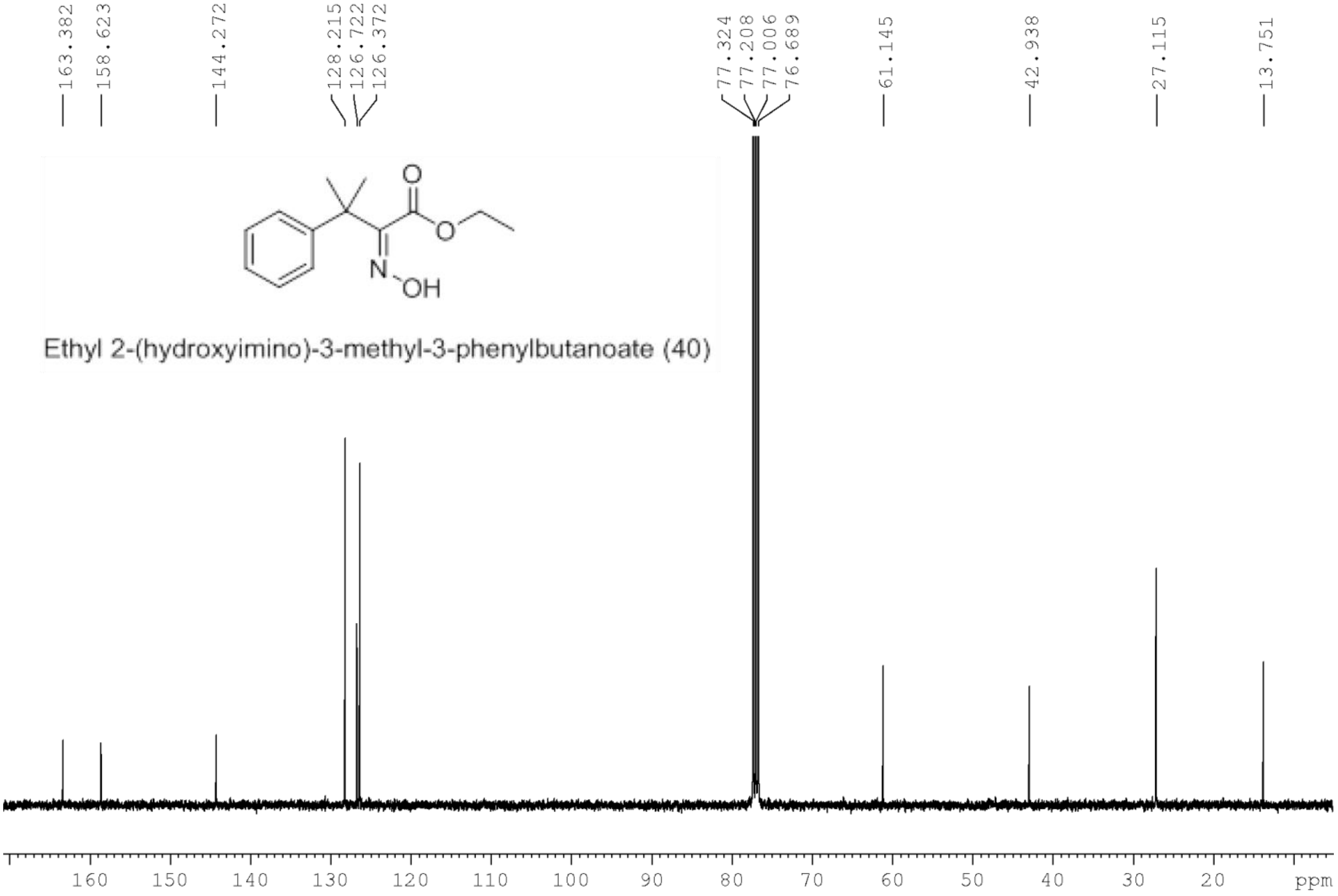
163.382
158.623
144.272
128.215
126.722
126.372
77.324
77.208
77.006
76.689
61.145
42.938
27.115
13.751
O
O
N
OH
Ethyl 2-(hydroxyimino)-3-methyl-3-phenylbutanoate (40)
160
150
140
130
120
110
100
90
80
70
60
50
40
30
20
ppm

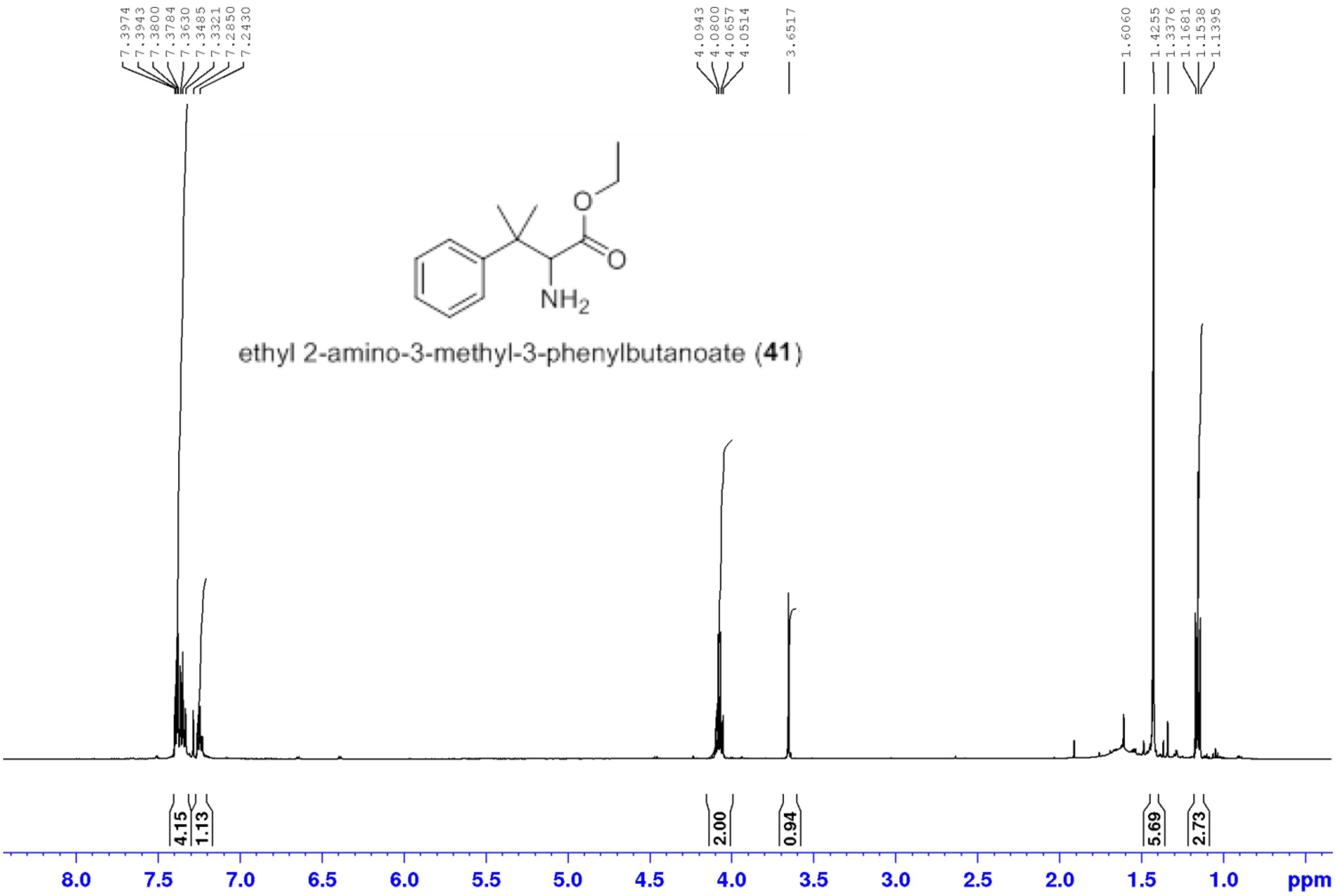
7.3974
7.3943
7.3800
7.3784
7.3630
7.3485
7.3321
7.2850
7.2430
4.0943
4.0800
4.0657
4.0514
3.6517
1.6060
1.4255
1.3376
1.1681
1.1538
1.1395
O
O
NH2
ethyl 2-amino-3-methyl-3-phenylbutanoate (41)
4.15
1.13
2.00
0.94
5.69
2.73
8.0
7.5
7.0
6.5
6.0
5.5
5.0
4.5
4.0
3.5
3.0
2.5
2.0
1.5
1.0
ppm

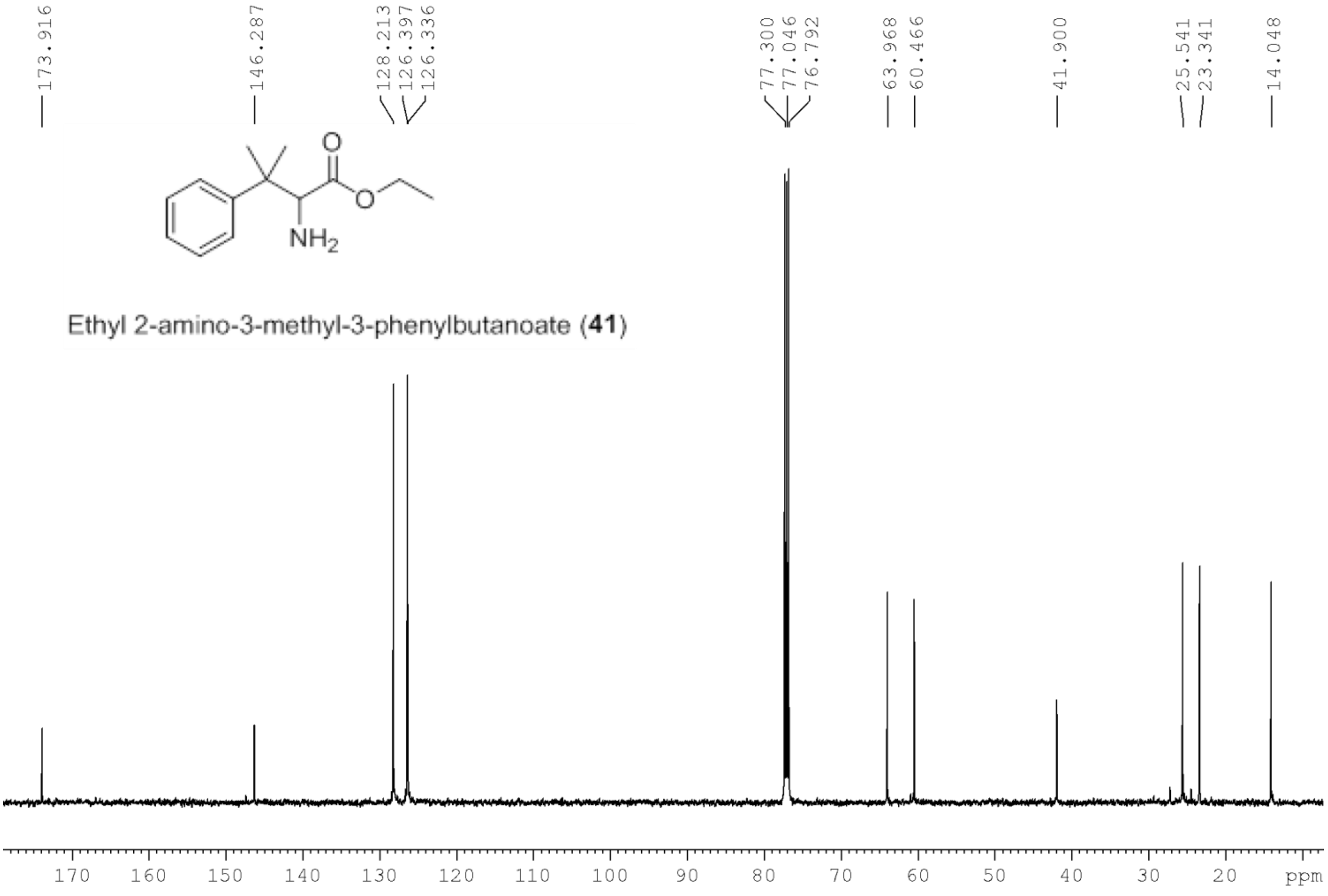
173.916
146.287
128.213
126.397
126.336
77.300
77.046
76.792
63.968
60.466
41.900
25.541
23.341
14.048
O
O
NH2
Ethyl 2-amino-3-methyl-3-phenylbutanoate (41)
170
160
150
140
130
120
110
100
90
80
70
60
50
40
30
20
ppm

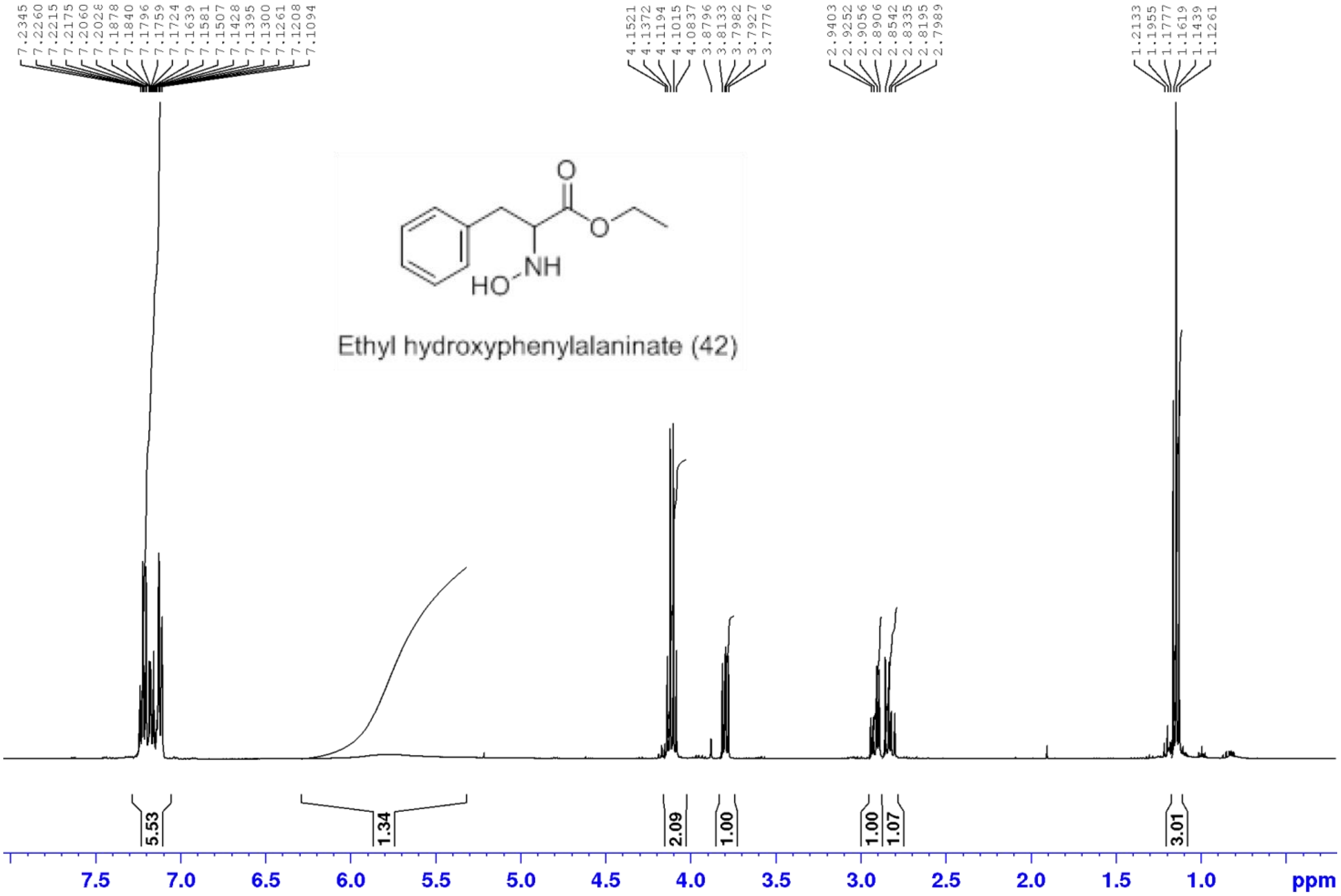
7.2345 7.2260 7.2215 7.2175 7.2026 7.1878 7.1840 7.1796 7.1759 7.1724 7.1639 7.1581 7.1507 7.1428 7.1395 7.1300 7.1261 7.1208 7.1094
4.1521 4.1372 4.1194 4.1015 4.0837 3.8796 3.8133 3.7982 3.7927 3.7776
2.9403 2.9252 2.9056 2.8906 2.8542 2.8335 2.8195 2.7989
1.2133 1.1955 1.1777 1.1619 1.1439 1.1261
HO
NH
O
O
Ethyl hydroxyphenylalaninate (42)
5.53
1.34
2.09
1.00
1.00
1.07
3.01
7.5 7.0 6.5 6.0 5.5 5.0 4.5 4.0 3.5 3.0 2.5 2.0 1.5 1.0 ppm

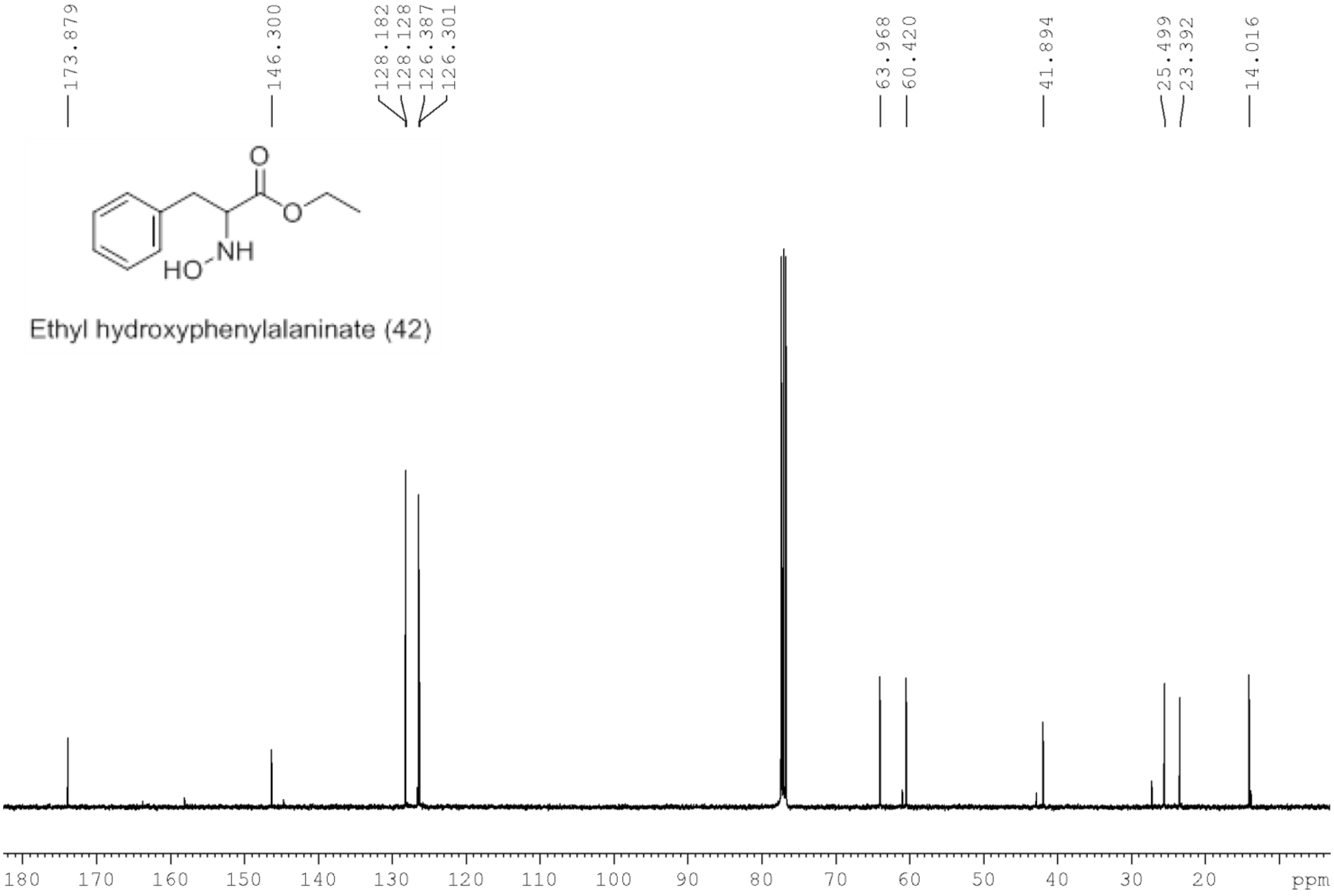
173.879
146.300
128.182 128.128 126.387 126.301
63.968 60.420
41.894
25.499 23.392
14.016
HO
NH
O
O
Ethyl hydroxyphenylalaninate (42)
180 170 160 150 140 130 120 110 100 90 80 70 60 50 40 30 20 ppm

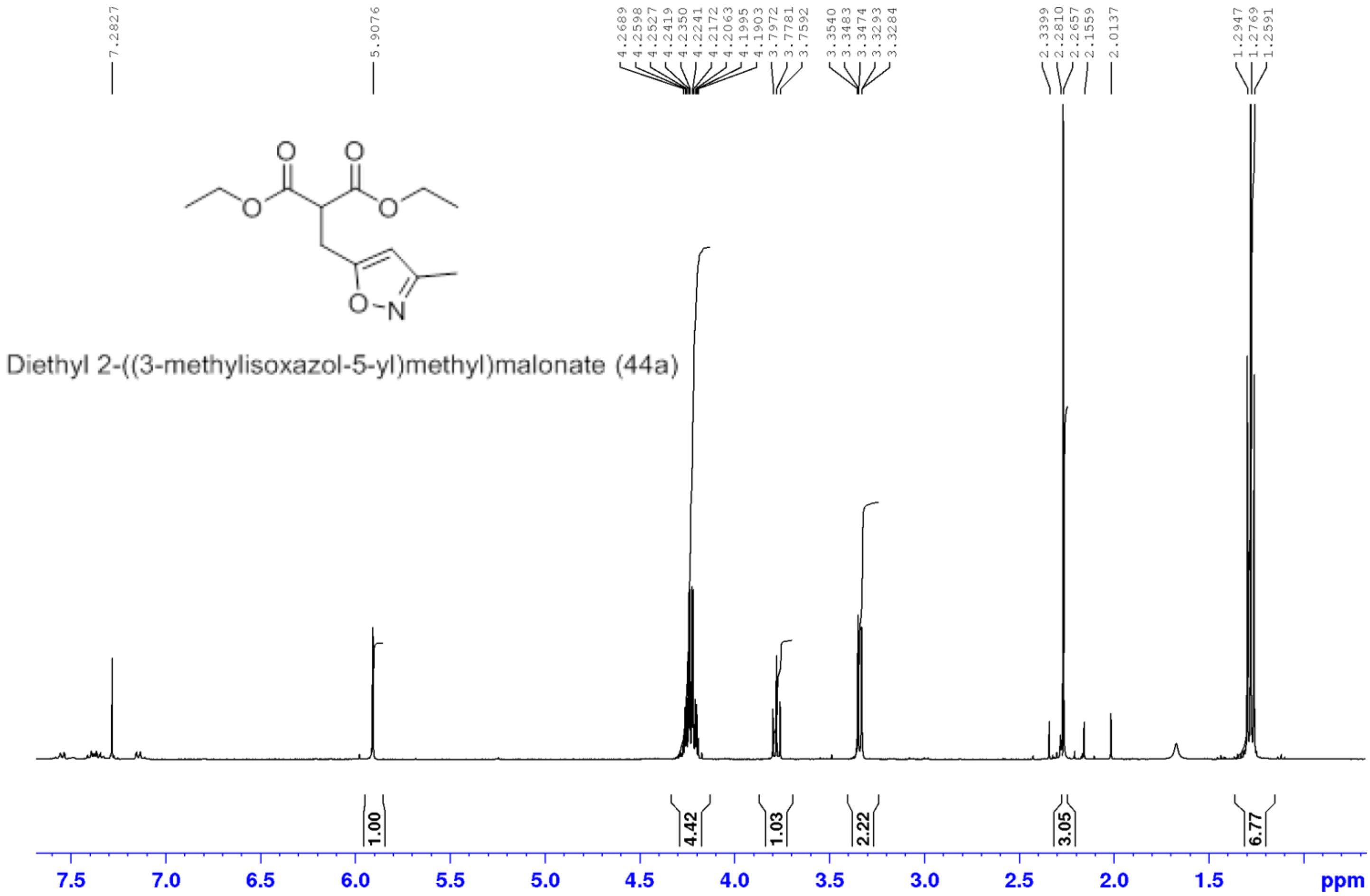
7.2827
5.9076
4.2689
4.2598
4.2527
4.2419
4.2350
4.2241
4.2172
4.2063
4.1995
4.1903
3.7972
3.7781
3.7592
3.3540
3.3483
3.3474
3.3293
3.3284
2.3399
2.2810
2.2657
2.1559
2.0137
1.2947
1.2769
1.2591
Diethyl 2-((3-methylisoxazol-5-yl)methyl)malonate (44a)
1.00
4.42
1.03
2.22
3.05
6.77
7.5
7.0
6.5
6.0
5.5
5.0
4.5
4.0
3.5
3.0
2.5
2.0
1.5
ppm

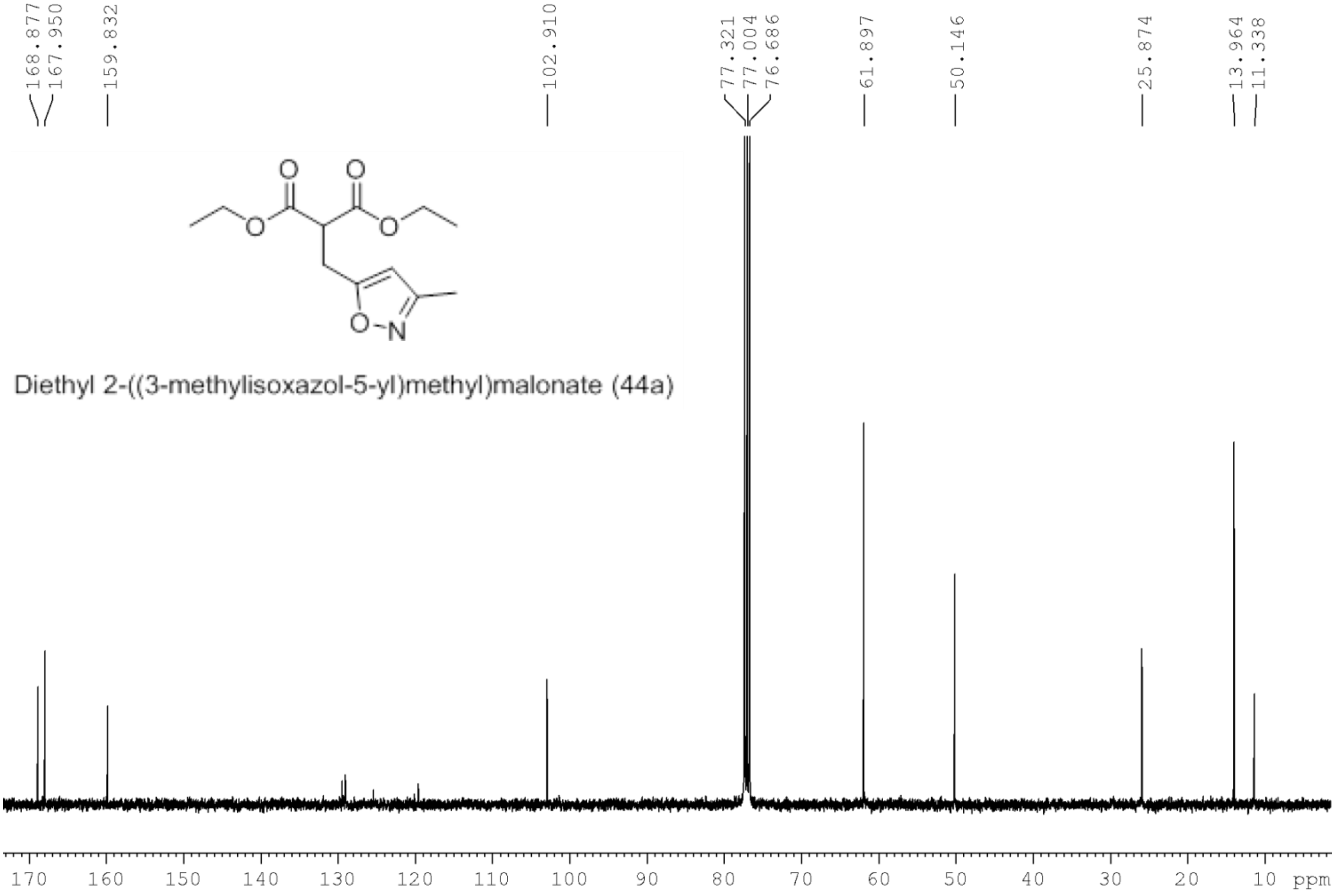
168.877
167.950
159.832
102.910
77.321
77.004
76.686
61.897
50.146
25.874
13.964
11.338
Diethyl 2-((3-methylisoxazol-5-yl)methyl)malonate (44a)
170
160
150
140
130
120
110
100
90
80
70
60
50
40
30
20
10 ppm

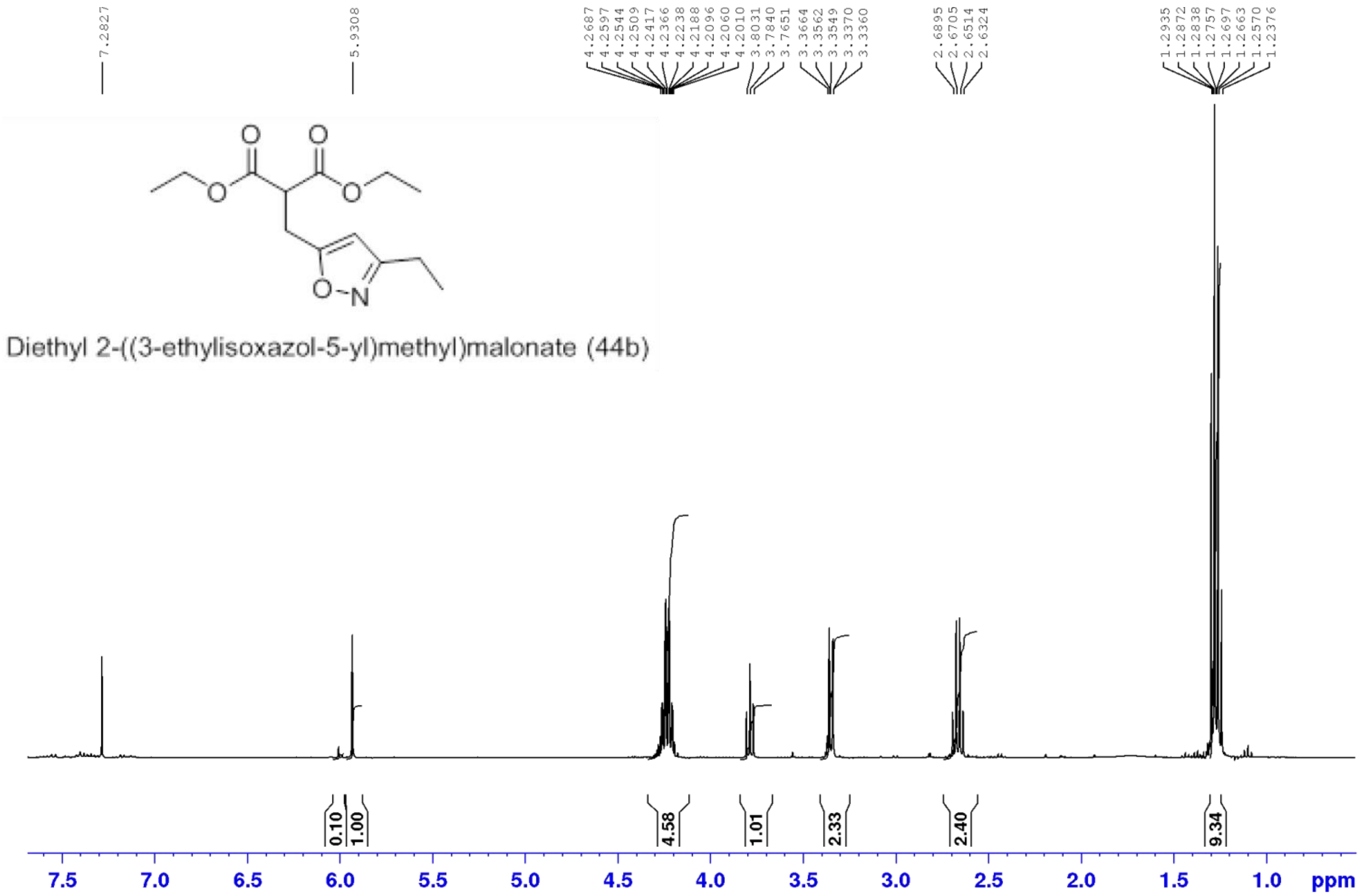
7.2827
5.9308
4.2687 4.2597 4.2544 4.2509 4.2417 4.2366 4.2238 4.2188 4.2096 4.2060 4.2010 3.8031 3.7840 3.7651 3.3664 3.3562 3.3549 3.3370 3.3360
2.6895 2.6705 2.6514 2.6324
1.2935 1.2872 1.2838 1.2757 1.2697 1.2663 1.2570 1.2376
Diethyl 2-((3-ethylisoxazol-5-yl)methyl)malonate (44b)
0.10 1.00 4.58 1.01 2.33 2.40 9.34
7.5 7.0 6.5 6.0 5.5 5.0 4.5 4.0 3.5 3.0 2.5 2.0 1.5 1.0 ppm

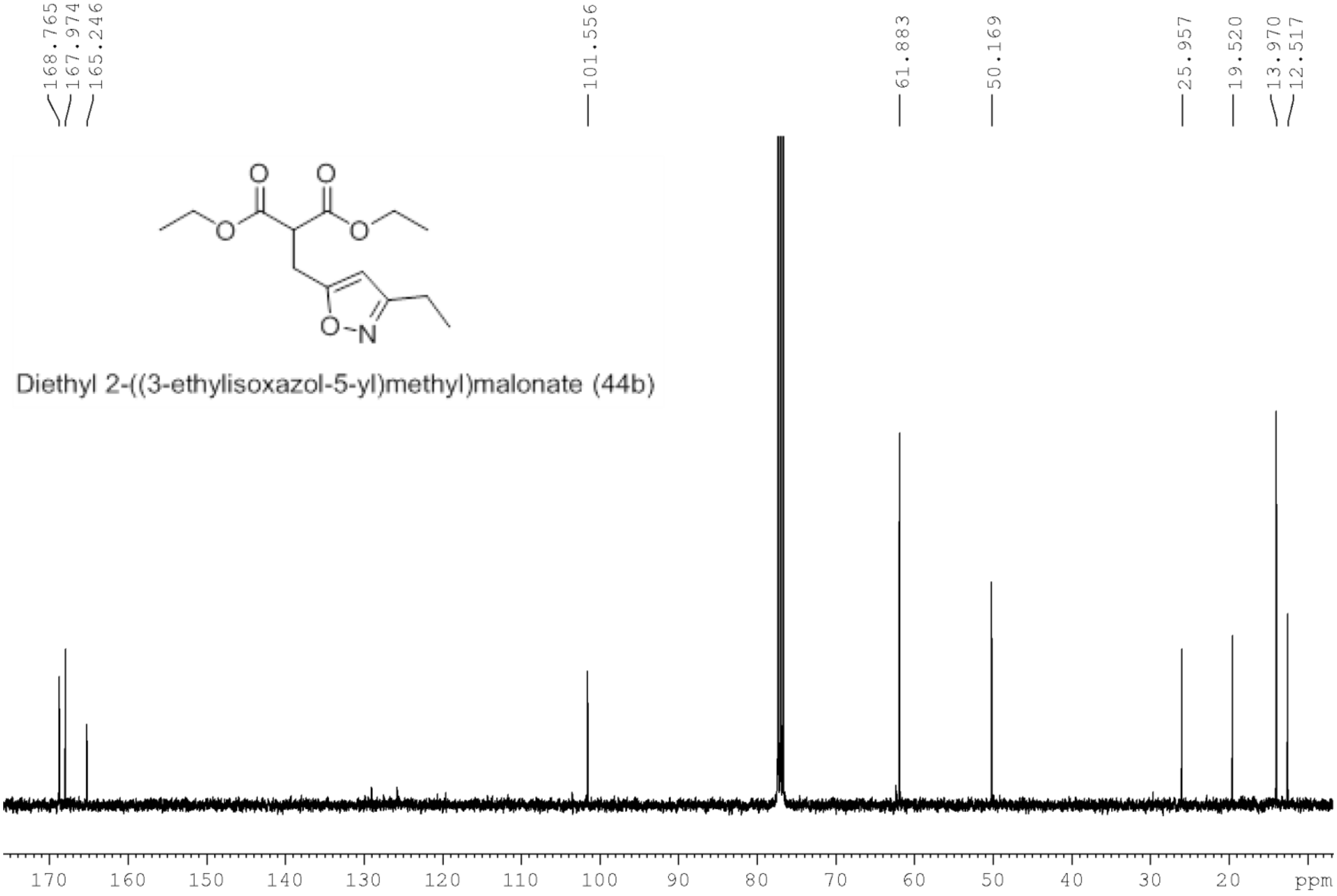
168.765 167.974 165.246
101.556
61.883
50.169
25.957
19.520
13.970 12.517
Diethyl 2-((3-ethylisoxazol-5-yl)methyl)malonate (44b)
170 160 150 140 130 120 110 100 90 80 70 60 50 40 30 20 ppm

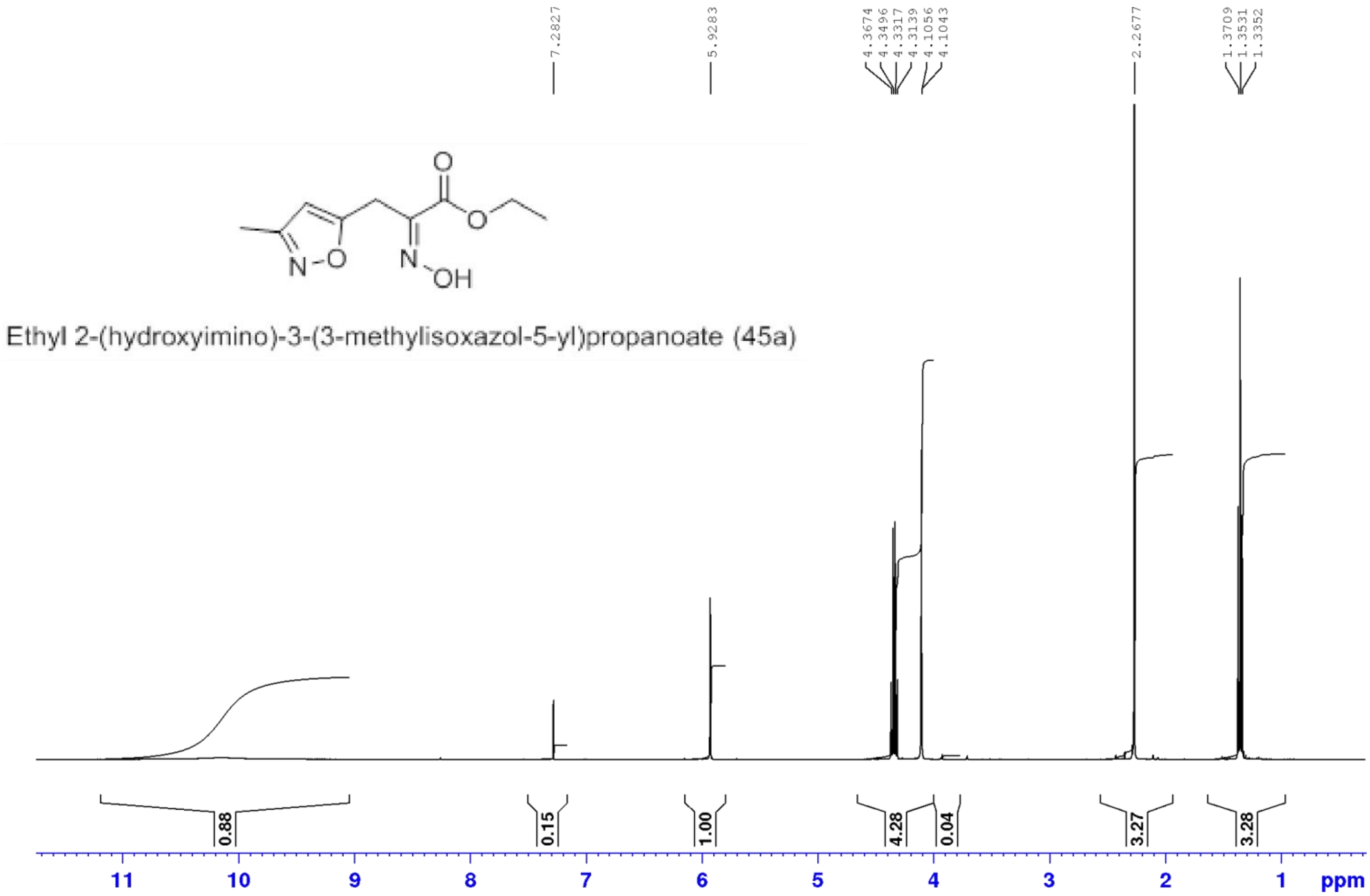
7.2827
5.9283
4.3674
4.3496
4.3317
4.3139
4.1056
4.1043
2.2677
1.3709
1.3531
1.3352
Ethyl 2-(hydroxyimino)-3-(3-methylisoxazol-5-yl)propanoate (45a)
0.88
0.15
1.00
4.28
0.04
3.27
3.28
11
10
9
8
7
6
5
4
3
2
1
ppm

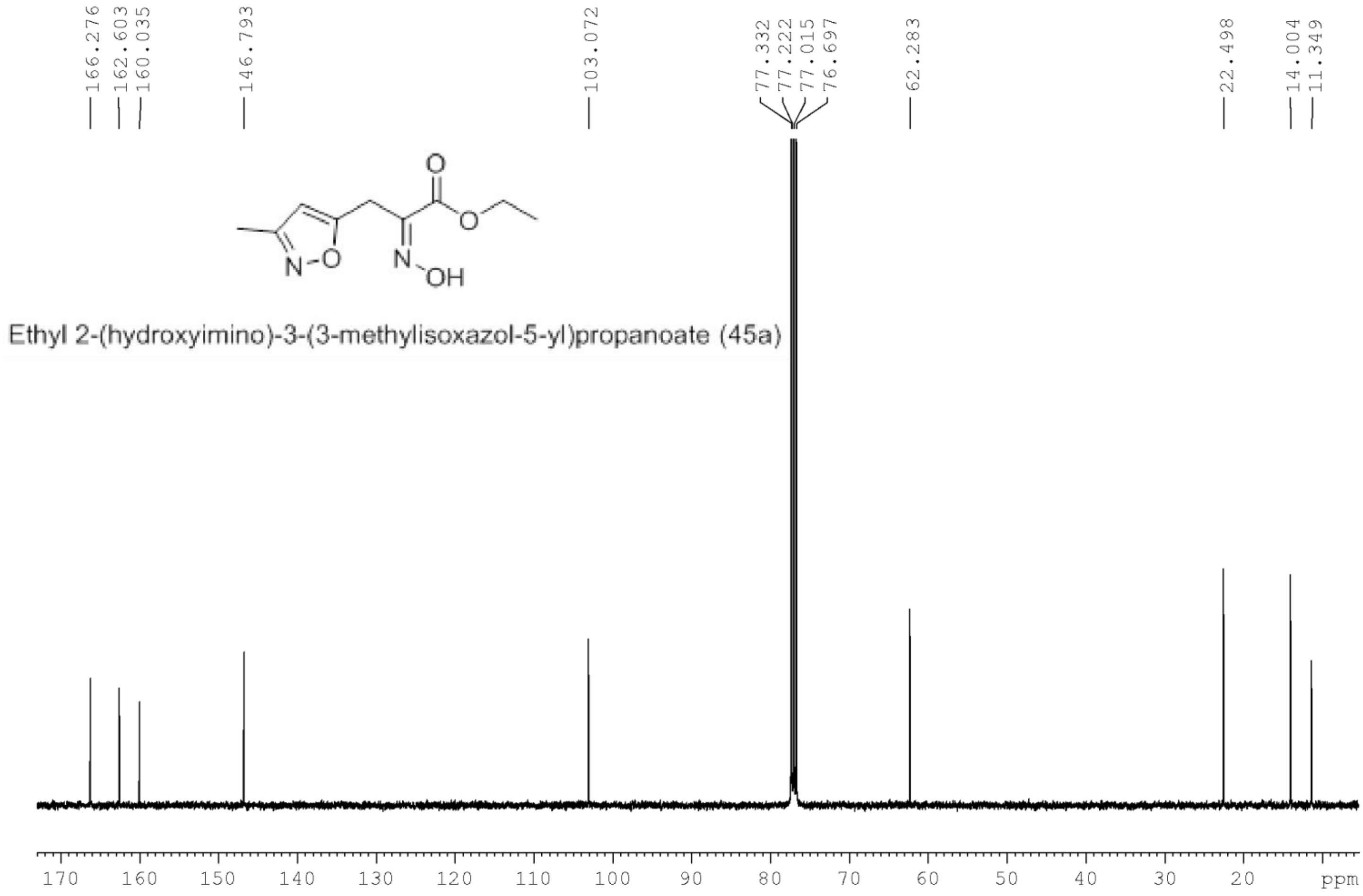
166.276
162.603
160.035
146.793
103.072
77.332
77.222
77.015
76.697
62.283
22.498
14.004
11.349
Ethyl 2-(hydroxyimino)-3-(3-methylisoxazol-5-yl)propanoate (45a)
170
160
150
140
130
120
110
100
90
80
70
60
50
40
30
20
ppm

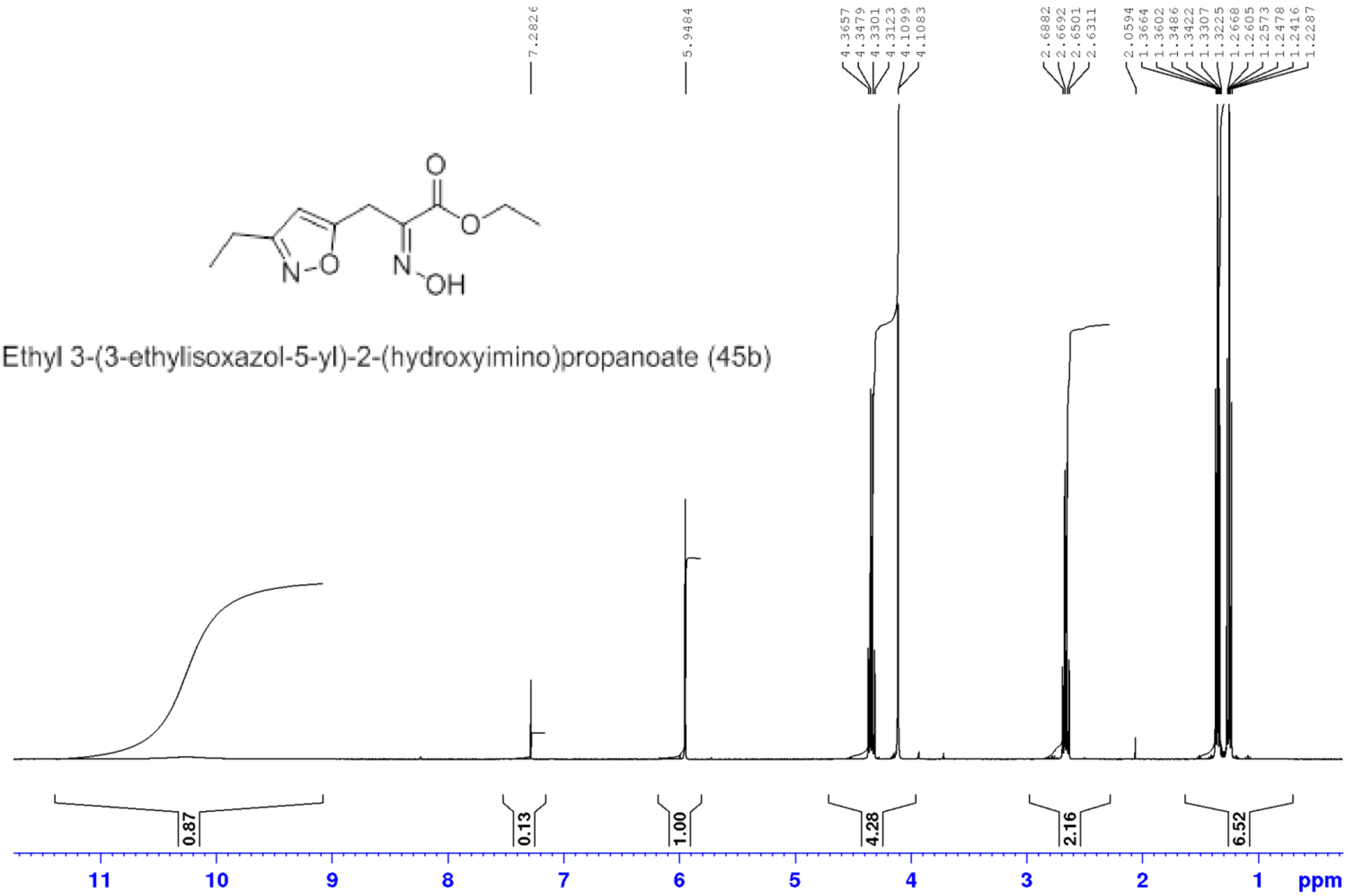
Ethyl 3-(3-ethylisoxazol-5-yl)-2-(hydroxyimino)propanoate (45b)
7.2826
5.9484
4.3657 4.3479 4.3301 4.3123 4.1099 4.1083
2.6882 2.6692 2.6501 2.6311
2.0594 1.3664 1.3602 1.3486 1.3422 1.3307 1.3225 1.2668 1.2605 1.2573 1.2478 1.2416 1.2287
0.87 0.13 1.00 4.28 2.16 6.52
11 10 9 8 7 6 5 4 3 2 1 ppm

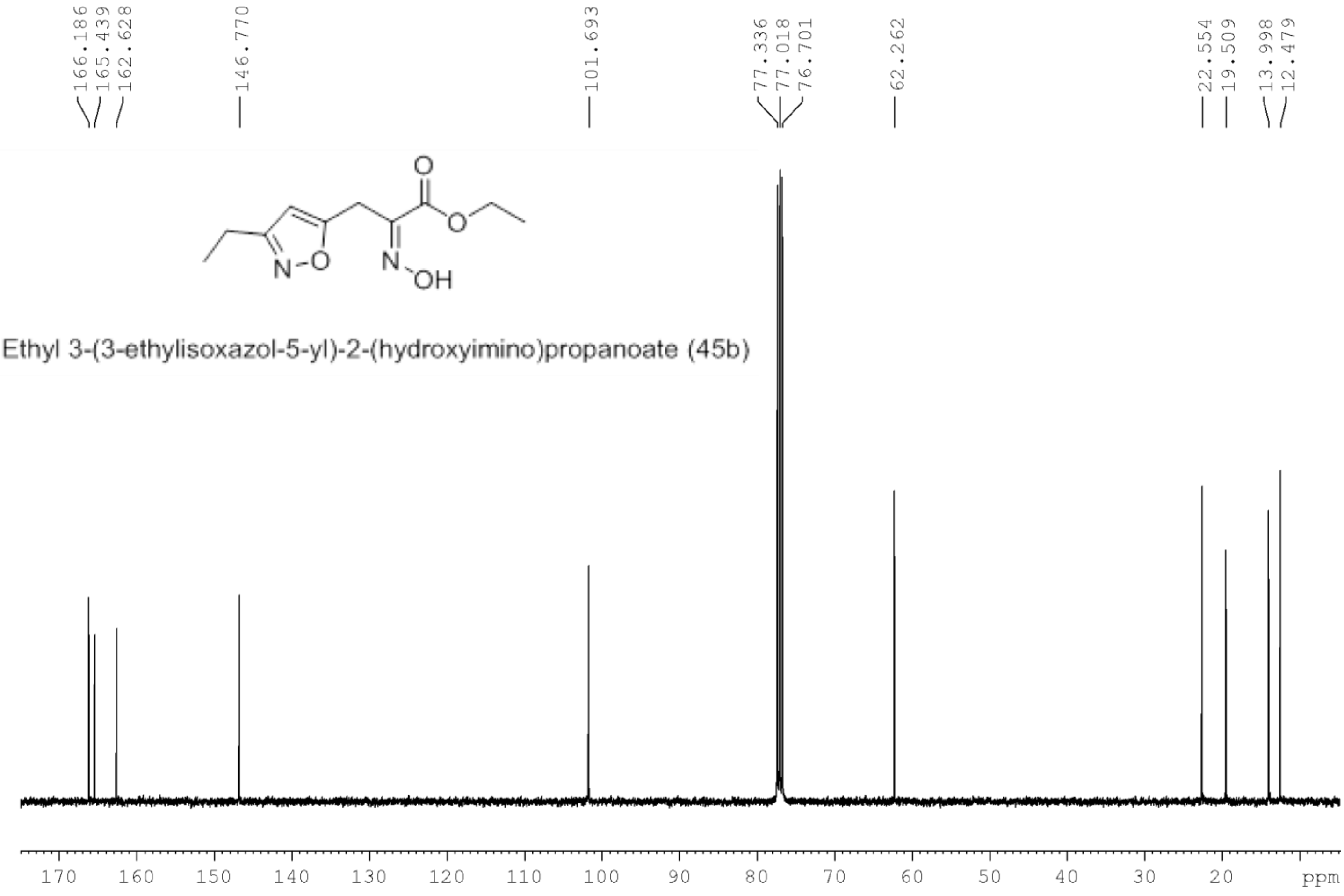
Ethyl 3-(3-ethylisoxazol-5-yl)-2-(hydroxyimino)propanoate (45b)
166.186 165.439 162.628
146.770
101.693
77.336 77.018 76.701
62.262
22.554 19.509 13.998 12.479
170 160 150 140 130 120 110 100 90 80 70 60 50 40 30 20 ppm

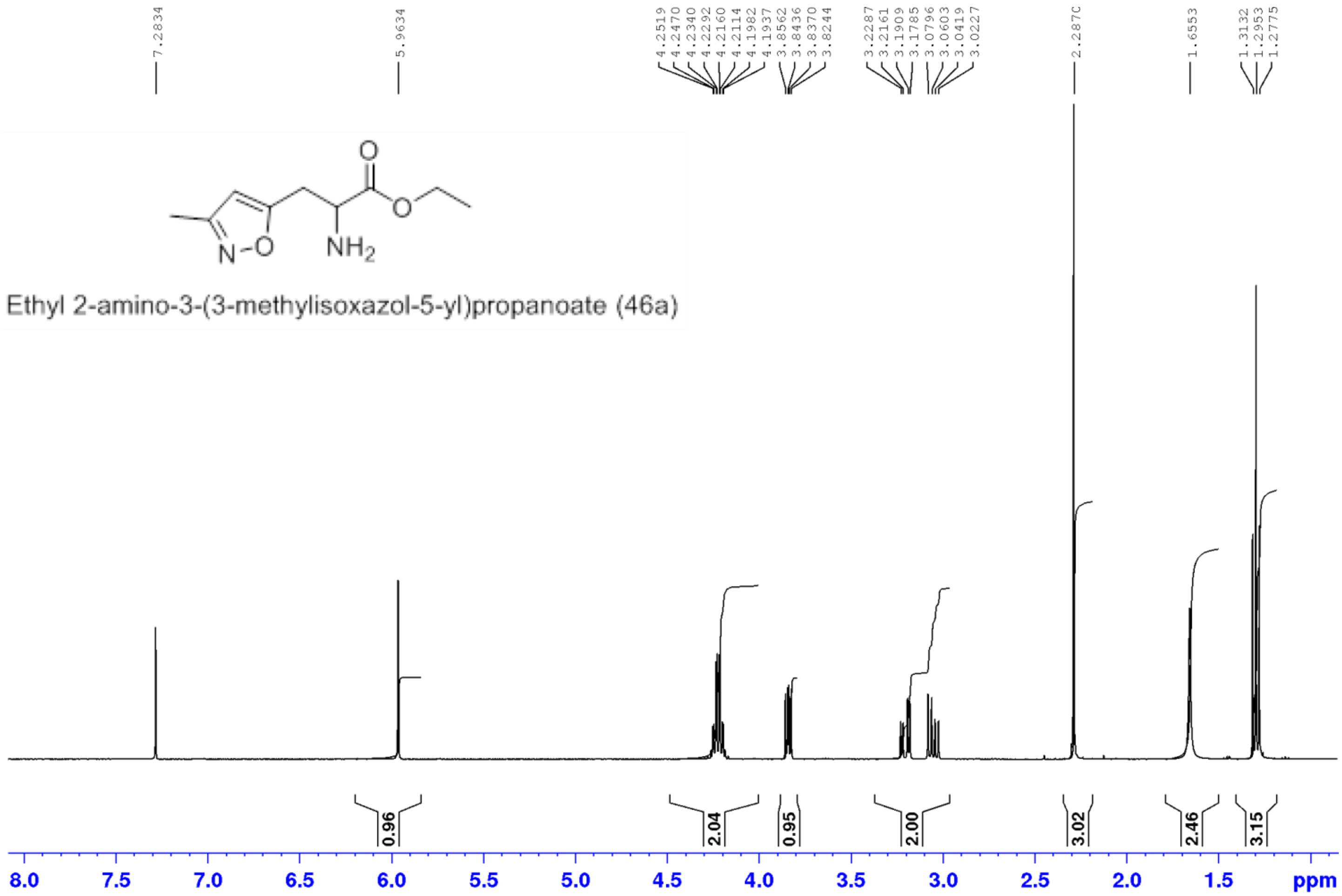
7.2834
5.9634
4.2519 4.2470 4.2340 4.2292 4.2160 4.2114 4.1982 4.1937 3.8562 3.8436 3.8370 3.8244
3.2287 3.2161 3.1909 3.1785 3.0796 3.0603 3.0419 3.0227
2.2870
1.6553
1.3132 1.2953 1.2775
Ethyl 2-amino-3-(3-methylisoxazol-5-yl)propanoate (46a)
0.96 2.04 0.95 2.00 3.02 2.46 3.15
8.0 7.5 7.0 6.5 6.0 5.5 5.0 4.5 4.0 3.5 3.0 2.5 2.0 1.5 ppm

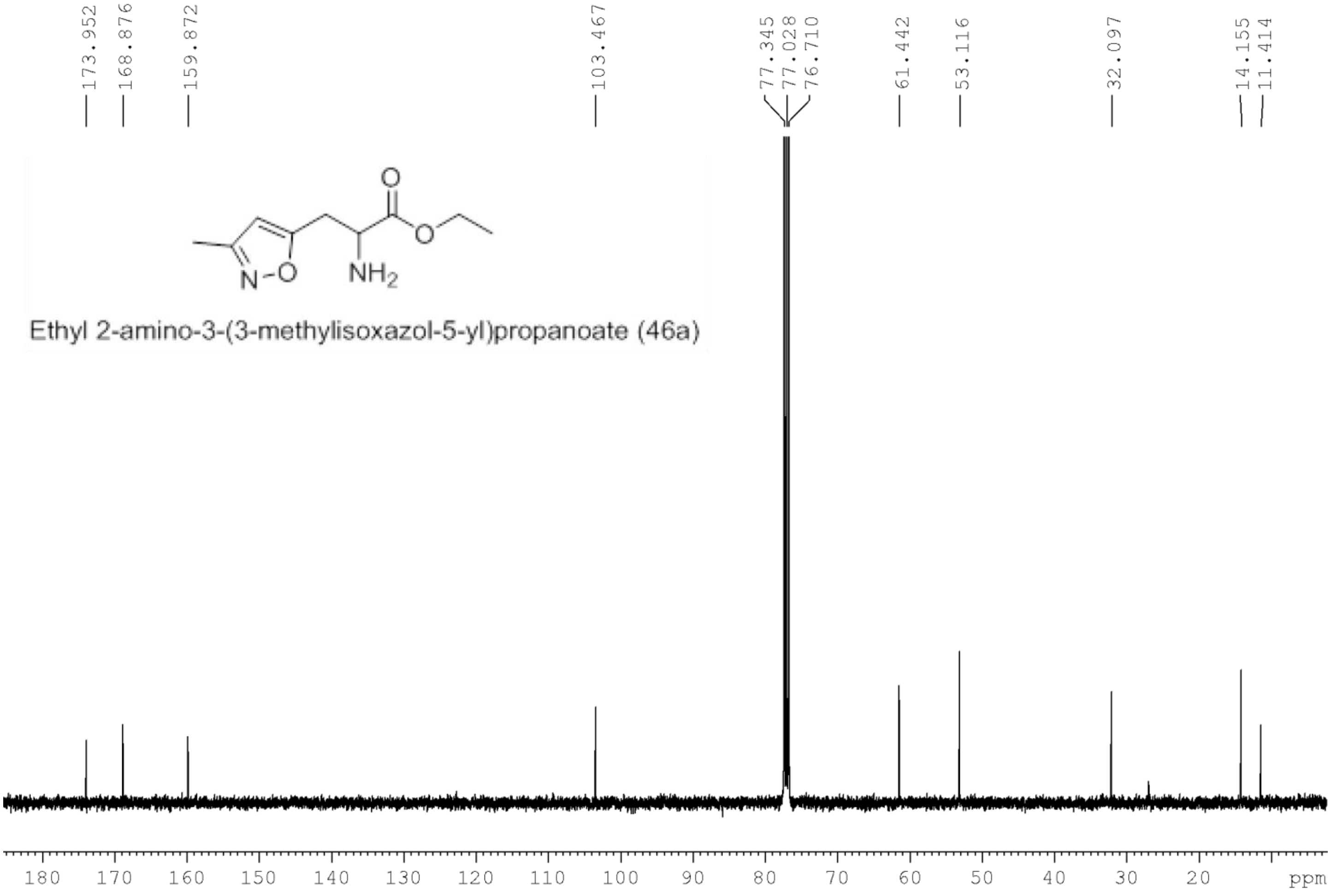
173.952 168.876 159.872
103.467
77.345 77.028 76.710
61.442 53.116 32.097 14.155 11.414
Ethyl 2-amino-3-(3-methylisoxazol-5-yl)propanoate (46a)
180 170 160 150 140 130 120 110 100 90 80 70 60 50 40 30 20 ppm

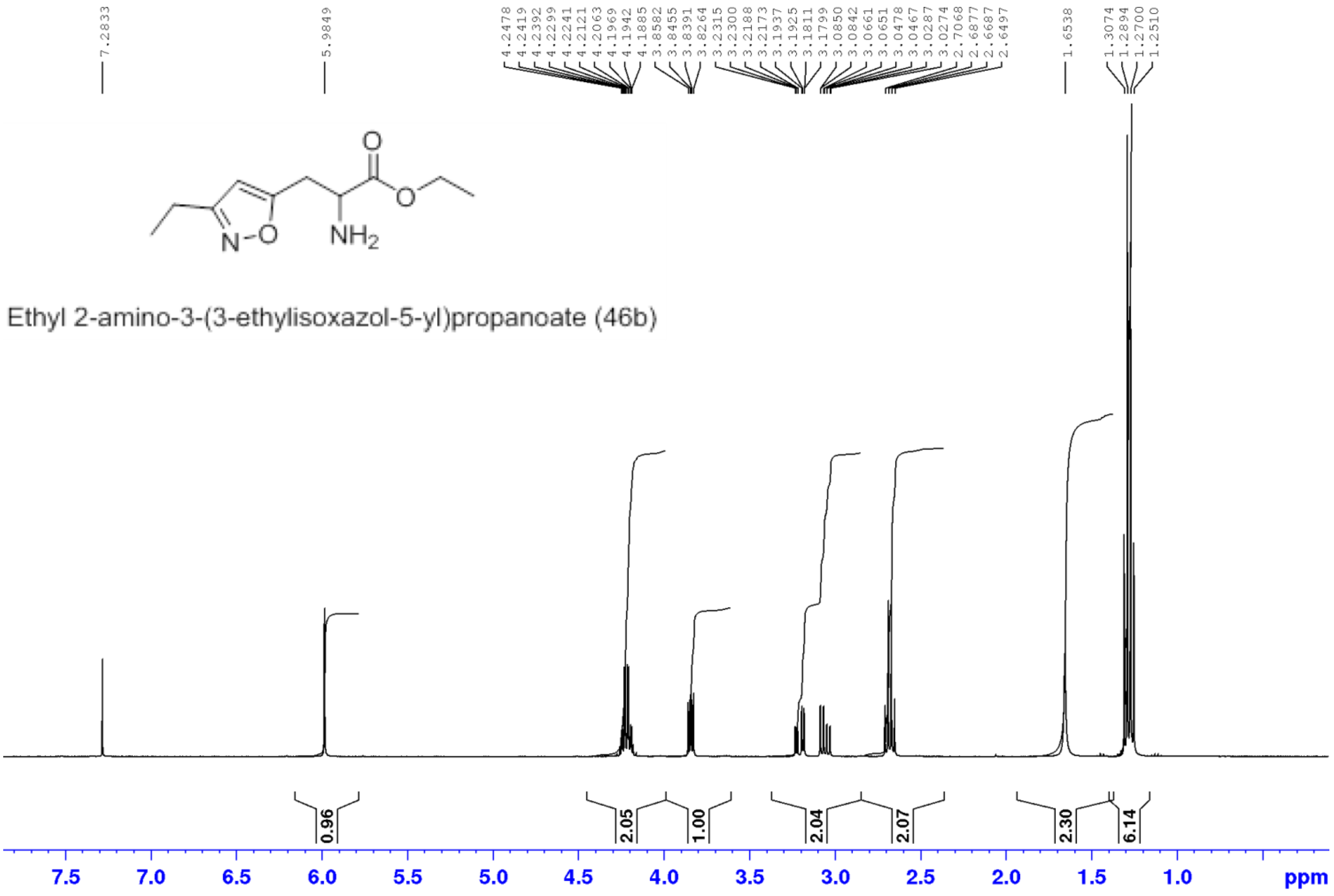

Ethyl 2-amino-3-(3-ethylisoxazol-5-yl)propanoate (46b)

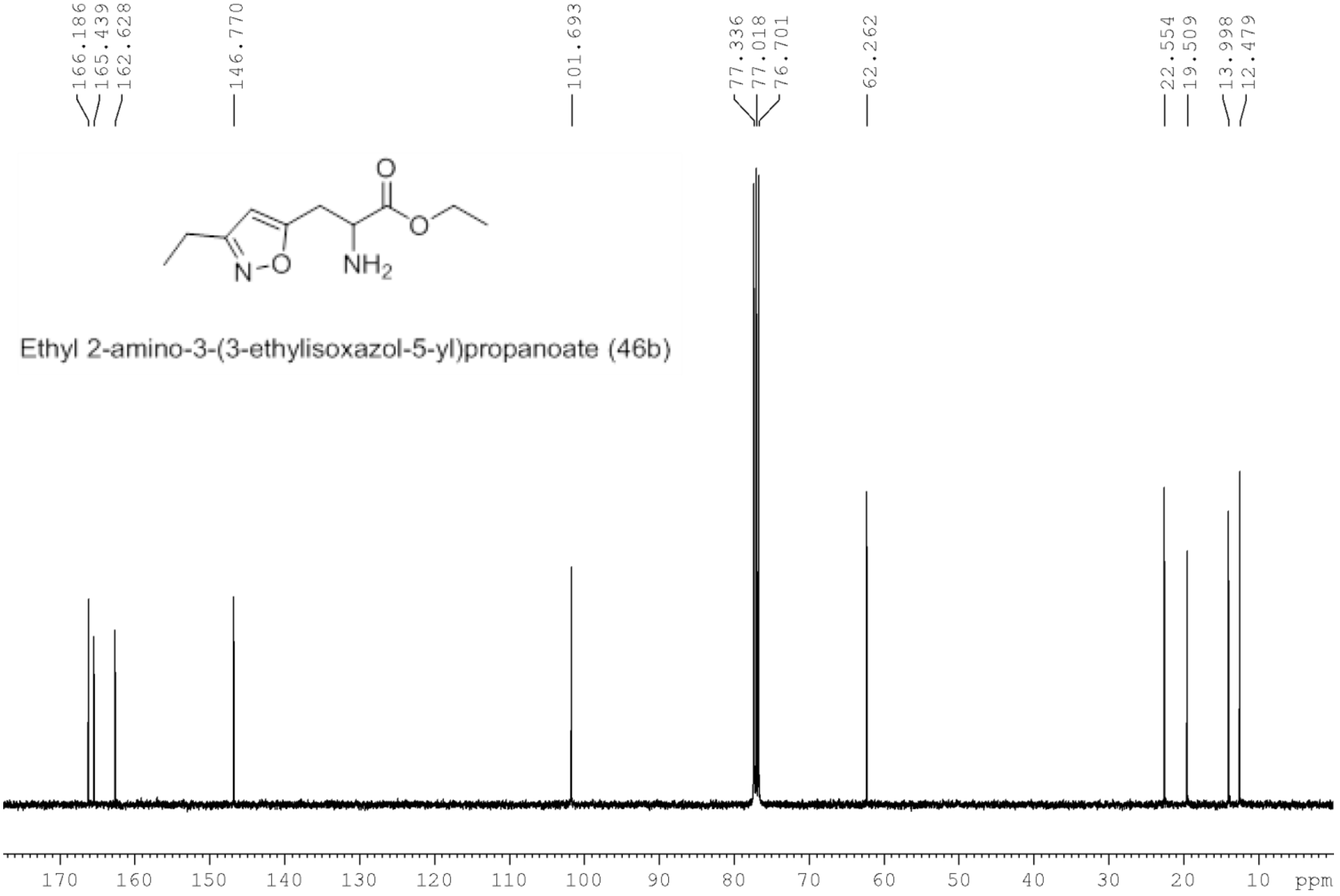

Ethyl 2-amino-3-(3-ethylisoxazol-5-yl)propanoate (46b)

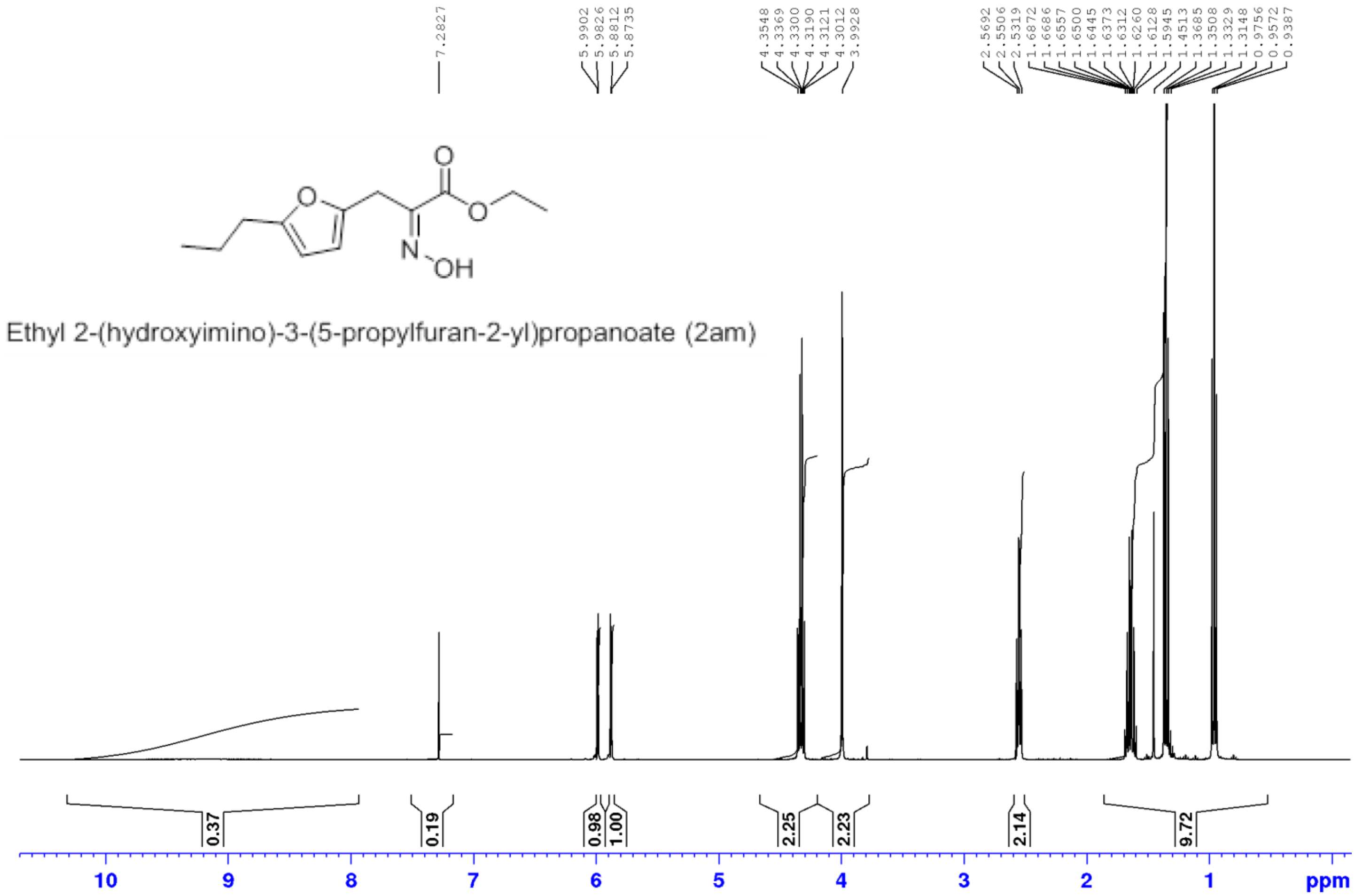
Ethyl 2-(hydroxyimino)-3-(5-propylfuran-2-yl)propanoate (2am)
7.2827
5.9902
5.9826
5.8812
5.8735
4.3548
4.3369
4.3300
4.3190
4.3121
4.3012
3.9928
2.5692
2.5506
2.5319
1.6872
1.6686
1.6557
1.6500
1.6445
1.6373
1.6312
1.6260
1.6128
1.5945
1.4513
1.3685
1.3508
1.3329
1.3148
0.9756
0.9572
0.9387
0.37
0.19
0.98
1.00
2.25
2.23
2.14
9.72
10
9
8
7
6
5
4
3
2
1
ppm

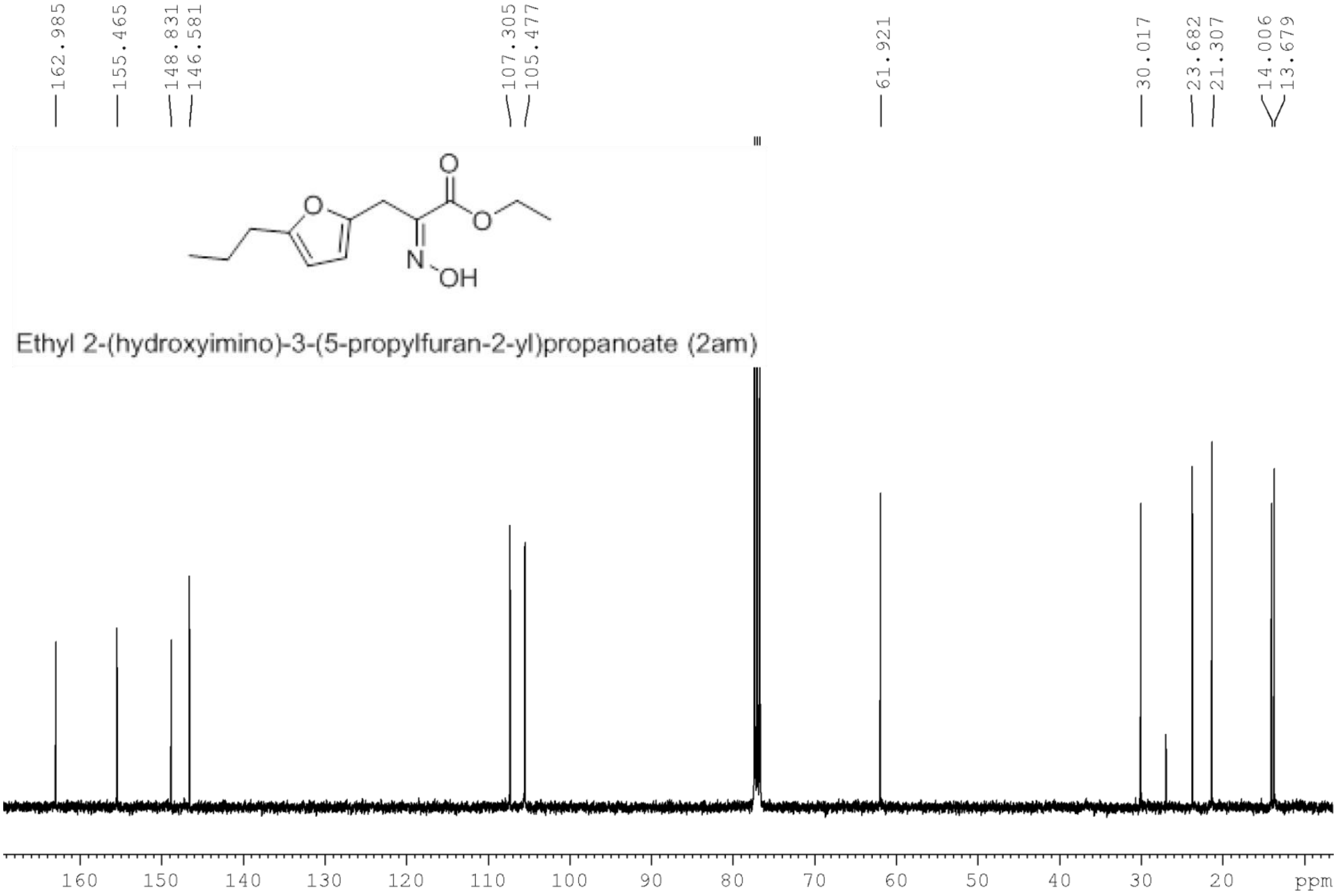
Ethyl 2-(hydroxyimino)-3-(5-propylfuran-2-yl)propanoate (2am)
162.985
155.465
148.831
146.581
107.305
105.477
61.921
30.017
23.682
21.307
14.006
13.679
160
150
140
130
120
110
100
90
80
70
60
50
40
30
20
ppm

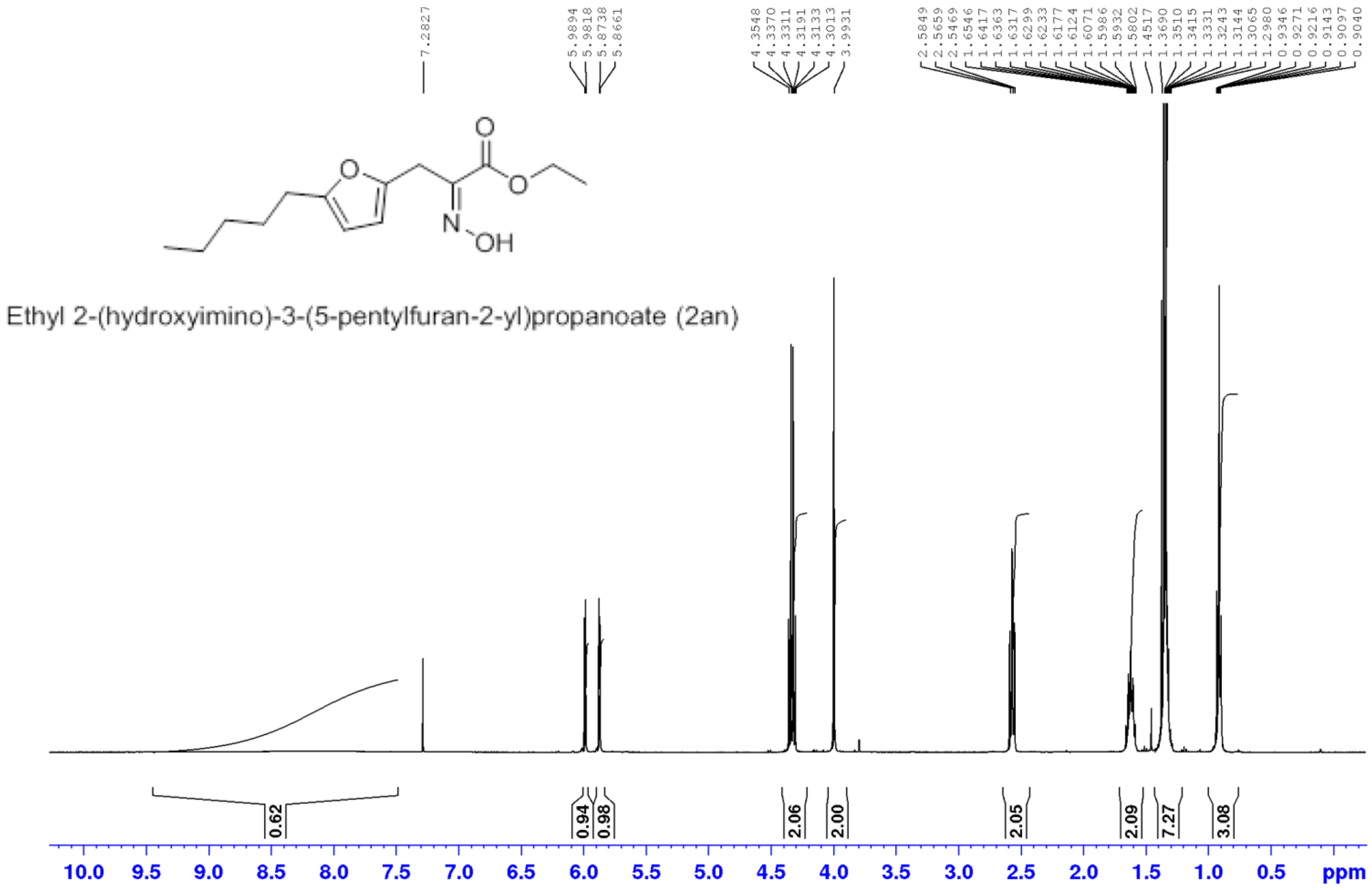

Ethyl 2-(hydroxyimino)-3-(5-pentylfuran-2-yl)propanoate (2an)

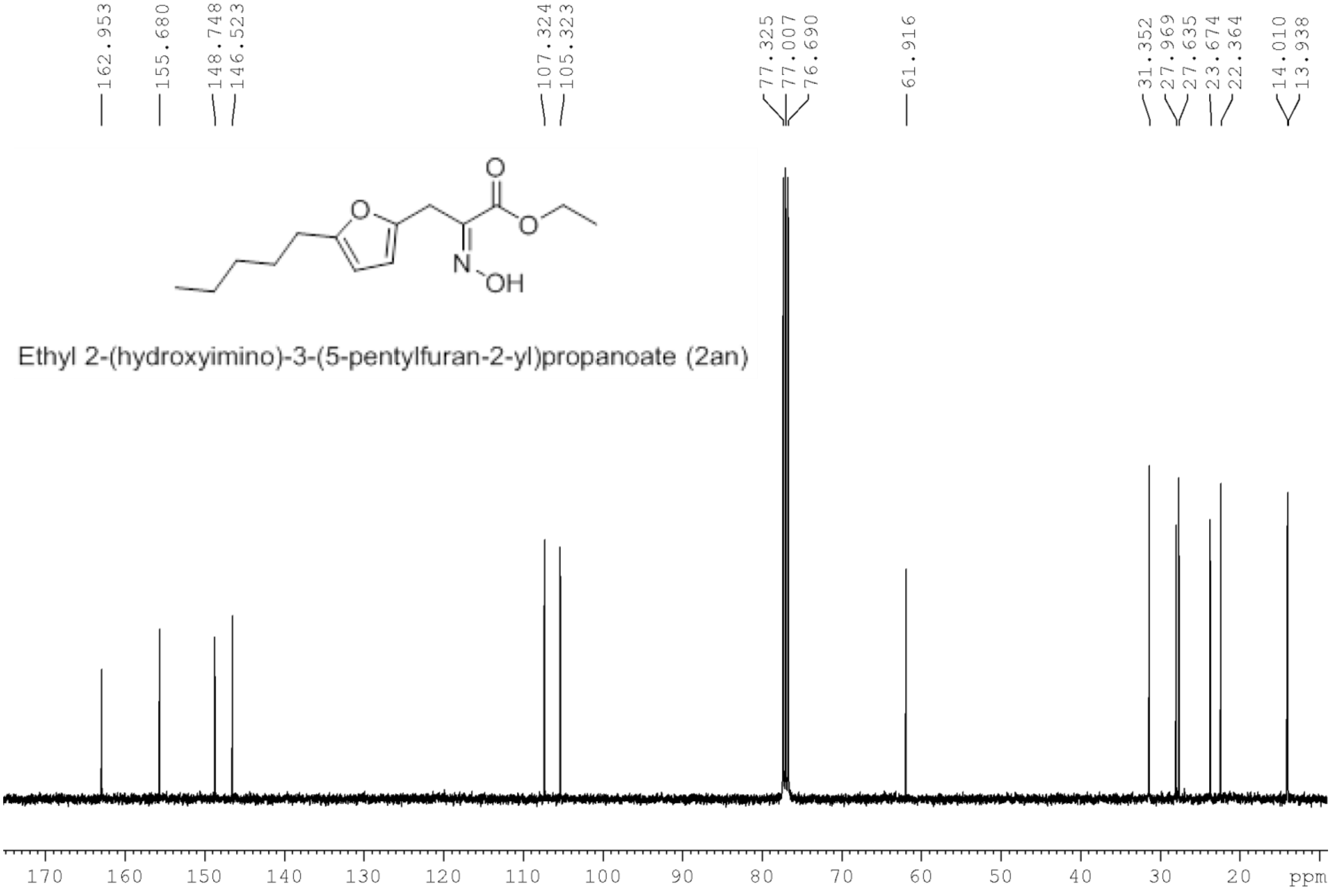

Ethyl 2-(hydroxyimino)-3-(5-pentylfuran-2-yl)propanoate (2an)

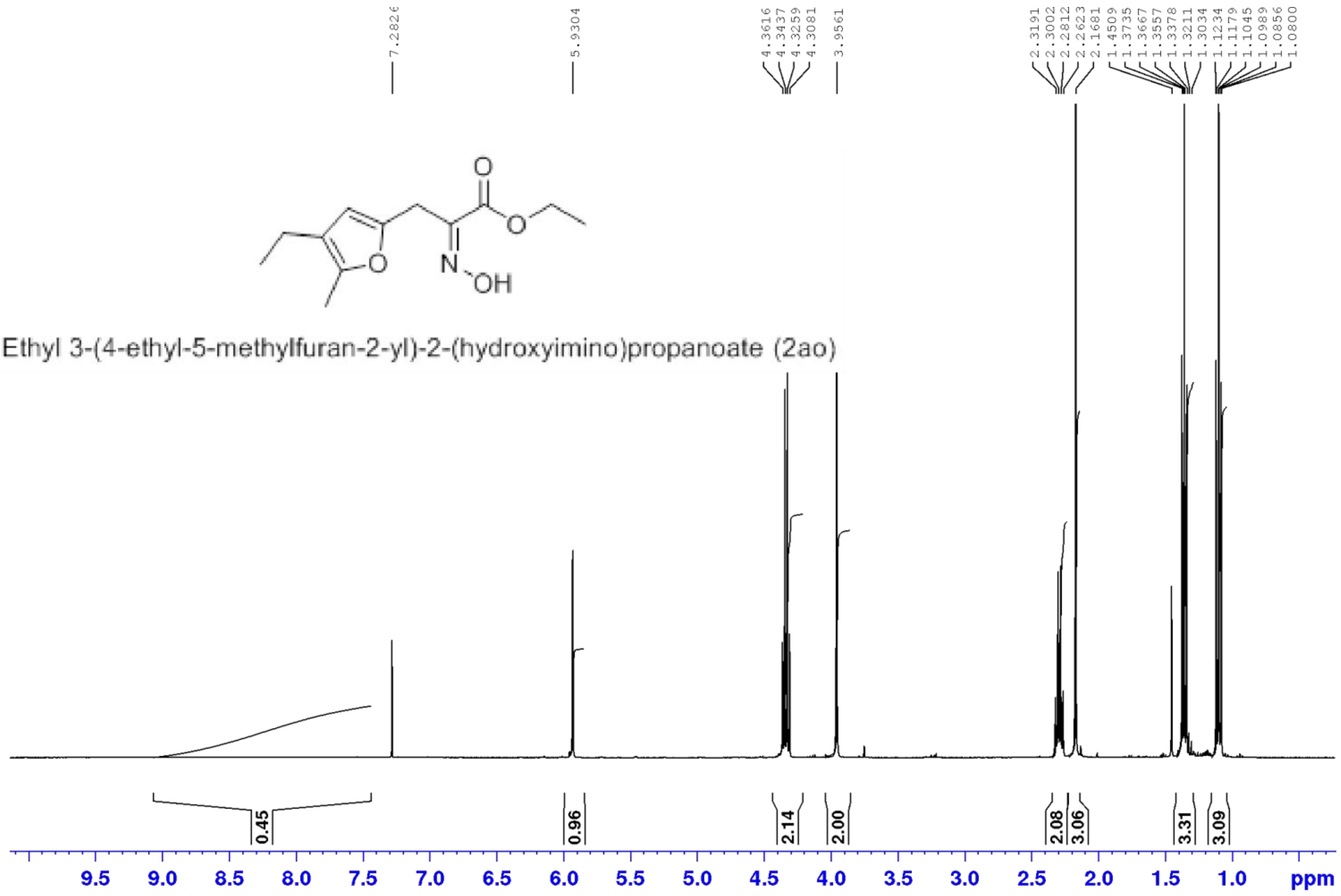

7.2826
5.9304
4.3616
4.3437
4.3259
4.3081
3.9561
2.3191
2.3002
2.2812
2.2623
2.1681
1.4509
1.3735
1.3667
1.3557
1.3378
1.3211
1.3034
1.1234
1.1179
1.1045
1.0989
1.0856
1.0800
O
O
N
OH
O
Ethyl 3-(4-ethyl-5-methylfuran-2-yl)-2-(hydroxyimino)propanoate (2ao)
0.45
0.96
2.14
2.00
2.08
3.06
3.31
3.09
9.5 9.0 8.5 8.0 7.5 7.0 6.5 6.0 5.5 5.0 4.5 4.0 3.5 3.0 2.5 2.0 1.5 1.0 ppm

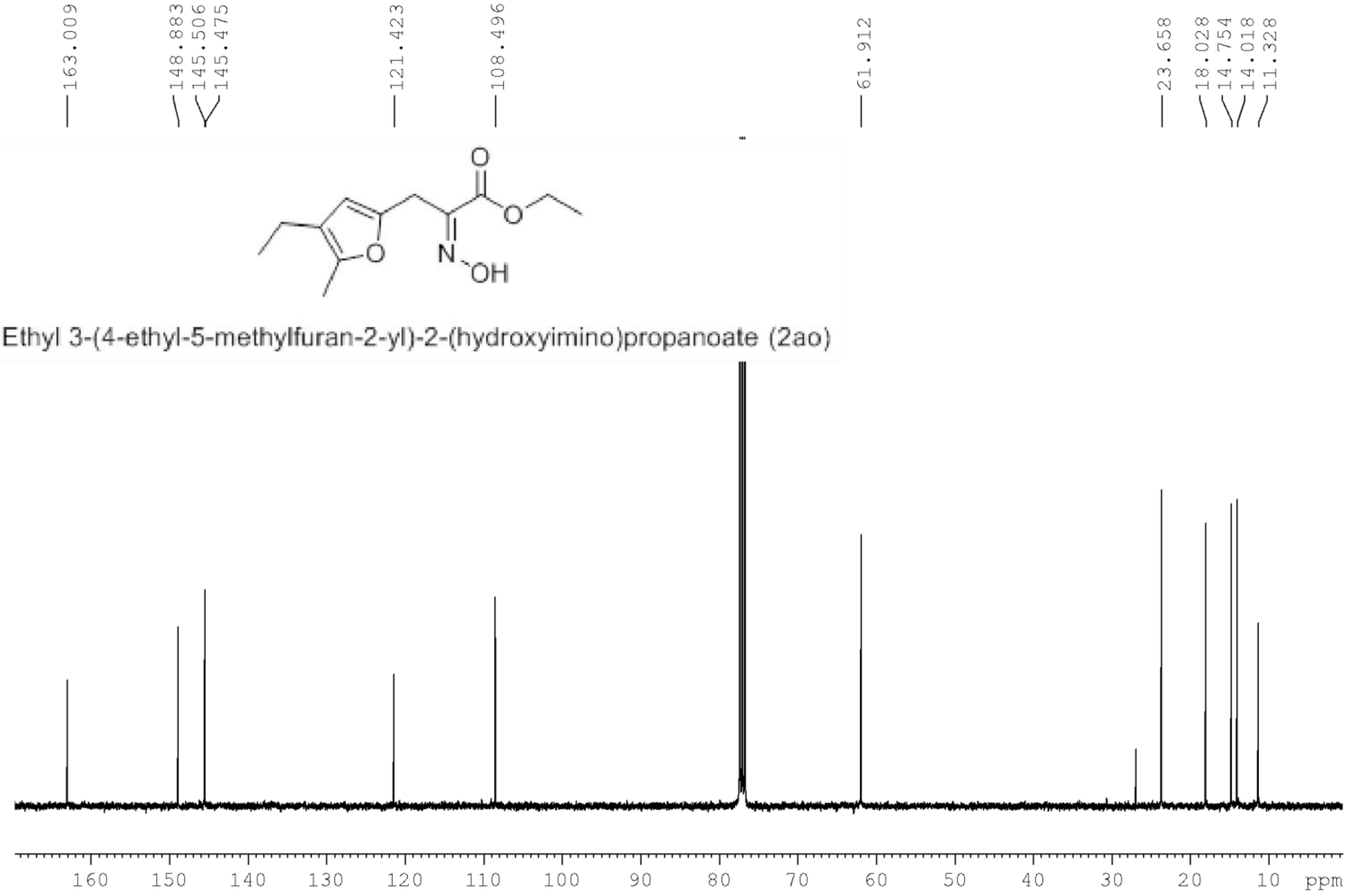

163.009
148.883
145.506
145.475
121.423
108.496
61.912
23.658
18.028
14.754
14.018
11.328
O
O
N
OH
O
Ethyl 3-(4-ethyl-5-methylfuran-2-yl)-2-(hydroxyimino)propanoate (2ao)
160 150 140 130 120 110 100 90 80 70 60 50 40 30 20 10 ppm

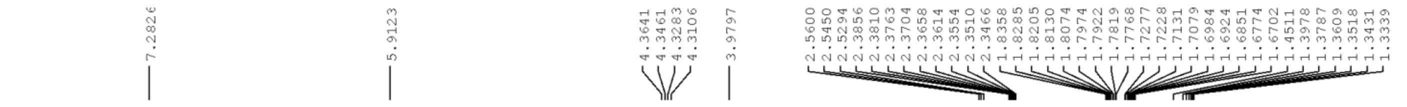

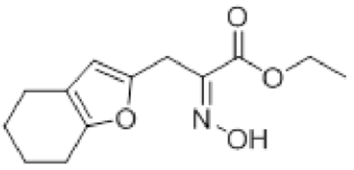

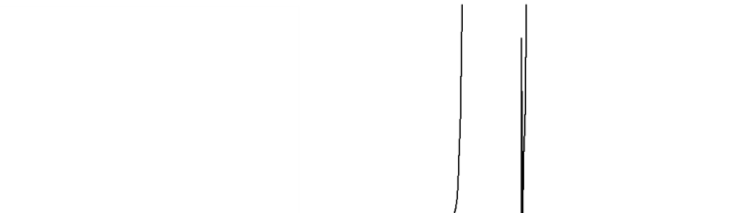

Ethyl 2-(hydroxyimino)-3-(4,5,6,7-tetrahydrobenzofuran-2-yl)propanoate (2ap)

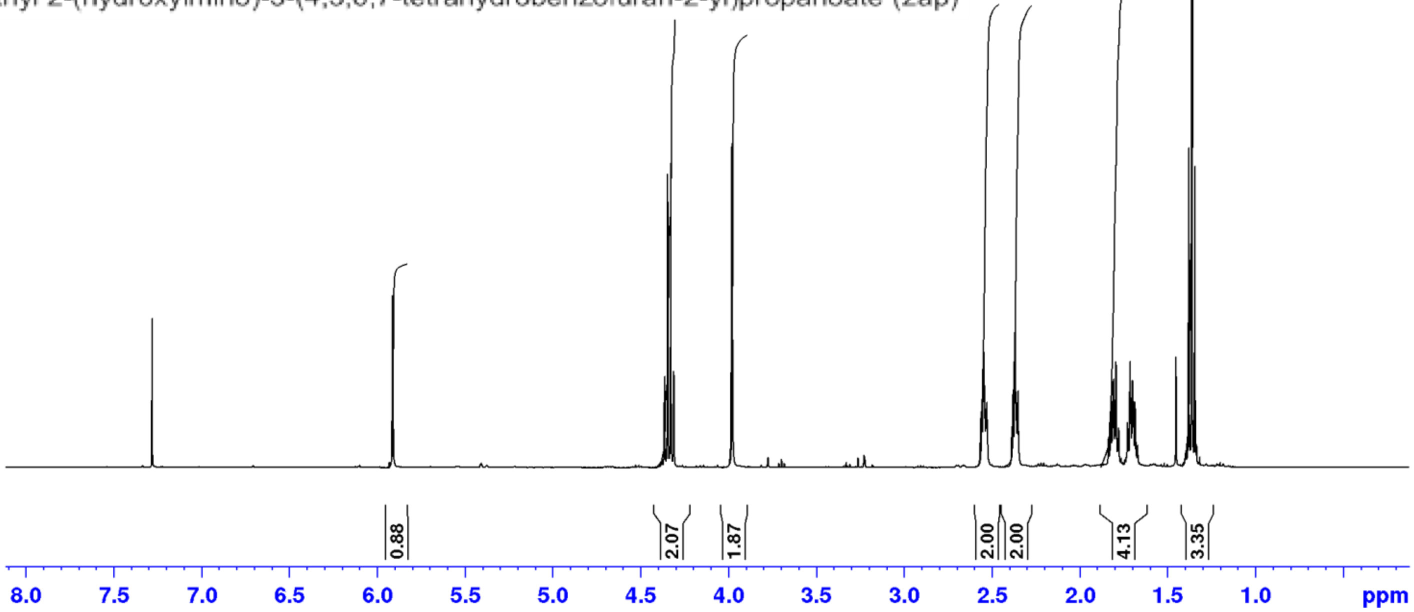

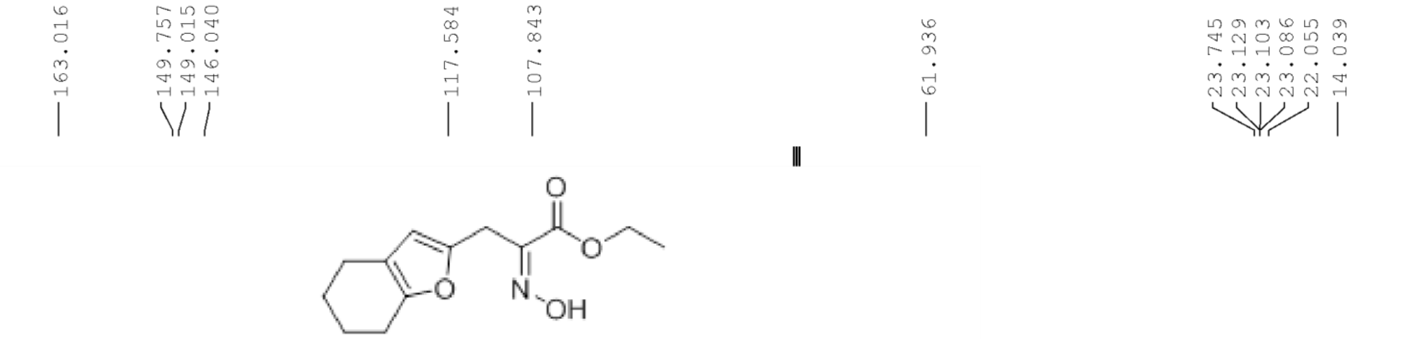

Ethyl 2-(hydroxyimino)-3-(4,5,6,7-tetrahydrobenzofuran-2-yl)propanoate (2ap)

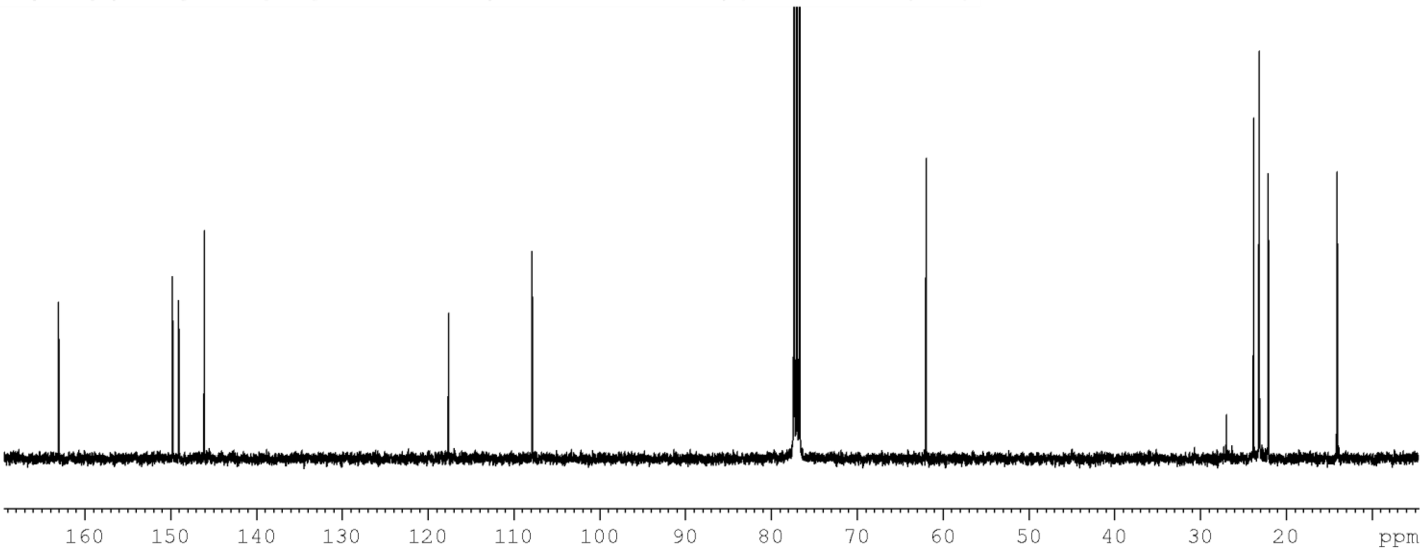

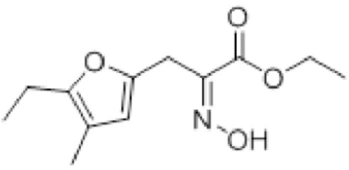

Ethyl-3-(5-ethyl-4-methylfuran-2-yl)-2-(hydroxyimino)propanoate (2aq)

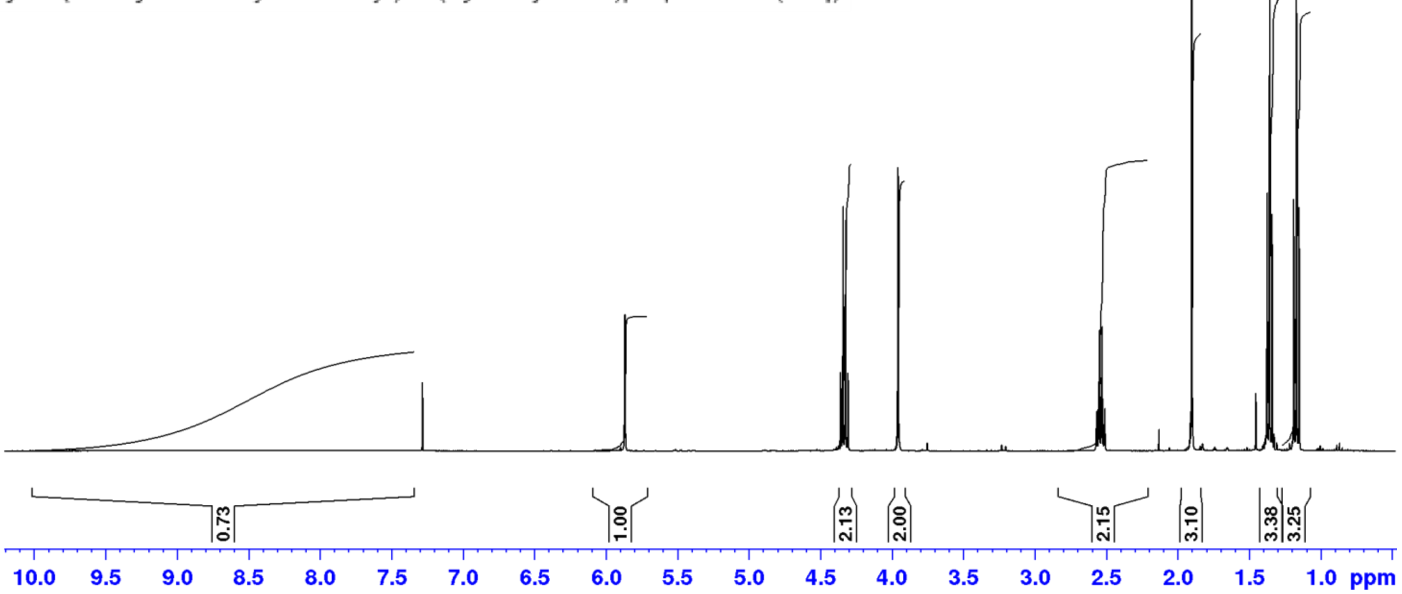

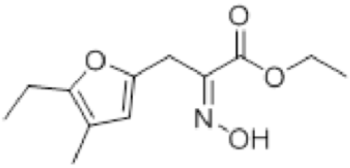

Ethyl-3-(5-ethyl-4-methylfuran-2-yl)-2-(hydroxyimino)propanoate (2aq)

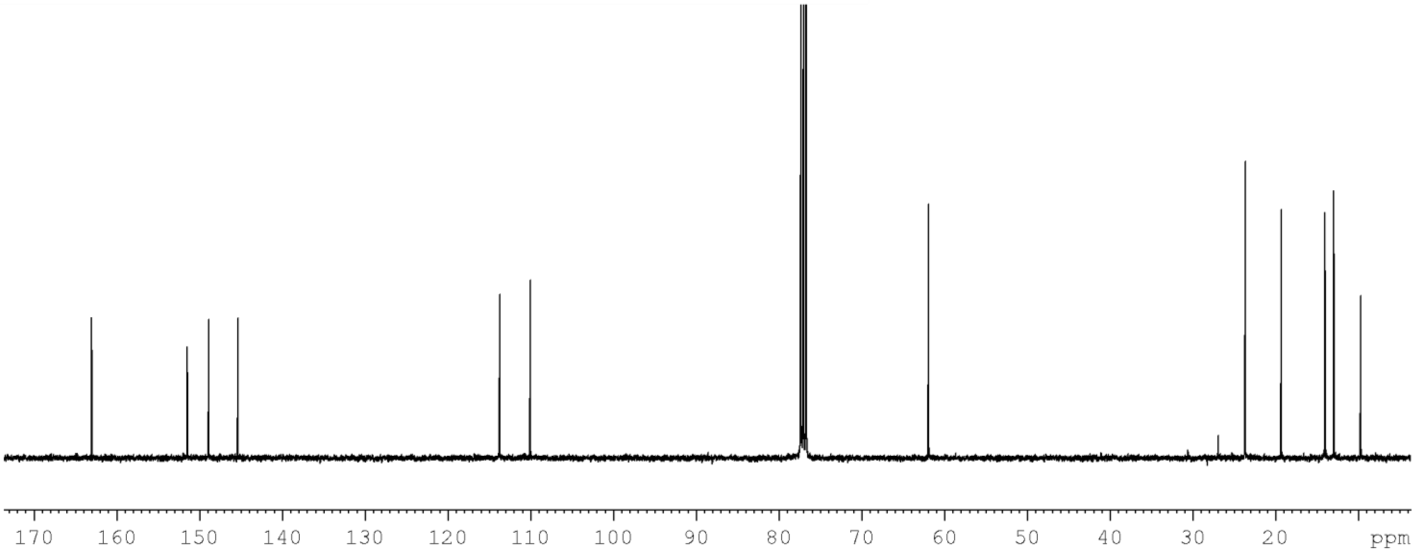

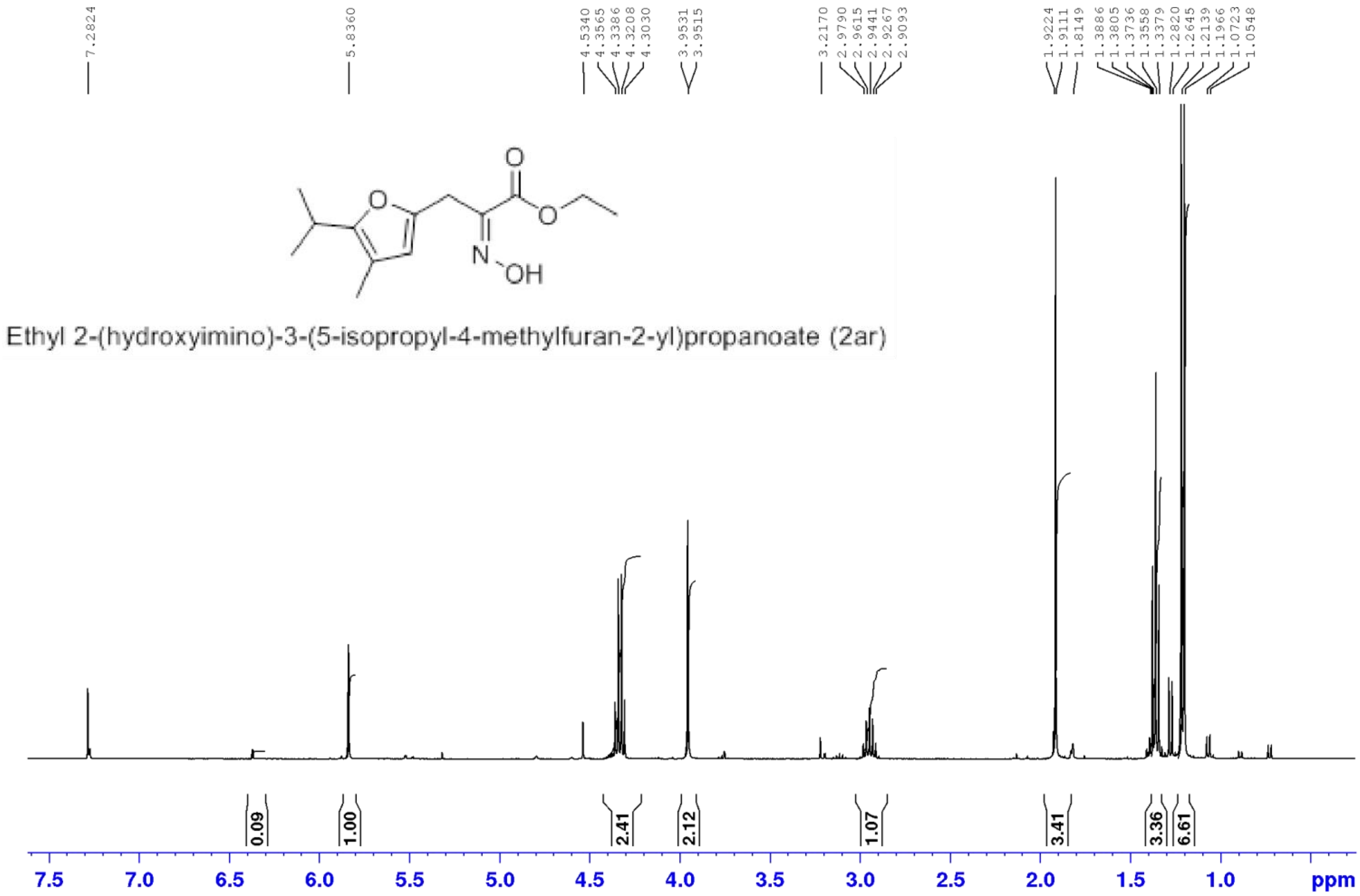

Ethyl 2-(hydroxyimino)-3-(5-isopropyl-4-methylfuran-2-yl)propanoate (2ar)

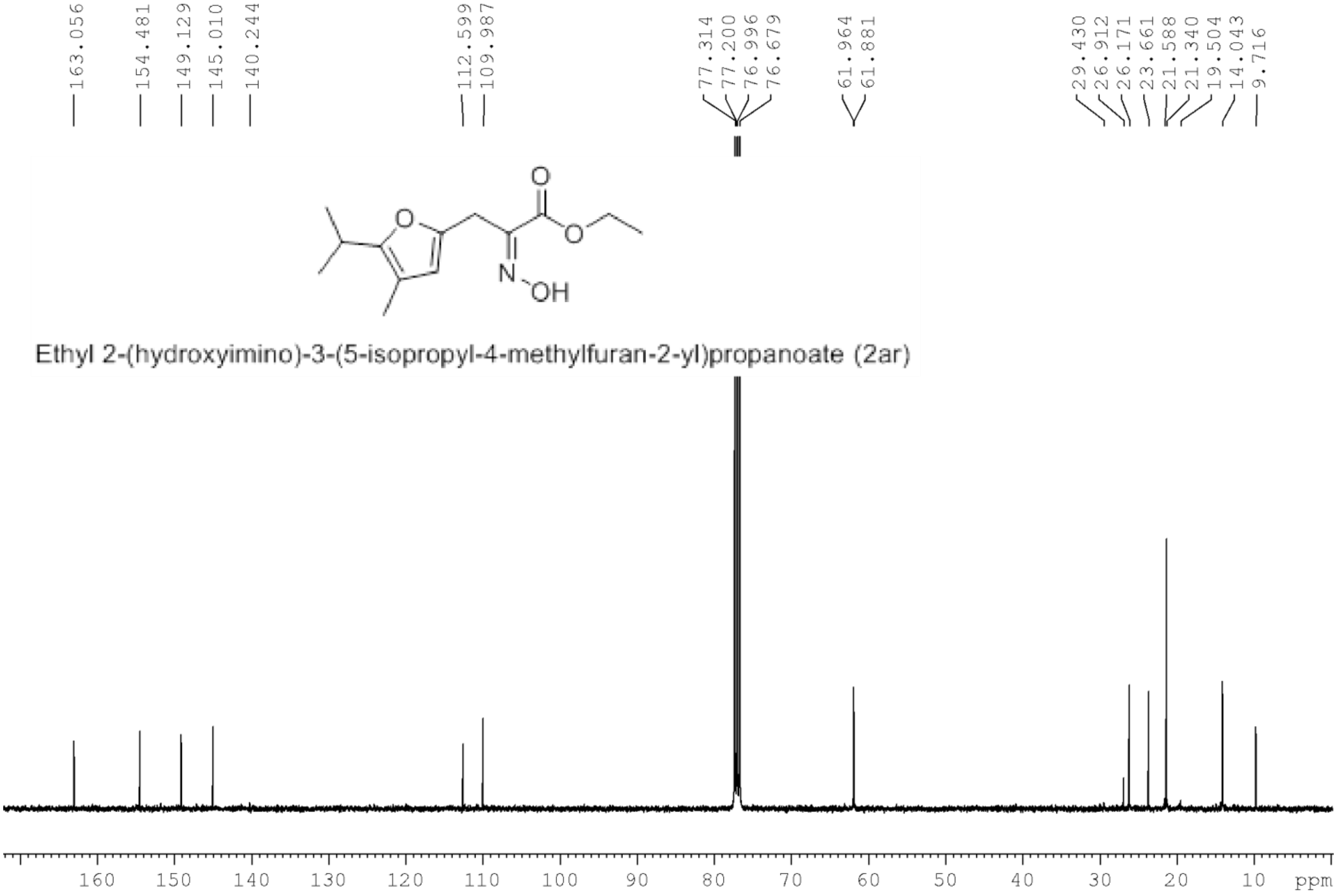

Ethyl 2-(hydroxyimino)-3-(5-isopropyl-4-methylfuran-2-yl)propanoate (2ar)

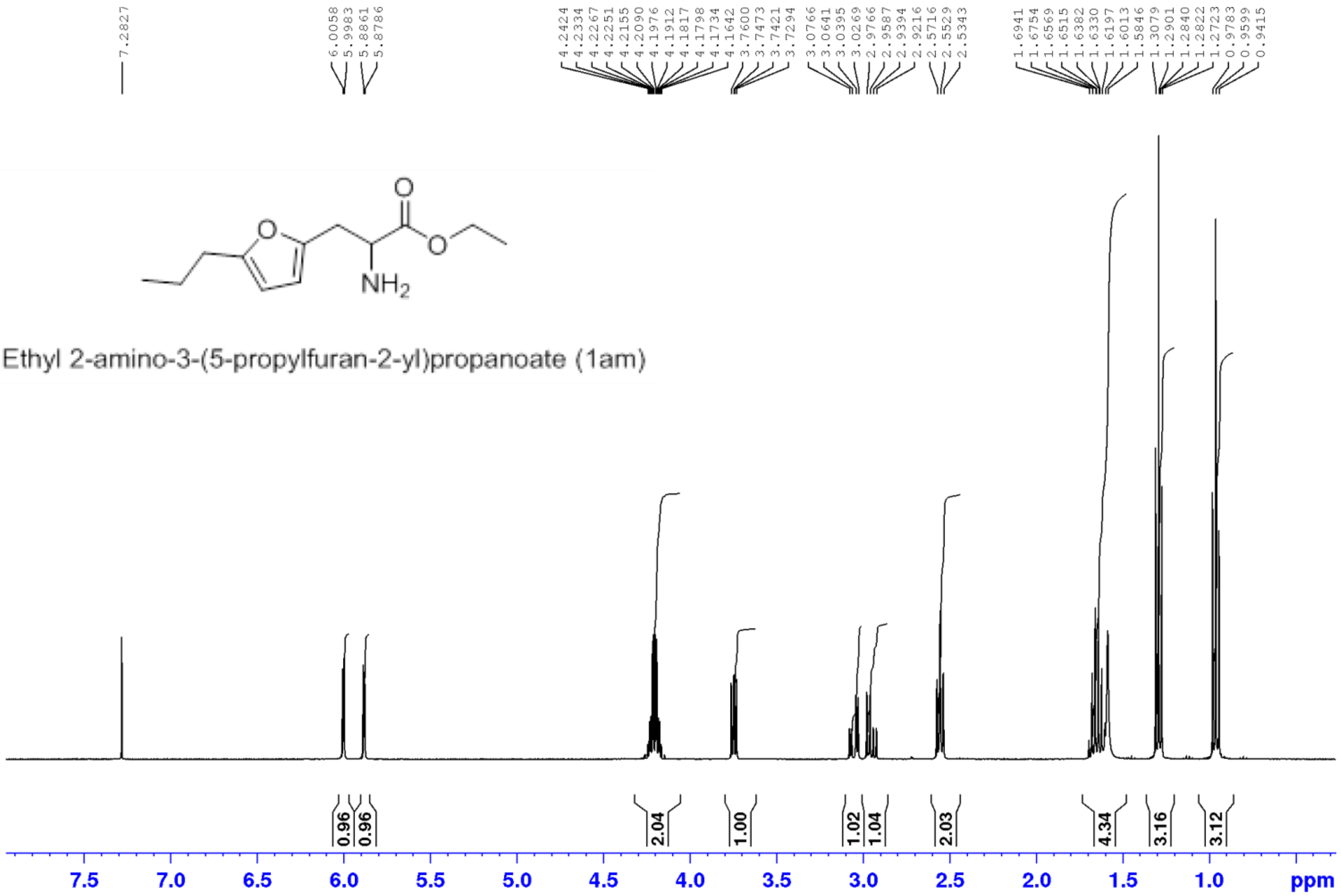

Ethyl 2-amino-3-(5-propylfuran-2-yl)propanoate (1am)

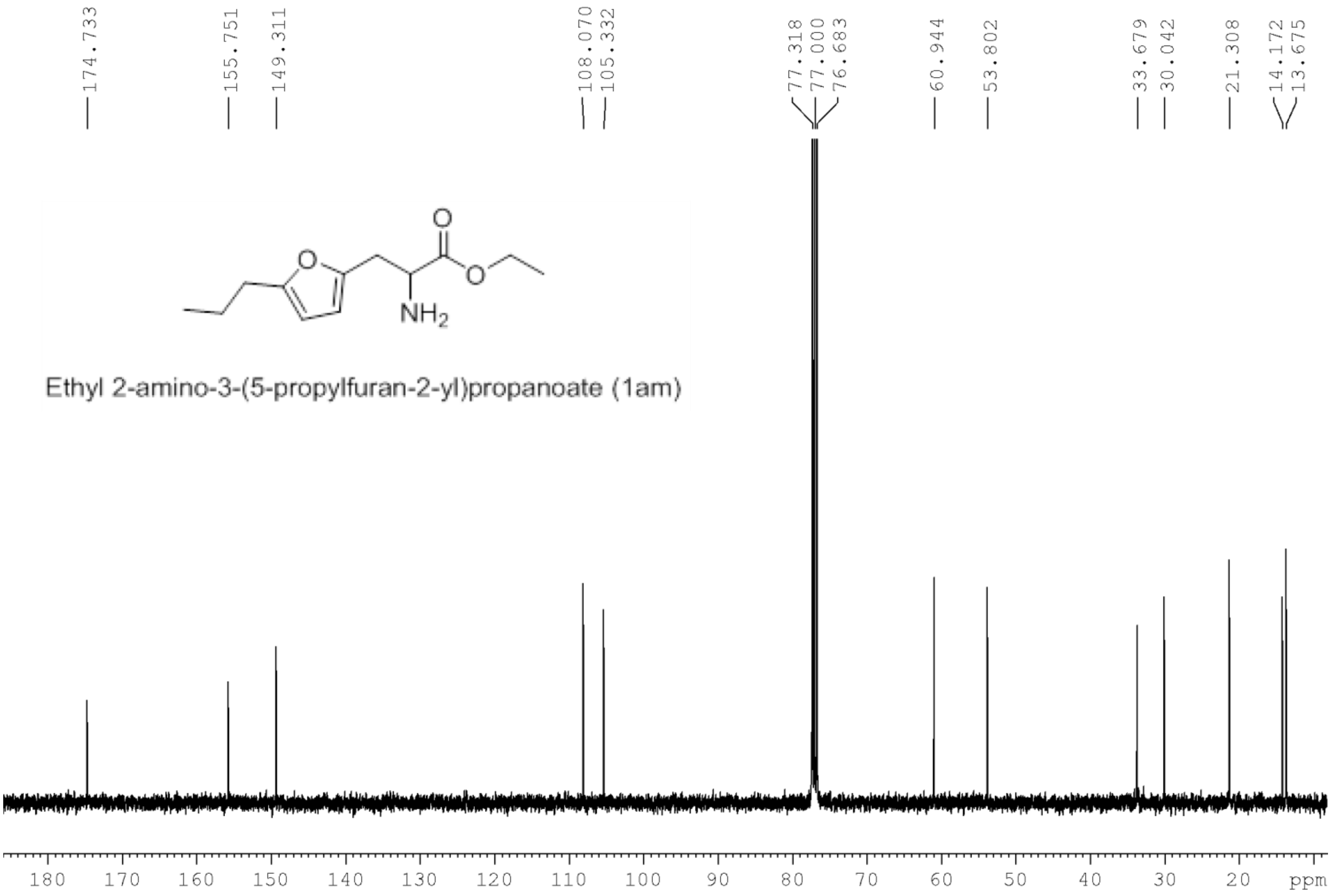

Ethyl 2-amino-3-(5-propylfuran-2-yl)propanoate (1am)

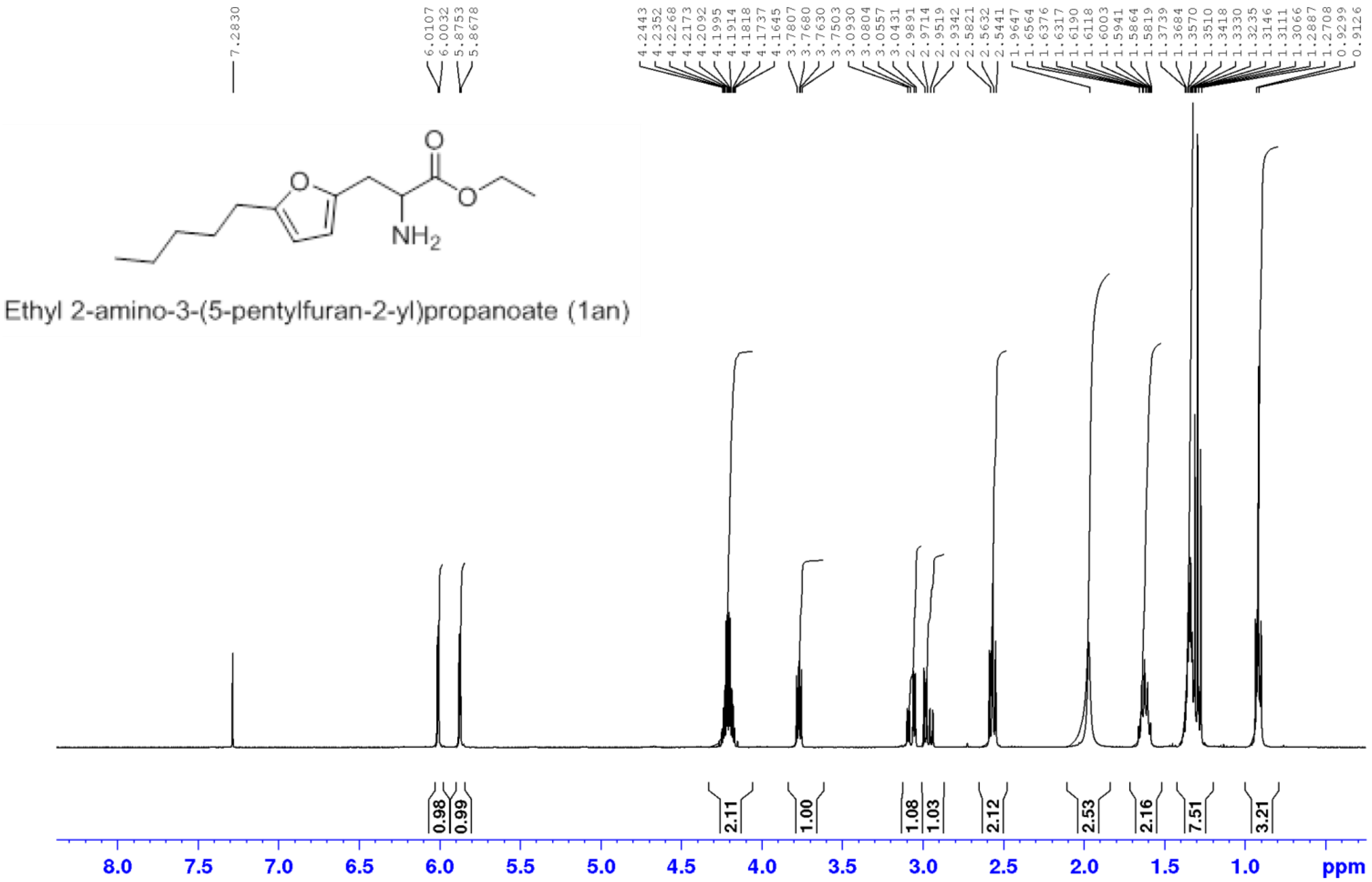
7.2830
6.0107
6.0032
5.8753
5.8678
4.2443
4.2352
4.2268
4.2173
4.2092
4.1995
4.1914
4.1818
4.1737
4.1645
3.7807
3.7680
3.7630
3.7503
3.0930
3.0804
3.0557
3.0431
2.9891
2.9714
2.9519
2.9342
2.5821
2.5632
2.5441
1.9647
1.6564
1.6376
1.6317
1.6190
1.6118
1.6003
1.5941
1.5864
1.5819
1.3739
1.3684
1.3570
1.3510
1.3418
1.3330
1.3235
1.3146
1.3111
1.3066
1.2887
1.2708
0.9299
0.9126
O
NH2
Ethyl 2-amino-3-(5-pentylfuran-2-yl)propanoate (1an)
0.98
0.99
2.11
1.00
1.08
1.03
2.12
2.53
2.16
7.51
3.21
8.0
7.5
7.0
6.5
6.0
5.5
5.0
4.5
4.0
3.5
3.0
2.5
2.0
1.5
1.0
ppm
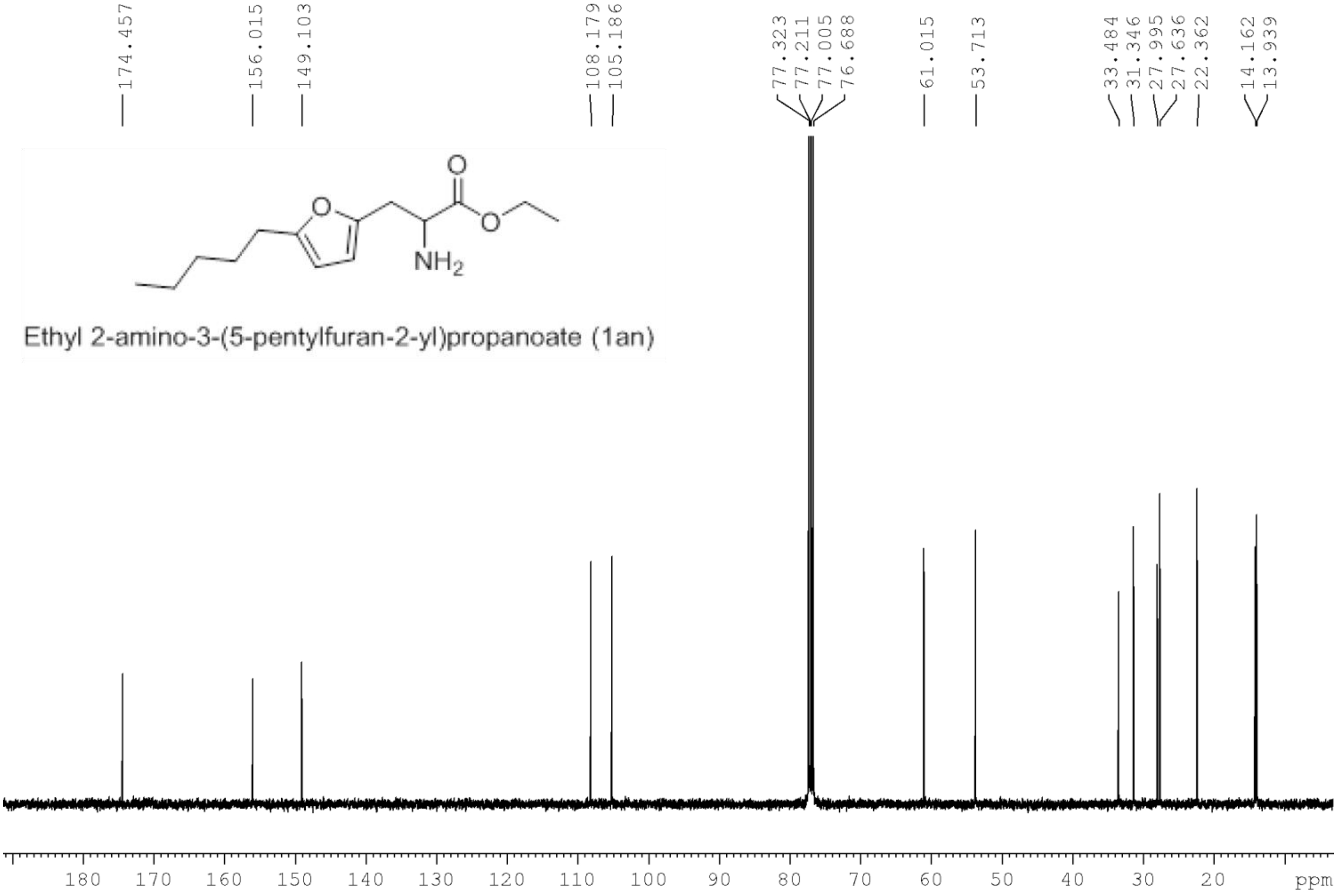
174.457
156.015
149.103
108.179
105.186
77.323
77.211
77.005
76.688
61.015
53.713
33.484
31.346
27.995
27.636
22.362
14.162
13.939
O
NH2
Ethyl 2-amino-3-(5-pentylfuran-2-yl)propanoate (1an)
180
170
160
150
140
130
120
110
100
90
80
70
60
50
40
30
20
ppm

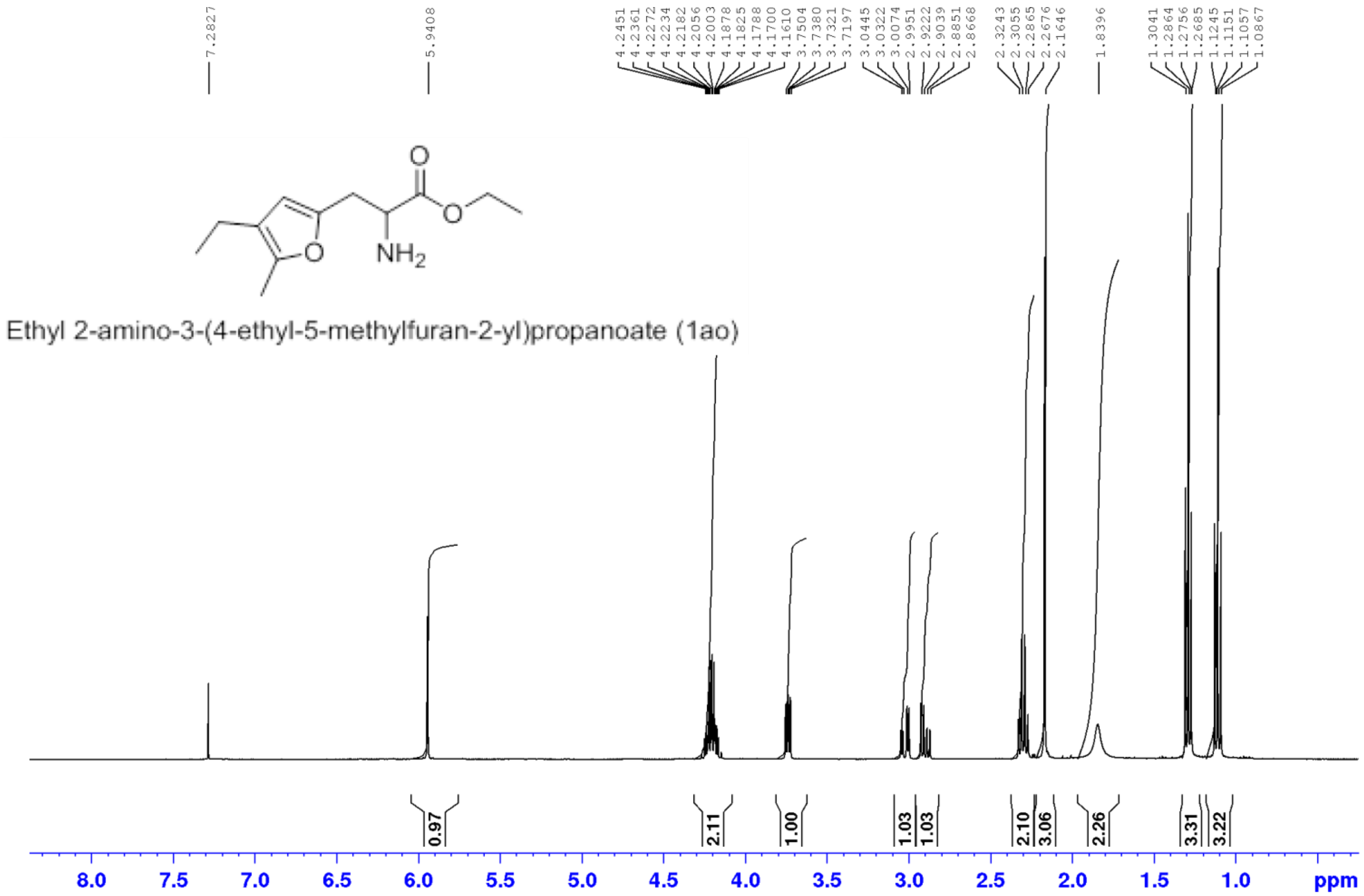

Ethyl 2-amino-3-(4-ethyl-5-methylfuran-2-yl)propanoate (1ao)

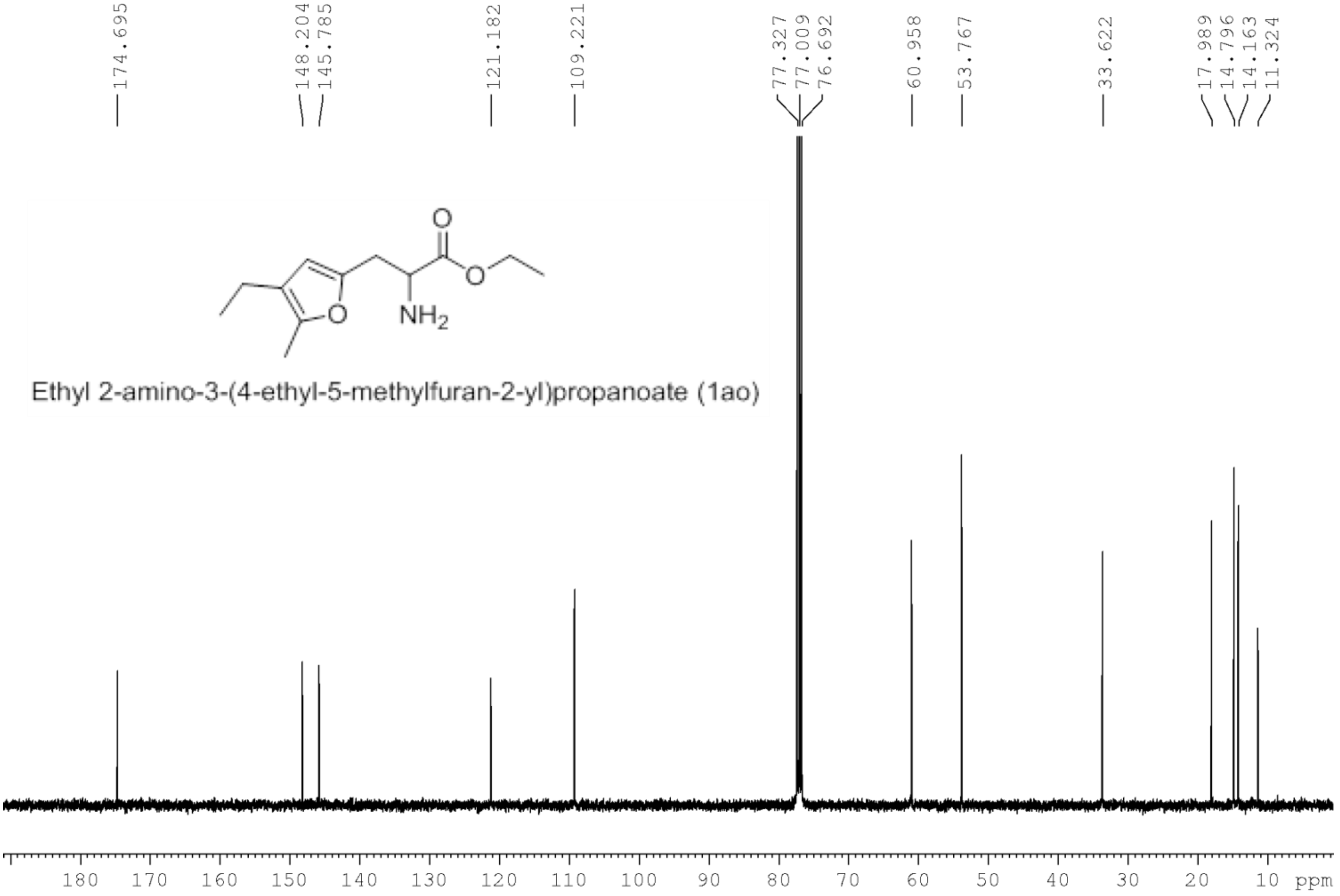

Ethyl 2-amino-3-(4-ethyl-5-methylfuran-2-yl)propanoate (1ao)

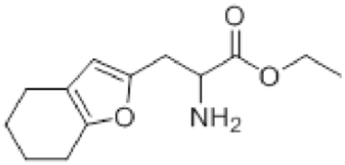

Ethyl 2-amino-3-(4,5,6,7-tetrahydrobenzofuran-2-yl)propanoate (1ap)

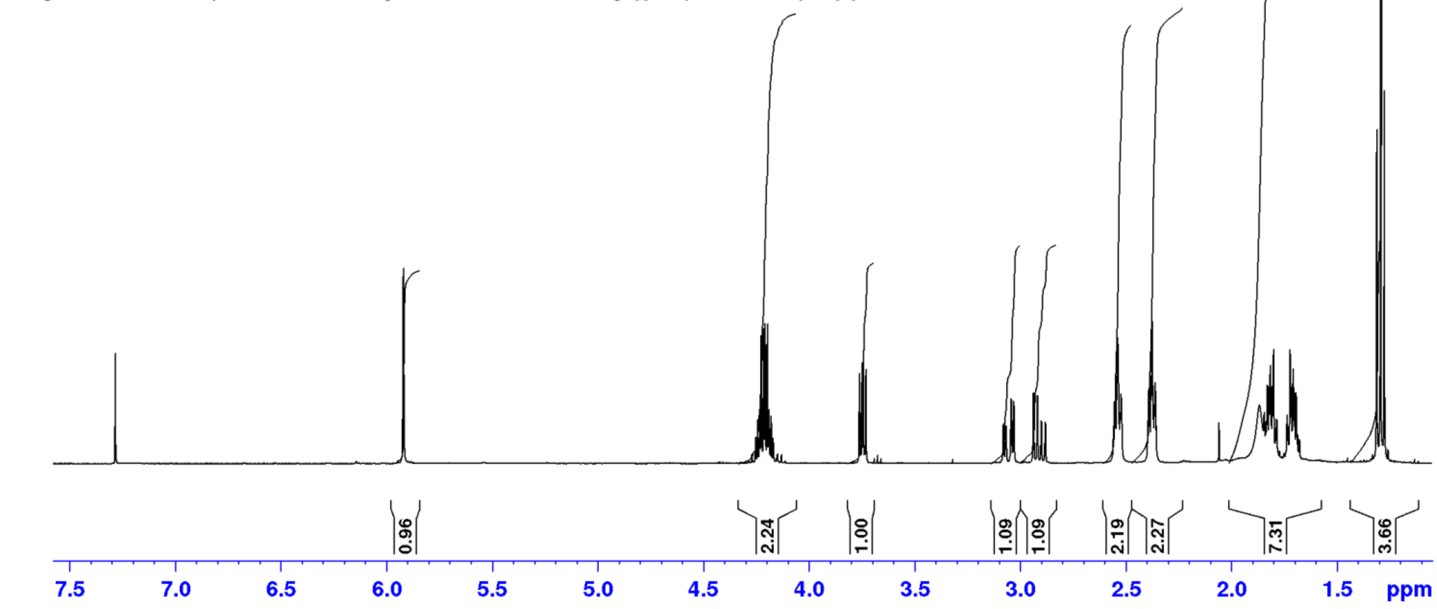

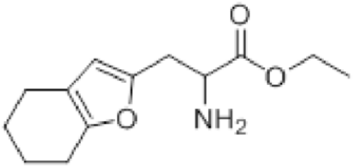

Ethyl 2-amino-3-(4,5,6,7-tetrahydrobenzofuran-2-yl)propanoate (1ap)

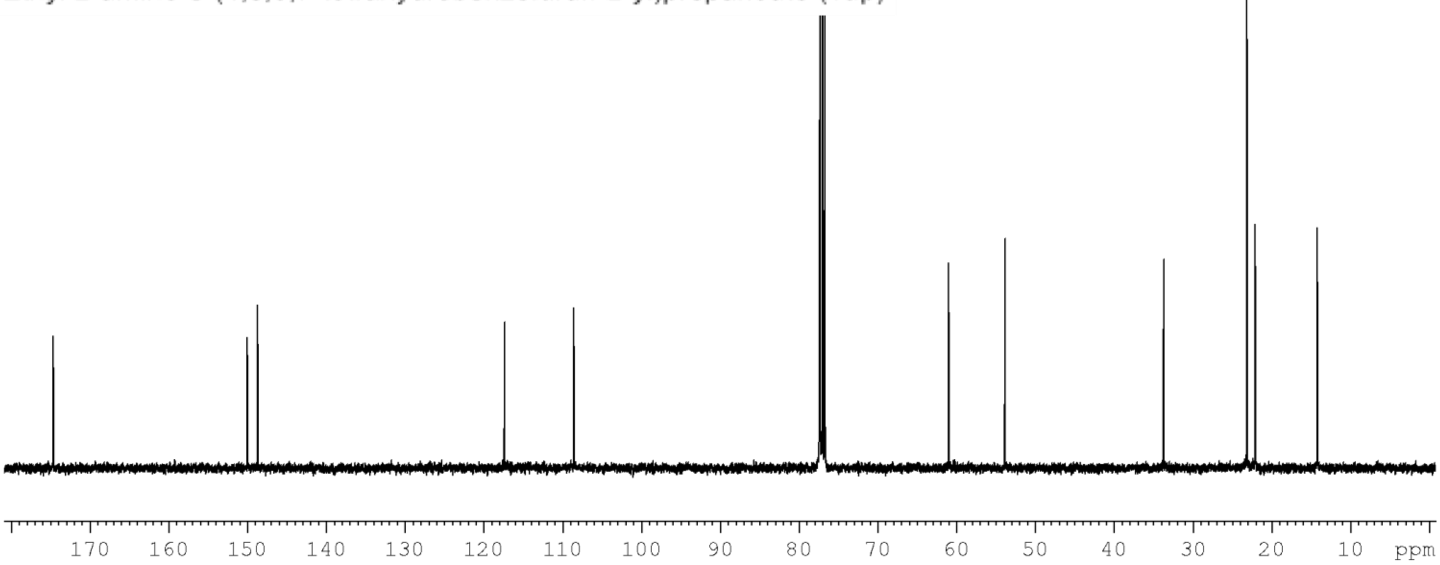

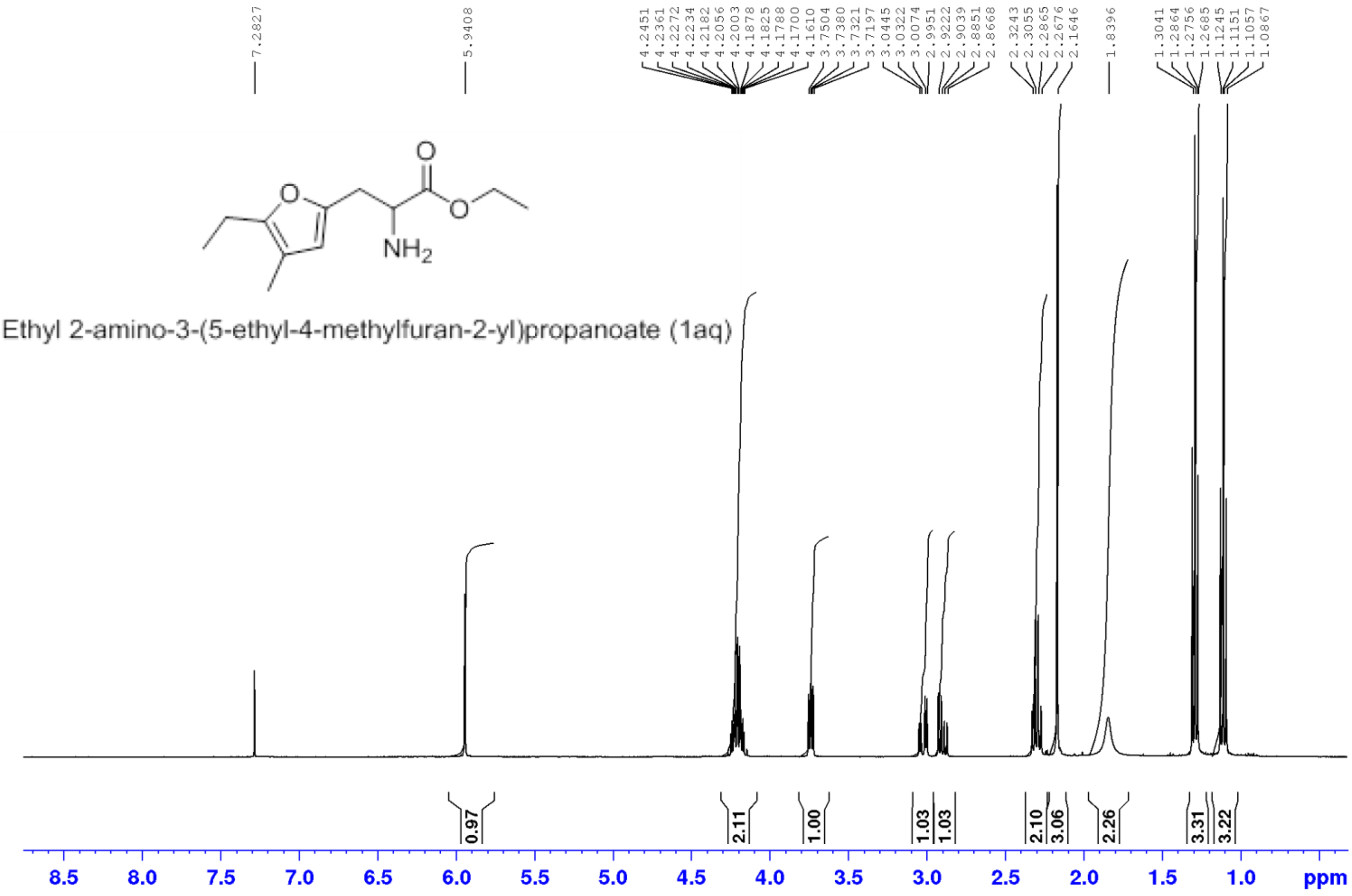

Ethyl 2-amino-3-(5-ethyl-4-methylfuran-2-yl)propanoate (1aq)

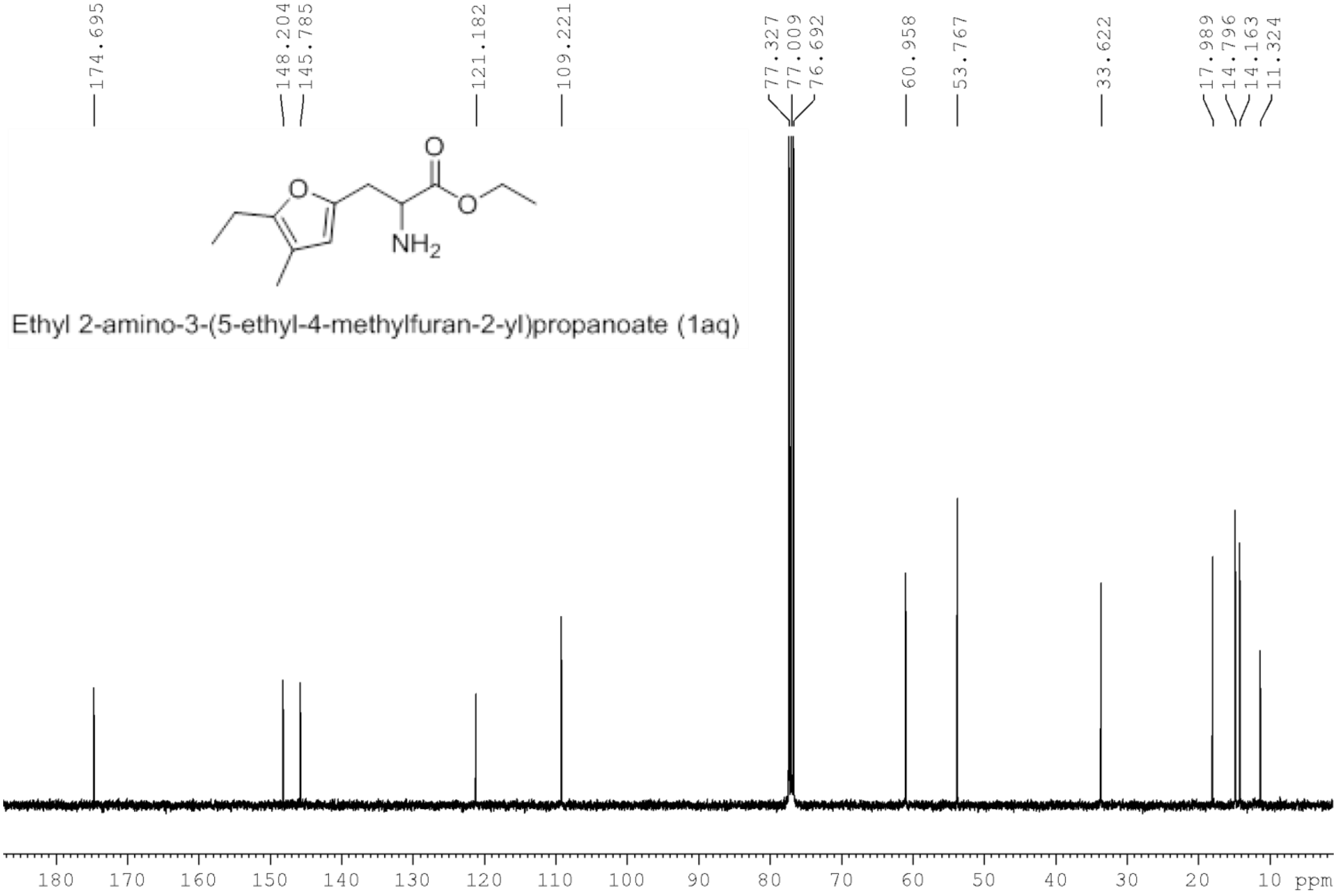

Ethyl 2-amino-3-(5-ethyl-4-methylfuran-2-yl)propanoate (1aq)

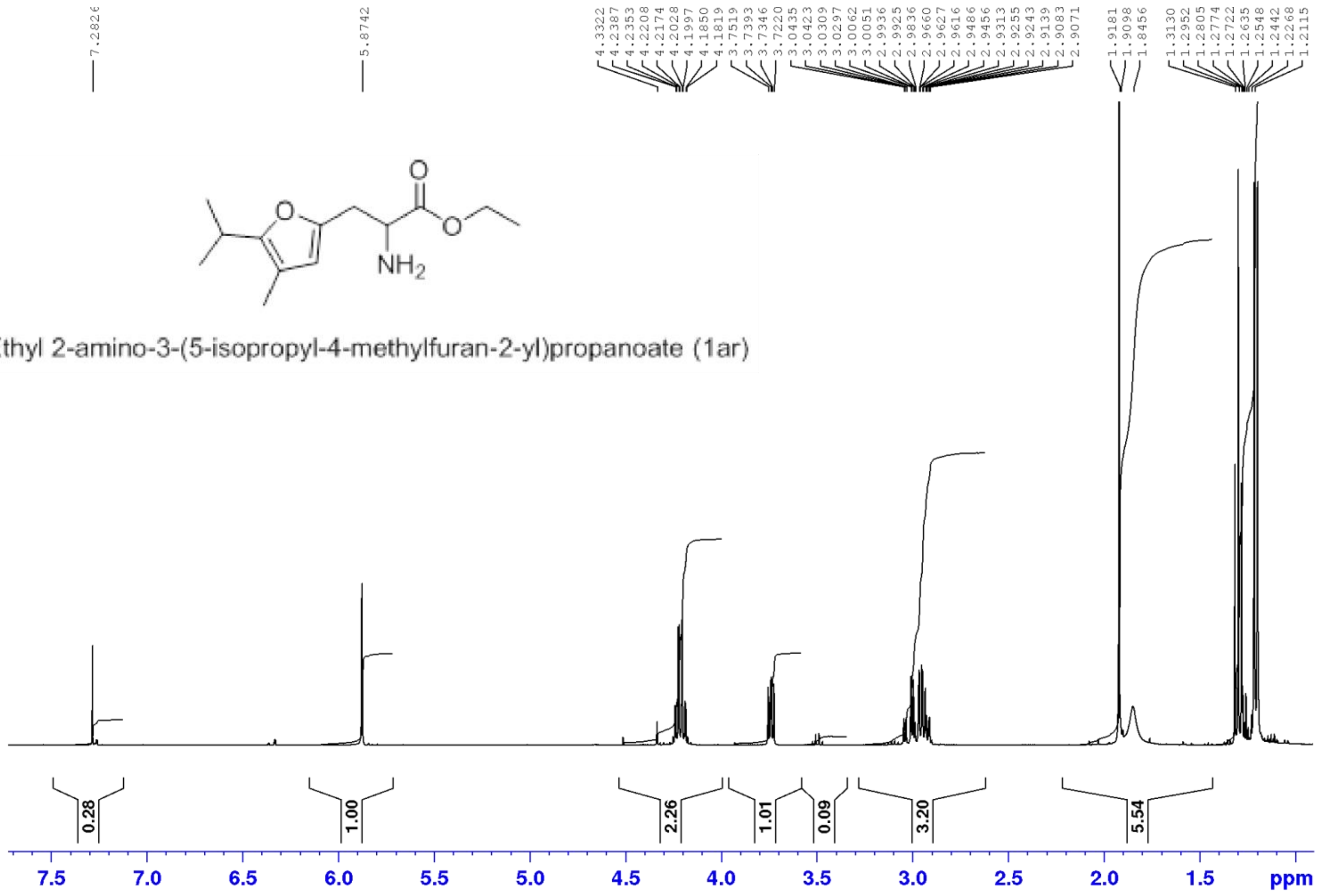

Ethyl 2-amino-3-(5-isopropyl-4-methylfuran-2-yl)propanoate (1ar)

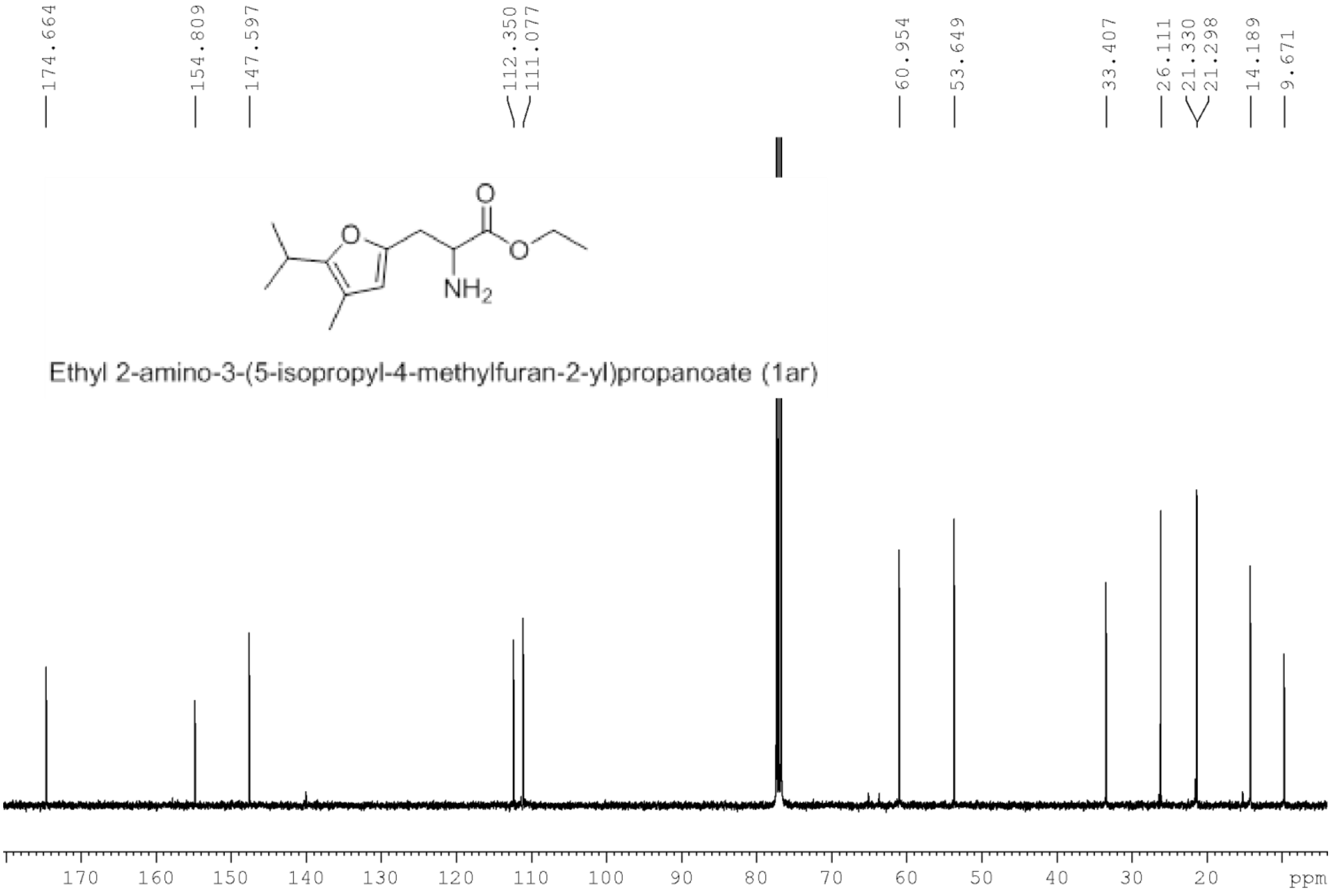

Ethyl 2-amino-3-(5-isopropyl-4-methylfuran-2-yl)propanoate (1ar)

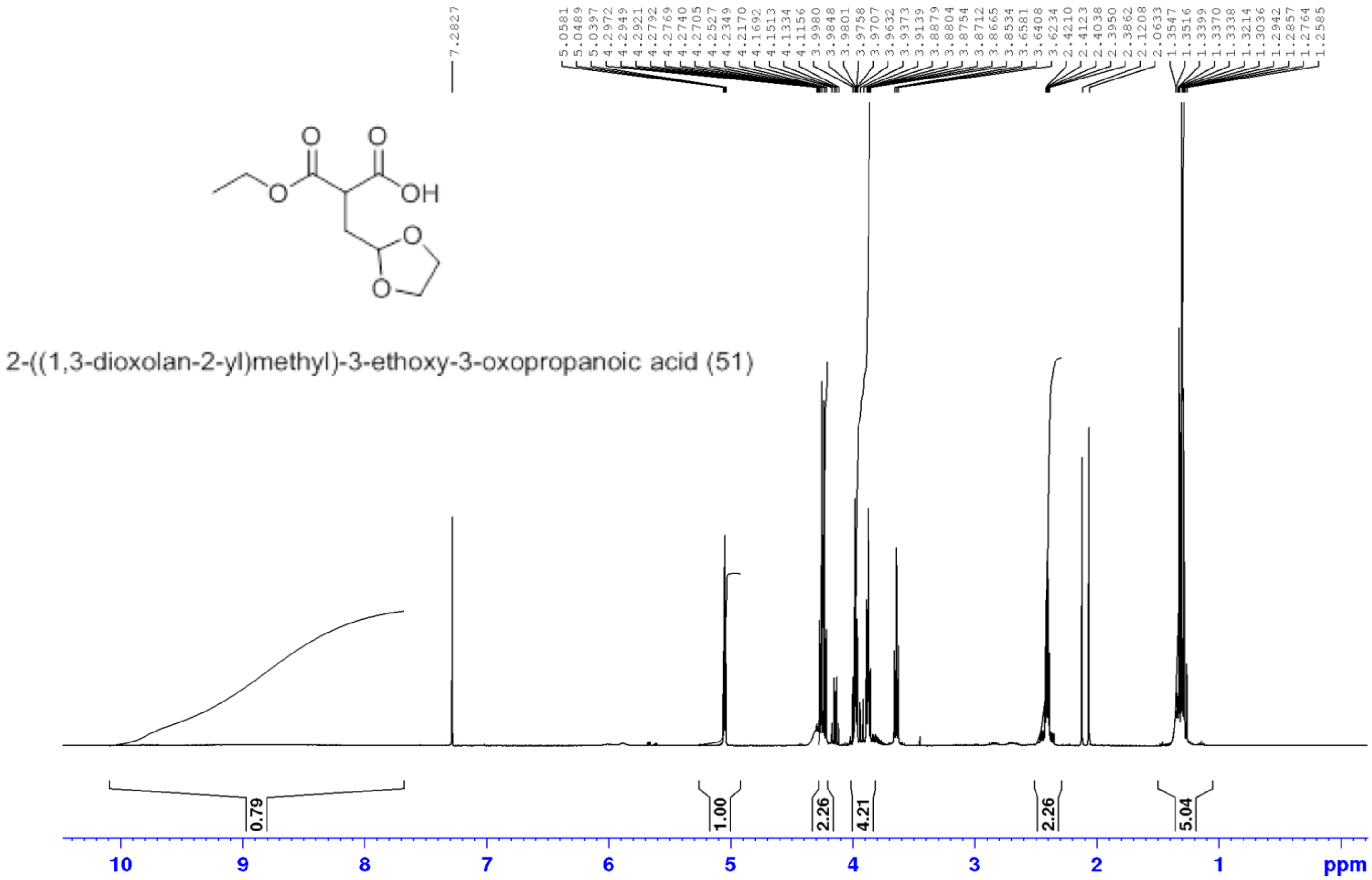
7.2827
5.0581 5.0489 5.0397 4.2972 4.2949 4.2921 4.2792 4.2769 4.2740 4.2705 4.2527 4.2349 4.2170 4.1692 4.1513 4.1334 4.1156 3.9980 3.9848 3.9801 3.9758 3.9707 3.9632 3.9373 3.9139 3.8879 3.8804 3.8754 3.8712 3.8665 3.8534 3.6581 3.6408 3.6234 2.4210 2.4123 2.4038 2.3950 2.3862 2.1208 2.0633 1.3547 1.3516 1.3399 1.3370 1.3338 1.3214 1.3036 1.2942 1.2857 1.2764 1.2585
OH
O
2-((1,3-dioxolan-2-yl)methyl)-3-ethoxy-3-oxopropanoic acid (51)
0.79
1.00
2.26
4.21
2.26
5.04
10 9 8 7 6 5 4 3 2 1 ppm

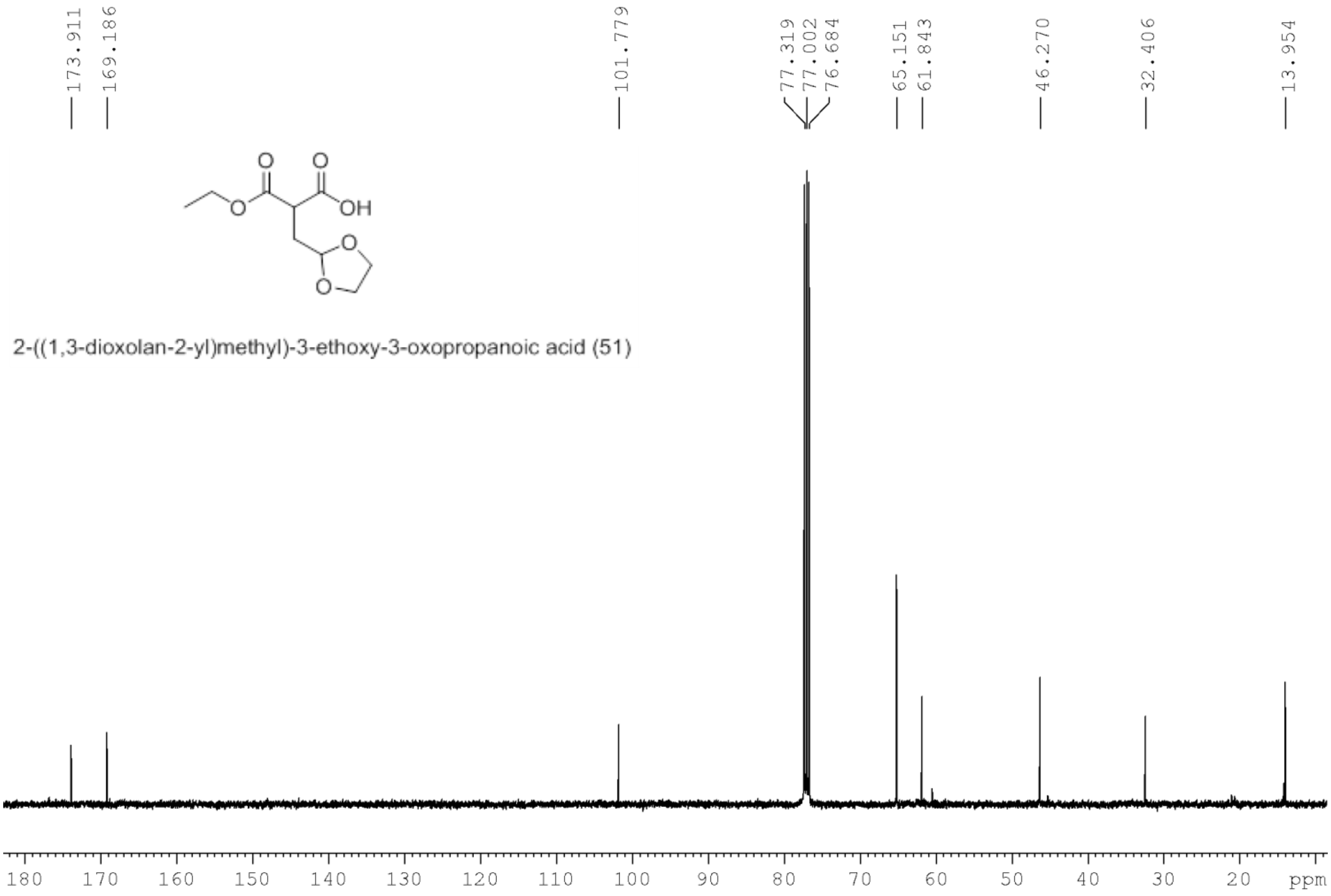
173.911
169.186
101.779
77.319
77.002
76.684
65.151
61.843
46.270
32.406
13.954
OH
O
2-((1,3-dioxolan-2-yl)methyl)-3-ethoxy-3-oxopropanoic acid (51)
180 170 160 150 140 130 120 110 100 90 80 70 60 50 40 30 20 ppm

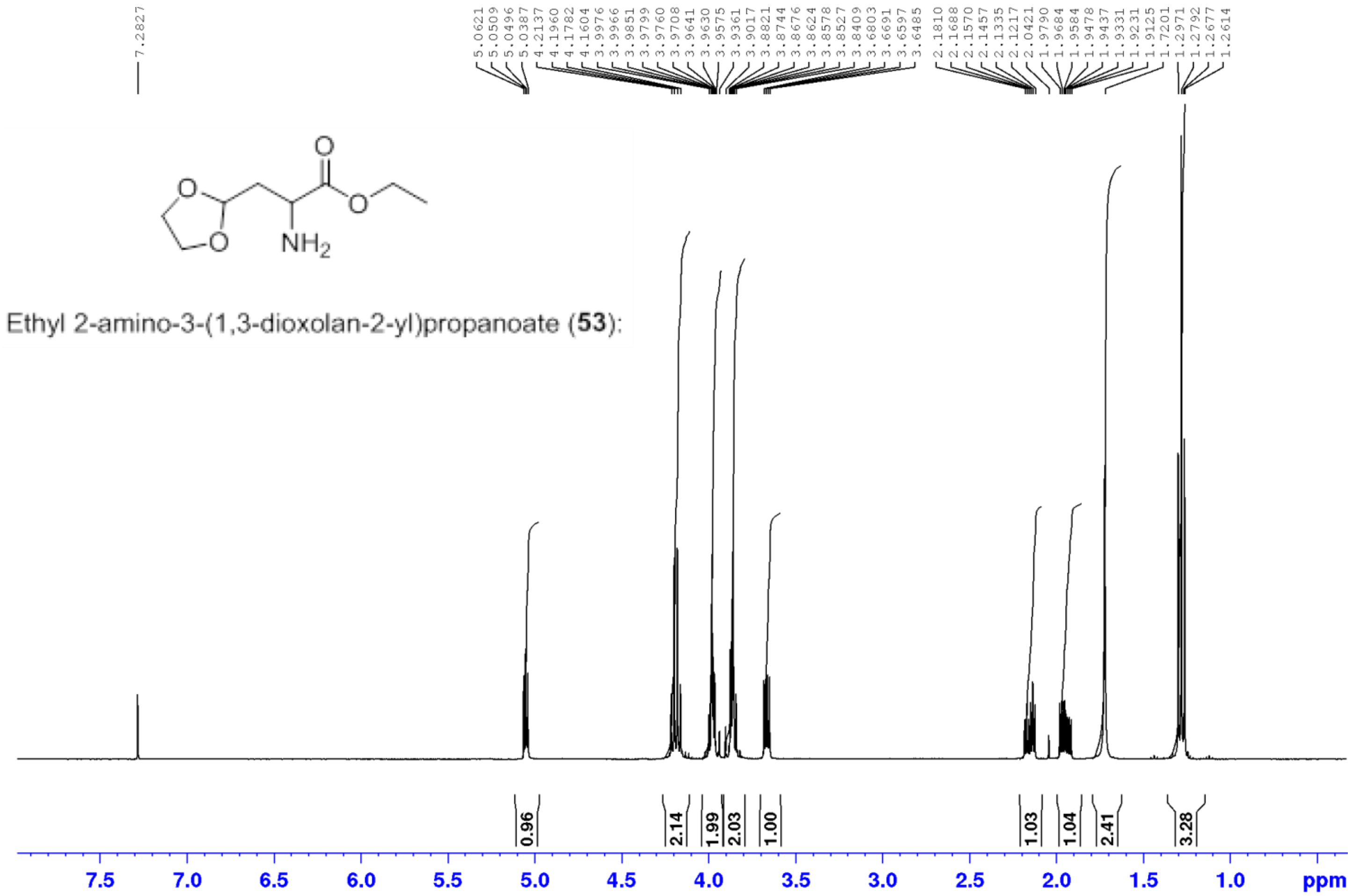
7.2827
5.0621 5.0509 5.0496 5.0387 4.2137 4.1960 4.1782 4.1604 3.9976 3.9966 3.9851 3.9799 3.9760 3.9708 3.9641 3.9630 3.9575 3.9361 3.9017 3.8821 3.8744 3.8676 3.8624 3.8578 3.8527 3.8409 3.6803 3.6691 3.6597 3.6485
2.1810 2.1688 2.1570 2.1457 2.1335 2.1217 2.0421 1.9790 1.9684 1.9584 1.9478 1.9437 1.9331 1.9231 1.9125 1.7201 1.2971 1.2792 1.2677 1.2614
NH2
Ethyl 2-amino-3-(1,3-dioxolan-2-yl)propanoate (53):
0.96
2.14
1.99
2.03
1.00
1.03
1.04
2.41
3.28
7.5 7.0 6.5 6.0 5.5 5.0 4.5 4.0 3.5 3.0 2.5 2.0 1.5 1.0 ppm

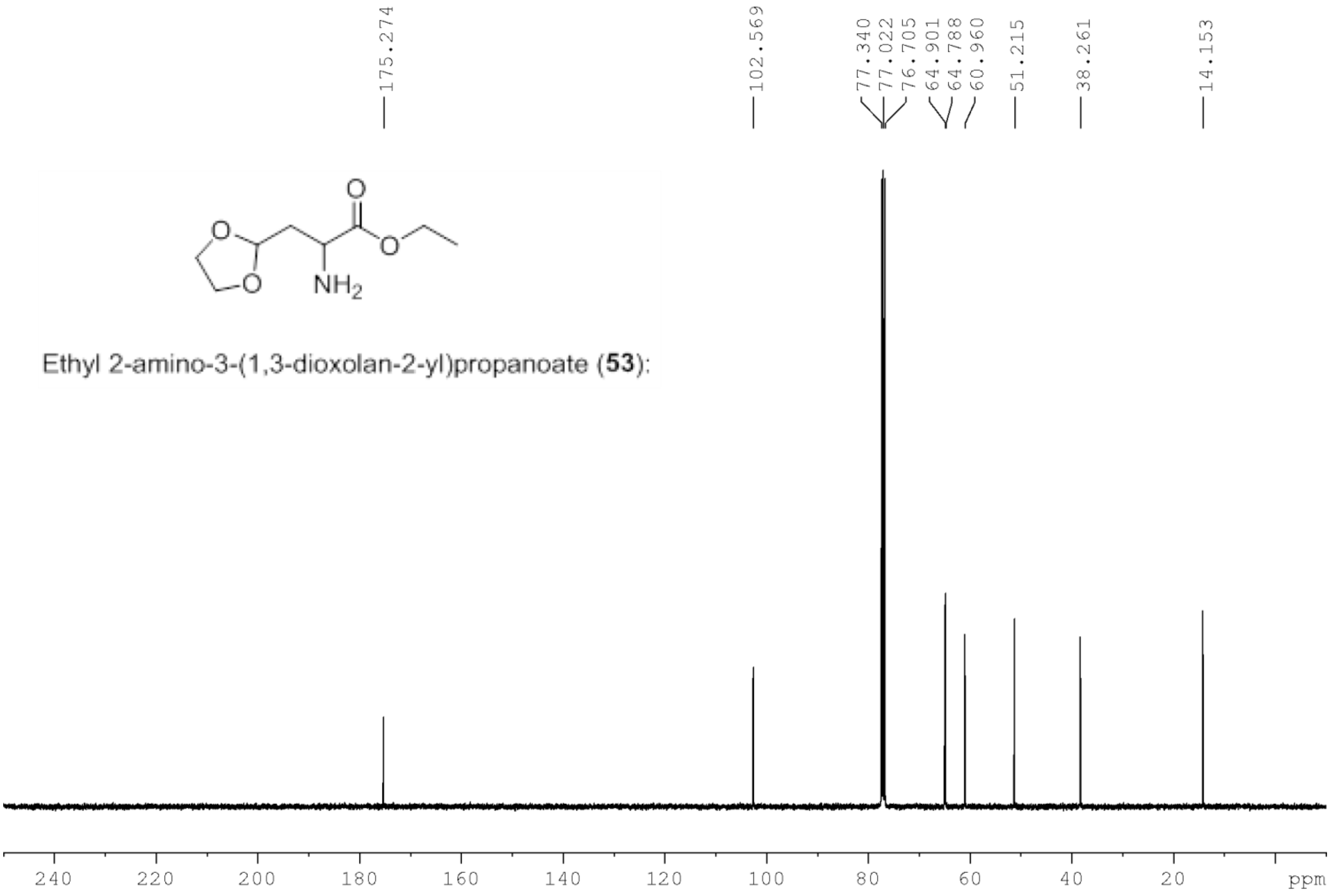
175.274
102.569
77.340 77.022 76.705 64.901 64.788 60.960
51.215
38.261
14.153
NH2
Ethyl 2-amino-3-(1,3-dioxolan-2-yl)propanoate (53):
240 220 200 180 160 140 120 100 80 60 40 20 ppm

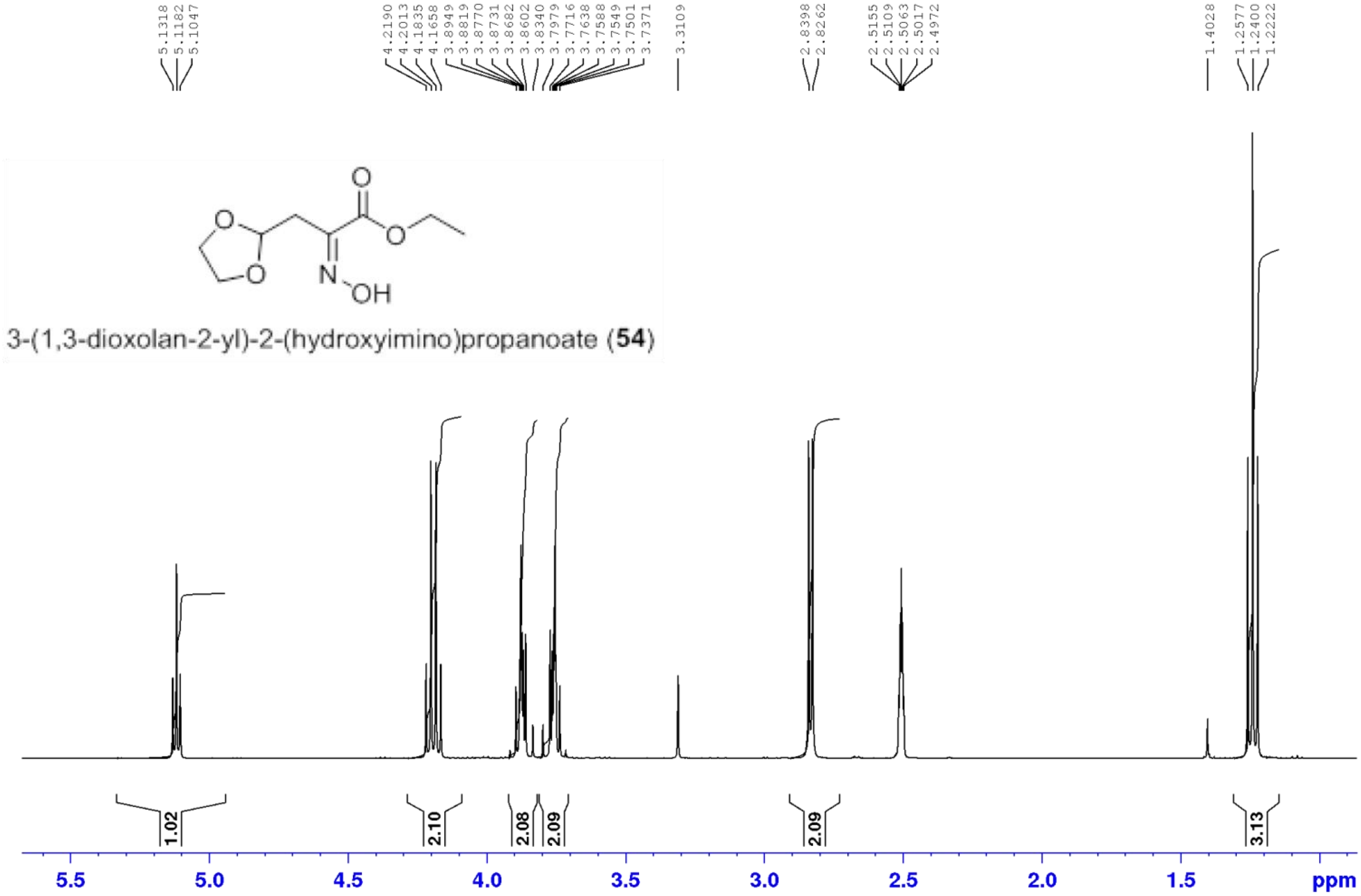
5.1318
5.1182
5.1047
4.2190
4.2013
4.1835
4.1658
3.8949
3.8819
3.8770
3.8731
3.8682
3.8602
3.8340
3.7979
3.7716
3.7638
3.7588
3.7549
3.7501
3.7371
3.3109
2.8398
2.8262
2.5155
2.5109
2.5063
2.5017
2.4972
1.4028
1.2577
1.2400
1.2222
3-(1,3-dioxolan-2-yl)-2-(hydroxyimino)propanoate (**54**)
1.02
2.10
2.08
2.09
2.09
3.13
5.5
5.0
4.5
4.0
3.5
3.0
2.5
2.0
1.5
ppm

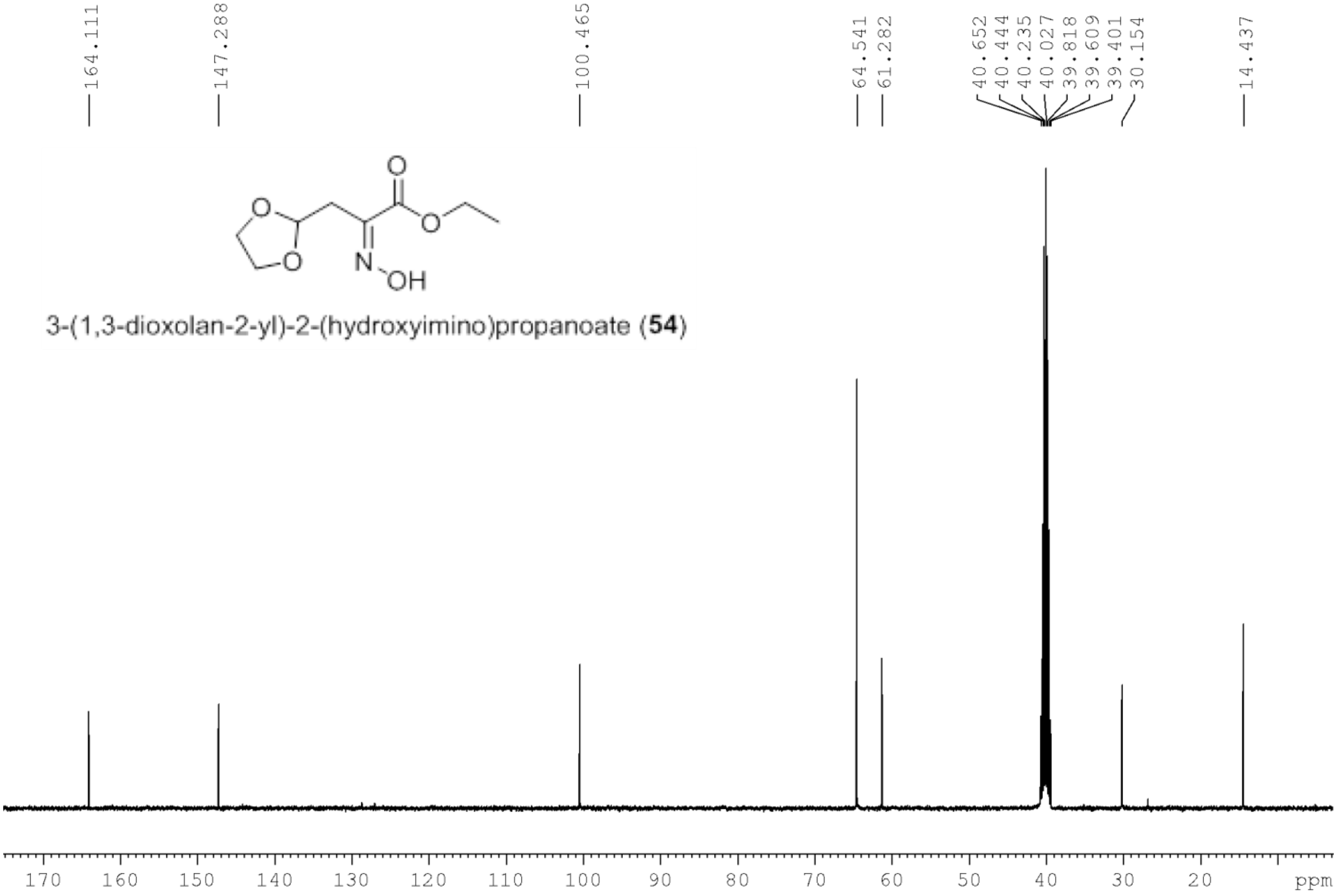
164.111
147.288
100.465
64.541
61.282
40.652
40.444
40.235
40.027
39.818
39.609
39.401
30.154
14.437
3-(1,3-dioxolan-2-yl)-2-(hydroxyimino)propanoate (**54**)
170
160
150
140
130
120
110
100
90
80
70
60
50
40
30
20
ppm

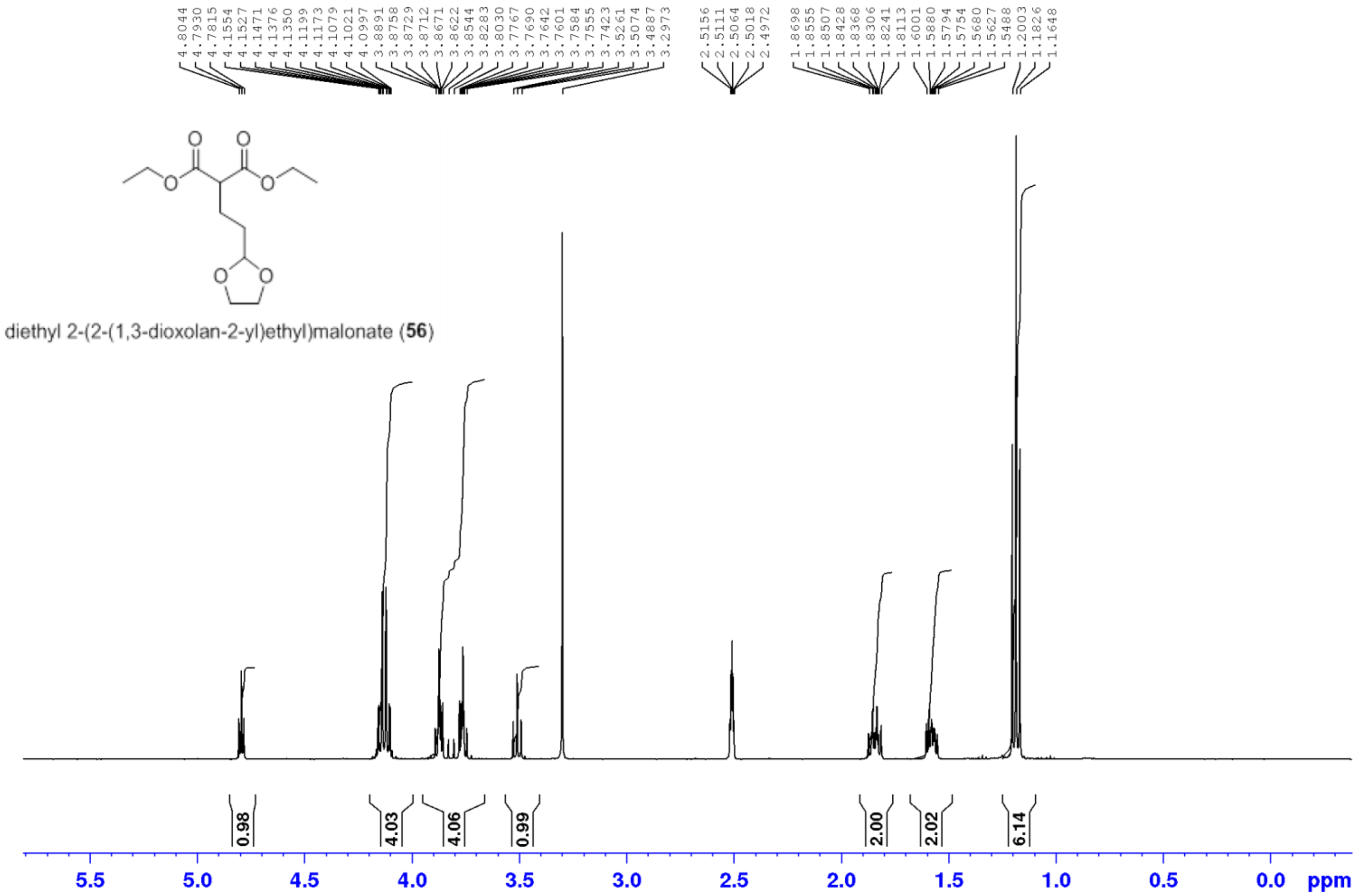

diethyl 2-(2-(1,3-dioxolan-2-yl)ethyl)malonate (**56**)

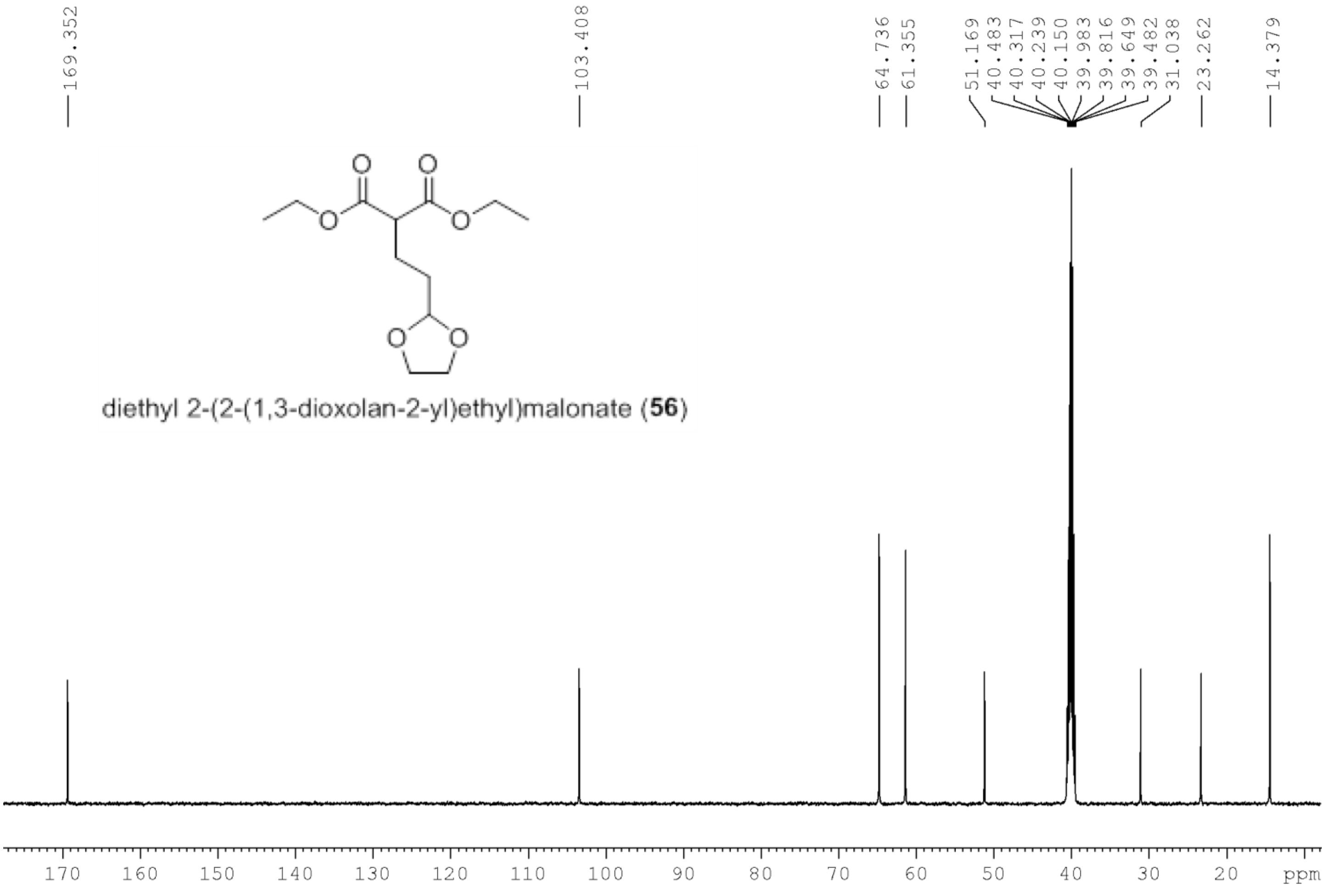

diethyl 2-(2-(1,3-dioxolan-2-yl)ethyl)malonate (**56**)

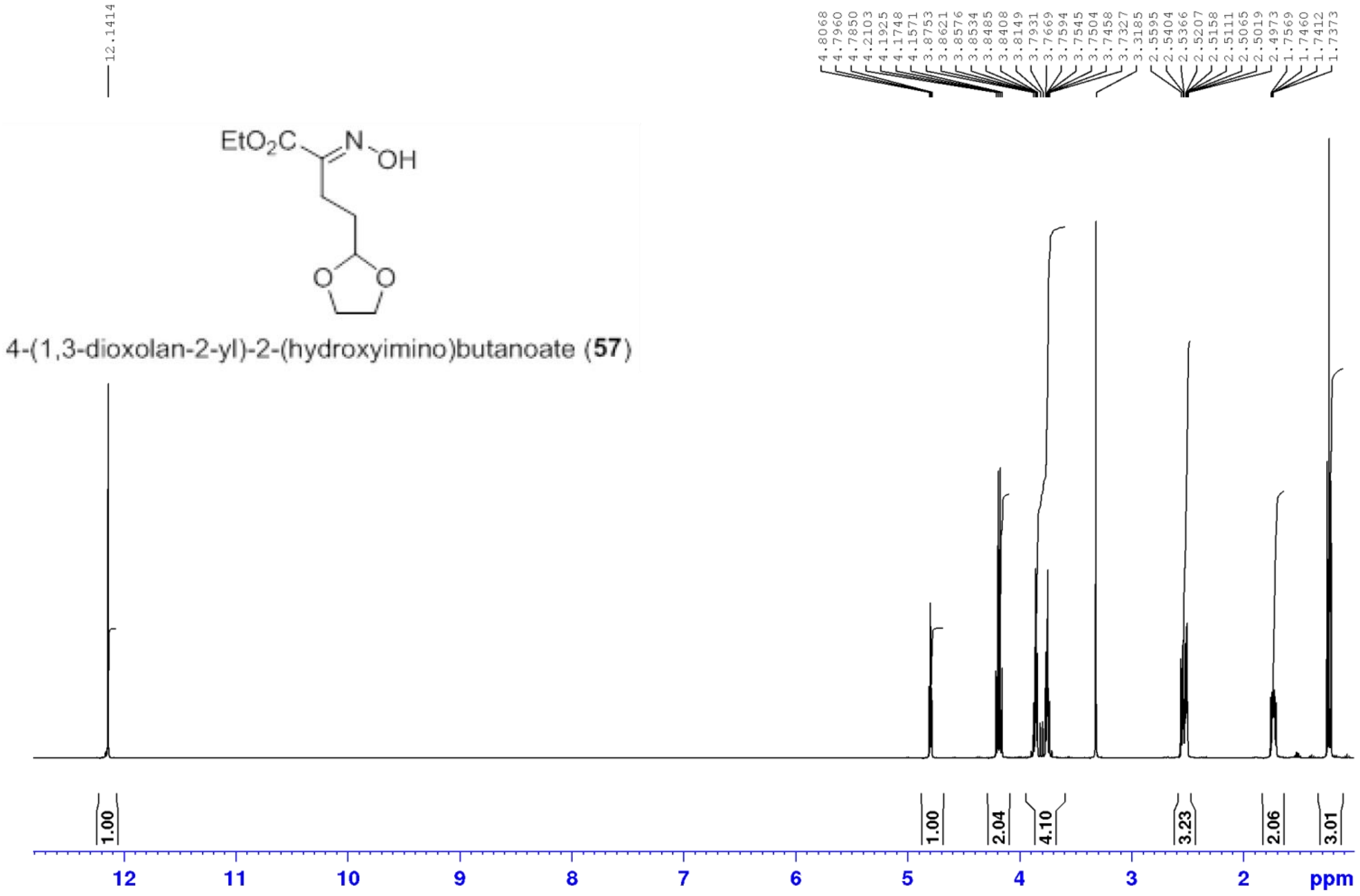
12.1414
4.8068 4.7960 4.7850 4.2103 4.1925 4.1748 4.1571 3.8753 3.8621 3.8576 3.8534 3.8485 3.8408 3.8149 3.7931 3.7669 3.7594 3.7545 3.7504 3.7458 3.7327 3.3185 2.5595 2.5404 2.5366 2.5207 2.5158 2.5111 2.5065 2.5019 2.4973 1.7569 1.7460 1.7412 1.7373
EtO_2C
N
OH
O
O
4-(1,3-dioxolan-2-yl)-2-(hydroxyimino)butanoate (57)
1.00
1.00
2.04
4.10
3.23
2.06
3.01
12
11
10
9
8
7
6
5
4
3
2
ppm

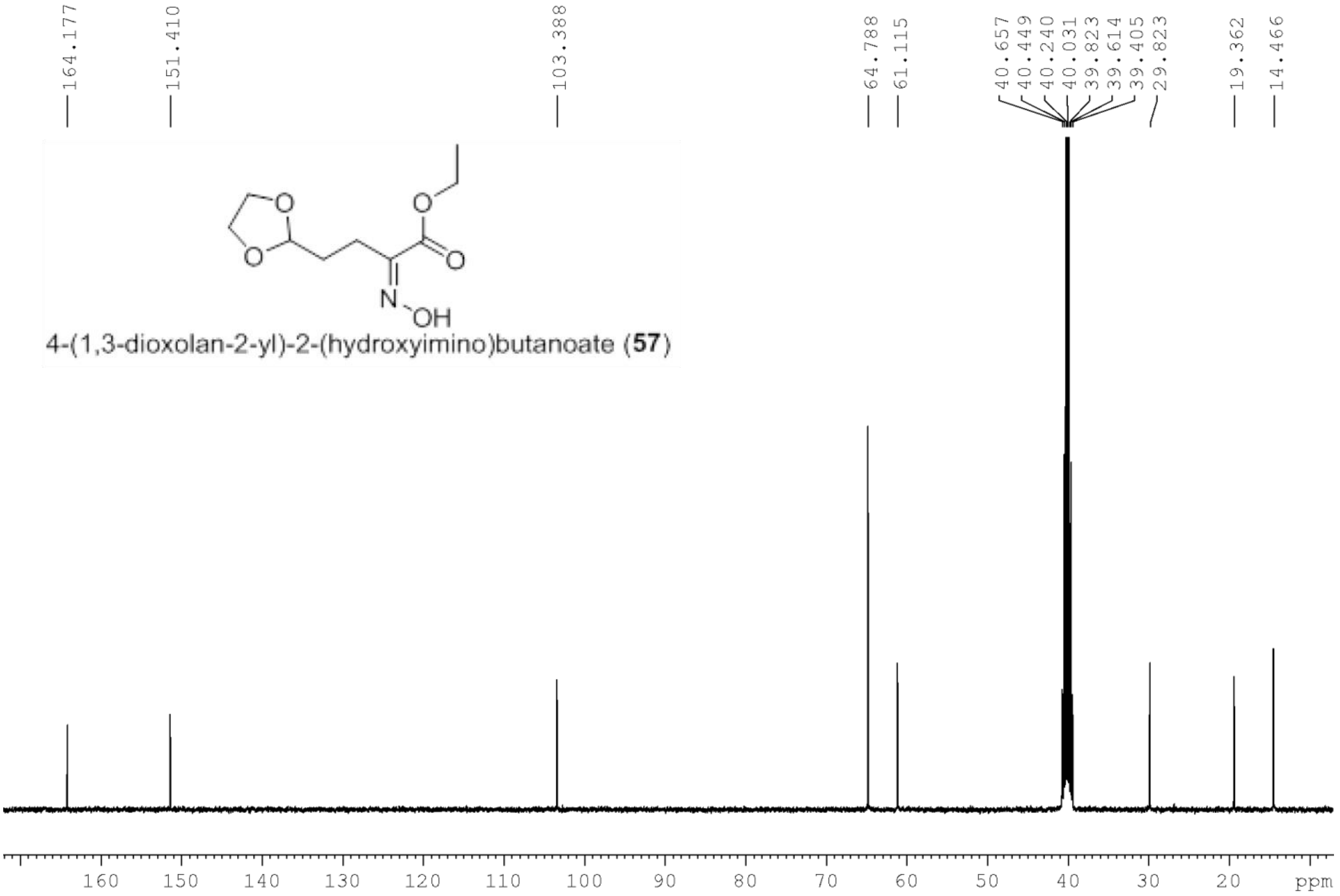
164.177
151.410
103.388
64.788
61.115
40.657 40.449 40.240 40.031 39.823 39.614 39.405 29.823
19.362
14.466
O
O
O
O
N
OH
4-(1,3-dioxolan-2-yl)-2-(hydroxyimino)butanoate (57)
160
150
140
130
120
110
100
90
80
70
60
50
40
30
20
ppm

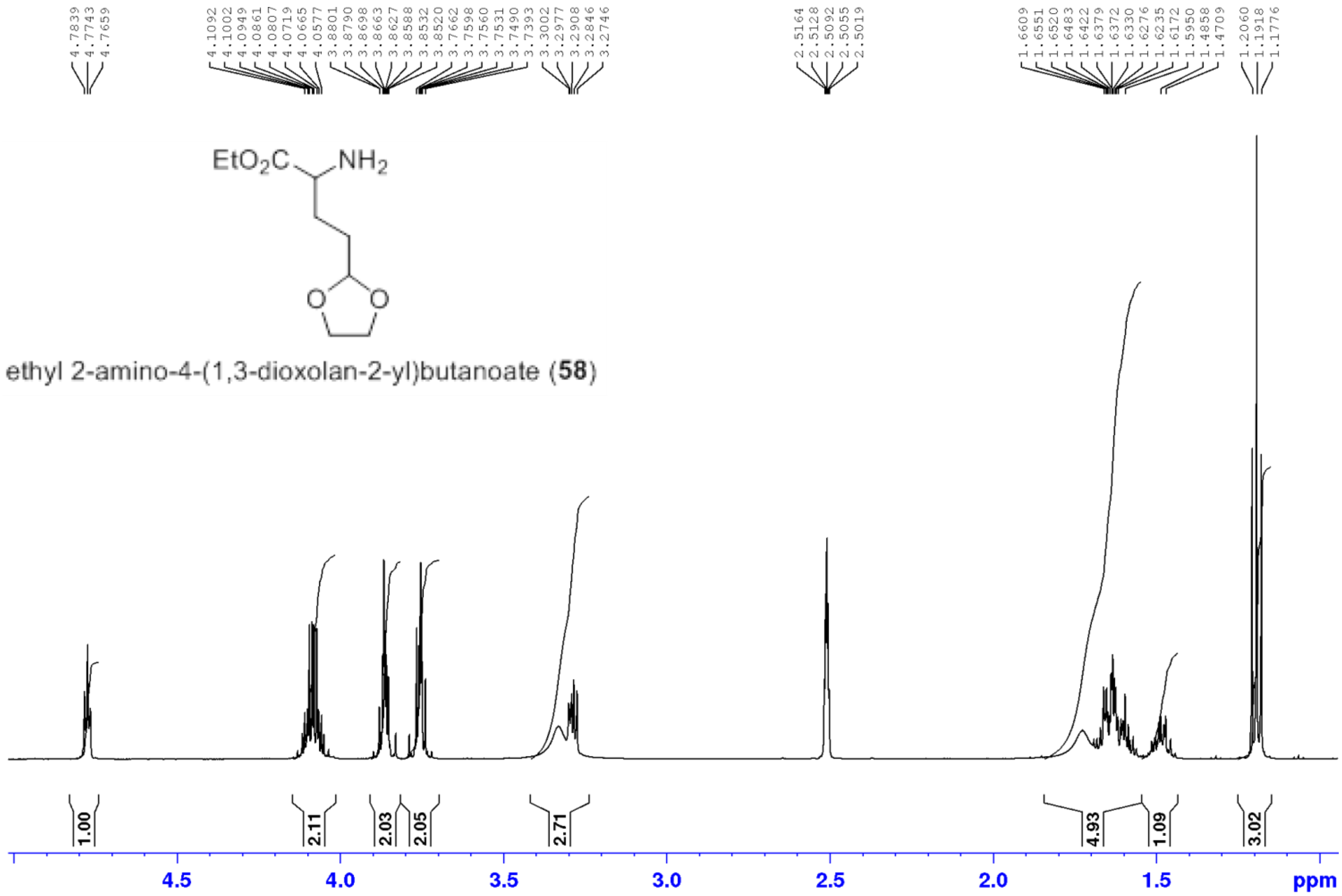

ethyl 2-amino-4-(1,3-dioxolan-2-yl)butanoate (**58**)

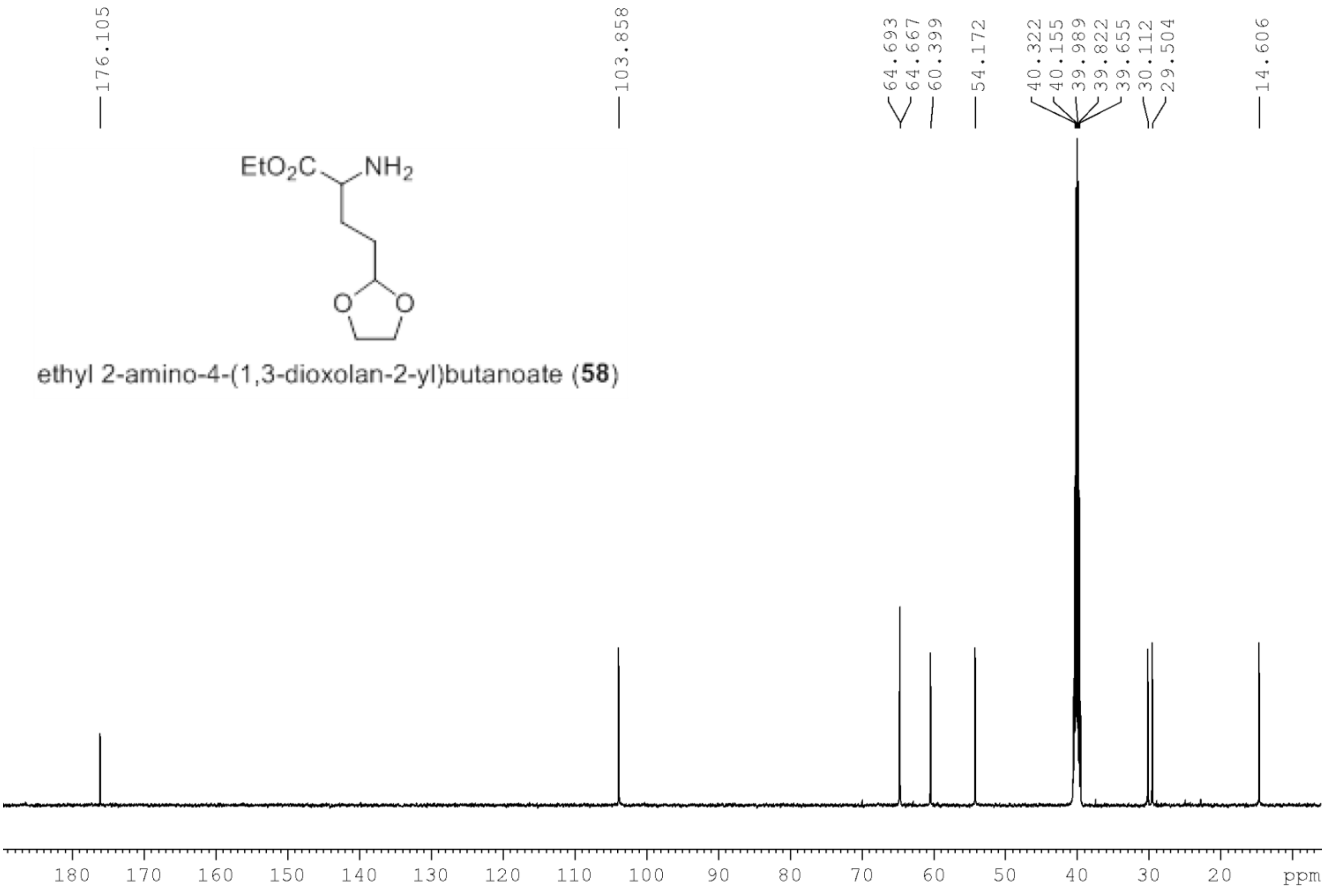

ethyl 2-amino-4-(1,3-dioxolan-2-yl)butanoate (**58**)

References

1. De Salas, C.; Heinrich, M. R. *Green Chem.* **2014,** *16*, 2982-2987.

2. Gilchrist, T. L.; Roberts, T. G. *J. Chem. Soc. Perkin. Trans. 1* **1983**, 1283-1292.

3. Gilchrist, T. L.; Hughes, D.; Stretch, W.; Chrystal, E. J. T. *J. Chem. Soc. Perkin. Trans. 1* **1987**, 2505-2509.

4. Gagnot, G.; Hervin, V.; Coutant, E. P.; Desmons, S.; Baatallah, R.; Monnot, V.; Janin, Y. L. *Beilstein J. Org. Chem.* **2018**, Submitted.

5. García-Rubia, A.; Laga, E.; Cativiela, C.; Urriolabeitia, E. P.; Gómez-Arrayás, R.; Carretero, J. C. *J. Org. Chem.* **2015,** *80*, 3321-3331.

6. Iqbal, J.; Tangellamudi, N. D.; Dulla, B.; Balasubramanian, S. *Org. Lett.* **2012,** *14*, 552-555.

7. Ansari, A. M.; Ugwu, S. O. *Synth. Commun.* **2008,** *38*, 2330-2340.

8. Holmberg, G. A.; Karlsson, M.; Ulfstedt, O.; Olli, M. *Acta Chem. Scand.* **1972,** *26*, 3483-3491.

9. Andersen, R.; Piers, E.; Nieman, J.; Coleman, J.; Roberge, M. Hemiasterlin analogs. US 20090264487, publication date: 22/10/2009.

10. Roberts, D. D. *J. Org. Chem.* **1964,** *29*, 2714-2717.

11. Tijhuis, M. W.; Herscheid, J. D. M.; Ottenheijm, H. C. J. *Synthesis* **1980**, 890-893.

12. Li, Q.; Woods, K. W.; Zhu, G.; Fischer, J. P.; Gong, J.; Li, T.; Gandhi, V.; Thomas, S. A.; Packard, G. K.; Song, X.; Abrams, J. N.; Diebold, R. B.; Dinges, J.; Hutchins, C. W.; Stoll, V. S.; Rosenberg, S. H.; Giranda, V. L. Kinase inhibitors. US 20030199511, publication date: 23/10/2003.

13. Brown, P. E.; Lewis, R. A.; Waring, M. A. *J. Chem. Soc. Perkin. Trans. I* **1990**, 2979-2988.

14. Tsukamoto, Y.; Komai, H.; Kadotani, J.; Koi, K.; Mio, S.; Takeshiba, H. 3-phenoxy-4-pyridazinol derivative and herbicide composition containing the same. WO 2003016286, publication date: 27/02/2003.

15. Lautens, M.; Fillion, E. *J. Org. Chem.* **1997,** *62*, 4418-4427.

16. Aïssa, C. *J. Org. Chem.* **2006,** *71*, 360-363.

17. Yamanaka, E.; Narushima, M.; Inukai, K.; Sakai, S. *Chem. Pharm. Bull.* **1985,** *34*, 77-81.
